# DFT calculations on the mechanism of copper-catalysed tandem arylation–cyclisation reactions of alkynes and diaryliodonium salts

**DOI:** 10.3762/bjoc.14.148

**Published:** 2018-07-12

**Authors:** Tamás Károly Stenczel, Ádám Sinai, Zoltán Novák, András Stirling

**Affiliations:** 1 Török Ignác Secondary School, Gödöllő, Hungary, Present address: St Catharine’s College, Cambridge CB2 1RL, UK; 2 ELTE "Lendület" Laboratory of Catalysis and Organic Synthesis, Eötvös Loránd University, Institute of Chemistry, Budapest, Hungary; 3 Servier Research Institute of Medicinal Chemistry, Záhony utca 7, H-1031, Budapest, Hungary; 4 Research Centre for Natural Sciences of the Hungarian Academy of Sciences, Institute of Organic Chemistry, Budapest, Hungary

**Keywords:** catalysis, DFT calculation, iodonium salts, reaction mechanism, tandem arylation–cyclisation

## Abstract

We present a computational mechanistic study on the copper(III)-catalysed carboarylation–ring closure reactions leading to the formation of functionalised heterocycles. We have performed DFT calculations along selected routes and compared their free energy profiles. The calculations considered two viable options for the underlying mechanism which differ in the order of the oxazoline ring formation and the aryl transfer steps. In our model transformation, it was found that the reaction generally features the aryl transfer–ring closing sequence and this sequence shows very limited sensitivity to the variation of the substituent of the reactants. On the basis of the mechanism the origin of the stereoselectivity is ascribed to the interaction of the Cu ion with the oxazoline oxygen driving the ring-closure step selectively.

## Introduction

**Scheme 1:**
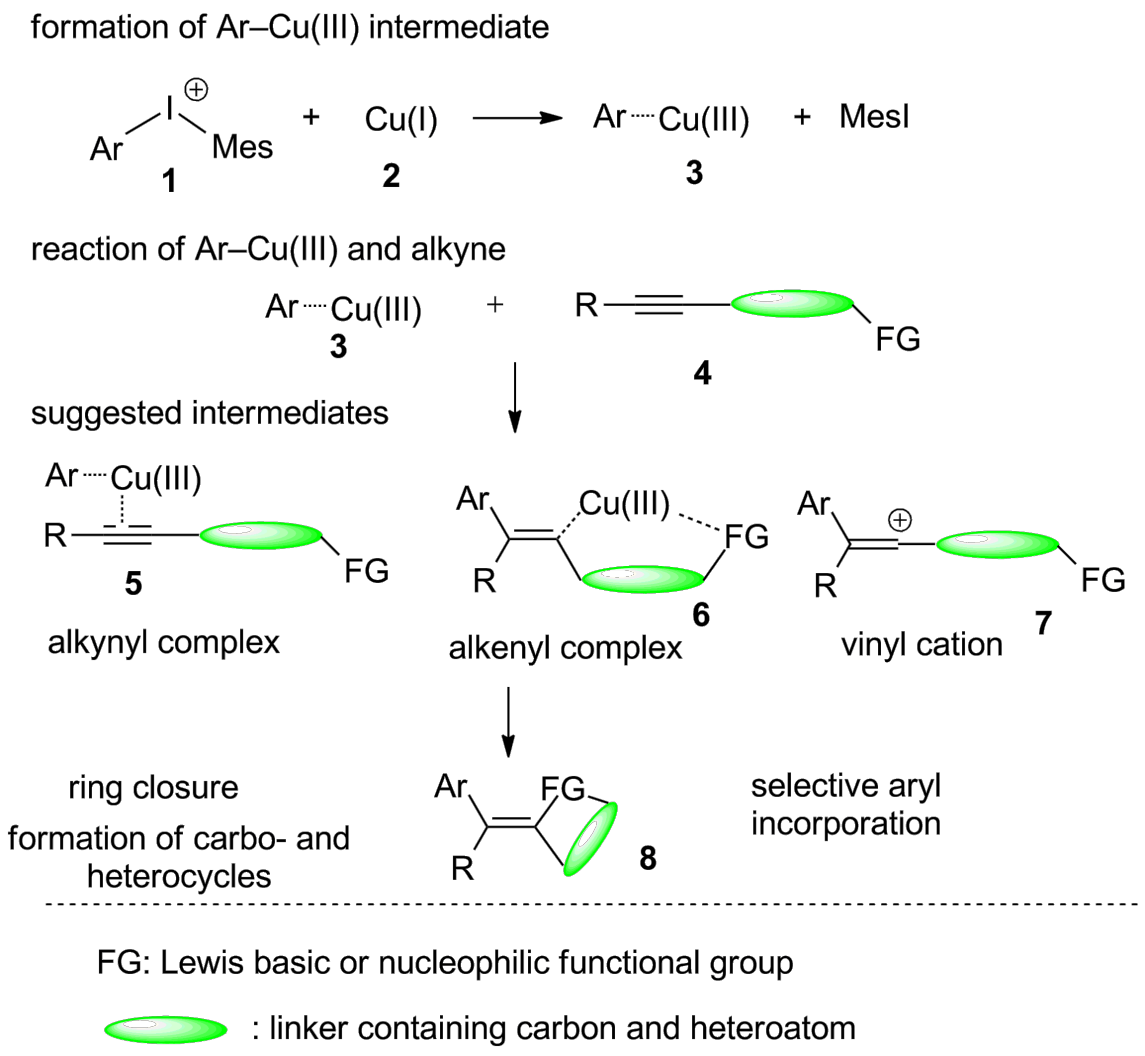
Possible intermediates of the interaction of alkynyl compounds with Ar–Cu(III) species.

Recently a very efficient synthetic strategy has been developed where diaryl iodonium salt **1** [1–8] and copper(I) catalyst **2** are employed together to produce in situ Ar–Cu(III) species **3** for the carbofunctionalisation of appropriate substrates **4** [9–28]. In particular, the arylation–cyclisation reactions promoted by the highly electrophilic Cu(III)–aryl intermediates **3** can allow access to aryl-functionalised carbocyclic and heterocyclic molecules **8** with valuable functionalities [9,29–44]. The mechanistic details of these cascade reactions are not clear as evidenced by the different mechanistic proposals (see, e.g., [18,30,40,44]). These mechanisms suggest the presence and existence of vinyl cation **7**, alkynyl–Cu(III) **5**, or alkenyl–Cu(III) complexes **6** before the C–O bond formation in the ring closing step (see Scheme 1).

As an example of the catalytic arylation–cyclisation strategy, an efficient procedure to form substituted oxazoline derivatives from alkyl and aryl propargylamides has been developed. The process involves a 5-*exo*-dig cyclisation and an aryl group transfer step affording a wide range of oxazoline derivatives [44]. An intriguing issue is the order of the arylation and ring-closure steps and whether this sequence can be affected by the electronic or steric properties of the ligands. Although these mechanistic variations have been postulated in the literature, the exact sequence remained unclear. In this article we report our theoretical studies addressing the mechanism of this reaction, which could provide valuable information for other, analogous copper-catalysed arylation–cyclisation reactions.

## Results and Discussion

**Scheme 2:**
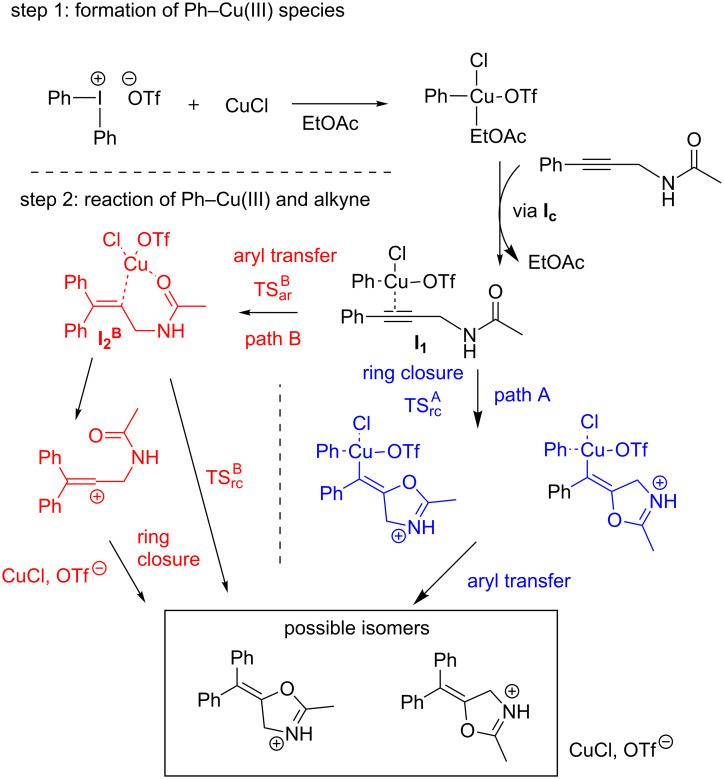
Two possible reaction routes for the oxazoline formation explored by computations. The schemes indicate the possible stereochemical outcomes. For the definition of the labels of intermediates and transition states see Scheme 3.

First, we explain our computational strategy and discuss the possible reaction paths leading to the formation of 5-(diphenylmethylene)-4,5-dihydrooxazole in the reaction of propargylic amides and diaryliodonium salts in the presence of a Cu(I) catalyst. This is a simplified model of the original reaction scheme [44] and allows the exploration of the possible reaction routes of the carboarylation–ring-closure reactions in a computationally efficient manner. As the first step of the reaction we considered the formation of the key Ar–Cu(III) species, followed by the interaction of this intermediate with the alkyne (Scheme 2, step 1). In the next step we compared the energetics of two different paths (paths A and B), to get insight into the order of the arylation and cyclisation steps. Additionally, the relevance of vinyl cation formation and the stereoselectivity were examined.

The energy profiles start with the interaction of the reactant with the catalyst complex formed in the EtOAc medium. In this process the complexing EtOAc ligand leaves and the reactant *N*-(3-phenylprop-2-yn-1-yl)acetamide binds to the Cu(III) ion in an η^2^ mode with its triple bond yielding **I_1_**. The process occurs via an associative substitution route often observed for the 16 electron metal complexes. We could locate a crucial structure (**I_c_**) where the incoming reactant and the leaving solvent molecule occupy the equatorial position of the trigonal bipyramid formed by the five ligands of the Cu(III) ion. We decided to characterise this step by the free energy level of the intermediate: 17.6 kcal/mol. There are two reasons behind this choice: i) the preceding and subsequent barriers were computed to be very close in energy to that of this structure; ii) one of the participants of this step is the solvent EtOAc molecule, i.e., the solvent plays a two-fold role: it is a reactant and a solvating agent; as it is known, such situations are difficult to describe by implicit solvent models [45]. The intermediate formed in this step (**I_1_**) is stabilised at 5.7 kcal/mol.

**Scheme 3:**
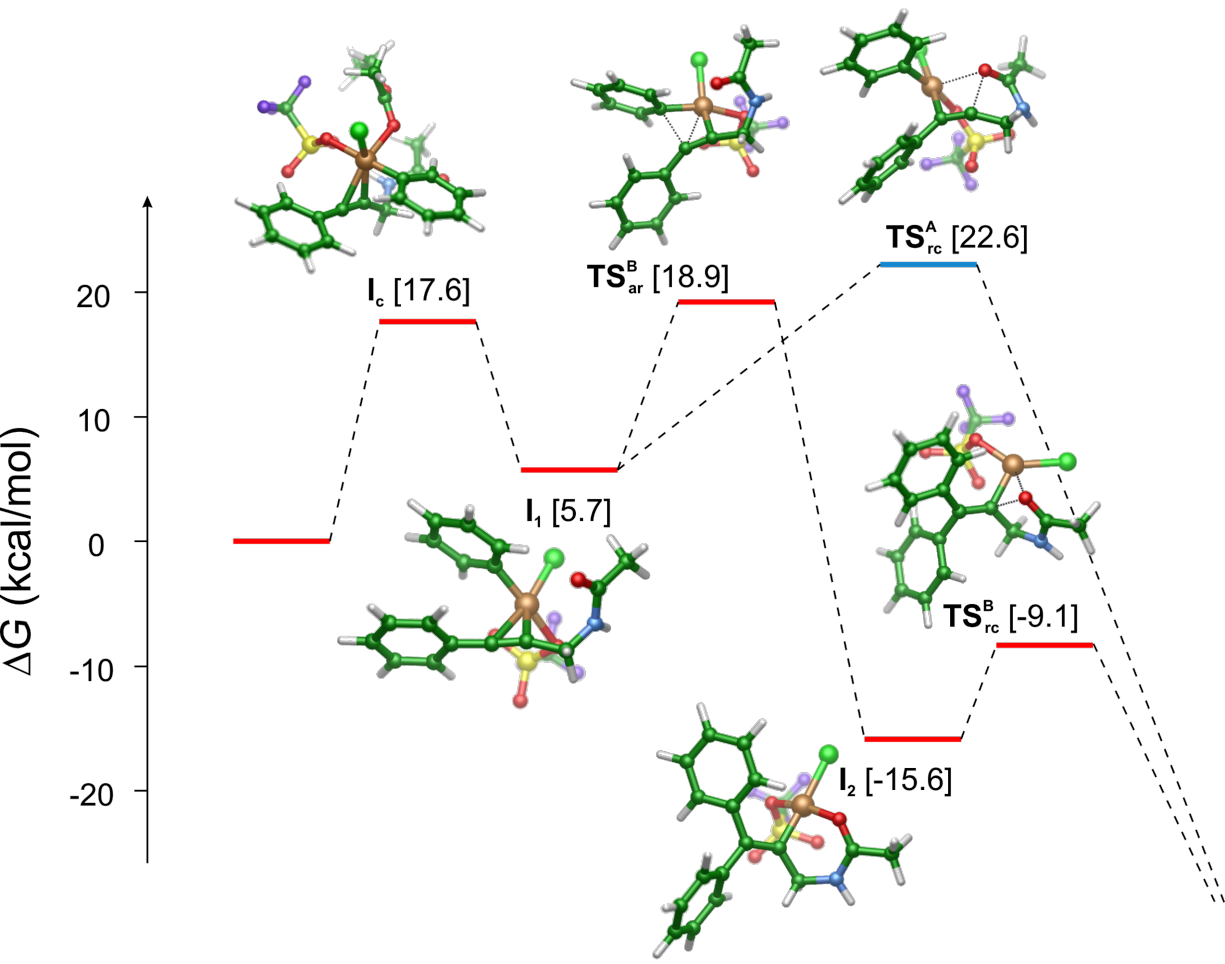
Free energy profiles for the possible reaction routes. The final energy state (−50.5 kcal/mol) is not shown. Red profile: first the aryl transfer occurs followed by the oxazoline ring closure; blue profile: ring closing takes place first followed by spontaneous aryl transfer. The dashed lines are only guides to the eyes. Colour code for the structures: green: C; red: O; light green: Cl; blue: N; yellow: S; violet: F; bronze: Cu.

From this intermediate, the two reaction paths diverge. On path A (blue in Scheme 2 and Scheme 3) the ring formation takes place with an activation free barrier of 22.6 kcal/mol (TS_rc_^A^). Along this path this is the rate determining step. The calculations revealed that once the ring is formed, the aryl transfer spontaneously occurs and a significant amount of free energy is released (more than 70 kcal/mol) by the formation of the adduct of the protonated product and the catalyst (free energy level of −50.5 kcal/mol, not shown in Scheme 3).

In contrast the route starting with the aryl transfer from Cu(III) to the activated reactant features a two-step mechanism (red in Scheme 2 and Scheme 3): the aryl-transfer leads to the formation of a quite stable intermediate **I_2_^B^** with a ca. −20 kcal/mol exergonicity with respect to the first intermediate (**I_1_**). We can also notice that this step requires a smaller, 18.9 kcal/mol activation free energy (TS_ar_^B^) as compared to TS_rc_^A^. The aryl transfer is followed by the O–C bond formation which results in the oxazoline ring. This step requires a moderate 6.5 kcal/mol activation energy (TS_rc_^B^) which indicates that this step is very fast under the reaction conditions. After the ring is formed the system is stabilised by releasing a large amount free energy to arrive at the same state as postulated for path A.

Comparison of the two free energy profiles indicates that the preferred route is the one where the aryl transfer precedes the oxazoline ring formation. On the other hand, the calculated activation free energy barriers are compatible for both routes with the experimental conditions and indicate that both mechanisms can operate at the relatively low, 50 °C temperature.

As the reaction profiles indicate the final state is highly stable. Further stabilisation is expected when the product is formed by deprotonation (presumably at the work-up stage). However, deprotonation may occur earlier if this is thermodynamically favourable in the presence of a suitable base. In the reaction mixture such potential bases are the triflate anion and the reactant. As they are very weak bases we can expect that deprotonation does not take place before the final product formation. Indeed, the calculations show that none of the reactants and intermediates is strong enough acids to deprotonate: +55 kcal/mol, +18 kcal/mol and +25 kcal/mol of free energy are required to deprotonate the reactant and intermediates **I_1_** and **I_2_^B^**, respectively.

As Scheme 2 indicates the products oxazoline can be *cis-* or *trans-*isomers regarding the relative positions of the oxazoline oxygen and the incoming aryl group at the double bond. Formation of a vinyl cation would imply a non-stereospecific oxazoline formation. The calculations, however, revealed that its formation requires ca. 3 kcal/mol more free energy investment than the barrier toward the intramolecular ring closure (TS_rc_^B^). Therefore, we can exclude that the reaction path goes through a vinyl cation intermediate. In contrast, the mechanism obtained from the calculations shows that the catalyst steadily interacts with the substrate via Cu–C bonds along the full path. Further inspection reveals a crucial interaction between the carbonyl oxygen and the catalyst Cu ion (see, e.g., **I_2_^B^** in Scheme 3 where the Cu–O bond length is 1.87 Å). In fact, this cooperation drives selectively the reaction toward the formation of the *cis-*isomer, which is consistent with the experimental results.

Although the above 3 kcal/mol energy difference is large enough to guide the reaction toward the intermolecular ring closure, it is important to note that this also indicates an opportunity to influence the reaction mechanism: stabilisation of the vinyl cation [17,42] may induce a deviation toward a path with less efficient stereocontrol.

To obtain further insight into the mechanism we have calculated these paths for a large number of reactions where the R^1^, R^2^ and R^3^ substituents of the reactants are varied (see reaction scheme in Table 1). A selection of these routes is summarised in Table 1 whereas the data of the full set of reactions are given in Supporting Information File 1. The reactions collected in Table 1 represent the scope of the methodology [44]. Inspection of Table 1 shows that the aryl transfer route is always preferred to the one where the oxazoline ring formation occurs first (the barriers of the ring closure are consistently higher than those of the aryl transfers). It is also interesting to note that in some cases the initial complex formation is the rate determining step along the aryl-transfer path although in most cases the differences in the two barrier heights are very small.

**Table 1:** Effect of the substituents on the barrier heights (kcal/mol). Selection of the substituents is based on [44].

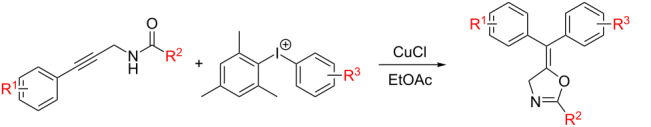					

			barrier of (kcal/mol)		
			
R^1^	R^2^	R^3^	complex formation	aryl transfer first	ring closure first

Ph	*t*-Bu	Ph	16.9	17.6	19.8
*o*-Me-Ph	*t*-Bu	Ph	19.4	18.8	21.3
*p*-Me-Ph	*t*-Bu	Ph	15.4	17.5	20.9
*p*-OMe-Ph	*t*-Bu	Ph	15.8	17.7	20.3
*p*-COOEt-Ph	*t*-Bu	Ph	18.5	18.8	21.7
*p*-Ac-Ph	*t*-Bu	Ph	18.6	18.9	20.3
*p*-Cl-Ph	*t*-Bu	Ph	16.6	17.8	19.1
*m*-Br-Ph	*t*-Bu	Ph	17.0	18.7	20.3
Ph	Ph	Ph	16.5	18.5	21.9
Ph	*p*-MeO-Ph	Ph	12.3	11.8	16.1
Ph	*p*-NO_2_-Ph	Ph	17.0	19.5	23.9
Ph	*t*-Bu	*m*-Br-Ph	18.8	17.1	19.4
Ph	*t*-Bu	*p*-Ac-Ph	18.1	17.6	18.8
2-thiophene	*t*-Bu	Ph	16.9	18.5	18.5
Ph	Et	Ph	18.0	18.6	21.0
Et	*t*-Bu	Ph	17.9	17.2	19.5

The full set of reactions also shows that the aryl transfer as the first step after the complex formation with the catalyst is preferred over the route where the ring closure precedes the aryl transfer. Only three cases from the calculated ca. fifty reactions show a reverse trend. We could not identify a common motif behind this discrepancy; instead we attribute these exceptions to the limitations of the methodology.

## Conclusion

In summary, we have shown with the selected model reaction that the above copper-catalysed carboarylation–ring closure reaction of alkynyl substrates with diaryliodonium salts can be depicted as follows: first the Cu(III)–aryl electrophile forms an intermediate with the triple bond of the reactant, then the aryl moiety migrates to the activated triple bond which is followed by a fast ring-closing step. The calculations provided several new chemical insights: deprotonation can take place only after the tandem arylation–cyclisation sequence; the mechanism shows a very limited sensitivity in a wide range of substituents installed on the reactants; a crucial copper–oxygen interaction is responsible for the very high stereoselectivity of the reaction and it also excludes the formation of vinyl-cation intermediates. The obtained results could serve as a useful and more general description of the mechanism of the carboarylation–ring closure strategy based on the utilisation of alkynes and diaryliodonium salts, beyond the selected and studied oxazoline synthesis.

## Experimental

The calculations have been performed using the Gaussian 09 program package [46]. The M06 exchange–correlation functionals have been employed to solve the Kohn–Sham equations [47]. For the geometry optimisations, transition state searches and vibrational calculations the 6 31G* basis set was used. All the stationary structures obtained by the optimisation procedures were further recalculated using the 6 311++G(3df,3pd) basis set and the SMD implicit solvent model [48] employing ethyl acetate as solvent. The equilibrium structures of the reactant, product and intermediate states had only positive frequencies. The transition states have been verified having a single imaginary frequency and connecting the corresponding intermediate structures. The discussions are based on Gibbs free energies obtained within the ideal-gas model using the rigid-rotor harmonic-oscillator model for 323.15 K (experimental condition). The present methodology and its close variants have been successfully applied to explore the mechanisms of Cu-catalysed organic reactions [49–51].

## Supporting Information

File 1 Full version of Table 1, total energies and Cartesian coordinates of all stationary points.
