# Supplementary material for: DFT calculations on the mechanism of copper-catalysed tandem arylation–cyclisation reactions of alkynes and diaryliodonium salts
Source: Beilstein J Org Chem. 2018 Jul 12;14:1743–9. doi: 10.3762/bjoc.14.148 (PMC6071690; doi:10.3762/bjoc.14.148)
Supplement: File 1 — Full version of Table 1, total energies and Cartesian coordinates of all stationary points. [file Beilstein_J_Org_Chem-14-1743-s001.pdf]

Supporting Information

for

DFT calculations on the mechanism of copper-catalysed tandem arylation–cyclisation reactions of alkynes and diaryliodonium salts

Tamás Károly Stenczel[1], Ádám Sinai[2,3], Zoltán Novák*[2] and András Stirling*[4]

Address: [1]Török Ignác Secondary School, Gödöllő, Hungary, Present address: St Catharine's College, Cambridge CB2 1RL, UK, [2]ELTE "Lendület" Laboratory of Catalysis and Organic Synthesis, Eötvös Loránd University, Institute of Chemistry, Budapest, Hungary, [3]Servier Research Institute of Medicinal Chemistry, Záhony utca 7, H-1031, Budapest, Hungary and [4]Research Centre for Natural Sciences of the, Hungarian Academy of Sciences, Institute of Organic Chemistry, Budapest, Hungary

Email: Zoltán Novák – novakz@elte.hu; András Stirling - stirling.andras@ttk.mta.hu

* Corresponding author

Full version of Table 1, total energies and Cartesian coordinates of all stationary points

Table of Contents

Full version of Table 1 ....S2

Cartesian coordinates and total energies of the stationary points...S4

Full version of Table 1

In Table 1 we have collected the whole set of reactions considered in this study. This set contains the reactions successfully performed in Ref. 1 to explore the scope of the title reaction and their analogones by varying the positions of the substituents (e.g. all the *ortho*, *meta*, *para* isomers are considered here for a given substituent, even if in Ref 1 not all of these possibilities are available).

Table 1. Effect of the substituents on the barrier heights in kcal/mol. Selection of the substituents are based on Ref. 1. Red values in the Table indicate the reactions where the order of the barriers for the aryl transfer and ring closing is reversed.

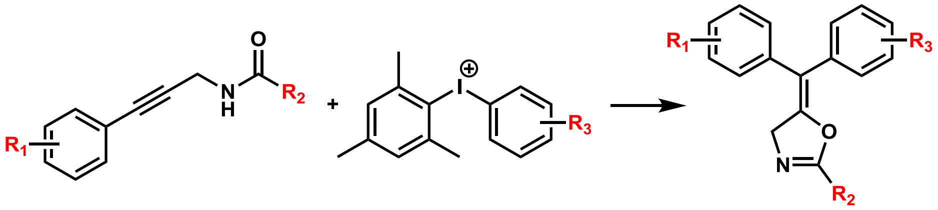

	R_1	R_2	R_3	barrier		
				complex formation	first aryl transfer	first ring closing
1	Ph	*t*-Bu	Ph	16.91	17.63	19.75
2	*o*-Me-Ph	*t*-Bu	Ph	19.37	18.84	21.29
3	*m*-Me-Ph	*t*-Bu	Ph	16.59	17.53	19.88
4	*p*-Me-Ph	*t*-Bu	Ph	15.41	17.50	20.86
5	*o*-OMe-Ph	*t*-Bu	Ph	19.57	17.36	20.38
6	*m*-OMe-Ph	*t*-Bu	Ph	17.48	20.65	20.52
7	*p*-OMe-Ph	*t*-Bu	Ph	15.81	17.66	20.33
8	*o*-COOEt-Ph	*t*-Bu	Ph	18.97	21.30	19.34
9	*m*-COOEt-	*t*-Bu	Ph	19.04	18.56	21.86
10	*p*-COOEt-Ph	*t*-Bu	Ph	18.55	18.83	21.67
11	*o*-Ac-Ph	*t*-Bu	Ph	20.24	17.78	19.78
12	*m*-Ac-Ph	*t*-Bu	Ph	19.14	17.96	20.36
13	*p*-Ac-Ph	*t*-Bu	Ph	18.56	18.91	20.28
14	*o*-Cl-Ph	*t*-Bu	Ph	23.30	19.96	24.85
15	*m*-Cl-Ph	*t*-Bu	Ph	17.80	18.39	20.97
16	*p*-Cl-Ph	*t*-Bu	Ph	16.64	17.81	19.11
17	*o*-Br-Ph	*t*-Bu	Ph	21.11	19.67	23.78
18	*m*-Br-Ph	*t*-Bu	Ph	17.04	18.65	20.34
19	*p*-Br-Ph	*t*-Bu	Ph	16.54	18.02	19.57
20	*o*-NO_2-Ph	*t*-Bu	Ph	24.04	24.75	22.69
21	*m*-NO_2-Ph	*t*-Bu	Ph	21.32	19.78	21.76

22	*p*-NO_2-Ph	*t*-Bu	Ph	18.37	19.09	19.96
23	*o*-OH-Ph	*t*-Bu	Ph	17.27	14.73	19.50
24	*m*-OH-Ph	*t*-Bu	Ph	17.59	18.33	19.88
25	*p*-OH-Ph	*t*-Bu	Ph	16.12	17.35	19.11
26	*o*-F-Ph	*t*-Bu	Ph	22.73	23.00	24.80
27	*m*-F-Ph	*t*-Bu	Ph	16.87	18.69	20.48
28	*p*-F-Ph	*t*-Bu	Ph	15.79	18.09	18.74
29	*o*-NH_2-Ph	*t*-Bu	Ph	12.90	15.09	20.74
30	*m*-NH_2-Ph	*t*-Bu	Ph	16.40	16.32	19.00
31	*p*-NH_2-Ph	*t*-Bu	Ph	12.26	15.73	20.60
32	Ph	Ph	Ph	16.48	18.45	21.86
33	Ph	*o*-OMe-Ph	Ph	12.77	11.79	16.06
34	Ph	*m*-OMe-Ph	Ph	12.32	13.74	15.10
35	Ph	*p*-OMe-Ph	Ph	19.39	17.66	21.90
36	Ph	*o*-NO_2-Ph	Ph	17.03	19.51	23.91
37	Ph	*m*-NO_2-Ph	Ph	15.01	19.72	25.23
38	Ph	*p*-NO_2-Ph	Ph	17.79	17.47	20.37
39	Ph	*o*-Me-Ph	Ph	14.46	15.76	19.87
40	Ph	*m*-Me-Ph	Ph	17.69	19.71	22.50
41	Ph	*p*-Me-Ph	Ph	17.21	16.62	21.30
42	Ph	*t*-Bu	*o*-Br-Ph	21.32	12.33	20.27
43	Ph	*t*-Bu	*m*-Br-Ph	18.80	17.08	19.36
44	Ph	*t*-Bu	*p*-Br-Ph	18.20	17.19	19.02
45	Ph	*t*-Bu	*p*-Ac-Ph	18.07	17.61	18.79
46	2-tiophene	*t*-Bu	Ph	16.92	18.49	18.52
47	3-tiophene	**t**-Bu	Ph	16.28	18.06	18.83
48	Ph	Et	Ph	17.96	18.61	21.04
49	Ph	CF_3	Ph	18.36	24.08	30.01
50	Ph	*t*-Bu+2Me	Ph	19.04	13.94	12.90
51	Et	*t*-Bu	Ph	17.94	17.15	19.53

[1] Sinai, Á.; Vangel, D.; Gáti, T.; Bombicz, P.; Novák, Z. *Org. Lett.* **2015**, *17*, 4136.

Cartesian coordinates and total energies of the stationary points.

CuCl

```
  2
Energy: -2100.33636207
 Cu 0.000000 0.000000 0.422401
 Cl 0.000000 0.000000 2.429399
```

OTf^-

```
  8
Energy: -961.277500421
 O 2.248393 0.155871 -0.965618
 S 2.480413 -0.111340 -2.391788
 O 3.862110 0.071139 -2.857335
 O 1.770186 -1.272333 -2.945679
 C 1.610881 1.295077 -3.195534
 F 1.709367 1.237405 -4.528835
 F 0.307459 1.305412 -2.893595
 F 2.116825 2.472418 -2.810340
```

EtOAc

```
  14
Energy: -307.495855628
 O -2.788293 -0.428396 -0.356844
 C -3.107612 -0.260801 1.020774
 C -4.211652 -1.204729 1.438742
 H -2.178671 -0.488364 1.556694
 H -3.360813 0.784956 1.243139
 H -4.395377 -1.132637 2.517607
 H -5.151960 -0.981513 0.917779
 H -3.934690 -2.238280 1.201725
 C -3.407565 0.268885 -1.335436
 O -3.161241 0.003900 -2.482286
 C -4.351352 1.375706 -0.934453
 H -5.139250 1.036404 -0.251527
 H -3.811206 2.191516 -0.436908
 H -4.809545 1.765841 -1.845685
```

Model reaction (Figure 3 in article)

reactant

```
  24
Energy: -555.309710497
 C -4.089402 -0.019916 0.153915
 O -4.681396 -0.037611 -0.912411
 C -4.801504 -0.110937 1.481473
 N -2.732078 0.081400 0.218168
 H -2.243852 0.107597 1.102131
 C -1.953259 0.173469 -1.006717
 C -0.528479 0.131280 -0.721183
 H -2.246406 -0.650566 -1.674900
 H -2.214593 1.098158 -1.545385
 C 0.655237 0.097340 -0.466187
 C 2.050893 0.050578 -0.170903
 C 2.678863 -1.175913 0.088459
 C 4.035695 -1.220156 0.377637
 C 4.782887 -0.045564 0.410841
 C 4.167654 1.176816 0.153510
 C 2.810765 1.228321 -0.134994
 H 2.086665 -2.088871 0.057991
 H 4.514391 -2.177291 0.577456
 H 5.847136 -0.082882 0.637108
 H 4.749628 2.096535 0.177872
 H 2.320957 2.179169 -0.337710
 H -4.134176 -0.081839 2.350516
 H -5.518049 0.714537 1.555191
 H -5.379506 -1.041285 1.508119
```

I_c

```
  59
Energy: -4155.93682091
```

```
 Cu 0.783789 0.275334 -0.507549
 Cl 1.287736 -0.294938 -2.547624
 C 2.142040 1.591438 -0.733841
 O -0.633375 -1.163690 -0.358198
 S -1.508930 -1.603697 0.782156
 O -2.297938 -0.517966 1.376416
 C -2.749160 -2.599696 -0.130874
 O -0.866324 -2.540520 1.696330
 F -3.676546 -3.031211 0.707675
 F -3.342104 -1.848896 -1.065044
 F -2.172697 -3.631161 -0.721278
 C 1.695253 2.872616 -0.476049
 C 2.658723 3.882853 -0.386495
 C 4.004227 3.601478 -0.581080
 C 4.407656 2.300362 -0.870093
 C 3.468593 1.273823 -0.957709
 C -1.146073 2.997621 1.921343
 O -0.373976 3.927007 1.729402
 C -2.639638 3.148383 1.820507
 N -0.730802 1.754206 2.310917
 C 0.669505 1.460838 2.420064
 C 1.162402 0.388765 1.531159
 C 1.820000 -0.627256 1.271544
 C 2.649744 -1.722016 0.920978
 C 2.109356 -2.987857 0.635069
 C 2.962259 -4.024755 0.290092
 C 4.339318 -3.817693 0.237304
 C 4.880072 -2.567721 0.533647
 C 4.041863 -1.517163 0.870579
 H 0.651018 3.114493 -0.297016
 H 2.325203 4.890996 -0.144944
 H 4.742988 4.398286 -0.515823
 H 5.458145 2.074132 -1.048191
 H 3.772124 0.261380 -1.217044
 H -1.382858 0.973642 2.241860
 H 1.205351 2.394928 2.190668
 H 0.936456 1.167505 3.447395
 H 1.032811 -3.133154 0.702716
 H 2.550040 -5.005448 0.062734
 H 4.999225 -4.640059 -0.033874
 H 5.956696 -2.414473 0.499355
 H 4.442174 -0.532166 1.106282
 H -3.162612 2.185956 1.793826
 H -2.991896 3.731848 2.680268
 H -2.875531 3.711136 0.912003
 O -3.120683 1.523119 -0.781660
 C -4.384990 1.075559 -1.293079
 C -5.274424 0.827547 -0.102515
 C -1.973314 1.296893 -1.404624
 O -0.945425 1.603400 -0.815314
 C -1.993846 0.696245 -2.774004
 H -4.785330 1.855780 -1.956432
 H -4.254062 0.157247 -1.875654
 H -6.260189 0.478138 -0.430937
 H -4.824006 0.065337 0.545683
 H -5.408948 1.744986 0.482814
 H -2.795572 1.111422 -3.394531
 H -1.022911 0.854228 -3.247598
 H -2.144709 -0.388148 -2.685479
```

I_1

```
  45
Energy: -3848.41312741
 Cu -0.014459 0.015715 0.000557
 Cl -0.007390 0.014221 2.134073
 C 1.851517 0.014226 0.294837
 O -2.025260 0.305370 -0.278029
 S -1.981675 1.722676 -0.801078
 O -2.638716 1.937762 -2.076237
 C -2.957037 2.645336 0.448114
 O -0.590750 2.208656 -0.653502
 F -2.957801 3.930403 0.132735
 F -4.199711 2.191089 0.454739
 F -2.422715 2.485334 1.644426
 C 2.592169 -1.149704 0.334135
 C 3.978650 -1.014669 0.241274
 C 4.564683 0.245179 0.144376
 C 3.774640 1.389746 0.143046
 C 2.385610 1.287910 0.242669
 H 2.123452 -2.131350 0.412163
```

H 4.597012 -1.911084 0.251853
H 5.647952 0.334847 0.084556
H 4.230253 2.376814 0.082202
H 1.754513 2.174825 0.272297
C -0.510300 -3.267051 0.615329
O 0.516914 -3.685286 0.099392
C -0.839597 -3.474245 2.066062
N -1.430492 -2.562546 -0.114210
C -1.152735 -2.245636 -1.495600
C -0.171305 -1.157826 -1.697686
C 0.562656 -0.353484 -2.271647
C 1.502137 0.515748 -2.885343
C 1.139250 1.823122 -3.245647
C 2.087878 2.659235 -3.815012
C 3.385878 2.200689 -4.032133
C 3.745417 0.899549 -3.686128
C 2.808533 0.054467 -3.112680
H -2.173696 -2.052363 0.349231
H -0.759373 -3.145177 -1.985480
H -2.091172 -1.958415 -1.983164
H 0.125191 2.166606 -3.052906
H 1.814344 3.675635 -4.090018
H 4.125056 2.864532 -4.477688
H 4.760348 0.547253 -3.859071
H 3.071653 -0.960244 -2.818952
H -1.876780 -3.228303 2.318266
H -0.631019 -4.515358 2.332058
H -0.178937 -2.832751 2.662961

TS_{rc}^{A}

45
Energy: -3848.38892019
Cu 0.653730 -1.010189 -1.051686
Cl 1.512475 -2.934619 -0.692560
C 2.394609 -0.350841 -1.297467
O -1.181389 -1.599145 -0.549483
S -2.594418 -1.089515 -0.438221
O -3.504193 -1.764658 -1.368196
C -3.045270 -1.700240 1.229860
O -2.705825 0.366365 -0.358262
F -4.287696 -1.339376 1.507604
F -2.946395 -3.015413 1.282654
F -2.226045 -1.162417 2.127278
C 2.939950 -0.266468 -2.565308
C 4.199229 0.318751 -2.698876
C 4.879710 0.791283 -1.580808
C 4.309482 0.676562 -0.317294
C 3.051633 0.094610 -0.163800
H 2.398563 -0.634883 -3.434657
H 4.645380 0.403025 -3.688969
H 5.862802 1.244876 -1.694330
H 4.842837 1.033626 0.562376
H 2.604696 0.002976 0.826257
C -1.266167 -1.404185 -3.536164
O -0.088531 -1.011842 -3.374615
C -1.618673 -2.843069 -3.601790
N -2.209992 -0.447281 -3.611443
C -1.671108 0.896124 -3.421918
C -0.508785 0.785334 -2.526245
C 0.089444 0.819886 -1.407886
C 0.342780 1.845523 -0.401625
C -0.115882 1.709410 0.911872
C 0.214378 2.673668 1.855901
C 1.009699 3.761975 1.507270
C 1.475452 3.889382 0.202275
C 1.144760 2.936153 -0.752459
H -3.153378 -0.676636 -3.300003
H -1.406932 1.331202 -4.394903
H -2.421843 1.524660 -2.926412
H -0.749924 0.866105 1.178737
H -0.153431 2.570095 2.874992
H 1.269659 4.509045 2.255027
H 2.103386 4.733863 -0.076134
H 1.532005 3.006257 -1.768754
H -1.410261 -3.273968 -2.612905
H -2.669868 -3.007476 -3.850836
H -0.967009 -3.340278 -4.327933

TS_{ar}^{B}

45
Energy: -3848.39813144
Cu 0.403835 -0.955543 -1.159232
Cl 1.314041 -2.611394 -0.100981
C 2.074920 0.033312 -1.210084
O -1.418500 -1.699452 -0.883227
S -2.625688 -0.995032 -0.314006
O -3.563453 -0.567288 -1.359618
C -3.446922 -2.411650 0.510839
O -2.301922 -0.033159 0.732847
F -4.588463 -2.003534 1.042895
F -3.696757 -3.355570 -0.387536
F -2.669030 -2.899694 1.457461
C 2.687487 0.120333 -2.453602
C 4.063619 0.336946 -2.503297
C 4.791314 0.485825 -1.327586
C 4.151668 0.411745 -0.090696
C 2.784775 0.181576 -0.022655
H 2.106047 0.005641 -3.366316
H 4.560075 0.396531 -3.470133
H 5.863897 0.665192 -1.373042
H 4.721293 0.518408 0.830455
H 2.277375 0.118676 0.937955
C -0.960817 -1.873793 -3.504633
O 0.254668 -1.703769 -3.349362
C -1.573300 -3.233066 -3.591210
N -1.800682 -0.807442 -3.565049
C -1.250452 0.488552 -3.261836
C -0.395257 0.586514 -2.052090
C 0.359231 1.190370 -1.242278
C 0.605927 2.300536 -0.347254
C -0.315809 2.548453 0.676332
C -0.123239 3.644352 1.510157
C 0.968526 4.485351 1.321536
C 1.885539 4.232749 0.301119
C 1.715673 3.135150 -0.527776
H -2.752628 -0.953005 -3.228420
H -0.630973 0.830244 -4.104205
H -2.084824 1.192759 -3.146939
H -1.159897 1.871724 0.809534
H -0.832701 3.838947 2.311816
H 1.111659 5.343448 1.976081
H 2.738703 4.892703 0.157128
H 2.431784 2.914793 -1.318821
H -2.641833 -3.208165 -3.829502
H -1.039048 -3.827985 -4.339098
H -1.431924 -3.716063 -2.615516

I_2

45
Energy: -3848.45265841
Cu 0.111367 -1.630304 -1.794350
Cl 0.749925 -3.713263 -2.080905
C 2.014027 0.292135 -0.733818
O -0.134133 -1.768608 0.056873
S -1.623209 -1.649573 0.401898
O -2.446225 -1.937056 -0.775652
C -1.814734 -3.081896 1.534117
O -1.932769 -0.458841 1.179101
F -3.072975 -3.115368 1.947673
F -1.528045 -4.198880 0.900448
F -1.014321 -2.934646 2.574708
C 2.591461 -0.635272 -1.611197
C 3.720723 -1.356111 -1.234666
C 4.296319 -1.141086 0.010621
C 3.747590 -0.194714 0.875788
C 2.616040 0.518827 0.508160
H 2.201132 -0.744608 -2.624471
H 4.140563 -2.089367 -1.920154
H 5.176700 -1.706750 0.309691
H 4.199511 -0.023201 1.851206
H 2.168326 1.235783 1.195255
C -1.188879 -0.842029 -4.086010
O -0.156195 -1.396041 -3.632871
C -1.637976 -1.249984 -5.450878
N -1.891606 0.082156 -3.439285
C -1.568041 0.653694 -2.128877
C -0.232897 0.234946 -1.648593
C 0.740756 0.955531 -1.083192

C 0.562911 2.385743 -0.736629
C -0.550735 2.799688 0.002069
C -0.708706 4.141549 0.334617
C 0.240538 5.076775 -0.065030
C 1.360526 4.667213 -0.785923
C 1.526692 3.327844 -1.112581
H -2.755995 0.389698 -3.866848
H -1.569657 1.749841 -2.237634
H -2.369300 0.374464 -1.429818
H -1.264320 2.052784 0.353886
H -1.571140 4.453729 0.921034
H 0.115439 6.126334 0.195970
H 2.110010 5.395763 -1.090574
H 2.405421 2.998860 -1.667290
H -2.470511 -0.648978 -5.829557
H -0.788991 -1.173005 -6.138105
H -1.930975 -2.305521 -5.418050

TS_{rc}^{B}

45
Energy: -3848.42590369
Cu -0.463796 -0.374066 -0.209004
Cl -1.029016 -1.897435 1.256436
C 1.786686 1.431106 -2.062991
O -1.377385 1.145933 -0.897228
S -2.265534 0.843394 -2.096691
O -2.160133 -0.557790 -2.510102
C -3.927496 0.988184 -1.336954
O -2.188983 1.874559 -3.119348
F -4.851067 0.743372 -2.253813
F -4.042446 0.107635 -0.354892
F -4.101479 2.207427 -0.854688
C 1.602362 1.911719 -0.757631
C 2.159618 3.113410 -0.356966
C 2.914297 3.868069 -1.252224
C 3.102991 3.409031 -2.551561
C 2.544495 2.204096 -2.955122
H 0.994405 1.345235 -0.058280
H 1.992361 3.469669 0.657402
H 3.346518 4.816414 -0.938066
H 3.686620 3.994339 -3.259675
H 2.699590 1.851841 -3.973098
C 1.785359 -2.280258 -0.027514
O 1.657596 -1.032999 0.010239
C 2.503402 -3.000339 1.054857
N 1.257811 -2.955853 -1.050901
C 0.361438 -2.223320 -1.938177
C 0.641147 -0.772028 -1.733889
C 1.245380 0.143793 -2.503493
C 1.275475 -0.190699 -3.960344
C 0.344873 0.375159 -4.834051
C 0.405978 0.069941 -6.190137
C 1.384661 -0.792557 -6.675559
C 2.310917 -1.358546 -5.803226
C 2.254975 -1.061427 -4.445909
H 1.194816 -3.965453 -1.002851
H 0.572510 -2.474208 -2.986119
H -0.685094 -2.482184 -1.723060
H -0.428966 1.040037 -4.445610
H -0.323892 0.505662 -6.869701
H 1.425044 -1.027588 -7.737786
H 3.081363 -2.028887 -6.180640
H 2.978516 -1.493573 -3.752218
H 1.808619 -3.065231 1.903720
H 2.813050 -4.007818 0.759316
H 3.372428 -2.417769 1.373596

product (protonated)

34
Energy: -786.204501272
N -0.066546 -1.137033 -0.202380
C -0.002177 -1.044339 1.060052
O 1.178180 -0.591541 1.604772
C 2.025503 -0.361723 0.536954
C 1.225953 -0.686300 -0.702656
H 1.705537 -1.467379 -1.312229
C -1.051467 -1.365501 2.052407
H -0.704449 -2.148979 2.737102

H -1.285249 -0.482502 2.659458
H -1.952227 -1.704587 1.535498
C 3.302321 0.030692 0.680884
C 4.088034 0.260099 -0.561936
C 5.240128 -0.495551 -0.808747
C 5.984485 -0.299103 -1.965069
C 5.594270 0.663212 -2.893296
C 4.459484 1.430792 -2.653296
C 3.715223 1.232062 -1.494245
H 5.547685 -1.242308 -0.076501
H 6.874675 -0.900358 -2.143978
H 6.178498 0.818493 -3.798854
H 4.155545 2.195408 -3.366713
H 2.839982 1.850827 -1.293458
C 3.984609 0.233192 1.976409
H 1.082026 0.174888 -1.370458
C 3.657377 -0.496942 3.127952
C 4.334694 -0.284680 4.321884
C 5.353133 0.660738 4.398713
C 5.692390 1.389879 3.263535
C 5.020606 1.174273 2.067216
H 2.868462 -1.242961 3.082398
H 4.066788 -0.869901 5.200660
H 5.881410 0.824917 5.336549
H 6.487434 2.132937 3.307462
H 5.295771 1.748556 1.183170

Reaction 1

reactant

33
Energy: -673.153098765
C -4.058140 0.007907 0.185254
O -4.640120 -0.004525 -0.890334
C -4.826386 -0.098931 1.507049
N -2.702046 0.101671 0.245077
H -2.203941 0.131332 1.122610
C -1.933000 0.190144 -0.987062
C -0.506143 0.140715 -0.713158
H -2.236614 -0.631924 -1.652685
H -2.195413 1.115676 -1.523528
C 0.679887 0.099636 -0.470043
C 2.078127 0.043723 -0.188988
C 2.705474 -1.189283 0.039155
C 4.065012 -1.242686 0.313779
C 4.815560 -0.070836 0.363510
C 4.200951 1.158004 0.137375
C 2.841405 1.218610 -0.136610
H 2.110588 -2.099981 -0.003969
H 4.543148 -2.204873 0.489197
H 5.881907 -0.115348 0.578319
H 4.785526 2.075640 0.174760
H 2.352119 2.174570 -0.315095
C -3.950746 -0.008699 2.752729
C -5.855280 1.032345 1.521011
C -5.548880 -1.447690 1.485909
H -4.579426 -0.082806 3.650413
H -3.219739 -0.828901 2.806363
H -3.415406 0.950220 2.813431
H -6.188135 -1.548534 2.374304
H -6.174006 -1.533587 0.589728
H -4.833334 -2.282349 1.486159
H -6.503728 0.942654 2.403844
H -5.365812 2.015900 1.558607
H -6.477509 0.995508 0.619835

I_c

68
Energy: -4273.78007071
Cu 0.716981 0.067950 -0.519656
Cl 1.305691 -0.507187 -2.541731
C 1.951517 1.500420 -0.743829
O -0.568468 -1.473260 -0.353936
S -1.400898 -1.911471 0.821487
O -2.072748 -0.810110 1.515757
C -2.759491 -2.777086 -0.055161
O -0.766117 -2.936294 1.641273
F -3.647663 -3.218154 0.819240

F -3.373721 -1.932397 -0.891713
F -2.288708 -3.793549 -0.755725
C 1.392725 2.740424 -0.502288
C 2.258971 3.836100 -0.428096
C 3.623808 3.675197 -0.621473
C 4.144092 2.411687 -0.891213
C 3.302905 1.302710 -0.960554
C -1.075129 2.981046 2.104370
O -0.218481 3.854979 2.018411
C -2.561733 3.341924 2.073731
N -0.751521 1.666465 2.271775
C 0.623751 1.278670 2.414620
C 1.110178 0.221574 1.504617
C 1.827792 -0.758107 1.255723
C 2.725145 -1.796599 0.905305
C 2.266178 -3.096102 0.628203
C 3.181913 -4.077428 0.282755
C 4.542202 -3.781550 0.221145
C 5.003059 -2.497240 0.507504
C 4.100893 -1.501350 0.843866
H 0.329991 2.885745 -0.329466
H 1.836142 4.811096 -0.191761
H 4.287142 4.536482 -0.566589
H 5.210965 2.279536 -1.066406
H 3.697878 0.318179 -1.203882
H -1.444998 0.926712 2.184621
H 1.222984 2.187604 2.251091
H 0.828514 0.927028 3.438486
H 1.201002 -3.307253 0.698024
H 2.832724 -5.083609 0.061478
H 5.252603 -4.560746 -0.050099
H 6.067303 -2.275125 0.465060
H 4.437781 -0.491357 1.072826
C -3.498599 2.150212 2.233905
C -2.790398 4.328916 3.221826
C -2.829219 4.040648 0.739540
O -3.286539 1.073912 -0.987031
C -4.503931 0.731934 -1.664138
C -5.577746 0.689480 -0.609677
C -2.087015 0.895784 -1.527935
O -1.121925 1.220970 -0.851191
C -1.994606 0.323231 -2.904963
H -4.708858 1.494636 -2.429412
H -4.396645 -0.240235 -2.159657
H -6.543700 0.427914 -1.056345
H -5.324093 -0.060161 0.150203
H -5.671859 1.663931 -0.115176
H -2.740921 0.756988 -3.579625
H -0.987934 0.480820 -3.296658
H -2.165596 -0.760550 -2.852312
H -3.340271 1.626886 3.187668
H -4.538816 2.506965 2.229908
H -3.391114 1.426741 1.415265
H -3.874905 4.379343 0.696568
H -2.173536 4.911844 0.621924
H -2.656748 3.355083 -0.100579
H -3.829528 4.686956 3.204953
H -2.614022 3.851689 4.196437
H -2.117982 5.189931 3.134706

I_1

54
Energy: -3966.25843998
Cu -0.008123 0.112228 0.009344
Cl -0.005284 0.326283 2.131331
C 1.857415 0.182388 0.311729
O -2.025402 0.305687 -0.285523
S -2.043725 1.682993 -0.909306
O -2.706135 1.771695 -2.196994
C -3.066876 2.645913 0.269262
O -0.679871 2.245917 -0.796887
F -3.117816 3.905513 -0.133355
F -4.290259 2.142564 0.300487
F -2.536417 2.590703 1.476946
C 2.620152 -0.960824 0.440947
C 4.005212 -0.803534 0.358902
C 4.568086 0.458351 0.183665
C 3.755921 1.583855 0.092095
C 2.368096 1.459866 0.178747
H 2.169332 -1.945092 0.574418
H 4.640951 -1.684095 0.439308

H 5.650242 0.565915 0.133574
H 4.193130 2.573672 -0.029412
H 1.719129 2.333626 0.140092
C -0.441175 -3.210474 0.705972
O 0.624995 -3.556851 0.209592
C -0.821624 -3.573962 2.139118
N -1.365694 -2.542217 -0.048157
C -1.067863 -2.237620 -1.427529
C -0.106509 -1.132804 -1.632299
C 0.614346 -0.339617 -2.239077
C 1.537217 0.521075 -2.886503
C 1.142852 1.796334 -3.321936
C 2.074936 2.624960 -3.928338
C 3.387131 2.190444 -4.107160
C 3.778421 0.921206 -3.684993
C 2.858314 0.083840 -3.074608
H -2.153035 -2.071021 0.379351
H -0.647073 -3.133697 -1.901095
H -2.003338 -1.973516 -1.933835
H 0.117761 2.121585 -3.156286
H 1.777845 3.616762 -4.262371
H 4.113004 2.848546 -4.582182
H 4.804691 0.588634 -3.827272
H 3.145591 -0.904289 -2.719198
C -2.044573 -2.824688 2.661039
C -1.111722 -5.079693 2.121043
C 0.385277 -3.295437 3.034500
H 0.174325 -3.634152 4.058146
H 1.270360 -3.827132 2.666200
H 0.614074 -2.221255 3.069927
H -1.351354 -5.424219 3.136561
H -1.968101 -5.313432 1.472254
H -0.240583 -5.639376 1.758931
H -2.219666 -3.100878 3.709348
H -1.904689 -1.734451 2.629114
H -2.958513 -3.087798 2.108280

TS_{rc}^{A}

54
Energy: -3966.23520387
Cu -0.573219 -0.442245 -0.769777
Cl -0.344734 -1.159287 -2.770815
C -2.253191 -1.284160 -0.772598
O 0.983932 0.772006 -0.843019
S 1.955963 1.437388 0.093059
O 3.177464 0.656248 0.295551
C 2.448620 2.875248 -0.931478
O 1.348713 2.015127 1.291934
F 3.319268 3.612119 -0.258341
F 2.999190 2.470624 -2.061557
F 1.377670 3.611964 -1.203847
C -2.409839 -2.546310 -0.229314
C -3.698790 -3.075112 -0.157462
C -4.786559 -2.349668 -0.634044
C -4.593376 -1.090674 -1.193135
C -3.312308 -0.546149 -1.273005
H -1.554123 -3.104801 0.144923
H -3.846170 -4.063255 0.276566
H -5.788499 -2.771611 -0.577201
H -5.438995 -0.523613 -1.579454
H -3.158487 0.438560 -1.714981
C 1.749181 -1.934981 1.139836
O 0.578327 -2.014379 0.711219
C 2.899355 -2.644154 0.461768
N 1.913310 -1.267822 2.297513
C 0.692284 -0.720030 2.868267
C -0.287614 -0.475229 1.800956
C -1.048314 0.166768 1.027657
C -2.031132 1.241433 1.117262
C -1.785123 2.497496 0.555927
C -2.771948 3.474600 0.608336
C -4.002487 3.206422 1.201233
C -4.249123 1.950300 1.747627
C -3.269631 0.966496 1.705575
H 2.812111 -0.858181 2.524078
H 0.294250 -1.407379 3.628598
H 0.910439 0.252005 3.330831
H -0.818790 2.706160 0.102079
H -2.574442 4.454813 0.178483
H -4.771733 3.975932 1.233236
H -5.211816 1.730918 2.206071

H -3.462849 -0.031526 2.099124
C 2.860200 -2.338464 -1.036430
C 4.252928 -2.261308 1.051618
C 2.640912 -4.141360 0.694198
H 3.440869 -4.719474 0.213316
H 2.639154 -4.390227 1.765104
H 1.683006 -4.450686 0.259266
H 5.038225 -2.819792 0.526459
H 4.459008 -1.192174 0.918006
H 4.329366 -2.526186 2.116692
H 3.653369 -2.911294 -1.535358
H 1.897942 -2.618790 -1.482377
H 3.026514 -1.271230 -1.226172

TS_{ar}^{B}

54
Energy: -3966.24687971
Cu 0.287213 -0.570805 0.460092
Cl 0.024224 -0.962233 2.575700
C 2.109237 -1.251930 0.487735
O -1.442230 0.407677 0.363162
S -1.671261 1.807576 -0.152476
O -2.343803 1.809696 -1.456802
C -2.936585 2.384021 1.043603
O -0.522934 2.690266 0.017937
F -3.319977 3.607868 0.715552
F -3.987896 1.574024 1.004600
F -2.434335 2.391038 2.263165
C 2.310864 -2.452510 -0.180580
C 3.394886 -3.246890 0.189813
C 4.262740 -2.823775 1.190401
C 4.051253 -1.605008 1.834134
C 2.968869 -0.808108 1.487252
H 1.627121 -2.771083 -0.964659
H 3.559072 -4.195892 -0.317413
H 5.112908 -3.443585 1.469220
H 4.723806 -1.276032 2.624016
H 2.793980 0.142086 1.987718
C -1.784238 -1.792255 -1.153564
O -0.774952 -2.300971 -0.645095
C -3.183594 -2.207953 -0.736444
N -1.648276 -0.890015 -2.160247
C -0.316211 -0.475352 -2.517780
C 0.605001 -0.118237 -1.410851
C 1.720087 0.220652 -0.931388
C 2.855675 1.116411 -0.899888
C 2.617431 2.486207 -0.737763
C 3.691429 3.368793 -0.763442
C 4.984590 2.893647 -0.954968
C 5.217424 1.527594 -1.114673
C 4.158558 0.634160 -1.076211
H -2.358841 -0.164262 -2.237748
H 0.179296 -1.283488 -3.076604
H -0.399620 0.393157 -3.184499
H 1.596352 2.834028 -0.582879
H 3.512995 4.434060 -0.632070
H 5.820926 3.590332 -0.976733
H 6.230532 1.159582 -1.264281
H 4.326129 -0.436855 -1.185039
C -4.270142 -1.278117 -1.269298
C -3.380427 -3.614821 -1.318815
C -3.245993 -2.267465 0.789964
H -4.371416 -3.993046 -1.033506
H -3.323095 -3.605966 -2.416804
H -2.621413 -4.307116 -0.934234
H -4.216540 -2.681377 1.095968
H -2.449447 -2.900781 1.196850
H -3.136745 -1.269147 1.230826
H -5.247665 -1.631909 -0.916151
H -4.147217 -0.247067 -0.909107
H -4.307903 -1.269544 -2.367977

I_2

54
Energy: -3966.29978967
Cu 0.263819 -1.437119 -1.959633
Cl 0.915397 -3.493218 -2.426942
C 2.071249 0.392301 -0.698732
O -0.137448 -1.738663 -0.146995
S -1.655056 -1.696675 0.052861

O -2.347440 -1.906629 -1.222530
C -1.904009 -3.226577 1.036715
O -2.090187 -0.585981 0.886568
F -3.196894 -3.341349 1.302851
F -1.499483 -4.274853 0.351472
F -1.228552 -3.140105 2.168741
C 2.688008 -0.461530 -1.623445
C 3.791959 -1.225047 -1.252442
C 4.300831 -1.126814 0.035271
C 3.713469 -0.252333 0.950003
C 2.608047 0.503586 0.588057
H 2.356599 -0.470397 -2.663319
H 4.243587 -1.899645 -1.976799
H 5.159515 -1.726964 0.330026
H 4.114433 -0.171511 1.958879
H 2.129405 1.163686 1.310453
C -1.111329 -0.677474 -4.140485
O 0.039163 -1.043962 -3.777051
C -1.702998 -1.352558 -5.362755
N -1.831683 0.210518 -3.464620
C -1.458057 0.830091 -2.194615
C -0.130455 0.403018 -1.696774
C 0.817676 1.091182 -1.055491
C 0.637398 2.502271 -0.642158
C -0.518530 2.900664 0.037419
C -0.678956 4.225898 0.429187
C 0.311203 5.161661 0.147944
C 1.472749 4.767609 -0.513336
C 1.640050 3.444167 -0.898852
H -2.780218 0.373410 -3.778234
H -1.430478 1.922627 -2.338866
H -2.258075 0.602744 -1.474350
H -1.267825 2.152669 0.299683
H -1.576013 4.524029 0.969136
H 0.184709 6.198518 0.454845
H 2.253660 5.495844 -0.725808
H 2.549929 3.127628 -1.408731
C -2.882018 -0.592017 -5.961674
C -0.607276 -1.533300 -6.412788
C -2.163155 -2.728321 -4.848926
H -2.588498 -3.299339 -5.685314
H -1.322526 -3.293701 -4.424689
H -2.930852 -2.628800 -4.068771
H -1.014074 -2.095722 -7.263087
H -0.242277 -0.567249 -6.787536
H 0.241043 -2.090676 -6.001462
H -3.209807 -1.104764 -6.874578
H -3.756864 -0.569860 -5.294515
H -2.615498 0.437442 -6.239452

TS_{rc}^{B}

54
Energy: -3966.27247286
Cu 0.237859 -1.323754 0.343937
Cl 1.256851 -3.160111 -0.322579
C 0.011656 1.906311 1.498094
O -1.554971 -1.072920 0.940999
S -2.554704 -0.839339 -0.182513
O -1.901774 -0.816569 -1.493317
C -3.488413 -2.416489 -0.147242
O -3.511546 0.210100 0.132076
F -4.404314 -2.403564 -1.103885
F -2.659249 -3.428025 -0.350350
F -4.082819 -2.565455 1.025199
C 0.187806 0.946339 2.506196
C 0.107982 1.295965 3.843027
C -0.152656 2.614829 4.207483
C -0.334900 3.578269 3.220914
C -0.255962 3.229177 1.879718
H 0.363921 -0.090255 2.234246
H 0.236081 0.530271 4.605445
H -0.222227 2.887354 5.259043
H -0.544590 4.610347 3.495792
H -0.398750 3.990072 1.114729
C 2.909340 -0.531523 -0.446185
O 2.149063 -0.309175 0.533871
C 4.346711 -0.943020 -0.222004
N 2.444667 -0.285541 -1.670812
C 1.024951 0.023011 -1.796072
C 0.543044 0.409318 -0.437754
C 0.128504 1.575214 0.075724

C -0.322424 2.577780 -0.937301
C -1.683212 2.748808 -1.198648
C -2.087780 3.702311 -2.127359
C -1.146167 4.481452 -2.793495
C 0.211163 4.309369 -2.533956
C 0.624085 3.356337 -1.609176
H 2.946968 -0.635167 -2.476994
H 0.879647 0.873359 -2.475354
H 0.480565 -0.841282 -2.202672
H -2.415372 2.125053 -0.682758
H -3.148317 3.829938 -2.335624
H -1.470136 5.224688 -3.520059
H 0.949800 4.921533 -3.048841
H 1.684580 3.215655 -1.391906
C 4.420658 -1.824999 1.023554
C 4.901732 -1.698681 -1.427345
C 5.132935 0.356327 0.003190
H 5.914701 -2.049830 -1.194476
H 4.287994 -2.578756 -1.663774
H 4.991556 -1.063187 -2.321147
H 6.184734 0.111272 0.201121
H 5.094516 1.011223 -0.878375
H 4.741542 0.909596 0.866403
H 5.465178 -2.115883 1.195048
H 4.059752 -1.291326 1.909974
H 3.813014 -2.730411 0.899873

Reaction 2

reactant

36
Energy: -712.437796218
C -4.075092 -0.033537 0.170783
O -4.672460 0.003571 -0.895925
C -4.829942 -0.077623 1.503949
N -2.715459 -0.037922 0.210105
H -2.202390 -0.067928 1.078929
C -1.960660 0.002127 -1.033772
C -0.530476 -0.011797 -0.774069
H -2.254080 -0.852120 -1.663310
H -2.248014 0.899429 -1.603421
C 0.658864 -0.023881 -0.543238
C 2.060381 -0.038819 -0.276892
C 2.762945 -1.250024 -0.213346
C 4.125889 -1.258079 0.042202
C 4.830294 -0.068766 0.245100
C 4.126432 1.136138 0.176601
C 2.763563 1.157371 -0.078412
H 2.224630 -2.182784 -0.373151
H 4.660743 -2.207588 0.084098
C 6.297726 -0.086025 0.551345
H 4.661729 2.074769 0.324408
H 2.225784 2.102451 -0.133116
C -3.930672 -0.116170 2.735387
C -5.714422 1.169442 1.554619
C -5.710287 -1.328359 1.473479
H 6.797001 0.820021 0.186029
H 6.478280 -0.139602 1.634594
H 6.794467 -0.953056 0.098497
H -4.549774 -0.145227 3.642307
H -3.292427 -1.011813 2.754374
H -3.293268 0.777080 2.810810
H -6.347215 -1.364741 2.368507
H -6.350560 -1.324517 0.584072
H -5.101392 -2.243439 1.453134
H -6.352239 1.144903 2.449408
H -5.108637 2.085906 1.595019
H -6.353795 1.221837 0.666133

I_c

71
Energy: -4313.06620441
Cu 0.698892 0.072645 -0.510747
Cl 1.350776 -0.522009 -2.513927
C 1.914572 1.528029 -0.688053
O -0.589096 -1.481247 -0.360937
S -1.430228 -1.887138 0.819258
O -2.135519 -0.767922 1.450173
C -2.761446 -2.823246 -0.026619
O -0.789823 -2.860942 1.695035

F -3.618339 -3.286363 0.866920
F -3.423364 -2.019662 -0.867935
F -2.257270 -3.830861 -0.716951
C 1.334774 2.755450 -0.432442
C 2.179473 3.866999 -0.351287
C 3.546456 3.733597 -0.549719
C 4.089491 2.482175 -0.830682
C 3.269608 1.357658 -0.905795
C -1.047053 2.997692 2.160589
O -0.165949 3.851001 2.169317
C -2.520912 3.399129 2.065099
N -0.763949 1.666743 2.251256
C 0.590946 1.229720 2.440955
C 1.087085 0.200968 1.501747
C 1.815390 -0.777781 1.268100
C 2.701198 -1.817177 0.911905
C 2.233507 -3.119114 0.653055
C 3.133749 -4.103262 0.289697
C 4.502998 -3.832895 0.179616
C 4.961496 -2.540060 0.458276
C 4.078133 -1.536966 0.813519
H 0.269667 2.877163 -0.256571
H 1.738359 4.830598 -0.101915
H 4.193934 4.606401 -0.487751
H 5.158372 2.371586 -1.008789
H 3.682972 0.382692 -1.156742
H -1.478186 0.953076 2.125456
H 1.222683 2.126266 2.348762
H 0.735445 0.826047 3.455795
H 1.169841 -3.326192 0.751251
H 2.773534 -5.111172 0.084885
C 5.454075 -4.905482 -0.249509
H 6.027666 -2.325258 0.390096
H 4.432526 -0.530623 1.032266
C -3.495554 2.233083 2.186777
C -2.778637 4.400984 3.192868
C -2.705984 4.091870 0.713176
O -3.301726 1.154065 -1.059273
C -4.520103 0.736008 -1.691432
C -5.578201 0.731453 -0.620304
C -2.102390 0.862088 -1.550204
O -1.135795 1.213727 -0.888927
C -2.009539 0.144182 -2.857545
H -4.754065 1.441604 -2.501680
H -4.396400 -0.263945 -2.123392
H -6.546900 0.437717 -1.040496
H -5.304820 0.019649 0.168699
H -5.677771 1.726502 -0.170165
H -2.736850 0.521213 -3.584809
H -0.994346 0.238001 -3.248671
H -2.202710 -0.924199 -2.692719
H -3.380409 1.697779 3.140165
H -4.524583 2.619481 2.153628
H -3.385975 1.513266 1.364921
H -3.739376 4.456353 0.616657
H -2.023532 4.945909 0.620753
H -2.512190 3.393883 -0.112084
H -3.803023 4.792373 3.117607
H -2.670718 3.926328 4.178586
H -2.074372 5.238618 3.137623
H 6.478895 -4.696718 0.079341
H 5.158208 -5.886138 0.142826
H 5.472728 -4.990330 -1.345423

I_1

57
Energy: -4005.54443318
Cu 0.025884 -0.009160 0.000991
Cl 0.012752 0.002357 2.133504
C 1.642529 0.927789 0.280630
O -1.844833 -0.781676 -0.263412
S -2.560089 0.451673 -0.768733
O -3.226927 0.295745 -2.048409
C -3.882561 0.689075 0.479643
O -1.650121 1.604976 -0.603441
F -4.574955 1.774367 0.171611
F -4.685604 -0.362715 0.474047
F -3.351778 0.831995 1.679832
C 2.869920 0.296155 0.286351
C 3.999367 1.115950 0.232541
C 3.870532 2.502284 0.201477

C 2.610841 3.091519 0.227780
C 1.463614 2.298631 0.288385
H 2.959957 -0.790645 0.305631
H 4.985487 0.653850 0.219035
H 4.760999 3.127912 0.170953
H 2.506685 4.175435 0.218917
H 0.471158 2.744704 0.339825
C 1.252401 -3.161118 0.313366
O 2.336488 -2.904096 -0.198209
C 1.141678 -3.798688 1.696312
N 0.094998 -2.949800 -0.383771
C 0.161533 -2.393957 -1.714464
C 0.445295 -0.941919 -1.774838
C 0.653663 0.147283 -2.316093
C 0.978235 1.408537 -2.864925
C -0.029745 2.338511 -3.174165
C 0.320437 3.579512 -3.675025
C 1.661063 3.923237 -3.887250
C 2.655320 2.988089 -3.581486
C 2.325714 1.742851 -3.075766
H -0.805385 -2.953899 0.079036
H 0.954215 -2.911377 -2.269140
H -0.797655 -2.573868 -2.213678
H -1.069552 2.077128 -2.988348
H -0.458261 4.306469 -3.905148
C 2.016791 5.263370 -4.452326
H 3.701882 3.250712 -3.735072
H 3.098740 1.022165 -2.813824
C -0.268186 -3.765145 2.280514
C 1.588433 -5.254679 1.516110
C 2.111549 -3.075704 2.630322
H 2.127933 -3.576257 3.608244
H 3.126307 -3.084829 2.215845
H 1.808103 -2.030914 2.786019
H 1.578054 -5.769930 2.486594
H 0.915561 -5.797110 0.836396
H 2.605074 -5.301734 1.106647
H -0.251967 -4.194758 3.291060
H -0.653965 -2.738872 2.367638
H -0.975453 -4.368721 1.692657
H 1.369687 6.053250 -4.051190
H 3.058458 5.530864 -4.239169
H 1.893446 5.272396 -5.544590

TS_{rc}^{A}

57
Energy: -4005.51975552
Cu -0.246281 -0.661733 -0.812789
Cl 0.248495 -1.345706 -2.776992
C -1.613996 -1.952842 -0.815645
O 0.838379 0.985286 -0.905448
S 1.626631 1.830321 0.060561
O 2.969832 1.305889 0.317667
C 1.869637 3.330091 -0.965569
O 0.871310 2.281956 1.227558
F 2.548180 4.227936 -0.266150
F 2.539931 3.035820 -2.065076
F 0.689042 3.839527 -1.293928
C -1.413333 -3.201703 -0.256275
C -2.498354 -4.076051 -0.192120
C -3.740349 -3.697095 -0.692240
C -3.905729 -2.441379 -1.267024
C -2.832012 -1.554521 -1.340056
H -0.439520 -3.489608 0.134580
H -2.363886 -5.060768 0.253819
H -4.580915 -4.387076 -0.641306
H -4.871903 -2.143656 -1.671719
H -2.960240 -0.571470 -1.793869
C 2.319043 -1.467401 1.232622
O 1.235013 -1.831320 0.731884
C 3.649835 -1.867553 0.637483
N 2.239783 -0.799801 2.399785
C 0.888657 -0.579800 2.891104
C -0.061552 -0.574176 1.770463
C -0.926071 -0.159186 0.952810
C -2.154830 0.624259 0.968846
C -2.256779 1.830001 0.268590
C -3.462939 2.515263 0.254316
C -4.591300 2.020355 0.913649
C -4.478951 0.804808 1.590312
C -3.277601 0.109610 1.622358

H 2.993299 -0.185921 2.684826
H 0.629733 -1.353564 3.628562
H 0.828685 0.407829 3.368091
H -1.386456 2.228894 -0.248408
H -3.532914 3.458738 -0.287745
C -5.880611 2.785265 0.904468
H -5.351186 0.392305 2.098277
H -3.207662 -0.856334 2.122704
C 3.611856 -1.643401 -0.874456
C 4.820658 -1.115939 1.262855
C 3.783039 -3.369674 0.937050
H 4.732179 -3.733238 0.521780
H 3.787268 -3.567938 2.018457
H 2.966009 -3.939234 0.477745
H 5.751786 -1.455371 0.791447
H 4.736187 -0.035209 1.095549
H 4.916195 -1.320534 2.339739
H 4.551588 -2.007528 -1.311042
H 2.780306 -2.183781 -1.343374
H 3.504216 -0.578214 -1.111248
H -6.739765 2.132348 1.101223
H -5.883908 3.569122 1.675186
H -6.046506 3.283308 -0.059144

TS_{ar}^{B}

57
Energy: -4005.53220768
Cu -0.025417 -0.705208 0.470114
Cl -0.417351 -1.120111 2.562139
C 1.661528 -1.672066 0.517969
O -1.573109 0.536077 0.358062
S -1.540273 1.975687 -0.093662
O -2.161611 2.150636 -1.411388
C -2.720941 2.706312 1.104020
O -0.262276 2.636321 0.146340
F -2.855525 3.998968 0.851956
F -3.903038 2.114171 0.982150
F -2.273039 2.540384 2.333495
C 1.680659 -2.871118 -0.182836
C 2.603442 -3.846971 0.189966
C 3.498616 -3.603572 1.225750
C 3.474871 -2.384302 1.902251
C 2.552311 -1.407522 1.553478
H 0.977998 -3.048438 -0.994364
H 2.621551 -4.796156 -0.342530
H 4.223765 -4.365119 1.506817
H 4.168545 -2.194945 2.719303
H 2.523441 -0.455394 2.079423
C -2.214124 -1.518553 -1.251652
O -1.321220 -2.207278 -0.738401
C -3.676705 -1.711915 -0.892978
N -1.896366 -0.613713 -2.215081
C -0.501770 -0.417577 -2.516003
C 0.424338 -0.251200 -1.368270
C 1.570104 -0.114392 -0.859804
C 2.832734 0.579984 -0.775800
C 2.823033 1.969618 -0.608151
C 4.026907 2.661782 -0.596221
C 5.245001 1.998446 -0.751404
C 5.235187 0.607594 -0.918814
C 4.047911 -0.102511 -0.919056
H -2.471492 0.224899 -2.277315
H -0.128200 -1.281696 -3.085670
H -0.414219 0.470297 -3.156121
H 1.870560 2.483560 -0.480177
H 4.020156 3.743577 -0.463971
C 6.541476 2.748080 -0.714862
H 6.180351 0.079772 -1.047675
H 4.047050 -1.185346 -1.039784
C -4.576479 -0.599761 -1.424238
C -4.078028 -3.048410 -1.533185
C -3.805279 -1.809276 0.627319
H -5.610618 -0.796751 -1.112484
H -4.297272 0.385235 -1.024344
H -4.573491 -0.552132 -2.522549
H -4.838013 -2.080837 0.885741
H -3.129045 -2.568026 1.037342
H -3.565114 -0.853376 1.107937
H 7.001468 2.690602 0.281813
H 7.266355 2.334237 -1.427052
H 6.401107 3.810080 -0.947875

H -5.127950 -3.267014 -1.295575
H -3.976817 -3.015352 -2.627547
H -3.457731 -3.867536 -1.149047

I_2

57
Energy: -4005.58453112
Cu 0.267363 -1.445253 -1.970005
Cl 0.924543 -3.502909 -2.426366
C 2.071504 0.393925 -0.727352
O -0.128346 -1.736306 -0.153919
S -1.645074 -1.697307 0.051136
O -2.341982 -1.918096 -1.219947
C -1.885776 -3.221334 1.046039
O -2.080845 -0.582752 0.879356
F -3.177340 -3.338323 1.318105
F -1.480895 -4.273246 0.366499
F -1.206327 -3.125561 2.175019
C 2.687482 -0.460521 -1.652024
C 3.793853 -1.221375 -1.282451
C 4.305643 -1.120098 0.003800
C 3.718802 -0.245168 0.918532
C 2.611250 0.508203 0.557974
H 2.353274 -0.471804 -2.690979
H 4.244739 -1.896630 -2.006666
H 5.166021 -1.718335 0.297552
H 4.121881 -0.162238 1.926419
H 2.132726 1.168597 1.280144
C -1.121166 -0.705825 -4.149920
O 0.032104 -1.066201 -3.789850
C -1.716773 -1.391310 -5.364582
N -1.840953 0.184878 -3.476896
C -1.463024 0.814802 -2.213162
C -0.132237 0.395335 -1.717878
C 0.815453 1.089714 -1.081944
C 0.632935 2.500473 -0.673358
C -0.524314 2.906908 -0.003132
C -0.681760 4.232434 0.386808
C 0.303422 5.183439 0.121117
C 1.467792 4.765176 -0.532518
C 1.637298 3.444666 -0.918340
H -2.791884 0.341603 -3.786265
H -1.437454 1.906324 -2.365650
H -2.260099 0.591983 -1.488252
H -1.279033 2.164376 0.259439
H -1.582329 4.531772 0.923762
C 0.127398 6.618143 0.519040
H 2.255202 5.492498 -0.734538
H 2.554064 3.130728 -1.417582
C -2.901212 -0.638862 -5.963026
C -0.625528 -1.575399 -6.418702
C -2.169681 -2.765100 -4.839388
H -2.595826 -3.343826 -5.670072
H -1.325268 -3.324068 -4.414126
H -2.934895 -2.662905 -4.057165
H -1.034535 -2.144491 -7.263509
H -0.265278 -0.610619 -6.801275
H 0.226452 -2.127436 -6.007755
H -3.231840 -1.159085 -6.870679
H -3.772801 -0.614771 -5.291688
H -2.639405 0.389490 -6.249281
H 1.047260 7.031152 0.952783
H -0.125759 7.244345 -0.348228
H -0.676564 6.737723 1.255024

TS_{rc}^{B}

57
Energy: -4005.55722256
Cu -0.007511 -0.003675 0.014784
Cl 0.028958 -0.000961 2.219636
C 0.894146 0.984171 -3.151871
O -1.327698 -0.702056 -1.169051
S -1.155774 -2.172328 -1.520131
O 0.043813 -2.744887 -0.904969
C -2.556092 -2.914056 -0.598701
O -1.404298 -2.440925 -2.927921
F -2.546758 -4.227088 -0.773816
F -2.431545 -2.645141 0.691436
F -3.702240 -2.422229 -1.039961
C -0.092639 1.785589 -2.558242

```
 C -0.732326 2.778283 -3.280241
 C -0.403540 2.994275 -4.616488
 C 0.568551 2.205016 -5.222222
 C 1.210046 1.208342 -4.499787
 H -0.376566 1.608277 -1.524988
 H -1.503027 3.377859 -2.800137
 H -0.911907 3.770627 -5.185480
 H 0.827955 2.362314 -6.267562
 H 1.970152 0.595988 -4.981234
 C 2.518410 1.174968 0.782360
 O 1.599496 1.448783 -0.035798
 C 3.057453 2.238019 1.712379
 N 3.071774 -0.035733 0.722866
 C 2.431309 -1.019251 -0.142722
 C 1.554185 -0.263122 -1.083621
 C 1.616344 -0.048324 -2.404317
 C 2.452754 -1.032634 -3.155176
 C 1.858820 -2.101890 -3.831649
 C 2.654697 -2.984774 -4.548895
 C 4.043283 -2.832620 -4.608334
 C 4.622736 -1.762324 -3.925043
 C 3.838563 -0.871131 -3.200763
 H 3.663859 -0.354547 1.479263
 H 3.186870 -1.561976 -0.725643
 H 1.868965 -1.748554 0.458157
 H 0.777042 -2.243148 -3.782957
 H 2.187919 -3.819885 -5.072228
 C 4.881645 -3.808741 -5.378067
 H 5.703394 -1.622089 -3.966401
 H 4.298663 -0.031665 -2.675916
 C 1.913041 3.161268 2.128064
 C 3.699335 1.617361 2.951292
 C 4.101618 3.021865 0.904591
 H 4.002286 2.418446 3.636950
 H 2.991007 0.969249 3.485454
 H 4.611524 1.048623 2.716247
 H 4.508117 3.828970 1.528203
 H 4.935821 2.379637 0.589733
 H 3.651574 3.472554 0.010833
 H 2.306593 3.942788 2.790967
 H 1.455721 3.643978 1.257086
 H 1.131015 2.603016 2.658271
 H 4.883233 -4.797184 -4.898162
 H 4.496862 -3.951052 -6.396451
 H 5.923722 -3.476275 -5.455187
```

Reaction 3

reactant

```
  36
Energy: -712.438974521
 C -4.057469 0.032476 0.202979
 O -4.636569 0.153476 -0.867476
 C -4.834095 -0.143405 1.512399
 N -2.698617 0.048420 0.267488
 H -2.200532 -0.047856 1.140188
 C -1.924042 0.209354 -0.953922
 C -0.498262 0.167805 -0.672996
 H -2.208111 -0.579691 -1.667432
 H -2.200911 1.158062 -1.439305
 C 0.686623 0.120558 -0.422312
 C 2.083840 0.078827 -0.134240
 C 2.678484 -1.130732 0.248183
 C 4.034424 -1.192216 0.536070
 C 4.808994 -0.040488 0.441094
 C 4.223430 1.162840 0.059838
 C 2.864480 1.249575 -0.232275
 H 2.055787 -2.021541 0.314039
 H 4.485852 -2.137210 0.832434
 H 5.874154 -0.077356 0.663812
 H 4.834594 2.062799 -0.014255
 C 2.240623 2.547115 -0.643256
 C -3.955312 -0.275126 2.752139
 C -5.743709 1.077925 1.656724
 C -5.688798 -1.402306 1.354513
 H -4.589016 -0.395976 3.641170
 H -3.300042 -1.157250 2.703503
 H -3.337051 0.619295 2.918500
 H -6.340103 -1.529721 2.230626
 H -6.313592 -1.331134 0.456990
 H -5.061635 -2.300696 1.264696
```

H -6.396293 0.962028 2.533470
H -5.156986 1.997991 1.790009
H -6.368539 1.197818 0.764447
H 2.980347 3.355903 -0.660199
H 1.789630 2.475758 -1.642482
H 1.431555 2.837292 0.040952

I_c

71
Energy: -4313.06885409
Cu 0.512494 -0.058828 -0.528593
Cl 1.157857 -0.778327 -2.504065
C 1.765830 1.374941 -0.670620
O -0.894850 -1.510145 -0.363826
S -1.903996 -1.692190 0.736212
O -2.945224 -0.656698 0.739145
C -2.760089 -3.206230 0.152758
O -1.328213 -2.043539 2.030686
F -3.685541 -3.551736 1.031777
F -3.347881 -2.974405 -1.019448
F -1.897198 -4.200174 0.013047
C 1.231464 2.561193 -0.206944
C 2.076258 3.671582 -0.140287
C 3.402160 3.574665 -0.541477
C 3.898604 2.362956 -1.011981
C 3.077461 1.236082 -1.079207
C -1.043038 2.732704 2.365294
O -0.045833 3.371177 2.677726
C -2.379999 3.432576 2.112331
N -1.000695 1.374930 2.190676
C 0.184056 0.642244 2.546920
C 0.903041 -0.081042 1.465062
C 1.792574 -0.900560 1.171703
C 2.874734 -1.732373 0.815142
C 2.680936 -3.086337 0.444191
C 3.815398 -3.834699 0.139954
C 5.090563 -3.282490 0.198492
C 5.275896 -1.950854 0.568453
C 4.170744 -1.177599 0.872020
H 0.200606 2.646651 0.122200
H 1.675767 4.602068 0.259449
H 4.053257 4.445269 -0.486584
H 4.931787 2.281971 -1.347482
H 3.458376 0.294172 -1.466631
H -1.835669 0.855238 1.932607
H 0.877865 1.380234 2.973587
H -0.052272 -0.110445 3.312030
C 1.318442 -3.686562 0.353357
H 3.691100 -4.877611 -0.149365
H 5.953017 -3.901126 -0.044896
H 6.275374 -1.524907 0.619014
H 4.277845 -0.133467 1.162133
C -3.572507 2.483637 2.068995
C -2.580189 4.459248 3.226575
C -2.245757 4.154907 0.767321
O -3.366646 1.475518 -1.231755
C -4.635618 0.971068 -1.672808
C -5.685869 1.559704 -0.768847
C -2.235423 0.884488 -1.601116
O -1.215143 1.211648 -1.008959
C -2.274577 -0.098040 -2.726006
H -4.794751 1.261704 -2.721529
H -4.629746 -0.125240 -1.605552
H -6.685849 1.279427 -1.119464
H -5.558112 1.189265 0.254748
H -5.614150 2.654384 -0.754573
H -2.936343 0.240508 -3.530976
H -1.262015 -0.253915 -3.105952
H -2.643467 -1.063732 -2.359885
H -3.676699 1.913711 3.003522
H -4.493992 3.068440 1.935120
H -3.510938 1.777511 1.230495
H -3.167305 4.715110 0.551366
H -1.408008 4.865017 0.792224
H -2.084065 3.440283 -0.051157
H -3.483832 5.052143 3.027479
H -2.705301 3.969551 4.202536
H -1.720207 5.134204 3.293679
H 0.800224 -3.326952 -0.547494
H 1.375552 -4.779651 0.299292
H 0.682759 -3.407111 1.203684

I_1

57
Energy: -4005.54410497
Cu -0.004162 0.142053 0.195926
Cl 0.048055 0.300036 2.325144
C 1.870820 0.156350 0.415887
O -2.006791 0.345606 0.012793
S -2.113152 1.722535 -0.606919
O -2.914190 1.782415 -1.815852
C -3.045853 2.665596 0.660421
O -0.760703 2.317189 -0.630192
F -3.215908 3.906441 0.231120
F -4.226393 2.098479 0.848188
F -2.378396 2.682199 1.798601
C 2.615345 -1.002903 0.521915
C 4.005094 -0.868767 0.496212
C 4.596133 0.388582 0.392889
C 3.805696 1.529995 0.312354
C 2.413432 1.425985 0.340145
H 2.147420 -1.985200 0.598937
H 4.622365 -1.763416 0.564872
H 5.681117 0.478760 0.389610
H 4.263208 2.515830 0.245398
H 1.782518 2.314127 0.313638
C -0.442277 -3.191222 0.664387
O 0.609605 -3.555052 0.150212
C -0.834950 -3.608345 2.079790
N -1.336386 -2.441430 -0.047862
C -1.026764 -2.056963 -1.403306
C -0.088537 -0.910597 -1.541005
C 0.624020 -0.178138 -2.241336
C 1.541749 0.640279 -2.929301
C 1.115205 1.735958 -3.719755
C 2.100068 2.477582 -4.368732
C 3.449761 2.166770 -4.241365
C 3.862100 1.084328 -3.465411
C 2.909565 0.318498 -2.820460
H -2.117701 -1.983501 0.404611
H -0.581597 -2.919691 -1.913640
H -1.961506 -1.785189 -1.908095
C -0.324541 2.104709 -3.856925
H 1.795914 3.321096 -4.987724
H 4.189594 2.774382 -4.760484
H 4.917943 0.841380 -3.366949
H 3.197261 -0.531780 -2.203711
C -2.030851 -2.843273 2.640848
C -1.172939 -5.101480 1.995985
C 0.382995 -3.407071 2.981564
H 0.162525 -3.777930 3.991981
H 1.248465 -3.953919 2.589587
H 0.648515 -2.343206 3.058533
H -1.416693 -5.484648 2.996576
H -2.040796 -5.278164 1.344321
H -0.322691 -5.671186 1.601301
H -2.215706 -3.167276 3.673740
H -1.852734 -1.758006 2.666042
H -2.953765 -3.045430 2.077346
H -0.653646 2.695476 -2.991848
H -0.490530 2.701562 -4.760961
H -0.982757 1.229040 -3.892265

TS_{rc}^{A}

57
Energy: -4005.52061783
Cu 0.538844 0.437383 -0.837560
Cl 0.239191 1.144032 -2.838963
C 2.130762 1.456132 -0.870868
O -1.070068 -0.699038 -0.876035
S -1.764671 -1.563257 0.142601
O -2.681022 -0.804644 1.002373
C -2.853175 -2.534177 -0.967759
O -0.904710 -2.541828 0.798830
F -3.597588 -3.351688 -0.237202
F -3.645722 -1.728539 -1.658425
F -2.116821 -3.248525 -1.805151
C 2.078931 2.760283 -0.407909
C 3.262959 3.495080 -0.362901
C 4.461734 2.930133 -0.785551
C 4.481053 1.624541 -1.261894

C 3.305628 0.875431 -1.315993
H 1.139069 3.203442 -0.087027
H 3.238211 4.521176 0.001873
H 5.381074 3.512099 -0.752342
H 5.412303 1.176631 -1.605316
H 3.329671 -0.139994 -1.708290
C -1.749571 1.867631 1.261929
O -0.673028 1.933155 0.640611
C -3.037710 2.429392 0.703751
N -1.691911 1.373788 2.518746
C -0.383749 0.864537 2.891841
C 0.390615 0.443795 1.717328
C 1.129199 -0.204078 0.922977
C 2.170845 -1.214080 1.097415
C 2.465066 -2.208266 0.142488
C 3.505084 -3.095774 0.426252
C 4.242913 -3.022014 1.600050
C 3.952610 -2.034932 2.535133
C 2.924285 -1.142841 2.279128
H -2.515376 0.910039 2.884027
H 0.165733 1.633549 3.455822
H -0.503376 -0.018846 3.535604
C 1.697876 -2.385887 -1.130327
H 3.734123 -3.871159 -0.304585
H 5.048277 -3.731821 1.780530
H 4.525431 -1.955262 3.456963
H 2.701748 -0.350677 2.993778
C -3.179856 2.006645 -0.758565
C -4.259584 1.989394 1.504265
C -2.882638 3.955935 0.790681
H -3.789284 4.431970 0.394480
H -2.749178 4.289534 1.829616
H -2.026426 4.300503 0.197737
H -5.158995 2.422412 1.048068
H -4.371400 0.897380 1.490388
H -4.225179 2.345931 2.544312
H -4.060428 2.503640 -1.187380
H -2.299780 2.287444 -1.349894
H -3.313402 0.922453 -0.846061
H 2.219330 -3.077392 -1.802030
H 0.696294 -2.787000 -0.933044
H 1.559081 -1.446804 -1.686490

TS_{ar}^{B}

57
Energy: -4005.53055703
Cu -0.170813 0.379750 0.540694
Cl 0.182824 0.476494 2.678394
C -1.983877 1.062892 0.775922
O 1.602353 -0.391478 0.234471
S 2.019203 -1.725885 -0.340190
O 2.755645 -1.565866 -1.598836
C 3.282962 -2.211398 0.896931
O 0.972725 -2.736783 -0.290858
F 3.828806 -3.361298 0.533636
F 4.226655 -1.279662 0.952236
F 2.724288 -2.342778 2.083966
C -2.203619 2.352579 0.310326
C -3.284811 3.070088 0.821223
C -4.132625 2.488654 1.756055
C -3.897570 1.188641 2.203751
C -2.816154 0.467640 1.720573
H -1.541031 2.789588 -0.432753
H -3.462295 4.086323 0.473493
H -4.983645 3.048272 2.139967
H -4.552645 0.735687 2.945481
H -2.626929 -0.543598 2.074414
C 1.705311 1.896259 -1.147334
O 0.685976 2.293321 -0.567019
C 3.081342 2.426591 -0.774364
N 1.609446 1.008790 -2.168524
C 0.336966 0.395365 -2.469493
C -0.593890 0.152736 -1.336539
C -1.737208 -0.144157 -0.896627
C -3.047550 -0.748374 -1.016315
C -3.257584 -2.088172 -0.642148
C -4.541964 -2.606950 -0.806406
C -5.579809 -1.834800 -1.315357
C -5.358969 -0.506425 -1.669941
C -4.094954 0.038736 -1.509060
H 2.410760 0.399003 -2.319546

H -0.222351 1.025289 -3.177042
H 0.544252 -0.562720 -2.965044
C -2.150507 -2.921609 -0.082136
H -4.725008 -3.643686 -0.524720
H -6.570308 -2.271720 -1.430575
H -6.169792 0.102961 -2.063433
H -3.901705 1.082131 -1.755815
C 4.226867 1.657619 -1.426096
C 3.103963 3.884995 -1.251219
C 3.224211 2.378591 0.748198
H 4.064091 4.344615 -0.980244
H 2.992052 3.951347 -2.343050
H 2.298176 4.462565 -0.782076
H 4.167539 2.861925 1.037334
H 2.397590 2.901678 1.242331
H 3.235773 1.344402 1.114646
H 5.180237 2.097394 -1.105005
H 4.238277 0.599732 -1.127834
H 4.197518 1.718123 -2.523549
H -2.499881 -3.932309 0.156126
H -1.302298 -3.005219 -0.772458
H -1.730694 -2.489212 0.838954

I_2

57
Energy: -4005.58462430
Cu 0.215987 -1.463896 -1.891670
Cl 0.955025 -3.495094 -2.342421
C 2.003293 0.413970 -0.597967
O -0.288625 -1.760882 -0.097446
S -1.809551 -1.931899 -0.023513
O -2.373414 -2.036182 -1.374217
C -1.952363 -3.600707 0.729627
O -2.435121 -1.010836 0.910939
F -3.240437 -3.873983 0.880563
F -1.398039 -4.503386 -0.048686
F -1.361892 -3.600169 1.911773
C 2.640317 -0.503998 -1.443307
C 3.744137 -1.225825 -0.997500
C 4.234732 -1.019616 0.284853
C 3.628376 -0.079691 1.118517
C 2.522318 0.634910 0.681654
H 2.329570 -0.595477 -2.485782
H 4.211492 -1.950621 -1.660883
H 5.095725 -1.584565 0.636940
H 4.017619 0.088859 2.121136
H 2.036387 1.356563 1.338127
C -1.114442 -0.691296 -4.104401
O 0.025742 -1.063938 -3.716361
C -1.669278 -1.347688 -5.353804
N -1.850876 0.193594 -3.440107
C -1.509559 0.786092 -2.149867
C -0.181889 0.375735 -1.647000
C 0.758345 1.086549 -1.024656
C 0.602169 2.536004 -0.722425
C -0.358771 3.007598 0.188740
C -0.416560 4.385402 0.424272
C 0.439869 5.272040 -0.214099
C 1.401402 4.793049 -1.100482
C 1.484349 3.429610 -1.340432
H -2.792689 0.356294 -3.773287
H -1.505813 1.882887 -2.261603
H -2.309188 0.517269 -1.441444
C -1.286324 2.088115 0.928155
H -1.150463 4.761151 1.138211
H 0.367346 6.338913 -0.008811
H 2.087422 5.478118 -1.595244
H 2.240775 3.033779 -2.018874
C -2.895303 -0.636698 -5.917560
C -0.567609 -1.409326 -6.411993
C -2.042854 -2.770330 -4.903059
H -2.447496 -3.324026 -5.761023
H -1.165802 -3.304889 -4.515193
H -2.802297 -2.751492 -4.108988
H -0.937574 -1.963930 -7.284038
H -0.272728 -0.405706 -6.748011
H 0.318959 -1.922810 -6.025277
H -3.200383 -1.135204 -6.846053
H -3.762695 -0.690467 -5.242498
H -2.692018 0.415567 -6.162388
H -1.550626 2.509434 1.905612

H -2.234239 1.932015 0.391418
H -0.860481 1.090356 1.091561

TS_{rc}^{B}

57
Energy: -4005.56002699
Cu -0.273459 -1.411613 0.313098
Cl 0.233516 -3.434977 -0.384817
C 0.405294 1.708515 1.588197
O -1.928105 -0.667404 0.895225
S -2.793720 -0.161122 -0.250567
O -2.122110 -0.317011 -1.543029
C -4.127268 -1.418931 -0.254350
O -3.428997 1.112326 0.047768
F -4.986798 -1.142311 -1.223062
F -3.605402 -2.617635 -0.462322
F -4.760153 -1.412159 0.907421
C 0.290409 0.705244 2.562542
C 0.268831 1.020359 3.909919
C 0.358894 2.348738 4.319636
C 0.464015 3.357100 3.367307
C 0.483327 3.042745 2.015308
H 0.191012 -0.331167 2.254243
H 0.167174 0.224392 4.644813
H 0.335798 2.596343 5.379364
H 0.524712 4.398690 3.677324
H 0.559467 3.838562 1.276112
C 2.549711 -1.388483 -0.387367
O 1.861218 -0.978481 0.583778
C 3.808181 -2.190713 -0.148707
N 2.205413 -0.995876 -1.614415
C 0.946825 -0.272989 -1.755986
C 0.544857 0.182593 -0.392355
C 0.469782 1.401344 0.157780
C 0.402743 2.523011 -0.830527
C -0.838008 2.998753 -1.254026
C -0.903571 4.054638 -2.157508
C 0.268797 4.630149 -2.633599
C 1.504625 4.152648 -2.206588
C 1.597990 3.092853 -1.304858
H 2.601262 -1.462479 -2.420687
H 1.085737 0.611471 -2.393568
H 0.186220 -0.916415 -2.220587
H -1.748961 2.534959 -0.871490
H -1.872894 4.422095 -2.488070
H 0.224728 5.457253 -3.340351
H 2.422020 4.613059 -2.575241
C 2.936129 2.580643 -0.853791
C 3.609429 -3.069774 1.084952
C 4.152179 -3.059701 -1.356313
C 4.920007 -1.162485 0.105781
H 5.022444 -3.682185 -1.113641
H 3.321454 -3.730641 -1.614794
H 4.432128 -2.466145 -2.239498
H 5.855221 -1.690014 0.335300
H 5.092987 -0.527591 -0.774604
H 4.672030 -0.516079 0.957904
H 4.526019 -3.645933 1.266368
H 3.398956 -2.464956 1.974145
H 2.773058 -3.765549 0.939829
H 3.740677 3.259905 -1.159305
H 2.983305 2.467802 0.238624
H 3.163677 1.591049 -1.281109

Reaction 4

reactant

36
Energy: -712.437536183
C -4.064919 0.011003 0.185068
O -4.647100 0.012562 -0.890550
C -4.834213 -0.101751 1.505844
N -2.708330 0.093770 0.245827
H -2.209942 0.110507 1.123550
C -1.937817 0.186333 -0.985271
C -0.511437 0.131318 -0.710046
H -2.243239 -0.631178 -1.655739
H -2.197522 1.115556 -1.516579
C 0.674125 0.084996 -0.465417
C 2.071873 0.023651 -0.182736

C 2.691921 -1.208290 0.058669
C 4.052362 -1.253290 0.334947
C 4.799306 -0.081822 0.371136
C 4.201693 1.159553 0.131358
C 2.838380 1.197373 -0.142301
H 2.096937 -2.119051 0.025915
H 4.534817 -2.210975 0.523489
H 5.867498 -0.123895 0.588645
C 5.021185 2.415722 0.166463
H 2.347818 2.152456 -0.331646
C -3.958403 -0.027144 2.752429
C -5.856083 1.035701 1.528869
C -5.565054 -1.445782 1.473512
H -4.587840 -0.103965 3.649368
H -3.232999 -0.852687 2.799876
H -3.416479 0.927561 2.820569
H -6.205097 -1.550056 2.360960
H -6.190531 -1.520377 0.576553
H -4.854657 -2.284811 1.466911
H -6.505565 0.942634 2.410606
H -5.360510 2.015831 1.574970
H -6.477999 1.010322 0.627085
H 4.400608 3.307221 0.017705
H 5.542989 2.527265 1.126488
H 5.791446 2.412604 -0.616872

I_c

71
Energy: -4313.06517359
Cu 0.693580 0.093632 -0.526695
Cl 1.305341 -0.481233 -2.544225
C 1.935378 1.523793 -0.725485
O -0.605245 -1.445808 -0.371096
S -1.438057 -1.871766 0.808481
O -2.160627 -0.768351 1.447768
C -2.747998 -2.824991 -0.050453
O -0.782447 -2.841273 1.677509
F -3.628067 -3.270230 0.830042
F -3.386906 -2.040364 -0.925664
F -2.225157 -3.846737 -0.705635
C 1.377161 2.764829 -0.489282
C 2.246275 3.857447 -0.403205
C 3.612819 3.691987 -0.579341
C 4.132400 2.426904 -0.843207
C 3.288569 1.320821 -0.923806
C -1.063265 3.009120 2.119215
O -0.186505 3.865735 2.074797
C -2.540598 3.402754 2.064065
N -0.770345 1.683082 2.252070
C 0.592782 1.264134 2.420208
C 1.083396 0.223680 1.493212
C 1.804306 -0.754399 1.244458
C 2.699597 -1.791666 0.889865
C 2.231665 -3.081137 0.587773
C 3.124369 -4.086018 0.233103
C 4.490424 -3.786330 0.206605
C 4.966523 -2.514873 0.517183
C 4.077818 -1.507014 0.853116
H 0.312316 2.911926 -0.332046
H 1.824218 4.833711 -0.170872
H 4.278344 4.550894 -0.514645
H 5.201123 2.291064 -1.003759
H 3.683822 0.334324 -1.158965
H -1.480263 0.961484 2.148457
H 1.212145 2.165806 2.297263
H 0.763553 0.881205 3.439041
H 1.162134 -3.277668 0.643940
C 2.630521 -5.454341 -0.129097
H 5.196878 -4.573242 -0.060628
H 6.035726 -2.314041 0.496238
H 4.424377 -0.505595 1.102968
C -3.504414 2.230634 2.207595
C -2.770103 4.395645 3.206450
C -2.768157 4.105076 0.724106
O -3.304825 1.156092 -1.014758
C -4.525603 0.716992 -1.628225
C -5.548012 0.621655 -0.526866
C -2.109033 0.915026 -1.539834
O -1.136207 1.262689 -0.885310
C -2.028579 0.256206 -2.878721
H -4.812511 1.447334 -2.398515

H -4.376509 -0.256834 -2.108643
H -6.510916 0.284285 -0.927220
H -5.208927 -0.094380 0.232494
H -5.691041 1.595959 -0.044410
H -2.777745 0.650626 -3.574026
H -1.023534 0.386062 -3.284878
H -2.200093 -0.821476 -2.756877
H -3.370437 1.702036 3.162260
H -4.536756 2.609059 2.188930
H -3.400513 1.507217 1.388429
H -3.805858 4.464159 0.660274
H -2.093308 4.963401 0.619465
H -2.593177 3.415699 -0.112242
H -3.800144 4.777429 3.167547
H -2.625080 3.915957 4.184989
H -2.076355 5.240741 3.132864
H 3.338332 -6.233667 0.179606
H 1.661761 -5.665759 0.337761
H 2.495523 -5.547210 -1.215527

I_1

57
Energy: -4005.54338908
Cu 0.011907 0.106380 0.027371
Cl 0.016233 0.315827 2.150344
C 1.878931 0.155232 0.323504
O -2.002507 0.319674 -0.265693
S -2.014712 1.702664 -0.876994
O -2.678440 1.805691 -2.163160
C -3.034832 2.658529 0.309887
O -0.649486 2.260027 -0.760643
F -3.079812 3.922386 -0.080878
F -4.260683 2.160710 0.334904
F -2.506538 2.590017 1.517729
C 2.630612 -0.995826 0.447789
C 4.017295 -0.851154 0.370201
C 4.592587 0.406195 0.203100
C 3.791320 1.539684 0.115241
C 2.402172 1.428336 0.197672
H 2.170478 -1.976752 0.574044
H 4.644454 -1.738171 0.447060
H 5.675835 0.503703 0.156040
H 4.238238 2.525910 -0.000094
H 1.761746 2.308511 0.162427
C -0.448928 -3.215294 0.711166
O 0.613528 -3.574336 0.215878
C -0.835983 -3.577544 2.142940
N -1.363353 -2.532603 -0.042313
C -1.059703 -2.226406 -1.420151
C -0.093456 -1.124325 -1.619617
C 0.630713 -0.336676 -2.230613
C 1.550786 0.525931 -2.877883
C 1.147759 1.799897 -3.306510
C 2.058431 2.658707 -3.912923
C 3.375567 2.218271 -4.081258
C 3.781293 0.951718 -3.669492
C 2.873513 0.096404 -3.065808
H -2.146585 -2.054704 0.385312
H -0.640801 -3.123526 -1.893268
H -1.992580 -1.958027 -1.929051
H 0.118953 2.114897 -3.135029
C 1.633401 4.015864 -4.388145
H 4.098726 2.889380 -4.546766
H 4.813427 0.637485 -3.811994
H 3.170854 -0.889816 -2.714321
C -2.054001 -2.819166 2.663172
C -1.138184 -5.080823 2.122394
C 0.371206 -3.309845 3.041394
H 0.155393 -3.648697 4.064004
H 1.252819 -3.847899 2.673983
H 0.608415 -2.237521 3.079168
H -1.382566 -5.424736 3.136996
H -1.995200 -5.306794 1.471664
H -0.270923 -5.646988 1.761108
H -2.234286 -3.096244 3.710380
H -1.904896 -1.730072 2.634242
H -2.968529 -3.073350 2.107207
H 2.437348 4.753127 -4.270763
H 1.364539 3.996572 -5.453489
H 0.756938 4.375550 -3.837033

TS_{rc}^{A}

```
  57
Energy: -4005.52041256
 Cu -0.370014 -0.717249 -0.739713
 Cl -0.031200 -1.287874 -2.772316
 C -1.729758 -2.019611 -0.701210
 O 0.782477 0.892214 -0.830238
 S 1.588125 1.770589 0.090387
 O 2.987881 1.354793 0.188523
 C 1.600372 3.322936 -0.886152
 O 0.924264 2.115062 1.346531
 F 2.267986 4.251316 -0.218195
 F 2.175789 3.128232 -2.058623
 F 0.352920 3.741730 -1.076298
 C -1.436625 -3.318049 -0.324130
 C -2.484164 -4.237123 -0.265287
 C -3.781084 -3.851743 -0.589372
 C -4.040801 -2.542026 -0.978325
 C -3.005987 -1.609497 -1.044499
 H -0.419260 -3.614545 -0.078425
 H -2.275292 -5.263550 0.033555
 H -4.591634 -4.577131 -0.545230
 H -5.052050 -2.235487 -1.241516
 H -3.210037 -0.584291 -1.353824
 C 2.288766 -1.545832 1.099057
 O 1.177817 -1.952548 0.700496
 C 3.576197 -1.917387 0.397403
 N 2.288384 -0.844312 2.248506
 C 0.981699 -0.670326 2.863007
 C -0.059865 -0.681432 1.829694
 C -0.954838 -0.239804 1.057705
 C -2.126602 0.619627 1.158110
 C -2.270950 1.713988 0.300832
 C -3.388616 2.541368 0.381951
 C -4.377532 2.238482 1.321232
 C -4.251031 1.137405 2.160873
 C -3.126703 0.325015 2.088722
 H 3.047287 -0.207720 2.461824
 H 0.815931 -1.454796 3.615414
 H 0.935256 0.316127 3.344486
 H -1.482254 1.941215 -0.417070
 C -3.527419 3.717502 -0.538187
 H -5.257921 2.877902 1.392185
 H -5.032340 0.912777 2.885015
 H -3.020973 -0.545241 2.734365
 C 3.423481 -1.648392 -1.100398
 C 4.785235 -1.173782 0.956049
 C 3.743522 -3.425796 0.640245
 H 4.661283 -3.766992 0.143450
 H 3.832277 -3.655593 1.711751
 H 2.898291 -3.990318 0.228488
 H 5.682443 -1.500115 0.414746
 H 4.688179 -0.090068 0.816929
 H 4.953170 -1.400076 2.019558
 H 4.334678 -1.981217 -1.615352
 H 2.569423 -2.191004 -1.524499
 H 3.281295 -0.579027 -1.296965
 H -4.110528 4.522979 -0.075101
 H -2.547547 4.122601 -0.818185
 H -4.041095 3.437577 -1.468650
```

TS_{ar}^{B}

```
  57
Energy: -4005.53168527
 Cu 0.078470 -0.731795 0.455805
 Cl -0.260142 -1.086850 2.567345
 C 1.773075 -1.685834 0.491171
 O -1.502450 0.464875 0.342968
 S -1.504880 1.904808 -0.109414
 O -2.159901 2.065223 -1.412223
 C -2.665501 2.618342 1.117845
 O -0.232714 2.586579 0.100163
 F -2.820039 3.910196 0.873002
 F -3.843476 2.013609 1.023025
 F -2.185752 2.453703 2.335370
 C 1.788998 -2.894664 -0.192325
 C 2.729466 -3.855495 0.175425
 C 3.643817 -3.587665 1.188075
 C 3.622219 -2.358565 1.846475
 C 2.682918 -1.396018 1.502815
```

H 1.071542 -3.091048 -0.986146
H 2.745974 -4.812248 -0.343403
H 4.382563 -4.337632 1.464816
H 4.331475 -2.149640 2.645132
H 2.656493 -0.436005 2.014284
C -2.129050 -1.609443 -1.214780
O -1.214194 -2.277713 -0.713581
C -3.578927 -1.815245 -0.813386
N -1.846886 -0.714481 -2.198816
C -0.461747 -0.499636 -2.530360
C 0.485551 -0.307238 -1.404271
C 1.634229 -0.150161 -0.909922
C 2.890448 0.564196 -0.850038
C 2.850911 1.950268 -0.665323
C 4.032389 2.691657 -0.655706
C 5.241513 2.017150 -0.834513
C 5.282459 0.636556 -1.018218
C 4.109722 -0.102161 -1.016385
H -2.437087 0.113662 -2.260091
H -0.085807 -1.363084 -3.099435
H -0.401554 0.383133 -3.180708
H 1.885816 2.436783 -0.516210
C 3.996476 4.180137 -0.477113
H 6.171705 2.586574 -0.823865
H 6.238695 0.134939 -1.154165
H 4.124438 -1.184226 -1.140096
C -4.507379 -0.723266 -1.337352
C -3.982269 -3.166378 -1.420594
C -3.662028 -1.890171 0.711864
H -5.020374 -3.396029 -1.144590
H -3.918435 -3.147904 -2.518113
H -3.337930 -3.971334 -1.046229
H -4.690725 -2.140484 1.005335
H -2.987719 -2.657247 1.109423
H -3.387800 -0.933700 1.174126
H -5.530748 -0.930908 -0.998263
H -4.233464 0.271347 -0.958783
H -4.531672 -0.691547 -2.436025
H 4.870867 4.539516 0.079534
H 3.995903 4.695303 -1.447876
H 3.094620 4.497245 0.058962

I_2

57
Energy: -4005.58441119
Cu 0.271931 -1.440317 -1.958571
Cl 0.920919 -3.499580 -2.418194
C 2.084188 0.391771 -0.713067
O -0.128847 -1.732930 -0.143545
S -1.645937 -1.692383 0.057952
O -2.340076 -1.905640 -1.215932
C -1.892695 -3.220372 1.045288
O -2.081806 -0.581005 0.890516
F -3.185225 -3.335245 1.313695
F -1.488828 -4.270179 0.362037
F -1.215752 -3.131234 2.176311
C 2.696266 -0.467279 -1.636053
C 3.799919 -1.231448 -1.265275
C 4.312971 -1.128821 0.020374
C 3.730201 -0.249221 0.933223
C 2.625244 0.507425 0.571553
H 2.362029 -0.479329 -2.674984
H 4.247817 -1.910081 -1.988185
H 5.171377 -1.729442 0.315014
H 4.134606 -0.164882 1.940452
H 2.150206 1.171990 1.292208
C -1.108748 -0.689134 -4.139764
O 0.041972 -1.055727 -3.777675
C -1.703996 -1.368298 -5.358101
N -1.826404 0.202665 -3.465928
C -1.449130 0.826573 -2.199031
C -0.120087 0.401474 -1.703774
C 0.830736 1.091435 -1.068589
C 0.653121 2.504014 -0.658994
C -0.499032 2.899706 0.027778
C -0.680066 4.221585 0.435342
C 0.322626 5.148796 0.143742
C 1.480172 4.764285 -0.525827
C 1.654140 3.444178 -0.920945
H -2.775650 0.364844 -3.777656
H -1.421026 1.918601 -2.346717

H -2.247270 0.602139 -1.475789
H -1.243713 2.146150 0.293880
C -1.921822 4.638211 1.166548
H 0.197657 6.184859 0.461127
H 2.256189 5.499164 -0.733958
H 2.562768 3.131910 -1.435084
C -2.884445 -0.609598 -5.956524
C -0.611098 -1.552654 -6.410461
C -2.162998 -2.742222 -4.838524
H -2.589935 -3.316354 -5.671975
H -1.321465 -3.305907 -4.413809
H -2.929150 -2.639891 -4.057221
H -1.020214 -2.117653 -7.257948
H -0.246816 -0.587870 -6.789168
H 0.238151 -2.108897 -5.999534
H -3.214933 -1.125546 -6.866658
H -3.757392 -0.584756 -5.286969
H -2.618374 0.418771 -6.238760
H -1.695403 5.352102 1.968619
H -2.636604 5.130788 0.492038
H -2.433330 3.777698 1.613771

TS_{rc}^{B}

57
Energy: -4005.55773730
Cu -0.796186 1.237856 0.286178
Cl -2.270598 2.653580 -0.543113
C 0.276146 -1.704924 1.683176
O 0.945188 1.582234 0.993951
S 2.033191 1.682234 -0.065607
O 1.505241 1.427056 -1.407552
C 2.393296 3.479374 -0.025349
O 3.265143 1.017632 0.332311
F 3.326479 3.758421 -0.923267
F 1.295965 4.162062 -0.309343
F 2.825618 3.825508 1.176308
C -0.263333 -0.791556 2.601086
C -0.218866 -1.040596 3.961972
C 0.367738 -2.209637 4.440825
C 0.911883 -3.123815 3.544616
C 0.870167 -2.874316 2.179697
H -0.700610 0.135369 2.241484
H -0.633129 -0.310208 4.653968
H 0.407674 -2.401817 5.511506
H 1.376861 -4.037728 3.909738
H 1.296590 -3.595366 1.484758
C -3.020139 -0.364430 -0.633003
O -2.322057 -0.292488 0.413022
C -4.529175 -0.409281 -0.545825
N -2.393573 -0.512596 -1.800247
C -0.941493 -0.373815 -1.805062
C -0.484078 -0.533342 -0.394033
C 0.207868 -1.491832 0.235108
C 1.058800 -2.332263 -0.660610
C 2.422102 -2.056504 -0.755653
C 3.246644 -2.814883 -1.586918
C 2.675923 -3.860019 -2.317357
C 1.317158 -4.144588 -2.221482
C 0.499405 -3.378881 -1.396845
H -2.903923 -0.377488 -2.663791
H -0.486463 -1.167447 -2.412524
H -0.652441 0.597439 -2.231117
H 2.840977 -1.223000 -0.187975
C 4.710560 -2.504979 -1.679782
H 3.308374 -4.455485 -2.976844
H 0.892146 -4.967054 -2.794798
H -0.568029 -3.590014 -1.313765
C -4.982457 0.445226 0.636789
C -5.173585 0.101577 -1.832859
C -4.901175 -1.879706 -0.308326
H -6.262103 0.134830 -1.699707
H -4.833691 1.118312 -2.073052
H -4.986598 -0.559874 -2.692179
H -5.991269 -1.963850 -0.207499
H -4.584790 -2.518298 -1.144700
H -4.441568 -2.258786 0.613411
H -6.077356 0.408299 0.706691
H -4.562596 0.074299 1.578550
H -4.666742 1.488628 0.510615
H 5.290223 -3.110392 -0.968724
H 5.104880 -2.715301 -2.681761

H 4.906072 -1.451600 -1.445213

Reaction 5

reactant

37
Energy: -787.615298972
C -4.084646 -0.102199 0.186780
O -4.675697 -0.075229 -0.883866
C -4.847228 -0.073308 1.516029
N -2.726242 -0.147130 0.234984
H -2.220134 -0.182180 1.107720
C -1.960992 -0.164086 -1.003433
C -0.533767 -0.107107 -0.734704
H -2.214675 -1.067057 -1.580805
H -2.279844 0.683422 -1.629045
C 0.653562 -0.059390 -0.496563
C 2.051257 -0.000413 -0.222348
C 2.814795 -1.175642 -0.111419
C 4.168428 -1.116637 0.153851
C 4.801393 0.121192 0.317013
C 4.058957 1.298944 0.210670
C 2.697025 1.229072 -0.056966
H 2.324214 -2.138964 -0.239279
H 4.771361 -2.018114 0.241207
O 6.128683 0.071829 0.573583
H 4.528550 2.271669 0.332758
H 2.115859 2.145762 -0.142097
C -3.959290 -0.156110 2.753461
C -5.638163 1.236244 1.540511
C -5.816687 -1.256089 1.501368
H -4.584313 -0.137204 3.656613
H -3.378398 -1.089579 2.784248
H -3.267740 0.696246 2.825416
H -6.462010 -1.227501 2.390648
H -6.446967 -1.223559 0.605575
H -5.276828 -2.213657 1.505004
H -6.276726 1.276222 2.434261
H -4.966476 2.106290 1.564101
H -6.271686 1.317769 0.649881
C 6.814538 1.287974 0.740380
H 7.858802 1.028841 0.932231
H 6.758426 1.911592 -0.164566
H 6.422499 1.859971 1.594901

I_c

72
Energy: -4388.24610007
Cu 0.690591 0.124694 -0.507591
Cl 1.357941 -0.429667 -2.520403
C 1.871919 1.609429 -0.617538
O -0.564266 -1.451304 -0.385440
S -1.416694 -1.871475 0.781080
O -2.165688 -0.768715 1.390458
C -2.696849 -2.856408 -0.087182
O -0.768244 -2.819753 1.679967
F -3.586354 -3.299839 0.785061
F -3.331298 -2.095013 -0.985684
F -2.148444 -3.880588 -0.716292
C 1.270742 2.819136 -0.327029
C 2.087196 3.950464 -0.240127
C 3.453888 3.854130 -0.462312
C 4.022015 2.621294 -0.774181
C 3.228357 1.478619 -0.856180
C -1.027910 3.002064 2.262818
O -0.134798 3.827091 2.421656
C -2.479601 3.446422 2.066140
N -0.774115 1.662528 2.229140
C 0.556516 1.176666 2.467625
C 1.076891 0.200607 1.482276
C 1.833611 -0.769679 1.280591
C 2.717567 -1.802634 0.938918
C 2.259566 -3.126360 0.740238
C 3.151552 -4.111308 0.389956
C 4.515535 -3.813157 0.233975
C 4.985709 -2.507792 0.441566
C 4.088391 -1.514846 0.785571
H 0.205011 2.905693 -0.135053
H 1.627628 4.899646 0.031137
H 4.081206 4.741113 -0.394211

H 5.090345 2.541142 -0.971472
H 3.660383 0.519308 -1.135190
H -1.500546 0.976759 2.035861
H 1.212128 2.060587 2.470552
H 0.632520 0.704156 3.459746
H 1.200452 -3.340629 0.874330
H 2.830227 -5.136801 0.223747
O 5.295692 -4.847122 -0.109505
H 6.039630 -2.267271 0.333557
H 4.435254 -0.495844 0.951627
C -3.494259 2.310212 2.143992
C -2.788448 4.483972 3.145815
C -2.544972 4.108656 0.686480
O -3.334216 1.111379 -1.078185
C -4.538006 0.637462 -1.697553
C -5.561963 0.504638 -0.600951
C -2.126825 0.881785 -1.584443
O -1.169089 1.247394 -0.919204
C -2.013458 0.214831 -2.917263
H -4.844513 1.361776 -2.466107
H -4.357854 -0.328939 -2.182295
H -6.511244 0.135992 -1.006354
H -5.202604 -0.201231 0.158319
H -5.740033 1.471117 -0.115206
H -2.750245 0.600072 -3.630969
H -0.999821 0.350775 -3.300508
H -2.178691 -0.863451 -2.794874
H -3.445039 1.783070 3.107529
H -4.507807 2.726299 2.049421
H -3.365596 1.576797 1.337226
H -3.554688 4.505957 0.507426
H -1.828019 4.937991 0.623147
H -2.317373 3.382990 -0.106158
H -3.790132 4.905904 2.982231
H -2.772005 4.032454 4.147868
H -2.053491 5.296057 3.126685
C 6.676336 -4.623385 -0.302959
H 7.102688 -5.588001 -0.586403
H 6.853314 -3.897143 -1.108483
H 7.157316 -4.271725 0.620656

I_1

58
Energy: -4080.72379540
Cu 0.135777 -0.022355 0.141199
Cl 0.111328 0.343009 2.243983
C 2.006507 -0.058244 0.372165
O -1.842629 0.244224 -0.179469
S -1.852865 1.580362 -0.893799
O -2.495042 1.564443 -2.197205
C -2.920527 2.597934 0.196559
O -0.505978 2.172870 -0.798737
F -3.001707 3.821844 -0.302007
F -4.130305 2.063768 0.250835
F -2.406760 2.650608 1.410889
C 2.712597 -1.230941 0.558244
C 4.106115 -1.145658 0.526823
C 4.739192 0.080271 0.335958
C 3.987120 1.238908 0.174175
C 2.592225 1.182920 0.201701
H 2.211550 -2.189262 0.700161
H 4.692988 -2.053886 0.656871
H 5.826729 0.132846 0.327492
H 4.477200 2.202308 0.040395
H 1.992127 2.087193 0.101102
C -0.475435 -3.280035 0.815154
O 0.595561 -3.735785 0.430467
C -1.009215 -3.551020 2.219955
N -1.269114 -2.560536 -0.035205
C -0.826599 -2.302737 -1.384002
C 0.087792 -1.140529 -1.533795
C 0.763603 -0.382522 -2.246672
C 1.582935 0.522408 -2.932424
C 1.043514 1.700979 -3.497600
C 1.866740 2.582259 -4.157429
C 3.242911 2.318811 -4.265321
C 3.791985 1.158127 -3.701602
C 2.962057 0.270732 -3.042343
H -2.059935 -2.028433 0.306769
H -0.306037 -3.197129 -1.746755
H -1.706578 -2.121263 -2.013083

H -0.020446 1.902548 -3.387882
H 1.483792 3.498410 -4.600502
O 3.954900 3.243033 -4.926234
H 4.856095 0.949732 -3.769583
H 3.372349 -0.627993 -2.583867
C -2.204672 -2.683394 2.604871
C -1.420133 -5.028035 2.231918
C 0.135616 -3.327323 3.207788
H -0.186843 -3.608882 4.219707
H 1.005786 -3.935545 2.935312
H 0.440512 -2.271633 3.228072
H -1.771338 -5.308405 3.234675
H -2.235954 -5.220979 1.520360
H -0.571138 -5.670121 1.966925
H -2.491585 -2.901838 3.642168
H -1.970913 -1.609937 2.550581
H -3.087357 -2.892800 1.982604
C 5.345685 3.050860 -5.074466
H 5.712553 3.911929 -5.637136
H 5.849911 3.013295 -4.098575
H 5.564197 2.130854 -5.634669

TS_{rc}^{A}

58
Energy: -4080.69681661
Cu -0.071711 -0.759300 0.822398
Cl -0.711220 -1.445315 2.746049
C 1.197325 -2.144624 0.856388
O -1.084033 0.934532 0.894943
S -1.672484 1.942263 -0.054993
O -3.068436 1.659115 -0.393085
C -1.710757 3.417910 1.031896
O -0.797186 2.315628 -1.165708
F -2.201347 4.444944 0.354374
F -2.464118 3.194797 2.093404
F -0.475455 3.708492 1.423478
C 0.949738 -3.352917 0.230287
C 1.974224 -4.299200 0.196108
C 3.203115 -4.030203 0.791294
C 3.415259 -2.813471 1.431553
C 2.402949 -1.855838 1.473693
H -0.012661 -3.553807 -0.236399
H 1.803233 -5.252705 -0.302136
H 3.995623 -4.776180 0.763766
H 4.369818 -2.602528 1.911520
H 2.567131 -0.902889 1.977470
C -2.598588 -1.233924 -1.333242
O -1.575934 -1.741891 -0.827070
C -3.982485 -1.503282 -0.786946
N -2.410770 -0.515190 -2.455866
C -1.029399 -0.419233 -2.901237
C -0.124410 -0.575217 -1.754173
C 0.741772 -0.272385 -0.889458
C 2.043044 0.382723 -0.842781
C 2.226061 1.610338 -0.191351
C 3.488354 2.167094 -0.111249
C 4.593866 1.508575 -0.661524
C 4.420706 0.277988 -1.296490
C 3.147929 -0.276409 -1.380553
H -3.087837 0.186665 -2.730421
H -0.832606 -1.176672 -3.673759
H -0.848227 0.581671 -3.316186
H 1.369796 2.132260 0.230431
H 3.650513 3.124288 0.379517
O 5.782538 2.139302 -0.522302
H 5.263398 -0.260473 -1.722321
H 3.011836 -1.254270 -1.842843
C -3.972142 -1.289937 0.727785
C -5.049056 -0.631973 -1.442940
C -4.258430 -2.981874 -1.102544
H -5.252136 -3.248686 -0.719395
H -4.249742 -3.172874 -2.185287
H -3.518088 -3.634144 -0.624053
H -6.026230 -0.883656 -1.011496
H -4.866257 0.433196 -1.254037
H -5.121950 -0.812486 -2.525855
H -4.961312 -1.550022 1.127999
H -3.222665 -1.921125 1.221071
H -3.756500 -0.244007 0.978093
C 6.925114 1.518283 -1.056002
H 7.765569 2.183500 -0.842623

H 7.111639 0.540591 -0.586220
H 6.840705 1.382368 -2.144893

TS_{ar}^{B}

58
Energy: -4080.71056271
Cu 0.297869 0.758943 0.469703
Cl 0.767471 1.181638 2.546377
C -1.322263 1.834485 0.528194
O 1.746300 -0.595389 0.346174
S 1.616739 -2.036704 -0.079947
O 2.226186 -2.276811 -1.393137
C 2.746737 -2.818324 1.133905
O 0.297350 -2.607235 0.167808
F 2.825231 -4.116833 0.888224
F 3.955811 -2.280644 1.023996
F 2.292427 -2.626985 2.357417
C -1.282627 3.021071 -0.193411
C -2.122265 4.065667 0.190026
C -2.995654 3.903630 1.259721
C -3.033604 2.697082 1.958533
C -2.194610 1.652203 1.597346
H -0.596937 3.135056 -1.030603
H -2.092883 5.004458 -0.360157
H -3.655291 4.719310 1.550247
H -3.708880 2.571704 2.802879
H -2.214670 0.709143 2.139740
C 2.495046 1.374675 -1.311011
O 1.669992 2.136881 -0.787610
C 3.975934 1.457008 -0.982790
N 2.085518 0.483093 -2.251520
C 0.672633 0.382569 -2.512958
C -0.231731 0.303287 -1.338048
C -1.380931 0.255563 -0.816407
C -2.677711 -0.355369 -0.694322
C -2.756037 -1.750004 -0.533114
C -3.990649 -2.365180 -0.478546
C -5.163988 -1.608234 -0.586616
C -5.091101 -0.218025 -0.742849
C -3.851532 0.399412 -0.784268
H 2.598601 -0.394422 -2.317934
H 0.346850 1.260179 -3.091290
H 0.505392 -0.508495 -3.132812
H -1.835408 -2.325366 -0.436964
H -4.081348 -3.441166 -0.348293
O -6.315059 -2.303751 -0.525246
H -5.991062 0.384971 -0.827710
H -3.787629 1.481283 -0.895760
C 4.787322 0.305797 -1.570048
C 4.454208 2.783557 -1.589321
C 4.145341 1.493827 0.537379
H 5.521328 2.922705 -1.369322
H 4.328678 2.793297 -2.681734
H 3.899005 3.629446 -1.166180
H 5.198422 1.696069 0.776874
H 3.525860 2.277545 0.988756
H 3.859860 0.537408 0.993133
H 5.840989 0.430880 -1.287901
H 4.461100 -0.670379 -1.184250
H 4.745516 0.286342 -2.668442
C -7.531574 -1.599000 -0.619218
H -8.324255 -2.347107 -0.545828
H -7.641171 -0.874795 0.201125
H -7.618761 -1.073232 -1.581268

I_2

58
Energy: -4080.76193865
Cu 0.262790 -1.457277 -1.973359
Cl 0.897768 -3.530746 -2.391790
C 2.084929 0.375829 -0.758122
O -0.140984 -1.712987 -0.153278
S -1.656895 -1.647846 0.049395
O -2.356595 -1.881480 -1.217816
C -1.921062 -3.149971 1.071014
O -2.077028 -0.512665 0.857469
F -3.214369 -3.242345 1.344416
F -1.532346 -4.219988 0.410456
F -1.240585 -3.045000 2.198615
C 2.691112 -0.495553 -1.673562

C 3.786158 -1.268316 -1.294745
C 4.296510 -1.162690 -0.008368
C 3.719753 -0.271560 0.897234
C 2.623714 0.493947 0.527405
H 2.358408 -0.511800 -2.712917
H 4.228695 -1.956726 -2.011697
H 5.147614 -1.770445 0.292804
H 4.121245 -0.185905 1.905554
H 2.152174 1.166701 1.242664
C -1.117372 -0.740379 -4.167289
O 0.030724 -1.110187 -3.801032
C -1.722642 -1.439763 -5.369346
N -1.824657 0.172708 -3.511087
C -1.439180 0.819873 -2.258240
C -0.114446 0.392419 -1.753874
C 0.838704 1.084570 -1.121969
C 0.671319 2.499789 -0.729238
C -0.495363 2.936377 -0.083775
C -0.646093 4.260656 0.286169
C 0.370274 5.184740 0.021955
C 1.546717 4.761663 -0.602698
C 1.688764 3.426996 -0.963485
H -2.773580 0.336131 -3.823101
H -1.397858 1.907907 -2.431269
H -2.241092 0.621781 -1.531378
H -1.267458 2.210385 0.173680
H -1.538040 4.608762 0.802931
O 0.128013 6.454622 0.417445
H 2.354947 5.459198 -0.807548
H 2.609583 3.096386 -1.444104
C -2.899837 -0.684939 -5.978937
C -0.635033 -1.653050 -6.421746
C -2.189365 -2.799563 -4.820604
H -2.621409 -3.388419 -5.641073
H -1.350597 -3.359376 -4.385398
H -2.953477 -2.676026 -4.040416
H -1.051235 -2.231723 -7.256492
H -0.265143 -0.698620 -6.820793
H 0.211648 -2.207125 -6.002682
H -3.237844 -1.217259 -6.876819
H -3.769809 -0.639493 -5.306637
H -2.627276 0.335312 -6.283607
C 1.130197 7.417790 0.201381
H 0.741431 8.359931 0.595662
H 2.059032 7.160762 0.732338
H 1.350857 7.539244 -0.869824

TS_{rc}^{B}

58
Energy: -4080.73428584
Cu -1.735469 0.295248 0.009969
Cl -3.518477 -0.113239 -1.222219
C 0.843903 -0.452818 2.137700
O -1.091295 1.950175 0.705913
S -0.199903 2.724082 -0.253881
O 0.007271 1.997818 -1.508864
C -1.302097 4.120939 -0.693233
O 0.957071 3.307870 0.406838
F -0.684642 4.909932 -1.559514
F -2.412676 3.654431 -1.242360
F -1.607936 4.815993 0.390285
C -0.385038 -0.225664 2.775246
C -0.469836 -0.161767 4.155301
C 0.673341 -0.320388 4.935162
C 1.900874 -0.541028 4.319461
C 1.987229 -0.605242 2.935437
H -1.279424 -0.073941 2.178045
H -1.433226 0.027163 4.624549
H 0.606784 -0.263102 6.020155
H 2.800753 -0.660967 4.920006
H 2.951183 -0.780907 2.461656
C -1.695828 -2.533484 -0.581815
O -1.538019 -1.825008 0.447044
C -2.630992 -3.721990 -0.563216
N -0.937802 -2.273482 -1.647929
C -0.143140 -1.050839 -1.620189
C -0.051483 -0.620545 -0.193979
C 0.961829 -0.568974 0.681284
C 2.317845 -0.546132 0.059649
C 3.029591 0.656258 -0.052655
C 4.298573 0.659492 -0.601182

C 4.881911 -0.531337 -1.050329
C 4.177004 -1.732953 -0.948858
C 2.898519 -1.727553 -0.396913
H -1.178027 -2.682499 -2.542125
H 0.869385 -1.246628 -1.995648
H -0.604309 -0.282418 -2.257108
H 2.570260 1.589536 0.279251
H 4.866142 1.581761 -0.704696
O 6.125305 -0.416844 -1.567514
H 4.611224 -2.671256 -1.284602
H 2.346491 -2.665160 -0.307220
C -3.197358 -3.995607 -1.955645
C -1.801789 -4.918124 -0.075373
C -3.770637 -3.439533 0.413637
H -2.448030 -5.803662 -0.013917
H -0.974140 -5.145796 -0.761258
H -1.385593 -4.728783 0.922661
H -4.448766 -4.302535 0.433115
H -3.392115 -3.273265 1.428517
H -4.334893 -2.549061 0.108948
H -3.941567 -4.799021 -1.888141
H -3.696019 -3.105361 -2.362613
H -2.431041 -4.342184 -2.665331
C 6.756758 -1.580991 -2.039690
H 7.736368 -1.270713 -2.411445
H 6.894138 -2.321111 -1.236832
H 6.189752 -2.043758 -2.861599

Reaction 6

reactant

37
Energy: -787.614514669
C -0.000072 0.100874 0.002835
O -0.029230 0.227158 1.219018
C -1.269494 -0.216339 -0.794991
N 1.165178 0.239085 -0.685621
H 1.212736 0.136920 -1.688762
C 2.392432 0.543348 0.034759
C 3.517898 0.646040 -0.879639
H 2.258546 1.478596 0.600401
H 2.571457 -0.235156 0.792303
C 4.444820 0.730390 -1.654944
C 5.540868 0.827965 -2.564511
C 5.655429 1.929786 -3.416955
C 6.729139 2.003830 -4.299441
C 7.682948 0.999997 -4.338634
C 7.573315 -0.104027 -3.485695
C 6.503999 -0.193938 -2.599726
H 4.904492 2.715790 -3.378537
H 6.823779 2.859271 -4.965971
H 8.529952 1.040428 -5.020427
O 8.554437 -1.031285 -3.598126
H 6.389806 -1.038899 -1.925750
C -1.052665 -0.339643 -2.299800
C -1.820389 -1.534114 -0.247223
C -2.264070 0.910965 -0.511736
H -2.008263 -0.566563 -2.791654
H -0.678687 0.595283 -2.742402
H -0.360211 -1.155846 -2.553173
H -3.230825 0.690397 -0.985825
H -2.417478 1.024468 0.567410
H -1.902550 1.870127 -0.908988
H -2.785301 -1.766144 -0.719599
H -1.133867 -2.368211 -0.450989
H -1.964743 -1.467756 0.837087
C 8.492845 -2.158693 -2.760803
H 9.364901 -2.771211 -3.003676
H 8.535114 -1.876779 -1.697786
H 7.579193 -2.746112 -2.938540

I_c

72
Energy: -4388.23995139
Cu 0.709355 0.101213 -0.524175
Cl 1.339161 -0.435953 -2.544917
C 1.932839 1.548794 -0.712569
O -0.574842 -1.448718 -0.384004
S -1.407516 -1.873351 0.796984
O -2.137026 -0.768788 1.427369

C -2.711350 -2.837914 -0.058606
O -0.748177 -2.832155 1.674039
F -3.589464 -3.283174 0.824002
F -3.354869 -2.059075 -0.936584
C 1.353023 2.779872 -0.476398
C 2.205025 3.883706 -0.365790
C 3.576335 3.739175 -0.521081
C 4.118085 2.483946 -0.788026
C 3.291719 1.366856 -0.892052
C -1.051545 3.008520 2.131643
O -0.174217 3.865445 2.110454
C -2.527825 3.402848 2.054692
N -0.760459 1.680931 2.250627
C 0.600946 1.260160 2.429742
C 1.096061 0.220311 1.505576
C 1.812085 -0.756102 1.243668
C 2.713768 -1.790450 0.885631
C 2.251921 -3.075307 0.588004
C 3.166322 -4.066694 0.232772
C 4.532026 -3.773102 0.190555
C 4.982884 -2.489995 0.503339
C 4.091983 -1.490090 0.845457
H 0.283482 2.910452 -0.338045
H 1.764763 4.850876 -0.129326
H 4.228418 4.606726 -0.437733
H 5.191147 2.364661 -0.931973
H 3.704417 0.387758 -1.128010
H -1.469268 0.959993 2.133677
H 1.222818 2.161051 2.314256
H 0.763155 0.874327 3.448952
H 1.190605 -3.308297 0.638450
O 2.637301 -5.276817 -0.044565
H 5.255149 -4.537682 -0.082396
H 6.050208 -2.280263 0.473161
H 4.430891 -0.486741 1.094942
C -3.494339 2.232034 2.191226
C -2.773551 4.403150 3.186944
C -2.735235 4.096786 0.706874
O -3.301016 1.140113 -1.027965
C -4.516916 0.688491 -1.642031
C -5.539498 0.584000 -0.541608
C -2.101917 0.898695 -1.545382
O -1.133434 1.250521 -0.886653
C -2.012328 0.236459 -2.882049
H -4.809965 1.415214 -2.413431
H -4.357988 -0.284378 -2.121106
H -6.498273 0.235666 -0.942532
H -5.193556 -0.127350 0.218997
H -5.693727 1.557259 -0.060636
H -2.761267 0.624210 -3.581320
H -1.006586 0.372112 -3.284656
H -2.176796 -0.841937 -2.758253
H -3.370017 1.705630 3.148400
H -4.525937 2.611766 2.161466
H -3.384048 1.506455 1.374792
H -3.771175 4.457428 0.625984
H -2.057320 4.953350 0.606203
H -2.550327 3.401468 -0.122463
H -3.802007 4.786782 3.129081
H -2.645337 3.929342 4.170648
H -2.076797 5.246226 3.119497
C 3.509409 -6.313530 -0.421589
H 2.882157 -7.189006 -0.605345
H 4.059191 -6.064815 -1.341871
H 4.229366 -6.548215 0.377205
F -2.183940 -3.858602 -0.709726

I_1

58
Energy: -4080.71820303
Cu 0.013924 0.114792 0.019366
Cl -0.001242 0.312408 2.143382
C 1.876915 0.168299 0.342074
O -2.000939 0.314700 -0.286398
S -2.008033 1.695092 -0.904830
O -2.661873 1.791847 -2.195863
C -3.034604 2.657903 0.270962
O -0.641775 2.248899 -0.778477
F -3.076349 3.919707 -0.125310
F -4.260997 2.160784 0.291052
F -2.513800 2.594061 1.482620

C 2.627542 -0.982368 0.475646
C 4.014684 -0.837441 0.410591
C 4.590736 0.419856 0.246203
C 3.789921 1.552953 0.149388
C 2.400179 1.441640 0.220218
H 2.166278 -1.963046 0.599620
H 4.641487 -1.724033 0.494804
H 5.674335 0.517735 0.208672
H 4.237603 2.539137 0.036485
H 1.759420 2.321256 0.177480
C -0.449849 -3.214704 0.696865
O 0.616214 -3.569574 0.206313
C -0.842481 -3.579585 2.126485
N -1.363405 -2.535053 -0.060354
C -1.053196 -2.225262 -1.435884
C -0.080805 -1.128354 -1.627993
C 0.652774 -0.332573 -2.214381
C 1.597894 0.515598 -2.850883
C 1.221063 1.788015 -3.285238
C 2.166936 2.612582 -3.895793
C 3.477575 2.158696 -4.065821
C 3.838063 0.881840 -3.633709
C 2.913368 0.050547 -3.029079
H -2.146707 -2.055006 0.364857
H -0.636650 -3.122208 -1.911565
H -1.982675 -1.950199 -1.947349
H 0.207350 2.153135 -3.133056
O 1.721040 3.826135 -4.286109
H 4.225739 2.791887 -4.536532
H 4.862881 0.543321 -3.773725
H 3.185363 -0.940647 -2.673219
C -2.062957 -2.822670 2.643117
C -1.144023 -5.082947 2.102028
C 0.361013 -3.313025 3.030149
H 0.140608 -3.651763 4.051815
H 1.243753 -3.851755 2.666424
H 0.598974 -2.240883 3.068942
H -1.392113 -5.428858 3.115060
H -1.998484 -5.307952 1.447627
H -0.275191 -5.648154 1.742971
H -2.247599 -3.102017 3.688974
H -1.914094 -1.733482 2.616955
H -2.974983 -3.075985 2.082709
C 2.630437 4.698584 -4.909807
H 2.071487 5.607929 -5.142862
H 3.469584 4.952639 -4.244478
H 3.027481 4.270605 -5.842851

TS_{rc}^{A}

58
Energy: -4080.69581781
Cu -0.004377 -0.821353 -0.785608
Cl 0.581312 -1.338470 -2.774708
C -1.009624 -2.409087 -0.788351
O 0.646081 1.038393 -0.878376
S 1.238123 2.049154 0.067323
O 2.683748 1.892301 0.242487
C 1.023729 3.575596 -0.925095
O 0.458661 2.269732 1.284935
F 1.494054 4.606263 -0.237531
F 1.679315 3.478521 -2.067982
F -0.264288 3.769785 -1.171270
C -0.499486 -3.573103 -0.242963
C -1.337667 -4.685510 -0.167231
C -2.643709 -4.618522 -0.643334
C -3.120594 -3.438906 -1.205415
C -2.297181 -2.316854 -1.289003
H 0.522124 -3.615669 0.129129
H -0.959497 -5.609371 0.268739
H -3.289729 -5.492784 -0.583870
H -4.137616 -3.384041 -1.590878
H -2.667613 -1.392548 -1.732911
C 2.765198 -0.973111 1.147177
O 1.788305 -1.597882 0.685680
C 4.130523 -1.031710 0.500233
N 2.568043 -0.337639 2.318341
C 1.223993 -0.445057 2.863397
C 0.257597 -0.703562 1.786795
C -0.722812 -0.535352 1.015162
C -2.115758 -0.109207 1.100509
C -2.531157 1.094697 0.543772

C -3.881534 1.451365 0.599639
C -4.810618 0.594874 1.192862
C -4.376859 -0.615258 1.731354
C -3.040976 -0.979879 1.692979
H 3.156639 0.446889 2.571804
H 1.191565 -1.237785 3.624903
H 0.938648 0.512379 3.320569
H -1.823270 1.781978 0.085987
O -4.187738 2.646530 0.046300
H -5.864727 0.855910 1.238112
H -5.104208 -1.284691 2.187612
H -2.704662 -1.938895 2.084441
C 3.979599 -0.831572 -1.007899
C 5.100250 -0.008866 1.083380
C 4.645150 -2.451770 0.787143
H 5.638515 -2.569303 0.334479
H 4.739578 -2.637388 1.866677
H 3.978964 -3.209233 0.356638
H 6.067749 -0.108044 0.574851
H 4.742251 1.015664 0.924209
H 5.284983 -0.177600 2.154878
H 4.962064 -0.956052 -1.482825
H 3.288069 -1.562058 -1.445413
H 3.604540 0.173089 -1.236559
C -5.530604 3.060028 0.066771
H -5.558423 4.037356 -0.421392
H -6.178398 2.362868 -0.486310
H -5.908141 3.160465 1.095793

TS_{ar}^{B}

58
Energy: -4080.70972105
Cu 0.167351 -0.917198 0.402424
Cl -0.266852 -1.671684 2.390628
C 1.890732 -1.795786 0.382778
O -1.452040 0.226179 0.449763
S -1.371974 1.732736 0.389882
O -1.812405 2.247253 -0.913209
C -2.716538 2.162513 1.561377
O -0.151897 2.278971 0.970483
F -2.837180 3.481118 1.618506
F -3.866368 1.642255 1.144074
F -2.437685 1.693091 2.761648
C 2.047468 -2.846874 -0.509991
C 3.030825 -3.800286 -0.253004
C 3.849482 -3.677354 0.864490
C 3.689555 -2.600969 1.735566
C 2.706740 -1.646562 1.498953
H 1.405908 -2.931197 -1.384860
H 3.157880 -4.635293 -0.939652
H 4.620810 -4.420848 1.056982
H 4.321789 -2.507043 2.616718
H 2.572063 -0.807527 2.178439
C -2.004390 -1.556234 -1.431116
O -1.081677 -2.282235 -1.039168
C -3.454608 -1.855299 -1.094202
N -1.728663 -0.504834 -2.251152
C -0.341568 -0.232594 -2.525266
C 0.576101 -0.191727 -1.364493
C 1.718235 -0.025810 -0.863351
C 2.850450 0.834113 -0.687443
C 2.621448 2.209275 -0.957875
C 3.657636 3.123785 -0.757827
C 4.910689 2.675018 -0.362755
C 5.162651 1.317955 -0.168040
C 4.133013 0.406139 -0.327328
H -2.327002 0.314197 -2.180186
H 0.047132 -0.994638 -3.216868
H -0.269921 0.745702 -3.019782
O 1.417526 2.534631 -1.438850
H 3.489023 4.183296 -0.931462
H 5.710129 3.400354 -0.220255
H 6.155317 0.976299 0.114865
H 4.308319 -0.655204 -0.168509
C -4.401080 -0.712549 -1.448792
C -3.815662 -3.095464 -1.924121
C -3.561841 -2.181562 0.395613
H -4.852629 -3.389201 -1.711807
H -3.732822 -2.894657 -3.002047
H -3.158485 -3.937283 -1.674050
H -4.584744 -2.513907 0.620085

H -2.863231 -2.976785 0.679877
H -3.334132 -1.303180 1.012281
H -5.426004 -0.999323 -1.178774
H -4.165731 0.204930 -0.891756
H -4.400871 -0.490654 -2.525490
C 1.006739 3.890412 -1.398520
H -0.062295 3.878497 -1.623895
H 1.154072 4.305951 -0.393763
H 1.549542 4.487532 -2.145251

I_2

58
Energy: -4080.76083411
Cu 0.265382 -1.434553 -1.963499
Cl 0.893229 -3.500352 -2.424180
C 2.092204 0.376072 -0.716010
O -0.128642 -1.723503 -0.147631
S -1.644654 -1.660788 0.060316
O -2.346169 -1.874176 -1.209595
C -1.905699 -3.179718 1.057780
O -2.061379 -0.538127 0.886607
F -3.198676 -3.278921 1.330036
F -1.515094 -4.238266 0.379972
F -1.224926 -3.091438 2.186593
C 2.698572 -0.488938 -1.636900
C 3.788955 -1.269276 -1.260176
C 4.294051 -1.176876 0.029319
C 3.717054 -0.291450 0.940292
C 2.625341 0.481300 0.572714
H 2.370280 -0.494760 -2.677823
H 4.232086 -1.952880 -1.981341
H 5.141623 -1.790416 0.328686
H 4.115139 -0.215541 1.950710
H 2.153522 1.149943 1.291729
C -1.114448 -0.676143 -4.140762
O 0.035820 -1.048500 -3.782361
C -1.719218 -1.354531 -5.354809
N -1.823353 0.221635 -3.466044
C -1.434760 0.848834 -2.204443
C -0.109851 0.411271 -1.709260
C 0.849468 1.092149 -1.077412
C 0.698885 2.512340 -0.683333
C -0.455912 2.940516 -0.034541
C -0.598581 4.279827 0.343164
C 0.422251 5.190717 0.071807
C 1.582236 4.748620 -0.565267
C 1.734102 3.423373 -0.938181
H -2.773180 0.389308 -3.773072
H -1.395359 1.939727 -2.358039
H -2.232607 0.638696 -1.476575
H -1.240167 2.234705 0.240559
O -1.757111 4.589384 0.970293
H 0.332261 6.235420 0.358499
H 2.378718 5.462673 -0.768601
H 2.643474 3.081746 -1.430211
C -2.895732 -0.588258 -5.951379
C -0.631482 -1.550502 -6.410397
C -2.187274 -2.723320 -4.829875
H -2.621663 -3.296247 -5.660316
H -1.348795 -3.292592 -4.406560
H -2.949837 -2.612807 -4.046203
H -1.048047 -2.114491 -7.254935
H -0.260754 -0.589694 -6.792963
H 0.214686 -2.112403 -6.000803
H -3.233168 -1.103985 -6.859094
H -3.766223 -0.554877 -5.279048
H -2.622640 0.437322 -6.237026
C -1.933246 5.911369 1.414831
H -2.908637 5.943266 1.906651
H -1.155558 6.202130 2.137155
H -1.928807 6.625497 0.577136

TS_{rc}^{B}

58
Energy: -4080.73282186
Cu -1.308460 1.090836 0.143599
Cl -3.018288 1.929490 -0.964675
C 0.430073 -1.311679 1.906676
O 0.168483 1.963780 0.965444
S 1.342578 2.214892 0.028944

O 1.088993 1.684487 -1.312472
C 1.243646 4.035762 -0.158409
O 2.623748 1.933294 0.655683
F 2.208503 4.446939 -0.967351
F 0.070315 4.364249 -0.676455
F 1.381455 4.622388 1.019492
C -0.425151 -0.530731 2.698507
C -0.418246 -0.643881 4.078049
C 0.445618 -1.540326 4.702587
C 1.303510 -2.319035 3.932819
C 1.298935 -2.205867 2.549459
H -1.083888 0.190175 2.224180
H -1.082463 -0.017506 4.670048
H 0.455199 -1.623847 5.787891
H 1.986292 -3.018254 4.412084
H 1.973395 -2.819569 1.955486
C -2.908188 -1.163258 -0.726674
O -2.346460 -0.811541 0.345029
C -4.350305 -1.616950 -0.721440
N -2.167048 -1.228128 -1.832416
C -0.816499 -0.682622 -1.761588
C -0.465438 -0.579394 -0.315100
C 0.416115 -1.244219 0.443749
C 1.511350 -1.919218 -0.318967
C 2.760940 -1.322937 -0.416327
C 3.787560 -1.978678 -1.106559
C 3.551751 -3.221927 -1.696894
C 2.289590 -3.804651 -1.592410
C 1.264418 -3.165598 -0.910975
H -2.619012 -1.310938 -2.734230
H -0.105403 -1.365398 -2.245668
H -0.770902 0.288582 -2.274310
H 2.958458 -0.343255 0.023124
O 4.969011 -1.327653 -1.148011
H 4.338618 -3.741813 -2.237448
H 2.114387 -4.777915 -2.048346
H 0.279065 -3.623608 -0.817727
C -5.119126 -0.805868 0.320791
C -4.992715 -1.440227 -2.095556
C -4.331032 -3.100674 -0.327697
H -6.055586 -1.704447 -2.030496
H -4.926626 -0.397963 -2.436567
H -4.554192 -2.103395 -2.856357
H -5.362409 -3.474173 -0.279730
H -3.781342 -3.707175 -1.060852
H -3.869185 -3.241212 0.657875
H -6.165912 -1.136287 0.331478
H -4.700116 -0.949768 1.323078
H -5.083972 0.266064 0.087719
C 6.031827 -1.923251 -1.848851
H 6.876380 -1.234841 -1.767320
H 6.313609 -2.893039 -1.410887
H 5.785479 -2.067110 -2.911826

Reaction 7

reactant

37
Energy: -787.613769379
C -4.017257 -0.033527 0.168143
O -4.606970 -0.117953 -0.900305
C -4.779118 -0.042832 1.498228
N -2.661420 0.059464 0.214390
H -2.158549 0.152486 1.084759
C -1.895414 0.065789 -1.023585
C -0.468394 0.038181 -0.750072
H -2.200886 -0.799288 -1.631228
H -2.160679 0.953571 -1.619197
C 0.717726 0.025513 -0.505189
C 2.112926 -0.024037 -0.223950
C 2.766925 -1.252806 -0.091575
C 4.127403 -1.318941 0.181002
C 4.848758 -0.140131 0.323872
C 4.224442 1.098593 0.197805
C 2.859175 1.165826 -0.074955
H 2.178785 -2.161245 -0.210079
H 4.619689 -2.283907 0.279826
H 5.916009 -0.174825 0.536930
H 4.810213 2.006857 0.313745
O 2.166531 2.317033 -0.212694
C -3.894620 0.106530 2.731894

C -5.784018 1.108840 1.445883
C -5.529911 -1.374309 1.565492
H -4.518954 0.094975 3.635678
H -3.175593 -0.720134 2.828897
H -3.344447 1.058961 2.733907
H -6.168150 -1.404345 2.459888
H -6.159315 -1.504363 0.677737
H -4.832030 -2.222142 1.617925
H -6.427889 1.090210 2.336506
H -5.273461 2.081966 1.416909
H -6.413315 1.027381 0.552498
C 2.866073 3.529842 -0.087368
H 2.131025 4.324221 -0.239132
H 3.313348 3.638420 0.912296
H 3.657488 3.620994 -0.846512

$\mathbf{I_c}$

72
Energy: -4388.24336188
Cu 0.431815 -0.212065 -0.359366
Cl 0.991401 -1.012487 -2.324183
C 1.762696 1.137821 -0.630524
O -1.086905 -1.544458 -0.195556
S -2.005014 -1.886440 0.944935
O -2.905917 -0.785974 1.313909
C -3.116565 -3.081067 0.108444
O -1.377313 -2.625030 2.030806
F -4.034952 -3.506970 0.957978
F -3.730502 -2.487622 -0.919072
C 1.296557 2.387427 -0.275089
C 2.207342 3.446198 -0.287892
C 3.529939 3.236710 -0.656261
C 3.956800 1.962696 -1.016396
C 3.067190 0.887419 -1.006130
C -1.016129 2.733176 2.317361
O 0.076859 3.186844 2.635244
C -2.192771 3.653804 1.975170
N -1.237202 1.390747 2.241653
C -0.239787 0.457758 2.678222
C 0.705917 -0.088669 1.664459
C 1.702207 -0.797641 1.440088
C 2.840008 -1.552403 1.106797
C 2.664139 -2.786231 0.428030
C 3.787533 -3.493536 -0.003259
C 5.055278 -2.996720 0.274275
C 5.241764 -1.795057 0.962311
C 4.134982 -1.069009 1.362417
H 0.271359 2.558684 0.036688
H 1.863571 4.430039 0.028419
H 4.232607 4.068139 -0.660817
H 4.987288 1.790068 -1.324407
H 3.396543 -0.104977 -1.306073
H -2.106975 0.995624 1.895047
H 0.374744 0.956584 3.439061
H -0.739215 -0.409134 3.135502
O 1.401985 -3.150530 0.235411
H 3.673618 -4.428532 -0.545360
H 5.923500 -3.562913 -0.059763
H 6.244611 -1.427631 1.164953
H 4.239064 -0.108117 1.863530
C -3.539498 2.937733 1.941310
C -2.224427 4.762542 3.026430
C -1.902968 4.260360 0.599110
O -3.450017 1.125680 -0.986185
C -4.699070 0.608056 -1.471438
C -5.648031 0.562190 -0.302430
C -2.282739 0.768487 -1.510039
O -1.271953 1.127394 -0.923503
C -2.272592 -0.023364 -2.778789
H -5.057340 1.267546 -2.275206
H -4.552034 -0.395596 -1.885212
H -6.617657 0.161025 -0.620134
H -5.232882 -0.083341 0.481297
H -5.807730 1.562987 0.116143
H -3.013468 0.347536 -3.496120
H -1.269891 0.010084 -3.209616
H -2.500847 -1.073253 -2.552167
H -3.765484 2.445278 2.897498
H -4.336674 3.670155 1.748378
H -3.586033 2.190028 1.138060
H -2.685954 4.986192 0.336537

H -0.937599 4.784905 0.602107
H -1.883177 3.482425 -0.177576
H -2.998103 5.499483 2.768863
H -2.458163 4.360125 4.022089
H -1.255278 5.269550 3.085826
C 1.103739 -4.205579 -0.657459
H 0.015535 -4.209139 -0.744327
H 1.548221 -4.011464 -1.642778
H 1.451995 -5.168980 -0.259535
F -2.436869 -4.122086 -0.353008

I_1

58
Energy: -4080.72423557
Cu 0.118452 0.102961 0.085810
Cl 0.112608 0.302970 2.215010
C 1.986721 0.061192 0.350670
O -1.853055 0.407858 -0.194168
S -1.903870 1.829832 -0.714218
O -2.766073 2.020529 -1.867065
C -2.740045 2.730862 0.649752
O -0.529533 2.363327 -0.752903
F -2.894521 3.997527 0.290209
F -3.928097 2.190761 0.871163
F -2.019828 2.676748 1.753872
C 2.702406 -1.115750 0.459665
C 4.095172 -1.014806 0.455586
C 4.717785 0.228800 0.371959
C 3.955905 1.389239 0.289903
C 2.561426 1.317880 0.291960
H 2.208804 -2.086423 0.523955
H 4.689944 -1.924552 0.524618
H 5.804585 0.292608 0.385305
H 4.437741 2.364485 0.238509
H 1.952895 2.221288 0.258699
C -0.455458 -3.207182 0.682762
O 0.605699 -3.635812 0.243410
C -0.946803 -3.555576 2.085845
N -1.275740 -2.449839 -0.106922
C -0.883131 -2.144955 -1.461801
C 0.057742 -1.005366 -1.605177
C 0.773737 -0.264417 -2.292397
C 1.626566 0.638326 -2.941326
C 1.079542 1.771652 -3.600068
C 1.947751 2.723320 -4.140102
C 3.321066 2.529322 -4.054480
C 3.868933 1.403213 -3.437151
C 3.020970 0.464251 -2.881650
H -2.065663 -1.949378 0.280945
H -0.407201 -3.036566 -1.887866
H -1.783874 -1.909505 -2.040994
O -0.247118 1.824524 -3.668786
H 1.555831 3.608013 -4.633973
H 3.981903 3.279478 -4.486699
H 4.946943 1.274304 -3.377869
H 3.410015 -0.409142 -2.359862
C -2.139280 -2.722015 2.547445
C -1.344178 -5.035491 2.031930
C 0.223474 -3.372964 3.051891
H -0.068228 -3.708692 4.056603
H 1.089732 -3.959139 2.724173
H 0.521849 -2.317462 3.117023
H -1.665382 -5.370819 3.027864
H -2.177401 -5.199950 1.333491
H -0.497040 -5.654163 1.711294
H -2.394812 -2.995509 3.579899
H -1.917017 -1.644880 2.539898
H -3.036917 -2.910131 1.939838
C -0.860886 3.028271 -4.095314
H -1.930451 2.878750 -3.944813
H -0.523626 3.868263 -3.473156
H -0.643295 3.226294 -5.154256

TS_{rc}^A

58
Energy: -4080.71130898
Cu 0.596313 0.526090 -0.596116
Cl 0.220029 0.959232 -2.673316
C 2.122045 1.630849 -0.718103
O -1.180785 -0.340550 -0.297181

S -1.675853 -1.661409 0.227636
O -2.623708 -1.485310 1.334999
C -2.695454 -2.194036 -1.197435
O -0.652201 -2.686215 0.380278
F -3.286058 -3.347729 -0.929326
F -3.623165 -1.280880 -1.458668
F -1.918658 -2.338992 -2.262126
C 1.989058 2.967106 -0.379486
C 3.118779 3.781787 -0.454008
C 4.340544 3.261392 -0.867173
C 4.438935 1.919042 -1.215319
C 3.320152 1.088826 -1.150331
H 1.032536 3.371961 -0.057976
H 3.032246 4.834285 -0.186923
H 5.216215 3.905588 -0.925414
H 5.388842 1.505796 -1.551811
H 3.393697 0.044710 -1.447368
C -1.804442 1.845778 1.314341
O -0.652814 2.217163 1.021501
C -3.029681 2.321674 0.564182
N -1.939075 1.090657 2.433688
C -0.655451 0.826890 3.070220
C 0.383887 0.698232 2.043223
C 1.138488 0.081191 1.231857
C 2.042131 -1.047696 1.372178
C 2.242367 -1.906610 0.274223
C 3.024811 -3.047619 0.405753
C 3.640987 -3.318664 1.623675
C 3.481478 -2.464875 2.710185
C 2.678197 -1.340348 2.580958
H -2.608889 0.320392 2.392723
H -0.429596 1.630135 3.784895
H -0.709833 -0.127295 3.613932
O 1.637439 -1.531789 -0.874600
H 3.153153 -3.725135 -0.434402
H 4.256465 -4.211401 1.718737
H 3.973070 -2.678182 3.656595
H 2.538797 -0.661398 3.421609
C -2.747078 2.366191 -0.935554
C -4.256161 1.458169 0.846466
C -3.273325 3.746895 1.088668
H -4.155736 4.167304 0.588136
H -3.460703 3.750861 2.171527
H -2.415045 4.397473 0.878122
H -5.106880 1.857889 0.279377
H -4.105901 0.416474 0.535088
H -4.537926 1.474319 1.908671
H -3.602321 2.829888 -1.445350
H -1.850287 2.956199 -1.161960
H -2.598638 1.360030 -1.345765
C 1.481232 -2.457876 -1.936631
H 0.910166 -1.924836 -2.702818
H 2.457143 -2.751865 -2.347098
H 0.921489 -3.334547 -1.587024

TS_{ar}^{B}

58
Energy: -4080.70698754
Cu -0.227446 -0.824543 0.461223
Cl -0.641679 -1.137908 2.566189
C 1.274952 -2.057412 0.503570
O -1.569999 0.629503 0.353193
S -1.308347 2.047644 -0.096625
O -1.913676 2.324585 -1.403999
C -2.338229 2.955893 1.118826
O 0.063891 2.486648 0.125059
F -2.297891 4.250354 0.844896
F -3.595031 2.534226 1.040753
F -1.881981 2.747831 2.338636
C 1.089718 -3.249104 -0.184853
C 1.852606 -4.355985 0.184408
C 2.793078 -4.249248 1.202962
C 2.975648 -3.036294 1.866108
C 2.214574 -1.928096 1.520979
H 0.353766 -3.318439 -0.983214
H 1.710053 -5.300514 -0.337558
H 3.392798 -5.114163 1.480536
H 3.705760 -2.952562 2.668885
H 2.347869 -0.978236 2.034859
C -2.543503 -1.290015 -1.230249
O -1.762194 -2.109515 -0.727543

C -4.011623 -1.244745 -0.845095
N -2.104192 -0.450902 -2.205365
C -0.700867 -0.472829 -2.528568
C 0.258685 -0.465841 -1.396788
C 1.410577 -0.515673 -0.890279
C 2.781960 -0.055126 -0.823665
C 3.010266 1.297264 -0.585317
C 4.324688 1.776936 -0.570549
C 5.387046 0.901927 -0.798958
C 5.134949 -0.451270 -1.034469
C 3.844022 -0.946203 -1.039427
H -2.543954 0.466157 -2.263630
H -0.475247 -1.378630 -3.111099
H -0.485649 0.397676 -3.162369
H 2.180946 1.980634 -0.402693
O 4.457891 3.098447 -0.329494
H 6.413795 1.259078 -0.793570
H 5.972738 -1.123101 -1.211439
H 3.642513 -2.003140 -1.204550
C -4.726254 0.005412 -1.351046
C -4.639393 -2.490377 -1.486493
C -4.126090 -1.334476 0.676745
H -5.705488 -2.538020 -1.226122
H -4.557975 -2.461354 -2.582605
H -4.153084 -3.404289 -1.123620
H -5.186226 -1.408780 0.955337
H -3.599447 -2.214057 1.064038
H -3.697830 -0.447787 1.160104
H -5.772880 -0.023037 -1.020602
H -4.282424 0.927572 -0.950347
H -4.736570 0.062745 -2.448878
C 5.755983 3.637959 -0.289532
H 5.638345 4.703033 -0.076394
H 6.361578 3.176039 0.504826
H 6.274053 3.518155 -1.253166

I_2

58
Energy: -4080.76308535
Cu 0.252129 -1.369473 -1.908685
Cl 0.942266 -3.433971 -2.288010
C 2.039779 0.465808 -0.692907
O -0.267624 -1.603486 -0.104318
S -1.787446 -1.730713 0.003967
O -2.389388 -1.842778 -1.329198
C -1.957634 -3.385431 0.781813
O -2.377181 -0.791345 0.944305
F -3.248925 -3.617852 0.975992
F -1.457036 -4.316105 -0.000888
F -1.332415 -3.392253 1.946430
C 2.660609 -0.410752 -1.593621
C 3.747424 -1.184258 -1.190920
C 4.234641 -1.073525 0.103281
C 3.642184 -0.177766 0.994889
C 2.554336 0.587361 0.602377
H 2.350308 -0.426810 -2.639885
H 4.200788 -1.876992 -1.896807
H 5.079928 -1.680263 0.422258
H 4.026401 -0.087621 2.009553
H 2.072291 1.265782 1.305249
C -1.120133 -0.693714 -4.119183
O 0.033799 -1.040507 -3.748000
C -1.712322 -1.415800 -5.314371
N -1.839407 0.219771 -3.476596
C -1.463056 0.881484 -2.230330
C -0.136139 0.477759 -1.722448
C 0.803836 1.178031 -1.086120
C 0.653660 2.602130 -0.705887
C -0.458780 3.043478 0.040803
C -0.543189 4.378681 0.440896
C 0.469117 5.270662 0.102040
C 1.577906 4.846562 -0.621953
C 1.664833 3.514967 -1.008900
H -2.794571 0.355584 -3.782268
H -1.435273 1.968115 -2.413353
H -2.251829 0.688039 -1.489051
O -1.385639 2.108907 0.340471
H -1.396455 4.723427 1.019990
H 0.387628 6.309513 0.417949
H 2.372348 5.544607 -0.876629
H 2.534863 3.157111 -1.559818

C -2.930473 -0.711636 -5.903509
C -0.631388 -1.574071 -6.383573
C -2.111870 -2.797105 -4.767562
H -2.538385 -3.396187 -5.583556
H -1.242209 -3.325636 -4.354937
H -2.861033 -2.709223 -3.968517
H -1.031118 -2.170909 -7.213654
H -0.314422 -0.602005 -6.785779
H 0.247201 -2.087233 -5.978476
H -3.259843 -1.257050 -6.796753
H -3.789486 -0.705304 -5.215743
H -2.707498 0.319746 -6.211407
C -2.396997 2.415440 1.275104
H -2.923720 1.474395 1.449959
H -1.964971 2.778319 2.218792
H -3.092974 3.171965 0.882031

TS_{rc}^{B}

58
Energy: -4080.73545509
Cu 0.746789 1.382060 0.297613
Cl 0.753567 3.458990 -0.440671
C -0.662077 -1.419016 1.719000
O 2.206661 0.280995 0.838706
S 2.861570 -0.492129 -0.296243
O 2.196004 -0.248973 -1.578060
C 4.465327 0.385858 -0.426677
O 3.171616 -1.865347 0.070062
F 5.182393 -0.153778 -1.401118
F 4.249730 1.663117 -0.699243
F 5.131803 0.287694 0.712024
C -0.229796 -0.457341 2.644475
C -0.277644 -0.708114 4.005088
C -0.758145 -1.927686 4.475999
C -1.185566 -2.894397 3.571528
C -1.135293 -2.645541 2.207021
H 0.170873 0.487442 2.288189
H 0.075475 0.049322 4.701961
H -0.789104 -2.126384 5.545826
H -1.556222 -3.853155 3.929798
H -1.467658 -3.408750 1.505443
C -2.004401 2.001601 -0.348885
O -1.386847 1.488937 0.622593
C -3.037229 3.079708 -0.114974
N -1.800813 1.496094 -1.562450
C -0.755214 0.490982 -1.701109
C -0.438519 0.003285 -0.326726
C -0.661577 -1.170299 0.276734
C -0.877808 -2.314831 -0.655494
C 0.166897 -3.167022 -1.001076
C -0.062555 -4.254362 -1.840524
C -1.340916 -4.483176 -2.330923
C -2.399120 -3.638118 -1.997932
C -2.168892 -2.547866 -1.161760
H -2.126163 1.994829 -2.380340
H -1.119566 -0.354913 -2.298608
H 0.123013 0.918377 -2.205576
H 1.164808 -2.965075 -0.607168
H 0.758888 -4.914166 -2.109607
H -1.530194 -5.330766 -2.987700
H -3.392305 -3.838592 -2.392363
O -3.122045 -1.654581 -0.780629
C -2.639747 3.887676 1.118540
C -3.152604 4.004390 -1.325445
C -4.364177 2.348593 0.132177
H -3.843534 4.822781 -1.087112
H -2.180270 4.446569 -1.581786
H -3.566484 3.496996 -2.209819
H -5.146936 3.084085 0.360343
H -4.677414 1.775843 -0.751868
H -4.281105 1.658213 0.981985
H -3.389254 4.670525 1.292901
H -2.587671 3.252394 2.009884
H -1.658379 4.358467 0.977534
C -4.448645 -1.908384 -1.173910
H -5.060937 -1.124259 -0.720778
H -4.563440 -1.870412 -2.267978
H -4.795605 -2.887709 -0.812618

Reaction 8

reactant

42
Energy: -940.199831038
C -4.027195 0.068797 0.183428
O -4.596252 0.199417 -0.890942
C -4.813953 -0.078438 1.489907
N -2.667926 0.047528 0.254072
H -2.176663 -0.052475 1.130214
C -1.887944 0.185069 -0.965866
C -0.464234 0.106246 -0.682416
H -2.189765 -0.598125 -1.678378
H -2.136773 1.139713 -1.454766
C 0.719161 0.039288 -0.432666
C 2.113773 -0.037139 -0.144295
C 2.712533 -1.272665 0.146036
C 4.066093 -1.341249 0.427900
C 4.846085 -0.182805 0.423868
C 4.257028 1.050238 0.134131
C 2.902159 1.123281 -0.146566
H 2.099150 -2.171621 0.144737
H 4.548362 -2.289673 0.655441
C 6.290445 -0.324214 0.734115
H 4.868235 1.949233 0.131496
H 2.434411 2.079548 -0.372775
C -3.944250 -0.225686 2.734323
C -5.691505 1.167496 1.622609
C -5.700889 -1.315314 1.335399
H -4.584858 -0.324746 3.621021
H -3.312107 -1.124947 2.694056
H -3.303900 0.653477 2.898636
H -6.358968 -1.420066 2.209371
H -6.319977 -1.232983 0.434883
H -5.097436 -2.230539 1.253689
H -6.350210 1.074347 2.497418
H -5.081329 2.072635 1.752570
H -6.309706 1.298133 0.727239
O 6.938283 0.851105 0.697693
O 6.831879 -1.376345 0.992330
C 8.340689 0.790860 0.987362
C 8.878918 2.194056 0.895512
H 8.476896 0.355068 1.986005
H 8.820575 0.110964 0.270990
H 9.953878 2.202820 1.109846
H 8.725237 2.606544 -0.108653
H 8.379294 2.852136 1.616239

I_c

77
Energy: -4540.82468098
Cu 0.635263 -0.005433 -0.515173
Cl 1.249126 -0.625969 -2.516262
C 1.921590 1.381590 -0.753159
O -0.707377 -1.504170 -0.341779
S -1.543064 -1.873159 0.855926
O -2.203056 -0.726090 1.485482
C -2.912758 -2.776468 0.035725
O -0.915672 -2.855159 1.731267
F -3.774159 -3.199634 0.944134
F -3.556277 -1.961600 -0.809051
C 1.399040 2.640291 -0.528499
C 2.301931 3.704848 -0.432661
C 3.664586 3.496117 -0.590979
C 4.147062 2.215161 -0.848312
C 3.269674 1.136514 -0.939534
C -1.037706 3.016957 2.078953
O -0.146161 3.854559 1.989035
C -2.508593 3.436037 2.042772
N -0.766621 1.691257 2.255796
C 0.591796 1.252310 2.409779
C 1.034702 0.156924 1.526011
C 1.701133 -0.852738 1.266427
C 2.558232 -1.930512 0.919225
C 2.056437 -3.213520 0.642511
C 2.938983 -4.222065 0.296147
C 4.311720 -3.973302 0.230783
C 4.814114 -2.702184 0.521385
C 3.942852 -1.682002 0.861006
H 0.338062 2.823244 -0.382691
H 1.907333 4.693664 -0.205654
H 4.355779 4.333865 -0.519206

H 5.212579 2.045297 -0.996948
H 3.636240 0.139308 -1.174264
H -1.489859 0.979898 2.178579
H 1.227105 2.133158 2.229214
H 0.782635 0.913365 3.440900
H 0.986117 -3.394129 0.715328
H 2.582450 -5.224549 0.070948
C 5.198523 -5.106475 -0.153249
H 5.884851 -2.522655 0.475914
H 4.314175 -0.685164 1.093349
C -3.491515 2.282331 2.205998
C -2.701607 4.435938 3.186219
C -2.744991 4.138282 0.704603
O -3.319470 1.210452 -1.023375
C -4.557465 0.836860 -1.646602
C -5.607361 0.869466 -0.568172
C -2.135578 0.880595 -1.524259
O -1.151479 1.202614 -0.871951
C -2.079492 0.157657 -2.830607
H -4.771121 1.551046 -2.454793
H -4.472024 -0.166212 -2.079781
H -6.587413 0.605101 -0.981425
H -5.351570 0.151528 0.221233
H -5.673076 1.868621 -0.120757
H -2.802969 0.555321 -3.550503
H -1.067318 0.218656 -3.235058
H -2.304721 -0.903555 -2.659672
H -3.357913 1.759202 3.163725
H -4.517171 2.678750 2.194954
H -3.408762 1.550015 1.392282
H -3.776789 4.516578 0.657039
H -2.056267 4.983366 0.585462
H -2.597272 3.442836 -0.132049
H -3.726064 4.833731 3.164688
H -2.546601 3.956547 4.163395
H -1.996381 5.270115 3.097388
O 6.489260 -4.745573 -0.190844
O 4.796762 -6.221312 -0.397993
C 7.422527 -5.771679 -0.567941
H 8.375128 -5.445017 -0.137815
H 7.119794 -6.713447 -0.095593
C 7.499628 -5.906484 -2.069310
H 8.272261 -6.634587 -2.344281
H 6.543001 -6.253876 -2.474921
H 7.752122 -4.945774 -2.533613
F -2.447776 -3.807464 -0.646409

I_1

63
Energy: -4233.30302189
Cu -0.043125 0.148151 -0.005046
Cl -0.024543 0.300117 2.121434
C 1.818433 0.269054 0.312612
O -2.070233 0.302131 -0.287634
S -2.107240 1.690884 -0.883664
O -2.774390 1.800531 -2.166658
C -3.130525 2.622599 0.319758
O -0.745837 2.262394 -0.765170
F -3.190194 3.889892 -0.054697
F -4.350351 2.110860 0.345663
F -2.593298 2.543232 1.523324
C 2.615335 -0.853414 0.413972
C 3.993244 -0.654526 0.307524
C 4.515194 0.625769 0.138720
C 3.669422 1.728602 0.079860
C 2.287829 1.563371 0.192511
H 2.194219 -1.851154 0.543354
H 4.655830 -1.516948 0.363896
H 5.592463 0.765718 0.068469
H 4.075356 2.732240 -0.036433
H 1.612356 2.417465 0.179385
C -0.391235 -3.200437 0.663103
O 0.677164 -3.501720 0.142844
C -0.737389 -3.603214 2.094288
N -1.349964 -2.551348 -0.065001
C -1.087235 -2.223517 -1.446095
C -0.138600 -1.111005 -1.656604
C 0.573450 -0.288717 -2.230037
C 1.498443 0.585224 -2.865196
C 1.110118 1.871455 -3.271352
C 2.044004 2.704908 -3.863332

C 3.356102 2.267158 -4.059437
C 3.738983 0.981980 -3.667613
C 2.813953 0.141561 -3.071987
H -2.143581 -2.110483 0.382635
H -0.670987 -3.107920 -1.945501
H -2.036077 -1.958454 -1.926101
H 0.088473 2.201375 -3.094972
H 1.778030 3.709655 -4.183836
C 4.307956 3.218156 -4.696550
H 4.762327 0.652454 -3.826841
H 3.095800 -0.857197 -2.743835
C -1.965557 -2.891133 2.654746
C -0.998065 -5.113831 2.045842
C 0.479327 -3.321161 2.974785
H 0.293778 -3.688274 3.993511
H 1.368460 -3.825259 2.578567
H 0.686666 -2.243615 3.032051
H -1.213179 -5.486324 3.056949
H -1.860861 -5.349640 1.406418
H -0.122525 -5.647665 1.656240
H -2.115163 -3.193349 3.699796
H -1.848445 -1.797860 2.643534
H -2.884355 -3.161519 2.113688
O 5.534813 2.695764 -4.832140
O 4.011021 4.336094 -5.051286
C 6.507112 3.556669 -5.443542
C 7.805682 2.797529 -5.502448
H 6.144175 3.845039 -6.438721
H 6.585301 4.476416 -4.849141
H 8.584294 3.415471 -5.964187
H 8.141218 2.517346 -4.496961
H 7.697183 1.881930 -6.095631

TS_{rc}^{A}

63
Energy: -4233.28152947
Cu -0.770305 0.820789 -0.833777
Cl -1.645707 1.458808 -2.673458
C 0.231533 2.407635 -0.913915
O -1.415426 -1.040079 -0.949989
S -1.977325 -2.073857 -0.012138
O -3.398049 -1.877671 0.279593
C -1.883762 -3.560738 -1.080046
O -1.118861 -2.378810 1.134417
F -2.349560 -4.602718 -0.407900
F -2.601313 -3.389872 -2.174959
F -0.619723 -3.787614 -1.411769
C -0.155852 3.529758 -0.204183
C 0.697491 4.633191 -0.204207
C 1.893633 4.600706 -0.915635
C 2.243703 3.465225 -1.638938
C 1.403264 2.352428 -1.649917
H -1.091984 3.546091 0.349974
H 0.418244 5.523013 0.358563
H 2.550353 5.468923 -0.914793
H 3.170199 3.439578 -2.210539
H 1.666847 1.467286 -2.228682
C -3.251415 0.889096 1.457826
O -2.325946 1.521053 0.906704
C -4.690634 1.028960 1.017179
N -2.914174 0.168057 2.543127
C -1.507830 0.213957 2.908588
C -0.687539 0.532033 1.730974
C 0.190665 0.422445 0.837058
C 1.566967 -0.045470 0.710862
C 1.853887 -1.348372 0.289855
C 3.176564 -1.745489 0.165798
C 4.216368 -0.858927 0.450498
C 3.924152 0.443361 0.861478
C 2.605474 0.851959 0.985884
H -3.494123 -0.606339 2.842654
H -1.352248 0.951507 3.709423
H -1.189500 -0.778078 3.256561
H 1.040087 -2.040287 0.085311
H 3.427803 -2.755119 -0.152446
C 5.606758 -1.354081 0.297744
H 4.734524 1.134326 1.079770
H 2.365914 1.870999 1.287948
C -4.756407 0.968943 -0.508835
C -5.598045 -0.028656 1.638094
C -5.119008 2.424268 1.501062

H -6.161255 2.599842 1.203856
H -5.058804 2.509484 2.595492
H -4.496843 3.207985 1.051865
H -6.622372 0.127128 1.276358
H -5.289716 -1.040258 1.346646
H -5.634027 0.049294 2.735155
H -5.792937 1.143388 -0.827070
H -4.118770 1.732306 -0.971140
H -4.436033 -0.012150 -0.879071
O 6.519756 -0.414383 0.595027
O 5.896550 -2.475844 -0.053715
C 7.886906 -0.825134 0.468952
C 8.746491 0.352841 0.844052
H 8.057346 -1.691960 1.121114
H 8.062290 -1.159123 -0.562274
H 9.807471 0.089364 0.763477
H 8.552693 1.204664 0.181425
H 8.548115 0.668904 1.875046

TS_{ar}^{B}

63
Energy: -4233.29192990
Cu 1.033411 0.798914 0.472252
Cl 1.633573 1.154516 2.522600
C -0.388700 2.120123 0.591042
O 2.288284 -0.725348 0.306693
S 1.908856 -2.144944 -0.044348
O 2.373388 -2.515266 -1.385257
C 2.989711 -3.058377 1.122095
O 0.539535 -2.488316 0.321391
F 2.820494 -4.358800 0.944591
F 4.259148 -2.748174 0.887778
F 2.682298 -2.737030 2.363417
C -0.197061 3.268450 -0.166190
C -0.867516 4.431610 0.209273
C -1.726843 4.422299 1.302366
C -1.918406 3.251572 2.035241
C -1.246907 2.088033 1.685137
H 0.473810 3.261693 -1.022775
H -0.718007 5.343016 -0.366761
H -2.255812 5.330740 1.584512
H -2.583686 3.244135 2.896524
H -1.385979 1.171733 2.255346
C 3.218951 1.066796 -1.426267
O 2.526455 1.944188 -0.891495
C 4.710395 0.955071 -1.164740
N 2.654245 0.220604 -2.328124
C 1.231883 0.309727 -2.534039
C 0.373505 0.397235 -1.327422
C -0.720609 0.533087 -0.719374
C -2.100054 0.140745 -0.514849
C -2.375609 -1.198779 -0.216413
C -3.696600 -1.601165 -0.077303
C -4.734125 -0.683473 -0.238145
C -4.451583 0.654291 -0.533361
C -3.137476 1.069997 -0.660751
H 3.039263 -0.720301 -2.389734
H 1.005225 1.205651 -3.131436
H 0.917335 -0.569081 -3.112468
H -1.549660 -1.898180 -0.090635
H -3.945681 -2.633688 0.157589
C -6.126528 -1.185097 -0.081377
H -5.267777 1.361151 -0.656688
H -2.901235 2.111495 -0.875429
C 5.314733 -0.344419 -1.689212
C 5.341990 2.147951 -1.896430
C 4.960408 1.079968 0.338431
H 6.385864 -0.363099 -1.449175
H 4.860453 -1.229777 -1.222651
H 5.227623 -0.432456 -2.781560
H 6.042914 1.115566 0.522306
H 4.506404 1.991681 0.743073
H 4.540676 0.225435 0.883514
O -7.035238 -0.211753 -0.243728
O -6.403091 -2.336647 0.167870
C -8.411686 -0.605007 -0.116068
H -8.935623 0.318642 0.151547
C -8.929065 -1.191381 -1.407118
H -8.498265 -1.319074 0.711124
H -9.999540 -1.412961 -1.319633
H -8.790809 -0.487931 -2.236861

H -8.405488 -2.124346 -1.643470
H 6.427708 2.145576 -1.730061
H 5.164041 2.093699 -2.980079
H 4.936199 3.095912 -1.522200

I_2

63
Energy: -4233.34601313
Cu 0.267670 -1.424793 -1.978953
Cl 0.971565 -3.464567 -2.432463
C 2.031294 0.460139 -0.749089
O -0.095209 -1.719693 -0.159120
S -1.610901 -1.709902 0.065475
O -2.316978 -1.951008 -1.196861
C -1.807186 -3.233389 1.071630
O -2.056940 -0.598581 0.892035
F -3.091817 -3.373981 1.362589
F -1.391112 -4.280060 0.390831
F -1.113110 -3.117534 2.189392
C 2.669247 -0.375347 -1.675866
C 3.795010 -1.107720 -1.307328
C 4.302651 -0.996821 -0.020177
C 3.692195 -0.140841 0.896923
C 2.565644 0.584380 0.537453
H 2.333898 -0.394762 -2.714342
H 4.264338 -1.768840 -2.032822
H 5.178058 -1.573244 0.272647
H 4.091589 -0.050903 1.905594
H 2.068960 1.228411 1.262285
C -1.151220 -0.731276 -4.150806
O 0.015209 -1.055512 -3.796651
C -1.736043 -1.440943 -5.356388
N -1.891076 0.141792 -3.476880
C -1.519300 0.793966 -2.223165
C -0.178406 0.404929 -1.728559
C 0.757533 1.124069 -1.103318
C 0.551633 2.536713 -0.710715
C -0.618871 2.929940 -0.051013
C -0.803077 4.256621 0.311551
C 0.178892 5.205571 0.025846
C 1.358677 4.814283 -0.613325
C 1.545283 3.488257 -0.971147
H -2.847843 0.272059 -3.780837
H -1.519693 1.883790 -2.389195
H -2.306479 0.562542 -1.490468
H -1.359532 2.176598 0.219270
H -1.701887 4.579799 0.832837
C -0.079050 6.609826 0.434848
H 2.125339 5.555471 -0.825006
H 2.465364 3.176124 -1.464429
C -2.940350 -0.720922 -5.955152
C -0.645848 -1.609893 -6.414115
C -2.154815 -2.819941 -4.816162
H -2.572282 -3.415254 -5.639446
H -1.295695 -3.356350 -4.391303
H -2.917951 -2.728265 -4.030626
H -1.046399 -2.194985 -7.251954
H -0.308813 -0.640810 -6.806706
H 0.220052 -2.139753 -6.003018
H -3.262962 -1.256240 -6.856846
H -3.809409 -0.711366 -5.280192
H -2.703799 0.310720 -6.251172
O 0.945221 7.424129 0.136971
O -1.095705 6.985061 0.974832
C 0.768258 8.798139 0.506736
C 2.016879 9.536233 0.102990
H -0.128177 9.186051 0.005105
H 0.581474 8.851339 1.587358
H 1.931734 10.597094 0.365309
H 2.895846 9.125484 0.613600
H 2.182924 9.461864 -0.978234

TS_{rc}^{B}

63
Energy: -4233.31821338
Cu -2.320823 0.106246 -0.253607
Cl -3.756306 -0.543105 -1.791442
C -0.125249 -0.282377 2.364161
O -2.012827 1.833224 0.480659
S -1.042382 2.689792 -0.322013

O -0.515379 1.967948 -1.482405
C -2.190374 3.937900 -1.018312
O -0.108926 3.414139 0.525799
F -1.510852 4.788908 -1.771188
F -3.104254 3.331475 -1.759347
F -2.787077 4.598331 -0.039844
C -1.470646 -0.185047 2.750406
C -1.822706 -0.098168 4.086100
C -0.838430 -0.104389 5.071679
C 0.500424 -0.196400 4.706455
C 0.855371 -0.282950 3.367376
H -2.245660 -0.152981 1.990467
H -2.872518 -0.010866 4.358427
H -1.116065 -0.028844 6.121435
H 1.277223 -0.196912 5.468761
H 1.906140 -0.356841 3.093582
C -1.828467 -2.716630 -0.681726
O -1.964977 -1.958506 0.316005
C -2.593925 -4.017504 -0.765122
N -0.914666 -2.391910 -1.596054
C -0.295874 -1.076538 -1.479086
C -0.540303 -0.605813 -0.084605
C 0.275876 -0.416689 0.961121
C 1.720945 -0.263048 0.613340
C 2.306183 1.004751 0.575337
C 3.658556 1.123429 0.285123
C 4.431405 -0.011137 0.031757
C 3.843190 -1.277578 0.064482
C 2.491134 -1.400844 0.352615
H -0.916130 -2.859250 -2.493987
H 0.787547 -1.149991 -1.642405
H -0.711942 -0.390186 -2.230384
H 1.694417 1.889053 0.761639
H 4.138431 2.098993 0.245258
C 5.873817 0.186938 -0.265646
H 4.450606 -2.158258 -0.129017
H 2.021893 -2.385328 0.392277
C -3.966606 -3.825126 -0.122129
C -2.756919 -4.471906 -2.214008
C -1.777209 -5.046421 0.029582
H -3.383971 -5.371798 -2.238542
H -3.252180 -3.701622 -2.820932
H -1.799841 -4.747909 -2.681641
H -2.307112 -6.007979 0.024652
H -0.782583 -5.202129 -0.410815
H -1.651678 -4.729803 1.072879
H -4.522907 -4.769919 -0.175484
H -3.874625 -3.536300 0.930961
H -4.536949 -3.044272 -0.641120
O 6.506003 -0.974971 -0.501833
O 6.418354 1.267704 -0.286670
C 7.907543 -0.880455 -0.800180
H 8.311085 -1.863219 -0.533701
C 8.131355 -0.557866 -2.257778
H 8.356537 -0.120808 -0.149695
H 9.203703 -0.566772 -2.487567
H 7.636475 -1.296207 -2.900059
H 7.739065 0.436955 -2.495966

Reaction 9

reactant

42
Energy: -940.199439061
C -3.995651 0.044487 0.175959
O -4.579459 0.012655 -0.898072
C -4.762844 -0.004998 1.501424
N -2.637296 0.111363 0.230346
H -2.136469 0.157887 1.105575
C -1.872096 0.149920 -1.006288
C -0.444779 0.092064 -0.736152
H -2.186941 -0.689413 -1.644564
H -2.124455 1.060765 -1.571866
C 0.741749 0.046459 -0.497540
C 2.140977 -0.015432 -0.222112
C 2.773099 -1.254061 -0.037455
C 4.134221 -1.317296 0.231761
C 4.881080 -0.149720 0.319760
C 4.263866 1.089383 0.137428
C 2.899259 1.157465 -0.132179
H 2.179020 -2.163451 -0.110012

H 4.614264 -2.283689 0.372727
H 5.948593 -0.170390 0.529064
C 5.113664 2.303463 0.242819
H 2.418007 2.121824 -0.274876
C -3.881697 0.093821 2.742571
C -5.758581 1.155727 1.486186
C -5.523996 -1.332349 1.518418
H -4.509768 0.059220 3.643038
H -3.172423 -0.743758 2.815671
H -3.321417 1.039679 2.778216
H -6.164329 -1.389714 2.409858
H -6.152860 -1.425093 0.625582
H -4.832979 -2.187100 1.541419
H -6.406443 1.109604 2.372774
H -5.240709 2.125315 1.495043
H -6.384657 1.111851 0.587967
O 4.416888 3.433677 0.049672
O 6.301505 2.283058 0.476698
C 5.174450 4.647731 0.135221
C 4.222753 5.788633 -0.108885
H 5.646261 4.700519 1.125329
H 5.983625 4.611957 -0.606115
H 4.755376 6.745128 -0.054217
H 3.762081 5.707678 -1.100464
H 3.422581 5.797254 0.640588

I_c

77
Energy: -4540.82292984
Cu 0.627171 0.092573 -0.477054
Cl 1.283202 -0.549689 -2.461114
C 1.921521 1.473671 -0.690120
O -0.725786 -1.388866 -0.305181
S -1.563594 -1.766525 0.889927
O -2.275273 -0.631706 1.487283
C -2.882647 -2.730974 0.057814
O -0.913046 -2.703096 1.795925
F -3.752243 -3.163899 0.954230
F -3.531208 -1.948047 -0.814925
C 1.397004 2.735101 -0.486196
C 2.299083 3.799522 -0.382885
C 3.664325 3.587824 -0.512710
C 4.150032 2.303882 -0.748266
C 3.273034 1.225565 -0.846081
C -1.076711 3.106459 2.078139
O -0.181071 3.942118 2.015191
C -2.545223 3.529089 2.003448
N -0.813075 1.778765 2.251836
C 0.539526 1.336137 2.444524
C 1.002594 0.245955 1.564916
C 1.663330 -0.770136 1.314656
C 2.516365 -1.849337 0.964135
C 2.013945 -3.115324 0.628736
C 2.899231 -4.122802 0.261729
C 4.275858 -3.882172 0.245438
C 4.776604 -2.632821 0.596502
C 3.904832 -1.614152 0.947513
H 0.333190 2.919419 -0.364751
H 1.901897 4.791284 -0.173866
H 4.355087 4.425467 -0.435517
H 5.218102 2.131736 -0.874574
H 3.642663 0.225664 -1.064834
H -1.538774 1.070022 2.168368
H 1.180910 2.216951 2.286681
H 0.698816 0.991385 3.478976
H 0.943623 -3.314260 0.659010
C 2.322793 -5.444572 -0.109806
H 4.950507 -4.684386 -0.043187
H 5.849421 -2.453237 0.589758
H 4.277317 -0.627573 1.219794
C -3.534341 2.380124 2.161765
C -2.761923 4.547577 3.125784
C -2.749593 4.210287 0.649001
O -3.313393 1.257265 -1.057605
C -4.543317 0.832445 -1.664182
C -5.583323 0.848323 -0.575774
C -2.122074 0.940877 -1.548890
O -1.145166 1.303134 -0.906257
C -2.050181 0.186025 -2.836218
H -4.786587 1.526081 -2.481858
H -4.429649 -0.174465 -2.081506

H -6.555490 0.532077 -0.970823
H -5.286621 0.162666 0.227848
H -5.686729 1.854660 -0.152274
H -2.776129 0.557122 -3.567968
H -1.037116 0.250571 -3.237741
H -2.262866 -0.873292 -2.639808
H -3.421976 1.869822 3.129132
H -4.558392 2.778902 2.123370
H -3.436323 1.635996 1.360529
H -3.778487 4.591892 0.573271
H -2.054829 5.050793 0.531187
H -2.587258 3.500662 -0.172798
H -3.784227 4.948015 3.073361
H -2.631958 4.083656 4.114009
H -2.051883 5.377990 3.040655
O 3.276202 -6.326382 -0.452438
O 1.139701 -5.690140 -0.108556
C 2.816572 -7.633391 -0.832656
H 3.617902 -8.032047 -1.463904
C 2.574409 -8.496750 0.381583
H 1.906703 -7.524633 -1.434303
H 2.299998 -9.513242 0.074533
H 3.476502 -8.557261 1.002192
H 1.756196 -8.089635 0.985869
F -2.369991 -3.755869 -0.594371

I_1

63
Energy: -4233.30134818
Cu -0.057677 0.129780 0.076450
Cl -0.025948 0.258015 2.205185
C 1.809508 0.221858 0.359482
O -2.077359 0.294826 -0.166974
S -2.120845 1.677146 -0.782928
O -2.814391 1.764651 -2.052664
C -3.118779 2.625389 0.429010
O -0.755488 2.243063 -0.694496
F -3.192794 3.885986 0.036799
F -4.335631 2.109227 0.492667
F -2.552418 2.566548 1.620738
C 2.593196 -0.912263 0.435788
C 3.974983 -0.728643 0.351312
C 4.515412 0.548943 0.226110
C 3.683548 1.663352 0.186517
C 2.298705 1.512272 0.276234
H 2.159593 -1.908471 0.533186
H 4.625957 -1.600819 0.391955
H 5.595302 0.677110 0.175032
H 4.103533 2.664638 0.104539
H 1.634879 2.375762 0.278172
C -0.428017 -3.227331 0.657888
O 0.631483 -3.531599 0.120957
C -0.762264 -3.650701 2.085940
N -1.386768 -2.553845 -0.047200
C -1.128921 -2.191599 -1.420413
C -0.177830 -1.075496 -1.606288
C 0.528931 -0.249675 -2.182855
C 1.451664 0.627485 -2.813150
C 1.058768 1.898953 -3.248955
C 1.997040 2.735150 -3.846578
C 3.317835 2.306618 -4.006462
C 3.704722 1.039902 -3.581816
C 2.777551 0.197725 -2.988251
H -2.167283 -2.108129 0.418656
H -0.717662 -3.064562 -1.943192
H -2.078544 -1.911107 -1.890163
H 0.035122 2.243941 -3.109727
C 1.536236 4.074036 -4.304761
H 4.038495 2.975584 -4.470671
H 4.734147 0.711812 -3.709249
H 3.063954 -0.790049 -2.630753
C -1.977267 -2.936504 2.671882
C -1.036788 -5.157935 2.016335
C 0.466959 -3.392816 2.956637
H 0.289437 -3.773281 3.971917
H 1.346857 -3.899085 2.542871
H 0.684774 -2.317997 3.027494
H -1.242396 -5.545261 3.023861
H -1.909633 -5.375695 1.384161
H -0.170994 -5.692756 1.606766
H -2.119571 -3.256879 3.712566

H -1.848543 -1.844363 2.680181
H -2.904078 -3.187181 2.135064
O 2.539760 4.807612 -4.810830
O 0.392782 4.457708 -4.234181
C 2.168570 6.111655 -5.281579
C 3.416417 6.773702 -5.802005
H 1.398938 5.999731 -6.056496
H 1.712929 6.668205 -4.452286
H 3.184257 7.777769 -6.175254
H 4.169019 6.867481 -5.010190
H 3.853285 6.194492 -6.624106

TS_{rc}^{A}

63
Energy: -4233.27969541
Cu 0.884870 0.874979 0.772056
Cl 1.667220 1.119915 2.745685
C 0.631872 2.736149 0.771689
O 0.740011 -1.085745 0.875675
S 0.786754 -2.276156 -0.045375
O 2.136425 -2.822488 -0.199178
C -0.142203 -3.496937 0.958616
O 0.009276 -2.122676 -1.275967
F -0.202160 -4.641586 0.294012
F 0.465559 -3.695616 2.115504
F -1.368194 -3.049678 1.173249
C 1.555396 3.576615 0.176432
C 1.253942 4.936606 0.100737
C 0.061102 5.425508 0.625663
C -0.837732 4.558114 1.237915
C -0.552831 3.195697 1.322204
H 2.484027 3.185599 -0.234669
H 1.962391 5.614114 -0.374280
H -0.163666 6.489041 0.566209
H -1.765425 4.936767 1.664464
H -1.249984 2.515480 1.811900
C 3.401200 -0.204022 -1.170431
O 2.803209 0.804791 -0.738952
C 4.658823 -0.732014 -0.517888
N 2.924179 -0.732455 -2.312780
C 1.765402 -0.051581 -2.868891
C 1.022176 0.624946 -1.796873
C 0.075682 0.900296 -1.012876
C -1.370359 1.091387 -1.084801
C -2.260760 0.117575 -0.635075
C -3.632644 0.363277 -0.689886
C -4.115668 1.577010 -1.181647
C -3.222641 2.549078 -1.615364
C -1.854654 2.314392 -1.563549
H 3.100449 -1.705265 -2.535236
H 2.086284 0.659971 -3.643354
H 1.084673 -0.792688 -3.308577
H -1.905892 -0.840202 -0.258680
C -4.538907 -0.710643 -0.202982
H -5.187764 1.752542 -1.215861
H -3.592068 3.500700 -1.992620
H -1.145946 3.081895 -1.874640
C 4.433751 -0.838175 0.991092
C 5.093731 -2.077690 -1.090405
C 5.735049 0.325583 -0.811168
H 6.680714 0.007769 -0.352778
H 5.904020 0.442288 -1.891302
H 5.458782 1.299983 -0.390598
H 6.013353 -2.396636 -0.583478
H 4.332764 -2.849234 -0.918165
H 5.326325 -2.014450 -2.163939
H 5.371511 -1.154671 1.467188
H 4.133026 0.123537 1.424577
H 3.656109 -1.576220 1.222470
O -5.833981 -0.371076 -0.315661
O -4.164006 -1.769271 0.244897
C -6.767417 -1.360123 0.137408
C -8.150175 -0.801424 -0.069826
H -6.601646 -2.287080 -0.427269
H -6.560554 -1.584545 1.192127
H -8.904696 -1.523869 0.262392
H -8.287058 0.125000 0.500477
H -8.328137 -0.581211 -1.129116

TS_{ar}^{B}

```
  63
Energy: -4233.28968422
 Cu 1.003672 0.835580 0.470628
 Cl 1.505892 0.952270 2.575619
 C 0.051849 2.527551 0.545810
 O 1.750659 -0.982038 0.334590
 S 0.987588 -2.206849 -0.120167
 O 1.501345 -2.717675 -1.395194
 C 1.543673 -3.407366 1.149640
 O -0.455486 -2.078530 0.035956
 F 1.022569 -4.594960 0.887201
 F 2.867676 -3.496732 1.123693
 F 1.154379 -3.006775 2.344489
 C 0.654231 3.585609 -0.122311
 C 0.338352 4.886379 0.268333
 C -0.580288 5.107202 1.288514
 C -1.187711 4.029140 1.931830
 C -0.873611 2.727650 1.565312
 H 1.367398 3.399389 -0.922547
 H 0.811920 5.725492 -0.238160
 H -0.829629 6.124829 1.583504
 H -1.899848 4.199359 2.737085
 H -1.337355 1.880881 2.067409
 C 3.335080 0.472022 -1.234178
 O 2.891256 1.514204 -0.732464
 C 4.691901 -0.087845 -0.843639
 N 2.628928 -0.160255 -2.208237
 C 1.317717 0.343213 -2.527647
 C 0.429264 0.700864 -1.393664
 C -0.624940 1.168342 -0.888856
 C -2.069793 1.255173 -0.846682
 C -2.814845 0.081267 -0.715962
 C -4.208413 0.153599 -0.733199
 C -4.845285 1.385820 -0.882431
 C -4.095961 2.551606 -1.014921
 C -2.711693 2.492978 -0.987395
 H 2.727297 -1.171867 -2.272528
 H 1.414145 1.256423 -3.133808
 H 0.805102 -0.413531 -3.136423
 H -2.317654 -0.881686 -0.590696
 C -4.967719 -1.119143 -0.594443
 H -5.931666 1.424595 -0.893577
 H -4.596310 3.510098 -1.135281
 H -2.113261 3.398899 -1.077141
 C 4.931394 -1.501699 -1.365685
 C 5.720954 0.869490 -1.461084
 C 4.815203 -0.065838 0.680446
 H 6.734108 0.538185 -1.195976
 H 5.645089 0.886722 -2.557872
 H 5.580492 1.890257 -1.084777
 H 5.835316 -0.359276 0.963779
 H 4.614016 0.933860 1.082103
 H 4.107854 -0.763053 1.146559
 H 5.919467 -1.843773 -1.030917
 H 4.190573 -2.216494 -0.980404
 H 4.930331 -1.545342 -2.464229
 O -6.294393 -0.911006 -0.572370
 O -4.450184 -2.208520 -0.517719
 C -7.112689 -2.084151 -0.432498
 H -8.080586 -1.793696 -0.854771
 H -6.682482 -2.888115 -1.041052
 C -7.230465 -2.492565 1.015820
 H -7.920365 -3.339252 1.115163
 H -6.255242 -2.798846 1.409955
 H -7.613663 -1.664195 1.623701
```

I_2

```
  63
Energy: -4233.34536002
 Cu 0.325631 -1.474114 -2.013892
 Cl 1.049818 -3.472362 -2.606503
 C 2.079579 0.324287 -0.687306
 O -0.009494 -1.890927 -0.213952
 S -1.522497 -1.906079 0.032573
 O -2.244426 -2.065119 -1.234101
 C -1.696799 -3.499013 0.928768
 O -1.960831 -0.859301 0.941167
 F -2.974902 -3.662808 1.236436
 F -1.296296 -4.493501 0.164700
 F -0.978942 -3.465064 2.037348
 C 2.720982 -0.437192 -1.673005
```

C 3.840707 -1.202760 -1.355155
C 4.337041 -1.199221 -0.059217
C 3.721502 -0.417951 0.919513
C 2.601945 0.339939 0.610079
H 2.390381 -0.374631 -2.711343
H 4.312434 -1.807013 -2.127145
H 5.206613 -1.802778 0.193599
H 4.110900 -0.413970 1.936102
H 2.099779 0.924056 1.380423
C -1.138586 -0.672802 -4.114615
O 0.040341 -0.996440 -3.803080
C -1.737870 -1.320475 -5.347792
N -1.876067 0.148985 -3.377140
C -1.484099 0.737491 -2.098887
C -0.133076 0.334488 -1.646949
C 0.809925 1.021576 -0.996215
C 0.636976 2.421492 -0.547703
C -0.557391 2.858719 0.025869
C -0.704942 4.185893 0.430902
C 0.351555 5.084214 0.279643
C 1.551073 4.650222 -0.274692
C 1.695744 3.330342 -0.680718
H -2.842709 0.278008 -3.648684
H -1.495605 1.834741 -2.209120
H -2.255338 0.464799 -1.362608
H -1.376768 2.163896 0.210783
C -2.012471 4.583951 1.014369
H 0.228994 6.115191 0.601857
H 2.379472 5.346447 -0.390980
H 2.636518 2.989041 -1.112037
C -2.971765 -0.592369 -5.872033
C -0.670685 -1.396684 -6.439311
C -2.113854 -2.740038 -4.887740
H -2.538295 -3.290985 -5.737947
H -1.233680 -3.282708 -4.517541
H -2.859741 -2.716024 -4.080988
H -1.078828 -1.937154 -7.303089
H -0.365004 -0.397043 -6.777294
H 0.216614 -1.930838 -6.083131
H -3.305055 -1.077705 -6.797826
H -3.823543 -0.644686 -5.177397
H -2.764730 0.460891 -6.108158
O -2.000558 5.847708 1.466989
O -2.979085 3.856445 1.064660
C -3.222522 6.319594 2.056223
H -3.169997 7.409268 1.958848
H -4.067070 5.947094 1.464663
C -3.328820 5.888941 3.499014
H -4.225651 6.321859 3.958470
H -3.400594 4.798083 3.570667
H -2.454494 6.223862 4.069749

TS_{rc}^{B}

63
Energy: -4233.31478328
Cu -1.913751 1.026209 0.043851
Cl -3.559979 1.722925 -1.241044
C -0.225214 -1.209284 2.042445
O -0.589133 2.013710 0.978981
S 0.669931 2.298665 0.169120
O 0.578386 1.745277 -1.184682
C 0.519654 4.111876 -0.054309
O 1.884944 2.069657 0.931853
F 1.536260 4.550904 -0.780298
F -0.614770 4.386755 -0.679876
F 0.528352 4.715903 1.122327
C -1.217967 -0.472474 2.705959
C -1.369516 -0.556521 4.079077
C -0.530641 -1.379073 4.827048
C 0.460492 -2.114978 4.186301
C 0.613684 -2.031424 2.809224
H -1.862236 0.191892 2.138253
H -2.139074 0.035737 4.569890
H -0.645132 -1.438514 5.907860
H 1.124440 -2.757083 4.762024
H 1.391277 -2.613844 2.318959
C -3.219117 -1.356481 -0.976114
O -2.817785 -0.955238 0.148842
C -4.608596 -1.930456 -1.134470
N -2.348784 -1.363699 -1.986375
C -1.069039 -0.701994 -1.761718

```
 C -0.898718 -0.579681 -0.284396
 C -0.066596 -1.168944 0.585934
 C 1.156051 -1.777284 -0.021307
 C 2.395656 -1.148130 0.087260
 C 3.524366 -1.750830 -0.470080
 C 3.415764 -2.972522 -1.137211
 C 2.179874 -3.599612 -1.240372
 C 1.052941 -3.004155 -0.684441
 H -2.683335 -1.482519 -2.934321
 H -0.248733 -1.310852 -2.163811
 H -1.048277 0.274295 -2.266847
 H 2.493278 -0.181105 0.587080
 C 4.827797 -1.047450 -0.319468
 H 4.304380 -3.425274 -1.569612
 H 2.094485 -4.558199 -1.748869
 H 0.078832 -3.492371 -0.752420
 C -5.558557 -1.195460 -0.190083
 C -5.101402 -1.798354 -2.573899
 C -4.507974 -3.409912 -0.736735
 H -6.139351 -2.149249 -2.630502
 H -5.082484 -0.751861 -2.907915
 H -4.525588 -2.418165 -3.277554
 H -5.502539 -3.869847 -0.805581
 H -3.828919 -3.962810 -1.400574
 H -4.151498 -3.517399 0.295697
 H -6.566063 -1.618514 -0.294680
 H -5.243595 -1.304415 0.853836
 H -5.594432 -0.124173 -0.425338
 O 5.813045 -1.668727 -0.991708
 O 4.983687 -0.044231 0.335280
 C 7.109474 -1.056456 -0.909836
 H 7.810982 -1.870910 -1.120815
 C 7.239553 0.070448 -1.905793
 H 7.268801 -0.702594 0.115523
 H 8.257834 0.477471 -1.886638
 H 7.029613 -0.283120 -2.922409
 H 6.542815 0.880141 -1.662472
```

Reaction 10

reactant

```
  42
Energy: -940.200705618
 C -3.631836 -0.435512 0.447648
 O -4.375524 -1.384207 0.230221
 C -4.162505 0.864655 1.063364
 N -2.311816 -0.490814 0.139371
 H -1.666063 0.274321 0.307983
 C -1.762403 -1.696689 -0.459012
 C -0.341140 -1.476450 -0.667158
 H -1.950572 -2.562750 0.194820
 H -2.283081 -1.922608 -1.402902
 C 0.776015 -1.013292 -0.715769
 C 2.084357 -0.462344 -0.767047
 C 3.125453 -1.240680 -1.292502
 C 4.419416 -0.749223 -1.362549
 C 4.703637 0.535679 -0.906916
 C 3.685987 1.319116 -0.383467
 C 2.373773 0.843531 -0.304055
 H 2.892657 -2.243451 -1.645196
 H 5.211131 -1.372180 -1.775052
 H 5.717623 0.926731 -0.959497
 H 3.899551 2.322441 -0.025676
 C 1.304260 1.708176 0.260634
 C -3.089468 1.914544 1.334886
 C -5.192262 1.425364 0.080243
 C -4.858141 0.490823 2.372643
 H -3.555074 2.803625 1.783091
 H -2.324293 1.553183 2.036995
 H -2.579957 2.238741 0.416126
 H -5.347804 1.374117 2.806928
 H -5.613907 -0.283219 2.197946
 H -4.138410 0.106000 3.109077
 H -5.674525 2.317910 0.503912
 H -4.718199 1.714022 -0.868826
 H -5.963175 0.676617 -0.136105
 O 1.779593 2.897123 0.664409
 O 0.130422 1.417438 0.364037
 C 0.811152 3.794866 1.222636
 C 1.541379 5.049121 1.623248
 H 0.032354 3.986666 0.472221
```

H 0.323698 3.304475 2.076077
H 0.841262 5.771712 2.058089
H 2.315469 4.829683 2.367913
H 2.022237 5.515931 0.755591

I_c

77
Energy: -4540.82102895
Cu 0.314687 -0.522078 -0.447236
Cl 0.398659 -1.319047 1.620137
C -1.427516 0.200874 -0.082722
O 2.189395 -1.172955 -0.779643
S 3.211901 -0.811899 -1.815478
O 3.879989 0.468304 -1.553286
C 4.516030 -2.057170 -1.472581
O 2.787940 -1.042266 -3.197786
F 5.571809 -1.791181 -2.227279
F 4.871130 -1.974395 -0.191680
C -1.622119 1.378042 -0.778530
C -2.885067 1.970287 -0.708156
C -3.897290 1.390301 0.046148
C -3.655703 0.208602 0.737896
C -2.404705 -0.409814 0.678013
C 0.300665 2.199282 -3.691786
O -0.901054 2.446310 -3.677867
C 1.328414 3.314249 -3.886275
N 0.771094 0.924438 -3.566156
C -0.127200 -0.194468 -3.605905
C -0.318174 -0.987018 -2.367203
C -0.743076 -1.992479 -1.781535
C -1.457989 -3.022120 -1.109255
C -0.826337 -4.037657 -0.364196
C -1.601175 -4.933308 0.361570
C -2.990813 -4.864195 0.314848
C -3.621800 -3.880045 -0.440694
C -2.859940 -2.950110 -1.133022
H -0.856774 1.846540 -1.389654
H -3.055803 2.880510 -1.281001
H -4.878570 1.859279 0.094833
H -4.435882 -0.247087 1.346599
H -2.214858 -1.326983 1.229547
H 1.761797 0.706255 -3.601073
H -1.111430 0.203877 -3.891197
H 0.204941 -0.906827 -4.374295
C 0.660159 -4.141964 -0.387743
H -1.104210 -5.692849 0.960229
H -3.584473 -5.582500 0.877239
H -4.707797 -3.823942 -0.474173
H -3.333434 -2.141315 -1.687120
C 2.778363 2.858493 -3.761218
C 1.085974 3.875306 -5.291189
C 1.027123 4.392363 -2.844036
O 2.757477 2.935530 -0.294675
C 4.100810 3.334996 0.012970
C 4.328196 4.659023 -0.667753
C 2.257431 1.790873 0.154306
O 1.206510 1.420700 -0.359322
C 2.951617 1.102049 1.282809
H 4.224693 3.418913 1.101986
H 4.781391 2.559830 -0.364446
H 5.340937 5.022518 -0.459257
H 4.211997 4.556787 -1.753243
H 3.608721 5.407045 -0.313812
H 3.083348 1.797341 2.121717
H 2.365677 0.240979 1.611462
H 3.940812 0.759929 0.957450
H 3.053713 2.119425 -4.528598
H 3.440958 3.724042 -3.908445
H 2.998993 2.428873 -2.774264
H 1.689412 5.257024 -2.998183
H -0.012803 4.728593 -2.928490
H 1.193823 4.010958 -1.827380
H 1.749997 4.732711 -5.470175
H 1.294476 3.120473 -6.063167
H 0.046640 4.205797 -5.404031
O 1.142399 -4.572537 0.777175
O 1.324284 -3.869099 -1.358815
C 2.573433 -4.498989 0.912180
F 4.097359 -3.281136 -1.726214
C 2.899825 -4.873040 2.332216
H 3.035674 -5.168964 0.175042

H 2.880405 -3.472441 0.672278
H 3.982598 -4.824132 2.496025
H 2.412287 -4.180722 3.028923
H 2.562175 -5.890995 2.561968

I_1

63
Energy: -4233.30141687
Cu 0.235709 0.131392 0.306714
Cl 0.266090 -0.042432 2.437438
C 2.094346 -0.055437 0.597665
O -1.747379 0.605832 0.140085
S -1.631978 2.072111 -0.205750
O -2.511512 2.541272 -1.259546
C -2.234507 2.923296 1.306140
O -0.190648 2.396812 -0.295018
F -2.203276 4.230961 1.097479
F -3.478932 2.546762 1.552041
F -1.473312 2.630610 2.344052
C 2.736760 -1.272962 0.479858
C 4.132125 -1.248147 0.442510
C 4.826454 -0.044787 0.557788
C 4.134934 1.151697 0.713319
C 2.739014 1.157934 0.752268
H 2.184503 -2.210645 0.396477
H 4.672610 -2.186478 0.327702
H 5.915027 -0.044853 0.544435
H 4.672714 2.092281 0.822628
H 2.184414 2.082557 0.904648
C -0.527941 -3.229473 0.578627
O 0.506825 -3.622159 0.050581
C -0.983552 -3.746808 1.940819
N -1.354091 -2.365665 -0.084646
C -0.996877 -1.921488 -1.410322
C 0.032698 -0.861963 -1.472369
C 0.732921 -0.029864 -2.048926
C 1.541114 0.973623 -2.641823
C 0.958741 2.056082 -3.338264
C 1.780193 3.064489 -3.825859
C 3.160363 3.003205 -3.647333
C 3.736142 1.926233 -2.982626
C 2.930507 0.912301 -2.482372
H -2.104980 -1.882939 0.392543
H -0.628446 -2.784309 -1.979110
H -1.890633 -1.522348 -1.906777
C -0.507385 2.053230 -3.620698
H 1.327915 3.898583 -4.356030
H 3.788655 3.800997 -4.039126
H 4.814758 1.873393 -2.846838
H 3.364349 0.067612 -1.949629
C -2.119499 -2.937290 2.560383
C -1.453744 -5.186512 1.698919
C 0.226710 -3.753595 2.873894
H -0.047767 -4.208642 3.835562
H 1.048741 -4.330640 2.435139
H 0.584666 -2.732914 3.066698
H -1.756836 -5.643651 2.651118
H -2.316856 -5.215303 1.018338
H -0.648804 -5.790476 1.262292
H -2.350809 -3.339290 3.555863
H -1.849315 -1.879203 2.688225
H -3.045600 -3.004248 1.970447
O -0.962456 3.277991 -3.873439
O -1.176880 1.044502 -3.656515
C -2.362845 3.384759 -4.194992
C -2.766593 4.813126 -3.949881
H -2.490355 3.082633 -5.244129
H -2.916604 2.686465 -3.561333
H -3.821932 4.955951 -4.210177
H -2.636248 5.061889 -2.890291
H -2.166302 5.507914 -4.549950

TS_{rc}^{A}

63
Energy: -4233.29269306
Cu -0.230472 -0.738074 -0.766479
Cl 0.455661 -0.971860 -2.791650
C -1.402417 -2.167664 -1.148963
O 1.296476 0.490688 -0.178503
S 1.408582 1.760964 0.610888

O 2.388201 1.654561 1.700841
C 2.215525 2.841502 -0.627687
O 0.138915 2.403688 0.937607
F 2.423038 4.046065 -0.118048
F 3.378193 2.314943 -0.990609
F 1.438477 2.951982 -1.697631
C -1.005708 -3.469557 -0.897767
C -1.928091 -4.491634 -1.118984
C -3.205179 -4.201929 -1.589636
C -3.566455 -2.884481 -1.848689
C -2.657741 -1.847566 -1.635393
H -0.002864 -3.687436 -0.537609
H -1.636183 -5.522704 -0.922980
H -3.917196 -5.006816 -1.764269
H -4.559214 -2.651715 -2.231767
H -2.926637 -0.818107 -1.863683
C 2.481799 -1.680466 1.070792
O 1.469634 -2.258277 0.632077
C 3.815175 -1.702529 0.356682
N 2.382292 -1.126922 2.305447
C 1.048637 -1.284167 2.864773
C 0.050580 -1.269493 1.786360
C -0.903435 -0.832987 1.075994
C -2.200461 -0.306226 1.488914
C -2.801227 0.851277 0.957573
C -4.014334 1.301539 1.483182
C -4.662382 0.606858 2.494299
C -4.088162 -0.553026 3.002075
C -2.869272 -0.995063 2.509438
H 2.835613 -0.221274 2.439828
H 0.999825 -2.223418 3.433892
H 0.838424 -0.445856 3.542783
C -2.205832 1.584102 -0.187000
H -4.450236 2.209792 1.075323
H -5.613201 0.968230 2.880156
H -4.585695 -1.115319 3.790111
H -2.417815 -1.904418 2.902194
C 3.614989 -1.509828 -1.143825
C 4.777097 -0.648319 0.898359
C 4.378716 -3.106647 0.631386
H 5.359144 -3.200925 0.145807
H 4.512262 -3.283902 1.707842
H 3.717297 -3.882787 0.226201
H 5.721222 -0.713569 0.342174
H 4.382641 0.368652 0.769954
H 5.012861 -0.807379 1.960010
H 4.576565 -1.662239 -1.652499
H 2.886848 -2.219921 -1.553154
H 3.253719 -0.499951 -1.370799
O -2.591656 2.846352 -0.246238
O -1.492922 1.068534 -1.035068
C -2.012686 3.638855 -1.303309
C -2.515888 5.045405 -1.125739
H -0.923229 3.565292 -1.214960
H -2.304393 3.202111 -2.266873
H -2.096776 5.692827 -1.904469
H -3.609459 5.087763 -1.196188
H -2.215496 5.442850 -0.149558

TS_{ar}^{B}

63
Energy: -4233.29165941
Cu 0.000339 -1.218034 0.384914
Cl -0.526988 -1.989637 2.349357
C 1.671194 -2.185327 0.400423
O -1.559803 0.003981 0.420550
S -1.404977 1.504018 0.420161
O -1.986110 2.110911 -0.779560
C -2.531260 1.960247 1.792343
O -0.077355 1.942286 0.845376
F -2.559095 3.282746 1.902298
F -3.756147 1.520301 1.534342
F -2.105507 1.435345 2.924125
C 1.778680 -3.249012 -0.484260
C 2.676933 -4.273813 -0.187871
C 3.459330 -4.207837 0.959950
C 3.350620 -3.117354 1.822395
C 2.454011 -2.092980 1.546881
H 1.164111 -3.288761 -1.381471
H 2.764835 -5.120081 -0.866743
H 4.163024 -5.007675 1.183276

H 3.954490 -3.068519 2.726663
H 2.357132 -1.243282 2.219350
C -2.168066 -1.671174 -1.488530
O -1.310645 -2.466751 -1.081020
C -3.645040 -1.861778 -1.191027
N -1.793626 -0.638419 -2.291342
C -0.386199 -0.470141 -2.537267
C 0.512766 -0.487260 -1.358517
C 1.645104 -0.408707 -0.817465
C 2.901778 0.284944 -0.628361
C 2.941436 1.695943 -0.713815
C 4.142919 2.358471 -0.473006
C 5.309503 1.651010 -0.210466
C 5.286240 0.260951 -0.194223
C 4.089928 -0.413926 -0.387170
H -2.341831 0.216382 -2.246090
H -0.037163 -1.272182 -3.204457
H -0.216900 0.495476 -3.030566
C 1.781449 2.496707 -1.207613
H 4.153285 3.444992 -0.515539
H 6.240267 2.186897 -0.035155
H 6.199976 -0.303599 -0.020992
H 4.071482 -1.499757 -0.358655
C -4.482999 -0.625662 -1.505697
C -4.091674 -3.026619 -2.086094
C -3.813065 -2.246669 0.278737
H -5.154291 -3.239866 -1.906964
H -3.967852 -2.784247 -3.151379
H -3.514463 -3.932851 -1.864437
H -4.866348 -2.495362 0.468362
H -3.195717 -3.114836 0.535599
H -3.521519 -1.422294 0.941411
H -5.531245 -0.828812 -1.249935
H -4.166067 0.246851 -0.917287
H -4.457653 -0.365431 -2.573647
O 1.690788 3.667831 -0.594278
O 1.075992 2.133009 -2.124897
C 0.556242 4.492042 -0.935669
C 0.231065 5.325608 0.274610
H 0.836801 5.100441 -1.806628
H -0.278189 3.841610 -1.221233
H -0.608633 5.995146 0.053722
H -0.053492 4.673499 1.108645
H 1.088374 5.936068 0.583401

I_2

63
Energy: -4233.33903949
Cu 0.100677 -1.435336 -1.990217
Cl 1.052103 -3.405516 -2.321783
C 1.517035 0.553395 -0.505829
O -0.480759 -1.749341 -0.236784
S -1.976973 -2.077160 -0.172308
O -2.562857 -2.037422 -1.516156
C -1.962058 -3.851323 0.308667
O -2.641912 -1.397225 0.923126
F -3.218230 -4.232075 0.503092
F -1.425767 -4.588650 -0.639323
F -1.282623 -3.999479 1.432873
C 2.370256 -0.266462 -1.253151
C 3.432953 -0.923218 -0.636943
C 3.661085 -0.749173 0.720950
C 2.832842 0.093206 1.463434
C 1.769112 0.743957 0.856120
H 2.246770 -0.342510 -2.335077
H 4.072051 -1.576810 -1.227042
H 4.486615 -1.266452 1.206133
H 3.013214 0.232188 2.528020
H 1.102815 1.378935 1.441155
C -0.965938 -0.839853 -4.380521
O 0.142956 -1.080014 -3.826493
C -1.292419 -1.606068 -5.646806
N -1.859191 -0.009128 -3.859494
C -1.748429 0.659770 -2.565437
C -0.484393 0.369742 -1.857103
C 0.314884 1.150747 -1.135540
C 0.116395 2.612547 -0.909839
C -0.846154 3.128399 -0.029448
C -0.858697 4.493014 0.266804
C 0.043056 5.356675 -0.339301
C 0.995405 4.852294 -1.220527

C 1.042885 3.489363 -1.481290
H -2.760915 0.051621 -4.315547
H -1.813415 1.748001 -2.725237
H -2.609831 0.364005 -1.954473
C -1.882352 2.252965 0.593817
H -1.591080 4.870517 0.978400
H 0.011561 6.421056 -0.114574
H 1.715704 5.519381 -1.690639
H 1.816535 3.079512 -2.130914
C -2.442451 -0.995070 -6.441263
C -0.039482 -1.692808 -6.517486
C -1.678052 -3.009982 -5.147630
H -1.937242 -3.639657 -6.009505
H -0.846792 -3.478154 -4.603611
H -2.544225 -2.970426 -4.471729
H -0.251522 -2.323242 -7.390729
H 0.270450 -0.703151 -6.880169
H 0.793088 -2.137609 -5.961905
H -2.574457 -1.562348 -7.371078
H -3.403939 -1.053327 -5.909321
H -2.247722 0.050646 -6.718006
O -1.877359 2.355385 1.922568
O -2.656523 1.565725 -0.033172
C -2.859799 1.545165 2.608834
C -2.601205 1.694795 4.083566
H -3.857751 1.901271 2.320238
H -2.762871 0.511117 2.257379
H -3.327364 1.102971 4.652567
H -1.596249 1.339721 4.340193
H -2.686974 2.741959 4.398246

TS_{rc}^{B}

63
Energy: -4233.31991983
Cu 1.530364 1.208266 0.105514
Cl 2.092993 3.111396 -0.816511
C -0.535296 -1.159249 1.369241
O 2.416607 -0.287426 0.901289
S 2.977690 -1.221666 -0.156839
O 2.623385 -0.775420 -1.508803
C 4.772616 -0.883276 -0.005164
O 2.793172 -2.627288 0.166647
F 5.433595 -1.600959 -0.900849
F 5.000114 0.403377 -0.222196
F 5.194197 -1.203120 1.207768
C -0.910091 -0.305418 2.413769
C -0.864232 -0.749272 3.729065
C -0.439917 -2.042254 4.020712
C -0.069802 -2.897154 2.987549
C -0.130449 -2.466063 1.669666
H -1.255385 0.698761 2.185923
H -1.163692 -0.078451 4.532765
H -0.396761 -2.382918 5.053969
H 0.274592 -3.905979 3.206406
H 0.189852 -3.130369 0.868846
C -0.971889 2.741317 -0.308322
O -0.467824 1.985648 0.563063
C -1.587093 4.057849 0.102598
N -1.026404 2.313040 -1.564057
C -0.388128 1.042219 -1.869535
C -0.067473 0.391067 -0.564574
C -0.570624 -0.732054 -0.041904
C -1.121938 -1.716356 -1.025427
C -0.257732 -2.476676 -1.811221
C -0.763083 -3.454298 -2.665035
C -2.132140 -3.681252 -2.739757
C -3.001679 -2.915323 -1.972882
C -2.512673 -1.921539 -1.123012
H -1.268132 2.949159 -2.311901
H -1.092039 0.397793 -2.415745
H 0.506810 1.208757 -2.486308
H 0.816014 -2.302477 -1.740036
H -0.075186 -4.041860 -3.270016
H -2.526887 -4.449207 -3.402110
H -4.075996 -3.071117 -2.035886
C -3.453068 -1.027841 -0.397467
C -0.692907 4.704825 1.160427
C -1.747864 5.000695 -1.086480
C -2.958495 3.707497 0.697094
H -2.139006 5.962651 -0.732432
H -0.783883 5.195077 -1.577718

H -2.468210 4.624114 -1.827918
H -3.454837 4.628433 1.031164
H -3.601650 3.210068 -0.041855
H -2.854402 3.034685 1.557847
H -1.136209 5.659307 1.473093
H -0.594216 4.062570 2.042677
H 0.313924 4.891725 0.764266
O -4.660444 -1.585788 -0.241246
O -3.186622 0.093091 -0.012917
C -5.640868 -0.773775 0.420167
C -6.905984 -1.584497 0.512269
H -5.252468 -0.490391 1.406969
H -5.778219 0.152907 -0.153420
H -7.689625 -1.007485 1.016615
H -7.269366 -1.858938 -0.485053
H -6.737142 -2.506138 1.081016

Reaction 11

reactant

38
Energy: -825.710226113
C -4.088895 -0.223412 0.218237
O -4.647020 -0.410269 -0.853417
C -4.883755 0.112921 1.484025
N -2.733095 -0.299114 0.320353
H -2.253150 -0.173641 1.199480
C -1.945627 -0.616234 -0.859688
C -0.523327 -0.506356 -0.580583
H -2.194396 -1.628389 -1.216297
H -2.240317 0.062540 -1.674660
C 0.659342 -0.413758 -0.335225
C 2.052009 -0.304805 -0.052212
C 2.942819 -1.298119 -0.482858
C 4.296927 -1.188878 -0.206457
C 4.791280 -0.092775 0.506784
C 3.900835 0.896652 0.936754
C 2.549249 0.797515 0.661846
H 2.556878 -2.152203 -1.035967
H 4.971783 -1.970816 -0.551381
C 6.237205 0.074772 0.834761
H 4.305102 1.742567 1.489182
H 1.856732 1.569548 0.992264
C -4.029091 0.288412 2.735243
C -5.642230 1.410234 1.196043
C -5.881452 -1.026176 1.700288
H -4.675225 0.523305 3.591819
H -3.477486 -0.627952 2.992248
H -3.314887 1.119059 2.636183
H -6.546189 -0.791146 2.543318
H -6.490336 -1.178209 0.801979
H -5.365929 -1.969950 1.928227
H -6.302533 1.656983 2.039246
H -4.950398 2.251862 1.049393
H -6.250350 1.305940 0.290291
O 6.622764 1.043375 1.462536
C 7.204867 -0.984121 0.369429
H 7.188332 -1.083456 -0.724149
H 8.211986 -0.706145 0.690884
H 6.949447 -1.966535 0.788638

I_c

73
Energy: -4426.33558341
Cu 0.681691 0.047601 -0.508006
Cl 1.266175 -0.538823 -2.525990
C 1.938907 1.458825 -0.756009
O -0.620665 -1.479799 -0.336044
S -1.447640 -1.907369 0.847816
O -2.099366 -0.797135 1.546706
C -2.823384 -2.758804 -0.015961
O -0.814644 -2.938327 1.661195
F -3.709591 -3.187187 0.866238
F -3.433295 -1.909028 -0.850218
F -2.369335 -3.782405 -0.717491
C 1.394537 2.706175 -0.520664
C 2.278306 3.787188 -0.432174
C 3.642708 3.604583 -0.607910
C 4.146551 2.333792 -0.874464
C 3.288760 1.238665 -0.958570

C -1.086317 2.995541 2.088332
O -0.224822 3.860725 1.970392
C -2.570479 3.365229 2.069557
N -0.768681 1.683522 2.288862
C 0.606210 1.293481 2.424942
C 1.079578 0.215824 1.535725
C 1.773470 -0.770445 1.260454
C 2.664791 -1.817031 0.903045
C 2.197168 -3.102570 0.590163
C 3.107398 -4.086228 0.237259
C 4.478012 -3.813423 0.201240
C 4.938928 -2.533584 0.528417
C 4.045338 -1.537319 0.871904
H 0.332431 2.869332 -0.360192
H 1.867891 4.768076 -0.198552
H 4.318811 4.455051 -0.542580
H 5.213190 2.184327 -1.035924
H 3.671695 0.249140 -1.200006
H -1.465597 0.945442 2.218430
H 1.207708 2.196482 2.237677
H 0.821662 0.960507 3.453119
H 1.130067 -3.307127 0.639402
H 2.736695 -5.079204 -0.009407
C 5.497949 -4.844641 -0.175182
H 6.010883 -2.350529 0.499751
H 4.391026 -0.536785 1.127536
C -3.512326 2.179900 2.247315
C -2.780373 4.359542 3.215258
C -2.847437 4.059441 0.735137
O -3.305467 1.089259 -0.962246
C -4.527816 0.763271 -1.639573
C -5.603468 0.740350 -0.586522
C -2.109398 0.899517 -1.504800
O -1.139611 1.215289 -0.829123
C -2.025545 0.326143 -2.881853
H -4.720210 1.526484 -2.407457
H -4.433861 -0.212041 -2.131651
H -6.571765 0.488873 -1.033857
H -5.361339 -0.008501 0.177813
H -5.686542 1.718458 -0.097357
H -2.766865 0.770115 -3.555343
H -1.017863 0.470598 -3.275775
H -2.211967 -0.755134 -2.829062
H -3.353685 1.668490 3.207482
H -4.550962 2.541052 2.242111
H -3.410118 1.445140 1.438113
H -3.891194 4.404843 0.701616
H -2.187455 4.925696 0.606644
H -2.687984 3.369301 -0.103747
H -3.818115 4.721830 3.209394
H -2.593761 3.886797 4.190132
H -2.105781 5.217468 3.114867
O 6.680697 -4.563454 -0.173715
C 5.019366 -6.221236 -0.552282
H 4.338253 -6.181397 -1.412751
H 5.885332 -6.838575 -0.804515
H 4.469635 -6.689556 0.275092

I_1

59
Energy: -4118.81398767
Cu -0.025169 0.132249 0.007543
Cl -0.014564 0.296573 2.132634
C 1.837896 0.199878 0.337464
O -2.048474 0.337345 -0.286607
S -2.047832 1.724717 -0.885272
O -2.704695 1.849336 -2.172671
C -3.055628 2.684524 0.309014
O -0.673789 2.264075 -0.761866
F -3.082258 3.951919 -0.070085
F -4.287521 2.202712 0.328488
F -2.528312 2.596703 1.516168
C 2.598177 -0.948021 0.435552
C 3.982184 -0.792247 0.335643
C 4.545839 0.471652 0.177818
C 3.735833 1.601146 0.123679
C 2.349018 1.479489 0.229325
H 2.146398 -1.933474 0.556505
H 4.616539 -1.675836 0.387778
H 5.627073 0.577658 0.111447
H 4.175064 2.591786 0.017625

H 1.701140 2.354661 0.222306
C -0.449062 -3.212471 0.700170
O 0.610438 -3.544370 0.180391
C -0.803394 -3.599036 2.133619
N -1.389685 -2.539964 -0.030367
C -1.120298 -2.230022 -1.414256
C -0.148701 -1.139748 -1.634243
C 0.580471 -0.333651 -2.208560
C 1.516168 0.525345 -2.848776
C 1.133518 1.804498 -3.276807
C 2.072484 2.628948 -3.877439
C 3.388789 2.193357 -4.059229
C 3.759422 0.911518 -3.639468
C 2.833868 0.077939 -3.040221
H -2.174889 -2.081207 0.414002
H -0.722668 -3.127455 -1.905591
H -2.063203 -1.949070 -1.896954
H 0.111837 2.139718 -3.111867
H 1.769963 3.622632 -4.202302
C 4.441529 3.050579 -4.692547
H 4.789883 0.598180 -3.793024
H 3.113147 -0.915720 -2.694226
C -2.022653 -2.865994 2.686054
C -1.084380 -5.106304 2.097277
C 0.416079 -3.326336 3.013567
H 0.223857 -3.681881 4.035138
H 1.298406 -3.846810 2.623285
H 0.639028 -2.251439 3.061832
H -1.305588 -5.467378 3.111178
H -1.949632 -5.335838 1.458873
H -0.215772 -5.655003 1.713061
H -2.179268 -3.158909 3.732693
H -1.890308 -1.774618 2.668115
H -2.943601 -3.126978 2.144027
O 5.579468 2.638246 -4.807771
C 4.053216 4.422593 -5.176838
H 3.258179 4.365405 -5.932245
H 3.672725 5.041077 -4.352947
H 4.932010 4.904369 -5.613069

TS_{rc}^{A}

59
Energy: -4118.79274690
Cu -0.261831 -0.798904 0.818221
Cl -0.986373 -1.404708 2.731601
C 0.776433 -2.364687 0.860274
O -0.948507 1.049568 0.928559
S -1.533428 2.061569 -0.019536
O -2.955087 1.842484 -0.291919
C -1.450506 3.563418 1.028217
O -0.690559 2.361354 -1.177869
F -1.932293 4.592132 0.347101
F -2.157406 3.398859 2.130976
F -0.186134 3.809578 1.348279
C 0.357861 -3.508866 0.205880
C 1.237474 -4.590020 0.148851
C 2.490263 -4.513667 0.750804
C 2.871223 -3.357256 1.423368
C 2.004802 -2.266621 1.491456
H -0.621612 -3.558506 -0.265220
H 0.934364 -5.496523 -0.373476
H 3.168851 -5.363472 0.703516
H 3.844542 -3.296429 1.907716
H 2.295470 -1.363457 2.028251
C -2.874557 -0.958295 -1.317853
O -1.906673 -1.557104 -0.803217
C -4.278271 -1.092322 -0.773525
N -2.617450 -0.281317 -2.452206
C -1.234623 -0.323192 -2.899815
C -0.341758 -0.581936 -1.760895
C 0.580371 -0.420853 -0.920329
C 1.956648 0.056458 -0.872972
C 2.270542 1.322958 -0.372272
C 3.599903 1.717469 -0.301767
C 4.625789 0.864018 -0.716002
C 4.298166 -0.403945 -1.206507
C 2.978268 -0.811470 -1.281223
H -3.225499 0.474232 -2.743828
H -1.115169 -1.090908 -3.678153
H -0.953676 0.656681 -3.308779
H 1.472560 1.993753 -0.062632

H 3.829825 2.709281 0.084063
C 6.068523 1.240227 -0.653947
H 5.109036 -1.058228 -1.520490
H 2.720721 -1.809411 -1.635473
C -4.240017 -0.941587 0.747593
C -5.244293 -0.085211 -1.389613
C -4.715078 -2.518778 -1.145229
H -5.730671 -2.690932 -0.765283
H -4.730509 -2.666234 -2.234603
H -4.049286 -3.266852 -0.697991
H -6.241061 -0.241085 -0.957779
H -4.941799 0.945619 -1.168456
H -5.345282 -0.219599 -2.477058
H -5.251537 -1.097769 1.145758
H -3.569140 -1.676139 1.209753
H -3.899557 0.060665 1.035191
O 6.928248 0.464125 -1.028060
C 6.431441 2.601127 -0.116179
H 7.518804 2.710613 -0.139495
H 5.973876 3.399411 -0.715644
H 6.076678 2.727292 0.915452

TS_{ar}^{B}

59
Energy: -4118.80267919
Cu 0.497427 0.780171 0.470825
Cl 0.974535 1.109649 2.557588
C -1.011528 2.005684 0.554662
O 1.830547 -0.686234 0.335227
S 1.575832 -2.104556 -0.115426
O 2.172024 -2.375473 -1.427890
C 2.611452 -3.016110 1.093103
O 0.206572 -2.553036 0.112684
F 2.561413 -4.310122 0.818762
F 3.868743 -2.601209 1.007516
F 2.161955 -2.805692 2.315217
C -0.861560 3.190825 -0.153207
C -1.628341 4.291887 0.225463
C -2.537736 4.185231 1.271959
C -2.684067 2.978624 1.955206
C -1.917993 1.876267 1.601614
H -0.151148 3.260021 -0.974295
H -1.514284 5.231412 -0.312180
H -3.141826 5.044973 1.555528
H -3.389223 2.895289 2.779901
H -2.021686 0.932452 2.133094
C 2.759417 1.251442 -1.277148
O 1.979179 2.059345 -0.752827
C 4.237270 1.228768 -0.930439
N 2.305148 0.404119 -2.236826
C 0.894568 0.405450 -2.527946
C -0.037851 0.404307 -1.374393
C -1.168963 0.452091 -0.823683
C -2.532004 -0.024467 -0.705376
C -2.745134 -1.380691 -0.440864
C -4.048145 -1.859441 -0.369918
C -5.132670 -1.003231 -0.567681
C -4.903026 0.352787 -0.831967
C -3.614206 0.847235 -0.889109
H 2.761103 -0.503341 -2.313308
H 0.644131 1.299910 -3.118052
H 0.675730 -0.476687 -3.144229
H -1.887867 -2.035691 -0.287244
H -4.207994 -2.915040 -0.157801
C -6.554072 -1.469837 -0.505744
H -5.765307 0.998820 -0.982514
H -3.427783 1.904440 -1.074946
C 4.964148 0.008028 -1.487529
C 4.822643 2.503785 -1.553767
C 4.387611 1.281892 0.590492
H 6.017654 0.047960 -1.181511
H 4.548659 -0.933989 -1.102490
H 4.947541 -0.019306 -2.586413
H 5.452073 1.378247 0.844400
H 3.847365 2.136145 1.014274
H 3.995865 0.371883 1.061526
O -7.465010 -0.685736 -0.689747
C -6.819335 -2.922699 -0.211133
H -7.899557 -3.089041 -0.202200
H -6.359045 -3.571221 -0.968448
H -6.399950 -3.212602 0.761522

H 5.893863 2.566393 -1.319265
H 4.713923 2.501989 -2.647883
H 4.327569 3.397352 -1.154392

I_2

59
Energy: -4118.85640521
Cu 0.219361 -1.490472 -1.953244
Cl 0.821289 -3.565915 -2.381859
C 2.058084 0.360452 -0.728601
O -0.131187 -1.752004 -0.128715
S -1.638472 -1.655001 0.128408
O -2.386466 -1.876934 -1.112482
C -1.893000 -3.147666 1.166864
O -2.003823 -0.507798 0.946628
F -3.176777 -3.214094 1.486584
F -1.547599 -4.226542 0.497362
F -1.170036 -3.048633 2.267804
C 2.668166 -0.508505 -1.643588
C 3.778667 -1.260275 -1.270194
C 4.301274 -1.135516 0.010192
C 3.720551 -0.246408 0.914315
C 2.607855 0.498073 0.549882
H 2.324121 -0.540546 -2.678745
H 4.226546 -1.945661 -1.986725
H 5.166000 -1.726064 0.306527
H 4.132188 -0.144312 1.916853
H 2.134590 1.168740 1.266168
C -1.115846 -0.728911 -4.163434
O 0.017782 -1.121155 -3.773122
C -1.677508 -1.375745 -5.414717
N -1.838871 0.163838 -3.497651
C -1.475074 0.790740 -2.227374
C -0.153256 0.357262 -1.718121
C 0.799037 1.048356 -1.086011
C 0.609791 2.458224 -0.673210
C -0.535986 2.835538 0.034511
C -0.707041 4.155981 0.433323
C 0.259512 5.117861 0.132031
C 1.412060 4.732822 -0.562836
C 1.592582 3.417289 -0.952053
H -2.772126 0.354715 -3.840021
H -1.442881 1.881460 -2.382274
H -2.281920 0.570811 -1.513246
H -1.266259 2.074667 0.313432
H -1.599311 4.427172 0.995614
C 0.127548 6.551660 0.530078
H 2.156506 5.496072 -0.780885
H 2.495451 3.116113 -1.482901
C -2.867597 -0.623486 -6.001486
C -0.564057 -1.488259 -6.456449
C -2.109340 -2.779945 -4.958537
H -2.505963 -3.331968 -5.821244
H -1.262431 -3.338137 -4.538062
H -2.894253 -2.727069 -4.190969
H -0.944993 -2.030087 -7.331668
H -0.222019 -0.499541 -6.791604
H 0.294475 -2.037206 -6.055443
H -3.177967 -1.117374 -6.930646
H -3.746698 -0.638488 -5.339701
H -2.620738 0.418285 -6.250587
O 0.988209 7.358102 0.231858
C -1.094426 6.970071 1.307502
H -1.028578 8.040754 1.517579
H -2.013544 6.768241 0.741344
H -1.169524 6.418662 2.254254

TS_{rc}^{B}

59
scf done: -4118.82965342
Cu -1.867837 0.445921 -0.042614
Cl -3.596971 0.259589 -1.396652
C 0.518158 -0.604902 2.203456
O -1.113221 2.019625 0.716308
S -0.066477 2.714733 -0.142327
O 0.189401 1.988121 -1.387995
C -0.981175 4.219934 -0.650579
O 1.075291 3.170715 0.635505
F -0.211091 4.952366 -1.440805
F -2.076114 3.872284 -1.307293

F -1.314420 4.928226 0.415539
C -0.712257 -0.268558 2.787479
C -0.859612 -0.230314 4.163213
C 0.220315 -0.526352 4.991603
C 1.448427 -0.858289 4.429250
C 1.598353 -0.895478 3.049851
H -1.554478 -0.010869 2.152410
H -1.820997 0.045961 4.591299
H 0.105118 -0.489526 6.073317
H 2.299312 -1.086980 5.068207
H 2.563174 -1.158848 2.620393
C -2.051389 -2.352692 -0.711345
O -1.893309 -1.673854 0.340325
C -3.079860 -3.458880 -0.759706
N -1.214704 -2.140171 -1.725460
C -0.310981 -1.000846 -1.616407
C -0.270381 -0.618555 -0.174545
C 0.694856 -0.690959 0.751601
C 2.081445 -0.785944 0.202625
C 2.886338 0.351761 0.133566
C 4.180875 0.245431 -0.361806
C 4.683055 -0.985636 -0.790712
C 3.868103 -2.119953 -0.717970
C 2.575775 -2.022684 -0.228175
H -1.425899 -2.520042 -2.639683
H 0.700138 -1.286757 -1.935626
H -0.654392 -0.176751 -2.258302
H 2.490340 1.316768 0.454447
H 4.795685 1.142179 -0.417788
C 6.065250 -1.154136 -1.334646
H 4.280598 -3.070286 -1.051399
H 1.938073 -2.906019 -0.162907
C -4.287158 -3.050645 0.083203
C -3.518969 -3.743414 -2.194429
C -2.404152 -4.696329 -0.151332
H -4.321906 -4.490825 -2.181107
H -3.911150 -2.838744 -2.678976
H -2.710671 -4.167738 -2.808753
H -3.121428 -5.527412 -0.137284
H -1.529190 -5.010031 -0.737347
H -2.082884 -4.503677 0.880229
H -5.030266 -3.858124 0.058352
H -4.001897 -2.874805 1.126534
H -4.744545 -2.131684 -0.304981
O 6.456065 -2.248093 -1.696572
C 6.953052 0.059714 -1.422513
H 6.507331 0.830084 -2.065834
H 7.104505 0.512779 -0.433723
H 7.920147 -0.239545 -1.834774

Reaction 12

reactant

38
Energy: -825.709700574
C -3.967944 -0.481618 0.367830
O -4.597690 -1.139184 -0.448588
C -4.669726 0.244682 1.519774
N -2.614245 -0.370947 0.278109
H -2.073848 0.163835 0.942228
C -1.913329 -1.043804 -0.802770
C -0.485558 -0.779215 -0.736109
H -2.113690 -2.125732 -0.753296
H -2.329676 -0.713180 -1.767288
C 0.700244 -0.545254 -0.661648
C 2.098746 -0.274950 -0.580501
C 3.006644 -0.967830 -1.397191
C 4.366250 -0.702626 -1.315945
C 4.841574 0.251919 -0.422578
C 3.951613 0.949932 0.398116
C 2.585798 0.681940 0.312694
H 2.628223 -1.714098 -2.093499
H 5.061713 -1.244823 -1.953736
H 5.911027 0.449219 -0.369130
C 4.396999 1.986023 1.378613
H 1.906932 1.235870 0.958167
C -3.729834 1.009159 2.446643
C -5.667782 1.216706 0.887600
C -5.426144 -0.817310 2.319722
H -4.312174 1.495884 3.240611

H -3.005670 0.345790 2.942011
H -3.182467 1.803998 1.918928
H -6.026417 -0.340419 3.107204
H -6.093110 -1.388453 1.664092
H -4.733307 -1.521822 2.801501
H -6.267885 1.703326 1.669241
H -5.152001 2.002622 0.317719
H -6.340691 0.685389 0.205013
O 3.588651 2.568226 2.076722
C 5.870494 2.289135 1.479185
H 6.269276 2.640116 0.518026
H 6.019970 3.063468 2.236100
H 6.440803 1.393501 1.759645

I$_c$

73
Energy: -4426.33979275
Cu 0.738829 0.072982 -0.426077
Cl 1.366039 -0.621897 -2.402251
C 1.973927 1.496919 -0.706376
O -0.552930 -1.478508 -0.206538
S -1.382526 -1.889583 0.981224
O -2.099504 -0.779053 1.611146
C -2.689006 -2.863076 0.139679
O -0.716538 -2.849554 1.854806
F -3.558174 -3.310042 1.028328
F -3.338258 -2.096107 -0.741456
F -2.154058 -3.887218 -0.506422
C 1.402630 2.740733 -0.526077
C 2.263068 3.843271 -0.487870
C 3.631192 3.681413 -0.653670
C 4.162472 2.410151 -0.857697
C 3.328923 1.293729 -0.889358
C -1.086896 3.043128 2.063384
O -0.237541 3.918502 1.935046
C -2.576268 3.388325 2.018034
N -0.749695 1.740798 2.297391
C 0.628917 1.379558 2.471471
C 1.143657 0.306612 1.598838
C 1.874465 -0.663219 1.355786
C 2.732260 -1.732560 0.995457
C 2.182481 -2.963355 0.609430
C 3.010223 -3.999267 0.204763
C 4.397118 -3.818339 0.215581
C 4.951492 -2.605745 0.611062
C 4.126803 -1.553866 0.988926
H 0.337232 2.887727 -0.372467
H 1.831608 4.825036 -0.300222
H 4.289354 4.547898 -0.626605
H 5.232814 2.276071 -1.007834
H 3.733600 0.300862 -1.076750
H -1.436935 0.992230 2.248770
H 1.214591 2.294001 2.289506
H 0.824874 1.060647 3.507736
H 1.103782 -3.075220 0.629645
C 2.445849 -5.305712 -0.260081
H 5.022339 -4.652186 -0.099608
H 6.031508 -2.474778 0.615416
H 4.543653 -0.593281 1.288637
C -3.501561 2.193745 2.219129
C -2.814173 4.410191 3.133759
C -2.849680 4.041537 0.662183
O -3.258300 1.043722 -0.978745
C -4.461928 0.617251 -1.634609
C -5.540113 0.606045 -0.584263
C -2.051647 0.821756 -1.484037
O -1.095409 1.191917 -0.815983
C -1.939904 0.153413 -2.815474
H -4.686566 1.320832 -2.449152
H -4.318579 -0.381370 -2.063989
H -6.497366 0.302232 -1.022855
H -5.277311 -0.099868 0.213326
H -5.657797 1.602080 -0.140566
H -2.678182 0.536759 -3.528273
H -0.928921 0.285746 -3.205358
H -2.108942 -0.924544 -2.689025
H -3.348232 1.714484 3.196673
H -4.545469 2.537837 2.188979
H -3.376916 1.435500 1.435184
H -3.897307 4.372653 0.610611
H -2.199732 4.912640 0.515922

H -2.674988 3.330948 -0.156324
H -3.857597 4.754709 3.107607
H -2.629962 3.967746 4.123225
H -2.152861 5.276224 3.016351
O 3.187657 -6.228497 -0.536107
C 0.950073 -5.408214 -0.390174
H 0.444962 -5.211242 0.566001
H 0.573128 -4.657740 -1.100704
H 0.690865 -6.409784 -0.742997

I_1

59
Energy: -4118.81746594
Cu 0.089768 0.072820 -0.029502
Cl 0.041645 0.280187 2.090665
C 1.947582 0.095215 0.327537
O -1.912500 0.359199 -0.408511
S -1.847739 1.758307 -0.972104
O -2.421254 1.926713 -2.296521
C -2.907399 2.715717 0.178435
O -0.474881 2.271655 -0.763727
F -2.890517 3.988544 -0.183270
F -4.145785 2.255043 0.119387
F -2.450679 2.600893 1.411014
C 2.680077 -1.068219 0.450860
C 4.069366 -0.942151 0.388408
C 4.664117 0.308899 0.240972
C 3.880404 1.454859 0.157762
C 2.488614 1.362793 0.223265
H 2.204437 -2.043210 0.565068
H 4.682862 -1.838829 0.462470
H 5.749035 0.391385 0.207600
H 4.342296 2.435879 0.059009
H 1.860436 2.251762 0.192904
C -0.442229 -3.231343 0.731916
O 0.632799 -3.615316 0.285266
C -0.896512 -3.568489 2.149441
N -1.311594 -2.544074 -0.070292
C -0.957953 -2.293790 -1.447098
C 0.006570 -1.193368 -1.658448
C 0.735256 -0.406484 -2.262882
C 1.603327 0.516461 -2.900024
C 1.100263 1.760417 -3.308180
C 1.952562 2.702983 -3.868612
C 3.303844 2.386360 -4.047014
C 3.803768 1.147985 -3.662469
C 2.960161 0.210447 -3.081282
H -2.115452 -2.061852 0.311348
H -0.516381 -3.207617 -1.864552
H -1.871855 -2.055181 -2.003458
H 0.045985 1.975469 -3.150164
C 1.475752 4.066638 -4.264278
H 3.946816 3.144864 -4.490672
H 4.857385 0.915662 -3.802931
H 3.337831 -0.752813 -2.741811
C -2.122361 -2.783114 2.607665
C -1.225989 -5.066370 2.130913
C 0.275477 -3.313516 3.096703
H 0.011004 -3.642318 4.111069
H 1.163232 -3.867444 2.770245
H 0.528055 -2.244985 3.138000
H -1.522157 -5.393735 3.137078
H -2.057309 -5.284003 1.444949
H -0.354450 -5.652785 1.815277
H -2.350894 -3.042549 3.649898
H -1.954489 -1.697189 2.570296
H -3.017294 -3.030554 2.017678
O 2.239543 4.846986 -4.799500
C 0.050311 4.429551 -3.951669
H -0.153389 4.326011 -2.876551
H -0.130897 5.460853 -4.265100
H -0.656599 3.764176 -4.465796

TS_{rc}^{A}

59
Energy: -4118.79457586
Cu 0.252432 0.925711 0.784615
Cl 1.057253 1.315717 2.722234
C -0.316773 2.715672 0.790475
O 0.315664 -1.044391 0.924454

S 0.758214 -2.166083 0.025033
O 2.207504 -2.212603 -0.175486
C 0.352559 -3.602691 1.088531
O -0.066513 -2.338221 -1.172746
F 0.683746 -4.717435 0.455290
F 1.009486 -3.535586 2.230911
F -0.953357 -3.614092 1.331084
C 0.412408 3.705684 0.157333
C -0.137100 4.986572 0.094797
C -1.376071 5.252492 0.670987
C -2.074431 4.240118 1.320849
C -1.541049 2.953462 1.392435
H 1.379433 3.488080 -0.291667
H 0.414055 5.778923 -0.410045
H -1.793726 6.256576 0.622267
H -3.036400 4.445768 1.787843
H -2.078585 2.161997 1.914510
C 2.922265 0.390328 -1.243041
O 2.121733 1.207440 -0.743966
C 4.302591 0.165680 -0.669383
N 2.543842 -0.181004 -2.402778
C 1.234015 0.214548 -2.891388
C 0.387940 0.674771 -1.780960
C -0.584357 0.783789 -0.991271
C -2.039928 0.662705 -1.023504
C -2.663447 -0.536155 -0.674760
C -4.052988 -0.635849 -0.723171
C -4.816521 0.471710 -1.101403
C -4.195910 1.669192 -1.427024
C -2.808902 1.772197 -1.386634
H 2.950458 -1.062039 -2.691940
H 1.339031 1.000230 -3.654059
H 0.731426 -0.654404 -3.337522
H -2.046930 -1.385684 -0.391445
C -4.763108 -1.910394 -0.392672
H -5.898819 0.362337 -1.130114
H -4.790314 2.533802 -1.716559
H -2.311580 2.710757 -1.630960
C 4.210892 0.102177 0.854483
C 4.968554 -1.090036 -1.224271
C 5.110164 1.405492 -1.088959
H 6.130649 1.316689 -0.693738
H 5.175570 1.495290 -2.182611
H 4.664827 2.323501 -0.686047
H 5.956507 -1.202200 -0.759825
H 4.383535 -1.988391 -0.992225
H 5.135971 -1.025035 -2.309986
H 5.223572 0.011281 1.269452
H 3.746581 1.005441 1.268364
H 3.619490 -0.762280 1.177705
O -5.975981 -1.974422 -0.465642
C -3.936448 -3.094669 0.032047
H -4.598085 -3.952048 0.180871
H -3.168981 -3.344700 -0.713342
H -3.404088 -2.881533 0.968719

TS_{ar}^{B}

59
Energy: -4118.80532177
Cu -0.339251 -0.942601 0.447906
Cl -0.755455 -1.234419 2.552595
C 1.047048 -2.310590 0.455815
O -1.492770 0.689511 0.382688
S -1.156293 2.071938 -0.115366
O -1.765416 2.354060 -1.419991
C -2.094650 3.088283 1.089412
O 0.245916 2.438541 0.063701
F -1.963552 4.366787 0.773195
F -3.379351 2.758649 1.045996
F -1.630409 2.884586 2.307109
C 0.750073 -3.467668 -0.252297
C 1.415357 -4.644607 0.087135
C 2.371256 -4.641820 1.096900
C 2.665783 -3.463369 1.781585
C 2.002559 -2.285066 1.466192
H 0.003846 -3.456962 -1.043838
H 1.185583 -5.561736 -0.452127
H 2.894972 -5.561467 1.350944
H 3.406128 -3.460584 2.579248
H 2.222268 -1.362714 1.999890
C -2.710000 -1.168096 -1.182453

O -1.995982 -2.050541 -0.683051
C -4.159272 -0.986448 -0.769833
N -2.211295 -0.382222 -2.171093
C -0.824174 -0.541064 -2.525136
C 0.154519 -0.611253 -1.413368
C 1.300147 -0.764128 -0.911052
C 2.692272 -0.377473 -0.827797
C 2.983512 0.969678 -0.594758
C 4.311040 1.390932 -0.550003
C 5.334715 0.461810 -0.749736
C 5.042691 -0.878011 -0.980311
C 3.722435 -1.307330 -1.006971
H -2.568971 0.568731 -2.242826
H -0.698033 -1.469888 -3.101784
H -0.542236 0.298653 -3.174293
H 2.156010 1.662111 -0.441120
C 4.683202 2.818684 -0.288747
H 6.361753 0.820875 -0.713349
H 5.847364 -1.594208 -1.133407
H 3.478075 -2.356835 -1.168621
C -4.770122 0.313596 -1.285609
C -4.909702 -2.181866 -1.373763
C -4.245493 -1.039957 0.755941
H -5.807442 0.385346 -0.933509
H -4.237175 1.199496 -0.912949
H -4.799475 0.351718 -2.383921
H -5.300609 -1.002197 1.059271
H -3.800886 -1.961826 1.147745
H -3.718440 -0.194418 1.215048
O 5.853620 3.148858 -0.275605
C 3.575204 3.805755 -0.045612
H 4.012735 4.788649 0.147314
H 2.901554 3.869574 -0.910848
H 2.950339 3.506330 0.806462
H -5.969110 -2.129469 -1.088571
H -4.852727 -2.178254 -2.471743
H -4.498314 -3.129406 -1.004732

I_2

59
Energy: -4118.85777827
Cu 0.200040 -1.458701 -1.955514
Cl 0.707875 -3.577980 -2.273615
C 2.127651 0.369519 -0.796654
O -0.188094 -1.612502 -0.120774
S -1.687648 -1.425641 0.119953
O -2.443812 -1.659961 -1.113106
C -2.029823 -2.852504 1.223313
O -1.997993 -0.224407 0.886416
F -3.315623 -2.830313 1.540013
F -1.745819 -3.976958 0.602049
F -1.303836 -2.746321 2.321438
C 2.693903 -0.561504 -1.679387
C 3.787858 -1.328752 -1.291242
C 4.341783 -1.157348 -0.029147
C 3.807215 -0.207568 0.840772
C 2.708902 0.551664 0.462493
H 2.331078 -0.629993 -2.706334
H 4.200507 -2.060922 -1.982202
H 5.195461 -1.758178 0.278339
H 4.243399 -0.068610 1.828409
H 2.271959 1.270679 1.154387
C -1.092942 -0.727585 -4.203251
O 0.015364 -1.166718 -3.790225
C -1.684920 -1.397310 -5.428412
N -1.767145 0.233112 -3.582733
C -1.378647 0.890252 -2.334888
C -0.079201 0.412321 -1.805904
C 0.887318 1.078217 -1.167782
C 0.680799 2.474385 -0.714118
C -0.438769 2.763296 0.069564
C -0.659756 4.055613 0.544562
C 0.251917 5.066145 0.228290
C 1.377201 4.781993 -0.535544
C 1.599716 3.488490 -0.998382
H -2.685931 0.461161 -3.941241
H -1.290198 1.970681 -2.531960
H -2.200585 0.739590 -1.619542
H -1.109060 1.945291 0.338203
C -1.841661 4.392743 1.396775
H 0.058645 6.068020 0.607380

H 2.090679 5.570313 -0.768853
H 2.487565 3.255869 -1.586581
C -2.843824 -0.618776 -6.042491
C -0.580109 -1.596320 -6.466467
C -2.175010 -2.762966 -4.917393
H -2.602048 -3.328822 -5.756248
H -1.350767 -3.341869 -4.480442
H -2.951244 -2.646253 -4.147877
H -0.985203 -2.156613 -7.318923
H -0.199744 -0.636273 -6.841403
H 0.256938 -2.163133 -6.045468
H -3.178927 -1.136172 -6.949991
H -3.719777 -0.569121 -5.378284
H -2.553476 0.400269 -6.335095
O -2.020092 5.534346 1.777738
C -2.792776 3.282259 1.759980
H -3.584107 3.687699 2.395835
H -3.241768 2.833591 0.862865
H -2.280131 2.464801 2.284597

TS_{rc}^{B}

59
Energy: -4118.83258414
Cu -1.301654 1.122961 0.163524
Cl -2.833774 2.194169 -1.008762
C 0.111090 -1.472616 1.930657
O 0.222848 1.843375 1.054449
S 1.455092 2.039063 0.186080
O 1.251226 1.544850 -1.178398
C 1.494978 3.865765 0.033117
O 2.684071 1.660616 0.868591
F 2.536309 4.216015 -0.706547
F 0.383922 4.287312 -0.547505
F 1.602035 4.416710 1.230510
C -0.712755 -0.642298 2.705899
C -0.765200 -0.778877 4.082179
C 0.007341 -1.747322 4.719569
C 0.836307 -2.572425 3.966932
C 0.891795 -2.435613 2.586716
H -1.296804 0.135390 2.222182
H -1.402473 -0.115016 4.662716
H -0.029902 -1.849466 5.802575
H 1.450549 -3.325362 4.457125
H 1.546235 -3.083742 2.006915
C -3.090526 -0.884322 -0.843249
O -2.537528 -0.632629 0.263080
C -4.574325 -1.163250 -0.908922
N -2.314143 -1.007583 -1.917286
C -0.906972 -0.652377 -1.772783
C -0.614762 -0.610449 -0.309138
C 0.160997 -1.375451 0.470998
C 1.239117 -2.102399 -0.269339
C 2.480895 -1.494598 -0.449208
C 3.488394 -2.156752 -1.150120
C 3.250810 -3.437309 -1.655118
C 2.015972 -4.046170 -1.474662
C 1.004458 -3.377401 -0.788649
H -2.729835 -1.016120 -2.840195
H -0.274896 -1.421415 -2.237154
H -0.705028 0.309268 -2.266782
H 2.646625 -0.497975 -0.040128
C 4.823291 -1.524879 -1.407076
H 4.056613 -3.931011 -2.195195
H 1.834485 -5.044500 -1.869088
H 0.029026 -3.844531 -0.643585
C -5.290501 -0.282513 0.114304
C -5.128199 -0.893845 -2.306202
C -4.745566 -2.644665 -0.542697
H -6.216333 -1.032599 -2.292277
H -4.926517 0.138247 -2.624219
H -4.735929 -1.593611 -3.059350
H -5.814562 -2.894789 -0.547349
H -4.237777 -3.301442 -1.262542
H -4.349480 -2.852051 0.459531
H -6.367293 -0.491856 0.074711
H -4.935905 -0.486164 1.131007
H -5.124669 0.781236 -0.098617
O 5.700655 -2.161930 -1.959319
C 5.018476 -0.095399 -0.987601
H 6.025344 0.222950 -1.270096
H 4.274279 0.558550 -1.463088

H 4.882635 0.033738 0.094369

Reaction 13

reactant

38
Energy: -825.705691123
C -4.135414 -0.039259 0.266156
O -4.713218 0.055691 -0.807000
C -4.903209 -0.298777 1.566039
N -2.780667 0.081106 0.340641
H -2.284392 0.026751 1.217975
C -2.020735 0.335175 -0.872226
C -0.591304 0.295964 -0.611618
H -2.304855 -0.409600 -1.631192
H -2.307191 1.311458 -1.296618
C 0.601819 0.316210 -0.400748
C 1.999324 0.220218 -0.123432
C 2.449794 -1.030137 0.333848
C 3.781644 -1.244752 0.647652
C 4.699349 -0.206297 0.510427
C 4.268627 1.031153 0.060761
C 2.930169 1.275266 -0.264490
H 1.721840 -1.833004 0.435618
H 4.103589 -2.223778 0.998216
H 5.748571 -0.363278 0.753296
H 4.963307 1.860009 -0.054848
C 2.631699 2.670829 -0.740281
C -4.022742 -0.395091 2.807924
C -5.897962 0.851767 1.730915
C -5.665743 -1.611365 1.376017
H -4.650111 -0.584616 3.689323
H -3.303272 -1.224463 2.742340
H -3.474570 0.538801 3.001488
H -6.310263 -1.802705 2.245394
H -6.290243 -1.565365 0.476724
H -4.976371 -2.461180 1.271349
H -6.542488 0.671674 2.602687
H -5.379177 1.808625 1.885011
H -6.528150 0.944682 0.839177
O 3.526921 3.496519 -0.754738
C 1.253817 3.056400 -1.203668
H 0.903225 2.406926 -2.015245
H 0.520901 2.964837 -0.392352
H 1.293692 4.093864 -1.547522

I_c

73
Energy: -4426.32990620
Cu 0.236538 -0.312517 -0.507136
Cl 0.702001 -1.152025 -2.461304
C 1.651340 0.950532 -0.830441
O -1.363763 -1.547474 -0.345397
S -2.418698 -1.825874 0.690257
O -3.323600 -0.688848 0.902101
C -3.422886 -3.075434 -0.200997
O -1.925507 -2.516547 1.876745
F -4.416090 -3.475296 0.573729
F -3.937774 -2.542909 -1.312013
F -2.672666 -4.110707 -0.529906
C 1.297626 2.179158 -0.308833
C 2.267009 3.184115 -0.305257
C 3.530554 2.949373 -0.833710
C 3.839577 1.702296 -1.364597
C 2.894833 0.674130 -1.363138
C -1.004328 2.535060 2.323496
O 0.147372 2.830116 2.622917
C -2.059379 3.611857 2.059097
N -1.390109 1.231796 2.210571
C -0.480793 0.183143 2.573409
C 0.481851 -0.303160 1.546924
C 1.477164 -0.980633 1.246162
C 2.799514 -1.459566 1.027272
C 3.152102 -2.727493 0.508195
C 4.504800 -2.994829 0.300321
C 5.491322 -2.068651 0.613543
C 5.144129 -0.831073 1.147385
C 3.807375 -0.526568 1.340788
H 0.321092 2.377468 0.118936
H 2.014421 4.143527 0.144154

H 4.277830 3.740935 -0.830665
H 4.822762 1.508518 -1.791476
H 3.141311 -0.301672 -1.774122
H -2.295642 0.958724 1.837900
H 0.136465 0.544768 3.406898
H -1.055987 -0.689556 2.909808
C 2.212025 -3.864707 0.169088
H 4.757560 -3.967961 -0.114357
H 6.537514 -2.315962 0.442133
H 5.909090 -0.098675 1.397067
H 3.506995 0.451574 1.715289
C -3.481262 3.066690 1.983633
C -1.957139 4.634250 3.191101
C -1.694282 4.280161 0.730146
O -3.508348 1.478099 -1.041875
C -4.800219 1.016393 -1.473145
C -5.803551 1.467685 -0.445890
C -2.397483 0.948841 -1.540474
O -1.350239 1.177922 -0.949192
C -2.485934 0.141500 -2.794706
H -5.018335 1.433990 -2.466748
H -4.781146 -0.078855 -1.542457
H -6.811583 1.162076 -0.749400
H -5.578026 1.011055 0.524869
H -5.790610 2.558716 -0.336128
H -3.199688 0.571866 -3.505460
H -1.493965 0.068849 -3.245528
H -2.811029 -0.878199 -2.552630
H -3.771267 2.550415 2.909622
H -4.184479 3.898445 1.833816
H -3.609107 2.379604 1.136732
H -2.388082 5.107484 0.523908
H -0.675814 4.689780 0.770476
H -1.756875 3.565062 -0.102504
H -2.637083 5.475319 2.996204
H -2.237631 4.189247 4.156067
H -0.934481 5.017050 3.278503
O 2.663439 -4.821102 -0.431230
C 0.778358 -3.824521 0.591832
H 0.626995 -3.430752 1.603461
H 0.199235 -3.183147 -0.087556
H 0.370811 -4.837585 0.523156

I_1

59
Energy: -4118.81111305
Cu 0.161451 0.104589 0.144087
Cl 0.177661 0.204838 2.277813
C 2.026501 0.043439 0.441691
O -1.818241 0.427675 -0.051077
S -1.892963 1.835866 -0.603722
O -2.888918 2.040882 -1.635812
C -2.495166 2.820931 0.825500
O -0.511197 2.306276 -0.844261
F -2.640507 4.079651 0.437195
F -3.663323 2.346721 1.225568
F -1.634848 2.774382 1.826368
C 2.728420 -1.146610 0.473450
C 4.121972 -1.060164 0.470190
C 4.758284 0.179947 0.466330
C 4.009485 1.351842 0.466860
C 2.614384 1.295034 0.470928
H 2.221162 -2.112686 0.478713
H 4.706965 -1.978643 0.476077
H 5.845694 0.230419 0.480787
H 4.501667 2.323122 0.482520
H 2.016788 2.205124 0.508272
C -0.480539 -3.215453 0.666221
O 0.583495 -3.609305 0.200761
C -0.948446 -3.615272 2.063449
N -1.326107 -2.455503 -0.092287
C -0.949318 -2.102603 -1.439611
C 0.046122 -1.012851 -1.554969
C 0.732803 -0.214543 -2.196014
C 1.558403 0.740944 -2.839515
C 0.994843 1.808100 -3.577347
C 1.844203 2.768087 -4.113597
C 3.225702 2.670577 -3.955146
C 3.777444 1.610727 -3.245117
C 2.946791 0.648619 -2.685999
H -2.106336 -1.962518 0.323650

H -0.533169 -2.991002 -1.930680
H -1.843702 -1.778320 -1.986801
C -0.483083 1.837281 -3.829634
H 1.427373 3.598854 -4.680193
H 3.872424 3.427672 -4.394911
H 4.855315 1.533332 -3.116448
H 3.360917 -0.176368 -2.108077
C -2.124220 -2.789070 2.578417
C -1.363320 -5.087708 1.956409
C 0.240037 -3.484711 3.015012
H -0.038359 -3.856025 4.010887
H 1.093237 -4.067466 2.649367
H 0.553716 -2.436414 3.115776
H -1.672607 -5.459828 2.943011
H -2.208911 -5.214563 1.265205
H -0.528273 -5.702165 1.597727
H -2.359801 -3.094224 3.606713
H -1.894510 -1.713755 2.599843
H -3.036267 -2.950487 1.984972
O -1.106264 0.794379 -3.862244
C -1.134889 3.170145 -4.061885
H -0.811213 3.898898 -3.308439
H -0.869744 3.563695 -5.053446
H -2.218102 3.039454 -4.004491

TS_{rc}^{A}

59
Energy: -4118.80223973
Cu 0.446431 0.573535 -0.759676
Cl -0.151316 1.316105 -2.685664
C 1.944610 1.706059 -0.946646
O -1.341744 -0.346125 -0.313161
S -1.745333 -1.638270 0.333440
O -2.770623 -1.449483 1.368313
C -2.620895 -2.453858 -1.051112
O -0.638767 -2.529254 0.683052
F -3.094382 -3.627495 -0.659846
F -3.625368 -1.689673 -1.454645
F -1.778136 -2.638539 -2.056971
C 1.891924 3.010231 -0.487180
C 3.049772 3.782761 -0.572839
C 4.215410 3.252074 -1.116992
C 4.229197 1.943121 -1.585758
C 3.081172 1.153788 -1.510158
H 0.973472 3.418322 -0.071261
H 3.030712 4.810738 -0.212809
H 5.112898 3.864619 -1.184763
H 5.134015 1.526373 -2.026357
H 3.078761 0.136950 -1.898480
C -1.913013 1.841141 1.321562
O -0.782149 2.209924 0.951869
C -3.187450 2.330687 0.669748
N -1.968503 1.068600 2.434680
C -0.643801 0.775589 2.961491
C 0.325185 0.706329 1.857952
C 1.124510 0.168620 1.035724
C 2.230468 -0.755022 1.264521
C 2.437743 -1.944441 0.535419
C 3.487013 -2.790173 0.903812
C 4.357196 -2.464763 1.935750
C 4.170016 -1.278111 2.633530
C 3.111679 -0.441708 2.305553
H -2.649111 0.306390 2.435170
H -0.360010 1.548537 3.689633
H -0.662194 -0.200185 3.466076
C 1.630590 -2.298437 -0.664720
H 3.637216 -3.717909 0.354663
H 5.176219 -3.134051 2.190148
H 4.842458 -1.002881 3.443862
H 2.959550 0.488037 2.850958
C -2.999606 2.421039 -0.841421
C -4.385210 1.442625 0.996494
C -3.409357 3.735496 1.254408
H -4.320897 4.165345 0.818186
H -3.535037 3.702709 2.345688
H -2.568365 4.399359 1.016703
H -5.274446 1.857132 0.504052
H -4.243903 0.416639 0.631458
H -4.597959 1.409523 2.074437
H -3.897711 2.870720 -1.285952
H -2.132792 3.037207 -1.107925

H -2.846932 1.428986 -1.283614
O 1.156596 -1.426493 -1.383038
C 1.480606 -3.740447 -1.047201
H 0.628686 -3.822464 -1.727144
H 2.379154 -4.095449 -1.570829
H 1.314879 -4.371593 -0.167532

TS_{ar}^{B}

59
Energy: -4118.80483169
Cu -0.080309 -0.711737 -0.646355
Cl 0.303824 -1.099617 -2.744919
C -1.904872 -1.375440 -0.815057
O 1.670845 0.165203 -0.361444
S 2.165370 1.317329 0.470770
O 3.314599 0.933186 1.296413
C 2.852186 2.402751 -0.836599
O 1.112658 2.089128 1.131612
F 3.392938 3.476021 -0.276286
F 3.778262 1.750086 -1.517510
F 1.886342 2.783428 -1.658308
C -2.172719 -2.645304 -0.320883
C -3.310477 -3.309463 -0.773631
C -4.163123 -2.698008 -1.686420
C -3.874826 -1.421461 -2.165134
C -2.737526 -0.749447 -1.736480
H -1.499488 -3.109971 0.395987
H -3.528029 -4.309157 -0.401225
H -5.056681 -3.217448 -2.028018
H -4.531546 -0.946641 -2.892096
H -2.493805 0.242248 -2.109544
C 1.874703 -2.318242 0.939147
O 0.838648 -2.680404 0.367973
C 3.235368 -2.795096 0.480056
N 1.811953 -1.484921 2.007879
C 0.539824 -0.915389 2.411861
C -0.387106 -0.577324 1.303723
C -1.469746 -0.177409 0.809058
C -2.623466 0.692073 0.944273
C -2.593596 2.025677 0.495593
C -3.677403 2.852039 0.791317
C -4.783593 2.364622 1.479398
C -4.814626 1.037470 1.893079
C -3.734280 0.203090 1.629476
H 2.636500 -0.919768 2.200811
C -0.193892 -1.835920 3.385691
H 0.773849 0.043669 2.902180
C -1.459421 2.508728 -0.352326
H -3.666355 3.889608 0.461643
H -5.625078 3.022444 1.687014
H -5.680146 0.649445 2.426318
H -3.738149 -0.838270 1.950983
C 4.392247 -2.415294 1.393658
H 3.130064 -3.892805 0.462550
C 3.490311 -2.329343 -0.955933
H 5.314761 -2.883505 1.029283
H 4.561116 -1.328266 1.387873
H 4.238193 -2.745132 2.429572
H 4.385191 -2.829821 -1.346845
H 2.642731 -2.555335 -1.613326
H 3.655832 -1.244436 -0.988102
H -1.139311 -1.385074 3.712426
H -0.407225 -2.796370 2.902504
H 0.435973 -2.017486 4.263741
O -0.872373 1.726190 -1.079968
C -1.088354 3.960046 -0.300253
H -0.162468 4.102705 -0.861947
H -1.875576 4.587674 -0.739699
H -0.942565 4.280377 0.738919

I_2

59
Energy: -4118.85298479
Cu 0.185877 -1.502638 -1.871159
Cl 0.940617 -3.514733 -2.372475
C 2.024931 0.329474 -0.606974
O -0.329734 -1.840169 -0.086115
S -1.838849 -2.128777 -0.068346
O -2.352958 -2.162016 -1.441697
C -1.884096 -3.859751 0.547234

O -2.545199 -1.331037 0.917384
F -3.154235 -4.221960 0.652307
F -1.262868 -4.661888 -0.287231
F -1.311514 -3.914084 1.737440
C 2.626406 -0.566061 -1.500539
C 3.739786 -1.308069 -1.115693
C 4.278963 -1.142009 0.152636
C 3.712867 -0.219703 1.032708
C 2.596072 0.513086 0.657111
H 2.278246 -0.626941 -2.532646
H 4.174414 -2.018552 -1.815661
H 5.146666 -1.723737 0.457529
H 4.141613 -0.079151 2.023389
H 2.145692 1.222669 1.350995
C -1.136986 -0.667219 -4.059903
O 0.007224 -1.045161 -3.683610
C -1.679836 -1.273329 -5.339420
N -1.880047 0.182356 -3.361040
C -1.539271 0.744084 -2.056467
C -0.202794 0.333006 -1.574610
C 0.771189 1.025139 -0.975451
C 0.663330 2.480598 -0.698051
C -0.432176 3.043857 -0.026098
C -0.545479 4.433984 0.062148
C 0.444589 5.260860 -0.449909
C 1.558410 4.702076 -1.072968
C 1.660223 3.324145 -1.201978
H -2.823490 0.354247 -3.685536
H -1.562000 1.843000 -2.140227
H -2.335490 0.458486 -1.350617
C -1.497388 2.240478 0.670101
H -1.418981 4.853107 0.559722
H 0.351506 6.341586 -0.359991
H 2.342155 5.342811 -1.472983
H 2.511632 2.880818 -1.718398
C -2.925816 -0.565404 -5.861425
C -0.580589 -1.247701 -6.402059
C -2.015040 -2.727181 -4.966351
H -2.404339 -3.244547 -5.853560
H -1.124746 -3.258456 -4.605570
H -2.775488 -2.770743 -4.174440
H -0.939686 -1.762272 -7.302713
H -0.312976 -0.219686 -6.682713
H 0.320469 -1.759070 -6.047422
H -3.224662 -1.025357 -6.811566
H -3.786667 -0.672794 -5.184664
H -2.749178 0.502035 -6.056194
O -2.668191 2.446823 0.413379
C -1.050858 1.287509 1.741192
H -1.923687 0.844663 2.226901
H -0.438928 0.474974 1.325689
H -0.431091 1.823979 2.474014

TS_{rc}^{B}

59
Energy: -4118.82330810
Cu 0.594250 0.544538 0.675027
Cl 1.935520 2.144125 1.396581
C -1.778936 1.229862 1.280999
O 1.102583 -1.293782 1.122004
S 2.068377 -1.838553 0.093767
O 2.315759 -0.850522 -0.967873
C 3.633417 -1.932907 1.042778
O 1.757194 -3.198974 -0.322637
F 4.593624 -2.386311 0.250149
F 3.960159 -0.736411 1.490390
F 3.481876 -2.761279 2.063989
C -2.127645 2.577356 1.139643
C -1.904836 3.467384 2.180115
C -1.344622 3.022611 3.377043
C -0.999019 1.687754 3.529336
C -1.200619 0.792348 2.481160
H -2.541380 2.923856 0.192332
H -2.161085 4.517928 2.056481
H -1.166258 3.726727 4.187110
H -0.543546 1.339829 4.453658
H -0.959952 -0.263605 2.613841
C 0.513407 1.349810 -2.124521
O -0.055604 1.600326 -1.008133
C 1.437719 2.358638 -2.748037
N 0.239920 0.172980 -2.650448

C -0.585106 -0.692571 -1.822719
C -0.846318 0.068586 -0.561676
C -1.965925 0.237698 0.173978
C -3.259593 -0.436358 -0.019641
C -3.420187 -1.732070 -0.556765
C -4.697048 -2.177286 -0.913218
C -5.824118 -1.408297 -0.666024
C -5.679334 -0.167012 -0.052474
C -4.416551 0.309470 0.262367
H 0.769245 -0.199618 -3.429139
H -1.534023 -0.913098 -2.336969
H -0.059989 -1.643492 -1.654140
C -2.341795 -2.769625 -0.689091
H -4.785061 -3.166019 -1.360637
H -6.811039 -1.782325 -0.931024
H -6.553900 0.440670 0.172914
H -4.313420 1.293519 0.715820
C 1.398828 2.256309 -4.271928
C 1.034024 3.760246 -2.290688
C 2.850123 2.033601 -2.227533
H 3.547372 2.758217 -2.669237
H 2.901004 2.115721 -1.133067
H 3.165364 1.020931 -2.508196
H 1.731462 4.486846 -2.726168
H 0.019320 4.019742 -2.621998
H 1.081181 3.845684 -1.198506
H 2.039700 3.040209 -4.693984
H 1.793061 1.298897 -4.641479
H 0.385569 2.398364 -4.671328
O -2.233308 -3.405514 -1.721401
C -1.527549 -3.092852 0.530790
H -0.560079 -3.522642 0.246993
H -1.370706 -2.235019 1.191643
H -2.087220 -3.849502 1.100506

Reaction 14

reactant

33
Energy: -1132.72069320
C -4.058284 -0.000405 0.187268
O -4.635153 -0.023106 -0.890646
C -4.830813 -0.097053 1.506986
N -2.701921 0.096826 0.250849
H -2.207174 0.137824 1.129803
C -1.932098 0.178967 -0.980597
C -0.505198 0.131388 -0.706576
H -2.234155 -0.646803 -1.642268
H -2.193037 1.101385 -1.523188
C 0.681299 0.092782 -0.465845
C 2.079228 0.040576 -0.188231
C 2.717312 -1.191096 0.016298
C 4.076162 -1.248930 0.287866
C 4.803585 -0.065412 0.354832
C 4.196521 1.169871 0.155292
C 2.836925 1.218003 -0.114890
H 2.132556 -2.107104 -0.041737
H 4.575120 -2.202153 0.446650
H 4.788294 2.080553 0.212259
H 2.345463 2.175836 -0.274251
C -3.958932 0.001323 2.754705
C -5.858421 1.035577 1.509207
C -5.554740 -1.445162 1.493288
Cl 6.514626 -0.131497 0.695726
H -6.509255 0.953430 2.390958
H -5.368025 2.018883 1.540520
H -6.478471 0.992407 0.606810
H -6.197162 -1.538410 2.380161
H -6.176934 -1.537142 0.595697
H -4.840299 -2.280693 1.502464
H -4.590375 -0.067069 3.650836
H -3.228076 -0.818487 2.815961
H -3.424156 0.960848 2.811257

I_c

68
Energy: -4733.34715040
Cu 0.705955 0.092502 -0.529073
Cl 1.337464 -0.485283 -2.538099
C 1.936468 1.534075 -0.722802

O -0.579985 -1.457858 -0.380472
S -1.418462 -1.883380 0.795442
O -2.144322 -0.780353 1.431136
C -2.723526 -2.840777 -0.066124
O -0.761908 -2.849512 1.668452
F -3.604468 -3.286251 0.812811
F -3.360871 -2.057073 -0.943173
F -2.196123 -3.861102 -0.718678
C 1.367167 2.760501 -0.441183
C 2.223610 3.860961 -0.333182
C 3.589220 3.718706 -0.534926
C 4.119905 2.469419 -0.847021
C 3.288966 1.354968 -0.948911
C -1.048861 2.995823 2.140575
O -0.166248 3.847480 2.124927
C -2.522609 3.397740 2.060366
N -0.765023 1.665785 2.252467
C 0.592175 1.235652 2.437800
C 1.092947 0.208715 1.501208
C 1.814466 -0.764351 1.239271
C 2.700503 -1.806041 0.875238
C 2.230295 -3.105209 0.613814
C 3.124087 -4.098959 0.254409
C 4.481235 -3.797645 0.166063
C 4.969965 -2.519904 0.432309
C 4.076471 -1.523047 0.782033
H 0.303343 2.890868 -0.263819
H 1.791076 4.822075 -0.060484
H 4.245112 4.583496 -0.453423
H 5.187171 2.352865 -1.030636
H 3.691898 0.383574 -1.228270
H -1.479023 0.949984 2.137450
H 1.218823 2.135615 2.343220
H 0.742268 0.832388 3.452061
H 1.165919 -3.311706 0.707904
H 2.778808 -5.108106 0.044065
Cl 5.600133 -5.050740 -0.280876
H 6.036360 -2.320712 0.361917
H 4.429296 -0.515787 0.997739
C -3.494876 2.231060 2.192130
C -2.767205 4.398014 3.192812
C -2.722083 4.093754 0.712319
O -3.288744 1.140698 -1.033544
C -4.506901 0.697930 -1.650841
C -5.532007 0.598881 -0.552401
C -2.091241 0.898423 -1.552371
O -1.121497 1.246718 -0.892710
C -2.002847 0.238758 -2.890198
H -4.793264 1.427812 -2.421635
H -4.353055 -0.275255 -2.131034
H -6.492129 0.256972 -0.955495
H -5.192161 -0.115329 0.208323
H -5.681163 1.572714 -0.071014
H -2.748479 0.632244 -3.589772
H -0.995911 0.369496 -3.291417
H -2.174167 -0.839006 -2.769229
H -3.376647 1.703017 3.149189
H -4.524584 2.615394 2.159089
H -3.385099 1.505960 1.375036
H -3.756399 4.458006 0.627672
H -2.041019 4.948187 0.615090
H -2.536113 3.398675 -0.116999
H -3.793395 4.786952 3.131439
H -2.645141 3.922743 4.176548
H -2.066079 5.237701 3.128772

I_1

54
Energy: -4425.82555218
Cu -0.016339 0.115048 0.003938
Cl -0.011369 0.320881 2.125579
C 1.847831 0.191033 0.316369
O -2.034437 0.315518 -0.296753
S -2.040021 1.691293 -0.923057
O -2.695598 1.784259 -2.213865
C -3.055956 2.667104 0.251188
O -0.670189 2.241751 -0.808672
F -3.090827 3.926485 -0.152750
F -4.284557 2.177215 0.278736
F -2.529127 2.606096 1.460148
C 2.613928 -0.950665 0.439458

C 3.998064 -0.789853 0.347557
C 4.556748 0.473767 0.170717
C 3.741471 1.597856 0.087862
C 2.354510 1.470405 0.184564
H 2.166049 -1.935927 0.575405
H 4.636459 -1.668964 0.422561
H 5.638211 0.584225 0.113432
H 4.175863 2.588874 -0.034093
H 1.702966 2.342542 0.154218
C -0.443677 -3.206158 0.704159
O 0.623257 -3.540323 0.201004
C -0.814559 -3.578185 2.137355
N -1.377596 -2.544555 -0.044464
C -1.089195 -2.239512 -1.425571
C -0.121697 -1.141912 -1.636022
C 0.603466 -0.349057 -2.236682
C 1.533236 0.507905 -2.878800
C 1.152377 1.789515 -3.307449
C 2.086077 2.621934 -3.902608
C 3.391712 2.166960 -4.071394
C 3.785849 0.894545 -3.664808
C 2.852311 0.065610 -3.066603
H -2.167997 -2.082135 0.386795
H -0.678615 -3.136890 -1.905875
H -2.026687 -1.967339 -1.923706
H 0.130157 2.125035 -3.144995
H 1.812122 3.620311 -4.234276
Cl 4.565736 3.218099 -4.810916
H 4.813238 0.570322 -3.811601
H 3.136093 -0.926224 -2.719415
C -2.038081 -2.836727 2.669027
C -1.098515 -5.085104 2.112899
C 0.395595 -3.299343 3.028121
H 0.190998 -3.644012 4.051020
H 1.281037 -3.825708 2.653111
H 0.620377 -2.224497 3.067973
H -1.331979 -5.435590 3.127776
H -1.956917 -5.319224 1.466968
H -0.226745 -5.639465 1.744159
H -2.205979 -3.117704 3.717198
H -1.903537 -1.745768 2.640396
H -2.954004 -3.102094 2.120648

TS_{rc}^{A}

54
Energy: -4425.80260498
Cu 0.050068 -0.770682 -0.807327
Cl 0.703774 -1.447063 -2.724638
C -1.137037 -2.226588 -0.833366
O 0.908628 1.003115 -0.928884
S 1.569458 1.977917 0.008303
O 2.970895 1.654687 0.280884
C 1.596377 3.472598 -1.052911
O 0.753569 2.351498 1.164453
F 2.147685 4.470582 -0.378957
F 2.292494 3.249460 -2.152384
F 0.353170 3.803992 -1.378107
C -0.830972 -3.408225 -0.182962
C -1.809962 -4.400864 -0.130027
C -3.049517 -4.202688 -0.731483
C -3.318478 -3.011627 -1.397970
C -2.352075 -2.008382 -1.460752
H 0.139681 -3.553185 0.287182
H -1.594797 -5.334688 0.387812
H -3.805470 -4.984855 -0.689198
H -4.280854 -2.856545 -1.883316
H -2.556256 -1.078632 -1.992119
C 2.647219 -1.155368 1.309360
O 1.627336 -1.672239 0.806032
C 4.030114 -1.402477 0.751227
N 2.458569 -0.453787 2.442112
C 1.080402 -0.370292 2.897879
C 0.161989 -0.556832 1.764959
C -0.749287 -0.317144 0.930632
C -2.080222 0.278841 0.900063
C -2.292649 1.560492 0.385123
C -3.579739 2.079306 0.328525
C -4.647531 1.310729 0.776945
C -4.454044 0.029204 1.279814
C -3.166805 -0.486700 1.335143
H 3.132627 0.247432 2.724523

H 0.898283 -1.119437 3.682125
H 0.889182 0.633550 3.300926
H -1.446463 2.154309 0.048147
H -3.755617 3.078614 -0.062600
Cl -6.265657 1.963373 0.699608
H -5.304784 -0.557931 1.617869
H -2.997690 -1.500667 1.696766
C 3.994391 -1.231825 -0.768317
C 5.080865 -0.485504 1.369515
C 4.348525 -2.864501 1.103467
H 5.343549 -3.116370 0.713787
H 4.358914 -3.025867 2.190931
H 3.619364 -3.548661 0.652779
H 6.057378 -0.715179 0.924617
H 4.860534 0.569479 1.164246
H 5.180958 -0.642243 2.454056
H 4.983426 -1.480522 -1.175909
H 3.252923 -1.893740 -1.232493
H 3.750896 -0.198233 -1.043809

TS_{ar}^{B}

54
Energy: -4425.81484111
Cu 0.380600 -0.801718 -0.485271
Cl 0.904099 -1.251802 -2.538451
C -1.268407 -1.822814 -0.636655
O 1.906083 0.438605 -0.308614
S 1.828824 1.903367 0.053532
O 2.423436 2.172802 1.366220
C 3.012794 2.585779 -1.168789
O 0.533768 2.506442 -0.243542
F 3.135747 3.889769 -0.978722
F 4.195634 2.007096 -1.004445
F 2.576668 2.355853 -2.391891
C -1.304431 -3.018528 0.067675
C -2.204028 -4.003379 -0.338681
C -3.057126 -3.772821 -1.412052
C -3.013438 -2.557551 -2.094806
C -2.113906 -1.571740 -1.712526
H -0.634120 -3.185399 0.907594
H -2.236260 -4.950282 0.197300
H -3.763759 -4.541694 -1.719038
H -3.672742 -2.378268 -2.942007
H -2.069729 -0.622829 -2.243714
C 2.445201 -1.537013 1.420482
O 1.574319 -2.247488 0.897667
C 3.913772 -1.732507 1.112066
N 2.099181 -0.574897 2.311297
C 0.699103 -0.271739 2.538868
C -0.167153 -0.318786 1.328006
C -1.284785 -0.249335 0.752968
C -2.571887 0.384883 0.581188
C -2.607365 1.757055 0.306614
C -3.831454 2.402687 0.187359
C -5.002634 1.671224 0.348474
C -4.981016 0.303548 0.617245
C -3.760314 -0.341233 0.722114
H 2.722657 0.225513 2.386471
C 0.100542 -1.187544 3.604432
H 0.673126 0.773524 2.884722
H -1.670186 2.299594 0.180005
H -3.879741 3.467041 -0.029624
Cl -6.539342 2.479114 0.205529
H -5.914847 -0.240689 0.735381
H -3.717619 -1.412820 0.913527
C 4.829559 -0.648688 1.665384
H 4.160648 -2.690433 1.603647
C 4.109711 -1.910089 -0.392472
H 5.870296 -0.883435 1.412141
H 4.602499 0.330826 1.218823
H 4.773068 -0.560774 2.758446
H 5.146129 -2.204825 -0.598087
H 3.438908 -2.673699 -0.799204
H 3.903631 -0.969964 -0.919662
H -0.946500 -0.928417 3.800138
H 0.151986 -2.231361 3.275212
H 0.672876 -1.085577 4.533152

I_2

54

Energy: -4425.86712906
Cu 0.262677 -1.440855 -1.973966
Cl 0.890949 -3.505323 -2.424095
C 2.080296 0.388232 -0.710623
O -0.127589 -1.728797 -0.158142
S -1.642300 -1.652652 0.055569
O -2.351564 -1.870489 -1.208960
C -1.909865 -3.159887 1.069158
O -2.045873 -0.517549 0.873071
F -3.202223 -3.247529 1.345981
F -1.527627 -4.225805 0.398718
F -1.224334 -3.064327 2.194046
C 2.695639 -0.466949 -1.635361
C 3.800827 -1.228778 -1.265660
C 4.313124 -1.127342 0.020703
C 3.727505 -0.251713 0.935179
C 2.620200 0.502353 0.574603
H 2.361334 -0.478440 -2.674297
H 4.251838 -1.903902 -1.989892
H 5.173331 -1.725830 0.314243
H 4.130870 -0.168539 1.942843
H 2.142564 1.161797 1.298328
C -1.103000 -0.683094 -4.159504
O 0.044317 -1.056665 -3.791819
C -1.699452 -1.362873 -5.376534
N -1.817253 0.214642 -3.490361
C -1.441682 0.840229 -2.223930
C -0.119698 0.402184 -1.718771
C 0.825898 1.084571 -1.067020
C 0.633149 2.487566 -0.634696
C -0.520512 2.860391 0.063030
C -0.703795 4.174713 0.477160
C 0.277458 5.115790 0.190212
C 1.443423 4.765019 -0.484396
C 1.618830 3.448526 -0.885600
H -2.762849 0.385457 -3.808871
H -1.403974 1.931124 -2.377401
H -2.246499 0.625511 -1.505363
H -1.257208 2.099431 0.324460
H -1.592593 4.467606 1.031370
Cl 0.051965 6.770214 0.699978
H 2.200844 5.519012 -0.686468
H 2.529870 3.156240 -1.407184
C -2.868860 -0.594962 -5.984866
C -0.604360 -1.567236 -6.422788
C -2.176147 -2.727744 -4.848173
H -2.610599 -3.301316 -5.678031
H -1.341965 -3.299793 -4.420226
H -2.940735 -2.610871 -4.067277
H -1.016417 -2.134174 -7.267479
H -0.228172 -0.609496 -6.807570
H 0.237169 -2.129743 -6.004552
H -3.200254 -1.112511 -6.893700
H -3.745027 -0.557132 -5.320160
H -2.590522 0.428836 -6.271709

TS_{rc}^{B}

54
Energy: -4425.83957715
Cu -1.690788 0.533004 0.024614
Cl -3.506120 0.441924 -1.219364
C 0.769008 -0.651936 2.114352
O -0.804199 2.052102 0.749814
S 0.225364 2.691090 -0.171025
O 0.345683 1.968872 -1.439558
C -0.621923 4.259566 -0.597802
O 1.446177 3.057957 0.530224
F 0.142545 4.958164 -1.422969
F -1.774252 3.990973 -1.190586
F -0.845499 4.966830 0.497267
C -0.408200 -0.266100 2.773145
C -0.467380 -0.218217 4.155046
C 0.650709 -0.552484 4.915662
C 1.827544 -0.932176 4.279014
C 1.888524 -0.980374 2.893062
H -1.278358 0.021942 2.190819
H -1.388728 0.096000 4.641115
H 0.605437 -0.507632 6.002250
H 2.708271 -1.189561 4.864368
H 2.814162 -1.280599 2.405675
C -2.072196 -2.253711 -0.637428

O -1.807217 -1.595205 0.405063
C -3.163448 -3.299288 -0.625300
N -1.291168 -2.080585 -1.702675
C -0.319211 -0.994955 -1.646107
C -0.168225 -0.623037 -0.208536
C 0.849110 -0.749000 0.654742
C 2.188524 -0.929689 0.017756
C 3.054820 0.155595 -0.131257
C 4.307477 -0.030166 -0.704445
C 4.683398 -1.300991 -1.125148
C 3.832830 -2.392999 -0.986997
C 2.581496 -2.198724 -0.415054
H -1.585201 -2.435239 -2.604044
H 0.652243 -1.335095 -2.028428
H -0.653885 -0.148522 -2.263138
H 2.740463 1.149727 0.192353
H 4.988373 0.808063 -0.832123
Cl 6.255706 -1.535039 -1.846152
H 4.153253 -3.378159 -1.318385
H 1.901512 -3.043072 -0.290984
C -4.287781 -2.832544 0.298043
C -3.711345 -3.543967 -2.029643
C -2.521627 -4.579301 -0.070915
H -4.552383 -4.245709 -1.968185
H -4.083084 -2.613934 -2.481122
H -2.970372 -4.006185 -2.699045
H -3.283685 -5.367461 -0.011974
H -1.708479 -4.937847 -0.717198
H -2.120391 -4.414903 0.937313
H -5.077315 -3.594935 0.314126
H -3.926586 -2.687068 1.322345
H -4.714321 -1.883861 -0.051524

Reaction 15

reactant

33
Energy: -1132.72020290
C -4.056720 0.003737 0.187438
O -4.631791 -0.032215 -0.890979
C -4.828546 -0.107001 1.506445
N -2.702492 0.129143 0.252716
H -2.211221 0.188311 1.132603
C -1.933101 0.230140 -0.977242
C -0.506415 0.166169 -0.704981
H -2.242921 -0.581461 -1.652391
H -2.185400 1.164374 -1.503741
C 0.679564 0.115731 -0.465241
C 2.077542 0.046368 -0.188501
C 2.695011 -1.190210 0.045425
C 4.055143 -1.250650 0.315232
C 4.822010 -0.090309 0.356356
C 4.201311 1.131828 0.122451
C 2.844220 1.218832 -0.148047
H 2.093612 -2.096157 0.010512
H 4.530030 -2.213070 0.495702
Cl 5.153661 2.596657 0.170569
H 2.377562 2.184412 -0.328652
C -3.959073 0.001602 2.755003
C -5.872464 1.010687 1.509952
C -5.532885 -1.465373 1.489751
H 5.888578 -0.125201 0.565904
H -6.521896 0.918124 2.391699
H -5.396425 2.000991 1.542293
H -6.492068 0.959428 0.607649
H -6.175098 -1.569055 2.375603
H -6.152401 -1.565043 0.591162
H -4.806498 -2.290516 1.498789
H -4.589769 -0.080814 3.650467
H -3.214302 -0.805762 2.813365
H -3.440966 0.970006 2.815841

I_c

68
Energy: -4733.34480228
Cu 0.004221 0.005282 0.002744
Cl -0.001425 0.007739 2.185445
C -0.569654 1.814210 0.183949
O 0.620423 -1.913965 -0.117531
S 0.751922 -2.781019 -1.342600

O 1.461401 -2.130961 -2.448156
C 1.938136 -4.037854 -0.727785
O -0.456516 -3.530140 -1.664337
F 2.174819 -4.927151 -1.675935
F 3.095380 -3.450488 -0.399548
F 1.458572 -4.647436 0.340779
C 0.219972 2.694000 -0.530075
C -0.245690 4.005232 -0.674984
C -1.440774 4.402244 -0.092146
C -2.190927 3.491033 0.647265
C -1.753829 2.176594 0.799040
C 1.489534 1.654217 -3.699352
O 0.997709 2.775302 -3.624701
C 2.932263 1.464455 -4.171080
N 0.775405 0.532026 -3.394987
C -0.615475 0.638307 -3.055572
C -1.040873 -0.009980 -1.799738
C -1.860086 -0.601179 -1.085474
C -2.837817 -1.156949 -0.218505
C -2.715915 -2.467191 0.271926
C -3.700118 -2.944467 1.121463
C -4.795664 -2.163587 1.482919
C -4.914446 -0.871581 0.979951
C -3.941051 -0.359583 0.135980
H 1.149763 2.399003 -1.008262
H 0.342050 4.695078 -1.277926
H -1.789307 5.426826 -0.208180
H -3.118012 3.799944 1.128335
H -2.316516 1.468939 1.404539
H 1.209878 -0.386117 -3.335425
H -0.838131 1.714546 -2.990769
H -1.249506 0.216305 -3.851944
H -1.871448 -3.085497 -0.026405
Cl -3.572795 -4.563827 1.750507
H -5.548701 -2.574051 2.152129
H -5.773571 -0.263579 1.254557
H -4.019683 0.648669 -0.266468
C 3.362552 0.006866 -4.288141
C 3.038140 2.140639 -5.540239
C 3.829607 2.194511 -3.169478
O 4.007734 -0.476755 -0.990546
C 5.196962 -1.216579 -0.677098
C 5.727366 -1.747903 -1.981782
C 3.074748 -0.202098 -0.086812
O 2.096475 0.421838 -0.475636
C 3.281675 -0.656128 1.321385
H 5.910640 -0.539717 -0.185532
H 4.959630 -2.034410 0.012662
H 6.648118 -2.318616 -1.815784
H 4.982075 -2.406319 -2.445132
H 5.943707 -0.926419 -2.675603
H 4.299317 -0.443079 1.668090
H 2.549880 -0.171930 1.970811
H 3.118715 -1.740491 1.376815
H 2.738285 -0.551212 -5.000555
H 4.396176 -0.035271 -4.661111
H 3.338659 -0.509195 -3.319499
H 4.878620 2.138878 -3.495890
H 3.542621 3.250466 -3.093010
H 3.756752 1.736447 -2.174215
H 4.078757 2.108272 -5.892643
H 2.413696 1.628332 -6.286014
H 2.715999 3.186524 -5.483901

I_1

54
Energy: -4425.82329985
Cu -0.020028 0.128684 0.000092
Cl -0.006571 0.315254 2.123214
C 1.843307 0.208953 0.320089
O -2.041973 0.316129 -0.287232
S -2.053221 1.691979 -0.914254
O -2.716131 1.783494 -2.200790
C -3.060832 2.667847 0.267160
O -0.681581 2.240860 -0.806631
F -3.099949 3.926771 -0.136085
F -4.288587 2.176292 0.304393
F -2.524316 2.606584 1.472111
C 2.609308 -0.933533 0.436773
C 3.992894 -0.772898 0.339724
C 4.550866 0.491336 0.164985

C 3.735644 1.615972 0.089758
C 2.349098 1.488896 0.192071
H 2.161286 -1.918858 0.571873
H 4.631415 -1.652338 0.409443
H 5.632187 0.601677 0.104370
H 4.169418 2.607494 -0.029848
H 1.697769 2.361361 0.167670
C -0.445963 -3.201111 0.697626
O 0.619812 -3.529974 0.188522
C -0.809931 -3.579503 2.130902
N -1.385826 -2.540967 -0.044839
C -1.105846 -2.233285 -1.426949
C -0.135624 -1.140224 -1.641791
C 0.591992 -0.336492 -2.221913
C 1.535956 0.510960 -2.864214
C 1.163627 1.795328 -3.287844
C 2.122080 2.593118 -3.892400
C 3.426233 2.143447 -4.085299
C 3.780602 0.863904 -3.668928
C 2.843749 0.043143 -3.059543
H -2.172915 -2.077801 0.391748
H -0.701745 -3.130489 -1.913276
H -2.045492 -1.956059 -1.918140
H 0.151119 2.156855 -3.120519
Cl 1.689604 4.195285 -4.424052
H 4.152955 2.797971 -4.561789
H 4.798853 0.511367 -3.818687
H 3.108952 -0.953400 -2.712371
C -2.031394 -2.840904 2.671240
C -1.093920 -5.086373 2.100496
C 0.404042 -3.304912 3.017661
H 0.204121 -3.654939 4.039658
H 1.287967 -3.829050 2.636001
H 0.628755 -2.230241 3.062093
H -1.323366 -5.441580 3.114647
H -1.954883 -5.317445 1.456899
H -0.223610 -5.639021 1.725748
H -2.193907 -3.125597 3.719249
H -1.898007 -1.749770 2.645520
H -2.949752 -3.105059 2.126419

TS_{rc}^{A}

54
Energy: -4425.80166544
Cu 0.032219 -0.879885 0.770410
Cl -0.586801 -1.450544 2.732402
C 1.035446 -2.467630 0.758115
O -0.611160 0.981329 0.901686
S -1.210679 2.023506 -0.003663
O -2.661995 1.898054 -0.143623
C -0.922261 3.526652 1.006121
O -0.459953 2.253553 -1.239548
F -1.393816 4.579803 0.356333
F -1.530522 3.424342 2.173644
F 0.381030 3.682044 1.198741
C 0.552302 -3.607307 0.141030
C 1.396200 -4.714601 0.053344
C 2.679941 -4.667916 0.589534
C 3.128280 -3.514386 1.224673
C 2.298787 -2.397531 1.320842
H -0.451650 -3.634429 -0.277625
H 1.040359 -5.618321 -0.439727
H 3.329600 -5.538734 0.521142
H 4.125560 -3.477186 1.660442
H 2.642522 -1.496840 1.829570
C -2.716196 -0.962502 -1.179973
O -1.739182 -1.602457 -0.736381
C -4.083372 -1.040542 -0.539708
N -2.511609 -0.288185 -2.326685
C -1.167124 -0.387150 -2.871861
C -0.208535 -0.679401 -1.796676
C 0.770433 -0.540812 -1.017759
C 2.155268 -0.081693 -1.076429
C 2.499377 1.204507 -0.653848
C 3.835473 1.579418 -0.693397
C 4.830150 0.712915 -1.132527
C 4.470015 -0.566905 -1.540867
C 3.141909 -0.972616 -1.510336
H -3.105263 0.495706 -2.570290
H -1.135414 -1.159589 -3.653868
H -0.875739 0.581129 -3.300863

H 1.735071 1.903842 -0.324398
Cl 4.271987 3.187999 -0.174025
H 5.866924 1.040705 -1.148695
H 5.237551 -1.258394 -1.883087
H 2.858934 -1.982428 -1.804943
C -3.939498 -0.875426 0.973583
C -5.052630 -0.005737 -1.102321
C -4.593393 -2.454427 -0.863108
H -5.588141 -2.584699 -0.417187
H -4.683705 -2.613979 -1.947156
H -3.927406 -3.220810 -0.448501
H -6.022532 -0.120808 -0.601961
H -4.699644 1.015666 -0.914129
H -5.231316 -0.147266 -2.178769
H -4.924368 -1.011902 1.440020
H -3.250217 -1.615922 1.397470
H -3.566863 0.124391 1.227558

TS_{ar}^{B}

54
Energy: -4425.81295121
Cu 0.220606 -0.867856 -0.510642
Cl 0.684178 -1.239090 -2.593184
C -1.435615 -1.872613 -0.664005
O 1.761502 0.343728 -0.325095
S 1.698574 1.796321 0.088422
O 2.341029 2.018311 1.387321
C 2.838721 2.516474 -1.153663
O 0.393513 2.405885 -0.141544
F 2.964763 3.815001 -0.933084
F 4.027759 1.936860 -1.044484
F 2.362258 2.316851 -2.366843
C -1.459229 -3.098840 -0.014260
C -2.381329 -4.056198 -0.436038
C -3.266950 -3.768646 -1.468628
C -3.234247 -2.523303 -2.095330
C -2.313005 -1.563772 -1.698185
H -0.763133 -3.309592 0.794423
H -2.404846 -5.027115 0.055650
H -3.990327 -4.516840 -1.787524
H -3.919398 -2.299328 -2.910800
H -2.276311 -0.591654 -2.186356
C 2.345905 -1.690120 1.298261
O 1.454524 -2.377223 0.779161
C 3.802454 -1.880921 0.934973
N 2.034660 -0.762656 2.238532
C 0.643581 -0.469866 2.525250
C -0.263700 -0.462283 1.343973
C -1.394863 -0.363453 0.803559
C -2.693244 0.269424 0.709038
C -2.737177 1.649325 0.483047
C -3.979477 2.270853 0.452115
C -5.155677 1.553908 0.642509
C -5.091183 0.179802 0.861826
C -3.867144 -0.470618 0.886208
H 2.656834 0.039195 2.314547
C 0.078006 -1.433508 3.566480
H 0.629845 0.558468 2.919085
H -1.814323 2.209618 0.330270
Cl -4.064868 3.988294 0.170717
H -6.111701 2.072057 0.614925
H -6.009812 -0.383922 1.009847
H -3.805546 -1.547091 1.039086
C 4.743743 -0.822921 1.495315
H 4.057843 -2.856477 1.386135
C 3.951235 -2.009317 -0.579683
H 5.774448 -1.059156 1.204978
H 4.514058 0.171984 1.085895
H 4.719169 -0.768676 2.591685
H 4.974767 -2.317864 -0.825743
H 3.252659 -2.743719 -0.993317
H 3.751986 -1.047058 -1.067548
H -0.961726 -1.183407 3.807717
H 0.117105 -2.460945 3.187790
H 0.680378 -1.374159 4.479735

I_2

54
Energy: -4425.86644656
Cu 0.265444 -1.435339 -1.966568

Cl 0.922466 -3.484385 -2.445864
C 2.064380 0.389724 -0.690127
O -0.118481 -1.749759 -0.154760
S -1.635809 -1.703888 0.055303
O -2.334702 -1.912373 -1.216814
C -1.880170 -3.233751 1.040776
O -2.061075 -0.590474 0.889878
F -3.171355 -3.348134 1.312929
F -1.478702 -4.281265 0.352518
F -1.198528 -3.146919 2.168734
C 2.689143 -0.451352 -1.620913
C 3.792426 -1.216467 -1.251208
C 4.291775 -1.132516 0.041273
C 3.695790 -0.270882 0.962557
C 2.591112 0.486729 0.601873
H 2.362741 -0.450599 -2.662435
H 4.250879 -1.881422 -1.980137
H 5.149599 -1.734358 0.334926
H 4.088812 -0.201994 1.975372
H 2.104945 1.135141 1.329916
C -1.115880 -0.672843 -4.139300
O 0.038959 -1.031709 -3.780172
C -1.707246 -1.348218 -5.361251
N -1.839872 0.207627 -3.457931
C -1.464104 0.829598 -2.190257
C -0.136757 0.401309 -1.691491
C 0.810427 1.088264 -1.047449
C 0.637134 2.501405 -0.637554
C -0.528552 2.906170 0.019972
C -0.670816 4.238020 0.390786
C 0.321624 5.175170 0.131213
C 1.487835 4.759515 -0.505578
C 1.651719 3.433312 -0.882287
H -2.790328 0.366707 -3.768005
H -1.438865 1.921811 -2.337915
H -2.262505 0.603529 -1.467773
H -1.295053 2.177712 0.284725
Cl -2.126526 4.741472 1.213900
H 0.182568 6.210281 0.434616
H 2.275602 5.483073 -0.706683
H 2.566535 3.107276 -1.375556
C -2.890320 -0.590793 -5.956191
C -0.613133 -1.523735 -6.413840
C -2.161506 -2.726379 -4.848157
H -2.588065 -3.296766 -5.684309
H -1.317795 -3.290430 -4.428336
H -2.926851 -2.630845 -4.065197
H -1.020128 -2.086149 -7.263995
H -0.252200 -0.555978 -6.788068
H 0.237928 -2.079134 -6.005485
H -3.217822 -1.102880 -6.869527
H -3.764119 -0.573414 -5.287556
H -2.628330 0.440227 -6.232373

TS_{rc}^{B}

54
Energy: -4425.83793566
Cu -1.212971 1.158649 0.211006
Cl -2.883007 2.178797 -0.798247
C 0.388417 -1.410852 1.841467
O 0.363818 1.889910 0.985012
S 1.514341 2.061785 0.002603
O 1.149017 1.614212 -1.343476
C 1.594865 3.889644 -0.110590
O 2.786106 1.621840 0.554423
F 2.535584 4.233398 -0.976077
F 0.427550 4.357313 -0.523767
F 1.879461 4.403926 1.074237
C -0.385453 -0.590838 2.676756
C -0.347723 -0.738829 4.052287
C 0.466981 -1.709800 4.629870
C 1.244288 -2.528198 3.817115
C 1.208074 -2.380723 2.437378
H -1.005057 0.186822 2.240050
H -0.948168 -0.081942 4.678265
H 0.501529 -1.820701 5.712120
H 1.888700 -3.285747 4.259160
H 1.819423 -3.027876 1.811354
C -3.000086 -0.925176 -0.699814
O -2.384447 -0.659532 0.368224
C -4.471338 -1.269968 -0.669328

N -2.294115 -1.002224 -1.827172
C -0.903069 -0.568783 -1.771442
C -0.512068 -0.551014 -0.331579
C 0.337461 -1.303286 0.381210
C 1.354817 -2.041994 -0.428336
C 2.651591 -1.540659 -0.551502
C 3.578423 -2.272600 -1.285006
C 3.242982 -3.477679 -1.894309
C 1.945969 -3.962391 -1.765653
C 1.000598 -3.248885 -1.037089
H -2.772931 -1.015314 -2.718970
H -0.260819 -1.283758 -2.302763
H -0.789110 0.415908 -2.247041
H 2.927265 -0.588927 -0.092334
Cl 5.203125 -1.664086 -1.452217
H 3.993776 -4.022986 -2.461677
H 1.675172 -4.907243 -2.233253
H -0.016239 -3.627336 -0.923922
C -5.149302 -0.449936 0.427410
C -5.134119 -0.987117 -2.015796
C -4.552140 -2.766745 -0.336854
H -6.212081 -1.172063 -1.929905
H -4.996848 0.060648 -2.316459
H -4.768689 -1.649001 -2.815303
H -5.606851 -3.064508 -0.271043
H -4.071430 -3.380518 -1.111251
H -4.074306 -2.983097 0.627187
H -6.216678 -0.704409 0.456787
H -4.714816 -0.665493 1.410082
H -5.042671 0.625461 0.236019

Reaction 16

reactant

33
Energy: -1132.72038488
C -4.041811 -0.371909 0.229976
O -4.679572 -0.822021 -0.711501
C -4.737446 0.138068 1.496349
N -2.683576 -0.309040 0.186758
H -2.135153 0.060375 0.950468
C -1.977126 -0.781184 -0.992498
C -0.547193 -0.607995 -0.802863
H -2.230312 -1.836872 -1.177633
H -2.330936 -0.233195 -1.879875
C 0.622320 -0.423330 -0.552252
C 1.991166 -0.198692 -0.241170
C 3.002327 -0.559668 -1.144397
C 4.338877 -0.342582 -0.845954
C 4.695868 0.242031 0.366083
C 3.714283 0.609279 1.279573
C 2.378484 0.388935 0.973926
H 2.709782 -1.015702 -2.088193
H 5.105901 -0.630659 -1.561820
H 5.743131 0.414835 0.605929
H 3.974839 1.066922 2.231214
C -3.786617 0.668166 2.565398
C -5.691420 1.251859 1.061170
C -5.542397 -1.033470 2.061898
H -4.365073 1.011435 3.433796
H -3.095200 -0.107107 2.927240
H -3.200988 1.528761 2.209806
H -6.138144 -0.701460 2.923772
H -6.218437 -1.437628 1.299847
H -4.881588 -1.844777 2.398859
H -6.288378 1.594230 1.918135
H -5.139979 2.116437 0.665162
H -6.369788 0.891964 0.279291
Cl 1.163800 0.859062 2.138295

I_c

68
Energy: -4733.34111095
Cu 0.533499 -0.107463 -0.534883
Cl 1.199195 -0.759495 -2.517948
C 1.779492 1.339393 -0.673887
O -0.878952 -1.543252 -0.421237
S -1.905594 -1.775562 0.651559
O -2.930348 -0.721568 0.698728
C -2.782906 -3.231446 -0.039016

O -1.360489 -2.216321 1.929186
F -3.730842 -3.609002 0.802554
F -3.349949 -2.914335 -1.203442
F -1.942401 -4.234085 -0.230079
C 1.235540 2.494038 -0.145424
C 2.068100 3.608800 -0.024979
C 3.391776 3.549883 -0.441120
C 3.896893 2.372244 -0.982090
C 3.087413 1.241056 -1.105018
C -1.035883 2.599415 2.416411
O -0.018268 3.174274 2.782515
C -2.327335 3.374061 2.143231
N -1.056951 1.247713 2.202537
C 0.073400 0.443608 2.578359
C 0.879031 -0.180267 1.496254
C 1.803520 -0.931804 1.153179
C 2.936059 -1.686471 0.768563
C 2.844270 -3.029368 0.346256
C 3.988466 -3.729014 -0.012998
C 5.229904 -3.107207 0.055809
C 5.344918 -1.785634 0.484398
C 4.206495 -1.081098 0.834556
H 0.204978 2.552705 0.189484
H 1.659316 4.511498 0.426706
H 4.032763 4.424416 -0.345458
H 4.927194 2.321779 -1.332226
H 3.475003 0.328605 -1.550665
H -1.899040 0.775254 1.880918
H 0.744866 1.101458 3.147196
H -0.255704 -0.385680 3.219516
Cl 1.315202 -3.820376 0.278992
H 3.896322 -4.760970 -0.342604
H 6.119824 -3.666626 -0.226763
H 6.320753 -1.309411 0.543035
H 4.265759 -0.046181 1.166435
C -3.565247 2.488671 2.051752
C -2.502178 4.388458 3.272810
C -2.124899 4.114178 0.816483
O -3.338862 1.436915 -1.223430
C -4.599429 0.917067 -1.673616
C -5.663488 1.489816 -0.775797
C -2.200468 0.868646 -1.605185
O -1.186132 1.183832 -0.995665
C -2.223394 -0.072325 -2.765063
H -4.756726 1.207929 -2.722470
H -4.576723 -0.179031 -1.607152
H -6.656760 1.196019 -1.134334
H -5.539087 1.119353 0.248141
H -5.607478 2.585329 -0.759763
H -2.888156 0.286295 -3.558683
H -1.208247 -0.200239 -3.147894
H -2.577991 -1.056117 -2.435368
H -3.720104 1.904613 2.970160
H -4.452878 3.121680 1.908581
H -3.518700 1.800089 1.197915
H -3.003289 4.739876 0.601223
H -1.242149 4.766349 0.868117
H -1.999650 3.408304 -0.016088
H -3.371531 5.028197 3.066222
H -2.672130 3.887303 4.236018
H -1.612370 5.019341 3.371925

I_1

54
Energy: -4425.81924754
Cu 0.012668 0.132749 0.007693
Cl 0.011221 0.249883 2.141780
C 1.872005 0.159964 0.350517
O -1.992183 0.326000 -0.232600
S -2.046467 1.735716 -0.787499
O -2.916924 1.912925 -1.931304
C -2.836020 2.688401 0.569524
O -0.651492 2.228005 -0.852619
F -2.966203 3.948819 0.186651
F -4.030854 2.179081 0.822798
F -2.096688 2.638141 1.662533
C 2.613998 -1.001179 0.460299
C 4.003576 -0.866832 0.460927
C 4.596024 0.392755 0.388061
C 3.807017 1.535524 0.311775
C 2.414376 1.431453 0.310759

H 2.143304 -1.983607 0.523646
H 4.619723 -1.762149 0.529026
H 5.680766 0.482841 0.409259
H 4.265293 2.522410 0.270842
H 1.782946 2.318777 0.288479
C -0.437324 -3.224485 0.690006
O 0.633188 -3.598206 0.223140
C -0.876717 -3.600097 2.102961
N -1.314760 -2.514312 -0.081434
C -0.980344 -2.221750 -1.453273
C -0.058491 -1.080762 -1.660094
C 0.649658 -0.310341 -2.309553
C 1.519069 0.565152 -3.002194
C 1.033956 1.541380 -3.895917
C 1.915873 2.386489 -4.555872
C 3.283579 2.268048 -4.333421
C 3.782771 1.299736 -3.465056
C 2.906942 0.449581 -2.812456
H -2.109193 -2.036012 0.324931
H -0.512028 -3.112237 -1.891136
H -1.905454 -2.001516 -1.999010
Cl -0.658627 1.678156 -4.206374
H 1.521645 3.133037 -5.241029
H 3.965769 2.939150 -4.852181
H 4.853455 1.207490 -3.296024
H 3.274252 -0.315041 -2.129769
C -2.099917 -2.831050 2.594792
C -1.201539 -5.098043 2.048528
C 0.304294 -3.366227 3.044468
H 0.051900 -3.723178 4.052428
H 1.190588 -3.907451 2.693545
H 0.553000 -2.298073 3.111843
H -1.486341 -5.453007 3.048651
H -2.039211 -5.300147 1.365544
H -0.331492 -5.672842 1.708285
H -2.317491 -3.120361 3.631567
H -1.935234 -1.743917 2.585867
H -2.999709 -3.064122 2.006528

TS_{rc}^{A}

54
Energy: -4425.80093196
Cu 0.432627 0.575017 -0.658570
Cl 0.013734 1.137209 -2.681670
C 1.953927 1.693707 -0.792849
O -1.315594 -0.379273 -0.391419
S -1.732565 -1.679581 0.252710
O -2.874091 -1.496112 1.159352
C -2.431649 -2.555719 -1.196410
O -0.629345 -2.492127 0.747000
F -2.867093 -3.750293 -0.828130
F -3.441280 -1.854787 -1.691139
F -1.499842 -2.696802 -2.126128
C 1.836956 2.985465 -0.306337
C 2.962268 3.808853 -0.343545
C 4.163247 3.343456 -0.867389
C 4.243254 2.048942 -1.367671
C 3.128399 1.211528 -1.344309
H 0.897790 3.351123 0.100218
H 2.887562 4.825193 0.041076
H 5.035143 3.994645 -0.895960
H 5.174076 1.680177 -1.796150
H 3.188534 0.212365 -1.770233
C -1.916090 1.738691 1.314549
O -0.763553 2.098251 1.005113
C -3.147778 2.284205 0.625793
N -2.031368 0.924964 2.390742
C -0.740358 0.581278 2.971855
C 0.285694 0.525700 1.921648
C 1.088592 -0.000507 1.094607
C 2.141700 -0.991278 1.252473
C 2.505522 -1.913810 0.260071
C 3.513941 -2.844809 0.473741
C 4.196248 -2.863999 1.683116
C 3.866392 -1.951111 2.679571
C 2.852351 -1.032453 2.462027
H -2.736702 0.186749 2.342427
H -0.480667 1.315805 3.747075
H -0.804648 -0.416195 3.430163
Cl 1.697104 -1.947042 -1.284285
H 3.755005 -3.551902 -0.316487

H 4.988553 -3.592562 1.841773
H 4.399313 -1.953050 3.627943
H 2.600944 -0.303749 3.231265
C -2.870778 2.466318 -0.863933
C -4.373385 1.397340 0.827227
C -3.386162 3.653971 1.283445
H -4.263780 4.124645 0.820701
H -3.579037 3.555956 2.360728
H -2.524309 4.318091 1.139086
H -5.227672 1.858286 0.314626
H -4.225182 0.393777 0.408035
H -4.647588 1.300058 1.887318
H -3.741254 2.943904 -1.333151
H -1.993061 3.100371 -1.040204
H -2.691964 1.502968 -1.356921

TS_{ar}^{B}

54
Energy: -4425.80894273
Cu 0.008866 0.459092 -0.629982
Cl -0.412154 0.497489 -2.753218
C 1.836971 1.040378 -0.979822
O -1.793769 -0.148179 -0.225897
S -2.260323 -1.435991 0.423090
O -2.962002 -1.171319 1.684048
C -3.577763 -1.911717 -0.761031
O -1.263795 -2.494071 0.401832
F -4.186976 -3.000593 -0.318386
F -4.462608 -0.926713 -0.857946
F -3.054189 -2.150798 -1.947364
C 2.149102 2.334102 -0.583955
C 3.276631 2.942801 -1.132892
C 4.076998 2.251950 -2.035245
C 3.745168 0.951693 -2.414950
C 2.615066 0.336449 -1.895084
H 1.523277 2.855356 0.136843
H 3.528526 3.960919 -0.840914
H 4.965040 2.727008 -2.448254
H 4.361313 0.414850 -3.133871
H 2.345644 -0.674116 -2.195950
C -1.714091 2.198569 1.050685
O -0.688400 2.515745 0.434581
C -3.068102 2.751512 0.656387
N -1.647415 1.360197 2.115560
C -0.407509 0.674064 2.427516
C 0.459536 0.344376 1.261753
C 1.567515 -0.029594 0.800850
C 2.869939 -0.638667 0.938045
C 3.116077 -1.957881 0.536524
C 4.376168 -2.520251 0.698010
C 5.399817 -1.764128 1.257366
C 5.172922 -0.447517 1.651045
C 3.916191 0.114411 1.483360
H -2.482423 0.810435 2.309595
C 0.446794 1.487653 3.399206
H -0.699531 -0.282286 2.887954
Cl 1.840479 -2.884588 -0.181028
H 4.543320 -3.547476 0.382327
H 6.385295 -2.208780 1.381294
H 5.977793 0.142780 2.083108
H 3.721322 1.149085 1.763976
C -4.254815 2.123367 1.374789
H -3.007788 3.818294 0.936473
C -3.234608 2.671810 -0.860266
H -5.180106 2.614958 1.050995
H -4.351172 1.055820 1.128020
H -4.195567 2.228856 2.466218
H -4.145317 3.204247 -1.161388
H -2.380034 3.114012 -1.382439
H -3.321038 1.626069 -1.181725
H 1.351279 0.934321 3.681195
H 0.741109 2.434760 2.930292
H -0.132825 1.707624 4.302554

I_2

54
Energy: -4425.86445047
Cu 0.249016 -1.411314 -1.893318
Cl 1.017789 -3.419317 -2.390224
C 2.014116 0.429925 -0.583625

O -0.242875 -1.741450 -0.102521
S -1.762425 -1.930714 -0.020435
O -2.328914 -2.014048 -1.372303
C -1.876276 -3.619509 0.692367
O -2.399206 -1.045738 0.938415
F -3.159450 -3.919292 0.836150
F -1.306884 -4.494685 -0.107057
F -1.285813 -3.638235 1.874231
C 2.668876 -0.434471 -1.470015
C 3.766019 -1.179845 -1.044502
C 4.227529 -1.050582 0.257925
C 3.598387 -0.166624 1.135739
C 2.499799 0.570958 0.720118
H 2.371763 -0.469112 -2.519891
H 4.248409 -1.864650 -1.738785
H 5.081604 -1.635237 0.594274
H 3.962013 -0.063758 2.156638
H 1.987205 1.238997 1.412246
C -1.137708 -0.651891 -4.064743
O 0.025687 -0.987991 -3.711493
C -1.720005 -1.335113 -5.286755
N -1.874367 0.217248 -3.380600
C -1.504971 0.825191 -2.106328
C -0.169190 0.418569 -1.623669
C 0.773956 1.120871 -0.997739
C 0.649646 2.569385 -0.692107
C -0.325367 3.098632 0.162034
C -0.388997 4.464323 0.426365
C 0.531653 5.322376 -0.160161
C 1.523664 4.816752 -0.996878
C 1.581560 3.453851 -1.248579
H -2.834263 0.345189 -3.675402
H -1.505868 1.920700 -2.231891
H -2.284951 0.573143 -1.370687
Cl -1.455295 2.041100 0.967347
H -1.155854 4.838707 1.100943
H 0.479055 6.389413 0.047643
H 2.254191 5.484420 -1.448994
H 2.359565 3.043435 -1.892443
C -2.938036 -0.615886 -5.857895
C -0.632761 -1.454419 -6.354536
C -2.116391 -2.736002 -4.788242
H -2.524402 -3.312232 -5.629679
H -1.248675 -3.270459 -4.378903
H -2.878576 -2.679960 -3.998957
H -1.027892 -2.021084 -7.207533
H -0.313913 -0.468546 -6.719871
H 0.243790 -1.981784 -5.963329
H -3.259445 -1.130914 -6.771788
H -3.801792 -0.637552 -5.176462
H -2.718119 0.426768 -6.127364

TS_{rc}^{B}

54
Energy: -4425.83380529
Cu 0.877608 -1.077513 0.653312
Cl 2.693910 -2.317978 0.465988
C -0.492972 1.961467 1.381236
O -0.846922 -1.686640 1.142500
S -1.873977 -2.110869 0.098709
O -1.689267 -1.414379 -1.176547
C -1.345731 -3.831564 -0.260452
O -3.202554 -2.220837 0.665293
F -2.213919 -4.391679 -1.089341
F -0.148123 -3.820488 -0.838211
F -1.282448 -4.540540 0.852570
C -0.030224 1.216967 2.474805
C -0.049388 1.746239 3.755133
C -0.535453 3.032106 3.973885
C -1.003323 3.782398 2.899262
C -0.985842 3.252614 1.616955
H 0.325939 0.199521 2.328429
H 0.304616 1.143012 4.588599
H -0.557985 3.443921 4.981144
H -1.389202 4.787390 3.060280
H -1.352754 3.846780 0.781096
C 2.823455 0.579185 -0.692915
O 2.127270 0.662544 0.354559
C 4.300311 0.897641 -0.669781
N 2.191166 0.316359 -1.837905
C 0.779356 -0.038755 -1.740477

C 0.324046 0.433709 -0.400427
C -0.459085 1.442934 0.008009
C -1.343588 2.055190 -1.018940
C -2.738266 1.930688 -0.983496
C -3.531721 2.545656 -1.947712
C -2.942170 3.297681 -2.955734
C -1.558400 3.441362 -3.003988
C -0.772399 2.821730 -2.041646
H 2.720173 0.056769 -2.661013
H 0.193226 0.466390 -2.519070
H 0.643809 -1.121629 -1.878784
Cl -3.507059 0.998105 0.257514
H -4.611074 2.420516 -1.899495
H -3.569962 3.774282 -3.706093
H -1.093459 4.040393 -3.784299
H 0.312711 2.940136 -2.056793
C 4.843533 0.664217 0.737766
C 5.054483 0.020583 -1.669364
C 4.429986 2.380662 -1.045964
H 6.131101 0.204382 -1.564646
H 4.870483 -1.045022 -1.477270
H 4.800805 0.250647 -2.715120
H 5.488419 2.669933 -1.008596
H 4.057490 2.579874 -2.060256
H 3.878522 3.016372 -0.340548
H 5.915367 0.900552 0.750507
H 4.339342 1.303959 1.471094
H 4.703352 -0.380462 1.042706

Reaction 17

reactant

33
Energy: -3243.80254809
C -4.055778 0.001900 0.187079
O -4.630724 -0.040678 -0.891160
C -4.827231 -0.106044 1.506545
N -2.701999 0.132390 0.251928
H -2.211136 0.197225 1.131644
C -1.932628 0.230907 -0.978234
C -0.505977 0.166270 -0.705975
H -2.243321 -0.581673 -1.651757
H -2.184063 1.164422 -1.506460
C 0.679996 0.115992 -0.466119
C 2.077709 0.045306 -0.188989
C 2.695907 -1.192575 0.035844
C 4.055786 -1.254881 0.306258
C 4.821249 -0.093814 0.356884
C 4.201358 1.130223 0.132259
C 2.844656 1.217599 -0.138593
H 2.094765 -2.098463 -0.006558
H 4.531301 -2.218335 0.479671
H 5.887434 -0.132755 0.567352
Br 5.234529 2.717236 0.198498
H 2.375225 2.183085 -0.312321
C -3.958186 0.010918 2.754656
C -5.875141 1.007899 1.505328
C -5.526697 -1.466992 1.495593
H -4.588622 -0.069985 3.650442
H -3.210531 -0.793522 2.816423
H -3.443554 0.981415 2.811460
H -6.168583 -1.569200 2.381854
H -6.145796 -1.572714 0.597409
H -4.797354 -2.289475 1.508190
H -6.524208 0.916788 2.387494
H -5.402655 2.000035 1.533388
H -6.494580 0.950538 0.603279

I_c

68
Energy: -6844.42703129
Cu 0.709280 0.076271 -0.530779
Cl 1.348135 -0.478286 -2.542157
C 1.933174 1.522795 -0.738865
O -0.568814 -1.479071 -0.386256
S -1.395940 -1.894988 0.802298
O -2.098595 -0.781086 1.445081
C -2.727090 -2.837163 -0.036642
O -0.736455 -2.865444 1.667197
F -3.584687 -3.290971 0.860655

F -3.386342 -2.038414 -0.884428
F -2.222087 -3.849292 -0.717423
C 1.352498 2.749163 -0.481304
C 2.203226 3.852923 -0.359794
C 3.573564 3.713983 -0.527829
C 4.115611 2.464613 -0.819950
C 3.290818 1.347243 -0.934987
C -1.048236 2.992396 2.126725
O -0.173974 3.851714 2.083930
C -2.526781 3.380337 2.064545
N -0.750994 1.667534 2.261845
C 0.613661 1.253522 2.429665
C 1.103810 0.205654 1.512836
C 1.810430 -0.770533 1.232447
C 2.704502 -1.810101 0.864343
C 2.236104 -3.104557 0.584603
C 3.156314 -4.075214 0.227029
C 4.518184 -3.794284 0.150492
C 4.975613 -2.512443 0.442008
C 4.078264 -1.516459 0.794380
H 0.284164 2.877140 -0.332110
H 1.761506 4.814154 -0.103005
H 4.224371 4.581599 -0.436444
H 5.187293 2.350440 -0.977385
H 3.702957 0.375113 -1.197852
H -1.457236 0.942626 2.154824
H 1.231386 2.155330 2.298859
H 0.789078 0.877714 3.450564
H 1.173386 -3.324722 0.663454
Br 2.557997 -5.825960 -0.158770
H 5.213880 -4.580954 -0.133017
H 6.040277 -2.295439 0.390311
H 4.419189 -0.509683 1.029044
C -3.487953 2.205347 2.203755
C -2.764955 4.373176 3.205259
C -2.750218 4.080584 0.722717
O -3.293565 1.138508 -1.028514
C -4.510844 0.722748 -1.665900
C -5.566688 0.693253 -0.593263
C -2.095284 0.875961 -1.535188
O -1.127115 1.214486 -0.867868
C -2.005890 0.207742 -2.868416
H -4.751169 1.439837 -2.464004
H -4.379219 -0.269105 -2.114097
H -6.533048 0.396197 -1.016350
H -5.285649 -0.026996 0.185230
H -5.675328 1.681012 -0.129368
H -2.738773 0.609967 -3.576370
H -0.994038 0.319454 -3.262805
H -2.197071 -0.866118 -2.743789
H -3.353789 1.673917 3.156777
H -4.521006 2.581722 2.185862
H -3.383104 1.484997 1.381916
H -3.788936 4.435754 0.653515
H -2.078273 4.941455 0.620129
H -2.569452 3.390678 -0.112175
H -3.795704 4.752243 3.160665
H -2.624055 3.894223 4.184724
H -2.073115 5.220125 3.135149

I_1

54
Energy: -6536.90564691
Cu -0.012344 0.126536 0.002532
Cl 0.008239 0.308540 2.126048
C 1.852274 0.202019 0.315753
O -2.034423 0.317961 -0.276960
S -2.046230 1.695307 -0.900929
O -2.713557 1.790159 -2.184915
C -3.049062 2.669374 0.286006
O -0.673757 2.242455 -0.796458
F -3.088609 3.929237 -0.114315
F -4.277092 2.178682 0.326261
F -2.508628 2.605085 1.489036
C 2.616169 -0.942432 0.426961
C 3.999800 -0.784552 0.325964
C 4.560009 0.478917 0.152803
C 3.747006 1.605497 0.082915
C 2.360493 1.481150 0.189179
H 2.166450 -1.927170 0.560780
H 4.636608 -1.665573 0.391330

H 5.641372 0.587040 0.089041
H 4.182513 2.596377 -0.035683
H 1.711084 2.355154 0.168766
C -0.443107 -3.203958 0.692425
O 0.620399 -3.533785 0.179203
C -0.803347 -3.585082 2.125913
N -1.383964 -2.540116 -0.045462
C -1.107498 -2.229285 -1.427566
C -0.135940 -1.137395 -1.642405
C 0.591812 -0.334123 -2.223062
C 1.534250 0.513810 -2.866538
C 1.162394 1.800328 -3.285141
C 2.117785 2.599856 -3.891434
C 3.420092 2.148473 -4.090447
C 3.776032 0.867285 -3.679757
C 2.840864 0.045723 -3.069008
H -2.168418 -2.075958 0.394818
H -0.706367 -3.125935 -1.917351
H -2.048074 -1.949238 -1.915367
H 0.150242 2.159729 -3.111703
Br 1.648196 4.339949 -4.462021
H 4.147598 2.801514 -4.567669
H 4.793396 0.514497 -3.835163
H 3.106438 -0.952409 -2.726507
C -2.022017 -2.845815 2.671598
C -1.089840 -5.091422 2.092863
C 0.413731 -3.314463 3.009653
H -1.316777 -5.448639 3.106875
H -1.953132 -5.319600 1.451351
H -0.221569 -5.644580 1.714163
H 0.216380 -3.666728 4.031385
H 1.295692 -3.839009 2.624020
H 0.640226 -2.240233 3.056068
H -2.181859 -3.132653 3.719433
H -1.886986 -1.754839 2.647985
H -2.942386 -3.107257 2.128870

TS$_{rc}^{A}$

54
Energy: -6536.88407140
Cu 0.675341 0.922661 0.761571
Cl 1.556078 1.207750 2.686101
C 0.442135 2.785390 0.733216
O 0.463701 -1.031628 0.939008
S 0.587107 -2.260228 0.079667
O 1.957830 -2.762202 -0.015960
C -0.328558 -3.467788 1.111613
O -0.165618 -2.197454 -1.175835
F -0.342225 -4.639068 0.493620
F 0.250744 -3.601200 2.290572
F -1.576059 -3.048764 1.276999
C 1.335291 3.604613 0.066568
C 1.040794 4.965659 -0.017573
C -0.113156 5.476066 0.570441
C -0.980230 4.629519 1.253560
C -0.702445 3.266096 1.346932
H 2.234284 3.196818 -0.391354
H 1.724106 5.627064 -0.548671
H -0.331239 6.540601 0.505009
H -1.875660 5.025526 1.730245
H -1.371039 2.602791 1.895408
C 3.135765 -0.203637 -1.237752
O 2.533313 0.803019 -0.807020
C 4.440676 -0.674721 -0.637710
N 2.617926 -0.776475 -2.339152
C 1.426317 -0.134600 -2.870047
C 0.718791 0.574607 -1.794936
C -0.200284 0.896868 -0.997853
C -1.652534 1.051371 -1.020131
C -2.485546 -0.011889 -0.662162
C -3.858919 0.188005 -0.671701
C -4.419628 1.409293 -1.026614
C -3.576418 2.458586 -1.377512
C -2.197580 2.291021 -1.369015
H 2.824218 -1.742420 -2.563522
H 1.701329 0.550650 -3.685039
H 0.738323 -0.901720 -3.249356
H -2.064008 -0.979434 -0.401469
Br -4.993367 -1.248231 -0.187586
H -5.499969 1.533237 -1.024954
H -4.001533 3.421001 -1.655948

H -1.533353 3.115847 -1.623656
C 4.319750 -0.674937 0.886009
C 4.854347 -2.052389 -1.146137
C 5.479859 0.370020 -1.076667
H 6.458161 0.094454 -0.661420
H 5.574981 0.411563 -2.171156
H 5.218593 1.369154 -0.707327
H 5.804061 -2.333324 -0.673463
H 4.110853 -2.814906 -0.883268
H 5.027041 -2.057505 -2.232852
H 5.287530 -0.960517 1.319420
H 4.046517 0.315276 1.270336
H 3.560725 -1.391489 1.221219

TS_{ar}^{B}

54
Energy: -6536.89528296
Cu -0.765866 0.935053 -0.505583
Cl -1.320967 1.152807 -2.587480
C 0.521996 2.382383 -0.659449
O -1.892660 -0.667349 -0.314841
S -1.393738 -2.041037 0.073301
O -1.918656 -2.458988 1.376275
C -2.287721 -3.052099 -1.167838
O 0.029526 -2.230989 -0.183956
F -2.018199 -4.332014 -0.968107
F -3.593580 -2.854604 -1.035455
F -1.911660 -2.703890 -2.382600
C 0.190327 3.559089 -0.002256
C 0.790499 4.746012 -0.421682
C 1.715528 4.734584 -1.459574
C 2.044215 3.536946 -2.093898
C 1.445722 2.348327 -1.698811
H -0.531894 3.553441 0.810914
H 0.532534 5.679048 0.076229
H 2.187161 5.662916 -1.776620
H 2.760130 3.526738 -2.913616
H 1.692263 1.410159 -2.192471
C -3.027176 1.098021 1.317208
O -2.376545 2.017226 0.799798
C -4.476000 0.853936 0.953706
N -2.455885 0.298585 2.252717
C -1.037346 0.416668 2.530948
C -0.175009 0.682789 1.346063
C 0.932463 0.921999 0.800236
C 2.357950 0.694181 0.694178
C 2.798600 -0.613102 0.459678
C 4.166631 -0.850019 0.416224
C 5.085400 0.176570 0.601994
C 4.629113 1.473136 0.829350
C 3.269411 1.741306 0.866684
H -2.823690 -0.646396 2.333122
C -0.766482 1.492743 3.580294
H -0.725262 -0.568099 2.912612
H 2.074073 -1.413715 0.309045
Br 4.792779 -2.605767 0.101261
H 6.150736 -0.039704 0.565104
H 5.346954 2.277905 0.973345
H 2.900520 2.753492 1.026501
C -5.065228 -0.434534 1.512672
H -5.007577 1.710542 1.405375
C -4.653816 0.935588 -0.561436
H -6.120396 -0.510284 1.224058
H -4.555133 -1.318363 1.101676
H -5.024471 -0.480479 2.609018
H -5.722068 0.911683 -0.809765
H -4.218203 1.853407 -0.969399
H -4.162504 0.086249 -1.053126
H 0.302702 1.550197 3.815514
H -1.101733 2.468843 3.212122
H -1.320705 1.254733 4.494953

I_2

54
Energy: -6536.94878995
Cu 1.621913 -0.104063 0.642991
Cl 3.681465 -0.787796 1.032296
C -0.555451 -1.142989 2.187530
O 1.099675 -1.572618 -0.404957
S 0.939245 -1.127673 -1.862805

O 1.675861 0.117200 -2.102518
C 1.872487 -2.456342 -2.719612
O -0.435265 -1.206113 -2.333233
F 1.831123 -2.216414 -4.021592
F 3.123574 -2.462648 -2.311118
F 1.310022 -3.624877 -2.469127
C 0.620677 -0.961725 2.927929
C 1.157077 -2.011857 3.668664
C 0.513793 -3.241641 3.693084
C -0.676182 -3.418170 2.986434
C -1.209542 -2.378100 2.239261
H 1.078427 0.026998 2.992169
H 2.083607 -1.860023 4.218394
H 0.934086 -4.066021 4.265865
H -1.183084 -4.381300 3.007720
H -2.121009 -2.524935 1.660896
C 1.878390 2.545194 0.287581
O 2.056720 1.643744 1.151865
C 3.015198 3.520630 0.052719
N 0.779366 2.622993 -0.453820
C -0.334760 1.677641 -0.438060
C -0.152585 0.564460 0.522085
C -1.040373 -0.069522 1.292865
C -2.490971 0.227749 1.251893
C -3.157327 0.316376 0.025636
C -4.516396 0.604881 0.008653
C -5.230909 0.797542 1.184426
C -4.566347 0.685721 2.402902
C -3.208950 0.396694 2.440928
H 0.760420 3.322765 -1.185115
H -1.247416 2.227844 -0.155637
H -0.465469 1.311039 -1.467065
H -2.625708 0.113458 -0.904173
Br -5.415912 0.725492 -1.653559
H -6.294382 1.021436 1.144115
H -5.119006 0.826314 3.329975
H -2.689462 0.304819 3.394020
C 2.580784 4.789007 -0.675353
C 3.652822 3.877935 1.394917
C 4.018677 2.731019 -0.806468
H 4.893035 3.364328 -1.008655
H 4.352277 1.820771 -0.290264
H 3.575757 2.431885 -1.766668
H 4.526877 4.517740 1.217604
H 2.954906 4.427445 2.041268
H 3.983106 2.977731 1.924170
H 3.438033 5.469977 -0.746489
H 2.258653 4.597069 -1.709879
H 1.780340 5.321345 -0.142637

TS_{rc}^{B}

54
Energy: -6536.92022691
Cu -1.710257 1.036544 0.101338
Cl -3.420346 1.740371 -1.090361
C 0.114079 -1.194059 1.988084
O -0.330263 2.014316 0.968127
S 0.861857 2.315466 0.069116
O 0.666327 1.785186 -1.282161
C 0.702347 4.133187 -0.106323
O 2.133319 2.069721 0.731299
F 1.672971 4.585440 -0.884629
F -0.468135 4.420528 -0.653508
F 0.785853 4.711574 1.080150
C -0.835391 -0.467047 2.722324
C -0.877157 -0.543641 4.103588
C 0.031150 -1.347663 4.788375
C 0.981753 -2.071733 4.076853
C 1.025091 -1.996141 2.691593
H -1.531958 0.183217 2.201812
H -1.615431 0.039662 4.650048
H 0.001923 -1.401400 5.875126
H 1.699594 -2.698226 4.602909
H 1.772126 -2.569217 2.145876
C -3.109486 -1.338141 -0.794994
O -2.620445 -0.930334 0.292921
C -4.514903 -1.892910 -0.842320
N -2.317785 -1.367943 -1.866351
C -1.011717 -0.730393 -1.746339
C -0.735629 -0.578635 -0.287494
C 0.156003 -1.164709 0.523969

C 1.321388 -1.792379 -0.171498
C 2.546338 -1.125763 -0.233708
C 3.615483 -1.755580 -0.859828
C 3.489238 -3.021920 -1.421420
C 2.262690 -3.674291 -1.355011
C 1.178890 -3.063630 -0.733849
H -2.722247 -1.497417 -2.785209
H -0.235487 -1.369534 -2.187795
H -1.006432 0.233430 -2.275092
H 2.654993 -0.126621 0.192571
Br 5.283677 -0.870297 -0.956501
H 4.344952 -3.487509 -1.904965
H 2.156649 -4.668227 -1.785846
H 0.215888 -3.572205 -0.669140
C -5.385272 -1.121760 0.149074
C -5.105992 -1.785728 -2.246345
C -4.407724 -3.364643 -0.418205
H -6.150074 -2.121650 -2.221703
H -5.096546 -0.747566 -2.605650
H -4.589985 -2.430370 -2.973650
H -5.411035 -3.810407 -0.407885
H -3.784213 -3.942603 -1.114431
H -3.982273 -3.455058 0.589387
H -6.404691 -1.527964 0.121707
H -5.002290 -1.216873 1.171366
H -5.418833 -0.054543 -0.104726

Reaction 18

reactant

33
Energy: -3243.80295309
C -4.056815 0.001726 0.186790
O -4.633469 -0.027801 -0.891044
C -4.828501 -0.098022 1.506773
N -2.701123 0.108906 0.250252
H -2.207062 0.156678 1.129252
C -1.931621 0.193849 -0.981132
C -0.504752 0.142207 -0.707523
H -2.235897 -0.629420 -1.644809
H -2.190358 1.118447 -1.521147
C 0.681562 0.100181 -0.466560
C 2.079152 0.042216 -0.188283
C 2.711409 -1.192500 0.015850
C 4.069977 -1.255483 0.288555
C 4.803498 -0.076120 0.357441
C 4.201072 1.161161 0.157913
C 2.841733 1.216361 -0.113665
H 2.122838 -2.106043 -0.043271
H 4.562199 -2.212236 0.446618
Br 6.657638 -0.157164 0.729678
H 4.794461 2.070700 0.215550
H 2.354825 2.176607 -0.272815
C -3.957665 0.012625 2.754211
C -5.867004 1.024586 1.505442
C -5.539345 -1.453141 1.497269
H -4.588578 -0.058291 3.650515
H -3.219472 -0.800352 2.818491
H -3.431575 0.977128 2.807411
H -6.180681 -1.549907 2.384550
H -6.160800 -1.553875 0.600098
H -4.816835 -2.281670 1.508857
H -6.517080 0.938810 2.387399
H -5.386199 2.012706 1.533760
H -6.486511 0.972622 0.603144

I_c

68
Energy: -6844.42917277
Cu 0.706289 0.091480 -0.529971
Cl 1.336841 -0.483210 -2.539651
C 1.938116 1.531583 -0.727178
O -0.579967 -1.458492 -0.381126
S -1.417802 -1.883723 0.795469
O -2.142538 -0.780222 1.431661
C -2.723982 -2.840241 -0.065410
O -0.761159 -2.850304 1.667779
F -3.604731 -3.285214 0.813971
F -3.361378 -2.056088 -0.942080
F -2.197595 -3.860823 -0.718355

C 1.368769 2.759376 -0.451810
C 2.226375 3.858760 -0.341448
C 3.592829 3.714078 -0.535461
C 4.123422 2.463407 -0.842270
C 3.291404 1.350027 -0.946375
C -1.050674 2.996884 2.134023
O -0.169320 3.849654 2.110655
C -2.525255 3.396534 2.058644
N -0.764596 1.667874 2.252898
C 0.594119 1.241187 2.434951
C 1.093232 0.210286 1.502255
C 1.812756 -0.763260 1.238227
C 2.699953 -1.805107 0.874913
C 2.230774 -3.103145 0.607715
C 3.127304 -4.095874 0.250407
C 4.484794 -3.796007 0.169752
C 4.970912 -2.519082 0.441925
C 4.076251 -1.521981 0.789718
H 0.304101 2.891619 -0.281136
H 1.793856 4.821125 -0.073196
H 4.249498 4.578116 -0.452291
H 5.191478 2.344922 -1.019922
H 3.694329 0.377372 -1.221382
H -1.477354 0.950354 2.140841
H 1.218800 2.141771 2.333187
H 0.749153 0.843423 3.450669
H 1.166082 -3.310279 0.695897
H 2.780168 -5.103531 0.036330
Br 5.701464 -5.154850 -0.312488
H 6.037195 -2.317223 0.378443
H 4.428539 -0.515463 1.009961
C -3.495337 2.228396 2.193227
C -2.767135 4.395590 3.192847
C -2.730649 4.093366 0.711983
O -3.288715 1.141104 -1.031542
C -4.507217 0.698930 -1.648615
C -5.531978 0.600180 -0.549831
C -2.091411 0.898496 -1.550520
O -1.121443 1.246006 -0.890703
C -2.003545 0.239545 -2.888729
H -4.793481 1.429062 -2.419205
H -4.353873 -0.274250 -2.128966
H -6.492389 0.258705 -0.952598
H -5.192079 -0.114235 0.210686
H -5.680514 1.574048 -0.068295
H -2.749828 0.632967 -3.587630
H -0.996983 0.370999 -3.290627
H -2.174193 -0.838369 -2.768133
H -3.374141 1.701034 3.150286
H -4.525749 2.611029 2.162393
H -3.386064 1.503107 1.376262
H -3.765803 4.456185 0.631454
H -2.051165 4.948808 0.612972
H -2.546724 3.399285 -0.118616
H -3.794341 4.782541 3.136017
H -2.640058 3.919814 4.175714
H -2.067960 5.236740 3.126466

I_1

54
Energy: -6536.90764805
Cu -0.011562 0.115736 0.003350
Cl 0.003376 0.317883 2.125231
C 1.853587 0.188717 0.311912
O -2.031182 0.318099 -0.288339
S -2.038155 1.695551 -0.910880
O -2.698199 1.792589 -2.199052
C -3.049121 2.668701 0.269794
O -0.667471 2.244665 -0.799704
F -3.084515 3.929318 -0.130142
F -4.277983 2.179599 0.300251
F -2.518054 2.603591 1.476710
C 2.617574 -0.954788 0.431345
C 4.001470 -0.797298 0.331009
C 4.562028 0.464876 0.149767
C 3.749015 1.590887 0.071219
C 2.362384 1.466877 0.176872
H 2.168063 -1.938858 0.570449
H 4.638216 -1.677879 0.402639
H 5.643348 0.572688 0.085355
H 4.184987 2.580666 -0.055115

H 1.712789 2.340562 0.150088
C -0.444948 -3.204920 0.703689
O 0.620090 -3.540348 0.197399
C -0.812796 -3.577658 2.137493
N -1.380019 -2.541504 -0.041920
C -1.095768 -2.237002 -1.423994
C -0.126737 -1.141691 -1.637977
C 0.598513 -0.347927 -2.236851
C 1.530289 0.507632 -2.879298
C 1.155075 1.793972 -3.297914
C 2.092530 2.624817 -3.890407
C 3.394927 2.164890 -4.067141
C 3.781389 0.887093 -3.671807
C 2.845553 0.058400 -3.075914
H -2.167973 -2.077293 0.391896
H -0.688646 -3.135177 -1.905793
H -2.034430 -1.962995 -1.918910
H 0.135428 2.134500 -3.129621
H 1.820632 3.626725 -4.212824
Br 4.673368 3.304874 -4.863215
H 4.805544 0.555267 -3.823665
H 3.124997 -0.937842 -2.737896
C -2.033687 -2.834550 2.672892
C -1.099717 -5.084020 2.112082
C 0.400048 -3.302244 3.025639
H 0.197428 -3.648214 4.048494
H 1.283640 -3.829547 2.647588
H 0.626819 -2.227868 3.066664
H -1.331607 -5.435102 3.127116
H -1.960003 -5.315804 1.467818
H -0.229834 -5.639675 1.740842
H -2.199115 -3.115638 3.721422
H -1.897654 -1.743796 2.644243
H -2.951504 -3.098277 2.126909

TS_{rc}^{A}

54
Energy: -6536.88494708
Cu -0.576536 0.843804 -0.819272
Cl -1.426428 1.453363 -2.680578
C 0.338406 2.483915 -0.842954
O -1.079709 -1.057386 -0.981562
S -1.638003 -2.113445 -0.066593
O -3.073296 -1.966602 0.178792
C -1.456973 -3.590383 -1.137100
O -0.807092 -2.394467 1.106133
F -1.906861 -4.652442 -0.485903
F -2.142698 -3.439339 -2.254822
F -0.174562 -3.770004 -1.426842
C -0.127970 3.575776 -0.133446
C 0.668558 4.720480 -0.091751
C 1.887615 4.757391 -0.762811
C 2.318072 3.650785 -1.487494
C 1.535304 2.497782 -1.539594
H -1.081637 3.537690 0.389187
H 0.326566 5.587585 0.471858
H 2.499350 5.657225 -0.729525
H 3.262895 3.679182 -2.028112
H 1.862394 1.634910 -2.119673
C -3.135970 0.754832 1.391724
O -2.228189 1.430198 0.863902
C -4.568721 0.826006 0.915173
N -2.794906 0.057836 2.491800
C -1.403357 0.172292 2.895958
C -0.564522 0.532485 1.743609
C 0.347908 0.477514 0.879848
C 1.750831 0.085104 0.791362
C 2.122903 -1.189692 0.355084
C 3.468932 -1.521178 0.261715
C 4.433160 -0.578968 0.599059
C 4.076970 0.695047 1.025555
C 2.732370 1.027543 1.114851
H -3.345698 -0.741031 2.781453
H -1.306170 0.915184 3.701170
H -1.046423 -0.802879 3.253893
H 1.356961 -1.921980 0.108701
H 3.768497 -2.512371 -0.070350
Br 6.266586 -1.035292 0.471945
H 4.845589 1.420701 1.280994
H 2.434379 2.027782 1.427830
C -4.593590 0.795344 -0.612483

C -5.433661 -0.290755 1.491517
C -5.082978 2.186438 1.415263
H -6.125243 2.313137 1.094177
H -5.054846 2.251656 2.512308
H -4.492362 3.011262 0.998007
H -6.454364 -0.184634 1.102595
H -5.061499 -1.278385 1.192967
H -5.505814 -0.235007 2.588185
H -5.629293 0.924246 -0.954229
H -3.984147 1.599417 -1.042580
H -4.214811 -0.160110 -0.993855

TS_{ar}^{B}

54
Energy: -6536.89693511
Cu -0.903616 0.841928 -0.500195
Cl -1.486398 1.189633 -2.557289
C 0.602239 2.060079 -0.672599
O -2.262460 -0.572996 -0.296037
S -1.966274 -2.015412 0.044715
O -2.482562 -2.377641 1.367634
C -3.065169 -2.851093 -1.162304
O -0.605179 -2.419959 -0.291953
F -2.971754 -4.161762 -1.004021
F -4.319876 -2.473726 -0.950674
F -2.710301 -2.526910 -2.390097
C 0.494588 3.260197 0.016667
C 1.265672 4.342176 -0.407204
C 2.135927 4.203344 -1.482605
C 2.238111 2.983112 -2.150072
C 1.467579 1.899977 -1.750111
H -0.187043 3.354220 0.858879
H 1.184610 5.292846 0.116866
H 2.742286 5.048611 -1.802851
H 2.910865 2.874732 -2.998678
H 1.537909 0.945681 -2.268737
C -3.043680 1.321722 1.403664
O -2.274234 2.137210 0.875476
C -4.524093 1.314941 1.089306
N -2.574345 0.423028 2.305143
C -1.146197 0.308905 2.531873
C -0.295270 0.455630 1.317784
C 0.821401 0.522647 0.741147
C 2.181858 0.063325 0.575728
C 2.400437 -1.292481 0.305584
C 3.700886 -1.768709 0.193780
C 4.764233 -0.888892 0.357716
C 4.558437 0.463958 0.621444
C 3.262269 0.941682 0.719134
H -3.086956 -0.451549 2.387092
C -0.671511 1.308068 3.584733
H -0.982159 -0.719660 2.889244
H 1.544672 -1.955455 0.176312
H 3.888658 -2.818059 -0.020026
Br 6.533474 -1.537433 0.213265
H 5.409254 1.129990 0.741991
H 3.076230 1.998784 0.905808
C -5.290271 0.128836 1.659679
H -4.898043 2.239557 1.564043
C -4.735997 1.440827 -0.418567
H -6.352662 0.221114 1.404412
H -4.937064 -0.818965 1.226869
H -5.223290 0.065170 2.753831
H -5.801532 1.592680 -0.630529
H -4.170799 2.280658 -0.835567
H -4.404442 0.528729 -0.931457
H 0.400948 1.191564 3.779763
H -0.860956 2.331899 3.243489
H -1.223500 1.142515 4.516592

I_2

54
Energy: -6536.94937659
Cu 0.262358 -1.440755 -1.973893
Cl 0.890767 -3.505111 -2.423949
C 2.079868 0.388591 -0.710555
O -0.127595 -1.728644 -0.158055
S -1.642352 -1.652711 0.055711
O -2.351516 -1.870741 -1.208848
C -1.909652 -3.159926 1.069416

O -2.046038 -0.517587 0.873087
F -3.201981 -3.247726 1.346286
F -1.527281 -4.225830 0.399028
F -1.224080 -3.064170 2.194253
C 2.695476 -0.466763 -1.634934
C 3.800612 -1.228424 -1.264741
C 4.312554 -1.126625 0.021738
C 3.726639 -0.250821 0.935847
C 2.619367 0.503073 0.574800
H 2.361409 -0.478572 -2.673945
H 4.251866 -1.903697 -1.988681
H 5.172729 -1.724973 0.315654
H 4.129739 -0.167340 1.943588
H 2.141524 1.162636 1.298287
C -1.103187 -0.683202 -4.159477
O 0.044210 -1.056499 -3.791681
C -1.699414 -1.363221 -5.376472
N -1.817655 0.214418 -3.490421
C -1.442233 0.840187 -2.224044
C -0.120239 0.402250 -1.718791
C 0.825497 1.084744 -1.067462
C 0.633167 2.488111 -0.635828
C -0.520205 2.861331 0.062074
C -0.701866 4.176277 0.475781
C 0.278846 5.117656 0.189180
C 1.443585 4.765087 -0.486019
C 1.619137 3.448474 -0.887608
H -2.763230 0.385145 -3.809051
H -1.404874 1.931077 -2.377589
H -2.246907 0.625291 -1.505373
H -1.257374 2.100921 0.323967
H -1.591523 4.467196 1.029544
Br 0.035470 6.911413 0.742995
H 2.202765 5.516737 -0.689749
H 2.529988 3.156029 -1.409522
C -2.868785 -0.595478 -5.985082
C -0.604166 -1.567728 -6.422536
C -2.176102 -2.728031 -4.847924
H -2.610433 -3.301747 -5.677744
H -1.341939 -3.299987 -4.419813
H -2.940783 -2.611079 -4.067132
H -1.016097 -2.134824 -7.267180
H -0.227949 -0.610048 -6.807434
H 0.237320 -2.130148 -6.004094
H -3.200025 -1.113206 -6.893870
H -3.745045 -0.557588 -5.320503
H -2.590470 0.428286 -6.272064

TS_{rc}^{B}

54
Energy: -6536.92177095
Cu -2.130695 0.264498 -0.117254
Cl -3.749896 -0.176984 -1.541094
C 0.234599 -0.419386 2.274931
O -1.592101 1.924541 0.649463
S -0.637638 2.714297 -0.235143
O -0.335666 1.997948 -1.476591
C -1.713305 4.110592 -0.738535
O 0.466889 3.293868 0.513902
F -1.047137 4.900042 -1.566646
F -2.788525 3.641561 -1.350927
F -2.081461 4.802947 0.326707
C -1.058556 -0.223095 2.782998
C -1.284216 -0.151254 4.146491
C -0.221808 -0.271027 5.039378
C 1.067434 -0.460416 4.553214
C 1.295458 -0.532398 3.185769
H -1.891808 -0.100437 2.097622
H -2.294997 0.013538 4.513991
H -0.399333 -0.207514 6.111390
H 1.905055 -0.549260 5.242446
H 2.308296 -0.683009 2.816905
C -1.964460 -2.569648 -0.682228
O -1.933944 -1.847303 0.350427
C -2.863877 -3.783294 -0.741464
N -1.105493 -2.297255 -1.663938
C -0.342670 -1.059136 -1.558556
C -0.420577 -0.619337 -0.134609
C 0.497581 -0.541745 0.838731
C 1.913123 -0.509308 0.361110
C 2.599926 0.702696 0.261459

C 3.923620 0.716094 -0.164001
C 4.550597 -0.481923 -0.486694
C 3.880567 -1.697164 -0.394319
C 2.557056 -1.704087 0.029080
H -1.232647 -2.722174 -2.573854
H 0.709153 -1.239449 -1.816965
H -0.742739 -0.304097 -2.250207
H 2.091870 1.637819 0.505837
H 4.464790 1.655239 -0.251495
Br 6.352150 -0.464200 -1.066764
H 4.392497 -2.623231 -0.645774
H 2.016321 -2.648077 0.114612
C -4.154470 -3.481387 0.019119
C -3.188124 -4.163252 -2.184613
C -2.095644 -4.918247 -0.049279
H -3.903110 -4.995345 -2.183504
H -3.650560 -3.325255 -2.724049
H -2.305030 -4.514157 -2.739414
H -2.719905 -5.821352 -0.038779
H -1.160821 -5.155216 -0.576023
H -1.855036 -4.656022 0.988897
H -4.805097 -4.364819 -0.015583
H -3.950345 -3.243186 1.069073
H -4.683604 -2.629721 -0.427293

Reaction 19

reactant

33
Energy: -3243.80354595
C -4.037199 -0.353858 0.211310
O -4.707925 -0.792130 -0.712745
C -4.688067 0.153671 1.502338
N -2.680785 -0.302828 0.127603
H -2.104889 0.056013 0.876458
C -2.010727 -0.775202 -1.072745
C -0.576087 -0.624173 -0.900947
H -2.285336 -1.824580 -1.262491
H -2.371860 -0.211859 -1.947444
C 0.589819 -0.444597 -0.631020
C 1.946240 -0.216582 -0.273536
C 2.988296 -0.563222 -1.146494
C 4.313360 -0.343281 -0.802026
C 4.627894 0.230038 0.426772
C 3.614137 0.582959 1.310827
C 2.290271 0.359984 0.959879
H 2.730231 -1.010749 -2.104340
H 5.104711 -0.620434 -1.495441
H 5.665728 0.405269 0.703125
H 3.844413 1.031562 2.274425
Br 0.934580 0.852325 2.189273
C -3.699334 0.671655 2.542801
C -5.648388 1.275951 1.104733
C -5.481775 -1.015990 2.087331
H -4.247188 1.015662 3.430576
H -3.002889 -0.110803 2.879085
H -3.118377 1.529085 2.171776
H -6.046608 -0.685479 2.970346
H -6.185271 -1.410962 1.345562
H -4.815676 -1.833689 2.397334
H -6.215952 1.616276 1.982246
H -5.104393 2.139756 0.696936
H -6.353223 0.925175 0.342367

I_c

68
Energy: -6844.42182384
Cu 0.526109 -0.319894 -0.325732
Cl 1.149381 -1.231013 -2.229720
C 1.741900 1.118581 -0.686021
O -0.923044 -1.690729 -0.037272
S -1.821587 -2.167373 1.065746
O -1.935593 -1.222901 2.183563
C -3.465031 -2.068672 0.250935
O -1.681920 -3.577913 1.389588
F -4.403754 -2.513066 1.065598
F -3.752541 -0.797847 -0.069036
F -3.483278 -2.783643 -0.867914
C 1.209238 2.322404 -0.264301
C 2.023155 3.455645 -0.325377

C 3.320814 3.366369 -0.811552
C 3.817158 2.139077 -1.239198
C 3.026444 0.989802 -1.177988
C -1.005766 2.683311 2.322904
O -0.076214 3.465266 2.491281
C -2.430064 3.194006 2.092314
N -0.823300 1.332864 2.314349
C 0.445292 0.758181 2.654829
C 1.066604 -0.137135 1.648357
C 1.939967 -0.974797 1.369503
C 3.046685 -1.779846 1.015457
C 2.948008 -3.174094 0.823676
C 4.074738 -3.911209 0.487302
C 5.304708 -3.276172 0.352557
C 5.426050 -1.901739 0.554020
C 4.305006 -1.158869 0.879239
H 0.201819 2.410719 0.128521
H 1.619090 4.397342 0.043044
H 3.948529 4.254451 -0.858355
H 4.826383 2.061098 -1.641778
H 3.408528 0.036261 -1.533140
H -1.590195 0.676485 2.199952
H 1.131905 1.599079 2.831790
H 0.365877 0.174280 3.584286
Br 1.291931 -4.015785 1.028661
H 3.982085 -4.983680 0.334678
H 6.181331 -3.865964 0.090527
H 6.393428 -1.414839 0.455243
H 4.371033 -0.083558 1.035514
C -3.473620 2.089789 1.957144
C -2.778207 4.087592 3.284621
C -2.395382 4.035425 0.813749
O -3.194356 1.529739 -1.441856
C -4.404870 1.364660 -2.192290
C -5.507107 1.979086 -1.370089
C -2.126342 0.760860 -1.636239
O -1.201408 0.912314 -0.850822
C -2.128820 -0.209940 -2.769808
H -4.288983 1.863325 -3.165346
H -4.593584 0.298193 -2.368568
H -6.460169 1.929029 -1.908801
H -5.611909 1.439848 -0.420835
H -5.284566 3.029545 -1.147857
H -2.583424 0.216581 -3.670527
H -1.105254 -0.532314 -2.976942
H -2.704385 -1.099028 -2.481029
H -3.540259 1.467856 2.861668
H -4.462690 2.544385 1.801473
H -3.274596 1.437395 1.096092
H -3.372157 4.515885 0.655260
H -1.630547 4.818501 0.889631
H -2.180286 3.409701 -0.063339
H -3.763609 4.549538 3.130954
H -2.819233 3.508350 4.218008
H -2.031240 4.880280 3.406134

I_1

54
Energy: -6536.90230045
Cu -0.010444 0.138851 0.011084
Cl 0.000438 0.183995 2.148877
C 1.850441 0.122269 0.348429
O -2.012650 0.382063 -0.213894
S -2.046198 1.815394 -0.705791
O -2.929479 2.060040 -1.827806
C -2.803982 2.725501 0.697813
O -0.642955 2.282419 -0.767648
F -2.921634 4.001117 0.364512
F -4.002429 2.222697 0.947483
F -2.052092 2.624781 1.778191
C 2.573303 -1.052259 0.442100
C 3.964818 -0.940574 0.445715
C 4.577474 0.310196 0.389366
C 3.807143 1.466416 0.327055
C 2.412856 1.385219 0.325077
H 2.084992 -2.026653 0.491292
H 4.566402 -1.846522 0.502854
H 5.663513 0.382533 0.412257
H 4.281480 2.446113 0.298529
H 1.796170 2.283106 0.314833
C -0.547012 -3.217672 0.595249

O 0.517983 -3.590592 0.115416
C -0.993481 -3.634340 1.994489
N -1.415503 -2.471684 -0.152004
C -1.077174 -2.137185 -1.513056
C -0.107253 -1.029712 -1.682969
C 0.635893 -0.281690 -2.320110
C 1.541559 0.563940 -3.003809
C 1.101907 1.564909 -3.892967
C 2.021761 2.370601 -4.549151
C 3.383175 2.192212 -4.324941
C 3.836589 1.201186 -3.457557
C 2.922446 0.387819 -2.810114
H -2.197921 -1.989899 0.272952
H -0.651564 -3.026972 -1.993854
H -1.995909 -1.853084 -2.039812
Br -0.728541 1.802599 -4.224095
H 1.665266 3.136412 -5.233678
H 4.095297 2.833818 -4.840871
H 4.901694 1.061787 -3.285342
H 3.255400 -0.393812 -2.129119
C -2.192306 -2.848704 2.519317
C -1.363118 -5.118773 1.882894
C 0.195395 -3.472516 2.940679
H -0.065334 -3.860948 3.934764
H 1.064846 -4.025069 2.566181
H 0.475360 -2.415598 3.048609
H -1.657265 -5.503687 2.869155
H -2.207146 -5.269085 1.194437
H -0.510960 -5.705810 1.518835
H -2.413450 -3.167321 3.546705
H -1.996647 -1.766999 2.546300
H -3.101111 -3.035783 1.928501

TS_{rc}^{A}

54
Energy: -6536.87919182
Cu 0.023467 -0.008454 -0.100912
Cl -0.029858 -0.199655 2.026850
C -0.244951 1.826438 0.196149
O -0.218024 -1.921849 -0.476042
S 0.767295 -3.056304 -0.542497
O 1.901365 -2.897877 0.368561
C -0.252042 -4.415757 0.140703
O 1.098737 -3.468773 -1.908081
F 0.459895 -5.534269 0.141364
F -0.618573 -4.130285 1.376729
F -1.327539 -4.585125 -0.612701
C 0.795125 2.734089 0.091757
C 0.500537 4.093339 0.205380
C -0.805053 4.516936 0.436331
C -1.826863 3.581154 0.564717
C -1.549702 2.219301 0.454585
H 1.813133 2.398045 -0.092420
H 1.304294 4.822390 0.110328
H -1.024049 5.579263 0.529324
H -2.847166 3.903957 0.766565
H -2.344069 1.484836 0.581312
C 3.273417 -0.664540 -0.811855
O 2.358932 0.166595 -0.631602
C 4.365602 -0.882779 0.210270
N 3.329851 -1.229083 -2.028801
C 2.276217 -0.864704 -2.960939
C 1.228462 -0.066221 -2.291117
C 0.060330 0.354147 -2.067391
C -1.152994 0.680299 -2.803951
C -1.848980 -0.260196 -3.578598
C -2.997081 0.096826 -4.275734
C -3.459222 1.406378 -4.227314
C -2.770326 2.358983 -3.483737
C -1.635117 1.995715 -2.773491
H 3.820547 -2.104548 -2.164713
H 2.695841 -0.291226 -3.801517
H 1.793396 -1.778376 -3.340001
Br -1.228585 -2.037516 -3.708010
H -3.517010 -0.657799 -4.861423
H -4.356745 1.680172 -4.778654
H -3.118112 3.389400 -3.450725
H -1.090389 2.735381 -2.190596
C 3.795348 -0.702930 1.615404
C 5.026999 -2.250135 0.063377
C 5.393600 0.226522 -0.072910

H 6.213293 0.145674 0.653162
H 5.820440 0.139672 -1.081703
H 4.941843 1.221636 0.032773
H 5.782126 -2.366501 0.851303
H 4.292653 -3.057082 0.171893
H 5.556692 -2.356983 -0.895449
H 4.612299 -0.786865 2.344471
H 3.325422 0.281618 1.733166
H 3.038772 -1.462936 1.836212

TS_{ar}^{B}

54
Energy: -6536.89334638
Cu 0.104124 -0.586374 -0.628538
Cl 0.511899 -0.498822 -2.753107
C -1.681143 -1.288316 -0.991323
O 1.865466 0.130545 -0.206211
S 2.308450 1.400431 0.489863
O 3.028049 1.101742 1.733336
C 3.602691 1.956485 -0.684915
O 1.294401 2.441503 0.520928
F 4.175134 3.052524 -0.211722
F 4.522764 1.008535 -0.816705
F 3.064495 2.212908 -1.861268
C -1.901085 -2.613482 -0.640093
C -2.984756 -3.280437 -1.209305
C -3.833325 -2.617387 -2.087985
C -3.594665 -1.284596 -2.421806
C -2.510076 -0.609184 -1.880241
H -1.239272 -3.114343 0.062387
H -3.164032 -4.322859 -0.951453
H -4.686402 -3.139419 -2.517659
H -4.248617 -0.767547 -3.121564
H -2.315784 0.428963 -2.142383
C 1.943751 -2.292619 0.962387
O 0.931389 -2.642397 0.341865
C 3.321148 -2.751220 0.531749
N 1.841291 -1.501944 2.059653
C 0.567929 -0.900509 2.411328
C -0.335343 -0.586513 1.269643
C -1.468391 -0.271160 0.825392
C -2.805317 0.253868 0.986527
C -3.139080 1.563097 0.618665
C -4.430852 2.038008 0.804386
C -5.398955 1.205016 1.354628
C -5.083491 -0.103048 1.713565
C -3.794746 -0.578623 1.521686
H 2.647154 -0.915339 2.269843
C -0.221205 -1.793634 3.368065
H 0.810799 0.056506 2.898384
Br -1.825349 2.671576 -0.147108
H -4.670479 3.058378 0.514457
H -6.409816 1.582035 1.497402
H -5.844319 -0.754603 2.137506
H -3.531931 -1.605610 1.774092
C 4.478272 -2.105128 1.281503
H 3.317994 -3.833914 0.750017
C 3.467596 -2.578496 -0.979913
H 5.426214 -2.525258 0.924449
H 4.516349 -1.020671 1.102840
H 4.431404 -2.280905 2.364464
H 4.407815 -3.033496 -1.315249
H 2.638179 -3.047918 -1.518794
H 3.480699 -1.514446 -1.248974
H -1.152789 -1.304718 3.679421
H -0.465746 -2.741468 2.872710
H 0.383980 -2.006584 4.256198

I_2

54
Energy: -6536.94868768
Cu 0.230675 -1.395642 -1.867752
Cl 0.977051 -3.421728 -2.324492
C 2.018028 0.445024 -0.582002
O -0.277831 -1.683295 -0.071824
S -1.787858 -1.935386 0.007806
O -2.364502 -1.935753 -1.342775
C -1.851888 -3.676929 0.590647
O -2.440362 -1.152126 1.041409
F -3.126043 -4.009428 0.746257

F -1.292646 -4.478926 -0.288031
F -1.228196 -3.774862 1.751382
C 2.661904 -0.446950 -1.449182
C 3.748004 -1.198304 -1.006829
C 4.210707 -1.047348 0.292942
C 3.593544 -0.135869 1.150734
C 2.505181 0.607570 0.718730
H 2.366377 -0.498739 -2.498803
H 4.220946 -1.904609 -1.685927
H 5.056526 -1.636281 0.642451
H 3.958313 -0.015594 2.169321
H 2.001988 1.297001 1.396384
C -1.132955 -0.651657 -4.062004
O 0.020740 -1.004797 -3.694307
C -1.718433 -1.343454 -5.277673
N -1.858423 0.241781 -3.397578
C -1.490726 0.855656 -2.125654
C -0.159206 0.444593 -1.636486
C 0.787602 1.142473 -1.011818
C 0.664103 2.592166 -0.706704
C -0.274807 3.111929 0.192175
C -0.337314 4.477150 0.457022
C 0.545976 5.343464 -0.174398
C 1.500700 4.845682 -1.057526
C 1.559494 3.482580 -1.310394
H -2.812931 0.383945 -3.703191
H -1.487362 1.950375 -2.256329
H -2.273835 0.608061 -1.391859
Br -1.447482 1.949431 1.119932
H -1.073759 4.849191 1.165799
H 0.493404 6.410403 0.034230
H 2.201915 5.519528 -1.545667
H 2.309766 3.078678 -1.990363
C -2.919396 -0.612075 -5.869047
C -0.626145 -1.499990 -6.335356
C -2.143232 -2.728642 -4.758902
H -2.558350 -3.310733 -5.592776
H -1.287099 -3.272232 -4.337397
H -2.907669 -2.645890 -3.974059
H -1.025288 -2.074099 -7.181485
H -0.287422 -0.526251 -6.715014
H 0.238219 -2.035917 -5.928929
H -3.243754 -1.136132 -6.776739
H -3.788030 -0.607208 -5.193503
H -2.678914 0.421925 -6.153834

TS_{rc}^{B}

54
Energy: -6536.92039952
Cu 0.673324 0.517411 0.711035
Cl 2.118026 1.810805 1.764815
C -1.647683 1.446789 1.252023
O 0.859546 -1.408105 0.969993
S 1.786481 -2.015401 -0.056175
O 2.257605 -0.995475 -1.008642
C 3.256747 -2.429272 0.955377
O 1.296638 -3.268916 -0.610885
F 4.184549 -2.965974 0.175138
F 3.734449 -1.334506 1.516268
F 2.922155 -3.298033 1.895743
C -1.792514 2.834949 1.312133
C -1.590977 3.501207 2.513219
C -1.254974 2.788957 3.662881
C -1.116199 1.408184 3.612665
C -1.302406 0.734336 2.409079
H -2.021555 3.390379 0.402357
H -1.684976 4.584852 2.552441
H -1.089190 3.318308 4.599019
H -0.838673 0.850994 4.504661
H -1.222923 -0.353509 2.364942
C 1.002634 1.684598 -1.943403
O 0.373638 1.892684 -0.853759
C 2.150364 2.572125 -2.340243
N 0.594311 0.650888 -2.657034
C -0.415473 -0.178572 -2.018022
C -0.682555 0.441678 -0.686355
C -1.822668 0.668698 -0.014774
C -3.152023 0.123389 -0.355601
C -3.425878 -1.225105 -0.626834
C -4.711249 -1.648634 -0.955104
C -5.754357 -0.734500 -0.999827

C -5.515260 0.603581 -0.697531
C -4.233764 1.017563 -0.370852
H 1.156235 0.285144 -3.416018
H -1.340062 -0.185923 -2.616282
H -0.046796 -1.208785 -1.935956
H -4.885646 -2.703164 -1.156442
H -6.757028 -1.072629 -1.253891
H -6.329152 1.325629 -0.713696
H -4.046786 2.061911 -0.123138
C 3.414974 1.930931 -1.738732
C 2.272213 2.657490 -3.860544
C 1.945908 3.961319 -1.736704
H 3.081401 3.354791 -4.110332
H 2.538916 1.694302 -4.318654
H 1.351152 3.028478 -4.330071
H 2.804610 4.590680 -2.002529
H 1.037125 4.442774 -2.122722
H 1.880020 3.908019 -0.643578
H 4.275607 2.553846 -2.017376
H 3.354107 1.883539 -0.642630
H 3.576154 0.913219 -2.115781
Br -2.076805 -2.545555 -0.472093

Reaction 20

reactant

30
Energy: -993.944134328
C -4.118795 -0.047008 0.188139
O -4.705282 0.009780 -0.883556
C -4.885460 -0.101572 1.513708
N -2.759005 -0.059157 0.239153
H -2.253387 -0.108107 1.111519
C -1.995940 -0.002100 -0.998462
C -0.567832 -0.000151 -0.734823
H -2.272461 -0.854738 -1.638132
H -2.290037 0.895427 -1.564335
C 0.622924 -0.011055 -0.505648
C 2.007318 -0.000895 -0.242149
C -3.997583 -0.189765 2.750824
C -5.737002 1.167537 1.583869
C -5.798382 -1.327250 1.449996
H -4.625019 -0.229367 3.651549
H -3.377508 -1.098250 2.751669
H -3.343564 0.688432 2.855295
H -6.444899 -1.363411 2.338070
H -6.429314 -1.289115 0.554830
H -5.214266 -2.257948 1.417801
H -6.379697 1.142898 2.475128
H -5.107440 2.066709 1.644440
H -6.370639 1.253527 0.693823
C 2.868473 -1.069201 -0.140882
C 4.202804 -0.684546 0.134323
C 4.350532 0.670117 0.241245
S 2.861841 1.504901 0.006815
H 5.257005 1.229395 0.444839
H 5.024515 -1.386028 0.248544
H 2.534694 -2.095621 -0.265183

I_c

65
Energy: -4594.57402180
Cu 0.719605 0.159380 -0.540676
Cl 1.416806 -0.338490 -2.554791
C 1.865607 1.673174 -0.648261
O -0.488761 -1.459369 -0.416319
S -1.322682 -1.877170 0.764402
O -2.046351 -0.769506 1.393680
C -2.631506 -2.852601 -0.071692
O -0.665627 -2.835807 1.647274
F -3.483706 -3.315504 0.826579
F -3.304703 -2.080346 -0.931755
F -2.102222 -3.863203 -0.738462
C 1.221736 2.879570 -0.450948
C 2.011809 4.027160 -0.332825
C 3.393217 3.949518 -0.437922
C 4.004981 2.718865 -0.664329
C 3.238747 1.561060 -0.775690
C -0.959244 3.020369 2.233472
O -0.080909 3.864830 2.365726

C -2.425113 3.431169 2.066275
N -0.677542 1.686116 2.210500
C 0.670425 1.231711 2.413397
C 1.151031 0.227092 1.438356
C 1.879342 -0.770343 1.252288
C 2.678029 -1.850688 0.922785
H 0.142516 2.951853 -0.353554
H 1.517859 4.976064 -0.130029
H 3.998280 4.849510 -0.345095
H 5.087141 2.650562 -0.765885
H 3.710644 0.603100 -0.987311
H -1.393575 0.983761 2.039953
H 1.313508 2.123521 2.352280
H 0.798320 0.801190 3.419130
C -3.413836 2.273175 2.159930
C -2.734131 4.458200 3.156178
C -2.535466 4.095381 0.690671
O -3.330472 1.099785 -1.075770
C -4.538900 0.650340 -1.703754
C -5.580788 0.574957 -0.619096
C -2.125825 0.855748 -1.580410
O -1.166806 1.218580 -0.915479
C -2.018169 0.181078 -2.909651
H -4.812090 1.366500 -2.492218
H -4.380841 -0.331868 -2.164568
H -6.541562 0.246750 -1.031818
H -5.263689 -0.139577 0.150460
H -5.718046 1.553824 -0.144692
H -2.724266 0.602313 -3.634487
H -0.993361 0.271810 -3.277030
H -2.239676 -0.887386 -2.794814
H -3.331808 1.740333 3.118109
H -4.437677 2.668995 2.092684
H -3.290489 1.548707 1.344092
H -3.556068 4.475554 0.537586
H -1.834544 4.936899 0.611100
H -2.317341 3.374313 -0.109029
H -3.748205 4.858240 3.015774
H -2.686236 4.003580 4.155809
H -2.017904 5.286519 3.124703
S 4.387839 -1.643419 0.556147
C 4.542494 -3.317455 0.244292
C 3.362802 -4.007795 0.399059
C 2.297673 -3.180312 0.786308
H 1.271008 -3.491136 0.971249
H 3.274356 -5.077772 0.237924
H 5.508271 -3.722971 -0.039445

I_1

51
Energy: -4287.05081086
Cu 0.025001 0.029780 0.082096
Cl 0.005404 0.457695 2.170567
C 1.897131 0.103825 0.301426
O -1.961441 0.225800 -0.234722
S -2.021399 1.523188 -1.013812
O -2.673578 1.423105 -2.307135
C -3.103901 2.556090 0.045947
O -0.691984 2.162526 -0.962502
F -3.240526 3.750161 -0.509124
F -4.291353 1.982449 0.154396
F -2.565132 2.685484 1.243771
C 2.669062 -1.019719 0.519728
C 4.054674 -0.857234 0.454520
C 4.612479 0.392727 0.200307
C 3.793897 1.501175 0.009312
C 2.405095 1.369944 0.073728
H 2.224726 -1.997615 0.708598
H 4.694587 -1.725360 0.605326
H 5.694732 0.505132 0.163478
H 4.226606 2.484036 -0.172220
H 1.752603 2.234961 -0.041868
C -0.425827 -3.211649 0.823799
O 0.655730 -3.575035 0.375366
C -0.845736 -3.505503 2.261507
N -1.329324 -2.581224 0.013338
C -0.973210 -2.304216 -1.356846
C -0.026394 -1.175672 -1.540794
C 0.660922 -0.448165 -2.274946
C 1.488875 0.413066 -2.975230
H -2.130274 -2.096015 0.397889

H -0.511775 -3.201903 -1.786566
H -1.887896 -2.071231 -1.914901
C -2.075308 -2.723081 2.715435
C -1.145364 -5.008434 2.309401
C 0.340678 -3.187604 3.171175
H 0.101444 -3.469113 4.205853
H 1.230526 -3.743895 2.854360
H 0.576828 -2.114219 3.154824
H -1.409506 -5.302766 3.334588
H -1.988775 -5.268150 1.653517
H -0.270211 -5.589825 1.993949
H -2.279124 -2.948181 3.770752
H -1.925638 -1.636215 2.636824
H -2.977589 -3.005217 2.152917
S 3.166787 0.014741 -3.286673
C 3.408776 1.536842 -4.027730
C 2.279093 2.321976 -4.033650
C 1.179128 1.684832 -3.436377
H 0.189356 2.113559 -3.299634
H 2.248709 3.321092 -4.457365
H 4.381961 1.785974 -4.438205

TS_{rc}^{A}

51
Energy: -4287.02494491
Cu -0.612168 -0.304130 -0.883804
Cl -0.343833 -1.050774 -2.867511
C -2.278562 -1.183691 -0.927862
O 0.907730 0.922736 -0.975232
S 1.783591 1.568586 0.070467
O 2.445530 0.590756 0.945008
C 3.127430 2.204729 -1.003252
O 1.193116 2.736984 0.704614
F 4.026290 2.819722 -0.247153
F 3.716807 1.203271 -1.642770
F 2.642521 3.060804 -1.886152
C -2.386239 -2.506955 -0.538178
C -3.656096 -3.081669 -0.499292
C -4.777327 -2.341032 -0.860531
C -4.635199 -1.019417 -1.268380
C -3.372133 -0.428695 -1.314265
H -1.504377 -3.084293 -0.268856
H -3.761437 -4.120534 -0.189099
H -5.764498 -2.799085 -0.833100
H -5.506831 -0.436984 -1.563106
H -3.263349 0.605690 -1.640304
C 1.479223 -1.943283 1.287469
O 0.421312 -1.898867 0.630487
C 2.741128 -2.573286 0.746354
N 1.399762 -1.527628 2.571807
C 0.099155 -0.977999 2.919035
C -0.556421 -0.419487 1.731488
C -1.125700 0.347114 0.903742
C -1.965206 1.511621 0.916754
H 2.231085 -1.147016 3.007787
H -0.524674 -1.761098 3.374709
H 0.221524 -0.166726 3.649591
C 2.911375 -2.191689 -0.723119
C 3.976114 -2.174853 1.548883
C 2.511761 -4.089111 0.865161
H 3.395130 -4.616449 0.481588
H 2.359025 -4.394298 1.909949
H 1.641668 -4.404351 0.275667
H 4.860072 -2.645355 1.099690
H 4.127808 -1.088238 1.529671
H 3.925793 -2.524482 2.590804
H 3.775431 -2.731331 -1.133373
H 2.027015 -2.455533 -1.316032
H 3.087745 -1.115920 -0.835649
S -3.209134 1.693496 2.124609
C -3.698816 3.199601 1.443621
C -2.945950 3.538714 0.355069
C -1.960489 2.570347 0.043255
H -1.238014 2.657491 -0.765246
H -3.093540 4.453490 -0.211501
H -4.527548 3.745257 1.880911

TS_{ar}^{B}

51
Energy: -4287.03732147

Cu -0.148167 -0.597659 -0.549293
Cl 0.189880 -1.014778 -2.650943
C -2.048613 -0.991154 -0.708703
O 1.675057 0.134417 -0.337101
S 2.005831 1.520078 0.165890
O 2.687145 1.480309 1.463578
C 3.293296 1.994603 -1.050602
O 0.913390 2.473890 0.000497
F 3.758454 3.196610 -0.747238
F 4.287704 1.117778 -1.007466
F 2.777182 2.014544 -2.264321
C -2.484208 -2.168589 -0.114005
C -3.648701 -2.766646 -0.591516
C -4.363768 -2.175446 -1.627188
C -3.918235 -0.984166 -2.199243
C -2.754274 -0.381827 -1.742640
H -1.915790 -2.619375 0.696647
H -3.997002 -3.694371 -0.141165
H -5.278354 -2.641175 -1.989636
H -4.472595 -0.524919 -3.015528
H -2.398471 0.548268 -2.182223
C 1.615429 -2.081804 1.202360
O 0.569009 -2.459935 0.656054
C 2.959419 -2.664730 0.821677
N 1.577157 -1.141552 2.179760
C 0.330846 -0.463212 2.478068
C -0.512025 -0.142562 1.290771
C -1.577746 0.353832 0.830053
C -2.563986 1.371257 0.791486
H 2.408659 -0.567120 2.296449
C -0.507176 -1.257574 3.477439
H 0.614660 0.507320 2.914437
C 4.155255 -1.982336 1.471791
H 2.905376 -3.707271 1.182069
C 3.100304 -2.700094 -0.699702
H 5.079190 -2.484174 1.160484
H 4.235803 -0.932790 1.151760
H 4.118905 -2.015066 2.568706
H 3.999244 -3.266228 -0.973514
H 2.231695 -3.168238 -1.174382
H 3.192746 -1.682923 -1.101920
H -1.431500 -0.722526 3.725329
H -0.765267 -2.236231 3.057767
H 0.072525 -1.413305 4.394111
S -4.269185 1.048140 0.979270
C -4.615322 2.729633 0.861170
C -3.493178 3.491126 0.672440
C -2.311764 2.716304 0.629677
H -1.301032 3.091149 0.478403
H -3.514703 4.571858 0.568480
H -5.641807 3.070331 0.945379

I_2

51
Energy: -4287.09025518
Cu 0.241650 -1.452147 -1.966082
Cl 0.822763 -3.544564 -2.343064
C 2.076922 0.372609 -0.724903
O -0.175328 -1.679315 -0.149509
S -1.696937 -1.646711 0.028302
O -2.366358 -1.821199 -1.265282
C -1.950193 -3.214349 0.947925
O -2.144652 -0.562401 0.887629
F -3.247208 -3.359742 1.176780
F -1.510050 -4.224379 0.229025
F -1.304068 -3.163593 2.099184
C 2.690222 -0.498336 -1.635880
C 3.775969 -1.277215 -1.244907
C 4.268641 -1.178625 0.049356
C 3.682821 -0.290144 0.951137
C 2.594695 0.481190 0.569532
H 2.369982 -0.509482 -2.679174
H 4.227426 -1.963370 -1.958367
H 5.113541 -1.790537 0.359328
H 4.070006 -0.211132 1.965442
H 2.112563 1.149071 1.282443
C -1.081492 -0.720648 -4.188550
O 0.051040 -1.113523 -3.796251
C -1.670758 -1.407583 -5.405331
N -1.786830 0.204544 -3.548102
C -1.417546 0.849181 -2.288939

C -0.109890 0.401546 -1.761280
C 0.837466 1.083797 -1.105825
C 0.634480 2.473591 -0.696051
H -2.722808 0.389660 -3.886113
H -1.358924 1.936807 -2.459796
H -2.234955 0.659913 -1.576987
C -2.825354 -0.635166 -6.035730
C -0.565037 -1.631580 -6.436456
C -2.166928 -2.762338 -4.869899
H -2.593701 -3.341631 -5.699805
H -1.344443 -3.336118 -4.422240
H -2.942261 -2.629852 -4.102264
H -0.971607 -2.203414 -7.280556
H -0.176176 -0.680798 -6.825944
H 0.266704 -2.196741 -6.002641
H -3.152651 -1.160540 -6.941586
H -3.707756 -0.579914 -5.380542
H -2.533054 0.381936 -6.332536
C -0.479431 3.018619 -0.105318
C -0.339177 4.401326 0.178680
C 0.879290 4.892083 -0.195784
S 1.883653 3.676131 -0.895084
H 1.252830 5.904968 -0.092185
H -1.105919 5.003961 0.657521
H -1.343442 2.414907 0.168116

TS_{rc}^{B}

51
Energy: -4287.06228511
Cu 0.052348 -1.326683 0.388596
Cl 0.837153 -3.299100 -0.199332
C 0.131501 1.930654 1.463545
O -1.715396 -0.850117 0.917743
S -2.644292 -0.563258 -0.253596
O -1.954920 -0.705342 -1.538254
C -3.771087 -2.005940 -0.157606
O -3.464553 0.619427 -0.043330
F -4.656553 -1.934296 -1.139812
F -3.070935 -3.121787 -0.280421
F -4.404802 -2.010592 1.003630
C 0.237857 0.992711 2.501188
C 0.157312 1.386650 3.825840
C -0.035498 2.728389 4.146791
C -0.156324 3.668972 3.129232
C -0.077753 3.275179 1.800672
H 0.357541 -0.060597 2.263567
H 0.231143 0.638729 4.612725
H -0.103876 3.036226 5.188613
H -0.319828 4.717772 3.369827
H -0.184393 4.015679 1.010119
C 2.794187 -0.890128 -0.371612
O 2.051724 -0.540235 0.587448
C 4.163348 -1.472984 -0.106973
N 2.381884 -0.627931 -1.608527
C 1.027057 -0.115158 -1.773876
C 0.560884 0.336674 -0.428964
C 0.250524 1.550938 0.053699
C -0.090579 2.532979 -0.999317
H 2.853279 -1.048216 -2.399289
H 1.031628 0.745104 -2.456971
H 0.372518 -0.890950 -2.196554
C 4.636612 -2.328343 -1.280292
C 5.103785 -0.276280 0.095843
C 4.106730 -2.319081 1.164012
H 6.113433 -0.646151 0.317754
H 5.159401 0.352734 -0.803283
H 4.774117 0.346594 0.937079
H 5.104553 -2.730344 1.364523
H 3.800222 -1.718318 2.027712
H 3.394399 -3.146544 1.053450
H 5.593555 -2.796619 -1.018419
H 3.920671 -3.131019 -1.504068
H 4.819825 -1.736157 -2.189472
S 1.143576 3.554007 -1.701038
C -0.020939 4.298269 -2.743030
C -1.271870 3.789271 -2.553904
C -1.314733 2.772707 -1.559963
H -2.208108 2.224928 -1.259774
H -2.142668 4.125334 -3.109745
H 0.290860 5.081200 -3.425757

Reaction 21

reactant

30
Energy: -993.943068608
C -4.082687 -0.035117 0.185732
O -4.670627 0.029493 -0.884785
C -4.848393 -0.102666 1.511454
N -2.723084 -0.042927 0.236093
H -2.217463 -0.101868 1.107800
C -1.959009 0.025032 -1.000532
C -0.531269 0.048294 -0.729815
H -2.222029 -0.831430 -1.640780
H -2.266218 0.918682 -1.565213
C 0.655905 0.074630 -0.490764
C 2.050348 0.086030 -0.215636
C -3.959721 -0.204337 2.746968
C -5.698391 1.166668 1.595216
C -5.762839 -1.326470 1.436236
H -4.586477 -0.253489 3.647715
H -3.339774 -1.112862 2.737483
H -3.305384 0.672526 2.860339
H -6.408939 -1.370704 2.324258
H -6.394107 -1.278698 0.541791
H -5.179897 -2.257527 1.394429
H -6.340283 1.134026 2.486816
H -5.067597 2.064407 1.664063
H -6.332768 1.262263 0.706667
C 2.859272 -1.091939 -0.108604
C 4.162772 -0.812593 0.161279
S 4.440849 0.889835 0.291793
C 2.785185 1.232051 -0.019102
H 4.985406 -1.506319 0.291707
H 2.461799 -2.095252 -0.233341
H 2.425082 2.254113 -0.046587

I_c

65
Energy: -4594.57135003
Cu 0.735001 0.125909 -0.514200
Cl 1.396980 -0.434321 -2.519897
C 1.945743 1.583574 -0.693657
O -0.522837 -1.446219 -0.372379
S -1.347041 -1.873695 0.813010
O -2.070298 -0.772162 1.453835
C -2.658446 -2.838137 -0.031209
O -0.678585 -2.835972 1.681073
F -3.516962 -3.300856 0.861002
F -3.322357 -2.055567 -0.889452
F -2.132865 -3.847957 -0.701983
C 1.356812 2.814636 -0.480237
C 2.200668 3.925419 -0.377365
C 3.574410 3.787822 -0.517086
C 4.126935 2.532649 -0.761398
C 3.307974 1.409468 -0.856725
C -1.061480 3.024108 2.122513
O -0.200472 3.896738 2.085608
C -2.545611 3.389620 2.050571
N -0.745053 1.703949 2.258309
C 0.625647 1.308282 2.426608
C 1.116854 0.260659 1.508838
C 1.831461 -0.724557 1.268583
C 2.675371 -1.788589 0.903945
H 0.285381 2.939908 -0.351312
H 1.752761 4.893449 -0.159554
H 4.219963 4.660794 -0.440204
H 5.201879 2.419574 -0.896157
H 3.726490 0.430454 -1.084312
H -1.440528 0.969068 2.148968
H 1.232093 2.216353 2.286475
H 0.807738 0.942904 3.449890
C -3.490339 2.201248 2.188609
C -2.805432 4.383305 3.185710
C -2.771617 4.081222 0.704619
O -3.281508 1.120165 -1.039192
C -4.486645 0.685237 -1.685468
C -5.549913 0.636917 -0.620734
C -2.075137 0.885305 -1.542424
O -1.117041 1.245795 -0.873157
C -1.966928 0.217206 -2.874510

H -4.733693 1.399089 -2.484533
H -4.335463 -0.303558 -2.134386
H -6.508462 0.325696 -1.051394
H -5.263500 -0.080037 0.158744
H -5.677186 1.621897 -0.155702
H -2.696798 0.612989 -3.589388
H -0.952095 0.337703 -3.258847
H -2.150611 -0.858177 -2.751458
H -3.353543 1.675046 3.144178
H -4.528675 2.562498 2.163611
H -3.370506 1.479541 1.370082
H -3.814713 4.421982 0.628604
H -2.110784 4.950700 0.602068
H -2.577020 3.390464 -0.126482
H -3.841235 4.747412 3.133268
H -2.663767 3.910188 4.167920
H -2.125196 5.239675 3.116614
C 4.041128 -1.608018 0.752455
S 4.817533 -3.056820 0.298392
C 3.312681 -3.925511 0.297694
C 2.262847 -3.137789 0.640388
H 1.228224 -3.460865 0.723923
H 3.305185 -4.978149 0.039376
H 4.597663 -0.690031 0.909023

I_1

51
Energy: -4287.04935115
Cu 0.008485 0.074915 0.017625
Cl -0.022782 0.382265 2.126560
C 1.872863 0.131627 0.317975
O -1.994408 0.264946 -0.307079
S -2.009623 1.610631 -0.999044
O -2.637336 1.623740 -2.307408
C -3.079197 2.616165 0.100979
O -0.656872 2.195758 -0.879920
F -3.138569 3.851546 -0.370070
F -4.294943 2.094441 0.126460
F -2.581334 2.634279 1.323229
C 2.623146 -1.017944 0.464614
C 4.010769 -0.877928 0.388560
C 4.589785 0.374999 0.202040
C 3.790788 1.508652 0.093370
C 2.400818 1.400771 0.168937
H 2.162398 -1.996656 0.605355
H 4.635466 -1.765039 0.483084
H 5.673396 0.469584 0.157365
H 4.240592 2.492081 -0.034376
H 1.763161 2.282038 0.113818
C -0.442232 -3.229024 0.752713
O 0.627240 -3.598852 0.280659
C -0.845294 -3.550505 2.189403
N -1.346968 -2.567464 -0.030387
C -1.018956 -2.288412 -1.408041
C -0.065138 -1.175357 -1.610541
C 0.651755 -0.396582 -2.243165
C 1.529780 0.490315 -2.893680
H -2.139494 -2.083795 0.373055
H -0.578371 -3.190058 -1.851704
H -1.943710 -2.042921 -1.943050
C -2.071568 -2.780944 2.672567
C -1.141424 -5.054814 2.211569
C 0.351143 -3.248994 3.091647
H 0.125080 -3.553416 4.122775
H 1.238309 -3.796294 2.752343
H 0.584740 -2.175084 3.095641
H -1.393798 -5.369047 3.233783
H -1.991641 -5.303712 1.560306
H -0.268839 -5.628162 1.875332
H -2.263741 -3.028263 3.725080
H -1.925239 -1.692532 2.614518
H -2.978719 -3.053764 2.113350
C 2.861852 0.188541 -3.098260
S 3.681453 1.470634 -3.870620
C 2.236016 2.432594 -3.931019
C 1.173327 1.790938 -3.381701
H 0.171450 2.200855 -3.289156
H 2.267685 3.419668 -4.377652
H 3.379539 -0.714883 -2.794149

TS_{rc}^{A}

51
Energy: -4287.02598798
Cu -0.532572 -0.342772 -0.845751
Cl -0.226416 -1.067367 -2.833899
C -2.184903 -1.232435 -0.927687
O 0.941329 0.943171 -0.904739
S 1.753827 1.634876 0.158509
O 2.555767 0.705948 0.965540
C 2.975944 2.503915 -0.897113
O 1.033118 2.679077 0.875832
F 3.794633 3.200378 -0.121340
F 3.683927 1.625804 -1.593184
F 2.358684 3.325877 -1.728108
C -2.338711 -2.502427 -0.402749
C -3.625649 -3.037004 -0.339356
C -4.714929 -2.310330 -0.811474
C -4.524419 -1.046254 -1.360162
C -3.244989 -0.496044 -1.430095
H -1.480298 -3.062585 -0.035867
H -3.770767 -4.030865 0.082382
H -5.715434 -2.736583 -0.761923
H -5.370895 -0.478920 -1.744367
H -3.093499 0.492549 -1.864136
C 1.738469 -1.913453 1.199416
O 0.651268 -1.913847 0.589210
C 3.009991 -2.449046 0.584300
N 1.694990 -1.528489 2.493735
C 0.391933 -1.038049 2.911561
C -0.368070 -0.536697 1.757191
C -1.066805 0.172077 0.983682
C -2.107177 1.180408 1.081743
H 2.525803 -1.114467 2.899074
H -0.164159 -1.842442 3.415084
H 0.520113 -0.208571 3.619591
C 3.103596 -1.985036 -0.868672
C 4.251825 -2.021920 1.361335
C 2.866047 -3.978831 0.632987
H 3.760967 -4.437246 0.191923
H 2.770029 -4.343031 1.665644
H 1.991959 -4.311335 0.059458
H 5.141337 -2.423970 0.859818
H 4.347493 -0.928617 1.387350
H 4.258291 -2.419871 2.386988
H 3.980560 -2.453222 -1.335452
H 2.213257 -2.267560 -1.443838
H 3.216250 -0.896482 -0.930257
C -3.325906 0.901167 1.644847
S -4.424785 2.211713 1.453313
C -3.230848 3.148182 0.622475
C -2.053331 2.478029 0.486833
H -1.154937 2.886124 0.030761
H -3.464648 4.155831 0.298182
H -3.646862 -0.039965 2.079899

TS_{ar}^{B}

51
Energy: -4287.03677237
Cu -0.143567 -0.643309 -0.513775
Cl 0.191981 -1.090528 -2.608413
C -2.010661 -1.178201 -0.644372
O 1.653112 0.167298 -0.343388
S 1.957984 1.572069 0.120626
O 2.641118 1.581827 1.418508
C 3.240531 2.032417 -1.106406
O 0.849887 2.501651 -0.070669
F 3.687757 3.248704 -0.835976
F 4.247989 1.171352 -1.035400
F 2.728395 2.010070 -2.321570
C -2.356091 -2.360420 -0.004712
C -3.495482 -3.041165 -0.433127
C -4.271780 -2.526562 -1.465144
C -3.912049 -1.329438 -2.084449
C -2.774908 -0.645863 -1.678073
H -1.742732 -2.750589 0.804243
H -3.774405 -3.973759 0.054220
H -5.164755 -3.057842 -1.789475
H -4.511353 -0.930653 -2.900804
H -2.486461 0.287692 -2.157159
C 1.719880 -1.992500 1.241677
O 0.681596 -2.427188 0.721787

C 3.082637 -2.520604 0.848052
N 1.654326 -1.040507 2.205654
C 0.379623 -0.421566 2.515952
C -0.496651 -0.152497 1.342000
C -1.570538 0.250950 0.820721
C -2.616880 1.231777 0.736835
H 2.455491 -0.418510 2.290192
C -0.399911 -1.242826 3.540318
H 0.622957 0.567103 2.936241
C 4.257252 -1.742302 1.426265
H 3.097489 -3.544287 1.262973
C 3.182322 -2.627612 -0.672841
H 5.197615 -2.209488 1.110248
H 4.270870 -0.706987 1.054716
H 4.255998 -1.723383 2.524136
H 4.106742 -3.151601 -0.945611
H 2.331607 -3.171968 -1.095457
H 3.199556 -1.629385 -1.129370
H -1.347368 -0.753792 3.794850
H -0.611462 -2.240453 3.139935
H 0.201262 -1.352056 4.449845
C -3.960410 0.949259 0.805316
S -4.898877 2.379859 0.746667
C -3.483368 3.370376 0.611618
C -2.341458 2.631193 0.619673
H -1.330752 3.023669 0.529582
H -3.585899 4.446430 0.530516
H -4.425951 -0.028831 0.873198

I_2

51
Energy: -4287.08925464
Cu 0.237985 -1.449546 -1.943008
Cl 0.822884 -3.541916 -2.322850
C 2.063209 0.380591 -0.690970
O -0.192972 -1.682294 -0.130085
S -1.715591 -1.652285 0.036449
O -2.375857 -1.826905 -1.261860
C -1.973157 -3.220917 0.952989
O -2.172015 -0.569323 0.893048
F -3.271455 -3.367776 1.173809
F -1.527731 -4.229890 0.235830
F -1.334200 -3.170905 2.108325
C 2.677618 -0.499881 -1.592473
C 3.763741 -1.274901 -1.194428
C 4.257158 -1.163934 0.098381
C 3.672069 -0.265127 0.990420
C 2.584541 0.503337 0.601180
H 2.359764 -0.520483 -2.636352
H 4.214537 -1.967862 -1.901780
H 5.101912 -1.772964 0.414387
H 4.059938 -0.175019 2.003621
H 2.107325 1.181713 1.307007
C -1.068476 -0.713065 -4.174325
O 0.060843 -1.106112 -3.774476
C -1.647986 -1.396453 -5.397894
N -1.780088 0.209927 -3.536838
C -1.424850 0.846004 -2.269540
C -0.116976 0.403547 -1.735023
C 0.825368 1.089605 -1.080398
C 0.644494 2.497837 -0.684712
H -2.713998 0.393647 -3.881321
H -1.374340 1.934606 -2.435996
H -2.246471 0.644576 -1.565534
C -2.797277 -0.622214 -6.035762
C -0.533897 -1.617843 -6.420510
C -2.148656 -2.752637 -4.870363
H -2.568207 -3.329755 -5.705464
H -1.330060 -3.327561 -4.417050
H -2.930649 -2.622196 -4.109181
H -0.933540 -2.187356 -7.269479
H -0.141754 -0.666055 -6.804259
H 0.294152 -2.184298 -5.981341
H -3.116899 -1.144924 -6.945900
H -3.685237 -0.568884 -5.387961
H -2.502576 0.395798 -6.327033
C -0.557277 3.024308 -0.115590
C -0.465673 4.352301 0.174699
S 1.089464 4.988966 -0.231304
C 1.624445 3.453400 -0.791419
H -1.210659 4.992933 0.632827

H -1.422818 2.407856 0.120739
H 2.629363 3.325397 -1.180410

TS_{rc}^{B}

51
Energy: -4287.06222403
Cu 0.375772 -1.337711 0.350868
Cl 1.518742 -3.088552 -0.350267
C -0.148408 1.821892 1.567607
O -1.436457 -1.257528 0.926271
S -2.461483 -1.044244 -0.178460
O -1.828788 -0.892574 -1.490454
C -3.261275 -2.693032 -0.227766
O -3.497288 -0.095653 0.200195
F -4.196692 -2.696469 -1.165413
F -2.355740 -3.615817 -0.510710
F -3.813873 -2.965135 0.942812
C 0.133896 0.866205 2.554904
C 0.017444 1.176130 3.898891
C -0.389114 2.449344 4.290902
C -0.680156 3.406674 3.324760
C -0.562398 3.097780 1.976573
H 0.424666 -0.138662 2.262591
H 0.231680 0.413694 4.645018
H -0.487617 2.689963 5.347897
H -1.005437 4.402193 3.621095
H -0.790801 3.853367 1.226909
C 2.951391 -0.330949 -0.450660
O 2.184224 -0.180888 0.539640
C 4.417287 -0.635518 -0.243050
N 2.457451 -0.102324 -1.665892
C 1.017818 0.106514 -1.773355
C 0.520388 0.426700 -0.403780
C 0.001769 1.537809 0.137316
C -0.567498 2.502454 -0.841139
H 2.977132 -0.395772 -2.483407
H 0.803438 0.958233 -2.431946
H 0.533298 -0.786790 -2.193424
C 4.569241 -1.525680 0.989568
C 5.016501 -1.330566 -1.463723
C 5.105532 0.716318 -0.005897
H 6.055024 -1.607635 -1.243925
H 4.468729 -2.250916 -1.708344
H 5.050358 -0.677645 -2.348823
H 6.174163 0.547893 0.181380
H 5.011501 1.377872 -0.878164
H 4.681026 1.227406 0.867628
H 5.634336 -1.738884 1.147901
H 4.177905 -1.032375 1.886425
H 4.030472 -2.472612 0.858500
C 0.184867 3.421205 -1.525308
S -0.797544 4.420028 -2.530247
C -2.229253 3.585842 -2.031484
C -1.961475 2.594780 -1.136422
H -2.707312 1.929545 -0.701821
H -3.189710 3.871922 -2.445117
H 1.257312 3.578457 -1.463507

Reaction 22

reactant

27
Energy: -594.590010232
C -3.998308 -0.343451 0.200265
O -4.601287 -0.401225 -0.858891
C -4.683212 -0.312388 1.553730
N -2.637354 -0.277651 0.243506
H -2.141081 -0.224813 1.122478
C -1.869474 -0.237606 -0.991133
C -0.445516 -0.142124 -0.714122
H -2.091405 -1.136670 -1.586765
H -2.206081 0.613514 -1.603679
C 0.737826 -0.062366 -0.467721
C 2.133598 0.029467 -0.183525
C 2.917042 -1.129794 -0.094093
C 4.274281 -1.037606 0.182374
C 4.866728 0.207938 0.373600
C 4.096159 1.364468 0.286849
C 2.738483 1.279386 0.010174
H 2.444958 -2.098905 -0.245938

H 4.874513 -1.943411 0.248875
H 5.931399 0.277502 0.590081
H 4.556909 2.339645 0.434926
H 2.128152 2.178005 -0.061402
H -4.095707 -0.895499 2.279119
H -4.657258 0.728459 1.913532
C -6.113970 -0.809365 1.478459
H -6.612857 -0.722440 2.450434
H -6.679531 -0.233420 0.738562
H -6.149846 -1.859651 1.165632

I_c

62
Energy: -4195.21632912
Cu -0.845654 0.270026 0.563582
Cl -1.554913 -0.370691 2.519579
C -1.962104 1.785552 0.855159
O 0.307208 -1.379843 0.333177
S 1.137940 -1.877358 -0.817519
O 2.151682 -0.918943 -1.272501
C 2.109420 -3.179861 0.032991
O 0.373627 -2.578924 -1.842215
F 2.990458 -3.697901 -0.806596
F 2.771382 -2.654779 1.068415
F 1.312089 -4.133465 0.480785
C -1.285002 2.983000 0.732773
C -2.051584 4.152625 0.692193
C -3.434525 4.100356 0.801969
C -4.073949 2.873005 0.956482
C -3.334195 1.692040 0.992509
C 1.651812 2.817425 -1.519363
O 1.030210 3.852044 -1.310946
C 3.143104 2.719202 -1.308593
N 1.066811 1.688218 -2.018138
C -0.350785 1.643695 -2.238233
C -1.081836 0.617231 -1.467478
C -1.927136 -0.276252 -1.327770
C -2.957849 -1.224532 -1.108868
C -2.668900 -2.584322 -0.898108
C -3.711765 -3.470561 -0.678257
C -5.031019 -3.021457 -0.676862
C -5.322407 -1.676639 -0.898976
C -4.292018 -0.774666 -1.109667
H -0.205606 3.046656 0.624346
H -1.534702 5.101334 0.555625
H -4.018487 5.018609 0.775351
H -5.156336 2.823997 1.068062
H -3.823726 0.732412 1.146823
H 1.580952 0.808212 -1.988586
H -0.739778 2.639023 -1.973034
H -0.583033 1.467175 -3.300076
H -1.632873 -2.917239 -0.923698
H -3.494909 -4.523027 -0.509026
H -5.841728 -3.727332 -0.504203
H -6.354864 -1.333446 -0.904935
H -4.496100 0.280711 -1.284409
H 3.496918 1.694659 -1.490093
C 3.867923 3.731844 -2.185002
H 3.327386 2.933948 -0.247554
O 3.185265 0.727829 1.105289
C 4.311380 -0.001767 1.615268
C 5.203508 -0.302200 0.438986
C 1.986295 0.684192 1.667147
O 1.068702 1.236138 1.074137
C 1.820283 -0.018833 2.976413
H 4.813763 0.620075 2.370340
H 3.974733 -0.927195 2.094939
H 6.085254 -0.866025 0.765026
H 4.654577 -0.894963 -0.303404
H 5.541283 0.624377 -0.040481
H 2.662107 0.162494 3.653452
H 0.883426 0.300370 3.437230
H 1.743678 -1.098973 2.791986
H 4.946630 3.725309 -1.988829
H 3.718598 3.514241 -3.250668
H 3.484795 4.740241 -1.990903

I_1

48
Energy: -3887.69370757

Cu 0.065110 -0.178551 -0.022752
Cl 0.065920 -0.243817 2.110189
C 1.930461 -0.165198 0.284445
O -1.954667 0.081430 -0.304886
S -1.936552 1.521038 -0.763941
O -2.586697 1.777328 -2.036087
C -2.946515 2.366670 0.511827
O -0.559932 2.031834 -0.580816
F -2.974524 3.663513 0.250625
F -4.178395 1.884069 0.486300
F -2.420794 2.168815 1.706500
C 2.677899 -1.325661 0.284408
C 4.064348 -1.179101 0.213229
C 4.643911 0.087411 0.178322
C 3.846795 1.226200 0.217592
C 2.456743 1.112547 0.291453
H 2.210705 -2.310431 0.325028
H 4.688223 -2.071547 0.194664
H 5.727350 0.185743 0.137561
H 4.296968 2.217578 0.206438
H 1.819720 1.993791 0.350709
C -0.504081 -3.399803 0.530483
O 0.587146 -3.803752 0.149030
C -0.986047 -3.613431 1.945304
N -1.345381 -2.735789 -0.319640
C -0.912886 -2.432416 -1.661567
C -0.021319 -1.256258 -1.781088
C 0.652764 -0.381279 -2.326219
C 1.500975 0.598122 -2.902272
C 0.984094 1.843012 -3.296910
C 1.835749 2.789519 -3.845804
C 3.190984 2.504822 -4.002127
C 3.706318 1.269345 -3.613889
C 2.866137 0.313374 -3.065630
H -2.152970 -2.238825 0.036368
H -0.373790 -3.304264 -2.052567
H -1.797241 -2.255348 -2.285182
H -0.073688 2.052789 -3.151599
H 1.442116 3.757183 -4.149149
H 3.853628 3.255277 -4.430301
H 4.766371 1.055103 -3.734349
H 3.250493 -0.650710 -2.735858
C -2.370205 -3.099173 2.300012
H -0.900491 -4.693346 2.131453
H -0.225690 -3.140495 2.583581
H -2.605165 -3.328077 3.345410
H -2.434439 -2.007010 2.191759
H -3.151918 -3.561236 1.682125

TS_{rc}^{A}

48
Energy: -3887.66980015
Cu 0.643533 -0.994486 -1.025741
Cl 1.506268 -2.914255 -0.652290
C 2.385571 -0.346166 -1.293293
O -1.182610 -1.570025 -0.480443
S -2.596226 -1.061388 -0.380952
O -3.506041 -1.759498 -1.292806
C -3.046116 -1.638179 1.299706
O -2.712602 0.395384 -0.332671
F -4.287300 -1.268131 1.572134
F -2.952022 -2.952765 1.376970
F -2.224917 -1.087411 2.187471
C 2.912373 -0.260371 -2.568914
C 4.169471 0.325302 -2.720455
C 4.866738 0.795911 -1.611974
C 4.315747 0.678414 -0.340146
C 3.060466 0.096008 -0.168778
H 2.357698 -0.627210 -3.430490
H 4.600475 0.411664 -3.717066
H 5.848017 1.249722 -1.739493
H 4.862488 1.033470 0.532096
H 2.628753 0.000891 0.827631
C -1.286209 -1.407531 -3.519587
O -0.110331 -1.020994 -3.330646
C -1.628701 -2.855492 -3.605899
N -2.221079 -0.443408 -3.602946
C -1.685358 0.897792 -3.395178
C -0.530676 0.789489 -2.489800
C 0.080681 0.840295 -1.381528
C 0.354626 1.861532 -0.377063

C -0.096987 1.728204 0.938976
C 0.250103 2.686221 1.883145
C 1.055446 3.766173 1.531646
C 1.514021 3.891019 0.223793
C 1.166817 2.943607 -0.730891
H -3.180761 -0.662505 -3.345691
H -1.412477 1.343317 -4.361316
H -2.442541 1.520188 -2.901355
H -0.737525 0.890842 1.207967
H -0.112223 2.584231 2.904368
H 1.328806 4.508680 2.279190
H 2.149576 4.729020 -0.056915
H 1.547958 3.012013 -1.749618
H -1.509186 -3.230638 -2.575700
C -3.004403 -3.189213 -4.153611
H -0.821849 -3.324849 -4.184675
H -3.105736 -4.273683 -4.265919
H -3.792118 -2.861427 -3.464944
H -3.174261 -2.735282 -5.138389

TS_{ar}^{B}

48
Energy: -3887.68126241
Cu 0.407090 -0.910259 -1.318448
Cl 1.327990 -2.573165 -0.277031
C 2.076739 0.084751 -1.383410
O -1.397289 -1.717005 -1.134987
S -2.594369 -1.087057 -0.463990
O -3.580118 -0.604050 -1.438207
C -3.350474 -2.582313 0.280967
O -2.243419 -0.201008 0.639433
F -4.477967 -2.246221 0.888036
F -3.616465 -3.461104 -0.677224
F -2.522473 -3.121181 1.154434
C 2.617420 0.236643 -2.654391
C 3.988701 0.452530 -2.775083
C 4.785321 0.539549 -1.638906
C 4.218483 0.403453 -0.372157
C 2.856467 0.172430 -0.234435
H 1.985480 0.169787 -3.537385
H 4.426795 0.561270 -3.765494
H 5.854257 0.719231 -1.737643
H 4.841326 0.461345 0.518447
H 2.407372 0.059387 0.750113
C -1.019520 -1.676615 -3.709247
O 0.199076 -1.534868 -3.556681
C -1.678372 -3.010865 -3.920826
N -1.841468 -0.590486 -3.701410
C -1.265805 0.672473 -3.320982
C -0.414391 0.683322 -2.104757
C 0.356568 1.222522 -1.264296
C 0.610601 2.257196 -0.284282
C -0.294732 2.414425 0.771572
C -0.094036 3.440942 1.687809
C 0.988958 4.302779 1.549148
C 1.889134 4.140821 0.495967
C 1.711387 3.112438 -0.415985
H -2.787646 -0.744449 -3.354030
H -0.636212 1.053408 -4.139008
H -2.084269 1.388433 -3.167369
H -1.132157 1.722667 0.864643
H -0.790378 3.564747 2.514622
H 1.138145 5.106189 2.268528
H 2.735243 4.816996 0.391017
H 2.413951 2.963257 -1.235311
H -1.821895 -3.136977 -5.005767
C -0.864713 -4.153169 -3.339926
H -2.686107 -2.978307 -3.479816
H -1.354083 -5.112600 -3.540890
H 0.141596 -4.176637 -3.771814
H -0.754329 -4.037063 -2.254997

I_2

48
Energy: -3887.73486044
Cu 0.210679 -1.514861 -1.915055
Cl 0.838321 -3.592169 -2.290001
C 2.046081 0.352665 -0.730941
O -0.164654 -1.741742 -0.089213
S -1.677671 -1.672832 0.141145

O -2.401086 -1.936574 -1.106147
C -1.918219 -3.147005 1.208757
O -2.081385 -0.517409 0.929261
F -3.202101 -3.221760 1.527220
F -1.559260 -4.236096 0.563399
F -1.199425 -3.016930 2.309368
C 2.647328 -0.541571 -1.627408
C 3.754392 -1.292081 -1.240198
C 4.282459 -1.140697 0.034731
C 3.710506 -0.226648 0.920012
C 2.601654 0.516372 0.542150
H 2.301403 -0.593299 -2.661389
H 4.193724 -1.998156 -1.941771
H 5.144194 -1.730171 0.341874
H 4.126177 -0.104361 1.918701
H 2.135063 1.207637 1.242898
C -1.160682 -0.790265 -4.118178
O -0.026304 -1.193558 -3.748989
C -1.751957 -1.421184 -5.339410
N -1.875494 0.115643 -3.458897
C -1.498549 0.757518 -2.200489
C -0.166232 0.336662 -1.709670
C 0.789672 1.040160 -1.096683
C 0.608272 2.459908 -0.714190
C -0.534241 2.861605 -0.014241
C -0.696812 4.193612 0.353325
C 0.277204 5.131799 0.026878
C 1.425225 4.734359 -0.655561
C 1.595364 3.404377 -1.016811
H -2.819553 0.282747 -3.785003
H -1.472188 1.847049 -2.365172
H -2.293163 0.542753 -1.470831
H -1.269133 2.111458 0.281692
H -1.582645 4.495532 0.909458
H 0.148771 6.173839 0.314876
H 2.193381 5.465006 -0.903319
H 2.495146 3.084764 -1.542446
H -2.618256 -0.848625 -5.697904
H -0.987290 -1.405309 -6.126191
C -2.128295 -2.863361 -4.992925
H -2.548155 -3.368953 -5.868837
H -1.245337 -3.417025 -4.651730
H -2.870857 -2.893215 -4.185182

TS_{rc}^{B}

48
Energy: -3887.71267272
Cu -0.115127 1.213667 -0.307638
Cl 1.533108 1.755234 0.913180
C 2.661160 -0.175428 -1.566232
O -1.736502 2.025672 -0.969186
S -3.027561 1.649739 -0.267592
O -2.768788 0.955442 0.994933
C -3.680701 3.300404 0.187952
O -4.010184 1.058498 -1.173456
F -4.835718 3.147310 0.815159
F -2.822860 3.911352 0.985306
F -3.864926 4.024618 -0.902879
C 2.791219 -0.879127 -0.362190
C 4.024839 -0.960595 0.269289
C 5.134250 -0.323136 -0.274918
C 5.014025 0.386141 -1.467866
C 3.791496 0.445217 -2.118538
H 1.919405 -1.357536 0.076403
H 4.113873 -1.511457 1.203475
H 6.096225 -0.375699 0.232193
H 5.877443 0.894844 -1.892469
H 3.696989 0.997895 -3.052122
C -1.479990 -1.338291 -0.174671
O -0.304336 -0.987716 0.085638
C -2.309646 -2.028893 0.862310
N -1.970915 -1.112634 -1.395247
C -1.156047 -0.412892 -2.377054
C 0.141593 0.038417 -1.763361
C 1.381416 -0.103138 -2.276277
C 1.422472 -0.124138 -3.768009
C 0.764852 0.850999 -4.523690
C 0.798222 0.803348 -5.913530
C 1.486280 -0.218499 -6.559009
C 2.149155 -1.190007 -5.811951
C 2.125276 -1.142332 -4.424283

H -2.975596 -1.131656 -1.541906
H -0.934411 -1.088988 -3.216380
H -1.737705 0.441818 -2.746169
H 0.248143 1.663714 -4.011828
H 0.291761 1.573706 -6.491975
H 1.514308 -0.254885 -7.646538
H 2.693118 -1.988214 -6.313951
H 2.650203 -1.893726 -3.836034
H -2.236740 -1.397406 1.756457
C -3.759123 -2.284192 0.492030
H -1.781285 -2.963590 1.101732
H -4.274129 -2.778437 1.322282
H -4.288756 -1.342916 0.294236
H -3.853491 -2.940520 -0.383594

Reaction 23

reactant

24
Energy: -852.953584546
C 0.003873 -0.003867 0.001199
O -0.014590 -0.017951 1.214669
C -1.319110 0.013978 -0.780088
N 1.115139 -0.002449 -0.761195
H 1.058234 0.009718 -1.770654
C 2.425180 -0.018831 -0.120428
C 3.486272 -0.012999 -1.111506
H 2.504242 0.850497 0.550022
H 2.493324 -0.905717 0.527866
C 4.361598 -0.007990 -1.948342
C 5.396707 -0.002149 -2.930912
C 5.913467 1.210434 -3.408383
C 6.919646 1.212318 -4.364465
C 7.421133 0.009334 -4.854643
C 6.913359 -1.199370 -4.385475
C 5.907107 -1.208895 -3.429504
H 5.515207 2.145364 -3.018116
H 7.315781 2.158130 -4.729746
H 8.210412 0.013821 -5.604382
H 7.304600 -2.140739 -4.767140
H 5.503893 -2.148399 -3.055651
F -1.128251 0.030071 -2.109108
F -2.033861 -1.063380 -0.481174
F -2.021410 1.091328 -0.453236

I_c

59
Energy: -4453.58103677
Cu -1.010173 0.260114 0.626519
Cl -1.777564 -0.210763 2.599409
C -1.913668 1.913559 0.915036
O -0.156647 -1.556599 0.427235
S 0.562278 -2.194731 -0.731702
O 1.627268 -1.361213 -1.297376
C 1.454118 -3.537013 0.147783
O -0.320471 -2.878701 -1.669144
F 2.271193 -4.150667 -0.692114
F 2.179105 -3.032756 1.150052
F 0.595782 -4.408799 0.645974
C -1.084847 3.018361 0.871134
C -1.692867 4.277164 0.820824
C -3.075852 4.399995 0.845906
C -3.873651 3.261736 0.926934
C -3.292417 1.995696 0.974124
C 1.958156 2.441748 -1.358329
O 1.533009 3.539426 -1.050748
C 3.468806 2.181720 -1.264049
N 1.249649 1.422178 -1.887059
C -0.161151 1.587894 -2.117146
C -1.062615 0.661927 -1.403807
C -2.061574 -0.064143 -1.326667
C -3.279725 -0.778248 -1.193673
C -3.304489 -2.156769 -0.922086
C -4.526887 -2.798855 -0.796970
C -5.716110 -2.089156 -0.950533
C -5.695439 -0.723926 -1.230773
C -4.483294 -0.062756 -1.345692
H -0.001533 2.938874 0.838622
H -1.055048 5.156959 0.750733
H -3.535949 5.385949 0.814409

H -4.958303 3.349227 0.974494
H -3.909214 1.104519 1.075119
H 1.637893 0.476640 -1.889159
H -0.413636 2.618073 -1.823957
H -0.383386 1.483530 -3.189766
H -2.364610 -2.696572 -0.828803
H -4.552574 -3.864798 -0.581778
H -6.669760 -2.605241 -0.853311
H -6.626903 -0.175844 -1.356285
H -4.444008 1.004415 -1.558841
F 3.801391 0.926511 -1.577153
F 4.091063 2.992808 -2.119770
F 3.919029 2.450860 -0.043845
O 2.994267 0.062409 1.037773
C 4.072758 -0.685558 1.619801
C 4.951669 -1.138460 0.484284
C 1.860341 0.295909 1.670928
O 1.011631 0.948029 1.073608
C 1.663010 -0.246098 3.049708
H 4.609312 -0.030938 2.321819
H 3.673687 -1.541840 2.175629
H 5.762389 -1.770995 0.863460
H 4.358151 -1.711755 -0.238677
H 5.387406 -0.280261 -0.037646
H 2.542597 -0.088657 3.683881
H 0.781956 0.218664 3.496573
H 1.474998 -1.326611 2.981617

I_1

45
Energy: -4146.05348170
Cu -0.008861 0.048421 -0.001201
Cl 0.033317 -0.019246 2.123142
C 1.851067 0.044884 0.327653
O -2.035637 0.328291 -0.257704
S -1.984278 1.745471 -0.778155
O -2.659284 1.973945 -2.041103
C -2.917299 2.678775 0.495121
O -0.581599 2.209613 -0.655299
F -2.901025 3.965162 0.187105
F -4.166617 2.246032 0.524979
F -2.358953 2.500407 1.677798
C 2.595776 -1.115783 0.398437
C 3.977997 -0.985584 0.258945
C 4.559118 0.270026 0.095649
C 3.768542 1.413889 0.075262
C 2.383044 1.316107 0.217815
H 2.133317 -2.090862 0.549110
H 4.597771 -1.880236 0.292407
H 5.640168 0.357400 0.002628
H 4.221026 2.397790 -0.037424
H 1.752047 2.203384 0.232821
C -0.528303 -3.253698 0.527855
O 0.493235 -3.682287 0.026526
C -0.853911 -3.581555 1.992137
N -1.462553 -2.531244 -0.130782
C -1.220301 -2.179481 -1.515599
C -0.200032 -1.129448 -1.706019
C 0.558575 -0.353264 -2.284421
C 1.513943 0.502723 -2.890868
C 1.169974 1.817593 -3.243286
C 2.132365 2.644674 -3.802503
C 3.424905 2.169673 -4.017019
C 3.765349 0.861257 -3.678745
C 2.814668 0.024759 -3.115324
H -2.186117 -2.034954 0.378916
H -0.890920 -3.079514 -2.049847
H -2.164965 -1.835134 -1.949558
H 0.159833 2.173387 -3.051297
H 1.874190 3.666656 -4.071508
H 4.175003 2.826395 -4.454733
H 4.776472 0.496850 -3.848799
H 3.063067 -0.994571 -2.824498
F -1.191193 -4.866944 2.080195
F 0.210658 -3.372424 2.748594
F -1.870264 -2.852846 2.461380

TS_{rc}^{A}

45
Energy: -4146.01926592

Cu 0.800757 -0.932973 -0.717660
Cl 1.611977 -2.872699 -0.387606
C 2.505717 -0.399712 -1.282581
O -0.843937 -1.150844 0.355030
S -2.251045 -1.260567 -0.165933
O -2.400896 -2.251528 -1.234716
C -3.103691 -1.968196 1.294046
O -2.894692 0.035499 -0.403900
F -4.385361 -2.130081 1.000840
F -2.574471 -3.132669 1.613410
F -2.991602 -1.132673 2.315123
C 2.888124 -0.389348 -2.611478
C 4.142335 0.130638 -2.930331
C 4.984527 0.607285 -1.929308
C 4.581476 0.561287 -0.598933
C 3.329266 0.047319 -0.262039
H 2.226018 -0.766718 -3.388213
H 4.458492 0.159110 -3.972192
H 5.964232 1.005395 -2.187504
H 5.243036 0.915868 0.190019
H 3.018021 -0.000673 0.781399
C -1.351946 -1.360887 -3.424716
O -0.197988 -1.093678 -3.063565
C -1.720044 -2.752545 -3.933509
N -2.239215 -0.373846 -3.547134
C -1.712061 0.927193 -3.156496
C -0.545106 0.722663 -2.277415
C 0.158591 0.838130 -1.231624
C 0.347546 1.937829 -0.279053
C -0.589676 2.179374 0.729578
C -0.378423 3.229132 1.616244
C 0.748634 4.037683 1.500600
C 1.676167 3.795015 0.491682
C 1.485084 2.742923 -0.395480
H -3.233757 -0.572111 -3.531366
H -1.454111 1.506318 -4.054141
H -2.466821 1.465121 -2.567691
H -1.472902 1.546632 0.804653
H -1.106733 3.416885 2.403132
H 0.904382 4.858722 2.198178
H 2.558249 4.425490 0.394550
H 2.208652 2.539978 -1.184534
F -1.105983 -3.693662 -3.262818
F -3.035612 -2.934657 -3.900055
F -1.328010 -2.800634 -5.211011

TS_{ar}^{B}

45
Energy: -4146.03652259
Cu 0.378824 -0.932257 -1.059374
Cl 1.213761 -2.468840 0.205239
C 2.063640 0.027979 -1.136898
O -1.391724 -1.769146 -1.011435
S -2.545693 -1.059276 -0.327042
O -3.508222 -0.532198 -1.299176
C -3.366037 -2.490152 0.474520
O -2.105990 -0.172248 0.744140
F -4.438551 -2.059196 1.120313
F -3.729686 -3.362433 -0.450529
F -2.535558 -3.059450 1.324284
C 2.676985 0.046120 -2.384349
C 4.056759 0.233829 -2.446095
C 4.788035 0.428901 -1.279899
C 4.148873 0.429355 -0.039927
C 2.779274 0.223774 0.041246
H 2.094178 -0.096477 -3.292153
H 4.552139 0.237677 -3.415136
H 5.863376 0.587768 -1.334636
H 4.721808 0.573377 0.873996
H 2.273109 0.215693 1.004376
C -0.960227 -1.839057 -3.522376
O 0.255998 -1.748412 -3.426097
C -1.638043 -3.210593 -3.628748
N -1.793322 -0.779704 -3.575889
C -1.215296 0.506213 -3.272288
C -0.375265 0.580347 -2.049000
C 0.368163 1.208154 -1.245800
C 0.599848 2.331916 -0.366417
C -0.343504 2.596127 0.633692
C -0.168726 3.705893 1.452697
C 0.927475 4.543092 1.272555

C 1.867064 4.273017 0.277108
C 1.714965 3.161664 -0.536435
H -2.742413 -0.925852 -3.228849
H -0.577692 0.823879 -4.109948
H -2.033838 1.230476 -3.174750
H -1.187237 1.918461 0.763442
H -0.894155 3.913818 2.236400
H 1.057075 5.411996 1.915466
H 2.723617 4.930214 0.141475
H 2.448378 2.926029 -1.307040
F -2.897955 -3.159263 -3.190114
F -1.660374 -3.581510 -4.910667
F -0.973318 -4.109843 -2.934912

I_2

45
Energy: -4146.08641837
Cu -0.810873 0.039600 0.753187
Cl -2.274496 0.869878 2.152647
C 1.963101 0.041803 1.511339
O -0.213932 1.692380 0.104559
S -0.665266 1.867755 -1.351713
O -1.753860 0.935834 -1.658979
C -1.414274 3.542508 -1.268424
O 0.450291 1.948285 -2.282469
F -1.849093 3.860008 -2.478088
F -2.419747 3.541486 -0.420917
F -0.499737 4.415050 -0.885447
C 1.083005 -0.255561 2.561864
C 1.137567 0.462345 3.753473
C 2.083305 1.465598 3.913415
C 2.985173 1.743037 2.885330
C 2.928887 1.037238 1.692827
H 0.409257 -1.111180 2.483294
H 0.431789 0.233474 4.549071
H 2.125421 2.032044 4.841633
H 3.728970 2.527581 3.012466
H 3.610616 1.274582 0.877154
C -1.768519 -2.346008 -0.214404
O -1.624638 -1.671026 0.821657
C -2.893707 -3.387539 -0.166341
N -1.053874 -2.294048 -1.321306
C 0.118708 -1.434858 -1.530482
C 0.565679 -0.786517 -0.277761
C 1.803100 -0.622998 0.203290
C 3.010267 -1.024194 -0.554751
C 3.176601 -0.612730 -1.881862
C 4.318543 -0.978487 -2.587358
C 5.298370 -1.754439 -1.976175
C 5.145145 -2.152816 -0.649681
C 4.011712 -1.782389 0.061671
H -1.363420 -2.868547 -2.097390
H 0.930680 -2.081474 -1.898167
H -0.134298 -0.712856 -2.319560
H 2.427999 0.037930 -2.337550
H 4.448189 -0.643134 -3.614814
H 6.190893 -2.040386 -2.530080
H 5.915649 -2.751539 -0.167050
H 3.889331 -2.082261 1.102423
F -2.986397 -4.043078 -1.329602
F -2.631785 -4.263224 0.790483
F -4.042968 -2.794139 0.080889

TS_{rc}^{B}

59
Energy: -4118.82965342
Cu -1.867837 0.445921 -0.042614
Cl -3.596971 0.259589 -1.396652
C 0.518158 -0.604902 2.203456
O -1.113221 2.019625 0.716308
S -0.066477 2.714733 -0.142327
O 0.189401 1.988121 -1.387995
C -0.981175 4.219934 -0.650579
O 1.075291 3.170715 0.635505
F -0.211091 4.952366 -1.440805
F -2.076114 3.872284 -1.307293
F -1.314420 4.928226 0.415539
C -0.712257 -0.268558 2.787479
C -0.859612 -0.230314 4.163213
C 0.220315 -0.526352 4.991603

C 1.448427 -0.858289 4.429250
C 1.598353 -0.895478 3.049851
H -1.554478 -0.010869 2.152410
H -1.820997 0.045961 4.591299
H 0.105118 -0.489526 6.073317
H 2.299312 -1.086980 5.068207
H 2.563174 -1.158848 2.620393
C -2.051389 -2.352692 -0.711345
O -1.893309 -1.673854 0.340325
C -3.079860 -3.458880 -0.759706
N -1.214704 -2.140171 -1.725460
C -0.310981 -1.000846 -1.616407
C -0.270381 -0.618555 -0.174545
C 0.694856 -0.690959 0.751601
C 2.081445 -0.785944 0.202625
C 2.886338 0.351761 0.133566
C 4.180875 0.245431 -0.361806
C 4.683055 -0.985636 -0.790712
C 3.868103 -2.119953 -0.717970
C 2.575775 -2.022684 -0.228175
H -1.425899 -2.520042 -2.639683
H 0.700138 -1.286757 -1.935626
H -0.654392 -0.176751 -2.258302
H 2.490340 1.316768 0.454447
H 4.795685 1.142179 -0.417788
C 6.065250 -1.154136 -1.334646
H 4.280598 -3.070286 -1.051399
H 1.938073 -2.906019 -0.162907
C -4.287158 -3.050645 0.083203
C -3.518969 -3.743414 -2.194429
C -2.404152 -4.696329 -0.151332
H -4.321906 -4.490825 -2.181107
H -3.911150 -2.838744 -2.678976
H -2.710671 -4.167738 -2.808753
H -3.121428 -5.527412 -0.137284
H -1.529190 -5.010031 -0.737347
H -2.082884 -4.503677 0.880229
H -5.030266 -3.858124 0.058352
H -4.001897 -2.874805 1.126534
H -4.744545 -2.131684 -0.304981
O 6.456065 -2.248093 -1.696572
C 6.953052 0.059714 -1.422513
H 6.507331 0.830084 -2.065834
H 7.104505 0.512779 -0.433723
H 7.920147 -0.239545 -1.834774

Reaction 24

reactant

31
Energy: -746.894725842
C 0.000637 -0.172489 -0.077863
O -0.007809 -0.226773 1.143627
C 1.267452 -0.080202 -0.864548
N -1.160409 -0.194318 -0.797454
H -1.170293 0.092956 -1.766583
C -2.429061 -0.200879 -0.085083
C -3.546643 -0.282232 -1.011404
H -2.428895 -1.053507 0.609042
H -2.515199 0.696458 0.549724
C -4.473910 -0.339354 -1.788647
C -5.570762 -0.412643 -2.698951
C -5.928180 -1.636702 -3.281859
C -6.996229 -1.705281 -4.165823
C -7.720639 -0.558016 -4.478926
C -7.372877 0.661732 -3.904282
C -6.305239 0.737562 -3.020346
H -5.357174 -2.528362 -3.028902
H -7.266644 -2.660432 -4.612758
H -8.558527 -0.614799 -5.171697
H -7.938060 1.560081 -4.146596
H -6.026296 1.686484 -2.565500
C 1.362750 -0.419248 -2.216205
C 2.578711 -0.309119 -2.881038
C 3.706071 0.141426 -2.200704
C 3.619200 0.468576 -0.849732
C 2.406936 0.351344 -0.183817
H 0.496413 -0.809061 -2.750485
H 2.647924 -0.584229 -3.932085
H 4.657336 0.229423 -2.723184

H 4.502070 0.813195 -0.314036
H 2.312949 0.586732 0.874462

I_c

66
Energy: -4347.52254648
Cu -1.216238 0.356574 0.570236
Cl -2.020692 -0.017911 2.551014
C -2.008471 2.073152 0.806368
O -0.480463 -1.513903 0.428365
S 0.224882 -2.255446 -0.673733
O 1.370944 -1.536387 -1.238689
C 0.976832 -3.618614 0.302168
O -0.675220 -2.914500 -1.613101
F 1.813855 -4.303928 -0.462032
F 1.660653 -3.126421 1.338072
F 0.039745 -4.427646 0.763034
C -1.100884 3.110247 0.714228
C -1.614220 4.408413 0.631632
C -2.983617 4.635390 0.672892
C -3.862400 3.562817 0.801251
C -3.376340 2.258512 0.880297
C 1.940679 2.210773 -1.389189
O 1.530494 3.326802 -1.090770
C 3.371401 1.833554 -1.206668
N 1.149798 1.250640 -1.959007
C -0.236916 1.534477 -2.203427
C -1.221203 0.707651 -1.474886
C -2.265880 0.051690 -1.376484
C -3.524249 -0.588616 -1.231419
C -3.622256 -1.963724 -0.959258
C -4.876540 -2.539569 -0.826631
C -6.026598 -1.766624 -0.971314
C -5.933518 -0.404277 -1.251261
C -4.688087 0.190457 -1.375773
H -0.027251 2.951917 0.657764
H -0.913332 5.234495 0.520713
H -3.370124 5.651390 0.615241
H -4.936703 3.732611 0.860757
H -4.054960 1.418787 1.018287
H 1.417607 0.270077 -1.854364
H -0.397839 2.589570 -1.934277
H -0.473777 1.423564 -3.273202
H -2.712296 -2.554193 -0.873128
H -4.958026 -3.602993 -0.612463
H -7.006237 -2.230240 -0.867895
H -6.834847 0.193556 -1.370064
H -4.592454 1.253745 -1.590729
O 2.743464 -0.171444 1.130728
C 3.714853 -0.995796 1.791284
C 4.474763 -1.730022 0.719425
C 1.631563 0.234001 1.718918
O 0.854004 0.906507 1.054034
C 1.372178 -0.140664 3.144549
H 4.371834 -0.341733 2.383893
H 3.212791 -1.696922 2.467893
H 5.216917 -2.396820 1.174411
H 3.786810 -2.326907 0.109062
H 4.991256 -1.021864 0.062024
H 2.259584 -0.008889 3.773959
H 0.541773 0.458137 3.524012
H 1.068885 -1.195709 3.191596
C 4.012890 0.902455 -2.024058
C 5.373088 0.656788 -1.864817
C 6.089069 1.314612 -0.868842
C 5.445152 2.229245 -0.036385
C 4.096115 2.500742 -0.218243
H 3.458745 0.390413 -2.809353
H 5.874766 -0.053093 -2.520624
H 7.153025 1.118288 -0.743012
H 6.004822 2.746645 0.741477
H 3.576496 3.235592 0.394390

I_1

52
Energy: -4039.99709184
Cu 0.022253 -0.000357 -0.005806
Cl 0.073432 -0.058912 2.121313
C 1.888649 -0.010713 0.289400
O -1.999269 0.319139 -0.249261

S -1.940178 1.743643 -0.747686
O -2.620681 1.995625 -2.003590
C -2.868764 2.661129 0.540155
O -0.537431 2.201554 -0.621577
F -2.853887 3.951790 0.248268
F -4.119278 2.229333 0.568802
F -2.309368 2.469795 1.720471
C 2.624894 -1.178410 0.293960
C 4.008867 -1.050091 0.167615
C 4.599470 0.208174 0.075084
C 3.815964 1.356622 0.111519
C 2.429435 1.260682 0.245099
H 2.147064 -2.155685 0.368348
H 4.622759 -1.949525 0.148816
H 5.681385 0.293220 -0.010741
H 4.275543 2.342014 0.053478
H 1.804123 2.149913 0.305092
C -0.593652 -3.207554 0.607052
O 0.464400 -3.591112 0.119704
C -0.951892 -3.433150 2.031029
N -1.515711 -2.537449 -0.157965
C -1.187178 -2.231331 -1.530567
C -0.180811 -1.161909 -1.714318
C 0.563905 -0.361240 -2.278552
C 1.511925 0.502687 -2.889102
C 1.164867 1.819432 -3.229689
C 2.119543 2.648987 -3.798798
C 3.408467 2.174974 -4.035205
C 3.752289 0.864744 -3.708489
C 2.809005 0.026182 -3.135492
H -2.198189 -1.936233 0.291596
H -0.799206 -3.139915 -2.006902
H -2.106283 -1.928924 -2.045734
H 0.158104 2.175524 -3.022024
H 1.857613 3.672486 -4.058490
H 4.152546 2.833512 -4.480506
H 4.760264 0.499840 -3.895741
H 3.060484 -0.995717 -2.856797
C 0.091730 -3.684767 2.923230
C -0.174012 -3.878779 4.270886
C -1.486924 -3.836237 4.734062
C -2.532951 -3.602652 3.846720
C -2.267301 -3.398864 2.497990
H 1.109340 -3.710097 2.537653
H 0.644214 -4.060736 4.965186
H -1.695941 -3.988960 5.791617
H -3.560345 -3.581741 4.205300
H -3.094309 -3.243348 1.806074

TS_{rc}^{A}

52
Energy: -4039.97432905
Cu -0.595631 -0.577914 -0.629944
Cl -0.058730 -1.407357 -2.532806
C -2.122641 -1.675624 -0.675535
O 0.911833 0.708951 -0.622357
S 1.424142 1.876565 0.180039
O 2.733470 1.616603 0.783820
C 1.724594 3.094943 -1.155165
O 0.422325 2.496735 1.046390
F 2.189680 4.216728 -0.627872
F 2.596915 2.624773 -2.027254
F 0.577550 3.350798 -1.775103
C -2.124200 -2.887130 -0.008482
C -3.306888 -3.627423 0.004305
C -4.442051 -3.154944 -0.646847
C -4.402485 -1.939954 -1.323204
C -3.229853 -1.186270 -1.346795
H -1.234825 -3.246812 0.505589
H -3.333283 -4.579885 0.532096
H -5.360109 -3.739837 -0.632711
H -5.284124 -1.571062 -1.845154
H -3.197650 -0.234654 -1.877614
C 1.744946 -1.291381 1.492734
O 0.641870 -1.761252 1.128456
C 3.008390 -1.622366 0.839286
N 1.719383 -0.439621 2.552026
C 0.369794 -0.240467 3.065231
C -0.580607 -0.325298 1.946681
C -1.353885 0.090316 1.034512
C -2.541259 0.936007 0.961878

C -2.552943 2.120775 0.219763
C -3.731900 2.847872 0.111218
C -4.899554 2.398916 0.722370
C -4.887967 1.213959 1.451884
C -3.714255 0.481284 1.572697
H 2.345476 0.365950 2.489182
H 0.155872 -0.990197 3.838929
H 0.289730 0.763497 3.501386
H -1.633279 2.479205 -0.239121
H -3.735523 3.775002 -0.458878
H -5.820074 2.972016 0.626633
H -5.798456 0.853197 1.927108
H -3.701429 -0.467864 2.108249
C 4.231067 -1.426701 1.491038
C 5.414261 -1.744652 0.842983
C 5.379537 -2.250765 -0.455024
C 4.163550 -2.444810 -1.104729
C 2.973861 -2.137501 -0.460516
H 4.251777 -1.046090 2.510989
H 6.366462 -1.598401 1.348524
H 6.310482 -2.494688 -0.964219
H 4.140089 -2.830010 -2.121721
H 2.018264 -2.264174 -0.968104

TS_{ar}^{B}

52
Energy: -4039.98504063
Cu 0.559065 -0.677404 0.333531
Cl 0.236462 -1.402752 2.354090
C 2.355746 -1.414594 0.263687
O -1.076386 0.468256 0.390249
S -1.305164 1.912127 0.030548
O -1.710724 2.089874 -1.371886
C -2.835269 2.216866 0.995216
O -0.283551 2.817762 0.542184
F -3.261628 3.449092 0.759700
F -3.767728 1.352552 0.619825
F -2.596541 2.074972 2.285604
C 2.503898 -2.514101 -0.572718
C 3.536245 -3.413722 -0.316591
C 4.410720 -3.190662 0.741269
C 4.253966 -2.071272 1.557650
C 3.220368 -1.173454 1.326418
H 1.817447 -2.680365 -1.400331
H 3.656208 -4.285633 -0.956864
H 5.222869 -3.889928 0.931040
H 4.930351 -1.900441 2.392993
H 3.085976 -0.301181 1.962977
C -1.603300 -1.434402 -1.369595
O -0.679728 -2.112855 -0.892768
C -2.997946 -1.545375 -0.906657
N -1.342729 -0.500933 -2.331139
C 0.042751 -0.242560 -2.620836
C 0.931264 0.013047 -1.460760
C 2.027215 0.259515 -0.885295
C 3.151125 1.148427 -0.675651
C 2.893766 2.469333 -0.290983
C 3.960031 3.348845 -0.138941
C 5.262191 2.920034 -0.374291
C 5.512966 1.601768 -0.754988
C 4.462133 0.708841 -0.894869
H -1.911148 0.347790 -2.282961
H 0.473125 -1.098240 -3.162163
H 0.096888 0.633010 -3.281743
H 1.866598 2.785495 -0.102991
H 3.767488 4.375328 0.166000
H 6.091653 3.615296 -0.256705
H 6.533093 1.269881 -0.937597
H 4.642244 -0.328202 -1.176640
C -4.077817 -1.145021 -1.697576
C -5.366908 -1.198600 -1.183928
C -5.576057 -1.641880 0.119277
C -4.500036 -2.044890 0.906878
C -3.210630 -2.003775 0.396342
H -3.911081 -0.811367 -2.721159
H -6.210118 -0.893094 -1.800383
H -6.586402 -1.673488 0.524104
H -4.666262 -2.382340 1.927901
H -2.352268 -2.286572 1.005061

I_2

52
Energy: -4040.03976362
Cu -0.444076 0.510088 0.686704
Cl -1.453848 2.023113 1.931322
C 1.961405 -0.890747 1.551871
O 0.940735 1.620419 0.112578
S 0.810348 1.933690 -1.383124
O -0.565892 1.720122 -1.835992
C 1.073658 3.750579 -1.353539
O 1.889356 1.367261 -2.177976
F 1.023426 4.197160 -2.600028
F 0.132620 4.330392 -0.638877
F 2.260590 4.018945 -0.839096
C 1.059049 -0.646427 2.595614
C 1.485078 -0.026589 3.766429
C 2.817966 0.335677 3.913912
C 3.728343 0.063724 2.893529
C 3.306076 -0.546932 1.721016
H 0.031623 -1.007049 2.525111
H 0.766708 0.172793 4.559027
H 3.152607 0.825917 4.826176
H 4.774082 0.343537 3.008817
H 4.009960 -0.731363 0.910305
C -2.474834 -1.051475 -0.196133
O -1.903812 -0.634994 0.848742
C -3.936669 -1.221835 -0.155312
N -1.821959 -1.339901 -1.322458
C -0.370043 -1.298093 -1.503740
C 0.355279 -0.927539 -0.266000
C 1.480225 -1.421423 0.258461
C 2.295549 -2.439548 -0.445581
C 2.660354 -2.251875 -1.782941
C 3.429815 -3.208111 -2.438325
C 3.841677 -4.354117 -1.765779
C 3.496799 -4.537435 -0.428150
C 2.735783 -3.581924 0.232135
H -2.380729 -1.472225 -2.156509
H -0.035608 -2.302770 -1.810213
H -0.160166 -0.598544 -2.325379
H 2.377477 -1.326734 -2.287284
H 3.720084 -3.048288 -3.475334
H 4.443833 -5.101440 -2.279864
H 3.826841 -5.428372 0.103523
H 2.470114 -3.714733 1.280844
C -4.599285 -2.165928 -0.945565
C -5.981642 -2.269066 -0.880413
C -6.701008 -1.428310 -0.033890
C -6.040613 -0.491750 0.757015
C -4.657042 -0.388948 0.706561
H -4.035197 -2.849630 -1.580120
H -6.499391 -3.011847 -1.483699
H -7.785565 -1.508880 0.012813
H -6.606159 0.163048 1.416390
H -4.117090 0.338999 1.311835

TS_{rc}^{B}

52
Energy: -4040.01084189
Cu 0.144387 -1.273640 0.307749
Cl 1.485103 -2.895549 -0.276329
C -0.504530 1.843506 1.570408
O -1.669495 -1.296051 0.894062
S -2.696646 -1.174929 -0.221592
O -2.062139 -0.972866 -1.526470
C -3.349554 -2.886691 -0.275904
O -3.816882 -0.321975 0.142999
F -4.276120 -2.972341 -1.218460
F -2.365586 -3.728152 -0.553279
F -3.882591 -3.206752 0.891708
C -0.182502 0.879082 2.537162
C -0.272726 1.167206 3.887849
C -0.690168 2.427892 4.308056
C -1.018218 3.394669 3.363280
C -0.929131 3.106784 2.008420
H 0.117052 -0.116262 2.221452
H -0.029111 0.397420 4.617206
H -0.767591 2.651517 5.370546
H -1.349947 4.381133 3.682017
H -1.184988 3.870485 1.276387
C 2.675824 0.040879 -0.519388

O 1.909206 0.046880 0.480017
C 4.119235 -0.153359 -0.362675
N 2.145926 0.248491 -1.732337
C 0.689804 0.246375 -1.807861
C 0.189883 0.504664 -0.425939
C -0.380459 1.581720 0.134780
C -1.007107 2.538097 -0.826563
C -2.390635 2.540149 -1.015831
C -2.961106 3.455861 -1.893686
C -2.162256 4.366159 -2.580030
C -0.782410 4.362749 -2.393197
C -0.203883 3.447533 -1.520475
H 2.681934 -0.020632 -2.548751
H 0.334868 1.051230 -2.464984
H 0.328466 -0.709788 -2.212449
H -3.010081 1.813194 -0.487497
H -4.038576 3.450584 -2.046643
H -2.615634 5.079732 -3.265991
H -0.155340 5.077108 -2.924172
H 0.875839 3.438470 -1.360191
C 5.032953 0.444510 -1.235965
C 6.392078 0.223831 -1.063368
C 6.835689 -0.598159 -0.029837
C 5.924464 -1.194058 0.838298
C 4.564143 -0.968998 0.680858
H 4.684170 1.111227 -2.024441
H 7.107937 0.697165 -1.732057
H 7.901970 -0.774172 0.100062
H 6.275535 -1.841365 1.638905
H 3.831188 -1.442822 1.330383

Reaction 25

reactant

35
Energy: -861.351288851
C 0.034304 2.572671 -1.087558
C 0.835824 1.345333 -1.350993
N -1.287548 2.430159 -0.727543
H -1.745780 3.328419 -0.622143
C -1.934509 1.284749 -0.111723
C -2.924197 0.634922 -0.969315
H -1.159800 0.563576 0.183703
H -2.413783 1.607668 0.824881
C -3.735226 0.102365 -1.695284
C -4.692028 -0.523857 -2.548682
C -4.381814 -0.784637 -3.890834
C -5.315444 -1.392516 -4.718907
C -6.566025 -1.749700 -4.221378
C -6.881463 -1.495407 -2.889292
C -5.953653 -0.885939 -2.055929
H -3.401661 -0.501614 -4.271370
H -5.065542 -1.589408 -5.760043
H -7.295978 -2.226867 -4.873029
H -7.858340 -1.773201 -2.497177
H -6.192537 -0.682274 -1.013525
C 0.314788 0.168777 -1.906894
C 1.142767 -0.902043 -2.191344
C 2.513300 -0.823480 -1.922166
C 3.048938 0.349197 -1.383558
C 2.208463 1.422369 -1.118842
H -0.746062 0.096072 -2.148517
H 0.756276 -1.818062 -2.633353
O 3.234982 -1.926249 -2.224507
H 4.112787 0.438278 -1.178653
H 2.612711 2.354167 -0.727549
C 4.621726 -1.892967 -1.991484
H 5.009118 -2.866928 -2.301032
H 5.112448 -1.105244 -2.582478
H 4.849575 -1.736485 -0.926395
O 0.518267 3.688523 -1.202873

I_c

70
Energy: -4461.98507866
Cu -1.490781 0.407031 0.543941
Cl -2.190124 0.117329 2.578283
C -2.118680 2.178991 0.830575
O -0.978520 -1.520276 0.365274
S -0.323433 -2.320861 -0.726829

O 0.832560 -1.664011 -1.342583
C 0.395822 -3.671810 0.288078
O -1.270499 -2.979255 -1.619282
F 1.190410 -4.423225 -0.459691
F 1.117389 -3.162032 1.289585
F -0.562289 -4.422824 0.801514
C -1.133625 3.138508 0.709161
C -1.541850 4.474364 0.636251
C -2.885926 4.811844 0.721775
C -3.844739 3.814620 0.885078
C -3.463730 2.475849 0.951899
C 1.599629 2.099985 -1.540386
O 1.257218 3.252885 -1.295859
C 2.983560 1.623322 -1.295086
N 0.749013 1.172159 -2.081808
C -0.627535 1.520038 -2.295507
C -1.619382 0.765866 -1.499499
C -2.697018 0.177973 -1.342774
C -3.975979 -0.393094 -1.118672
C -4.130830 -1.767929 -0.871128
C -5.403123 -2.278509 -0.663781
C -6.515812 -1.441296 -0.710990
C -6.367373 -0.079368 -0.968287
C -5.102444 0.450477 -1.166642
H -0.079545 2.892124 0.617282
H -0.779796 5.239202 0.493700
H -3.190321 5.855760 0.671064
H -4.898941 4.072055 0.979610
H -4.202749 1.693185 1.114574
H 0.958887 0.183496 -1.938932
H -0.725233 2.590752 -2.059663
H -0.907800 1.383238 -3.351518
H -3.251375 -2.409368 -0.860653
H -5.528654 -3.341170 -0.467886
H -7.510227 -1.854205 -0.549646
H -7.240590 0.568273 -1.012480
H -4.962899 1.511365 -1.369133
O 2.461909 -0.516858 1.001745
C 3.373107 -1.352053 1.732530
C 4.101802 -2.221918 0.741363
C 1.417730 0.056329 1.579801
O 0.677485 0.735892 0.880794
C 1.178953 -0.156231 3.042404
H 4.065068 -0.703455 2.291731
H 2.817479 -1.969082 2.448656
H 4.709206 -2.962367 1.275540
H 3.381407 -2.749961 0.106177
H 4.760153 -1.630974 0.095852
H 2.100681 -0.095789 3.631320
H 0.452542 0.579031 3.394134
H 0.737262 -1.152151 3.188540
C 3.545025 0.523690 -1.953224
C 4.848125 0.144551 -1.693294
C 5.605207 0.834166 -0.740150
C 5.056927 1.934605 -0.077393
C 3.757407 2.326504 -0.376234
H 2.968984 -0.032175 -2.691296
H 5.309189 -0.698079 -2.205636
O 6.850601 0.347302 -0.520762
H 5.632269 2.493258 0.656889
H 3.314756 3.193819 0.110653
C 7.647084 0.988077 0.443931
H 8.594769 0.444284 0.473137
H 7.842079 2.037761 0.177915
H 7.180393 0.953361 1.440801

I_1

56
Energy: -4154.46020398
Cu 0.010975 0.016563 -0.041615
Cl 0.036309 -0.014789 2.086563
C 1.873968 -0.000201 0.276445
O -2.005702 0.349386 -0.309939
S -1.934157 1.769915 -0.817533
O -2.597923 2.015379 -2.083795
C -2.877451 2.699278 0.450849
O -0.532449 2.224963 -0.676052
F -2.857815 3.987599 0.148668
F -4.129128 2.269295 0.467851
F -2.333686 2.517606 1.640041
C 2.603827 -1.171525 0.303793

C 3.990205 -1.051098 0.196551
C 4.588550 0.203177 0.100016
C 3.810457 1.355848 0.112704
C 2.421722 1.268036 0.226685
H 2.119478 -2.145513 0.380946
H 4.599741 -1.953693 0.196103
H 5.672066 0.281917 0.029554
H 4.275876 2.338301 0.051153
H 1.800017 2.160879 0.267919
C -0.623998 -3.170939 0.580584
O 0.436458 -3.559965 0.099547
C -0.984487 -3.373583 2.000543
N -1.541948 -2.509911 -0.202014
C -1.193455 -2.214370 -1.571494
C -0.178690 -1.151163 -1.747999
C 0.577941 -0.356018 -2.304122
C 1.538159 0.500237 -2.906833
C 1.201465 1.816851 -3.258069
C 2.167013 2.639651 -3.818598
C 3.456712 2.158945 -4.036205
C 3.790256 0.848587 -3.699478
C 2.835936 0.016768 -3.134854
H -2.216378 -1.890791 0.234795
H -0.803225 -3.127296 -2.037389
H -2.103430 -1.909904 -2.101597
H 0.193630 2.178155 -3.065149
H 1.912997 3.663094 -4.086298
H 4.209471 2.812148 -4.474748
H 4.798846 0.478235 -3.872251
H 3.079201 -1.005071 -2.848812
C 0.050507 -3.663399 2.898692
C -0.208423 -3.829637 4.242725
C -1.519817 -3.719256 4.722709
C -2.564841 -3.448541 3.836769
C -2.287076 -3.275706 2.485722
H 1.065175 -3.739949 2.512394
H 0.584490 -4.040533 4.957014
O -1.672842 -3.896391 6.052335
H -3.591647 -3.373944 4.184651
H -3.117148 -3.090024 1.804832
C -2.965392 -3.770544 6.594259
H -2.863988 -3.929804 7.670618
H -3.381726 -2.768254 6.414732
H -3.651730 -4.525011 6.181532

TS_{rc}^{A}

56
Energy: -4154.43961176
Cu -0.812133 -0.626954 -0.550159
Cl 0.018685 -1.478905 -2.336095
C -2.117875 -1.980649 -0.618703
O 0.424536 0.919669 -0.557236
S 0.671280 2.199445 0.198575
O 1.946988 2.197513 0.919133
C 0.891926 3.367965 -1.195312
O -0.489503 2.696867 0.935465
F 1.136811 4.578733 -0.718645
F 1.894754 2.989249 -1.966471
F -0.224951 3.403601 -1.914075
C -1.963592 -3.123908 0.144085
C -2.994940 -4.063412 0.134797
C -4.136423 -3.850462 -0.631690
C -4.253205 -2.698105 -1.402111
C -3.232708 -1.747996 -1.405565
H -1.071355 -3.279999 0.747136
H -2.898404 -4.966281 0.736491
H -4.935786 -4.589515 -0.634069
H -5.138533 -2.531840 -2.014021
H -3.322613 -0.845615 -2.010559
C 1.428453 -0.790454 1.766237
O 0.454161 -1.466248 1.355751
C 2.772413 -0.923766 1.234910
N 1.165770 0.102989 2.763369
C -0.236340 0.088619 3.157733
C -1.067352 -0.202730 1.981336
C -1.822498 0.012568 0.990252
C -3.123641 0.627179 0.751341
C -3.266700 1.723898 -0.103583
C -4.534987 2.226463 -0.366265
C -5.661036 1.637639 0.202616
C -5.517473 0.538933 1.044479

C -4.254059 0.031723 1.319876
H 1.632119 1.007881 2.671203
H -0.388638 -0.653453 3.953209
H -0.518964 1.081153 3.531948
H -2.382461 2.192555 -0.531058
H -4.641718 3.086870 -1.024208
H -6.651662 2.033614 -0.013810
H -6.393837 0.069107 1.487544
H -4.131584 -0.850777 1.947614
C 3.892734 -0.441954 1.931533
C 5.153305 -0.568911 1.393160
C 5.321691 -1.171277 0.136749
C 4.212490 -1.655270 -0.564302
C 2.948400 -1.533842 -0.010686
H 3.770829 0.016377 2.911551
H 6.037444 -0.209973 1.914644
O 6.589241 -1.239414 -0.306608
H 4.320774 -2.113609 -1.543247
H 2.080055 -1.888492 -0.564810
C 6.825419 -1.814617 -1.573013
H 7.904081 -1.758479 -1.736365
H 6.508541 -2.867032 -1.600684
H 6.308135 -1.258607 -2.367627

TS_{ar}^{B}

56
Energy: -4154.44909218
Cu 0.794430 -0.663467 0.280542
Cl 0.244428 -1.447171 2.231363
C 2.490563 -1.610527 0.295673
O -0.678885 0.686118 0.323076
S -0.633604 2.157320 0.006253
O -1.022194 2.448182 -1.381033
C -2.044159 2.739713 1.023021
O 0.553835 2.832823 0.517804
F -2.207960 4.041001 0.833876
F -3.146990 2.104016 0.656486
F -1.806689 2.509828 2.301363
C 2.576835 -2.700032 -0.561170
C 3.493042 -3.708171 -0.266244
C 4.314597 -3.601431 0.850722
C 4.221600 -2.490037 1.687597
C 3.304519 -1.483818 1.416331
H 1.932207 -2.771719 -1.435056
H 3.564601 -4.573605 -0.922495
H 5.036011 -4.386257 1.070381
H 4.856498 -2.409517 2.567872
H 3.221462 -0.614686 2.065946
C -1.336980 -1.104742 -1.558776
O -0.504986 -1.892773 -1.075842
C -2.753527 -1.118923 -1.184633
N -0.931556 -0.147961 -2.447263
C 0.485497 -0.029758 -2.664550
C 1.348442 0.040302 -1.457714
C 2.445605 0.120586 -0.841437
C 3.688868 0.813609 -0.577695
C 3.636790 2.155168 -0.182289
C 4.825433 2.848548 0.017628
C 6.048443 2.216608 -0.181003
C 6.095014 0.878908 -0.573592
C 4.919029 0.169995 -0.761347
H -1.408622 0.752878 -2.371270
H 0.841540 -0.891407 -3.248611
H 0.665719 0.876803 -3.257524
H 2.667690 2.629059 -0.024269
H 4.791225 3.889854 0.331344
H 6.975007 2.766716 -0.025506
H 7.053539 0.387399 -0.728037
H 4.939292 -0.879360 -1.053758
C -3.753284 -0.540275 -1.978765
C -5.064495 -0.527631 -1.549099
C -5.399453 -1.082226 -0.306474
C -4.408377 -1.657868 0.494514
C -3.095314 -1.676063 0.049147
H -3.503866 -0.121141 -2.952815
H -5.860122 -0.095016 -2.151452
O -6.704053 -1.013818 0.027539
H -4.647080 -2.079656 1.467057
H -2.306551 -2.094857 0.673725
C -7.101170 -1.545586 1.270148
H -8.179642 -1.384741 1.340967

H -6.891303 -2.623465 1.332405
H -6.602868 -1.032183 2.105186

I_2

56
Energy: -4154.50471311
Cu -0.020266 -0.512405 0.671752
Cl 0.991208 -2.034790 1.910556
C -2.373598 0.942774 1.574613
O -1.434622 -1.601967 0.124765
S -1.334133 -1.922570 -1.370983
O 0.035925 -1.726664 -1.850123
C -1.613255 -3.736496 -1.328301
O -2.421183 -1.349608 -2.149993
F -1.582429 -4.191098 -2.572730
F -0.668160 -4.320804 -0.622142
F -2.795785 -3.992724 -0.797524
C -1.457352 0.688971 2.603769
C -1.874255 0.087354 3.787402
C -3.211038 -0.247215 3.962193
C -4.134284 0.034368 2.956072
C -3.721443 0.627102 1.771060
H -0.424405 1.027946 2.511162
H -1.145659 -0.119594 4.568674
H -3.538541 -0.723268 4.884543
H -5.183091 -0.223690 3.092540
H -4.436244 0.819516 0.971811
C 2.033079 0.949787 -0.274973
O 1.455974 0.610706 0.798369
C 3.494767 1.008181 -0.274394
N 1.364004 1.249980 -1.391302
C -0.091577 1.288371 -1.527873
C -0.804117 0.935474 -0.276162
C -1.905867 1.457067 0.269333
C -2.710095 2.493287 -0.421089
C -3.110972 2.309539 -1.748468
C -3.870270 3.283013 -2.390337
C -4.235722 4.442490 -1.714023
C -3.854487 4.622502 -0.385865
C -3.103719 3.649936 0.261038
H 1.900034 1.297818 -2.248816
H -0.386505 2.312055 -1.812167
H -0.359959 0.611945 -2.351580
H -2.863625 1.375165 -2.254561
H -4.188806 3.126622 -3.419566
H -4.829854 5.203315 -2.217563
H -4.148040 5.524307 0.148937
H -2.809782 3.780036 1.302532
C 4.227468 1.840425 -1.133864
C 5.605557 1.830817 -1.101445
C 6.281586 0.980355 -0.212713
C 5.558114 0.150477 0.649690
C 4.172193 0.174090 0.619853
H 3.716149 2.531295 -1.804452
H 6.197413 2.477642 -1.744875
O 7.623279 1.040876 -0.263551
H 6.063794 -0.517666 1.341215
H 3.591264 -0.473929 1.276752
C 8.365231 0.215036 0.608197
H 9.417446 0.422949 0.401859
H 8.148594 0.447495 1.660529
H 8.161825 -0.848854 0.421148

TS_{rc}^{B}

56
Energy: -4154.47576685
Cu -0.158331 -1.199961 0.263262
Cl 1.288235 -2.722188 -0.364877
C -1.081828 1.823199 1.588931
O -1.940639 -1.428907 0.908109
S -3.017194 -1.400962 -0.164543
O -2.460454 -1.125264 -1.491223
C -3.500348 -3.168387 -0.213506
O -4.202040 -0.667337 0.253008
F -4.444596 -3.340645 -1.126821
F -2.448927 -3.908566 -0.528368
F -3.962281 -3.544735 0.967949
C -0.627108 0.888059 2.530792
C -0.691272 1.155042 3.887410
C -1.214868 2.364238 4.338822

C -1.676216 3.300228 3.419040
C -1.613798 3.033266 2.058323
H -0.243121 -0.069802 2.191223
H -0.343239 0.407528 4.597423
H -1.271241 2.570759 5.406119
H -2.092489 4.245606 3.762264
H -1.975489 3.772393 1.345942
C 2.172390 0.329486 -0.651459
O 1.433703 0.261789 0.373922
C 3.620649 0.255858 -0.537763
N 1.579052 0.506011 -1.840675
C 0.126322 0.381141 -1.854360
C -0.329332 0.580788 -0.447233
C -0.989413 1.586417 0.146363
C -1.750403 2.478529 -0.779443
C -3.128304 2.318850 -0.941632
C -3.822120 3.177833 -1.787991
C -3.152659 4.190084 -2.469730
C -1.778260 4.346813 -2.310870
C -1.076529 3.489993 -1.469697
H 2.097988 0.276428 -2.679626
H -0.324724 1.158549 -2.484917
H -0.171546 -0.599016 -2.254223
H -3.647216 1.512851 -0.418715
H -4.894566 3.047463 -1.919726
H -3.702906 4.857922 -3.130449
H -1.251863 5.140223 -2.839043
H 0.000256 3.604881 -1.332139
C 4.472676 0.865675 -1.471440
C 5.839668 0.748267 -1.343108
C 6.384534 0.006523 -0.283409
C 5.541384 -0.603288 0.651522
C 4.168036 -0.470222 0.522565
H 4.061327 1.468050 -2.281225
H 6.522840 1.222675 -2.043636
O 7.725767 -0.056882 -0.252649
H 5.945330 -1.189880 1.471794
H 3.498527 -0.963199 1.224310
C 8.340260 -0.793811 0.783053
H 9.416396 -0.716675 0.613468
H 8.097376 -0.375169 1.770000
H 8.042490 -1.851355 0.751645

Reaction 26

reactant

35
Energy: -861.350540233
C 0.162344 1.951657 -0.879202
C 0.759687 0.583812 -0.964692
N -1.188742 2.064796 -0.650913
H -1.491658 3.032435 -0.659044
C -2.093949 1.094480 -0.060343
C -3.091025 0.573649 -0.993851
H -1.499053 0.268677 0.353030
H -2.599380 1.560394 0.799493
C -3.912944 0.143771 -1.773544
C -4.884228 -0.363842 -2.687430
C -4.902938 0.072965 -4.019480
C -5.849261 -0.425080 -4.904647
C -6.786065 -1.361816 -4.476057
C -6.773765 -1.800530 -3.154660
C -5.830748 -1.307071 -2.263479
H -4.166469 0.804881 -4.346541
H -5.855585 -0.079936 -5.937130
H -7.526585 -1.750586 -5.172900
H -7.504504 -2.532959 -2.815826
H -5.813949 -1.645007 -1.228738
C 0.096357 -0.508510 -1.520765
C 0.763453 -1.728492 -1.628291
C 2.066867 -1.862220 -1.181979
C 2.737309 -0.761077 -0.636053
C 2.086962 0.464390 -0.539390
H -0.920286 -0.408489 -1.899084
H 0.256765 -2.584058 -2.071437
H 2.602124 -2.807185 -1.252396
O 4.012488 -0.986608 -0.239010
H 2.579068 1.354924 -0.156979
C 4.734596 0.099282 0.288168
H 5.727338 -0.283555 0.538410
H 4.834503 0.913177 -0.445316

H 4.260756 0.496732 1.198475
O 0.845849 2.953158 -1.024824

I_c

70
Energy: -4461.98446527
Cu -1.316596 0.499235 0.524437
Cl -2.031917 0.400677 2.573204
C -1.714535 2.352807 0.703046
O -1.021111 -1.488985 0.454190
S -0.577101 -2.406757 -0.652791
O 0.666536 -1.989949 -1.307519
C -0.108017 -3.878313 0.341255
O -1.662959 -2.864021 -1.512283
F 0.501045 -4.763159 -0.433326
F 0.728218 -3.531195 1.322210
F -1.181912 -4.429313 0.878656
C -0.623743 3.167828 0.475742
C -0.862787 4.539336 0.341161
C -2.146713 5.052653 0.467315
C -3.214172 4.198246 0.731775
C -3.003343 2.826545 0.862295
C 1.916334 1.630239 -1.728416
O 1.715487 2.828716 -1.559443
C 3.254577 1.022242 -1.481084
N 0.954216 0.771847 -2.182483
C -0.374943 1.263481 -2.414949
C -1.438964 0.731918 -1.535725
C -2.586120 0.325748 -1.309684
C -3.930741 -0.017555 -1.015223
C -4.288949 -1.317949 -0.620445
C -5.617642 -1.597603 -0.339931
C -6.588100 -0.604785 -0.458715
C -6.238515 0.683072 -0.861028
C -4.913561 0.983729 -1.133521
H 0.384709 2.783808 0.350132
H -0.018498 5.190341 0.118768
H -2.318326 6.123057 0.368354
H -4.221017 4.594313 0.857836
H -3.827143 2.157288 1.103236
H 1.056918 -0.227468 -1.998169
H -0.333730 2.356245 -2.292132
H -0.692733 1.053426 -3.447807
H -3.518864 -2.083979 -0.555982
H -5.900238 -2.601073 -0.029217
H -7.628652 -0.837081 -0.238133
H -7.001338 1.452783 -0.958762
H -4.617329 1.984263 -1.445005
O 2.465183 -0.896255 0.904157
C 3.232785 -1.904492 1.580298
C 3.731638 -2.869921 0.538599
C 1.508994 -0.211912 1.508996
O 0.869923 0.591290 0.839996
C 1.247024 -0.446215 2.964116
H 4.061135 -1.406935 2.106128
H 2.606576 -2.417965 2.318734
H 4.271336 -3.693584 1.021019
H 2.890976 -3.281598 -0.031369
H 4.407419 -2.367110 -0.160036
H 2.172172 -0.519872 3.546549
H 0.617273 0.359299 3.346521
H 0.687752 -1.385297 3.079608
C 3.702780 -0.112008 -2.150690
C 4.989566 -0.584575 -1.897486
C 5.802191 0.041733 -0.965931
C 5.343705 1.177639 -0.287876
C 4.076482 1.679447 -0.561831
H 3.071245 -0.610765 -2.883315
H 5.360869 -1.457522 -2.432796
H 6.807150 -0.316679 -0.750104
O 6.209562 1.720180 0.604255
H 3.687158 2.576840 -0.087567
C 5.805411 2.886467 1.276885
H 6.634124 3.168433 1.931660
H 5.602137 3.709137 0.575087
H 4.905876 2.711914 1.887254

I_1

56
Energy: -4154.45783591

Cu 0.021612 0.010547 0.000244
Cl 0.040552 0.019674 2.128351
C 1.090282 1.533185 0.335818
O -1.389109 -1.470212 -0.286921
S -2.513489 -0.603998 -0.801622
O -3.087026 -1.010320 -2.070566
C -3.823311 -0.843359 0.459111
O -2.089059 0.807750 -0.659910
F -4.862649 -0.083610 0.152017
F -4.193604 -2.113878 0.471464
F -3.368332 -0.505711 1.651494
C 2.468315 1.468373 0.391063
C 3.159200 2.675749 0.277609
C 2.469402 3.879258 0.149370
C 1.078812 3.894595 0.137450
C 0.359546 2.703872 0.255618
H 2.994574 0.518772 0.495427
H 4.248002 2.664214 0.298858
H 3.021985 4.814303 0.074885
H 0.536402 4.834822 0.051394
H -0.728932 2.700388 0.275365
C 2.298421 -2.314070 0.672132
O 3.236428 -1.676433 0.206604
C 2.227407 -2.721486 2.100903
N 1.241909 -2.685598 -0.120865
C 1.218164 -2.245295 -1.495934
C 0.907425 -0.811624 -1.689869
C 0.681494 0.256319 -2.257161
C 0.510387 1.527117 -2.868634
C -0.766441 1.970936 -3.247564
C -0.908377 3.227094 -3.817867
C 0.208226 4.036835 -4.016386
C 1.477844 3.594860 -3.649565
C 1.633808 2.342575 -3.076112
H 0.347805 -2.907038 0.304015
H 2.202783 -2.437223 -1.939305
H 0.467843 -2.835200 -2.035015
H -1.628846 1.332396 -3.067235
H -1.896491 3.578564 -4.107186
H 0.087637 5.022833 -4.462359
H 2.346131 4.232219 -3.804913
H 2.613380 1.986406 -2.761228
C 3.028834 -2.022734 2.993623
C 2.987379 -2.316588 4.356320
C 2.145263 -3.328860 4.820217
C 1.358983 -4.037776 3.914072
C 1.389533 -3.744479 2.559920
H 3.680945 -1.228670 2.636199
O 3.796096 -1.570077 5.144909
H 2.095720 -3.574623 5.878066
H 0.714794 -4.834756 4.281137
H 0.789327 -4.330295 1.866234
C 3.767549 -1.808484 6.529345
H 4.470016 -1.101643 6.978288
H 2.765103 -1.635527 6.949612
H 4.085736 -2.834090 6.771861

TS_{rc}^{A}

56
Energy: -4154.43957405
Cu -0.078530 0.122151 -0.011261
Cl -0.258245 0.250398 2.125568
C -1.211659 1.622710 -0.112810
O 0.951674 -1.563176 0.046372
S 1.570830 -2.564504 -0.896285
O 3.031975 -2.578114 -0.828996
C 1.013709 -4.127830 -0.119238
O 0.980094 -2.573991 -2.234557
F 1.463917 -5.153839 -0.824007
F 1.459763 -4.217064 1.120408
F -0.315406 -4.159779 -0.109786
C -0.674557 2.886408 -0.276765
C -1.555603 3.958589 -0.424489
C -2.931035 3.750962 -0.395941
C -3.438755 2.468898 -0.212211
C -2.574301 1.385424 -0.062734
H 0.402503 3.040600 -0.306451
H -1.154549 4.961515 -0.564973
H -3.610457 4.593748 -0.510804
H -4.514225 2.301928 -0.176690
H -2.971289 0.380397 0.081818

C 3.038278 0.534501 -0.866887
O 1.988519 1.199290 -0.712131
C 3.991380 0.266479 0.208776
N 3.271867 0.051864 -2.114820
C 2.224409 0.402285 -3.063097
C 0.938219 0.486445 -2.357234
C -0.207942 0.137369 -1.955072
C -1.465697 -0.297775 -2.553214
C -1.996266 -1.563348 -2.286957
C -3.235123 -1.909776 -2.812098
C -3.953786 -1.003142 -3.586557
C -3.428479 0.260123 -3.840873
C -2.188453 0.616381 -3.326110
H 3.702954 -0.870800 -2.166889
H 2.475599 1.349002 -3.560716
H 2.135318 -0.391578 -3.816230
H -1.424821 -2.275721 -1.695045
H -3.641545 -2.899153 -2.610919
H -4.926432 -1.280557 -3.988899
H -3.987870 0.976507 -4.439910
H -1.783697 1.615728 -3.485864
C 5.332179 0.011540 -0.080166
C 6.207694 -0.244438 0.969677
C 5.747767 -0.261966 2.276113
C 4.398081 -0.015210 2.562087
C 3.513790 0.267065 1.524302
H 5.691239 0.041850 -1.106844
H 7.259490 -0.435305 0.766746
H 6.414393 -0.472321 3.110185
O 4.053646 -0.072976 3.862199
H 2.456482 0.447839 1.708393
C 2.688165 0.102522 4.186883
H 2.614729 -0.029013 5.268942
H 2.336703 1.108654 3.915862
H 2.054801 -0.637801 3.678108

TS_{ar}^{B}

56
Energy: -4154.44594850
Cu 0.937297 -0.669432 0.377904
Cl 0.716670 -1.348835 2.428223
C 2.704774 -1.460230 0.219091
O -0.646496 0.535340 0.514783
S -0.842897 1.979663 0.137965
O -1.336985 2.145342 -1.236591
C -2.287006 2.366052 1.201610
O 0.246730 2.852882 0.558397
F -2.685974 3.607177 0.963489
F -3.274890 1.528265 0.920023
F -1.959680 2.248636 2.475168
C 2.781004 -2.583936 -0.594335
C 3.808071 -3.499258 -0.373225
C 4.746391 -3.268656 0.626815
C 4.660382 -2.125414 1.420426
C 3.634451 -1.211253 1.223652
H 2.044804 -2.754727 -1.377263
H 3.873221 -4.389634 -0.995831
H 5.553413 -3.980864 0.788256
H 5.387391 -1.948293 2.210682
H 3.556166 -0.319192 1.841894
C -1.353432 -1.386593 -1.166689
O -0.424341 -2.087582 -0.736119
C -2.719107 -1.435428 -0.610045
N -1.125376 -0.481566 -2.161127
C 0.244099 -0.270429 -2.546281
C 1.216333 -0.030319 -1.451533
C 2.355759 0.191925 -0.956836
C 3.522554 1.042314 -0.844819
C 3.340372 2.379177 -0.472868
C 4.445971 3.220322 -0.411166
C 5.712820 2.737963 -0.722890
C 5.888859 1.404072 -1.090732
C 4.798989 0.549196 -1.141006
H -1.674200 0.378414 -2.107308
H 0.611039 -1.145566 -3.103150
H 0.279959 0.595569 -3.220829
H 2.340348 2.737206 -0.223862
H 4.311977 4.259006 -0.116290
H 6.573315 3.403207 -0.675323
H 6.881585 1.030041 -1.333153
H 4.921790 -0.499412 -1.410542

C -3.818235 -0.991584 -1.338335
C -5.082407 -0.970769 -0.747623
C -5.232657 -1.388072 0.575486
C -4.121638 -1.833635 1.291391
C -2.864490 -1.869091 0.712078
H -3.730931 -0.674002 -2.376043
O -6.092310 -0.533603 -1.537761
H -6.207177 -1.370666 1.057340
H -4.248802 -2.147612 2.325580
H -1.986633 -2.186085 1.272386
C -7.380099 -0.464198 -0.978953
H -8.036282 -0.079809 -1.763980
H -7.739429 -1.455599 -0.663112
H -7.411018 0.219443 -0.117072

I_2

56
Energy: -4154.50460161
Cu -0.206220 0.301680 0.608906
Cl -1.651177 1.576402 1.709286
C 2.373907 -0.469565 1.661979
O 0.904901 1.691016 0.033985
S 0.787018 1.884079 -1.482019
O -0.468185 1.312590 -1.976432
C 0.591576 3.707582 -1.562143
O 2.013728 1.561975 -2.194899
F 0.478365 4.055939 -2.835274
F -0.490214 4.073283 -0.906887
F 1.654349 4.292986 -1.039509
C 1.371882 -0.411528 2.640007
C 1.559244 0.338565 3.797605
C 2.752970 1.020064 3.996924
C 3.767214 0.937729 3.043556
C 3.582488 0.197046 1.884607
H 0.470802 -1.018065 2.535497
H 0.764375 0.387001 4.539138
H 2.899404 1.612348 4.898183
H 4.705058 1.467796 3.200386
H 4.361383 0.157297 1.124032
C -1.811310 -1.580125 -0.381310
O -1.365583 -1.151446 0.721516
C -3.255693 -1.832997 -0.487539
N -1.032341 -1.779962 -1.443987
C 0.406269 -1.526222 -1.493282
C 0.949417 -0.947896 -0.238045
C 2.118033 -1.150246 0.374573
C 3.188208 -1.979021 -0.228888
C 3.594701 -1.752475 -1.547983
C 4.605158 -2.526334 -2.109913
C 5.216052 -3.527642 -1.361823
C 4.826965 -3.745705 -0.041608
C 3.824858 -2.970091 0.526237
H -1.500510 -1.923030 -2.331020
H 0.925450 -2.481580 -1.676174
H 0.585556 -0.867977 -2.354873
H 3.146455 -0.931976 -2.110471
H 4.924833 -2.335650 -3.132946
H 6.006184 -4.132841 -1.803131
H 5.310605 -4.522039 0.548898
H 3.521509 -3.128885 1.561042
C -3.794948 -2.807497 -1.326211
C -5.179859 -2.939162 -1.387454
C -6.001543 -2.104786 -0.645231
C -5.452588 -1.122461 0.189912
C -4.069337 -0.995203 0.282039
H -3.150722 -3.484173 -1.886330
H -5.624632 -3.704372 -2.020432
H -7.085384 -2.190121 -0.691706
O -6.326264 -0.350462 0.861121
H -3.592947 -0.245031 0.912382
C -5.797831 0.678791 1.677439
H -6.658766 1.195449 2.107198
H -5.173966 0.270907 2.485410
H -5.196478 1.387636 1.090989

TS_{rc}^{B}

56
Energy: -4154.47037204
Cu -0.239157 -1.296554 0.294417
Cl 0.856601 -3.049179 -0.413057

C -0.577135 1.981471 1.282676
O -1.939327 -1.121643 1.135671
S -3.110030 -1.035281 0.166142
O -2.662522 -1.035117 -1.228214
C -3.883910 -2.678422 0.415124
O -4.099619 -0.051171 0.575895
F -4.933604 -2.792765 -0.384442
F -3.009603 -3.629200 0.126321
F -4.275200 -2.810145 1.672125
C -0.181712 1.111491 2.310154
C -0.098734 1.549643 3.620558
C -0.410949 2.869200 3.939389
C -0.809148 3.743810 2.933867
C -0.893833 3.305758 1.619278
H 0.038080 0.073521 2.078966
H 0.196869 0.851042 4.400787
H -0.352976 3.210549 4.971436
H -1.060763 4.775375 3.173350
H -1.204559 3.998204 0.839241
C 2.245391 -0.267084 -0.911475
O 1.609788 -0.090071 0.162711
C 3.689698 -0.530446 -0.902894
N 1.587130 -0.146196 -2.067330
C 0.136287 -0.040288 -2.005090
C -0.198538 0.394850 -0.617440
C -0.633904 1.560419 -0.119722
C -1.305130 2.453599 -1.112531
C -2.698721 2.522347 -1.167081
C -3.307222 3.377912 -2.079672
C -2.536386 4.160961 -2.934251
C -1.146674 4.090525 -2.881469
C -0.530617 3.235411 -1.974066
H 2.049746 -0.351155 -2.943340
H -0.218167 0.727140 -2.705922
H -0.333976 -0.997545 -2.271406
H -3.297489 1.895146 -0.504187
H -4.393494 3.425176 -2.126477
H -3.019758 4.827150 -3.646865
H -0.540767 4.705784 -3.544642
H 0.557880 3.175364 -1.918123
C 4.287687 -1.279627 -1.913913
C 5.659955 -1.509250 -1.859232
C 6.416920 -0.996454 -0.819922
C 5.809648 -0.258447 0.204538
C 4.437397 -0.029273 0.167793
H 3.688038 -1.743143 -2.695933
H 6.140427 -2.106869 -2.630822
H 7.490113 -1.165272 -0.760516
O 6.632919 0.184258 1.176387
H 3.924771 0.529070 0.946230
C 6.063082 0.891371 2.254245
H 6.886492 1.135516 2.929297
H 5.584467 1.822570 1.916702
H 5.323990 0.278441 2.790338

Reaction 27

reactant

35
Energy: -861.349375354
C 0.099602 -0.815301 -0.011702
O 0.094976 -1.180258 1.151035
C 1.339295 -0.597654 -0.822531
N -1.063093 -0.621041 -0.708411
H -1.050701 -0.131626 -1.593368
C -2.336426 -0.780775 -0.025430
C -3.447590 -0.632002 -0.951350
H -2.348604 -1.771795 0.451423
H -2.421265 -0.055403 0.800644
C -4.366035 -0.494900 -1.729142
C -5.453204 -0.332504 -2.639426
C -5.670220 -1.265986 -3.662731
C -6.728250 -1.102420 -4.546438
C -7.583297 -0.010568 -4.420859
C -7.375969 0.920629 -3.406509
C -6.318341 0.764473 -2.521165
H -4.998453 -2.118078 -3.751035
H -6.888374 -1.832757 -5.337787
H -8.413090 0.114445 -5.114432
H -8.043038 1.775027 -3.305353
H -6.147708 1.487679 -1.725586

C 1.451102 -1.169237 -2.087825
C 2.615177 -1.045917 -2.840479
C 3.680692 -0.328220 -2.315941
C 3.591586 0.263644 -1.058616
C 2.424990 0.133770 -0.303075
H 0.611654 -1.747978 -2.472981
H 2.688189 -1.511731 -3.820769
H 4.601458 -0.219491 -2.887167
H 4.436367 0.829446 -0.674476
O 2.246590 0.704665 0.905508
C 3.314253 1.423087 1.468784
H 2.965879 1.770605 2.444469
H 4.200583 0.786400 1.609056
H 3.590228 2.292683 0.852611

I_c

70
Energy: -4461.97202338
Cu -1.448576 0.411906 0.611278
Cl -2.687156 -0.008636 2.352450
C -1.667322 2.225755 1.149953
O -1.383480 -1.529363 0.123224
S -0.628678 -2.266642 -0.949410
O 0.727621 -1.767539 -1.177278
C -0.382911 -3.875758 -0.101991
O -1.436376 -2.573878 -2.124206
F 0.419767 -4.644674 -0.822869
F 0.183940 -3.681722 1.092607
F -1.537670 -4.491915 0.076039
C -0.476802 2.884623 1.390414
C -0.531058 4.271275 1.568568
C -1.744266 4.945169 1.533756
C -2.925753 4.238910 1.320679
C -2.897426 2.858088 1.134680
C 2.430361 1.656832 -0.817989
O 2.292931 2.709495 -0.211993
C 3.715065 0.892505 -0.874695
N 1.417485 1.116626 -1.574464
C 0.179378 1.821107 -1.723959
C -1.057383 1.134435 -1.296202
C -2.239800 0.799254 -1.444723
C -3.624364 0.510320 -1.559891
C -4.090156 -0.810289 -1.676490
C -5.453362 -1.039144 -1.784102
C -6.350332 0.026965 -1.785911
C -5.890983 1.339018 -1.683954
C -4.532617 1.586158 -1.566272
H 0.487011 2.381856 1.388381
H 0.404567 4.808290 1.717674
H -1.773788 6.023463 1.680232
H -3.884623 4.755635 1.315283
H -3.820129 2.295017 1.006017
H 1.473681 0.160507 -1.908800
H 0.281931 2.753403 -1.148015
H 0.019896 2.101779 -2.777531
H -3.373103 -1.629195 -1.688818
H -5.820009 -2.059789 -1.869434
H -7.418571 -0.165325 -1.871222
H -6.595034 2.168553 -1.694633
H -4.151718 2.603406 -1.487469
O 2.071718 -1.445782 1.668191
C 2.576053 -2.544131 2.440763
C 3.454279 -3.349332 1.521118
C 1.005082 -0.755603 2.042940
O 0.606077 0.119691 1.288288
C 0.346920 -1.081032 3.346458
H 3.136919 -2.146656 3.299155
H 1.741618 -3.146932 2.818778
H 3.823485 -4.246012 2.031891
H 2.880864 -3.654479 0.635978
H 4.311388 -2.752712 1.189627
H 1.070671 -1.308011 4.136297
H -0.291354 -0.246622 3.644337
H -0.304071 -1.955687 3.205460
C 3.720561 -0.501355 -0.894603
C 4.908933 -1.219473 -0.991332
C 6.113740 -0.535493 -1.059728
C 6.138067 0.855180 -1.038280
C 4.946861 1.579964 -0.953062
H 2.777606 -1.040430 -0.808611
H 4.885023 -2.307190 -1.026389

H 7.053014 -1.081350 -1.138676
H 7.091644 1.372090 -1.103633
O 4.904519 2.928034 -0.983426
C 6.111799 3.633783 -1.101433
H 5.843845 4.693394 -1.111165
H 6.781503 3.441201 -0.249392
H 6.640754 3.386351 -2.034867

I_1

56
Energy: -4154.45700327
Cu 0.057657 -0.123957 0.090154
Cl 0.171611 -0.236804 2.215316
C 1.928935 -0.314328 0.279619
O -1.924250 0.387113 -0.043949
S -1.771999 1.823261 -0.487714
O -2.486913 2.178106 -1.699287
C -2.567933 2.756545 0.875939
O -0.334905 2.167868 -0.410368
F -2.486304 4.051971 0.615939
F -3.840237 2.404336 0.965699
F -1.957144 2.498098 2.017447
C 2.562038 -1.537482 0.184859
C 3.949407 -1.513414 0.021741
C 4.642094 -0.305146 -0.013598
C 3.959405 0.899541 0.117461
C 2.573971 0.908561 0.289625
H 2.011027 -2.477871 0.226457
H 4.483686 -2.457884 -0.075669
H 5.724492 -0.304599 -0.131152
H 4.498298 1.845610 0.104056
H 2.027482 1.841326 0.420573
C -0.745937 -3.365877 0.425756
O 0.227394 -3.775168 -0.190198
C -1.062191 -3.704695 1.839049
N -1.667274 -2.543477 -0.183029
C -1.413523 -2.108635 -1.535723
C -0.310813 -1.131626 -1.681756
C 0.486395 -0.369947 -2.229385
C 1.481725 0.444039 -2.831173
C 1.229925 1.799981 -3.094317
C 2.227124 2.579457 -3.660804
C 3.464255 2.017736 -3.970255
C 3.713424 0.669616 -3.719527
C 2.726688 -0.120337 -3.150746
H -2.277619 -1.968461 0.388365
H -1.160410 -2.985837 -2.144007
H -2.328402 -1.654659 -1.933033
H 0.263348 2.222990 -2.829263
H 2.039764 3.632280 -3.860897
H 4.242706 2.637290 -4.412794
H 4.681853 0.236924 -3.962712
H 2.905172 -1.170923 -2.927560
C -0.051148 -3.690997 2.820858
C -0.379397 -3.991710 4.143497
C -1.687121 -4.324263 4.482893
C -2.688444 -4.350624 3.521182
C -2.366740 -4.029401 2.207548
O 1.188476 -3.349592 2.421275
H 0.385113 -3.969567 4.915559
H -1.919225 -4.563741 5.519484
H -3.709107 -4.614179 3.788733
H -3.137540 -4.047877 1.437523
C 2.146968 -3.040137 3.404945
H 3.012546 -2.640593 2.868078
H 1.772083 -2.273165 4.098010
H 2.450320 -3.934212 3.969914

TS_{rc}^{A}

56
Energy: -4154.43626603
Cu 0.671877 0.447668 -0.450384
Cl 0.467614 0.760812 -2.570289
C 1.945568 1.837443 -0.433355
O -0.854132 -0.799355 -0.347793
S -1.100524 -2.197448 0.158495
O -2.365069 -2.289225 0.894756
C -1.365865 -3.097110 -1.415162
O 0.068288 -2.824409 0.768546
F -1.611415 -4.373017 -1.160055

F -2.383529 -2.578868 -2.079078
F -0.266658 -3.004679 -2.153359
C 1.486815 3.068022 0.007158
C 2.392797 4.130268 0.034755
C 3.708155 3.950281 -0.377725
C 4.131810 2.703105 -0.826137
C 3.242519 1.630427 -0.865629
H 0.460032 3.201501 0.342105
H 2.056275 5.104558 0.387303
H 4.405539 4.786007 -0.354254
H 5.157870 2.556787 -1.160384
H 3.570625 0.656595 -1.229733
C -1.665409 0.847807 1.769197
O -0.615693 1.476462 1.525520
C -2.956044 1.113830 1.140368
N -1.569940 -0.147636 2.704551
C -0.211003 -0.272814 3.213796
C 0.742495 0.011395 2.131905
C 1.537679 -0.218262 1.178859
C 2.777989 -0.942117 0.942383
C 2.891715 -1.845431 -0.120792
C 4.109503 -2.462664 -0.373362
C 5.222243 -2.175243 0.413118
C 5.112037 -1.271457 1.465489
C 3.895850 -0.655528 1.732625
H -2.078550 -1.010501 2.490490
H -0.069341 0.410095 4.062298
H -0.046452 -1.304626 3.551252
H 2.018280 -2.084410 -0.725880
H 4.188435 -3.171332 -1.195363
H 6.176505 -2.655243 0.203804
H 5.978699 -1.040186 2.082320
H 3.805871 0.072762 2.537236
C -4.124190 0.604412 1.720728
C -5.364738 0.795533 1.139187
C -5.442747 1.505628 -0.055183
C -4.305113 2.029092 -0.653432
C -3.048902 1.853188 -0.064578
H -4.048412 0.062314 2.661109
H -6.259782 0.394545 1.607811
H -6.408414 1.661883 -0.533358
H -4.397275 2.577176 -1.586243
O -1.920403 2.350667 -0.588668
C -1.978204 3.002258 -1.844332
H -0.947189 3.267771 -2.089053
H -2.358951 2.326472 -2.621304
H -2.598946 3.907862 -1.787643

TS_{ar}^{B}

56
Energy: -4154.43782558
Cu -0.531211 0.369260 0.478873
Cl -0.163040 0.568396 2.603034
C -2.035417 1.601525 0.562545
O 0.790674 -1.095933 0.322590
S 0.561212 -2.479157 -0.232175
O 1.252920 -2.677302 -1.511460
C 1.504184 -3.476850 0.985027
O -0.822371 -2.930966 -0.134884
F 1.434909 -4.755143 0.643816
F 2.775626 -3.097540 0.987549
F 0.994481 -3.315246 2.190554
C -1.842705 2.828838 -0.057991
C -2.621604 3.910753 0.350550
C -3.583357 3.746932 1.341194
C -3.771487 2.500446 1.937513
C -2.995541 1.416060 1.552179
H -1.088595 2.942862 -0.833845
H -2.474412 4.881543 -0.119476
H -4.194939 4.593042 1.649320
H -4.517715 2.371335 2.719213
H -3.133616 0.440622 2.014157
C 1.798555 0.977795 -1.171118
O 0.986598 1.735887 -0.624685
C 3.223026 0.871142 -0.774264
N 1.387674 0.166966 -2.182980
C -0.011141 0.124594 -2.518895
C -0.982502 0.101585 -1.398749
C -2.146960 0.140112 -0.920103
C -3.518002 -0.321539 -0.908806
C -3.758333 -1.693975 -0.775403

C -5.067699 -2.160054 -0.819795
C -6.122364 -1.271325 -1.001265
C -5.877822 0.095757 -1.132250
C -4.579286 0.576416 -1.075005
H 1.917715 -0.678379 -2.372432
H -0.270277 1.008554 -3.121823
H -0.180881 -0.766726 -3.137424
H -2.914978 -2.369097 -0.631208
H -5.261656 -3.225268 -0.711480
H -7.144952 -1.643266 -1.037838
H -6.705499 0.787976 -1.273786
H -4.372789 1.642848 -1.159332
C 3.818327 -0.383766 -0.639254
C 5.124469 -0.513208 -0.184611
C 5.848527 0.629824 0.126319
C 5.283457 1.893320 -0.016119
C 3.968367 2.026417 -0.463649
H 3.236637 -1.281088 -0.845676
H 5.560946 -1.501481 -0.060168
H 6.872388 0.547110 0.487620
H 5.873974 2.773187 0.224287
O 3.362541 3.213786 -0.663489
C 4.050466 4.384849 -0.299591
H 3.365102 5.213044 -0.495580
H 4.314535 4.380744 0.767913
H 4.963684 4.522609 -0.898272

I_2

56
Energy: -4154.49845103
Cu -0.262043 0.439060 0.634879
Cl -1.324688 1.892276 1.917697
C 2.240334 -0.722438 1.598924
O 1.033023 1.655210 0.045661
S 0.875367 1.920959 -1.454874
O -0.476499 1.574965 -1.899840
C 0.973710 3.753664 -1.469850
O 2.000346 1.436740 -2.240948
F 0.868173 4.165521 -2.725109
F -0.008275 4.262971 -0.755491
F 2.136843 4.141499 -0.977488
C 1.293868 -0.541644 2.615620
C 1.637496 0.120087 3.790412
C 2.932947 0.587495 3.970009
C 3.889385 0.381739 2.976299
C 3.548368 -0.269637 1.799751
H 0.298807 -0.979165 2.520512
H 0.882765 0.270876 4.559547
H 3.202624 1.109607 4.886179
H 4.906151 0.744871 3.116148
H 4.286752 -0.404951 1.010356
C -2.110714 -1.325332 -0.286481
O -1.628282 -0.820624 0.762097
C -3.543928 -1.669641 -0.321360
N -1.388957 -1.605206 -1.372853
C 0.052817 -1.409293 -1.515360
C 0.691009 -0.942806 -0.262556
C 1.843722 -1.312473 0.302968
C 2.776817 -2.252119 -0.364752
C 3.169373 -2.034102 -1.689632
C 4.055368 -2.910025 -2.309198
C 4.556321 -4.005488 -1.613288
C 4.181906 -4.217969 -0.287953
C 3.304144 -3.341900 0.336824
H -1.907179 -1.847384 -2.208820
H 0.503963 -2.375889 -1.792560
H 0.212837 -0.704620 -2.344428
H 2.811738 -1.145386 -2.212099
H 4.365760 -2.725828 -3.336303
H 5.249983 -4.689699 -2.099229
H 4.580297 -5.068907 0.262162
H 3.015377 -3.496090 1.376485
C -3.970145 -2.926380 -0.749155
C -5.318405 -3.258658 -0.734986
C -6.241118 -2.313380 -0.296245
C -5.840607 -1.047267 0.116686
C -4.485747 -0.707313 0.103387
H -3.226214 -3.661706 -1.056467
H -5.644821 -4.246252 -1.051271
H -7.301106 -2.561678 -0.277813
H -6.582055 -0.319766 0.436873

O -3.993571 0.490571 0.418052
C -4.834018 1.475903 0.980155
H -4.166759 2.294891 1.258843
H -5.581102 1.822121 0.251316
H -5.340274 1.093662 1.877476

TS_{rc}^{B}

56
Energy: -4154.46750411
Cu -0.206389 -1.313675 0.367865
Cl 0.820269 -3.151359 -0.257482
C -0.495992 1.998286 1.259235
O -1.950066 -1.070622 1.101692
S -3.054134 -0.965237 0.060237
O -2.524198 -1.022123 -1.303813
C -3.910042 -2.567605 0.304605
O -4.025821 0.069141 0.380624
F -4.920884 -2.658990 -0.546801
F -3.064331 -3.561853 0.086059
F -4.372637 -2.650142 1.541660
C -0.192021 1.144150 2.330021
C -0.183836 1.612021 3.632572
C -0.481816 2.946266 3.899867
C -0.790211 3.805678 2.850647
C -0.799857 3.337876 1.543701
H 0.017119 0.096315 2.137664
H 0.043130 0.925850 4.446024
H -0.482814 3.311028 4.925576
H -1.030877 4.848493 3.049521
H -1.041772 4.018175 0.729323
C 2.369423 -0.419257 -0.678698
O 1.661518 -0.193114 0.338462
C 3.790621 -0.788836 -0.585016
N 1.805255 -0.315514 -1.882397
C 0.361885 -0.123988 -1.930232
C -0.041325 0.353172 -0.573531
C -0.472576 1.543402 -0.133214
C -1.043735 2.432020 -1.190786
C -2.425748 2.518192 -1.370005
C -2.937928 3.368773 -2.344713
C -2.083008 4.128719 -3.137794
C -0.704626 4.040644 -2.960304
C -0.184270 3.191073 -1.989979
H 2.326157 -0.580171 -2.709169
H 0.106788 0.650733 -2.665175
H -0.143843 -1.056989 -2.216850
H -3.090776 1.909464 -0.754414
H -4.014997 3.430253 -2.488400
H -2.491419 4.790777 -3.899521
H -0.033118 4.637588 -3.575375
H 0.893822 3.116676 -1.835848
C 4.222217 -1.967496 -1.191794
C 5.547459 -2.369441 -1.080080
C 6.435314 -1.580025 -0.359040
C 6.020591 -0.400398 0.253404
C 4.690064 0.003833 0.150597
H 3.489649 -2.597808 -1.696619
H 5.877195 -3.298905 -1.537173
H 7.475948 -1.884644 -0.261662
H 6.737683 0.198723 0.808101
O 4.189919 1.136445 0.678273
C 5.032031 1.931744 1.481931
H 4.413589 2.755959 1.844056
H 5.417118 1.363167 2.340230
H 5.875663 2.335626 0.903291

Reaction 28

reactant

33
Energy: -951.305678900
C -0.007125 -0.186140 -0.089760
O 0.008151 -0.228779 1.130912
C 1.257073 -0.109833 -0.891078
N -1.170466 -0.206882 -0.797383
H -1.189903 0.050614 -1.774928
C -2.437788 -0.214005 -0.077851
C -3.557207 -0.285224 -1.001579
H -2.436846 -1.070798 0.611118
H -2.512943 0.681063 0.560717

C -4.484478 -0.335941 -1.779161
C -5.581204 -0.401601 -2.690212
C -5.966801 -1.629136 -3.247139
C -7.034255 -1.690411 -4.132233
C -7.729103 -0.532402 -4.471686
C -7.353004 0.690763 -3.922693
C -6.285897 0.759525 -3.037637
H -5.418658 -2.528993 -2.973498
H -7.327306 -2.647826 -4.559377
H -8.566481 -0.583442 -5.165431
H -7.895496 1.597066 -4.185972
H -5.984928 1.710871 -2.602396
C 1.342672 -0.499978 -2.230281
C 2.548363 -0.410401 -2.911011
C 3.657552 0.073982 -2.231572
C 3.605466 0.454961 -0.896659
C 2.395966 0.352321 -0.228155
H 0.475702 -0.912687 -2.744015
H 2.646784 -0.713344 -3.949157
N 4.936438 0.177649 -2.951245
H 4.504569 0.819178 -0.408384
H 2.306508 0.622199 0.821663
O 5.896229 0.604935 -2.332285
O 4.953031 -0.169245 -4.120677

I_c

68
Energy: -4551.93585079
Cu -1.576292 0.411514 0.536210
Cl -2.275366 0.180439 2.575224
C -2.123324 2.207236 0.828575
O -1.116334 -1.530229 0.367741
S -0.446738 -2.298838 -0.738636
O 0.710508 -1.611402 -1.321543
C 0.283120 -3.671166 0.237531
O -1.379492 -2.936870 -1.659306
F 1.071210 -4.401701 -0.535716
F 1.014044 -3.179796 1.241628
F -0.668475 -4.434304 0.743333
C -1.087269 3.116215 0.747013
C -1.422361 4.470518 0.652133
C -2.749300 4.876366 0.686005
C -3.763664 3.930447 0.819775
C -3.454619 2.574878 0.904314
C 1.384861 2.144979 -1.636218
O 1.053510 3.315722 -1.496241
C 2.765389 1.686949 -1.295201
N 0.553373 1.175376 -2.111695
C -0.831981 1.487248 -2.344267
C -1.794395 0.744891 -1.502762
C -2.870005 0.159841 -1.321699
C -4.133502 -0.420767 -1.051368
C -4.265279 -1.800119 -0.813212
C -5.522335 -2.325018 -0.556833
C -6.643384 -1.497494 -0.547757
C -6.519284 -0.131720 -0.798681
C -5.269238 0.412244 -1.045163
H -0.045782 2.811025 0.711440
H -0.618264 5.195070 0.533896
H -2.997376 5.934081 0.619954
H -4.805573 4.242759 0.875982
H -4.236926 1.831074 1.047194
H 0.779333 0.197013 -1.917705
H -0.952817 2.564999 -2.157396
H -1.105528 1.295593 -3.392693
H -3.379687 -2.431810 -0.846326
H -5.630145 -3.390617 -0.366789
H -7.626064 -1.921371 -0.347995
H -7.399986 0.507079 -0.800502
H -5.149134 1.475310 -1.248875
O 2.434784 -0.550269 0.991557
C 3.305152 -1.432585 1.722315
C 4.016567 -2.310468 0.724465
C 1.359843 -0.008683 1.543525
O 0.624398 0.657572 0.825346
C 1.091194 -0.225129 3.000107
H 4.006607 -0.820718 2.308962
H 2.713756 -2.044047 2.413389
H 4.541095 -3.117684 1.248785
H 3.287806 -2.754140 0.035702
H 4.755749 -1.753943 0.138539

H 2.003082 -0.180231 3.605170
H 0.370124 0.520340 3.342135
H 0.629493 -1.212717 3.138851
C 3.363392 0.576510 -1.891788
C 4.651456 0.207366 -1.535010
C 5.310110 0.947674 -0.561797
C 4.741431 2.065943 0.037293
C 3.464710 2.437932 -0.348505
H 2.832066 0.000181 -2.646563
H 5.148090 -0.643977 -1.991532
H 5.304421 2.619239 0.783358
H 2.980027 3.314429 0.076569
N 6.650359 0.523849 -0.138735
O 7.074639 -0.532386 -0.583599
O 7.252521 1.243635 0.640894

I_1

54
Energy: -4244.40764566
Cu 0.035012 -0.004818 -0.003765
Cl 0.085329 -0.102252 2.124084
C 1.902061 -0.023858 0.280198
O -1.988093 0.304044 -0.235400
S -1.932364 1.731888 -0.728521
O -2.618232 1.987840 -1.980091
C -2.851304 2.644292 0.570211
O -0.526557 2.183941 -0.608108
F -2.836153 3.935431 0.283908
F -4.101100 2.212378 0.604472
F -2.282586 2.444949 1.744553
C 2.637532 -1.191911 0.268546
C 4.021434 -1.062791 0.142491
C 4.611897 0.196605 0.065770
C 3.828903 1.344681 0.118346
C 2.442290 1.247883 0.252655
H 2.160754 -2.170290 0.331280
H 4.635125 -1.961951 0.111524
H 5.693685 0.282554 -0.019463
H 4.288779 2.330555 0.073865
H 1.818068 2.136834 0.326778
C -0.606696 -3.214874 0.599794
O 0.443696 -3.631576 0.127970
C -0.971339 -3.425591 2.030833
N -1.512392 -2.531405 -0.166093
C -1.174489 -2.230026 -1.539684
C -0.169913 -1.158079 -1.716964
C 0.571911 -0.359882 -2.289372
C 1.514054 0.509456 -2.898775
C 1.159509 1.825778 -3.234575
C 2.109472 2.661993 -3.801434
C 3.400716 2.195109 -4.040044
C 3.752117 0.885681 -3.717753
C 2.813819 0.040321 -3.147048
H -2.184356 -1.914063 0.278893
H -0.782959 -3.141089 -2.007813
H -2.091689 -1.932863 -2.061053
H 0.151026 2.176017 -3.025256
H 1.842338 3.684936 -4.057691
H 4.140964 2.858961 -4.483696
H 4.761869 0.527023 -3.907080
H 3.070997 -0.981157 -2.872019
C 0.072894 -3.682693 2.921530
C -0.187001 -3.873694 4.268390
C -1.505867 -3.817104 4.701031
C -2.565156 -3.579483 3.836902
C -2.288923 -3.381826 2.491601
H 1.089652 -3.717060 2.536357
H 0.604840 -4.062547 4.986964
N -1.792787 -4.025665 6.129298
H -3.578989 -3.557070 4.225095
H -3.112916 -3.221017 1.798688
O -2.959496 -3.975870 6.481194
O -0.845266 -4.236751 6.867258

TS_{rc}^{A}

54
Energy: -4244.38235234
Cu -0.988851 -0.649404 -0.601568
Cl -0.160578 -1.514076 -2.379318
C -2.282220 -2.015168 -0.620740

O 0.232570 0.909875 -0.627633
S 0.531543 2.161481 0.157014
O 1.857506 2.129818 0.780032
C 0.640169 3.389105 -1.199209
O -0.569527 2.617925 1.004795
F 0.903558 4.581150 -0.689294
F 1.589796 3.053748 -2.051485
F -0.525766 3.436079 -1.834828
C -2.092004 -3.164354 0.124412
C -3.112399 -4.115525 0.134094
C -4.278187 -3.906229 -0.596122
C -4.431508 -2.745832 -1.347624
C -3.422807 -1.783225 -1.369244
H -1.179921 -3.318639 0.697996
H -2.987764 -5.024629 0.720824
H -5.068528 -4.654656 -0.584517
H -5.336546 -2.582655 -1.930655
H -3.542833 -0.873762 -1.958332
C 1.301932 -0.739031 1.728518
O 0.346848 -1.440084 1.327150
C 2.655285 -0.845516 1.170924
N 1.037381 0.142168 2.722912
C -0.361466 0.103130 3.135988
C -1.181550 -0.248742 1.967300
C -1.938924 -0.033268 0.973032
C -3.239793 0.605227 0.790759
C -3.392557 1.716168 -0.044043
C -4.659569 2.246630 -0.251707
C -5.774720 1.671016 0.351395
C -5.621754 0.557710 1.171987
C -4.358999 0.022899 1.393682
H 1.516396 1.042783 2.668386
H -0.482212 -0.615519 3.957482
H -0.670117 1.100940 3.473025
H -2.517043 2.174601 -0.499954
H -4.774184 3.117997 -0.893556
H -6.764692 2.088881 0.177894
H -6.490040 0.098518 1.641116
H -4.230315 -0.870325 2.004887
C 3.768545 -0.451503 1.920292
C 5.037849 -0.555660 1.377341
C 5.162469 -1.050349 0.085459
C 4.072972 -1.445139 -0.677829
C 2.804750 -1.347303 -0.125020
H 3.643117 -0.081820 2.936094
H 5.926452 -0.265491 1.929272
N 6.513756 -1.163528 -0.494885
H 4.226275 -1.816344 -1.686568
H 1.929170 -1.631747 -0.708113
O 6.601846 -1.605783 -1.626342
O 7.451418 -0.809169 0.199345

TS_{ar}^{B}

54
Energy: -4244.39492170
Cu -0.964369 -0.670946 -0.265588
Cl -0.411577 -1.538884 -2.174924
C -2.674504 -1.588216 -0.251780
O 0.545040 0.628268 -0.330402
S 0.516751 2.115489 -0.079480
O 0.826156 2.453196 1.318208
C 2.014671 2.609037 -1.016731
O -0.620661 2.791407 -0.690377
F 2.194079 3.914209 -0.892378
F 3.066274 1.973189 -0.516323
F 1.877524 2.301211 -2.291387
C -2.782433 -2.656636 0.629223
C -3.709270 -3.658896 0.348451
C -4.520182 -3.565130 -0.777501
C -4.406522 -2.473585 -1.637867
C -3.477249 -1.474770 -1.382454
H -2.147693 -2.717213 1.511192
H -3.798093 -4.508659 1.022623
H -5.250073 -4.345016 -0.985995
H -5.033449 -2.404386 -2.524671
H -3.376716 -0.622080 -2.051182
C 1.167751 -1.074992 1.585224
O 0.353535 -1.906503 1.162833
C 2.584240 -1.070090 1.152418
N 0.772196 -0.091277 2.440590
C -0.645975 0.025018 2.665536

C -1.506897 0.076765 1.456343
C -2.606210 0.162129 0.843606
C -3.830886 0.877525 0.559606
C -3.742600 2.210888 0.142047
C -4.912238 2.930261 -0.075880
C -6.151355 2.331548 0.126375
C -6.233858 1.001652 0.539672
C -5.077328 0.266691 0.745534
H 1.235294 0.814425 2.328100
H -0.994018 -0.829740 3.263394
H -0.825159 0.940065 3.245288
H -2.761702 2.658328 -0.021268
H -4.850783 3.964832 -0.406832
H -7.063069 2.901776 -0.043103
H -7.205286 0.537054 0.696125
H -5.125267 -0.776917 1.054772
C 3.580942 -0.407376 1.873175
C 4.882285 -0.381882 1.394879
C 5.156057 -1.022663 0.194280
C 4.182166 -1.684549 -0.542304
C 2.885386 -1.708729 -0.053778
H 3.348794 0.071337 2.822860
H 5.683129 0.118074 1.930946
N 6.537433 -0.997939 -0.316464
H 4.448644 -2.158654 -1.482064
H 2.084754 -2.190184 -0.613570
O 6.757431 -1.572400 -1.368678
O 7.371241 -0.406387 0.348951

I_2

54
Energy: -4244.44640850
Cu -0.240979 -0.522290 0.727774
Cl 0.706841 -2.082104 1.963546
C -2.609474 0.918225 1.541919
O -1.622751 -1.587531 0.060817
S -1.413432 -1.867802 -1.433026
O -0.009596 -1.662846 -1.799878
C -1.703759 -3.680937 -1.466454
O -2.434695 -1.261147 -2.273006
F -1.595936 -4.093867 -2.720200
F -0.808327 -4.292065 -0.720491
F -2.918708 -3.942412 -1.019617
C -1.738259 0.617213 2.598221
C -2.207023 -0.035352 3.734974
C -3.550125 -0.374298 3.834989
C -4.428884 -0.044838 2.803335
C -3.965005 0.598594 1.664896
H -0.704896 0.967640 2.570861
H -1.513719 -0.278197 4.537627
H -3.918033 -0.890370 4.719656
H -5.482726 -0.305743 2.883013
H -4.643860 0.827453 0.844261
C 1.875292 0.889371 -0.144517
O 1.275072 0.562874 0.912629
C 3.352926 0.876488 -0.119209
N 1.253302 1.235319 -1.270030
C -0.197933 1.318449 -1.446655
C -0.961195 0.954448 -0.228844
C -2.082648 1.473695 0.277640
C -2.845971 2.536362 -0.417049
C -3.183541 2.395452 -1.767260
C -3.907269 3.392547 -2.413656
C -4.298638 4.532878 -1.719389
C -3.980368 4.669990 -0.369670
C -3.265893 3.673531 0.281886
H 1.813157 1.278835 -2.113323
H -0.451727 2.359112 -1.707897
H -0.455544 0.677155 -2.301191
H -2.918292 1.475160 -2.290047
H -4.178061 3.269793 -3.460860
H -4.864552 5.312448 -2.226561
H -4.295079 5.556629 0.178075
H -3.021408 3.769692 1.339716
C 4.119261 1.750907 -0.895009
C 5.502832 1.686686 -0.842456
C 6.087328 0.737505 -0.014348
C 5.346999 -0.138973 0.766737
C 3.962553 -0.062215 0.719090
H 3.640328 2.510721 -1.511387
H 6.133843 2.356632 -1.418545

N 7.560264 0.666582 0.040208
H 5.858051 -0.861986 1.395488
H 3.337646 -0.730850 1.312207
O 8.051875 -0.168926 0.776840
O 8.182149 1.450112 -0.656391

TS_{rc}^{B}

54
Energy: -4244.41485098
Cu -0.419866 -1.228903 0.324382
Cl 1.024344 -2.696838 -0.374002
C -1.294052 1.898758 1.426353
O -2.189079 -1.295080 1.015778
S -3.246857 -1.336363 -0.081429
O -2.644688 -1.220042 -1.411520
C -3.794691 -3.081839 0.038866
O -4.404433 -0.514249 0.231131
F -4.716659 -3.315636 -0.881633
F -2.759092 -3.881354 -0.165540
F -4.300331 -3.317697 1.237587
C -0.862725 1.039812 2.449046
C -0.995026 1.396501 3.779471
C -1.563907 2.621072 4.122589
C -2.000804 3.482572 3.121811
C -1.869560 3.126169 1.786761
H -0.441635 0.071557 2.194955
H -0.664457 0.707942 4.554351
H -1.673617 2.897684 5.169610
H -2.451561 4.438763 3.380705
H -2.212603 3.807439 1.010534
C 2.070347 0.206059 -0.516977
O 1.332422 0.254663 0.492517
C 3.535151 0.085379 -0.375661
N 1.532316 0.315184 -1.736336
C 0.081779 0.241427 -1.852859
C -0.486092 0.509180 -0.499556
C -1.128225 1.568650 0.010061
C -1.799299 2.427532 -1.015109
C -3.161425 2.275144 -1.281853
C -3.770107 3.102720 -2.220489
C -3.030746 4.072632 -2.891075
C -1.671449 4.221120 -2.626542
C -1.054087 3.396954 -1.692174
H 2.108414 0.201989 -2.560640
H -0.279675 1.020619 -2.537569
H -0.219908 -0.738739 -2.248051
H -3.735556 1.505364 -0.762085
H -4.830498 2.980771 -2.432633
H -3.514164 4.714716 -3.625336
H -1.091633 4.983012 -3.144838
H 0.009074 3.506862 -1.470739
C 4.284078 -0.661613 -1.288272
C 5.656477 -0.776207 -1.124752
C 6.248971 -0.133958 -0.046767
C 5.522704 0.596349 0.884155
C 4.149898 0.695259 0.719852
H 3.790738 -1.206431 -2.092108
H 6.269616 -1.359496 -1.805090
N 7.709890 -0.242711 0.123373
H 6.037757 1.066312 1.716611
H 3.541873 1.245481 1.434218
O 8.203250 0.322980 1.082971
O 8.322008 -0.887117 -0.709919

Reaction 29

reactant

33
Energy: -951.305217570
C 0.007212 -0.310280 -0.068417
O 0.024693 -0.384716 1.150011
C 1.268124 -0.220699 -0.871653
N -1.158432 -0.309865 -0.775073
H -1.179500 -0.007252 -1.739574
C -2.424250 -0.326488 -0.052848
C -3.543882 -0.357716 -0.978967
H -2.431318 -1.204482 0.608711
H -2.490767 0.548859 0.613423
C -4.466327 -0.372814 -1.763810
C -5.556136 -0.394995 -2.685300

C -5.951217 -1.598127 -3.286962
C -7.010076 -1.616170 -4.184267
C -7.686932 -0.438775 -4.491749
C -7.301579 0.760346 -3.898031
C -6.243050 0.785836 -3.000495
H -5.417157 -2.513578 -3.038305
H -7.310515 -2.554992 -4.646213
H -8.517510 -0.455885 -5.195291
H -7.830058 1.681806 -4.136028
H -5.934949 1.718165 -2.530452
C 1.352293 -0.570501 -2.221257
C 2.560210 -0.475592 -2.906421
C 3.699066 -0.027679 -2.251531
C 3.599757 0.305002 -0.905939
C 2.411790 0.208327 -0.201550
H 0.476545 -0.956255 -2.742060
H 2.615033 -0.759336 -3.955033
H 4.657325 0.061905 -2.754717
N 4.806441 0.772830 -0.204006
H 2.362520 0.455987 0.855530
O 4.693970 1.066923 0.972946
O 5.840220 0.836378 -0.849263

I_c

68
Energy: -4551.93504414
Cu -1.500958 0.488858 0.514112
Cl -2.145058 0.373593 2.580364
C -1.880953 2.334782 0.762583
O -1.264291 -1.493568 0.398546
S -0.667867 -2.403732 -0.638253
O 0.527223 -1.861897 -1.292847
C -0.028418 -3.733807 0.455598
O -1.659833 -3.050310 -1.488772
F 0.715552 -4.576884 -0.246288
F 0.728838 -3.211224 1.423429
F -1.028894 -4.390601 1.013121
C -0.781789 3.156950 0.610089
C -1.015530 4.532172 0.505786
C -2.304200 5.041634 0.592177
C -3.381523 4.180762 0.789787
C -3.174597 2.806614 0.889976
C 1.677732 1.660211 -1.761345
O 1.544098 2.852104 -1.516112
C 2.996919 0.981261 -1.587285
N 0.676503 0.869827 -2.248121
C -0.646638 1.420738 -2.382099
C -1.681449 0.796173 -1.532927
C -2.809753 0.331546 -1.327988
C -4.125315 -0.109066 -1.038443
C -4.399406 -1.464345 -0.784417
C -5.702735 -1.850255 -0.511826
C -6.729466 -0.908257 -0.500191
C -6.463521 0.434406 -0.764800
C -5.165583 0.840257 -1.029436
H 0.230101 2.771529 0.523300
H -0.163699 5.190850 0.343560
H -2.471873 6.114570 0.518398
H -4.392325 4.574677 0.887050
H -4.005083 2.130081 1.085266
H 0.739659 -0.136565 -2.078279
H -0.577508 2.490520 -2.133635
H -0.997077 1.341255 -3.422326
H -3.586206 -2.187830 -0.815293
H -5.920858 -2.896630 -0.309855
H -7.749403 -1.223541 -0.286743
H -7.271102 1.163468 -0.763177
H -4.935526 1.883502 -1.241545
O 2.393306 -0.827074 0.933697
C 3.238742 -1.678012 1.724129
C 3.824311 -2.713996 0.800905
C 1.408584 -0.118277 1.464944
O 0.712077 0.540781 0.703667
C 1.193035 -0.152614 2.945220
H 4.017458 -1.057003 2.191743
H 2.652031 -2.152083 2.519795
H 4.459941 -3.403470 1.368852
H 3.024641 -3.288879 0.319571
H 4.431311 -2.244659 0.017981
H 2.134737 -0.097579 3.502447
H 0.534700 0.671118 3.229661

H 0.683657 -1.088559 3.214761
C 3.366326 -0.162512 -2.296327
C 4.637867 -0.708705 -2.142897
C 5.541715 -0.138375 -1.257256
C 5.145394 0.986272 -0.541128
C 3.899391 1.567923 -0.704693
H 2.669393 -0.619852 -2.996077
H 4.923292 -1.588227 -2.716254
N 6.086593 1.579157 0.419302
H 3.623956 2.467884 -0.161299
H 6.538936 -0.543014 -1.108291
O 5.705823 2.543710 1.061542
O 7.187911 1.061855 0.521223

I_1

54
Energy: -4244.40798533
Cu -0.008855 -0.052862 -0.006121
Cl 0.009594 -0.172486 2.121668
C 1.853834 -0.097373 0.305017
O -2.028235 0.277989 -0.251958
S -1.957391 1.712061 -0.724276
O -2.628902 1.989529 -1.979123
C -2.886364 2.611408 0.576378
O -0.551244 2.154753 -0.581791
F -2.869050 3.905503 0.303761
F -4.137015 2.179779 0.596410
F -2.328284 2.400290 1.753946
C 2.576761 -1.273017 0.292234
C 3.964041 -1.156806 0.192431
C 4.568280 0.097217 0.141757
C 3.796479 1.252897 0.194634
C 2.406896 1.169096 0.303400
H 2.091095 -2.247914 0.335992
H 4.568409 -2.062169 0.164092
H 5.652327 0.172669 0.078395
H 4.267404 2.234296 0.171551
H 1.790680 2.063551 0.378546
C -0.660177 -3.276778 0.503272
O 0.368441 -3.713937 0.003268
C -0.988220 -3.478910 1.943010
N -1.564888 -2.563588 -0.238200
C -1.225397 -2.230751 -1.604535
C -0.205115 -1.168639 -1.747945
C 0.554604 -0.366298 -2.290057
C 1.514910 0.505601 -2.866706
C 1.176629 1.830465 -3.185169
C 2.143123 2.668815 -3.719975
C 3.434782 2.195742 -3.943580
C 3.770277 0.878159 -3.637990
C 2.815293 0.030376 -3.099338
H -2.213020 -1.934565 0.225090
H -0.846754 -3.133758 -2.097682
H -2.138116 -1.905375 -2.116688
H 0.167264 2.185059 -2.987491
H 1.888788 3.698339 -3.962597
H 4.188096 2.861337 -4.361913
H 4.780712 0.514951 -3.814541
H 3.059928 -0.997185 -2.835547
C 0.067650 -3.825409 2.782671
C -0.180002 -3.973930 4.135709
C -1.444527 -3.800320 4.684985
C -2.495112 -3.475811 3.837737
C -2.269095 -3.315587 2.474339
H 1.067203 -3.963787 2.379602
N 0.935971 -4.330057 5.026012
H -1.583447 -3.925802 5.754801
H -3.496505 -3.347028 4.241682
H -3.108999 -3.080128 1.822292
O 0.688371 -4.449093 6.214649
O 2.033030 -4.486063 4.518409

TS_{rc}^{A}

54
Energy: -4244.38118336
Cu -0.647538 -0.635664 -0.388911
Cl 0.463054 -1.656442 -1.904708
C -1.857129 -2.072817 -0.402153
O 0.500039 0.990429 -0.466669
S 0.495130 2.422139 0.001123

O 1.702939 2.759703 0.763892
C 0.649151 3.311110 -1.593716
O -0.774904 2.864515 0.573130
F 0.650929 4.615266 -1.371512
F 1.764373 2.966410 -2.208583
F -0.390914 3.001599 -2.359960
C -1.740609 -3.104362 0.510840
C -2.730157 -4.088130 0.516468
C -3.792298 -4.025268 -0.379959
C -3.869929 -2.982749 -1.297679
C -2.890372 -1.991064 -1.319363
H -0.909708 -3.144487 1.212961
H -2.663246 -4.906594 1.231924
H -4.559140 -4.797750 -0.368726
H -4.690949 -2.937099 -2.011437
H -2.947759 -1.177245 -2.042053
C 1.395381 -0.187348 2.019598
O 0.520955 -1.049032 1.766077
C 2.762772 -0.287119 1.504074
N 1.010359 0.849827 2.802172
C -0.397456 0.744273 3.179496
C -1.112202 0.098322 2.066832
C -1.800636 0.087860 0.998282
C -3.146499 0.535948 0.655340
C -3.374651 1.440874 -0.385592
C -4.677599 1.770030 -0.735463
C -5.755838 1.191691 -0.070447
C -5.528209 0.282249 0.957408
C -4.228899 -0.045882 1.323327
H 1.388810 1.771586 2.566711
H -0.484303 0.177250 4.115617
H -0.814453 1.749725 3.317964
H -2.530226 1.903794 -0.893030
H -4.850375 2.483965 -1.538559
H -6.774153 1.448104 -0.356924
H -6.365887 -0.178752 1.477681
H -4.038943 -0.783017 2.102966
C 3.778141 0.575719 1.930589
C 5.053830 0.473700 1.393572
C 5.327943 -0.490933 0.431364
C 4.310460 -1.348123 0.033019
C 3.028584 -1.268127 0.546572
H 3.575172 1.329507 2.688231
H 5.838549 1.149969 1.723005
H 6.313084 -0.595286 -0.014473
N 4.610634 -2.384930 -0.969195
H 2.250074 -1.938244 0.187302
O 3.738378 -3.197119 -1.211903
O 5.719964 -2.360841 -1.477460

TS_{ar}^{B}

54
Energy: -4244.39265705
Cu 0.674655 -0.496114 0.276370
Cl -0.040568 -1.092036 2.226851
C 2.094558 -1.824208 0.295924
O -0.516936 1.098077 0.257953
S -0.183615 2.535373 -0.051902
O -0.719519 2.953967 -1.356446
C -1.233517 3.392891 1.184000
O 1.193143 2.901540 0.255820
F -1.064022 4.699988 1.058673
F -2.507603 3.096512 0.966113
F -0.893855 3.017814 2.401126
C 1.924394 -2.908207 -0.556026
C 2.572290 -4.103242 -0.249084
C 3.388107 -4.187817 0.873853
C 3.559014 -3.080505 1.704231
C 2.909361 -1.886746 1.422108
H 1.286156 -2.828562 -1.433446
H 2.439503 -4.965000 -0.900449
H 3.898886 -5.120965 1.103808
H 4.189179 -3.148283 2.588875
H 3.032654 -1.019515 2.067647
C -1.464725 -0.576443 -1.612233
O -0.807174 -1.552675 -1.227492
C -2.869363 -0.373137 -1.182004
N -0.897619 0.334872 -2.444662
C 0.522245 0.222531 -2.667107
C 1.372933 0.041477 -1.463024
C 2.467177 -0.152013 -0.867038

C 3.840069 0.225228 -0.611413
C 4.106818 1.552385 -0.254060
C 5.424683 1.949926 -0.059158
C 6.462807 1.039207 -0.225485
C 6.192088 -0.282200 -0.581636
C 4.882532 -0.697197 -0.764056
H -1.240416 1.293745 -2.411784
H 0.723813 -0.639135 -3.320202
H 0.854931 1.128484 -3.191166
H 3.276432 2.246121 -0.124324
H 5.638023 2.978224 0.225254
H 7.492814 1.357268 -0.073500
H 7.007321 -0.991095 -0.711607
H 4.654819 -1.729039 -1.029181
C -3.520342 0.862778 -1.249884
C -4.823504 0.996855 -0.785417
C -5.496348 -0.099729 -0.262572
C -4.831619 -1.317533 -0.200139
C -3.525959 -1.472983 -0.633345
H -3.004358 1.749429 -1.617077
H -5.313777 1.966718 -0.823447
H -6.516293 -0.029772 0.104180
N -5.539920 -2.483861 0.350070
H -3.020013 -2.430194 -0.543413
O -4.950426 -3.550193 0.351710
O -6.674997 -2.306504 0.760878

I_2

54
Energy: -4244.44430993
Cu -0.002299 0.470293 0.567092
Cl -1.211044 1.937736 1.668936
C 2.362687 -0.764336 1.698700
O 1.361936 1.649283 0.082569
S 1.329430 1.922253 -1.426925
O 0.007349 1.610061 -1.975510
C 1.480264 3.752506 -1.428821
O 2.497831 1.404181 -2.122231
F 1.503247 4.163455 -2.687887
F 0.452700 4.289124 -0.806739
F 2.604536 4.103635 -0.831102
C 1.337737 -0.550541 2.630704
C 1.594255 0.126826 3.819213
C 2.878452 0.577219 4.096468
C 3.910437 0.335844 3.190050
C 3.657605 -0.331390 1.999897
H 0.350348 -0.985279 2.466117
H 0.782174 0.301796 4.521936
H 3.080765 1.112455 5.022175
H 4.918281 0.684521 3.408480
H 4.455935 -0.492023 1.276339
C -1.848760 -1.189572 -0.518340
O -1.428617 -0.743789 0.580392
C -3.301460 -1.428972 -0.644693
N -1.061207 -1.461198 -1.558347
C 0.400797 -1.363122 -1.571730
C 0.965760 -0.934923 -0.271150
C 2.056100 -1.352703 0.377895
C 2.997052 -2.335860 -0.207678
C 3.493631 -2.155743 -1.503060
C 4.383157 -3.078888 -2.044272
C 4.782408 -4.183941 -1.299367
C 4.304208 -4.358778 -0.002238
C 3.423629 -3.435382 0.545335
H -1.510144 -1.610654 -2.453740
H 0.804698 -2.361008 -1.809241
H 0.674397 -0.678074 -2.386211
H 3.215676 -1.259931 -2.060734
H 4.776149 -2.924710 -3.047732
H 5.478270 -4.905256 -1.724523
H 4.623678 -5.217186 0.586242
H 3.053225 -3.560302 1.562794
C -3.824980 -2.406290 -1.496269
C -5.198659 -2.592674 -1.588894
C -6.057243 -1.803929 -0.833424
C -5.515266 -0.845868 0.013576
C -4.151412 -0.640236 0.133579
H -3.157489 -3.054043 -2.063840
H -5.602166 -3.360763 -2.244128
H -7.136312 -1.919640 -0.880191
N -6.434806 -0.022243 0.822392

H -3.751547 0.119883 0.804720
O -5.937596 0.792039 1.575496
O -7.629283 -0.222173 0.677420

TS_{rc}^{B}

54
Energy: -4244.41517401
Cu -0.440210 -1.288440 0.280847
Cl 0.717715 -2.969300 -0.479692
C -0.775937 1.973091 1.289469
O -2.120091 -1.096826 1.151368
S -3.288767 -1.056509 0.173365
O -2.826475 -1.089861 -1.216048
C -4.034041 -2.705895 0.464781
O -4.294933 -0.076271 0.547846
F -5.075244 -2.862565 -0.337378
F -3.137533 -3.645947 0.206965
F -4.429012 -2.810050 1.722475
C -0.350629 1.105924 2.307583
C -0.274844 1.534756 3.620988
C -0.625174 2.841885 3.951569
C -1.054224 3.713191 2.955911
C -1.130778 3.284737 1.637924
H -0.098849 0.077472 2.066759
H 0.048574 0.840417 4.393616
H -0.570159 3.176661 4.985790
H -1.334373 4.734743 3.205755
H -1.463660 3.975111 0.865378
C 2.091948 -0.234081 -0.905315
O 1.484603 -0.046045 0.172683
C 3.535169 -0.539114 -0.915766
N 1.436094 -0.107445 -2.064128
C -0.016183 0.001139 -2.017155
C -0.384015 0.406183 -0.629315
C -0.820536 1.563823 -0.115259
C -1.489118 2.461729 -1.107219
C -2.882277 2.506348 -1.186228
C -3.488704 3.367118 -2.095534
C -2.715734 4.177493 -2.922036
C -1.325974 4.130588 -2.844117
C -0.711524 3.270735 -1.940367
H 1.895254 -0.327982 -2.938455
H -0.356446 0.785688 -2.706507
H -0.480196 -0.950111 -2.313294
H -3.482198 1.860150 -0.542977
H -4.574549 3.397286 -2.161217
H -3.197256 4.847624 -3.632103
H -0.719044 4.768720 -3.484224
H 0.376538 3.229825 -1.863759
C 4.080519 -1.424741 -1.849244
C 5.439383 -1.716555 -1.824660
C 6.261628 -1.124377 -0.874369
C 5.694054 -0.259070 0.052088
C 4.341706 0.036334 0.063153
H 3.431371 -1.934361 -2.560739
H 5.857600 -2.418920 -2.541373
H 7.327754 -1.327342 -0.828610
N 6.561751 0.364287 1.066465
H 3.927981 0.702595 0.815404
O 6.036681 1.128045 1.856899
O 7.745187 0.074851 1.041092

Reaction 30

reactant

33
Energy: -951.296604973
C 0.065954 -0.857759 -0.063175
O 0.117689 -1.364403 1.040971
C 1.316178 -0.499793 -0.823646
N -1.088722 -0.666927 -0.756246
H -1.077533 -0.038080 -1.550415
C -2.355533 -0.823988 -0.050606
C -3.478986 -0.653371 -0.956268
H -2.362606 -1.821843 0.409068
H -2.410849 -0.104647 0.783792
C -4.406121 -0.497831 -1.719885
C -5.499821 -0.322265 -2.619755
C -5.724174 -1.246889 -3.649519
C -6.786402 -1.073806 -4.526090

C -7.638150 0.018925 -4.386367
C -7.423532 0.941126 -3.365323
C -6.361484 0.775619 -2.486833
H -5.054582 -2.099516 -3.748449
H -6.952578 -1.796949 -5.322731
H -8.471425 0.151808 -5.074245
H -8.088225 1.795967 -3.253414
H -6.185031 1.491840 -1.686257
C 1.760289 -1.358240 -1.826593
C 2.978831 -1.139983 -2.462714
C 3.778773 -0.061351 -2.097502
C 3.353800 0.807934 -1.101551
C 2.129562 0.581920 -0.487993
H 1.142280 -2.213857 -2.095196
H 3.307663 -1.821066 -3.245275
H 4.732275 0.109023 -2.592316
H 3.945156 1.666477 -0.795763
N 1.666334 1.565596 0.500136
O 2.499589 2.303766 0.995562
O 0.468564 1.598829 0.736203

I_c

68
Energy: -4551.91976137
Cu -1.557333 0.308649 0.612045
Cl -2.760041 -0.287100 2.322032
C -1.838146 2.064534 1.291318
O -1.494836 -1.568806 -0.045981
S -0.648180 -2.277990 -1.065446
O 0.648122 -1.645166 -1.315686
C -0.239498 -3.801729 -0.124101
O -1.406654 -2.730247 -2.224983
F 0.686278 -4.498790 -0.767805
F 0.241760 -3.479652 1.080700
F -1.315518 -4.548338 0.040875
C -0.686487 2.746906 1.638276
C -0.810313 4.104870 1.952981
C -2.050530 4.728532 1.936444
C -3.190494 3.999940 1.606246
C -3.093522 2.646439 1.289732
C 2.342609 1.551621 -0.653091
O 2.258575 2.441359 0.177110
C 3.611921 0.772242 -0.851550
N 1.338850 1.200854 -1.505561
C 0.110439 1.942955 -1.525575
C -1.125739 1.197046 -1.211978
C -2.287966 0.840845 -1.445234
C -3.651548 0.510554 -1.650217
C -4.053306 -0.807967 -1.926645
C -5.398922 -1.074643 -2.128049
C -6.339562 -0.048612 -2.064790
C -5.943067 1.261797 -1.802591
C -4.603806 1.546340 -1.591324
H 0.299766 2.287586 1.618620
H 0.090877 4.664674 2.198247
H -2.133226 5.784126 2.188733
H -4.170355 4.475502 1.611873
H -3.985800 2.061108 1.073182
H 1.373853 0.304270 -1.981434
H 0.226231 2.765790 -0.805255
H -0.041182 2.394940 -2.517762
H -3.305938 -1.598339 -1.981537
H -5.716820 -2.093568 -2.337736
H -7.393185 -0.271153 -2.224928
H -6.681049 2.060249 -1.763073
H -4.270623 2.563495 -1.390195
O 2.045150 -1.440871 1.809847
C 2.569945 -2.453223 2.680039
C 3.533953 -3.271699 1.863041
C 0.950844 -0.754059 2.114780
O 0.515428 0.010378 1.267961
C 0.317257 -0.945807 3.455746
H 3.068363 -1.970567 3.532960
H 1.749628 -3.075682 3.060073
H 3.949960 -4.085859 2.467307
H 3.016146 -3.705170 0.997670
H 4.359241 -2.647288 1.500988
H 1.053639 -1.092381 4.252537
H -0.316136 -0.083804 3.678625
H -0.340236 -1.826056 3.417943
C 3.620979 -0.618737 -0.777600

```
 C 4.817954 -1.323642 -0.891984
 C 6.025483 -0.652897 -1.045179
 C 6.037207 0.735235 -1.109929
 C 4.835597 1.422057 -1.034265
 H 2.683573 -1.150110 -0.616297
 H 4.800150 -2.412155 -0.861187
 H 6.957364 1.296995 -1.246346
 N 4.890889 2.873387 -1.264979
 H 6.958518 -1.206113 -1.129614
 O 3.970895 3.374128 -1.887503
 O 5.879936 3.464872 -0.865424
```

I_1

```
  54
Energy: -4244.39638315
 Cu 0.274213 -0.019425 0.028497
 Cl 0.365037 -0.074355 2.154518
 C 2.145102 -0.103978 0.222212
 O -1.754635 0.296112 -0.150857
 S -1.714204 1.723326 -0.646887
 O -2.431121 1.971290 -1.884944
 C -2.619122 2.632527 0.663662
 O -0.312985 2.187870 -0.559092
 F -2.615159 3.923720 0.375082
 F -3.867119 2.195476 0.716860
 F -2.034392 2.439734 1.831074
 C 2.831181 -1.296239 0.101972
 C 4.214226 -1.214625 -0.064780
 C 4.853911 0.023193 -0.081100
 C 4.122079 1.195181 0.073797
 C 2.737431 1.143763 0.250234
 H 2.326885 -2.260935 0.137246
 H 4.786945 -2.134391 -0.171429
 H 5.935198 0.071009 -0.197518
 H 4.621046 2.162858 0.077677
 H 2.153972 2.049762 0.405602
 C -0.566379 -3.526401 0.678387
 O 0.217687 -4.321871 0.193511
 C -1.060896 -3.629783 2.091583
 N -1.143742 -2.512965 -0.038464
 C -0.813955 -2.316606 -1.430931
 C 0.049856 -1.146178 -1.690346
 C 0.666003 -0.284174 -2.317107
 C 1.437165 0.695241 -2.992671
 C 0.830737 1.872646 -3.460327
 C 1.599266 2.813439 -4.128504
 C 2.961403 2.593097 -4.323997
 C 3.567270 1.429057 -3.854259
 C 2.809694 0.476163 -3.191981
 H -1.724452 -1.816952 0.419260
 H -0.296013 -3.226292 -1.761823
 H -1.732852 -2.200203 -2.021075
 H -0.230670 2.035170 -3.280843
 H 1.135831 3.727752 -4.492571
 H 3.558860 3.339240 -4.845433
 H 4.632694 1.266473 -4.004187
 H 3.264394 -0.434679 -2.804534
 C -0.223694 -3.898872 3.178144
 C -0.720943 -4.137125 4.449704
 C -2.092750 -4.092988 4.666984
 C -2.948757 -3.824887 3.606693
 C -2.434234 -3.604288 2.332348
 N 1.240900 -3.873162 3.043277
 H -2.487531 -4.266520 5.665460
 H -4.024735 -3.795028 3.765143
 H -3.113580 -3.438053 1.497749
 H -0.020871 -4.340313 5.255164
 O 1.719981 -3.114689 2.220971
 O 1.877853 -4.588360 3.798388
```

TS_{rc}^{A}

```
  54
Energy: -4244.37505555
 Cu -1.049508 -0.115990 -0.932227
 Cl -0.622091 -0.218727 -3.025492
 C -2.306626 -1.480336 -1.227086
 O -0.003617 1.526887 -0.675279
 S 0.496148 2.316638 0.511529
 O 1.566542 1.627714 1.239973
 C 1.343379 3.682548 -0.372888
```

O -0.557020 2.939770 1.299973
F 1.903588 4.490210 0.514542
F 2.289725 3.192389 -1.166304
F 0.476449 4.364659 -1.099888
C -1.978551 -2.807114 -1.018086
C -2.994675 -3.757501 -1.121940
C -4.292825 -3.370143 -1.440122
C -4.584830 -2.029211 -1.667194
C -3.582258 -1.065223 -1.568702
H -0.960636 -3.102107 -0.769100
H -2.761180 -4.807690 -0.951744
H -5.078849 -4.118923 -1.521462
H -5.595679 -1.722536 -1.931555
H -3.808492 -0.015098 -1.753501
C 1.750080 -1.199156 0.749847
O 0.682023 -1.472327 0.169147
C 3.008287 -0.981415 0.002089
N 1.699477 -1.012372 2.080112
C 0.351046 -1.121470 2.610512
C -0.625560 -0.775611 1.562042
C -1.590291 -0.234848 0.947320
C -2.920376 0.252309 1.306302
C -3.348186 1.538839 0.967262
C -4.651829 1.924658 1.253702
C -5.537650 1.034076 1.853784
C -5.114798 -0.251647 2.177583
C -3.810148 -0.644109 1.908294
H 2.345148 -0.348158 2.493380
H 0.184308 -2.144484 2.977339
H 0.226552 -0.418601 3.444058
H -2.647546 2.236859 0.512824
H -4.977558 2.931617 0.999729
H -6.560318 1.341317 2.065246
H -5.804177 -0.956146 2.639568
H -3.479384 -1.660021 2.123512
C 2.974112 -0.039596 -1.024529
C 4.140631 0.289550 -1.707583
C 5.347280 -0.317773 -1.377038
C 5.391987 -1.279933 -0.374891
C 4.222959 -1.609646 0.293552
H 2.031344 0.448831 -1.267640
H 4.098233 1.031668 -2.501435
H 6.257469 -0.058213 -1.912946
H 6.309979 -1.800398 -0.117117
N 4.277595 -2.726397 1.246274
O 3.243442 -3.348352 1.435789
O 5.353552 -2.976128 1.760536

TS_{ar}^{B}

54
Energy: -4244.38766009
Cu 0.763548 -0.306524 0.675668
Cl 0.613775 -0.102143 2.831062
C 2.333236 -1.443507 0.800202
O -0.637949 1.067095 0.404364
S -0.593000 2.306751 -0.455978
O -1.403898 2.163849 -1.669807
C -1.525207 3.460403 0.622532
O 0.744438 2.869428 -0.598129
F -1.640363 4.630630 0.015792
F -2.734893 2.967745 0.855947
F -0.888101 3.617645 1.767732
C 2.136143 -2.777344 0.467511
C 3.005119 -3.729488 0.997724
C 4.056766 -3.336523 1.818277
C 4.245836 -1.988830 2.122559
C 3.381146 -1.028538 1.615465
H 1.312485 -3.073697 -0.178715
H 2.856723 -4.780224 0.755918
H 4.738325 -4.083366 2.221149
H 5.062780 -1.680670 2.772208
H 3.517868 0.024905 1.851442
C -1.739789 -1.277521 -0.470844
O -0.868665 -1.876163 0.164415
C -3.113776 -1.067103 0.074588
N -1.477348 -0.682922 -1.660072
C -0.139364 -0.782658 -2.177626
C 0.971007 -0.429787 -1.257374
C 2.183798 -0.307492 -0.935683
C 3.503223 0.227714 -1.188880
C 3.629790 1.606776 -1.395013

C 4.879702 2.134435 -1.698422
C 5.986678 1.298510 -1.801626
C 5.855859 -0.074790 -1.594095
C 4.619253 -0.613694 -1.276226
H -1.974023 0.182854 -1.874915
H 0.040640 -1.819170 -2.501490
H -0.067847 -0.135574 -3.061785
H 2.749440 2.242677 -1.302304
H 4.985759 3.206038 -1.854101
H 6.962180 1.717667 -2.042292
H 6.724335 -0.725161 -1.676524
H 4.503562 -1.682296 -1.098300
C -3.239563 -0.318913 1.243840
C -4.500654 -0.023849 1.752534
C -5.648017 -0.476527 1.108381
C -5.539829 -1.246681 -0.042016
C -4.277013 -1.539603 -0.537200
H -2.339955 0.046275 1.736976
H -4.582349 0.569272 2.660902
H -6.631820 -0.243313 1.509533
H -6.411552 -1.642010 -0.555520
N -4.190556 -2.444658 -1.690645
O -3.138411 -3.043584 -1.855599
O -5.180029 -2.561585 -2.392845

I_2

54
Energy: -4244.43756091
Cu 0.144905 0.790949 0.674558
Cl -0.093682 2.348498 2.206816
C 1.382677 -1.696788 1.415662
O 1.952536 1.144844 0.339059
S 2.126176 1.740132 -1.062678
O 0.860841 2.300566 -1.543801
C 3.218117 3.165380 -0.678408
O 2.875166 0.872533 -1.959231
F 3.499451 3.789846 -1.812901
F 2.607560 3.994184 0.141236
F 4.337473 2.729677 -0.128190
C 0.496163 -1.239843 2.400014
C 0.928572 -1.073896 3.713152
C 2.239344 -1.378101 4.054845
C 3.117188 -1.864078 3.085469
C 2.693482 -2.025804 1.774607
H -0.556339 -1.088643 2.153259
H 0.234295 -0.699936 4.462673
H 2.581085 -1.242446 5.079235
H 4.143990 -2.105945 3.354321
H 3.383829 -2.377737 1.008890
C -2.290411 0.372475 -0.566300
O -1.707457 0.584191 0.520481
C -3.749385 0.678056 -0.622221
N -1.718137 -0.015129 -1.698260
C -0.340926 -0.512300 -1.822330
C 0.280639 -0.706888 -0.494599
C 0.952249 -1.748352 0.002825
C 1.331597 -2.917126 -0.823553
C 1.966439 -2.732832 -2.056708
C 2.332598 -3.834191 -2.824169
C 2.070199 -5.121948 -2.367741
C 1.452928 -5.310780 -1.132701
C 1.093024 -4.215256 -0.359282
H -2.321901 -0.111112 -2.505776
H -0.394479 -1.489304 -2.327071
H 0.223904 0.182399 -2.458901
H 2.214174 -1.721302 -2.383207
H 2.838395 -3.683642 -3.776375
H 2.357705 -5.982663 -2.969352
H 1.254462 -6.317874 -0.769849
H 0.615414 -4.354785 0.610354
C -4.150047 1.881579 -1.194550
C -5.493967 2.246600 -1.191268
C -6.446064 1.416592 -0.609133
C -6.060500 0.213307 -0.030957
C -4.720670 -0.142196 -0.047012
H -3.396961 2.543422 -1.620160
H -5.793908 3.191447 -1.638956
H -7.494665 1.704461 -0.603659
H -6.777551 -0.460572 0.429088
N -4.337419 -1.441059 0.524102
O -3.205387 -1.840502 0.289090

O -5.172057 -2.041826 1.172429

TS_{rc}^{B}

54
Energy: -4244.41444892
Cu -0.606443 -1.415329 0.316120
Cl 0.276985 -3.314258 -0.318963
C -0.326967 1.774124 1.565105
O -2.349840 -0.931352 0.927957
S -3.302860 -0.584501 -0.206474
O -2.626944 -0.616073 -1.506208
C -4.388880 -2.061134 -0.204310
O -4.155548 0.551427 0.104328
F -5.280620 -1.953342 -1.177225
F -3.658166 -3.148358 -0.401162
F -5.016580 -2.160964 0.955832
C -0.297226 0.763592 2.538162
C -0.289928 1.074866 3.886322
C -0.315170 2.405843 4.296653
C -0.351705 3.420232 3.345347
C -0.360983 3.109783 1.992713
H -0.305952 -0.277820 2.229505
H -0.278405 0.273012 4.621640
H -0.315561 2.650098 5.357447
H -0.376126 4.462642 3.657399
H -0.385565 3.908785 1.254147
C 2.230343 -0.719832 -0.474003
O 1.476878 -0.586499 0.517095
C 3.619946 -1.214411 -0.295294
N 1.764726 -0.494246 -1.696287
C 0.362065 -0.112094 -1.800651
C -0.060381 0.277902 -0.421816
C -0.296138 1.476438 0.133131
C -0.630347 2.560105 -0.838697
C -1.960317 2.916656 -1.068118
C -2.248028 3.945734 -1.959032
C -1.219669 4.615921 -2.615335
C 0.106540 4.257579 -2.385717
C 0.405013 3.226217 -1.501635
H 2.318764 -0.711612 -2.514482
H 0.250901 0.759333 -2.459364
H -0.234343 -0.939043 -2.209092
H -2.761537 2.376145 -0.560797
H -3.284962 4.218945 -2.144836
H -1.451872 5.420195 -3.311497
H 0.912570 4.785534 -2.892701
H 1.440424 2.941410 -1.304053
C 3.799735 -2.591478 -0.199245
C 5.078817 -3.111171 -0.019278
C 6.179895 -2.264798 0.071517
C 6.010991 -0.888491 -0.011701
C 4.733178 -0.378355 -0.189380
H 2.919640 -3.235444 -0.255591
H 5.211365 -4.188280 0.055363
H 7.176617 -2.675494 0.215259
H 6.846904 -0.199320 0.065232
N 4.565238 1.079435 -0.251781
O 5.566939 1.768497 -0.242969
O 3.419120 1.506262 -0.309463

Reaction 31

reactant

34
Energy: -786.179535542
C 0.001779 -0.084000 0.014054
O -0.007184 -0.152510 1.235058
C -1.250204 0.009645 -0.792186
N 1.174685 -0.087350 -0.688205
H 1.177508 -0.283126 -1.679886
C 2.427445 -0.256170 0.031440
C 3.560181 -0.180318 -0.877160
H 2.488125 0.523130 0.804765
H 2.430260 -1.214270 0.577067
C 4.493382 -0.126101 -1.647512
C 5.595593 -0.057672 -2.551683
C 5.959118 1.164072 -3.135713
C 7.029801 1.227375 -4.016888
C 7.751664 0.077259 -4.325354
C 7.398422 -1.140041 -3.748866

C 6.327497 -1.210811 -2.868482
H 5.390323 2.058164 -2.886319
H 7.304346 2.180710 -4.465225
H 8.591634 0.129857 -5.015942
H 7.961467 -2.040724 -3.987509
H 6.043603 -2.158069 -2.413256
C -1.292284 0.483401 -2.104518
C -2.499345 0.547429 -2.789416
C -3.690266 0.138674 -2.186916
C -3.641512 -0.318604 -0.866655
C -2.441393 -0.377890 -0.175705
H -0.387451 0.847975 -2.590818
H -2.521816 0.931551 -3.809743
C -4.988222 0.176310 -2.935989
H -4.565490 -0.627462 -0.376218
H -2.397603 -0.718628 0.856973
H -5.821689 0.463557 -2.282578
H -4.952642 0.884774 -3.772496
H -5.233876 -0.810135 -3.354476

I_c

69
Energy: -4386.80779891
Cu -1.331340 0.386464 0.557055
Cl -2.044525 0.105957 2.587021
C -2.011367 2.143558 0.820488
O -0.757201 -1.529060 0.406994
S -0.071925 -2.306035 -0.683782
O 1.077217 -1.616860 -1.277081
C 0.663897 -3.651288 0.326784
O -0.992161 -2.978282 -1.593601
F 1.474923 -4.384386 -0.422321
F 1.373469 -3.138009 1.335904
F -0.283876 -4.421735 0.829623
C -1.047214 3.124558 0.705751
C -1.484945 4.449175 0.608051
C -2.837873 4.755595 0.666042
C -3.775678 3.738126 0.825916
C -3.364824 2.409503 0.915954
C 1.715933 2.190428 -1.513572
O 1.312259 3.329235 -1.302954
C 3.121446 1.794745 -1.230927
N 0.926740 1.206024 -2.045162
C -0.460631 1.479170 -2.298276
C -1.438326 0.713523 -1.495441
C -2.502332 0.102316 -1.336482
C -3.770668 -0.493624 -1.113961
C -3.897468 -1.864351 -0.831104
C -5.160143 -2.397978 -0.623006
C -6.290815 -1.587922 -0.704394
C -6.170048 -0.230556 -0.997192
C -4.915139 0.322306 -1.196468
H 0.013643 2.901541 0.639923
H -0.738473 5.229738 0.468621
H -3.165708 5.791324 0.597028
H -4.837020 3.971782 0.899970
H -4.087783 1.611698 1.077173
H 1.185743 0.231754 -1.881427
H -0.611938 2.552159 -2.105358
H -0.710029 1.289899 -3.353787
H -3.003818 -2.484383 -0.793823
H -5.263965 -3.457471 -0.399213
H -7.277337 -2.018770 -0.541582
H -7.056904 0.395895 -1.067627
H -4.797409 1.380514 -1.425142
O 2.650323 -0.395423 0.987803
C 3.620057 -1.207037 1.664871
C 4.287574 -2.078545 0.633333
C 1.585388 0.103748 1.593628
O 0.815662 0.780778 0.924406
C 1.362216 -0.181766 3.046182
H 4.339324 -0.535409 2.158308
H 3.131216 -1.820178 2.431313
H 5.031185 -2.724615 1.115613
H 3.546099 -2.708765 0.130524
H 4.786867 -1.467349 -0.126781
H 2.275819 -0.057850 3.638536
H 0.573906 0.474629 3.419935
H 1.014173 -1.218078 3.158585
C 3.779492 0.781797 -1.927954
C 5.103178 0.483135 -1.633117

C 5.784420 1.152459 -0.613496
C 5.116248 2.168817 0.074957
C 3.806960 2.499606 -0.241854
H 3.267442 0.238485 -2.721117
H 5.619916 -0.295269 -2.197289
H 5.640391 2.713466 0.861548
H 3.287230 3.306543 0.272254
C 7.184337 0.760388 -0.246942
H 7.765902 0.462203 -1.128415
H 7.718414 1.577919 0.252616
H 7.186198 -0.098227 0.442386

I_1

55
Energy: -4079.28213696
Cu 0.021630 -0.003049 -0.010752
Cl 0.073509 -0.068420 2.116183
C 1.888405 -0.023432 0.283280
O -1.997703 0.330450 -0.250985
S -1.932027 1.757006 -0.742330
O -2.612551 2.018389 -1.996372
C -2.855729 2.672370 0.550491
O -0.527475 2.208412 -0.614917
F -2.837319 3.964202 0.263617
F -4.107683 2.244623 0.579642
F -2.295266 2.475005 1.729403
C 2.619291 -1.194492 0.281421
C 4.003929 -1.071541 0.156345
C 4.600292 0.184500 0.071308
C 3.821979 1.336278 0.114155
C 2.435024 1.245757 0.246539
H 2.136804 -2.170002 0.349896
H 4.613760 -1.973627 0.132524
H 5.682626 0.265117 -0.013683
H 4.286066 2.319899 0.062036
H 1.813691 2.137438 0.311277
C -0.606583 -3.206409 0.579370
O 0.450576 -3.586456 0.086029
C -0.961191 -3.437643 2.000650
N -1.530099 -2.531639 -0.181466
C -1.200740 -2.216270 -1.551427
C -0.188263 -1.151110 -1.727363
C 0.561023 -0.350121 -2.285052
C 1.513739 0.512657 -2.890092
C 1.173301 1.832986 -3.223349
C 2.132172 2.660961 -3.787706
C 3.418757 2.181920 -4.026584
C 3.756041 0.868232 -3.707015
C 2.808492 0.031170 -3.138830
H -2.209381 -1.930432 0.272746
H -0.817861 -3.122735 -2.035881
H -2.118230 -1.904292 -2.063786
H 0.168314 2.192946 -3.013690
H 1.875373 3.687196 -4.041696
H 4.166169 2.839225 -4.468124
H 4.762281 0.499406 -3.895957
H 3.054866 -0.993373 -2.865257
C 0.079742 -3.708166 2.891197
C -0.184779 -3.907686 4.235902
C -1.492805 -3.852580 4.727258
C -2.530059 -3.602946 3.827210
C -2.271392 -3.392902 2.479279
H 1.096802 -3.746962 2.505002
H 0.635116 -4.108115 4.926330
C -1.767148 -4.038565 6.188774
H -3.557577 -3.576324 4.190506
H -3.104107 -3.227506 1.796333
H -2.803605 -4.347515 6.370491
H -1.103896 -4.793011 6.630408
H -1.603599 -3.103093 6.742312

TS_{rc}^{A}

55
Energy: -4079.26001777
Cu -0.698039 -0.602730 -0.595452
Cl -0.014040 -1.420858 -2.456081
C -2.108856 -1.846073 -0.665266
O 0.672555 0.827815 -0.571340
S 1.017718 2.071455 0.206058
O 2.316958 1.975808 0.877318

C 1.252496 3.265784 -1.163219
O -0.085274 2.611192 0.999388
F 1.563764 4.451433 -0.662869
F 2.215369 2.864126 -1.972314
F 0.118392 3.368970 -1.847770
C -2.023723 -3.029238 0.045778
C -3.129406 -3.880463 0.036731
C -4.274773 -3.541950 -0.676819
C -4.321857 -2.351608 -1.395456
C -3.226659 -1.489341 -1.399565
H -1.127720 -3.282526 0.609659
H -3.087824 -4.813385 0.597465
H -5.132307 -4.212644 -0.678748
H -5.210509 -2.087970 -1.966949
H -3.260625 -0.559089 -1.966565
C 1.614909 -1.022881 1.616342
O 0.578932 -1.617318 1.234982
C 2.924411 -1.232389 1.012531
N 1.464912 -0.147251 2.648048
C 0.085607 -0.078569 3.113234
C -0.813961 -0.283474 1.969008
C -1.588176 0.029937 1.017697
C -2.851325 0.745123 0.875362
C -2.967353 1.861586 0.042148
C -4.207816 2.462249 -0.130733
C -5.335829 1.950718 0.505355
C -5.221096 0.832287 1.325183
C -3.984120 0.228749 1.512480
H 1.996845 0.722611 2.567931
H -0.076578 -0.830142 3.897672
H -0.111389 0.920493 3.523093
H -2.080889 2.268420 -0.440525
H -4.291855 3.337703 -0.771872
H -6.305759 2.422467 0.358344
H -6.099764 0.422669 1.820312
H -3.888326 -0.669151 2.122628
C 4.100316 -0.873082 1.680312
C 5.327785 -1.077507 1.074119
C 5.411356 -1.630758 -0.207975
C 4.229512 -1.990891 -0.861951
C 2.995046 -1.800585 -0.264248
H 4.053186 -0.455471 2.684861
H 6.243107 -0.804700 1.598953
C 6.738394 -1.809342 -0.878207
H 4.281045 -2.420443 -1.862072
H 2.079102 -2.063564 -0.792080
H 6.980004 -0.935656 -1.499949
H 7.549436 -1.923528 -0.148923
H 6.742388 -2.685261 -1.538268

TS_{ar}^{B}

55
Energy: -4079.27029944
Cu 0.662033 -0.641161 0.335925
Cl 0.244268 -1.325331 2.353506
C 2.393817 -1.524652 0.311809
O -0.895106 0.603578 0.348146
S -0.949676 2.062678 -0.022776
O -1.411263 2.276027 -1.401493
C -2.351065 2.598679 1.031466
O 0.216440 2.822908 0.412944
F -2.597789 3.880959 0.805632
F -3.427867 1.887697 0.730955
F -2.051095 2.428599 2.305543
C 2.477577 -2.641843 -0.509566
C 3.437960 -3.610020 -0.222908
C 4.305214 -3.437990 0.850365
C 4.213730 -2.300550 1.651681
C 3.253065 -1.333385 1.388846
H 1.795549 -2.766354 -1.348209
H 3.507464 -4.495836 -0.851573
H 5.060825 -4.191752 1.063691
H 4.884354 -2.168955 2.498668
H 3.171039 -0.445006 2.011899
C -1.499051 -1.289858 -1.421337
O -0.617349 -2.012929 -0.929738
C -2.906896 -1.354197 -1.000762
N -1.171431 -0.357710 -2.363917
C 0.229488 -0.165417 -2.628210
C 1.119759 0.005267 -1.452311
C 2.231566 0.156343 -0.875607

C 3.444551 0.921603 -0.677348
C 3.334102 2.272335 -0.327705
C 4.490519 3.032022 -0.189162
C 5.738297 2.456029 -0.403474
C 5.842845 1.108980 -0.750450
C 4.700281 0.334868 -0.876524
H -1.705384 0.512164 -2.327576
H 0.620463 -1.028099 -3.188108
H 0.338251 0.723822 -3.263366
H 2.346434 2.701623 -0.156888
H 4.411657 4.080976 0.088975
H 6.639223 3.057778 -0.296021
H 6.820684 0.661534 -0.917235
H 4.766395 -0.722113 -1.132492
C -3.949835 -0.877647 -1.797605
C -5.254236 -0.913427 -1.325702
C -5.541983 -1.409236 -0.052196
C -4.488290 -1.886091 0.734814
C -3.183749 -1.865385 0.271148
H -3.746792 -0.504337 -2.800811
H -6.067570 -0.551426 -1.954719
C -6.944571 -1.413003 0.474874
H -4.700586 -2.271703 1.732188
H -2.358403 -2.212405 0.892023
H -7.104190 -0.577919 1.171424
H -7.681613 -1.313181 -0.330889
H -7.164093 -2.335893 1.026586

I_2

55
Energy: -4079.32543352
Cu 0.176357 -0.536916 0.692881
Cl 1.108064 -2.086596 1.955125
C -2.166835 0.970170 1.541042
O -1.251180 -1.587322 0.106319
S -1.119242 -1.910035 -1.386792
O 0.267267 -1.746460 -1.829247
C -1.442724 -3.716799 -1.352896
O -2.171583 -1.310302 -2.192854
F -1.396653 -4.170330 -2.597158
F -0.526815 -4.323900 -0.628019
F -2.641922 -3.944754 -0.847430
C -1.285140 0.684970 2.591988
C -1.749110 0.087219 3.759891
C -3.098624 -0.211896 3.897164
C -3.987198 0.101161 2.869301
C -3.527457 0.689970 1.699827
H -0.241408 0.996906 2.529378
H -1.047049 -0.144788 4.558225
H -3.462998 -0.684839 4.807179
H -5.045805 -0.129150 2.976531
H -4.215493 0.906861 0.883532
C 2.285334 0.902914 -0.192442
O 1.684308 0.542468 0.858005
C 3.752392 0.966045 -0.153524
N 1.650841 1.222501 -1.321719
C 0.199694 1.272060 -1.501222
C -0.549248 0.933360 -0.267836
C -1.652478 1.478525 0.251315
C -2.414876 2.535361 -0.455106
C -2.790361 2.363197 -1.791442
C -3.511850 3.355603 -2.447844
C -3.864225 4.522479 -1.777309
C -3.508136 4.691402 -0.440740
C -2.795477 3.700062 0.220604
H 2.214870 1.299397 -2.159080
H -0.074752 2.297683 -1.798195
H -0.051755 0.594642 -2.329460
H -2.554051 1.423904 -2.293850
H -3.811372 3.208459 -3.484107
H -4.428778 5.298084 -2.292206
H -3.791703 5.599204 0.089261
H -2.521781 3.821331 1.268662
C 4.491361 1.834150 -0.961695
C 5.876072 1.831755 -0.890982
C 6.549239 0.963557 -0.026233
C 5.796266 0.106436 0.782561
C 4.412011 0.105071 0.730909
H 3.987648 2.546635 -1.615416
H 6.450219 2.521442 -1.509246
C 8.045789 0.937534 0.025623

H 6.310027 -0.571355 1.464057
H 3.821070 -0.565565 1.354737
H 8.409431 0.805034 1.052058
H 8.443088 0.100638 -0.565737
H 8.482668 1.859178 -0.376347

TS_{rc}^{B}

55
Energy: -4079.29389565
Cu -0.018373 -1.241395 0.354184
Cl 1.315437 -2.849454 -0.302506
C -0.806205 1.930630 1.438261
O -1.808430 -1.304643 1.001655
S -2.880372 -1.264988 -0.077863
O -2.303685 -1.117424 -1.415877
C -3.492201 -2.991860 -0.015780
O -4.005463 -0.418366 0.287395
F -4.456389 -3.147123 -0.910880
F -2.500917 -3.823975 -0.293101
F -3.967081 -3.262768 1.189047
C -0.430386 1.048691 2.462748
C -0.524217 1.421410 3.792444
C -0.999061 2.685613 4.133684
C -1.380891 3.570700 3.130883
C -1.288046 3.198220 1.796871
H -0.084440 0.050778 2.210817
H -0.238213 0.713961 4.568182
H -1.079417 2.975150 5.179948
H -1.758405 4.558864 3.387406
H -1.587127 3.898464 1.019177
C 2.435343 0.081005 -0.545214
O 1.675270 0.143121 0.460978
C 3.884910 -0.044489 -0.384253
N 1.886619 0.196305 -1.757835
C 0.433016 0.160821 -1.839121
C -0.074772 0.492627 -0.475633
C -0.679426 1.580457 0.021119
C -1.339003 2.452366 -0.997630
C -2.719807 2.384041 -1.192923
C -3.322076 3.219203 -2.128175
C -2.557234 4.118030 -2.866365
C -1.179888 4.185080 -2.672873
C -0.569865 3.351071 -1.741986
H 2.448201 0.075826 -2.590327
H 0.073270 0.924606 -2.541036
H 0.086884 -0.821944 -2.190201
H -3.310704 1.666754 -0.620858
H -4.397370 3.159321 -2.285037
H -3.035246 4.767516 -3.597787
H -0.579525 4.891167 -3.244278
H 0.508013 3.398178 -1.576125
C 4.678390 -0.656346 -1.358554
C 6.046310 -0.766221 -1.166253
C 6.651636 -0.273270 -0.006562
C 5.844846 0.326734 0.964784
C 4.474620 0.430704 0.789939
H 4.225182 -1.101858 -2.244190
H 6.659735 -1.257067 -1.921358
C 8.126166 -0.421901 0.208768
H 6.303585 0.711376 1.875415
H 3.846743 0.888386 1.551443
H 8.528095 0.391586 0.824583
H 8.351152 -1.363620 0.728859
H 8.674623 -0.437192 -0.740612

Reaction 32

reactant

34
Energy: -786.179182908
C -0.001351 0.004253 0.000476
O -0.000536 -0.001838 1.223282
C -1.261774 -0.036599 -0.800630
N 1.165268 0.049116 -0.709414
H 1.177916 -0.188930 -1.691752
C 2.428742 0.007233 0.010298
C 3.553622 0.125617 -0.903213
H 2.431421 0.822908 0.747488
H 2.502900 -0.922297 0.598725
C 4.486912 0.213442 -1.670331

C 5.591258 0.320831 -2.568095
C 5.971090 1.569538 -3.080249
C 7.046870 1.670498 -3.951734
C 7.756744 0.531439 -4.322645
C 7.386559 -0.712691 -3.818622
C 6.311177 -0.820859 -2.947527
H 5.411293 2.454437 -2.782440
H 7.334661 2.644612 -4.343481
H 8.600691 0.613633 -5.005435
H 7.940271 -1.604786 -4.106352
H 6.014848 -1.789027 -2.547575
C -1.341262 0.376665 -2.131694
C -2.557881 0.312430 -2.801397
C -3.689237 -0.164143 -2.149632
C -3.631169 -0.574722 -0.814780
C -2.409100 -0.494601 -0.152885
H -0.470224 0.791055 -2.638618
H -2.626684 0.648035 -3.834885
H -4.641559 -0.210299 -2.679734
C -4.851994 -1.102900 -0.121460
H -2.322708 -0.784901 0.894090
H -4.765109 -1.013422 0.967918
H -5.757070 -0.567391 -0.435337
H -5.011568 -2.166336 -0.349436

I$_c$

69
Energy: -4386.80672648
Cu -1.267200 0.422118 0.550414
Cl -1.953720 0.226458 2.598162
C -1.803162 2.230812 0.799586
O -0.837585 -1.533134 0.415586
S -0.274152 -2.377003 -0.695497
O 0.890954 -1.790229 -1.363003
C 0.409806 -3.759715 0.300100
O -1.291714 -2.987354 -1.543009
F 1.114765 -4.567903 -0.477728
F 1.214696 -3.286951 1.255799
F -0.565405 -4.442329 0.873032
C -0.766331 3.131140 0.658764
C -1.098903 4.484990 0.551064
C -2.422380 4.898587 0.624703
C -3.435181 3.960550 0.810602
C -3.129702 2.604413 0.911324
C 1.894564 1.944273 -1.594999
O 1.589171 3.113679 -1.386904
C 3.277850 1.445861 -1.354154
N 1.015965 1.022266 -2.093522
C -0.348368 1.405548 -2.325969
C -1.373959 0.730040 -1.502571
C -2.483466 0.213287 -1.320888
C -3.799134 -0.258894 -1.076160
C -4.051224 -1.602905 -0.752410
C -5.356964 -2.010337 -0.525016
C -6.408051 -1.101488 -0.627417
C -6.163122 0.229816 -0.959795
C -4.863280 0.657456 -1.178210
H 0.272293 2.823756 0.582250
H -0.295263 5.202432 0.391908
H -2.668259 5.956217 0.547700
H -4.473475 4.278423 0.896769
H -3.911349 1.868400 1.091682
H 1.198294 0.030439 -1.933419
H -0.408703 2.488737 -2.139936
H -0.629539 1.229708 -3.375864
H -3.219157 -2.302388 -0.700481
H -5.557377 -3.048668 -0.269896
H -7.429456 -1.433913 -0.449724
H -6.988181 0.933910 -1.046036
H -4.648630 1.693273 -1.436821
O 2.629029 -0.680861 0.910077
C 3.549848 -1.548312 1.587695
C 4.175159 -2.447518 0.553858
C 1.634174 -0.072838 1.535792
O 0.909804 0.659832 0.874950
C 1.431045 -0.308577 3.000089
H 4.302247 -0.922958 2.092324
H 3.021016 -2.137072 2.346577
H 4.856990 -3.155557 1.040072
H 3.400317 -3.012509 0.024058
H 4.739215 -1.863314 -0.181372

H 2.370120 -0.261697 3.562898
H 0.719596 0.425851 3.383056
H 0.990795 -1.305014 3.144822
C 3.827035 0.375486 -2.057625
C 5.149502 0.011458 -1.825242
C 5.904617 0.684182 -0.871432
C 5.361486 1.748579 -0.144106
C 4.051147 2.129564 -0.415368
H 3.239832 -0.153826 -2.806490
H 5.594153 -0.804669 -2.393674
C 6.176575 2.455474 0.898905
H 3.601226 2.977585 0.101409
H 6.939837 0.389464 -0.690985
H 5.725886 3.415061 1.178840
H 6.264921 1.854926 1.815957
H 7.198096 2.651967 0.547992

I_1

55
Energy: -4079.28167363
Cu 0.016926 -0.048653 0.004580
Cl 0.073198 -0.159479 2.129439
C 1.884142 -0.106136 0.290368
O -1.996179 0.321784 -0.218911
S -1.911541 1.757451 -0.680305
O -2.598002 2.055711 -1.922799
C -2.809378 2.661243 0.638987
O -0.498871 2.183314 -0.555133
F -2.780265 3.957160 0.371973
F -4.065416 2.246615 0.676911
F -2.236629 2.439629 1.807687
C 2.597875 -1.287404 0.255659
C 3.983898 -1.181081 0.129160
C 4.598217 0.067922 0.074496
C 3.836989 1.229400 0.150347
C 2.449331 1.155451 0.284772
H 2.101608 -2.257569 0.300304
H 4.580530 -2.090936 0.079709
H 5.681347 0.134977 -0.012164
H 4.315349 2.207153 0.122868
H 1.841412 2.054154 0.375043
C -0.663427 -3.253075 0.527541
O 0.388689 -3.636891 0.027342
C -1.025819 -3.512185 1.944578
N -1.576006 -2.547799 -0.217337
C -1.243337 -2.207885 -1.580671
C -0.215384 -1.154333 -1.735342
C 0.544101 -0.353704 -2.279756
C 1.505822 0.509056 -2.870039
C 1.179267 1.838844 -3.179008
C 2.146209 2.666154 -3.730326
C 3.427057 2.177394 -3.980019
C 3.750729 0.854764 -3.683990
C 2.795026 0.018108 -3.129035
H -2.249374 -1.950383 0.250574
H -0.874496 -3.109413 -2.084902
H -2.156702 -1.871782 -2.085019
H 0.178735 2.206187 -2.960901
H 1.900334 3.699522 -3.965479
H 4.180842 2.834242 -4.411275
H 4.752743 0.478605 -3.880742
H 3.030891 -1.013362 -2.872574
C 0.014126 -3.792649 2.830275
C -0.230976 -4.018245 4.181625
C -1.554472 -3.981323 4.628284
C -2.601495 -3.720782 3.751572
C -2.342279 -3.481242 2.407907
H 1.032633 -3.816711 2.441874
H -1.764250 -4.164736 5.682816
H -3.626873 -3.710661 4.116733
H -3.168928 -3.307115 1.720389
C 0.898667 -4.266950 5.136108
H 0.581924 -4.886108 5.984620
H 1.738556 -4.770703 4.642172
H 1.283783 -3.323197 5.547071

TS_{rc}^A

55
Energy: -4079.26013438
Cu -0.614713 -0.602554 -0.523946

```
 Cl 0.093707 -1.522130 -2.330611
 C -2.036329 -1.835717 -0.551202
 O 0.766430 0.814228 -0.552449
 S 1.117280 2.093962 0.161947
 O 2.398331 2.013534 0.868538
 C 1.405441 3.203791 -1.267274
 O 0.006066 2.696475 0.896425
 F 1.725902 4.410760 -0.827717
 F 2.379612 2.740566 -2.028276
 F 0.289615 3.286228 -1.983900
 C -1.976872 -2.977960 0.226097
 C -3.091433 -3.817576 0.243073
 C -4.219364 -3.508642 -0.510431
 C -4.240090 -2.360252 -1.295512
 C -3.135824 -1.510088 -1.326465
 H -1.094892 -3.207983 0.820970
 H -3.070424 -4.717757 0.856039
 H -5.083971 -4.169902 -0.491215
 H -5.114950 -2.120385 -1.897900
 H -3.149554 -0.612416 -1.944653
 C 1.637142 -0.934426 1.761933
 O 0.598000 -1.532698 1.397081
 C 2.952306 -1.197925 1.185289
 N 1.484871 0.001577 2.736544
 C 0.097594 0.127437 3.165681
 C -0.782630 -0.127186 2.015881
 C -1.531817 0.136257 1.030546
 C -2.780738 0.856752 0.810129
 C -2.858689 1.926698 -0.086179
 C -4.086796 2.528219 -0.330042
 C -5.239088 2.063292 0.298215
 C -5.161683 0.990990 1.181626
 C -3.937853 0.386823 1.439491
 H 2.042240 0.851484 2.625538
 H -0.098284 -0.572741 3.989006
 H -0.085247 1.152940 3.511692
 H -1.953465 2.298259 -0.562416
 H -4.141710 3.367673 -1.020587
 H -6.198779 2.535408 0.095666
 H -6.059372 0.617884 1.671434
 H -3.870538 -0.477138 2.100344
 C 4.127196 -0.822951 1.844659
 C 5.348958 -1.070713 1.238789
 C 5.396120 -1.675419 -0.014899
 C 4.231164 -2.057047 -0.685007
 C 3.008411 -1.818014 -0.065152
 H 4.083409 -0.356448 2.827348
 H 6.271829 -0.785379 1.739721
 H 6.361610 -1.851847 -0.490767
 C 4.282960 -2.711862 -2.032529
 H 2.082817 -2.082424 -0.576539
 H 5.137474 -2.354635 -2.620322
 H 4.382758 -3.802619 -1.941639
 H 3.366859 -2.513299 -2.601892
```

TS_{ar}^{B}

```
  55
Energy: -4079.26990549
 Cu -0.580235 -0.670388 -0.213947
 Cl -0.058141 -1.610126 -2.103556
 C -2.324593 -1.523167 -0.182526
 O 0.952072 0.601334 -0.329599
 S 0.987070 2.090615 -0.107494
 O 1.291158 2.448756 1.285872
 C 2.515806 2.501304 -1.033678
 O -0.107196 2.808091 -0.751431
 F 2.747719 3.801992 -0.933013
 F 3.535758 1.834922 -0.512221
 F 2.380779 2.177727 -2.306090
 C -2.476119 -2.555562 0.733931
 C -3.442685 -3.528677 0.486028
 C -4.248489 -3.441888 -0.644113
 C -4.089691 -2.386337 -1.541246
 C -3.121612 -1.417010 -1.317573
 H -1.843500 -2.611165 1.617730
 H -3.565772 -4.350461 1.189046
 H -5.009101 -4.198565 -0.827140
 H -4.712526 -2.322352 -2.431419
 H -2.986664 -0.592056 -2.014437
 C 1.515878 -1.103605 1.652220
 O 0.646136 -1.892853 1.247887
```

C 2.924460 -1.187228 1.228708
N 1.165203 -0.074708 2.477616
C -0.244391 0.121378 2.691014
C -1.102446 0.161286 1.479420
C -2.189263 0.264904 0.848251
C -3.383021 1.016796 0.524220
C -3.239154 2.323464 0.043452
C -4.377906 3.076749 -0.219788
C -5.641422 2.538267 -0.000507
C -5.779383 1.234732 0.476475
C -4.654115 0.465984 0.728709
H 1.679845 0.798340 2.345612
H -0.637886 -0.687097 3.324527
H -0.379997 1.069869 3.227277
H -2.240114 2.724147 -0.132285
H -4.272558 4.090743 -0.599963
H -6.528560 3.134892 -0.206217
H -6.769537 0.816447 0.646209
H -4.745540 -0.558878 1.087088
C 3.955159 -0.539186 1.914470
C 5.243452 -0.580958 1.399781
C 5.496658 -1.251987 0.206844
C 4.476722 -1.905273 -0.489272
C 3.189682 -1.869404 0.040446
H 3.756781 -0.023380 2.853020
H 6.055435 -0.082528 1.925986
H 6.508656 -1.264970 -0.199958
C 4.747665 -2.629840 -1.773837
H 2.361135 -2.337483 -0.492300
H 3.892148 -2.554817 -2.456455
H 5.630627 -2.227636 -2.285457
H 4.929963 -3.699384 -1.597843

I_2

55
Energy: -4079.32499195
Cu -0.306309 0.484879 0.619179
Cl -1.428041 1.971169 1.801144
C 2.099894 -0.825855 1.622711
O 1.069546 1.638666 0.110979
S 1.005248 1.937487 -1.391874
O -0.338202 1.674113 -1.912081
C 1.205897 3.762424 -1.365478
O 2.141858 1.403285 -2.126521
F 1.199973 4.198206 -2.616836
F 0.213465 4.315742 -0.701081
F 2.357530 4.074435 -0.798034
C 1.138868 -0.601323 2.617375
C 1.486825 0.038636 3.802963
C 2.799403 0.441275 4.014673
C 3.767959 0.190013 3.043696
C 3.423526 -0.440769 1.856663
H 0.127122 -0.992188 2.497806
H 0.723785 0.221594 4.556814
H 3.072653 0.946968 4.938916
H 4.797840 0.501591 3.209285
H 4.172729 -0.609945 1.084169
C -2.232736 -1.149770 -0.357496
O -1.731717 -0.707726 0.713126
C -3.688500 -1.363199 -0.396589
N -1.510491 -1.426307 -1.443712
C -0.053492 -1.336317 -1.547276
C 0.591388 -0.931279 -0.276118
C 1.703393 -1.381443 0.311273
C 2.589483 -2.374902 -0.340974
C 3.021143 -2.180638 -1.657268
C 3.858846 -3.112169 -2.262744
C 4.272337 -4.239981 -1.561046
C 3.859904 -4.429464 -0.243563
C 3.030585 -3.498254 0.367305
H -2.019656 -1.585152 -2.304496
H 0.330792 -2.331025 -1.826490
H 0.177138 -0.635920 -2.362639
H 2.734108 -1.268616 -2.182767
H 4.200566 -2.946893 -3.283104
H 4.927829 -4.967977 -2.036180
H 4.190490 -5.305978 0.311258
H 2.711994 -3.635348 1.400612
C -4.278278 -2.331585 -1.211843
C -5.660810 -2.462652 -1.214535
C -6.439966 -1.630577 -0.418213

C -5.862895 -0.658842 0.406020
C -4.476299 -0.539936 0.413940
H -3.665340 -3.008825 -1.806366
H -6.133260 -3.222365 -1.834011
H -7.525480 -1.737180 -0.428948
C -6.722140 0.228727 1.255158
H -3.984318 0.207581 1.038298
H -6.118388 0.927231 1.844601
H -7.413349 0.819652 0.639588
H -7.334740 -0.359091 1.951725

TS_{rc}^{B}

55
Energy: -4079.29328316
Cu -0.042527 -1.275753 0.317521
Cl 1.150157 -2.970305 -0.385165
C -0.565898 1.957440 1.373724
O -1.790802 -1.199845 1.068450
S -2.917200 -1.118873 0.047663
O -2.407897 -1.041465 -1.323052
C -3.630843 -2.801176 0.191632
O -3.964888 -0.193365 0.449531
F -4.645100 -2.919712 -0.652055
F -2.707443 -3.701534 -0.106519
F -4.062280 -3.008104 1.425122
C -0.187022 1.074821 2.396547
C -0.189154 1.477537 3.720784
C -0.572788 2.773356 4.058216
C -0.956169 3.659854 3.057319
C -0.955193 3.257355 1.728764
H 0.088586 0.054182 2.148991
H 0.097000 0.769612 4.496032
H -0.581057 3.086881 5.100617
H -1.262863 4.672905 3.311190
H -1.254462 3.959129 0.952604
C 2.451961 -0.130786 -0.725259
O 1.755056 -0.001553 0.317301
C 3.900023 -0.352394 -0.644662
N 1.851828 -0.000447 -1.911436
C 0.396655 0.058586 -1.924487
C -0.025600 0.441752 -0.545216
C -0.532768 1.574549 -0.040206
C -1.187288 2.468495 -1.043369
C -2.577650 2.481291 -1.170844
C -3.173461 3.337313 -2.091259
C -2.392887 4.175999 -2.881879
C -1.006121 4.161299 -2.756509
C -0.402561 3.306144 -1.840755
H 2.365223 -0.171141 -2.766264
H 0.054814 0.835107 -2.621590
H -0.027440 -0.904586 -2.242552
H -3.182617 1.811055 -0.557729
H -4.256830 3.341253 -2.194865
H -2.866147 4.842306 -3.601155
H -0.393051 4.820272 -3.369203
H 0.683108 3.289276 -1.728162
C 4.570571 -1.086950 -1.623793
C 5.939822 -1.285556 -1.502357
C 6.627297 -0.752224 -0.418812
C 5.966108 -0.026807 0.577977
C 4.592799 0.156869 0.456330
H 4.021488 -1.560144 -2.437481
H 6.470431 -1.873330 -2.248447
H 7.702344 -0.913029 -0.329990
C 6.725878 0.531341 1.743592
H 4.041640 0.705529 1.219288
H 7.313572 -0.248372 2.245292
H 7.431984 1.309669 1.423822
H 6.054705 0.975876 2.487110

Reaction 33

reactant

34
Energy: -786.177300649
C -0.014156 -0.367337 0.040395
O -0.038428 -0.545799 1.250194
C 1.244643 -0.303695 -0.763049
N -1.171210 -0.228003 -0.676330
H -1.148223 0.138935 -1.618144

C -2.449536 -0.244875 0.015706
C -3.550340 -0.281569 -0.933906
H -2.464953 -1.123071 0.677038
H -2.541711 0.628733 0.682352
C -4.453095 -0.302201 -1.741258
C -5.515360 -0.337507 -2.693670
C -5.594846 -1.382693 -3.624887
C -6.624367 -1.414439 -4.555304
C -7.587948 -0.409286 -4.567742
C -7.518068 0.631036 -3.644791
C -6.489118 0.670857 -2.713735
H -4.838849 -2.165771 -3.605161
H -6.676885 -2.229807 -5.274772
H -8.395210 -0.436953 -5.297637
H -8.270402 1.417879 -3.651468
H -6.425529 1.481259 -1.989651
C 1.260353 -0.810992 -2.065762
C 2.417096 -0.786235 -2.833577
C 3.575071 -0.238150 -2.295544
C 3.566207 0.262964 -0.998843
C 2.419793 0.233383 -0.202839
H 0.357099 -1.266426 -2.471171
H 2.413559 -1.196399 -3.841726
H 4.491192 -0.204112 -2.883188
H 4.478121 0.690748 -0.581246
C 2.483728 0.773452 1.195327
H 3.435374 1.293278 1.360017
H 1.665437 1.471749 1.407655
H 2.388645 -0.029615 1.934806

I_c

69
Energy: -4386.80069210
Cu -1.323358 0.326186 0.585571
Cl -2.313076 -0.143577 2.464584
C -1.789366 2.116788 1.032623
O -1.022901 -1.611086 0.200524
S -0.246000 -2.324419 -0.871300
O 0.959795 -1.618925 -1.307785
C 0.382662 -3.752427 0.099384
O -1.088854 -2.908594 -1.907460
F 1.288059 -4.414642 -0.606325
F 0.960329 -3.326538 1.226042
F -0.607337 -4.566299 0.418098
C -0.710308 2.972988 1.141146
C -0.979166 4.342365 1.239242
C -2.286122 4.810307 1.257175
C -3.347126 3.911383 1.178721
C -3.104753 2.543079 1.074869
C 2.208669 2.001882 -1.122990
O 1.937126 3.101892 -0.649833
C 3.593636 1.439088 -1.112157
N 1.266796 1.259978 -1.787098
C -0.064937 1.767245 -1.943968
C -1.169020 0.959844 -1.383507
C -2.298633 0.455209 -1.429344
C -3.631896 -0.029498 -1.427598
C -3.910853 -1.406980 -1.428481
C -5.231637 -1.828921 -1.430718
C -6.269604 -0.899558 -1.441315
C -5.996598 0.467395 -1.452526
C -4.682867 0.907586 -1.440716
H 0.320318 2.630711 1.096734
H -0.137311 5.031772 1.284683
H -2.482338 5.877792 1.340497
H -4.375995 4.267005 1.216245
H -3.929299 1.832617 1.052026
H 1.425823 0.280193 -1.999750
H -0.080080 2.760774 -1.471108
H -0.301226 1.906569 -3.010957
H -3.087287 -2.119095 -1.435216
H -5.453461 -2.893870 -1.426755
H -7.302732 -1.243053 -1.444198
H -6.811031 1.188711 -1.468949
H -4.446562 1.970692 -1.450490
O 2.518230 -0.893977 1.528070
C 3.222247 -1.832274 2.354397
C 4.128765 -2.616187 1.443365
C 1.396922 -0.310082 1.923984
O 0.806436 0.388479 1.112753
C 0.912829 -0.525690 3.323295

H 3.786891 -1.279406 3.119021
H 2.506049 -2.494771 2.856281
H 4.673978 -3.377902 2.012680
H 3.532898 -3.114364 0.667627
H 4.852457 -1.953352 0.955363
H 1.731559 -0.571208 4.049050
H 0.215745 0.273723 3.584912
H 0.351253 -1.469648 3.368501
C 3.803501 0.058071 -1.190658
C 5.086553 -0.468335 -1.263531
C 6.181549 0.386446 -1.243868
C 5.979758 1.756825 -1.143150
C 4.701039 2.314651 -1.073493
H 2.959225 -0.629686 -1.160113
H 5.224132 -1.545772 -1.345146
H 6.839501 2.427325 -1.127317
C 4.584993 3.808782 -0.983815
H 7.193601 -0.010788 -1.309237
H 3.908937 4.218579 -1.742870
H 4.171320 4.123188 -0.019295
H 5.572194 4.270640 -1.109938

I_1

55
Energy: -4079.28132215
Cu 0.118821 -0.015940 0.017630
Cl 0.245644 -0.105808 2.141883
C 1.997767 -0.089096 0.193431
O -1.892133 0.366283 -0.134836
S -1.817741 1.800255 -0.605797
O -2.547780 2.095378 -1.824063
C -2.661789 2.717898 0.739828
O -0.399130 2.218290 -0.535593
F -2.628384 4.012017 0.464582
F -3.919658 2.316110 0.821640
F -2.052046 2.496792 1.889496
C 2.699212 -1.273113 0.081949
C 4.081317 -1.173387 -0.089600
C 4.704032 0.071860 -0.122891
C 3.955907 1.235423 0.021164
C 2.573641 1.167334 0.206902
H 2.196671 -2.240903 0.100603
H 4.666801 -2.085617 -0.196004
H 5.783595 0.134104 -0.248522
H 4.441246 2.209967 0.008594
H 1.978462 2.067254 0.352836
C -0.586974 -3.269190 0.629579
O 0.451470 -3.668931 0.114235
C -1.006542 -3.587619 2.022039
N -1.473073 -2.507202 -0.095548
C -1.182103 -2.197798 -1.474209
C -0.177277 -1.132865 -1.693285
C 0.542997 -0.332517 -2.290654
C 1.466072 0.530596 -2.937783
C 1.112975 1.853339 -3.248574
C 2.046026 2.681941 -3.853470
C 3.319402 2.201826 -4.153698
C 3.669012 0.886162 -3.856235
C 2.747123 0.048002 -3.249343
H -2.145868 -1.919360 0.384589
H -0.812050 -3.108356 -1.961557
H -2.113667 -1.890385 -1.964064
H 0.120036 2.214725 -2.989909
H 1.779891 3.709880 -4.090133
H 4.046877 2.860150 -4.625912
H 4.664766 0.516711 -4.093136
H 3.004644 -0.978002 -2.992224
C -0.050526 -3.764014 3.043027
C -0.516807 -4.077685 4.319823
C -1.870957 -4.234404 4.593035
C -2.805520 -4.076416 3.577661
C -2.367310 -3.750513 2.301550
C 1.423444 -3.597772 2.824314
H -2.193821 -4.484595 5.602405
H -3.867666 -4.208227 3.774053
H -3.093123 -3.652569 1.495235
H 0.210956 -4.200874 5.122008
H 1.967560 -3.755778 3.763010
H 1.802001 -4.297609 2.071918
H 1.656607 -2.584082 2.468345

TS_{rc}^{A}

55
Energy: -4079.25666006
Cu -0.691781 -0.572090 -0.616667
Cl -0.206877 -1.375642 -2.543220
C -2.193301 -1.706071 -0.621609
O 0.805628 0.731360 -0.642193
S 1.271906 1.965673 0.083196
O 2.611701 1.808423 0.657486
C 1.469413 3.129737 -1.317910
O 0.262795 2.573795 0.948393
F 1.871071 4.304979 -0.859302
F 2.353542 2.670424 -2.183621
F 0.296822 3.279579 -1.924882
C -2.150327 -2.908540 0.060353
C -3.315785 -3.674383 0.112296
C -4.477498 -3.235286 -0.514556
C -4.482142 -2.028726 -1.207387
C -3.327413 -1.250837 -1.271657
H -1.240714 -3.240805 0.557330
H -3.307520 -4.620184 0.652528
H -5.381735 -3.839795 -0.469062
H -5.384569 -1.686450 -1.711628
H -3.328478 -0.308115 -1.818637
C 1.717652 -1.201609 1.402822
O 0.604436 -1.720338 1.135007
C 2.928134 -1.544362 0.649891
N 1.711363 -0.317579 2.435664
C 0.384025 -0.143332 3.012624
C -0.616528 -0.274074 1.945879
C -1.434076 0.101180 1.056480
C -2.651283 0.901481 0.999915
C -2.742877 2.033455 0.185150
C -3.947466 2.719453 0.096637
C -5.065258 2.277975 0.799678
C -4.976066 1.143688 1.600805
C -3.774417 0.454603 1.703201
H 2.316139 0.499348 2.350194
H 0.228415 -0.885188 3.807651
H 0.303430 0.865877 3.436557
H -1.863112 2.383642 -0.351024
H -4.011524 3.606527 -0.530685
H -6.007727 2.816700 0.719383
H -5.847827 0.788543 2.147533
H -3.702201 -0.455975 2.297614
C 4.246117 -1.201886 1.031016
C 5.284523 -1.627295 0.202625
C 5.052526 -2.341993 -0.966124
C 3.752926 -2.665822 -1.338688
C 2.702927 -2.273538 -0.528180
C 4.605539 -0.403429 2.248917
H 6.307004 -1.378177 0.485238
H 5.893588 -2.643947 -1.588232
H 3.553401 -3.213393 -2.256837
H 1.680392 -2.511170 -0.812327
H 5.682927 -0.466737 2.438170
H 4.368350 0.660847 2.099814
H 4.084822 -0.745818 3.151363

TS_{ar}^{B}

55
Energy: -4079.26759578
Cu 0.600851 -0.508800 0.397739
Cl 0.467818 -0.740448 2.555343
C 2.292267 -1.470371 0.321774
O -1.049125 0.568390 0.342504
S -1.098006 2.035794 -0.013210
O -1.604631 2.255738 -1.372876
C -2.449898 2.572204 1.103347
O 0.098948 2.767718 0.384789
F -2.692394 3.859377 0.905993
F -3.543138 1.873735 0.834797
F -2.098491 2.377949 2.360081
C 2.248708 -2.650659 -0.410600
C 3.185945 -3.644324 -0.131405
C 4.159188 -3.435451 0.838748
C 4.197942 -2.234156 1.545797
C 3.261544 -1.241377 1.293172
H 1.487227 -2.804514 -1.172364
H 3.154008 -4.578659 -0.688854

```
 H 4.896808 -4.209246 1.043271
 H 4.953259 -2.070777 2.312125
 H 3.282559 -0.304029 1.844831
 C -1.700217 -1.337504 -1.314813
 O -0.812613 -2.019545 -0.784145
 C -3.138323 -1.413949 -0.975185
 N -1.369530 -0.436571 -2.295857
 C 0.023474 -0.271388 -2.598678
 C 0.952510 -0.035482 -1.467999
 C 2.096009 0.139769 -0.964454
 C 3.315398 0.910575 -0.856307
 C 3.222102 2.264397 -0.513968
 C 4.382873 3.027673 -0.453592
 C 5.616612 2.451192 -0.737455
 C 5.703971 1.100446 -1.075120
 C 4.557832 0.322793 -1.123126
 H -1.896611 0.437316 -2.285064
 H 0.395092 -1.169202 -3.115638
 H 0.123026 0.574795 -3.292152
 H 2.244971 2.692986 -0.288834
 H 4.319060 4.079311 -0.182016
 H 6.520879 3.055677 -0.691354
 H 6.671333 0.653188 -1.294944
 H 4.609606 -0.738167 -1.366526
 C -4.076396 -0.990621 -1.926818
 C -5.434848 -0.999694 -1.653611
 C -5.868916 -1.436992 -0.407557
 C -4.946351 -1.872420 0.534783
 C -3.572410 -1.883093 0.283941
 H -3.732187 -0.675695 -2.910278
 H -6.146784 -0.671214 -2.407935
 H -6.931346 -1.446995 -0.168930
 H -5.295181 -2.217351 1.507956
 C -2.649535 -2.358335 1.362346
 H -2.023392 -3.193186 1.030185
 H -1.960694 -1.567064 1.685953
 H -3.225753 -2.678425 2.238325
```

I_2

```
  55
Energy: -4079.32473247
 Cu -0.258853 0.500851 0.684735
 Cl -1.114268 1.994511 2.063888
 C 2.187673 -0.803551 1.512685
 O 1.054565 1.638066 -0.035054
 S 0.655343 2.003039 -1.468422
 O -0.747520 1.648054 -1.708382
 C 0.710836 3.834720 -1.374519
 O 1.643735 1.595811 -2.454317
 F 0.369335 4.323422 -2.557775
 F -0.133913 4.257867 -0.458920
 F 1.936205 4.227250 -1.074360
 C 1.290641 -0.600299 2.569587
 C 1.705914 0.034799 3.736498
 C 3.023599 0.451998 3.866727
 C 3.930858 0.222945 2.832149
 C 3.518998 -0.401104 1.663739
 H 0.279103 -1.005963 2.511876
 H 0.988810 0.205668 4.536671
 H 3.349946 0.953226 4.775986
 H 4.965144 0.546876 2.934312
 H 4.219240 -0.554078 0.843478
 C -2.275128 -1.165259 -0.170822
 O -1.751592 -0.615252 0.834634
 C -3.714659 -1.496603 -0.132089
 N -1.604895 -1.499910 -1.273848
 C -0.169622 -1.336985 -1.497998
 C 0.550587 -0.927302 -0.272625
 C 1.710594 -1.361011 0.229872
 C 2.565294 -2.335814 -0.488261
 C 2.893199 -2.130249 -1.832603
 C 3.704638 -3.040615 -2.502188
 C 4.195213 -4.159365 -1.836376
 C 3.886200 -4.360436 -0.492569
 C 3.083125 -3.449841 0.181396
 H -2.164781 -1.785809 -2.068153
 H 0.232684 -2.309224 -1.825320
 H -0.035006 -0.617648 -2.319215
 H 2.544083 -1.225835 -2.333542
 H 3.965137 -2.866225 -3.544683
 H 4.830175 -4.871211 -2.361142
```

H 4.277080 -5.229989 0.033157
H 2.845213 -3.596060 1.234975
C -4.120373 -2.771810 -0.538763
C -5.460222 -3.129051 -0.502241
C -6.396231 -2.195284 -0.070046
C -5.991102 -0.924360 0.321484
C -4.649049 -0.540604 0.310914
H -3.370395 -3.499141 -0.850396
H -5.769554 -4.127436 -0.803637
H -7.452585 -2.456870 -0.039250
H -6.735270 -0.196833 0.644497
C -4.258420 0.848026 0.713572
H -3.603491 1.326445 -0.027823
H -5.148191 1.476377 0.832020
H -3.692793 0.865145 1.653773

TS_{rc}^{B}

55
Energy: -4079.29271955
Cu -0.141669 -1.363745 0.345381
Cl 0.908331 -3.172202 -0.296314
C -0.296606 1.943351 1.339361
O -1.888355 -1.079155 1.046875
S -2.963442 -0.902368 -0.016815
O -2.400167 -0.921394 -1.368557
C -3.864001 -2.491022 0.144103
O -3.913771 0.145617 0.321405
F -4.854156 -2.525414 -0.734842
F -3.035333 -3.495858 -0.092046
F -4.358748 -2.609115 1.365369
C -0.036421 1.047307 2.388133
C 0.013553 1.484754 3.700650
C -0.198544 2.828691 4.000530
C -0.465696 3.728389 2.974200
C -0.516804 3.291790 1.657306
H 0.103039 -0.007108 2.170557
H 0.204134 0.767705 4.496679
H -0.165319 3.169776 5.033714
H -0.640077 4.779083 3.198699
H -0.724964 4.004055 0.861164
C 2.500612 -0.397259 -0.602375
O 1.751371 -0.266516 0.401683
C 3.942958 -0.632936 -0.456212
N 1.963984 -0.256361 -1.816488
C 0.515285 -0.111463 -1.894189
C 0.074791 0.328090 -0.536991
C -0.300512 1.525558 -0.065022
C -0.816229 2.474324 -1.097467
C -2.190093 2.637798 -1.282584
C -2.649797 3.545507 -2.231550
C -1.749828 4.286492 -2.992207
C -0.379289 4.121790 -2.808620
C 0.088143 3.214110 -1.864377
H 2.522115 -0.415440 -2.645574
H 0.252358 0.672564 -2.616448
H 0.045557 -1.051923 -2.212724
H -2.888537 2.043140 -0.691203
H -3.720930 3.667361 -2.380660
H -2.117158 4.994062 -3.733572
H 0.326902 4.704013 -3.398308
H 1.160016 3.079645 -1.704722
C 4.547377 -1.592493 -1.273036
C 5.905745 -1.850176 -1.158819
C 6.656364 -1.134437 -0.232391
C 6.049250 -0.183039 0.580666
C 4.682646 0.082653 0.503714
H 3.933474 -2.178738 -1.957204
H 6.371584 -2.610559 -1.781418
H 7.724239 -1.322033 -0.135347
H 6.648678 0.371021 1.302666
C 4.062910 1.109354 1.406228
H 3.416601 1.811503 0.862102
H 3.428591 0.642374 2.170157
H 4.840267 1.690229 1.915314

Reaction 34

reactant

39
Energy: -751.717999135

C -4.120910 0.023203 0.152277
O -4.838699 0.084106 -0.838096
C -4.746381 -0.077334 1.552782
N -2.762042 0.034434 0.080851
H -2.219558 -0.014519 0.931452
C -1.993689 0.119960 -1.178985
C -0.574789 0.098576 -0.803700
C -2.297923 -1.085137 -2.074733
C -2.305327 1.430494 -1.908498
C 0.599376 0.079910 -0.504000
C 1.982323 0.055771 -0.150747
C 2.646813 -1.165459 0.030520
C 3.991093 -1.186493 0.375810
C 4.689406 0.006397 0.544083
C 4.037658 1.223859 0.365273
C 2.693277 1.251959 0.020524
H 2.092819 -2.092794 -0.104848
H 4.498161 -2.139907 0.514068
H 5.743751 -0.012732 0.814673
H 4.581236 2.158102 0.495453
H 2.175328 2.198830 -0.122044
C -3.743802 -0.121322 2.701802
C -5.657153 1.140244 1.719569
C -5.590316 -1.353466 1.565571
H -4.283240 -0.185840 3.656583
H -3.086556 -1.001684 2.647069
H -3.122502 0.785257 2.745855
H -6.137423 -1.435964 2.515322
H -6.312761 -1.343739 0.741671
H -4.960184 -2.248030 1.458484
H -6.208820 1.072800 2.667837
H -5.075898 2.073312 1.731384
H -6.376804 1.196744 0.895285
H -1.692941 -1.030751 -2.988266
H -2.055408 -2.018375 -1.552343
H -3.359605 -1.086369 -2.341402
H -1.701499 1.500162 -2.821779
H -3.367412 1.460377 -2.171967
H -2.067524 2.288469 -1.268196

I_c

74
Energy: -4352.34271646
Cu -0.878269 0.083282 0.778062
Cl -1.783554 -0.547299 2.669395
C -1.493248 1.804130 1.319172
O -0.227206 -1.780213 0.311143
S 0.364629 -2.247116 -0.991214
O 1.341708 -1.317521 -1.566455
C 1.396434 -3.645934 -0.402011
O -0.609087 -2.841351 -1.898816
F 1.950894 -4.259317 -1.432358
F 2.371064 -3.185675 0.392335
F 0.670072 -4.509973 0.287266
C -0.474184 2.722458 1.462841
C -0.836364 4.065376 1.617763
C -2.171244 4.440530 1.657644
C -3.169911 3.474297 1.547268
C -2.834878 2.132744 1.385770
C 1.781475 2.645097 -1.154963
O 1.303515 3.721411 -0.820752
C 3.304140 2.435079 -1.077729
N 1.059709 1.601333 -1.656863
C -0.340387 1.552164 -2.054062
C -1.177327 0.680406 -1.158660
C -2.214860 0.007128 -1.047441
C -3.448693 -0.656688 -0.851830
C -3.516424 -2.054563 -0.717299
C -4.749985 -2.656135 -0.524835
C -5.910614 -1.885364 -0.480197
C -5.850413 -0.500519 -0.629030
C -4.624071 0.118408 -0.807635
H 0.575058 2.448974 1.414171
H -0.042658 4.808084 1.673513
H -2.439168 5.488136 1.781998
H -4.220404 3.756501 1.603045
H -3.606768 1.366548 1.338879
H 1.518680 0.697472 -1.734021
C -1.024529 2.919115 -2.117422
C -0.388445 0.895243 -3.442641
H -2.598741 -2.636557 -0.774423

H -4.809452 -3.736449 -0.412239
H -6.874333 -2.369514 -0.331165
H -6.761477 0.093566 -0.601893
H -4.549468 1.198763 -0.928979
C 3.790489 1.053376 -1.503696
C 3.915289 3.488078 -2.007921
C 3.734090 2.719800 0.361789
O 3.237543 -0.509571 1.440042
C 4.218555 -1.394699 1.997169
C 5.343374 -1.464917 0.998679
C 1.979205 -0.467146 1.865077
O 1.239459 0.332528 1.308592
C 1.561440 -1.382840 2.969041
H 4.550792 -0.988385 2.963502
H 3.778090 -2.384119 2.167591
H 6.147525 -2.109183 1.371918
H 4.975572 -1.873866 0.049856
H 5.753491 -0.465705 0.808393
H 2.310243 -1.428336 3.767432
H 0.599050 -1.056773 3.368921
H 1.429771 -2.393446 2.559232
H 3.544229 0.835852 -2.553146
H 4.886257 1.015313 -1.416509
H 3.382097 0.250778 -0.875227
H 4.830677 2.683020 0.441244
H 3.394620 3.714840 0.673904
H 3.317402 1.972829 1.050289
H 5.012483 3.444339 -1.954621
H 3.621502 3.311431 -3.052932
H 3.587384 4.494722 -1.724373
H -1.425283 0.800634 -3.789115
H 0.168916 1.525048 -4.147968
H 0.063866 -0.103854 -3.418106
H -2.032251 2.772570 -2.529459
H -1.097695 3.400034 -1.139242
H -0.461006 3.584840 -2.780778

I_1

60
Energy: -4044.82527265
Cu 0.209802 -0.087542 0.130794
Cl 0.389649 0.253643 2.225861
C 2.089020 0.147393 0.264561
O -1.818381 0.093545 0.038530
S -1.975652 1.449373 -0.612266
O -2.779318 1.445217 -1.823386
C -2.937280 2.380861 0.640815
O -0.657161 2.108672 -0.665039
F -3.134964 3.614724 0.203776
F -4.106487 1.788510 0.829210
F -2.274879 2.418862 1.782067
C 2.954757 -0.920837 0.379740
C 4.311483 -0.655643 0.181516
C 4.750888 0.638627 -0.085828
C 3.841363 1.689236 -0.153076
C 2.480048 1.456063 0.046699
H 2.588191 -1.919706 0.609652
H 5.025264 -1.475567 0.249947
H 5.813317 0.831872 -0.224677
H 4.182457 2.705327 -0.345893
H 1.757648 2.271015 0.026447
C -0.492072 -3.031524 0.853467
O 0.717721 -3.072364 0.653709
C -1.118166 -3.379906 2.205011
N -1.361520 -2.753086 -0.163891
C -0.909740 -2.420746 -1.511268
C 0.023041 -1.247783 -1.552373
C 0.690732 -0.432255 -2.196902
C 1.520582 0.482756 -2.889612
C 1.023492 1.740084 -3.271769
C 1.859485 2.631330 -3.926802
C 3.176615 2.275400 -4.211078
C 3.668910 1.023249 -3.846074
C 2.846455 0.125575 -3.184404
H -2.284015 -2.413458 0.081823
C -0.190176 -3.614332 -2.144248
C -2.146813 -2.066720 -2.337666
H -0.002546 2.002560 -3.021964
H 1.483554 3.610179 -4.216702
H 3.827223 2.981776 -4.724345
H 4.698884 0.753043 -4.070163

H 3.216306 -0.847997 -2.867155
C -2.290201 -2.461283 2.557473
C -1.605506 -4.831063 2.102273
C -0.044776 -3.281959 3.284154
H -0.470597 -3.578161 4.252541
H 0.801309 -3.940567 3.057796
H 0.338756 -2.257265 3.370017
H -2.022503 -5.149994 3.067698
H -2.386345 -4.945837 1.338357
H -0.776080 -5.506487 1.851033
H -2.626063 -2.681011 3.579899
H -2.004603 -1.401362 2.515324
H -3.163114 -2.614172 1.905628
H -1.855599 -1.858297 -3.373449
H -2.836733 -2.921552 -2.334970
H -2.656555 -1.180330 -1.942219
H 0.084501 -3.377599 -3.179755
H 0.710638 -3.873159 -1.582240
H -0.871878 -4.474655 -2.147664

TS_{rc}^{A}

60
Energy: -4044.80999661
Cu -0.572852 -0.124342 -1.080059
Cl -0.302977 -0.540643 -3.158680
C -2.245907 -0.967785 -1.262260
O 0.933329 1.130136 -0.962197
S 1.773190 1.639255 0.180707
O 2.540358 0.580459 0.850362
C 3.030161 2.593831 -0.753420
O 1.097009 2.601572 1.041370
F 3.902284 3.106375 0.103552
F 3.671741 1.799596 -1.598506
F 2.453082 3.575044 -1.426067
C -2.409723 -2.289601 -0.889161
C -3.699700 -2.820150 -0.895112
C -4.783381 -2.035292 -1.277653
C -4.583468 -0.715578 -1.668591
C -3.300106 -0.170160 -1.673581
H -1.556262 -2.895058 -0.588678
H -3.851648 -3.856826 -0.596874
H -5.786765 -2.457642 -1.281071
H -5.425331 -0.100521 -1.982824
H -3.140667 0.860054 -1.991266
C 1.616089 -2.037600 0.713034
O 0.552010 -1.923164 0.073272
C 2.889399 -2.532577 0.064293
N 1.552559 -1.816463 2.041729
C 0.265513 -1.320634 2.537069
C -0.441425 -0.635763 1.434097
C -1.120208 0.198256 0.782533
C -2.131620 1.215751 1.033913
C -2.039869 2.493766 0.476804
C -3.058089 3.412541 0.697112
C -4.175643 3.060911 1.449342
C -4.274097 1.782575 1.991383
C -3.255150 0.861057 1.788712
H 2.395586 -1.526949 2.524708
C -0.576072 -2.491419 3.042867
C 0.496593 -0.265860 3.620527
H -1.157566 2.764710 -0.099385
H -2.977454 4.411233 0.272236
H -4.973812 3.783535 1.609763
H -5.149330 1.499633 2.573591
H -3.334034 -0.154337 2.179176
C 3.042071 -1.876210 -1.307549
C 4.121293 -2.259670 0.922028
C 2.696527 -4.047842 -0.102810
H 3.588232 -4.474727 -0.580749
H 2.558146 -4.546042 0.867432
H 1.828742 -4.266673 -0.737340
H 3.923009 -2.299053 -1.809122
H 2.164804 -2.053571 -1.941453
H 3.181312 -0.792666 -1.214774
H 5.012406 -2.622231 0.393762
H 4.250862 -1.183967 1.098393
H 4.084865 -2.791708 1.884433
H -0.462022 0.159546 3.943396
H 0.973129 -0.730812 4.494271
H 1.125981 0.546342 3.235435
H -1.551260 -2.135543 3.399940

H -0.743495 -3.217028 2.237871
H -0.057799 -2.992129 3.870270

TS_{ar}^{B}

60
Energy: -4044.81704073
Cu -0.252193 -0.505463 -0.665365
Cl 0.078926 -0.708928 -2.799398
C -2.065522 -1.171934 -0.859960
O 1.487082 0.421308 -0.442176
S 1.724731 1.817314 0.076980
O 2.588374 1.814399 1.260996
C 2.756962 2.505411 -1.273083
O 0.515285 2.633733 0.123018
F 3.110880 3.743373 -0.963012
F 3.846084 1.762917 -1.416512
F 2.075637 2.512966 -2.402698
C -2.287832 -2.454690 -0.379792
C -3.371637 -3.174321 -0.882309
C -4.217614 -2.599414 -1.824204
C -3.983931 -1.301706 -2.277577
C -2.900694 -0.576907 -1.798637
H -1.620190 -2.892620 0.359001
H -3.552190 -4.186486 -0.524528
H -5.067136 -3.162554 -2.206259
H -4.638524 -0.852791 -3.022339
H -2.706548 0.433562 -2.153153
C 1.798373 -1.816880 0.925318
O 0.822292 -2.300744 0.332963
C 3.219702 -2.179341 0.525638
N 1.613019 -1.011754 2.005438
C 0.260585 -0.637012 2.396782
C -0.616956 -0.271913 1.240066
C -1.726731 0.116393 0.792892
C -2.863315 1.004093 0.851355
C -2.611967 2.379542 0.938330
C -3.680593 3.257992 1.074690
C -4.982832 2.771766 1.129691
C -5.229104 1.401325 1.042818
C -4.174523 0.514210 0.894090
H 2.331928 -0.312172 2.180250
C -0.398808 -1.807793 3.133910
C 0.321659 0.586966 3.314224
H -1.582943 2.734328 0.880978
H -3.492001 4.327850 1.136067
H -5.816063 3.464077 1.238079
H -6.249545 1.026008 1.087929
H -4.350995 -0.557885 0.815773
C 4.274501 -1.254739 1.126889
C 3.430879 -3.607940 1.048231
C 3.324391 -2.168338 -0.999273
H 4.437951 -3.952247 0.776367
H 3.340458 -3.651594 2.143266
H 2.699204 -4.297502 0.609070
H 4.313764 -2.542607 -1.296061
H 2.557249 -2.804092 -1.455345
H 3.199120 -1.154602 -1.398904
H 5.266227 -1.570765 0.777367
H 4.132800 -0.209310 0.818789
H 4.292973 -1.302239 2.225478
H -0.691678 0.865196 3.629202
H 0.906502 0.336410 4.209636
H 0.787622 1.444760 2.815683
H -1.426885 -1.551831 3.419313
H -0.413793 -2.706502 2.509708
H 0.180099 -2.020425 4.041281

I_2

60
Energy: -4044.86166906
Cu -0.689562 0.048583 0.877805
Cl -2.243595 1.048311 2.103905
C 1.971020 0.527872 1.489923
O -0.239716 1.610248 -0.079126
S -0.698052 1.537416 -1.533048
O -1.640486 0.429990 -1.724698
C -1.701014 3.070440 -1.646041
O 0.394837 1.684265 -2.482148
F -2.189693 3.160982 -2.875200
F -2.691885 3.021015 -0.780345

F -0.939289 4.121568 -1.399408
C 1.192991 0.327565 2.638176
C 1.150060 1.297173 3.638525
C 1.895576 2.459426 3.507650
C 2.700122 2.647450 2.382247
C 2.742184 1.689166 1.381888
H 0.679354 -0.624524 2.787752
H 0.521358 1.137943 4.512044
H 1.858004 3.221839 4.283314
H 3.288773 3.557723 2.281945
H 3.345843 1.848319 0.489071
C -1.850253 -2.205677 0.258049
O -1.389373 -1.639740 1.293971
C -3.300817 -2.644558 0.252782
N -1.102535 -2.361340 -0.821439
C 0.342385 -2.059808 -0.911284
C 0.718977 -0.905079 -0.003924
C 1.915355 -0.453162 0.382060
C 3.210167 -0.806430 -0.262250
C 3.519947 -0.287070 -1.522994
C 4.758774 -0.554204 -2.099618
C 5.689914 -1.338134 -1.425366
C 5.390198 -1.843649 -0.162289
C 4.159877 -1.568965 0.422872
H -1.534950 -2.733820 -1.658758
C 1.076174 -3.317332 -0.418602
C 0.640842 -1.814512 -2.388991
H 2.787852 0.344350 -2.030856
H 4.997624 -0.141645 -3.078348
H 6.656262 -1.548794 -1.880388
H 6.121570 -2.447734 0.372080
H 3.921239 -1.947111 1.417757
C -3.578369 -3.747233 -0.765490
C -3.679980 -3.116657 1.656120
C -4.105195 -1.382091 -0.103051
H -5.174132 -1.635343 -0.112395
H -3.937155 -0.584352 0.633104
H -3.826115 -0.992876 -1.091456
H -4.747565 -3.371379 1.671253
H -3.112578 -4.010069 1.951072
H -3.498788 -2.329287 2.395640
H -4.620042 -4.075511 -0.660009
H -3.470464 -3.402866 -1.804768
H -2.936095 -4.625764 -0.612355
H 2.160262 -3.190483 -0.516061
H 0.839274 -3.514554 0.634130
H 0.772279 -4.183906 -1.021569
H 1.719553 -1.715759 -2.542666
H 0.302436 -2.679872 -2.979348
H 0.143307 -0.911373 -2.755836

TS_{rc}^{B}

60
Energy: -4044.84033862
Cu -0.243077 -0.410497 0.628793
Cl -0.701835 -1.035745 2.660250
C 2.321745 -1.047361 0.936721
O -0.250719 2.140027 0.952612
S -1.480411 2.094602 0.148005
O -1.824831 0.666925 -0.202473
C -2.838105 2.547828 1.291941
O -1.580710 3.010180 -0.982877
F -3.996332 2.473455 0.648769
F -2.856659 1.722187 2.319644
F -2.657165 3.787548 1.719690
C 2.251513 -2.442897 0.893436
C 2.710539 -3.188383 1.970952
C 3.244445 -2.550604 3.086153
C 3.327738 -1.160619 3.123638
C 2.879074 -0.408448 2.049293
H 1.819264 -2.934664 0.023814
H 2.648904 -4.274388 1.938945
H 3.595125 -3.138670 3.932245
H 3.737594 -0.660021 3.998412
H 2.920085 0.680411 2.074300
C -1.416801 -1.902301 -1.350995
O -0.551291 -2.235759 -0.505341
C -2.817205 -2.473738 -1.280658
N -1.068903 -1.054377 -2.324074
C 0.286064 -0.492237 -2.324299
C 0.766390 -0.402830 -0.906313

C 1.914972 -0.229761 -0.239377
C 2.860330 0.816270 -0.722197
C 2.461788 2.146405 -0.877506
C 3.378922 3.086722 -1.338886
C 4.680005 2.706029 -1.648490
C 5.078497 1.380222 -1.484455
C 4.176008 0.436794 -1.013295
H -1.790771 -0.560816 -2.835692
C 1.217510 -1.431881 -3.095446
C 0.226852 0.880139 -2.999341
H 1.449706 2.442685 -0.601220
H 3.070836 4.124756 -1.447295
H 5.392362 3.445814 -2.009537
H 6.099371 1.082476 -1.716998
H 4.480236 -0.599417 -0.864751
C -3.765484 -1.807472 -2.271866
C -2.692531 -3.969942 -1.592818
C -3.334831 -2.277022 0.148260
H -4.320819 -2.752625 0.237134
H -2.659290 -2.722100 0.888107
H -3.437249 -1.211481 0.391895
H -3.682921 -4.438291 -1.520723
H -2.309499 -4.139726 -2.609263
H -2.023827 -4.464693 -0.878571
H -4.763625 -2.250888 -2.166491
H -3.866782 -0.731157 -2.070243
H -3.455703 -1.959281 -3.316373
H 2.241626 -1.036840 -3.086509
H 1.222291 -2.431323 -2.644667
H 0.880611 -1.516068 -4.136459
H 1.228938 1.315723 -3.067631
H -0.150236 0.743220 -4.022945
H -0.429715 1.577276 -2.463303

Reaction 35

reactant

29
Energy: -520.845971852
C -0.000141 0.004386 -0.000723
O 0.001657 0.000285 1.222503
C -1.295746 -0.199796 -0.793895
N 1.150260 0.190697 -0.701431
H 1.168862 0.198340 -1.710545
C 2.404124 0.389231 0.009997
C 3.508020 0.557822 -0.922639
H 2.310294 1.263661 0.672485
H 2.579568 -0.467936 0.678296
C 4.415581 0.704281 -1.708320
C 5.526718 0.845597 -2.642445
C -1.118118 -0.167780 -2.308496
C -1.864047 -1.556476 -0.373900
C -2.256710 0.911440 -0.367690
H -2.090186 -0.321595 -2.796456
H -0.732826 0.800842 -2.659642
H -0.450912 -0.966810 -2.663926
H -3.239958 0.761907 -0.835639
H -2.380184 0.913836 0.721211
H -1.882957 1.899502 -0.671996
H -2.845338 -1.717347 -0.842186
H -1.202121 -2.378164 -0.682475
H -1.980162 -1.602449 0.714840
C 6.249414 -0.475037 -2.900400
H 5.155207 1.260725 -3.591636
H 6.236343 1.590415 -2.251635
H 7.078684 -0.334230 -3.603830
H 6.655269 -0.885613 -1.968449
H 5.563982 -1.219734 -3.321968

I_c

64
Energy: -4121.47142170
Cu 0.712734 0.098132 -0.554431
Cl 1.346309 -0.461769 -2.574007
C 1.954244 1.536843 -0.762575
O -0.576228 -1.442359 -0.406417
S -1.397784 -1.856287 0.789202
O -2.123379 -0.741297 1.407091
C -2.709065 -2.821595 -0.054204
O -0.738087 -2.801853 1.677179

F -3.580390 -3.264543 0.835776
F -3.358091 -2.041310 -0.927452
F -2.188784 -3.843528 -0.708980
C 1.377799 2.755550 -0.466309
C 2.230215 3.858495 -0.347421
C 3.596581 3.723963 -0.547372
C 4.132857 2.479971 -0.868935
C 3.307546 1.362792 -0.983895
C -1.089682 3.007453 2.112922
O -0.228793 3.878213 2.034685
C -2.573940 3.373819 2.073883
N -0.770496 1.690454 2.274956
C 0.605761 1.303519 2.420321
C 1.091180 0.235990 1.529968
C 1.793233 -0.700030 1.142129
C 2.730938 -1.766398 0.805117
H 0.314021 2.883544 -0.287931
H 1.791025 4.814247 -0.066720
H 4.248968 4.590244 -0.455458
H 5.201319 2.366896 -1.046049
H 3.719243 0.399666 -1.275874
H -1.464650 0.951914 2.178636
H 1.205574 2.211139 2.252428
H 0.812859 0.958773 3.446673
C -3.513591 2.183719 2.228389
C -2.807510 4.361368 3.220449
C -2.829093 4.074391 0.738072
O -3.266742 1.170297 -1.032082
C -4.491009 0.734066 -1.642833
C -5.513244 0.657805 -0.540176
C -2.073713 0.910943 -1.548280
O -1.099534 1.251974 -0.889022
C -1.994463 0.244900 -2.883162
H -4.770916 1.459373 -2.420205
H -4.347978 -0.245742 -2.112746
H -6.480601 0.330022 -0.937541
H -5.181602 -0.058058 0.222639
H -5.642909 1.636913 -0.063352
H -2.741953 0.639215 -3.580170
H -0.989930 0.369192 -3.291878
H -2.170832 -0.831261 -2.755982
H -3.367890 1.667195 3.187919
H -4.553590 2.540208 2.207580
H -3.394260 1.453392 1.417490
H -3.875205 4.410093 0.684608
H -2.175035 4.948053 0.630041
H -2.645794 3.391876 -0.101998
H -3.845379 4.722477 3.195829
H -2.639552 3.884196 4.196522
H -2.132225 5.220562 3.137704
H 3.232411 -2.017202 1.754106
C 2.143639 -3.025187 0.173287
H 3.513967 -1.338627 0.157620
H 2.945247 -3.758797 0.032902
H 1.366280 -3.455258 0.811008
H 1.699395 -2.803250 -0.803488

I_1

50
Energy: -3813.94966323
Cu 0.027077 0.053566 -0.047513
Cl -0.130049 0.405479 2.045516
C 1.872152 0.122293 0.368548
O -1.952903 0.218099 -0.453101
S -1.954059 1.495959 -1.266252
O -2.574614 1.388424 -2.572819
C -3.025607 2.594878 -0.263343
O -0.601420 2.088519 -1.181334
F -3.107011 3.776487 -0.852624
F -4.233571 2.064408 -0.167792
F -2.510497 2.738900 0.944472
C 2.616354 -1.039339 0.439334
C 4.005248 -0.900313 0.423895
C 4.593374 0.360446 0.367156
C 3.801700 1.503553 0.339835
C 2.410048 1.396340 0.363620
H 2.153796 -2.027359 0.466807
H 4.623658 -1.796049 0.450403
H 5.677913 0.452230 0.361192
H 4.257864 2.491969 0.320983
H 1.777225 2.282426 0.378897

C -0.369969 -3.290761 0.762348
O 0.687009 -3.695385 0.291223
C -0.764782 -3.569124 2.209992
N -1.258920 -2.615361 -0.027308
C -0.948052 -2.397529 -1.421560
C 0.004082 -1.299159 -1.665631
C 0.743340 -0.431570 -2.116467
C 1.608147 0.525887 -2.797986
H -2.059210 -2.132454 0.361721
H -0.514188 -3.318423 -1.831843
H -1.878283 -2.170737 -1.955636
C -2.015810 -2.823783 2.665682
C -1.007718 -5.080306 2.294787
C 0.422980 -3.188637 3.094949
H 0.209130 -3.455456 4.138995
H 1.327413 -3.720140 2.776504
H 0.620302 -2.108115 3.052608
H -1.241441 -5.361375 3.330910
H -1.853873 -5.384669 1.662083
H -0.118550 -5.635810 1.973291
H -2.209075 -3.052225 3.722227
H -1.899801 -1.733149 2.585124
H -2.909896 -3.135294 2.105622
C 1.138597 0.718073 -4.240057
H 1.562214 1.485524 -2.267056
H 2.650098 0.175548 -2.744807
H 1.782453 1.443716 -4.748879
H 1.169534 -0.222626 -4.801819
H 0.109359 1.096440 -4.254273

TS_{rc}^{A}

50
Energy: -3813.93058960
Cu 0.621732 -0.872893 -0.720838
Cl 1.522475 -2.690483 -0.035600
C 2.352318 -0.265212 -1.138141
O -1.165710 -1.319419 -0.014881
S -2.589928 -0.882212 -0.228718
O -3.274027 -1.658690 -1.266186
C -3.328107 -1.426033 1.358437
O -2.789899 0.566310 -0.267320
F -4.617411 -1.120573 1.363322
F -3.188107 -2.731299 1.508534
F -2.730820 -0.800037 2.363116
C 2.809825 -0.344912 -2.442586
C 4.085295 0.139397 -2.730705
C 4.876473 0.687917 -1.726679
C 4.398064 0.746071 -0.422425
C 3.128217 0.257779 -0.115361
H 2.186771 -0.768083 -3.227534
H 4.456719 0.085317 -3.753232
H 5.871638 1.062583 -1.959453
H 5.017648 1.156552 0.373593
H 2.776612 0.266272 0.915531
C -1.234809 -1.567123 -3.457750
O -0.106990 -1.242157 -3.033589
C -1.599328 -3.005929 -3.746705
N -2.077496 -0.555340 -3.746752
C -1.542271 0.770002 -3.476495
C -0.550222 0.703847 -2.396256
C 0.014177 0.882989 -1.282103
C 0.144811 2.006962 -0.303933
H -3.077506 -0.716157 -3.714436
H -1.108110 1.189640 -4.395571
H -2.352289 1.427892 -3.131908
C -1.195880 -3.874672 -2.554314
C -3.082334 -3.183567 -4.057614
C -0.763754 -3.391292 -4.977824
H -0.974983 -4.436410 -5.239663
H -1.013214 -2.766358 -5.847317
H 0.309529 -3.297647 -4.773068
H -3.273893 -4.242338 -4.274023
H -3.706598 -2.900099 -3.200719
H -3.389729 -2.614546 -4.947550
H -1.386655 -4.927330 -2.802971
H -0.131717 -3.763717 -2.311811
H -1.778537 -3.614771 -1.662427
H -0.563812 2.803715 -0.568036
C -0.052991 1.596524 1.148554
H 1.160017 2.411667 -0.441483
H 0.132831 2.454402 1.805294

H -1.074025 1.241844 1.318976
H 0.637837 0.794191 1.443466

TS_{ar}^{B}

50
Energy: -3813.94034868
Cu 0.279876 -0.850868 -0.997442
Cl 1.016116 -2.324656 0.408539
C 2.004091 0.050306 -1.099861
O -1.568195 -1.556782 -0.857657
S -2.811479 -0.825021 -0.409040
O -3.732575 -0.585322 -1.526541
C -3.598301 -2.143541 0.593613
O -2.547984 0.280501 0.501374
F -4.762189 -1.710387 1.051651
F -3.800899 -3.216536 -0.163330
F -2.816873 -2.464970 1.606405
C 2.607737 0.018143 -2.351738
C 3.988385 0.177427 -2.438567
C 4.741957 0.400867 -1.292528
C 4.117805 0.449516 -0.048195
C 2.744792 0.264987 0.059247
H 2.014587 -0.145940 -3.247920
H 4.467526 0.138703 -3.415102
H 5.818874 0.539354 -1.366603
H 4.703696 0.605797 0.855673
H 2.271834 0.257706 1.037561
C -0.936158 -1.950666 -3.402802
O 0.257428 -1.776706 -3.119246
C -1.508351 -3.338963 -3.628051
N -1.746152 -0.875762 -3.592889
C -1.199385 0.433797 -3.342637
C -0.426088 0.628917 -2.092884
C 0.314240 1.226976 -1.268526
C 0.556423 2.399659 -0.396480
H -2.729207 -0.985971 -3.347995
H -0.524042 0.709397 -4.166950
H -2.031694 1.150714 -3.339995
C -3.032211 -3.357099 -3.708541
C -0.918457 -3.807979 -4.965822
C -1.031937 -4.259066 -2.503256
H -3.368698 -4.390799 -3.862371
H -3.501448 -2.995806 -2.782871
H -3.410320 -2.765283 -4.554316
H -1.340169 -5.289786 -2.725879
H 0.059318 -4.234075 -2.403831
H -1.464912 -3.964668 -1.539380
H 0.053338 3.245681 -0.887465
C -0.006992 2.207497 1.008900
H 1.630421 2.624982 -0.373401
H 0.211084 3.090485 1.620002
H -1.088557 2.045566 0.976012
H 0.430228 1.331458 1.504726
H -1.272648 -4.824379 -5.184817
H -1.230450 -3.154025 -5.792857
H 0.177618 -3.823841 -4.926473

I_2

50
Energy: -3813.99111441
Cu 0.278676 -1.360679 -1.928694
Cl 0.975719 -3.408240 -2.383432
C 2.042074 0.430490 -0.670811
O -0.173390 -1.659012 -0.123878
S -1.694755 -1.763736 0.013435
O -2.318469 -1.882869 -1.310086
C -1.858814 -3.412095 0.804423
O -2.247144 -0.795089 0.944584
F -3.147145 -3.649126 1.007080
F -1.357230 -4.342037 0.020968
F -1.225455 -3.410092 1.964399
C 2.699894 -0.399226 -1.587842
C 3.767804 -1.196334 -1.180036
C 4.197471 -1.157435 0.138435
C 3.564516 -0.312091 1.050275
C 2.495531 0.476844 0.651079
H 2.421523 -0.370734 -2.642901
H 4.249195 -1.854585 -1.900120
H 5.025485 -1.785022 0.462000
H 3.898902 -0.281960 2.085841

H 1.979295 1.102963 1.378208
C -1.128899 -0.653219 -4.100477
O 0.037281 -0.981410 -3.753476
C -1.735319 -1.370648 -5.290750
N -1.853889 0.239140 -3.431716
C -1.466789 0.880149 -2.178743
C -0.125156 0.484273 -1.696488
C 0.833422 1.179374 -1.093063
C 0.777764 2.658511 -0.800411
H -2.818895 0.356168 -3.714090
H -1.472795 1.969702 -2.342578
H -2.242698 0.646770 -1.434168
C -2.932271 -0.639383 -5.891161
C -0.656421 -1.572512 -6.354138
C -2.173244 -2.735253 -4.729523
H -2.603085 -3.334653 -5.543482
H -1.320508 -3.278561 -4.300535
H -2.929397 -2.621265 -3.940624
H -1.073261 -2.162002 -7.180928
H -0.305606 -0.614800 -6.762434
H 0.203250 -2.111620 -5.941927
H -3.268774 -1.180707 -6.784188
H -3.797094 -0.608258 -5.211390
H -2.681952 0.385004 -6.200808
H -0.181529 3.080700 -1.128594
H 0.803043 2.793963 0.292654
C 1.937437 3.412266 -1.439524
H 1.881014 4.483787 -1.213688
H 2.901446 3.039343 -1.071734
H 1.929683 3.292830 -2.530805

TS_{rc}^{B}

50
Energy: -3813.96372725
Cu 0.121158 -1.293776 -1.242842
Cl 0.979237 -3.208837 -1.890362
C 2.100779 0.378211 -1.216592
O -0.963481 -1.158114 0.384171
S -2.400720 -1.541802 0.111616
O -2.611858 -1.799135 -1.321670
C -2.514374 -3.184421 0.913551
O -3.362452 -0.679451 0.781026
F -3.734040 -3.672280 0.738991
F -1.631690 -4.004774 0.375380
F -2.273352 -3.065007 2.209540
C 3.062270 0.342597 -2.228761
C 4.191461 -0.453288 -2.085847
C 4.375548 -1.207804 -0.929059
C 3.431653 -1.165458 0.088759
C 2.291466 -0.380929 -0.054136
H 2.901138 0.920980 -3.138660
H 4.929375 -0.492567 -2.885007
H 5.257560 -1.836987 -0.827051
H 3.566162 -1.763032 0.987703
H 1.555888 -0.328114 0.750750
C -1.144720 -0.548910 -3.762688
O 0.010417 -0.522362 -3.231531
C -1.435670 -1.479039 -4.910010
N -2.051232 0.262070 -3.239393
C -1.626296 0.988189 -2.053025
C -0.230430 0.543914 -1.766538
C 0.852123 1.190447 -1.328850
C 0.821741 2.576406 -0.743648
H -3.035470 0.146739 -3.447115
H -1.648410 2.070078 -2.253192
H -2.311593 0.774024 -1.221545
C -2.550565 -0.926063 -5.795540
C -0.157550 -1.685257 -5.722598
C -1.869549 -2.815544 -4.281663
H -2.081401 -3.520926 -5.096520
H -1.076955 -3.235591 -3.646770
H -2.770934 -2.700659 -3.667208
H -0.367047 -2.390449 -6.536821
H 0.198147 -0.745955 -6.167479
H 0.640934 -2.103444 -5.099202
H -2.689111 -1.599849 -6.650073
H -3.518664 -0.880329 -5.276258
H -2.312592 0.071561 -6.188922
H -0.174890 3.023176 -0.867849
H 0.985831 2.498249 0.343209
C 1.889489 3.477827 -1.354820

H 1.857607 4.474937 -0.899914
H 2.893533 3.066083 -1.194687
H 1.741970 3.592284 -2.436709

Reaction 36

reactant

35
Energy: -877.561793565
C -3.979904 -0.380091 0.198096
O -4.696878 -1.018335 -0.560601
C -4.538883 0.305145 1.449280
N -2.644724 -0.264962 -0.031269
H -2.028529 0.241899 0.591527
C -2.044310 -0.927657 -1.175694
C -0.605430 -0.749795 -1.093073
H -2.323110 -1.992927 -1.178893
H -2.447092 -0.515348 -2.114503
C 0.529187 -0.457610 -0.788029
C 1.875216 -0.142963 -0.471784
C 2.884418 -0.525161 -1.370910
C 4.219098 -0.254181 -1.116701
C 4.593827 0.406207 0.052106
C 3.622481 0.789972 0.962032
C 2.282690 0.521634 0.701608
H 2.586539 -1.043923 -2.279481
H 4.975065 -0.561599 -1.836632
H 5.640432 0.621152 0.254525
H 3.876976 1.301902 1.884967
N 1.312037 0.964689 1.713120
C -3.511935 1.124153 2.226248
C -5.681600 1.212985 0.993080
C -5.094025 -0.805675 2.343742
H -3.996679 1.589638 3.095188
H -2.685081 0.509573 2.611211
H -3.084774 1.936078 1.619907
H -5.585669 -0.370467 3.225206
H -5.824805 -1.411711 1.795475
H -4.291966 -1.469568 2.696647
H -6.185611 1.652339 1.865381
H -5.309799 2.036358 0.366893
H -6.414797 0.644665 0.409688
O 1.757046 1.407554 2.759383
O 0.120281 0.873210 1.458305

I_c

70
Energy: -4478.17554702
Cu 0.336355 -0.337724 -0.423007
Cl 0.893586 -1.238710 -2.336878
C 1.712569 0.969853 -0.749615
O -1.213327 -1.586112 -0.229757
S -2.261134 -1.822212 0.822060
O -3.211815 -0.703057 0.926385
C -3.211705 -3.173226 0.025084
O -1.765999 -2.379466 2.073065
F -4.207985 -3.531182 0.818863
F -3.726183 -2.746626 -1.132780
F -2.434058 -4.209895 -0.206359
C 1.324773 2.167960 -0.181392
C 2.258132 3.205334 -0.148295
C 3.525275 3.032611 -0.692099
C 3.870750 1.816985 -1.270553
C 2.960645 0.757804 -1.301123
C -1.022038 2.579548 2.369883
O 0.087721 2.976132 2.707855
C -2.149427 3.558543 2.035425
N -1.293687 1.246435 2.260532
C -0.326444 0.277200 2.696003
C 0.612039 -0.272264 1.688644
C 1.548674 -0.964573 1.280608
C 2.822904 -1.520957 0.959554
C 3.039761 -2.753049 0.321315
C 4.312751 -3.176223 -0.020124
C 5.409374 -2.380743 0.294029
C 5.225053 -1.161340 0.937543
C 3.944099 -0.730846 1.254269
H 0.343638 2.320554 0.255215
H 1.974800 4.137701 0.337774
H 4.245941 3.847986 -0.664311

H 4.856140 1.672574 -1.711995
H 3.237848 -0.192199 -1.750157
H -2.180704 0.897844 1.903205
H 0.297279 0.752856 3.465233
H -0.849474 -0.585754 3.129404
N 1.919906 -3.660263 0.008260
H 4.422530 -4.130440 -0.526729
H 6.409810 -2.718387 0.032447
H 6.079362 -0.533335 1.180726
H 3.778821 0.242501 1.714271
C -3.518493 2.896836 1.925747
C -2.173763 4.619531 3.135540
C -1.786466 4.213891 0.698856
O -3.399795 1.354479 -1.106360
C -4.669114 0.822048 -1.527109
C -5.703889 1.292756 -0.540770
C -2.268871 0.814789 -1.537902
O -1.241096 1.099001 -0.931944
C -2.304499 -0.071508 -2.739532
H -4.885853 1.175983 -2.545377
H -4.608388 -0.273698 -1.532381
H -6.696578 0.944277 -0.848824
H -5.485474 0.888288 0.454317
H -5.723197 2.387689 -0.483350
H -3.054758 0.251133 -3.468405
H -1.312907 -0.095548 -3.196493
H -2.536901 -1.096471 -2.423892
H -3.802301 2.386112 2.856952
H -4.279524 3.664332 1.724255
H -3.559238 2.176661 1.098070
H -2.540779 4.969170 0.436187
H -0.810557 4.713557 0.765228
H -1.750603 3.470722 -0.110369
H -2.910817 5.395923 2.887262
H -2.455755 4.182976 4.103903
H -1.189571 5.087254 3.247838
O 2.142810 -4.560820 -0.781033
O 0.864699 -3.456430 0.577265

I_1

56
Energy: -4170.65704994
Cu 0.035697 0.142335 0.029523
Cl 0.011806 0.116766 2.165964
C 1.881960 0.035634 0.420013
O -1.940125 0.537122 -0.226165
S -1.840390 1.951750 -0.757762
O -2.702079 2.271434 -1.873756
C -2.453289 2.976500 0.638117
O -0.392145 2.266910 -0.860416
F -2.406823 4.250176 0.282951
F -3.701810 2.638323 0.913163
F -1.703315 2.796890 1.711537
C 2.561341 -1.168178 0.437182
C 3.956030 -1.106416 0.429238
C 4.613791 0.122792 0.443546
C 3.885974 1.307934 0.467110
C 2.490053 1.276628 0.475672
H 2.036794 -2.125046 0.432797
H 4.524530 -2.035078 0.420579
H 5.701909 0.153784 0.457564
H 4.395401 2.269776 0.498743
H 1.906390 2.194261 0.530378
C -0.623461 -3.239057 0.628732
O 0.424394 -3.623325 0.120307
C -1.048051 -3.673327 2.029001
N -1.490095 -2.464695 -0.090470
C -1.171299 -2.127902 -1.456013
C -0.141214 -1.085115 -1.638235
C 0.566140 -0.290312 -2.250848
C 1.428916 0.620980 -2.918960
C 0.961021 1.643094 -3.764624
C 1.822900 2.558809 -4.339201
C 3.189191 2.462598 -4.093470
C 3.679826 1.449450 -3.278283
C 2.808669 0.537379 -2.696213
H -2.260101 -1.980369 0.353276
H -0.819879 -3.031811 -1.970275
H -2.081052 -1.767880 -1.952649
N -0.463647 1.737880 -4.124378
H 1.407930 3.332368 -4.978692

H 3.868428 3.179031 -4.550230
H 4.747526 1.365219 -3.085386
H 3.184511 -0.251144 -2.046787
C -2.229693 -2.885859 2.588027
C -1.434673 -5.152170 1.900930
C 0.160035 -3.538370 2.954972
O -0.849707 2.795567 -4.583767
O -1.144753 0.737748 -3.973245
H -0.086265 -3.938308 3.948147
H 1.015874 -4.095190 2.556233
H 0.454863 -2.486539 3.072567
H -1.713653 -5.549823 2.886509
H -2.293410 -5.282981 1.226902
H -0.595895 -5.741643 1.510922
H -2.437142 -3.220424 3.613151
H -2.022915 -1.807126 2.630774
H -3.149953 -3.053209 2.009182

TS_{rc}^{A}

56
Energy: -4170.64708126
Cu 0.333657 0.622195 -0.728476
Cl -0.205408 1.268354 -2.697335
C 1.807987 1.785172 -0.932005
O -1.396440 -0.381610 -0.376412
S -1.680623 -1.690089 0.316744
O -2.830020 -1.586467 1.225167
C -2.263841 -2.723494 -1.078735
O -0.494913 -2.361508 0.845600
F -2.611432 -3.918230 -0.626073
F -3.313588 -2.148349 -1.645940
F -1.294187 -2.852961 -1.965166
C 1.715653 3.079241 -0.449699
C 2.851348 3.886293 -0.517113
C 4.033198 3.399756 -1.066815
C 4.085761 2.101098 -1.561620
C 2.960782 1.278512 -1.506089
H 0.785584 3.454563 -0.028378
H 2.801368 4.906211 -0.137899
H 4.912441 4.039353 -1.120943
H 5.002127 1.719883 -2.010144
H 2.987853 0.273356 -1.923070
C -2.056989 1.749696 1.292184
O -0.926039 2.165551 0.973750
C -3.322571 2.227031 0.615640
N -2.123328 0.932886 2.371797
C -0.810635 0.660002 2.939031
C 0.203395 0.668407 1.873836
C 1.044440 0.194080 1.056729
C 2.204323 -0.656009 1.264027
C 2.535102 -1.806902 0.531064
C 3.630896 -2.597622 0.856541
C 4.465858 -2.231900 1.900680
C 4.182402 -1.079467 2.625955
C 3.066354 -0.315858 2.316315
H -2.783655 0.153197 2.327425
H -0.579847 1.409572 3.708998
H -0.815420 -0.337904 3.398889
N 1.772141 -2.248000 -0.639968
H 3.813655 -3.493026 0.269428
H 5.332421 -2.842528 2.142226
H 4.828552 -0.773777 3.446339
H 2.844217 0.583751 2.886491
C -3.055903 2.475226 -0.865548
C -4.478134 1.244042 0.786078
C -3.663374 3.554238 1.314204
H -4.570501 3.974414 0.859810
H -3.853482 3.409061 2.386536
H -2.851623 4.283766 1.196009
H -5.361238 1.649107 0.275183
H -4.249816 0.264126 0.347442
H -4.753100 1.102177 1.840876
H -3.959902 2.895843 -1.325805
H -2.230306 3.181482 -1.015969
H -2.796649 1.546112 -1.387618
O 1.226497 -1.387709 -1.330315
O 1.773169 -3.427081 -0.910211

TS_{ar}^{B}

56

Energy: -4170.64343225
Cu -0.000850 0.894355 0.415263
Cl 0.500260 1.628905 2.396960
C -1.685173 1.836375 0.455545
O 1.576727 -0.260757 0.406387
S 1.437808 -1.769121 0.321636
O 2.021142 -2.283018 -0.916575
C 2.588138 -2.262737 1.662525
O 0.115270 -2.228700 0.731825
F 2.659062 -3.583054 1.712957
F 3.795764 -1.769843 1.414214
F 2.156333 -1.798652 2.819318
C -1.800734 2.921145 -0.402944
C -2.718716 3.923655 -0.090859
C -3.510688 3.816815 1.047363
C -3.391452 2.707927 1.885155
C -2.475209 1.705594 1.594066
H -1.175720 2.992647 -1.290893
H -2.813278 4.785461 -0.748897
H -4.229004 4.599718 1.283684
H -4.001363 2.628826 2.783193
H -2.367407 0.844046 2.249796
C 2.133921 1.477895 -1.467853
O 1.234158 2.227673 -1.063501
C 3.596593 1.733944 -1.150209
N 1.819792 0.431577 -2.277941
C 0.426341 0.176392 -2.524036
C -0.486728 0.198001 -1.355039
C -1.616640 0.106438 -0.812713
C -2.890446 -0.568879 -0.667330
C -3.002085 -1.971486 -0.745308
C -4.209528 -2.618555 -0.530880
C -5.359613 -1.877480 -0.295590
C -5.291240 -0.489159 -0.285013
C -4.071659 0.151482 -0.451562
H 2.409469 -0.395651 -2.223513
H 0.034759 0.930540 -3.223124
H 0.327028 -0.812141 -2.989951
N -1.895430 -2.840425 -1.178577
H -4.228137 -3.703923 -0.576065
H -6.308393 -2.386433 -0.141709
H -6.190390 0.104307 -0.135245
H -4.024483 1.236170 -0.422769
C 4.497927 0.552757 -1.498542
C 3.990155 2.950925 -1.999313
C 3.732810 2.073222 0.334472
H 5.532314 0.798183 -1.224461
H 4.220165 -0.354156 -0.942664
H 4.494136 0.328475 -2.574786
H 4.767130 2.381608 0.539769
H 3.059983 2.889873 0.620305
H 3.493013 1.206473 0.963158
H 5.039750 3.209512 -1.804391
H 3.885367 2.741546 -3.073621
H 3.367384 3.818940 -1.750010
O -1.851372 -3.959530 -0.713888
O -1.156405 -2.396357 -2.042914

I_2

56
Energy: -4170.69814398
Cu 0.182240 -1.459101 -1.872185
Cl 1.056348 -3.410478 -2.414466
C 1.855099 0.430359 -0.435811
O -0.293350 -1.863847 -0.102894
S -1.798042 -2.179138 -0.058256
O -2.339360 -2.145241 -1.424338
C -1.793609 -3.947143 0.445085
O -2.498407 -1.468070 0.990481
F -3.054525 -4.336173 0.573754
F -1.196467 -4.688127 -0.462265
F -1.175197 -4.071324 1.605963
C 2.581250 -0.420355 -1.277120
C 3.682322 -1.119581 -0.789920
C 4.079649 -0.953313 0.529914
C 3.380605 -0.079534 1.362951
C 2.276036 0.610389 0.884831
H 2.328825 -0.489389 -2.337079
H 4.218736 -1.797846 -1.450255
H 4.937546 -1.501565 0.914275
H 3.692899 0.053394 2.397152

H 1.713425 1.270407 1.545867
C -1.149661 -0.669646 -4.062907
O 0.013691 -0.976674 -3.679962
C -1.658952 -1.323522 -5.332022
N -1.938493 0.148566 -3.375131
C -1.626756 0.729838 -2.073576
C -0.294773 0.349633 -1.562740
C 0.614268 1.077292 -0.920771
C 0.533030 2.557936 -0.746947
C -0.231631 3.252197 0.197460
C -0.153303 4.633959 0.345901
C 0.677849 5.366544 -0.485590
C 1.458143 4.705133 -1.430923
C 1.396887 3.322970 -1.540234
H -2.893198 0.252003 -3.695471
H -1.669297 1.827484 -2.164290
H -2.408907 0.426404 -1.362063
N -1.140310 2.554549 1.119609
H -0.751658 5.109674 1.118079
H 0.726109 6.448674 -0.387482
H 2.127354 5.267838 -2.078786
H 2.033408 2.799539 -2.253437
C -2.923101 -0.670935 -5.881956
C -0.550518 -1.288218 -6.384631
C -1.948105 -2.779072 -4.926657
H -2.311626 -3.329586 -5.804840
H -1.042921 -3.271404 -4.547498
H -2.713299 -2.830834 -4.139824
H -0.883076 -1.837271 -7.275019
H -0.313877 -0.259362 -6.689175
H 0.362891 -1.760197 -6.007373
H -3.193577 -1.158841 -6.826661
H -3.788416 -0.797099 -5.214064
H -2.782764 0.398601 -6.093843
O -1.266733 3.033988 2.233944
O -1.717698 1.561727 0.711325

TS_{rc}^{B}

56
Energy: -4170.67189718
Cu -1.042910 0.937150 0.766021
Cl -2.953389 2.043144 0.686925
C 0.475228 -2.064769 1.322422
O 0.578339 1.750028 1.300190
S 1.632337 2.267498 0.332611
O 1.721622 1.454299 -0.886241
C 0.857149 3.824912 -0.256891
O 2.847835 2.667828 1.005241
F 1.699872 4.461273 -1.054792
F -0.250609 3.543880 -0.949654
F 0.538106 4.604124 0.758783
C -0.072240 -1.432708 2.445746
C -0.135618 -2.085477 3.666011
C 0.352580 -3.383290 3.791824
C 0.911150 -4.018171 2.686937
C 0.977668 -3.363980 1.464171
H -0.416714 -0.401871 2.376528
H -0.553221 -1.569512 4.528071
H 0.309680 -3.892682 4.752646
H 1.303700 -5.029437 2.777591
H 1.416630 -3.869334 0.604503
C -2.759300 -0.691066 -0.844494
O -2.111712 -0.844776 0.228184
C -4.212523 -1.091891 -0.938300
N -2.083722 -0.275380 -1.914750
C -0.694617 0.122189 -1.703483
C -0.309087 -0.428373 -0.369044
C 0.528808 -1.403336 0.014131
C 1.520306 -1.853326 -1.003355
C 2.881518 -1.527220 -1.016504
C 3.730999 -1.975344 -2.019285
C 3.237850 -2.782600 -3.034562
C 1.890515 -3.127421 -3.045560
C 1.046666 -2.658496 -2.045239
H -2.578702 0.031778 -2.742700
H -0.046294 -0.307422 -2.478405
H -0.594245 1.215004 -1.760676
N 3.485488 -0.695054 0.040792
H 4.773257 -1.672101 -1.984069
H 3.905854 -3.138341 -3.815683
H 1.492473 -3.763081 -3.834236

H -0.012001 -2.923534 -2.050381
C -4.976817 -0.111622 -1.829321
C -4.237791 -2.504201 -1.540359
C -4.823977 -1.106988 0.460290
H -5.277442 -2.852541 -1.594664
H -3.817870 -2.525757 -2.555425
H -3.675789 -3.210745 -0.914832
H -5.878747 -1.401035 0.384832
H -4.311082 -1.823121 1.112740
H -4.760762 -0.114878 0.924805
H -6.043759 -0.366567 -1.806016
H -4.863043 0.919320 -1.468440
H -4.667574 -0.162812 -2.884094
O 2.841119 -0.531568 1.061523
O 4.597894 -0.251248 -0.169942

Reaction 37

reactant

35
Energy: -877.564242625
C -4.055860 -0.060832 0.188502
O -4.629053 -0.197093 -0.882541
C -4.823694 -0.085949 1.513765
N -2.704856 0.108577 0.240121
H -2.216938 0.257180 1.111289
C -1.942695 0.143231 -0.997028
C -0.514019 0.099685 -0.730381
H -2.253155 -0.707099 -1.621892
H -2.198349 1.044829 -1.576550
C 0.673697 0.071621 -0.497030
C 2.073912 0.026081 -0.228612
C 2.740322 -1.205640 -0.131811
C 4.103607 -1.258592 0.129715
C 4.830920 -0.087099 0.300870
C 4.159498 1.124642 0.203005
C 2.801456 1.208551 -0.057478
H 2.168564 -2.121734 -0.266924
H 4.605402 -2.221009 0.200795
H 5.897199 -0.092326 0.506517
N 4.919516 2.371574 0.384232
H 2.319915 2.179643 -0.126496
C -3.952357 0.114742 2.749640
C -5.874126 1.023634 1.441150
C -5.520015 -1.446056 1.593942
H -4.579724 0.082031 3.650556
H -3.196478 -0.677084 2.856931
H -3.447940 1.092326 2.747380
H -6.162598 -1.489119 2.484456
H -6.137761 -1.613831 0.704436
H -4.789108 -2.264256 1.663201
H -6.519199 0.991664 2.330308
H -5.403707 2.016277 1.399431
H -6.497569 0.903584 0.547994
O 4.306573 3.422092 0.296097
O 6.113689 2.272912 0.611821

I_c

70
Energy: -4478.18680655
Cu 0.690807 0.133204 -0.495724
Cl 1.383962 -0.356520 -2.504154
C 1.895138 1.597075 -0.668170
O -0.555217 -1.445778 -0.364445
S -1.406499 -1.815404 0.825890
O -2.178240 -0.689103 1.358821
C -2.647922 -2.884327 0.002774
O -0.731891 -2.683176 1.782794
F -3.555357 -3.271718 0.882434
F -3.266506 -2.197609 -0.965754
F -2.063530 -3.938342 -0.531112
C 1.277220 2.811687 -0.445760
C 2.099825 3.925423 -0.246498
C 3.480618 3.807218 -0.310848
C 4.062521 2.570059 -0.579105
C 3.265854 1.443381 -0.769810
C -0.968678 3.065649 2.181581
O -0.061414 3.886140 2.262119
C -2.419188 3.516092 1.996467
N -0.729168 1.722816 2.244374

C 0.605574 1.250264 2.486982
C 1.101268 0.210766 1.567732
C 1.773525 -0.773222 1.245547
C 2.616941 -1.841683 0.832561
C 2.095283 -3.096161 0.482016
C 2.985193 -4.071534 0.073042
C 4.358413 -3.864380 0.010059
C 4.866540 -2.623797 0.374594
C 4.003993 -1.614140 0.778490
H 0.198097 2.917664 -0.388147
H 1.627209 4.877641 -0.012396
H 4.109297 4.682575 -0.159505
H 5.144595 2.474278 -0.657219
H 3.709458 0.480777 -1.016883
H -1.460903 1.032898 2.087462
H 1.263442 2.130788 2.420858
H 0.704741 0.838913 3.504651
H 1.028161 -3.298658 0.540017
N 2.456541 -5.393436 -0.311847
H 5.001595 -4.675556 -0.318870
H 5.938312 -2.444285 0.339330
H 4.388582 -0.637364 1.068015
C -3.442852 2.394173 2.135720
C -2.693855 4.593046 3.047294
C -2.508316 4.133151 0.597755
O -3.316720 1.017064 -0.992485
C -4.527366 0.510777 -1.577213
C -5.490552 0.273693 -0.443810
C -2.121420 0.830603 -1.532176
O -1.156253 1.228329 -0.890899
C -2.018358 0.169540 -2.868656
H -4.901400 1.256135 -2.293379
H -4.324874 -0.420716 -2.116913
H -6.437142 -0.124376 -0.827165
H -5.062925 -0.448975 0.262223
H -5.696573 1.204631 0.096637
H -2.786994 0.527707 -3.562384
H -1.022442 0.345270 -3.280389
H -2.140015 -0.914496 -2.743373
H -3.390824 1.913948 3.123287
H -4.453222 2.815051 2.030009
H -3.327362 1.622202 1.363870
H -3.514332 4.544289 0.431709
H -1.778024 4.945648 0.487592
H -2.315794 3.376596 -0.174941
H -3.697555 5.014102 2.895140
H -2.653447 4.176756 4.063724
H -1.956570 5.400556 2.980201
O 3.269028 -6.240355 -0.645788
O 1.250911 -5.549397 -0.270372

I_1

56
Energy: -4170.66518723
Cu -0.022333 0.141749 0.013633
Cl 0.009422 0.284523 2.138835
C 1.842630 0.202741 0.328507
O -2.040969 0.352337 -0.249753
S -2.033684 1.725608 -0.884561
O -2.708170 1.821905 -2.163831
C -3.003142 2.729548 0.305567
O -0.646919 2.242826 -0.794894
F -3.020696 3.986054 -0.103228
F -4.239425 2.262928 0.362863
F -2.449102 2.660858 1.502423
C 2.600156 -0.949175 0.406002
C 3.984800 -0.797773 0.306550
C 4.553405 0.466481 0.169631
C 3.747618 1.600109 0.134240
C 2.360464 1.481729 0.239095
H 2.144807 -1.934614 0.514638
H 4.615811 -1.684476 0.346712
H 5.635429 0.569438 0.109088
H 4.189971 2.591162 0.046419
H 1.717112 2.360325 0.249263
C -0.451089 -3.205277 0.674593
O 0.601598 -3.521600 0.130981
C -0.777282 -3.612416 2.108616
N -1.409345 -2.531832 -0.031459
C -1.165001 -2.206028 -1.415885
C -0.181902 -1.127928 -1.640936

C 0.550323 -0.318984 -2.204775
C 1.501270 0.524181 -2.846787
C 1.142947 1.804709 -3.286769
C 2.121961 2.577532 -3.886596
C 3.426911 2.132689 -4.064545
C 3.766608 0.856314 -3.633737
C 2.810711 0.052386 -3.028487
H -2.187589 -2.079793 0.431763
H -0.791127 -3.100925 -1.930323
H -2.112744 -1.903037 -1.875096
H 0.135830 2.189941 -3.144422
N 1.770180 3.928420 -4.358787
H 4.148926 2.791554 -4.538575
H 4.782149 0.490478 -3.765515
H 3.065434 -0.942761 -2.668258
C -1.987521 -2.889375 2.693199
C -1.056615 -5.119635 2.055927
C 0.457651 -3.349913 2.969801
O 2.668896 4.602344 -4.835467
O 0.610556 4.277655 -4.243550
H 0.284581 -3.720107 3.989530
H 1.333569 -3.863488 2.556525
H 0.679857 -2.275392 3.029460
H -1.258752 -5.495175 3.068511
H -1.933095 -5.341637 1.430375
H -0.194380 -5.661602 1.648429
H -2.124113 -3.196329 3.738560
H -1.857779 -1.797592 2.687123
H -2.917895 -3.145229 2.165092

TS_{rc}^{A}

56
Energy: -4170.64506668
Cu 0.292824 0.946979 0.761964
Cl 1.135321 1.403247 2.667741
C -0.270547 2.737892 0.734505
O 0.366254 -1.011056 0.960371
S 0.795956 -2.174328 0.105287
O 2.249321 -2.322828 0.021470
C 0.191235 -3.569304 1.130358
O 0.064855 -2.290034 -1.159669
F 0.495850 -4.706699 0.524610
F 0.760007 -3.541521 2.322003
F -1.122113 -3.479219 1.261557
C 0.414622 3.693275 0.005200
C -0.139791 4.970080 -0.089790
C -1.339642 5.268908 0.550127
C -1.992036 4.293956 1.297798
C -1.452684 3.011973 1.403138
H 1.350401 3.450920 -0.494555
H 0.376036 5.733158 -0.671183
H -1.760485 6.270057 0.475752
H -2.919823 4.527152 1.818050
H -1.947994 2.253344 2.008551
C 2.921802 0.307650 -1.250556
O 2.131692 1.166434 -0.803726
C 4.299415 0.100700 -0.663845
N 2.526464 -0.338401 -2.362910
C 1.222621 0.045847 -2.876837
C 0.390990 0.591672 -1.794943
C -0.570768 0.741954 -0.999450
C -2.024981 0.614552 -0.997668
C -2.635791 -0.609379 -0.721975
C -4.021603 -0.656362 -0.712789
C -4.817753 0.452031 -0.968081
C -4.193103 1.662399 -1.238721
C -2.805698 1.750146 -1.244925
H 2.924044 -1.238762 -2.601752
H 1.339692 0.775833 -3.691069
H 0.703662 -0.845395 -3.254319
H -2.047798 -1.505062 -0.535174
N -4.678219 -1.942089 -0.420293
H -5.898570 0.349646 -0.948054
H -4.791040 2.548370 -1.440545
H -2.312617 2.701395 -1.441366
C 4.200988 0.087255 0.861631
C 4.964062 -1.174710 -1.173251
C 5.111356 1.323732 -1.121893
H 6.130215 1.244899 -0.720561
H 5.181109 1.376452 -2.217797
H 4.667454 2.255904 -0.751573

H 5.952849 -1.269203 -0.706799
H 4.380433 -2.063795 -0.904571
H 5.128393 -1.150229 -2.261087
H 5.212447 0.006928 1.281797
H 3.739248 1.005584 1.244387
H 3.605959 -0.765359 1.210967
O -5.898576 -1.963721 -0.442522
O -3.963399 -2.896967 -0.174870

TS_{ar}^{B}

56
Energy: -4170.65315822
Cu 0.387217 0.873786 0.452884
Cl 0.864998 1.179327 2.541103
C -1.005483 2.226393 0.519540
O 1.613514 -0.661589 0.332708
S 1.201199 -2.064902 -0.055043
O 1.786706 -2.460543 -1.339099
C 2.105146 -3.017073 1.225334
O -0.217069 -2.326438 0.162327
F 1.895835 -4.309417 1.039853
F 3.403996 -2.764180 1.124829
F 1.680409 -2.664862 2.422808
C -0.741653 3.387901 -0.194806
C -1.412447 4.556327 0.164314
C -2.341620 4.540420 1.198786
C -2.604044 3.357361 1.889015
C -1.933662 2.188147 1.555197
H -0.016399 3.386060 -1.005895
H -1.206964 5.477550 -0.377771
H -2.868539 5.453614 1.469048
H -3.322315 3.345100 2.706600
H -2.123555 1.263758 2.097111
C 2.699907 1.121884 -1.294343
O 1.986610 2.008272 -0.802917
C 4.164375 0.974301 -0.921406
N 2.185287 0.296155 -2.243738
C 0.782462 0.409694 -2.546944
C -0.154042 0.524845 -1.402515
C -1.278780 0.681930 -0.861559
C -2.683845 0.334820 -0.776835
C -3.033465 -1.005414 -0.594551
C -4.384199 -1.322194 -0.584649
C -5.380268 -0.369879 -0.747592
C -5.013096 0.959590 -0.926558
C -3.672198 1.315591 -0.932246
H 2.561447 -0.648394 -2.296044
H 0.611964 1.304167 -3.164939
H 0.491561 -0.468797 -3.138203
H -2.270786 -1.771412 -0.453307
N -4.781597 -2.729790 -0.400423
H -6.420283 -0.682797 -0.731333
H -5.778828 1.720161 -1.059246
H -3.375013 2.355340 -1.062676
C 4.783504 -0.326637 -1.425050
C 4.873769 2.168010 -1.576579
C 4.299897 1.064891 0.599235
H 5.942941 2.139433 -1.326771
H 4.779837 2.139119 -2.671661
H 4.458753 3.116688 -1.214625
H 5.365624 1.072066 0.865657
H 3.836773 1.979469 0.986881
H 3.821003 0.210561 1.093629
H 5.832702 -0.370237 -1.105043
H 4.280424 -1.213078 -1.013310
H 4.778698 -0.391858 -2.522501
O -5.977605 -2.970310 -0.369980
O -3.892408 -3.553178 -0.295580

I_2

56
Energy: -4170.70952845
Cu 0.280361 -1.429756 -1.981685
Cl 0.953666 -3.465302 -2.483937
C 2.058584 0.382916 -0.685877
O -0.071253 -1.768096 -0.170323
S -1.586482 -1.720842 0.063982
O -2.302571 -1.924070 -1.199479
C -1.813974 -3.256271 1.045240
O -1.995103 -0.608892 0.907735

F -3.100488 -3.375444 1.334186
F -1.418674 -4.298282 0.344948
F -1.116464 -3.173119 2.163549
C 2.697067 -0.423151 -1.637796
C 3.800218 -1.195209 -1.281703
C 4.284206 -1.153593 0.018589
C 3.673059 -0.327815 0.962544
C 2.568902 0.436644 0.615218
H 2.377473 -0.392558 -2.681008
H 4.269734 -1.833677 -2.027013
H 5.141022 -1.761918 0.301407
H 4.052641 -0.294016 1.982182
H 2.068832 1.053469 1.361191
C -1.127905 -0.671053 -4.133206
O 0.037727 -1.011118 -3.788813
C -1.724050 -1.350947 -5.349975
N -1.857162 0.194562 -3.438948
C -1.472308 0.823437 -2.178152
C -0.141281 0.399397 -1.684336
C 0.802627 1.085260 -1.033322
C 0.634024 2.494098 -0.611570
C -0.557441 2.931625 -0.030195
C -0.672302 4.265309 0.337909
C 0.357837 5.179232 0.169535
C 1.549487 4.732758 -0.390201
C 1.687993 3.404222 -0.771860
H -2.814476 0.339525 -3.734950
H -1.453041 1.915073 -2.333546
H -2.264377 0.600017 -1.447901
H -1.371729 2.240825 0.182430
N -1.936577 4.728141 0.932220
H 0.214731 6.209932 0.480236
H 2.374865 5.427497 -0.529372
H 2.624022 3.055012 -1.206536
C -2.920718 -0.604478 -5.931602
C -0.638759 -1.514107 -6.413625
C -2.159311 -2.734434 -4.834286
H -2.589073 -3.307092 -5.667189
H -1.305814 -3.291461 -4.425016
H -2.917397 -2.648054 -4.043271
H -1.049136 -2.078638 -7.260681
H -0.290760 -0.542352 -6.789682
H 0.221530 -2.062458 -6.015166
H -3.251430 -1.117844 -6.843012
H -3.788471 -0.597896 -5.254998
H -2.672345 0.429881 -6.207725
O -1.988307 5.887105 1.309198
O -2.852143 3.926005 0.998885

TS_{rc}^{B}

56
Energy: -4170.67939921
Cu -1.433424 1.109218 0.193594
Cl -3.137215 2.003469 -0.875283
C 0.299381 -1.329169 1.897350
O 0.070004 1.955655 0.974784
S 1.277584 2.141171 0.063154
O 1.040594 1.593416 -1.275049
C 1.252211 3.960481 -0.160479
O 2.526553 1.811697 0.729719
F 2.249784 4.317882 -0.952545
F 0.103053 4.315691 -0.714123
F 1.380892 4.562674 1.009301
C -0.547101 -0.552150 2.702581
C -0.539444 -0.685808 4.080048
C 0.316982 -1.599045 4.690316
C 1.165408 -2.375213 3.908012
C 1.158925 -2.242120 2.526393
H -1.201290 0.181939 2.242117
H -1.196865 -0.061946 4.682006
H 0.327968 -1.697860 5.774146
H 1.842182 -3.087953 4.375332
H 1.825932 -2.858024 1.926187
C -3.037391 -1.100083 -0.749459
O -2.476015 -0.782678 0.334374
C -4.480180 -1.549654 -0.761639
N -2.291600 -1.134499 -1.853736
C -0.941706 -0.591142 -1.758738
C -0.593510 -0.548189 -0.308458
C 0.284634 -1.235550 0.434858
C 1.381834 -1.896264 -0.335739

C 2.655041 -1.326557 -0.377538
C 3.642152 -1.991572 -1.089752
C 3.415099 -3.186598 -1.759570
C 2.143988 -3.743411 -1.708455
C 1.132922 -3.100859 -0.999883
H -2.741233 -1.183652 -2.759527
H -0.229483 -1.246254 -2.277481
H -0.894556 0.401473 -2.229150
H 2.873473 -0.379838 0.120377
N 4.995110 -1.405939 -1.141244
H 4.230284 -3.655396 -2.302718
H 1.942233 -4.683951 -2.216542
H 0.133870 -3.536880 -0.948805
C -5.249987 -0.772910 0.305762
C -5.118011 -1.325168 -2.131024
C -4.464342 -3.046102 -0.417959
H -6.181934 -1.587234 -2.077296
H -5.047723 -0.272401 -2.437210
H -4.680345 -1.964631 -2.912304
H -5.496714 -3.418083 -0.384460
H -3.915578 -3.629220 -1.170534
H -4.005367 -3.221139 0.563457
H -6.296903 -1.102647 0.302795
H -4.834041 -0.950957 1.303855
H -5.214404 0.306201 0.108971
O 5.829242 -1.991924 -1.813588
O 5.188564 -0.385818 -0.508575

Reaction 38

reactant

35
Energy: -877.565146406
C -3.920224 0.129090 0.224928
O -4.313597 -0.168041 -0.892408
C -4.711146 -0.275620 1.473372
N -2.768533 0.841758 0.414042
H -2.440239 1.053922 1.345031
C -1.942090 1.231156 -0.711845
C -0.542621 0.877298 -0.518524
H -2.366909 0.716724 -1.586056
H -2.030501 2.311470 -0.908240
C 0.622891 0.599323 -0.339204
C 1.991867 0.265715 -0.134730
C 2.340577 -0.941568 0.492130
C 3.670461 -1.267525 0.696488
C 4.649279 -0.379371 0.267294
C 4.337220 0.821384 -0.358554
C 3.005052 1.141771 -0.556807
H 1.552410 -1.618897 0.813475
H 3.965518 -2.194819 1.178717
N 6.059559 -0.721123 0.482179
H 5.138669 1.481714 -0.676502
H 2.730415 2.075522 -1.042537
C -4.075599 0.149766 2.793367
C -6.095734 0.363185 1.344166
C -4.844945 -1.799313 1.438410
H -4.706919 -0.184442 3.627524
H -3.084199 -0.302964 2.942413
H -3.986861 1.242817 2.879405
H -5.484517 -2.140017 2.264500
H -5.288966 -2.124210 0.490919
H -3.866115 -2.288286 1.543533
H -6.740138 0.036700 2.172284
H -6.033185 1.460255 1.375597
H -6.565257 0.074402 0.397083
O 6.898198 0.075770 0.093081
O 6.304448 -1.780376 1.036977

I_c

70
Energy: -4478.18791968
Cu 0.694553 0.075501 -0.534159
Cl 1.330258 -0.468819 -2.545794
C 1.952123 1.491735 -0.764431
O -0.596194 -1.470776 -0.379566
S -1.419434 -1.885168 0.814037
O -2.127330 -0.771611 1.451474
C -2.740961 -2.840754 -0.024279
O -0.750036 -2.847623 1.679474

F -3.612154 -3.276445 0.868258
F -3.384758 -2.058272 -0.897790
F -2.225324 -3.866473 -0.677491
C 1.387811 2.730185 -0.532332
C 2.260439 3.813136 -0.377616
C 3.632381 3.641580 -0.493127
C 4.156438 2.379794 -0.764730
C 3.310892 1.282167 -0.912155
C -1.063205 3.015922 2.071468
O -0.188900 3.870446 1.974972
C -2.541851 3.402839 2.032278
N -0.762613 1.697174 2.261933
C 0.606287 1.296495 2.422573
C 1.088299 0.221510 1.539090
C 1.765331 -0.758636 1.218131
C 2.656379 -1.807392 0.849551
C 2.188020 -3.068383 0.447867
C 3.097520 -4.053031 0.098110
C 4.454861 -3.766968 0.162466
C 4.945372 -2.531251 0.564369
C 4.037349 -1.543480 0.905064
H 0.318145 2.883849 -0.422428
H 1.831737 4.784344 -0.137062
H 4.299167 4.493976 -0.377964
H 5.230254 2.240129 -0.880876
H 3.710108 0.300344 -1.158341
H -1.467376 0.967393 2.183039
H 1.216986 2.197019 2.252487
H 0.801439 0.954949 3.452347
H 1.118295 -3.257899 0.426442
H 2.772552 -5.039065 -0.219796
N 5.418906 -4.818552 -0.206975
H 6.017693 -2.364956 0.600567
H 4.381054 -0.561423 1.224749
C -3.497531 2.227646 2.201766
C -2.756536 4.404147 3.170837
C -2.793258 4.093968 0.691006
O -3.289444 1.136545 -1.004335
C -4.515179 0.701785 -1.614514
C -5.535273 0.619902 -0.510240
C -2.097325 0.885657 -1.526820
O -1.122117 1.229104 -0.870231
C -2.019606 0.224299 -2.864183
H -4.797022 1.430256 -2.388074
H -4.371751 -0.275624 -2.089175
H -6.500660 0.284669 -0.906063
H -5.197960 -0.092439 0.253407
H -5.672111 1.598532 -0.034712
H -2.770466 0.617592 -3.558077
H -1.016799 0.353959 -3.275529
H -2.191608 -0.853043 -2.740357
H -3.356608 1.718017 3.165668
H -4.532302 2.598839 2.181240
H -3.391968 1.490289 1.395404
H -3.833544 4.447315 0.640968
H -2.124943 4.954941 0.569429
H -2.627814 3.399773 -0.143225
H -3.789944 4.777733 3.148327
H -2.589261 3.933984 4.150407
H -2.071354 5.254258 3.076490
O 6.603969 -4.537830 -0.143510
O 4.967457 -5.896909 -0.550048

I_1

56
Energy: -4170.66674038
Cu -0.020502 0.151037 -0.017617
Cl 0.011573 0.291452 2.107331
C 1.842071 0.215019 0.323273
O -2.046608 0.368715 -0.298849
S -2.028687 1.754930 -0.899556
O -2.687078 1.889134 -2.184668
C -3.009941 2.735762 0.299982
O -0.642222 2.268468 -0.784695
F -3.015904 4.001827 -0.081507
F -4.249180 2.274931 0.329341
F -2.473820 2.639138 1.502642
C 2.598291 -0.937002 0.408146
C 3.981343 -0.787563 0.288576
C 4.548677 0.474528 0.127573
C 3.743622 1.608443 0.089935

C 2.357821 1.493020 0.215567
H 2.143078 -1.920283 0.534200
H 4.612255 -1.674161 0.330560
H 5.629571 0.575883 0.049004
H 4.185871 2.597737 -0.016150
H 1.714370 2.371348 0.223458
C -0.454482 -3.196019 0.684394
O 0.600150 -3.514151 0.146148
C -0.786397 -3.596610 2.118896
N -1.410711 -2.527233 -0.029358
C -1.166457 -2.219165 -1.417931
C -0.179777 -1.148497 -1.656957
C 0.561041 -0.341258 -2.210395
C 1.515652 0.501208 -2.852069
C 1.160707 1.792375 -3.270989
C 2.112389 2.601807 -3.870452
C 3.397288 2.103423 -4.044036
C 3.769554 0.824210 -3.651317
C 2.817622 0.017435 -3.051433
H -2.193758 -2.076845 0.427088
H -0.795622 -3.121262 -1.922202
H -2.113809 -1.919113 -1.879762
H 0.148668 2.151586 -3.099214
H 1.877816 3.608799 -4.201965
N 4.411859 2.968577 -4.671978
H 4.789013 0.487480 -3.813582
H 3.072115 -0.984632 -2.712456
C -1.999076 -2.871195 2.695342
C -1.066057 -5.104018 2.071103
C 0.444961 -3.331341 2.984222
O 5.534441 2.510459 -4.802681
O 4.062011 4.083926 -5.016152
H 0.267764 -3.698211 4.004416
H 1.322521 -3.846331 2.576287
H 0.667164 -2.256740 3.041225
H -1.273212 -5.475159 3.084273
H -1.939592 -5.328407 1.442294
H -0.202067 -5.648009 1.670139
H -2.139023 -3.172252 3.741949
H -1.870289 -1.779441 2.683369
H -2.927560 -3.130706 2.165661

TS_{rc}^{A}

56
Energy: -4170.64705303
Cu 0.033154 -0.014577 -0.002196
Cl -0.020728 0.001282 2.128894
C -0.879304 1.623271 0.115615
O 0.524304 -1.917236 -0.167955
S 1.371781 -2.761464 -1.082125
O 2.794629 -2.729740 -0.741730
C 0.777406 -4.431577 -0.614965
O 1.042538 -2.624166 -2.502426
F 1.422010 -5.336249 -1.335197
F 0.993961 -4.655683 0.667667
F -0.522318 -4.521398 -0.866048
C -0.202210 2.822433 -0.010643
C -0.955532 3.995615 -0.069064
C -2.344290 3.948408 0.015208
C -2.993116 2.727254 0.166162
C -2.257165 1.544205 0.227765
H 0.883631 2.848894 -0.077648
H -0.445173 4.951277 -0.181141
H -2.922557 4.869687 -0.025137
H -4.077983 2.685762 0.249561
H -2.759978 0.587330 0.367823
C 3.275795 0.231517 -0.872113
O 2.217659 0.842385 -0.610656
C 4.347549 0.000531 0.168681
N 3.459678 -0.112175 -2.160170
C 2.379539 0.250704 -3.062860
C 1.117965 0.377544 -2.318922
C -0.071350 0.177319 -1.964440
C -1.373104 -0.068911 -2.569420
C -1.899013 -1.362607 -2.664149
C -3.157802 -1.551303 -3.215106
C -3.874363 -0.446422 -3.655990
C -3.374262 0.846054 -3.562147
C -2.118277 1.032899 -3.009394
H 4.091894 -0.866294 -2.400238
H 2.624565 1.186208 -3.586430

H 2.234022 -0.552256 -3.798286
H -1.308399 -2.211378 -2.327518
H -3.591062 -2.542657 -3.310019
N -5.206503 -0.648264 -4.238028
H -3.972905 1.680105 -3.916149
H -1.706652 2.035222 -2.900470
C 3.695372 -0.502759 1.456722
C 5.422627 -0.973302 -0.303961
C 4.974294 1.383901 0.408432
H 5.760343 1.291569 1.169306
H 5.433079 1.784535 -0.506788
H 4.227358 2.100520 0.770920
H 6.168370 -1.090261 0.492550
H 5.000722 -1.963955 -0.513969
H 5.959873 -0.600512 -1.188883
H 4.469035 -0.605092 2.229380
H 2.931287 0.193749 1.822869
H 3.222044 -1.480156 1.303298
O -5.811666 0.342537 -4.615487
O -5.624029 -1.792323 -4.307631

TS_{ar}^{B}

56
Energy: -4170.65526362
Cu 0.470171 0.771812 0.467552
Cl 0.942623 1.105615 2.552204
C -1.065424 1.960587 0.568300
O 1.841390 -0.646882 0.319983
S 1.593013 -2.077891 -0.097969
O 2.148036 -2.360707 -1.424927
C 2.684361 -2.945315 1.093638
O 0.237424 -2.537048 0.184926
F 2.658244 -4.243823 0.842579
F 3.927471 -2.499405 0.961032
F 2.266871 -2.722371 2.324282
C -0.947864 3.149761 -0.139032
C -1.745009 4.228779 0.241007
C -2.650896 4.095973 1.287658
C -2.763146 2.885511 1.970724
C -1.966462 1.805116 1.616561
H -0.239858 3.238934 -0.960352
H -1.656494 5.171921 -0.295051
H -3.277826 4.938690 1.572604
H -3.463975 2.782748 2.796909
H -2.040738 0.859720 2.150389
C 2.716547 1.282910 -1.291287
O 1.918542 2.076765 -0.771718
C 4.193399 1.286850 -0.940336
N 2.281287 0.426122 -2.251722
C 0.871230 0.395198 -2.541185
C -0.058836 0.383634 -1.386188
C -1.180232 0.409329 -0.816669
C -2.535869 -0.094366 -0.696684
C -2.723879 -1.450577 -0.408386
C -4.015118 -1.957823 -0.336647
C -5.082065 -1.100488 -0.560562
C -4.914484 0.250230 -0.845641
C -3.628417 0.757563 -0.902454
H 2.757610 -0.470244 -2.329157
H 0.599374 1.279811 -3.136716
H 0.670122 -0.496395 -3.149696
H -1.855413 -2.086498 -0.237716
H -4.203558 -3.003508 -0.112034
N -6.451488 -1.639344 -0.490250
H -5.786925 0.875129 -1.010823
H -3.458176 1.814055 -1.104293
C 4.935389 0.057936 -1.459279
C 4.765657 2.549866 -1.599687
C 4.339727 1.385065 0.578564
H 5.835035 2.632634 -1.363298
H 4.661115 2.514247 -2.693567
H 4.258667 3.449098 -1.228637
H 5.403121 1.493278 0.831792
H 3.795684 2.249471 0.975885
H 3.950813 0.487185 1.074831
H 5.988031 0.121370 -1.154249
H 4.532363 -0.876629 -1.043846
H 4.922372 -0.003709 -2.556856
O -7.369517 -0.860357 -0.684568
O -6.574950 -2.826089 -0.243295

I_2

56
Energy: -4170.71067863
Cu 0.216784 -1.475092 -1.952458
Cl 0.810798 -3.549026 -2.382748
C 2.059109 0.368781 -0.720679
O -0.118715 -1.739686 -0.127803
S -1.624285 -1.628770 0.140619
O -2.381269 -1.847037 -1.095431
C -1.882631 -3.116795 1.185224
O -1.971617 -0.474390 0.956435
F -3.163689 -3.171184 1.515480
F -1.551519 -4.198874 0.513833
F -1.148749 -3.020598 2.278716
C 2.672146 -0.493131 -1.640268
C 3.780077 -1.249023 -1.267787
C 4.296605 -1.135530 0.016222
C 3.712431 -0.253938 0.925357
C 2.602189 0.494758 0.561853
H 2.330684 -0.518391 -2.676479
H 4.230740 -1.929068 -1.987607
H 5.159035 -1.729724 0.311740
H 4.118572 -0.161670 1.931004
H 2.125060 1.157185 1.283396
C -1.118036 -0.709669 -4.158696
O 0.016937 -1.101463 -3.769956
C -1.683304 -1.357481 -5.407475
N -1.839365 0.183190 -3.491437
C -1.472266 0.812183 -2.223560
C -0.152733 0.372346 -1.712339
C 0.801466 1.059542 -1.078511
C 0.618698 2.470404 -0.666556
C -0.532725 2.856083 0.031159
C -0.707633 4.176944 0.421285
C 0.281915 5.098382 0.108583
C 1.445862 4.740984 -0.562479
C 1.612819 3.418627 -0.940360
H -2.772777 0.375829 -3.832833
H -1.438743 1.902305 -2.382616
H -2.278661 0.596009 -1.507786
H -1.268166 2.100288 0.308808
H -1.586305 4.500480 0.971759
N 0.098867 6.500181 0.510359
H 2.194657 5.498618 -0.774291
H 2.518110 3.108163 -1.460295
C -2.869611 -0.600480 -5.995884
C -0.571256 -1.480823 -6.449429
C -2.122925 -2.757443 -4.944897
H -2.526375 -3.309151 -5.804562
H -1.278274 -3.320404 -4.526290
H -2.904748 -2.697103 -4.174696
H -0.956341 -2.023910 -7.321969
H -0.223748 -0.495614 -6.789166
H 0.284362 -2.033467 -6.047226
H -3.182448 -1.095091 -6.923769
H -3.749027 -0.609494 -5.334433
H -2.617588 0.439360 -6.247693
O 0.990663 7.285284 0.233137
O -0.934096 6.788477 1.091546

TS_{rc}^{B}

56
Energy: -4170.68278989
Cu -1.869802 0.423891 -0.019790
Cl -3.616820 0.204414 -1.341869
C 0.572486 -0.564885 2.190226
O -1.124369 2.011617 0.708292
S -0.087763 2.709300 -0.162282
O 0.168391 1.973390 -1.402556
C -1.019890 4.201609 -0.677425
O 1.054684 3.177013 0.607321
F -0.263227 4.932632 -1.480461
F -2.116010 3.834799 -1.321532
F -1.351383 4.915208 0.385080
C -0.654754 -0.246633 2.791131
C -0.780729 -0.197767 4.168570
C 0.317801 -0.464364 4.982320
C 1.542830 -0.778506 4.403499
C 1.671043 -0.827166 3.022302
H -1.512478 -0.010855 2.168397

H -1.740324 0.064218 4.609335
H 0.219355 -0.418430 6.065246
H 2.408374 -0.983824 5.030437
H 2.634849 -1.076946 2.582611
C -2.013830 -2.376179 -0.679780
O -1.848296 -1.692933 0.368498
C -3.025061 -3.498583 -0.709333
N -1.197244 -2.151354 -1.707756
C -0.312941 -0.995585 -1.614768
C -0.255951 -0.610235 -0.174320
C 0.726107 -0.660240 0.735925
C 2.107159 -0.739895 0.170235
C 2.898605 0.409174 0.091868
C 4.189839 0.323047 -0.412283
C 4.665017 -0.912997 -0.829655
C 3.897040 -2.068306 -0.763573
C 2.608017 -1.973532 -0.259539
H -1.420706 -2.532386 -2.618773
H 0.697480 -1.262751 -1.952239
H -0.680861 -0.178520 -2.252026
H 2.494230 1.369924 0.414857
H 4.827857 1.198386 -0.492164
N 6.032419 -1.007947 -1.363727
H 4.318505 -3.011775 -1.097884
H 1.981241 -2.862879 -0.184056
C -4.228319 -3.106078 0.146974
C -3.477385 -3.797447 -2.137015
C -2.321311 -4.721743 -0.103561
H -4.266593 -4.558895 -2.109833
H -3.892423 -2.902362 -2.620361
H -2.669829 -4.210298 -2.760075
H -3.024128 -5.564752 -0.078591
H -1.447858 -5.023112 -0.698280
H -1.992358 -4.519761 0.923771
H -4.956301 -3.927486 0.137715
H -3.932819 -2.917690 1.185258
H -4.708365 -2.198779 -0.241400
O 6.412140 -2.104825 -1.740898
O 6.696484 0.013425 -1.394148

Reaction 39

reactant

34
Energy: -748.343919123
C -4.014439 -0.428816 0.256675
O -4.631003 -1.000067 -0.632211
C -4.741887 0.207390 1.446277
N -2.658739 -0.334074 0.218273
H -2.127686 0.128466 0.942259
C -1.926695 -0.918488 -0.894576
C -0.503172 -0.672068 -0.738019
H -2.140783 -1.997663 -0.948348
H -2.303611 -0.496792 -1.839467
C 0.669586 -0.436702 -0.551116
C 2.048587 -0.160719 -0.332845
C 3.017432 -0.496974 -1.285944
C 4.362365 -0.227500 -1.071785
C 4.757029 0.388873 0.111699
C 3.812659 0.732383 1.072404
C 2.464105 0.462399 0.858509
H 2.687656 -0.978264 -2.204929
H 5.099281 -0.496977 -1.825087
H 5.807990 0.606538 0.293460
H 4.121548 1.215936 2.000745
O 1.505867 0.778586 1.763653
C -3.820725 0.865484 2.468451
C -5.698532 1.253538 0.871073
C -5.546162 -0.904224 2.122355
H -4.421390 1.291606 3.283600
H -3.125834 0.144705 2.923786
H -3.240449 1.691456 2.031681
H -6.165931 -0.486445 2.928286
H -6.198959 -1.400799 1.395580
H -4.884093 -1.663481 2.562441
H -6.316663 1.682615 1.672334
H -5.147829 2.074813 0.390688
H -6.356804 0.799624 0.121610
H 1.924998 1.195801 2.529382

I_c

69
Energy: -4348.97388099
Cu 0.637921 -0.023831 -0.491375
Cl 1.276596 -0.878172 -2.425202
C 1.874992 1.407266 -0.762312
O -0.708861 -1.494618 -0.222667
S -1.899033 -1.575973 0.683265
O -3.087920 -0.898498 0.157912
C -2.334047 -3.352794 0.528255
O -1.599834 -1.358286 2.100880
F -3.433932 -3.593388 1.224009
F -2.552608 -3.644926 -0.746431
F -1.363651 -4.137072 0.986814
C 1.348659 2.632097 -0.399380
C 2.200078 3.740447 -0.428632
C 3.523207 3.607536 -0.826997
C 4.011478 2.359203 -1.201778
C 3.184267 1.236257 -1.172600
C -1.177331 2.729513 2.184411
O -0.339168 3.620436 2.091966
C -2.669300 3.058044 2.228449
N -0.819852 1.418017 2.311697
C 0.559214 1.055568 2.479113
C 1.125214 0.109962 1.491023
C 1.954749 -0.780871 1.239748
C 2.918995 -1.729953 0.861543
C 2.546679 -3.080236 0.662033
C 3.498690 -3.995774 0.215512
C 4.804781 -3.583697 -0.003455
C 5.196521 -2.260346 0.219132
C 4.256439 -1.341414 0.643116
H 0.322605 2.764849 -0.068757
H 1.800274 4.702091 -0.110568
H 4.177182 4.477612 -0.848819
H 5.041609 2.246962 -1.537990
H 3.556243 0.266078 -1.493167
H -1.503182 0.668225 2.370444
H 1.131931 1.993624 2.427992
H 0.723149 0.611210 3.472760
O 1.280183 -3.400201 0.928875
H 3.208369 -5.032678 0.047682
H 5.536520 -4.311592 -0.349804
H 6.227246 -1.957533 0.053358
H 4.523254 -0.299565 0.812368
C -3.582366 1.843448 2.100838
C -2.906850 3.738090 3.581568
C -2.953727 4.048417 1.099608
O -3.173441 1.684816 -1.342398
C -4.468656 1.295518 -1.820297
C -5.444298 2.336995 -1.338911
C -2.098920 0.939860 -1.579342
O -1.097479 1.202866 -0.923142
C -2.168889 -0.088104 -2.660553
H -4.458852 1.234985 -2.917926
H -4.699385 0.302788 -1.410864
H -6.456414 2.093544 -1.682046
H -5.449128 2.380245 -0.243373
H -5.175394 3.329179 -1.720717
H -2.530233 0.367409 -3.591193
H -1.180575 -0.523079 -2.825773
H -2.865771 -0.883640 -2.371774
H -3.462654 1.135189 2.934354
H -4.629979 2.177742 2.125238
H -3.423268 1.297054 1.161188
H -4.005206 4.369277 1.139272
H -2.312730 4.932289 1.191926
H -2.771723 3.585053 0.120456
H -3.954966 4.060329 3.658663
H -2.703277 3.049097 4.414213
H -2.260627 4.617122 3.694521
H 1.067619 -4.293514 0.622081

I_1

55
Energy: -4041.45409108
Cu 0.366264 -0.076094 -0.090916
Cl 0.374005 -0.070830 2.048746
C 2.187528 -0.392484 0.289402
O -1.564114 0.584393 -0.442701
S -1.341782 2.065381 -0.596508

O -2.030278 2.696238 -1.723535
C -2.183460 2.786839 0.867808
O 0.098465 2.342380 -0.448349
F -1.997837 4.096330 0.864858
F -3.477605 2.522197 0.792520
F -1.697354 2.274606 1.981311
C 2.716094 -1.666153 0.377189
C 4.103022 -1.779103 0.291855
C 4.902611 -0.643137 0.170266
C 4.325414 0.621098 0.139375
C 2.937425 0.765253 0.212310
H 2.077182 -2.540268 0.516462
H 4.556329 -2.768136 0.335717
H 5.985194 -0.746662 0.124026
H 4.947030 1.512603 0.067407
H 2.470780 1.749562 0.215898
C -0.965455 -3.074327 0.790761
O 0.070028 -3.718066 0.890009
C -2.023284 -3.051140 1.894295
N -1.280601 -2.416067 -0.373383
C -0.402571 -2.482013 -1.518116
C 0.322121 -1.222119 -1.779502
C 0.894967 -0.357484 -2.448182
C 1.500232 0.743562 -3.080116
C 0.651801 1.744224 -3.607935
C 1.209126 2.940328 -4.054586
C 2.586177 3.114492 -4.013907
C 3.435934 2.109632 -3.540678
C 2.894445 0.927560 -3.070574
H -2.064687 -1.775543 -0.407415
H 0.315659 -3.286531 -1.308654
H -0.968215 -2.751531 -2.420669
O -0.656357 1.461344 -3.678753
H 0.554710 3.717895 -4.444161
H 3.010881 4.051560 -4.370701
H 4.512688 2.262272 -3.530375
H 3.524588 0.137915 -2.662964
C -2.855617 -4.324091 1.686793
C -1.319591 -3.119901 3.246925
C -2.938460 -1.827445 1.849691
H -1.213212 2.177275 -3.303983
H -2.067084 -3.211517 4.046878
H -0.646284 -3.982639 3.292528
H -0.725984 -2.214648 3.429173
H -3.612064 -1.854221 2.716818
H -2.374759 -0.884917 1.894044
H -3.584038 -1.810434 0.958784
H -3.618545 -4.401283 2.473845
H -3.369664 -4.315389 0.715099
H -2.219127 -5.217256 1.733861

TS_{rc}^{A}

55
Energy: -4041.44271625
Cu -0.633753 -0.410637 -0.694932
Cl -0.202987 -1.077310 -2.687565
C -2.224342 -1.405651 -0.825893
O 1.207998 0.373999 -0.419254
S 1.729546 1.693534 0.062739
O 2.456408 1.603520 1.329207
C 3.002486 2.047553 -1.207912
O 0.755119 2.785822 -0.069362
F 3.612872 3.185220 -0.922101
F 3.897527 1.065686 -1.225994
F 2.431489 2.137288 -2.394963
C -2.242023 -2.698075 -0.332331
C -3.446570 -3.399773 -0.380035
C -4.585355 -2.811428 -0.920719
C -4.526530 -1.516961 -1.425442
C -3.331237 -0.798425 -1.390718
H -1.346459 -3.151607 0.086820
H -3.485738 -4.415986 0.010237
H -5.520170 -3.368152 -0.957719
H -5.410442 -1.057353 -1.865430
H -3.271403 0.202424 -1.814777
C 1.700785 -1.756010 1.396188
O 0.543869 -2.055784 1.045943
C 2.933883 -2.331766 0.737133
N 1.819253 -0.982006 2.502225
C 0.525788 -0.610596 3.057767
C -0.448476 -0.488887 1.967357

C -1.186804 0.099866 1.122837
C -2.149687 1.183378 1.214563
C -2.318792 2.030045 0.109696
C -3.254891 3.054785 0.142410
C -4.027345 3.248099 1.281829
C -3.867947 2.416560 2.388612
C -2.933844 1.391749 2.355123
H 2.561712 -0.286220 2.520333
H 0.216800 -1.361049 3.798962
H 0.610349 0.366437 3.552663
O -1.571720 1.807451 -1.017061
H -3.360099 3.690260 -0.734328
H -4.760549 4.052139 1.305647
H -4.478001 2.563933 3.277439
H -2.818398 0.720852 3.205509
C 2.728966 -2.430109 -0.772142
C 4.185886 -1.518341 1.056688
C 3.071367 -3.742973 1.333084
H 3.956070 -4.229524 0.901293
H 3.197514 -3.709905 2.424176
H 2.192415 -4.356780 1.099019
H 5.041375 -1.962485 0.531811
H 4.097311 -0.476464 0.721651
H 4.424015 -1.529782 2.129672
H 3.582717 -2.960971 -1.214065
H 1.812942 -2.979453 -1.020543
H 2.655945 -1.438932 -1.233483
H -0.718105 2.296808 -0.919496

TS_{ar}^{B}

55
Energy: -4041.44749487
Cu 0.102244 -0.666450 0.509730
Cl -0.332379 -1.221176 2.563005
C 1.909974 -1.360480 0.629958
O -1.615016 0.315472 0.370124
S -1.674008 1.783158 0.043964
O -2.245574 2.043424 -1.277472
C -2.915806 2.348361 1.268343
O -0.435857 2.490309 0.394878
F -3.128139 3.643440 1.108169
F -4.048416 1.688310 1.070056
F -2.475016 2.115131 2.487430
C 2.132248 -2.547959 -0.056807
C 3.147339 -3.394676 0.386443
C 3.933124 -3.032059 1.475211
C 3.709633 -1.822610 2.133905
C 2.691310 -0.977350 1.716635
H 1.513133 -2.813911 -0.911591
H 3.324041 -4.335669 -0.131290
H 4.730989 -3.691682 1.811430
H 4.320104 -1.541964 2.990022
H 2.503177 -0.036531 2.230104
C -1.857976 -1.666007 -1.391742
O -0.911910 -2.269906 -0.868960
C -3.301058 -2.067812 -1.139099
N -1.608960 -0.659648 -2.272137
C -0.240654 -0.261269 -2.472286
C 0.599625 -0.077416 -1.253377
C 1.759344 0.167718 -0.809460
C 2.994304 0.885249 -0.804701
C 2.982731 2.298636 -0.907109
C 4.206293 2.966460 -1.068487
C 5.395144 2.267016 -1.105984
C 5.414004 0.870612 -0.985450
C 4.224101 0.194845 -0.831299
H -2.295414 0.087722 -2.340123
H 0.273031 -1.021887 -3.078302
H -0.238796 0.678399 -3.039782
O 1.893540 3.059133 -0.901108
H 4.174835 4.050597 -1.149164
H 6.330056 2.812537 -1.223950
H 6.354508 0.325956 -1.015032
H 4.209297 -0.890784 -0.747432
C -4.308595 -1.026636 -1.618254
C -3.500856 -3.374355 -1.920372
C -3.490189 -2.326366 0.355376
H -4.523687 -3.742674 -1.763093
H -3.355881 -3.222642 -2.999626
H -2.798926 -4.144918 -1.578368
H -4.496818 -2.731346 0.527634

H -2.753954 -3.045517 0.731502
H -3.382665 -1.401407 0.935292
H -5.323214 -1.379835 -1.392071
H -4.178574 -0.061641 -1.108259
H -4.259310 -0.866362 -2.704808
H 1.084739 2.634820 -0.534055

I_2

55
Energy: -4041.49038894
Cu 0.258515 -1.374616 -1.920362
Cl 0.962211 -3.430804 -2.320000
C 2.040032 0.460086 -0.702159
O -0.229398 -1.625977 -0.114081
S -1.750338 -1.743358 0.018775
O -2.369341 -1.846803 -1.308105
C -1.912118 -3.405247 0.782825
O -2.318701 -0.807141 0.972668
F -3.200901 -3.638113 0.993508
F -1.424547 -4.329851 -0.015860
F -1.270322 -3.425219 1.938463
C 2.667404 -0.392036 -1.621291
C 3.763225 -1.161977 -1.236642
C 4.252299 -1.072460 0.058500
C 3.652505 -0.201355 0.969318
C 2.556187 0.560384 0.594344
H 2.351236 -0.394074 -2.665792
H 4.221833 -1.836054 -1.957051
H 5.104153 -1.677186 0.363532
H 4.037346 -0.128423 1.985119
H 2.068648 1.218654 1.312362
C -1.124154 -0.700468 -4.119061
O 0.036635 -1.033696 -3.755979
C -1.718659 -1.430086 -5.308358
N -1.848237 0.204677 -3.470755
C -1.470871 0.869983 -2.226674
C -0.142984 0.468679 -1.717367
C 0.796689 1.169661 -1.080265
C 0.645890 2.587459 -0.679909
C -0.474075 3.026687 0.047629
C -0.553859 4.349498 0.484163
C 0.472520 5.241019 0.202669
C 1.592166 4.821415 -0.511488
C 1.672894 3.501924 -0.934230
H -2.807785 0.328716 -3.767657
H -1.445631 1.956139 -2.413275
H -2.258538 0.677363 -1.484879
O -1.445553 2.120462 0.308778
H -1.428593 4.672706 1.050253
H 0.396908 6.270275 0.549127
H 2.400276 5.516371 -0.728586
H 2.552615 3.150829 -1.473704
C -2.939180 -0.730360 -5.898053
C -0.640873 -1.594745 -6.379608
C -2.115349 -2.808464 -4.751679
H -2.545849 -3.411972 -5.562317
H -1.243394 -3.334809 -4.340955
H -2.860304 -2.716593 -3.949124
H -1.042916 -2.196882 -7.204728
H -0.325202 -0.625180 -6.788801
H 0.238953 -2.105195 -5.973672
H -3.269148 -1.279799 -6.788612
H -3.797219 -0.722769 -5.209093
H -2.718570 0.300310 -6.209887
H -2.043437 2.445512 0.996271

TS_{rc}^{B}

55
Energy: -4041.46894560
Cu -0.442295 -0.358866 0.612531
Cl -2.022772 -0.801759 2.079286
C 1.452685 -1.788885 0.790484
O 0.289645 1.516490 0.864597
S -0.359312 2.522383 -0.038628
O -1.452456 1.941613 -0.829351
C -1.172385 3.666831 1.138368
O 0.619101 3.346099 -0.762544
F -1.762464 4.642161 0.463196
F -2.075837 3.009982 1.840974
F -0.268034 4.184580 1.953565

C 1.127018 -3.148267 0.784521
C 1.044389 -3.846173 1.982122
C 1.295471 -3.198465 3.189111
C 1.641516 -1.851590 3.200032
C 1.729318 -1.146286 2.006025
H 0.917079 -3.642773 -0.162205
H 0.778100 -4.901367 1.974074
H 1.217625 -3.747082 4.125770
H 1.832894 -1.341438 4.141510
H 2.005829 -0.091702 2.001794
C -1.939817 -1.478986 -1.562325
O -1.093320 -1.919909 -0.736285
C -3.397736 -1.876274 -1.503025
N -1.499270 -0.618046 -2.472535
C -0.113541 -0.194204 -2.366792
C 0.428538 -0.659456 -1.061017
C 1.589416 -1.012870 -0.496460
C 2.933144 -0.555111 -0.878381
C 3.143001 0.671419 -1.537666
C 4.439484 1.121709 -1.778990
C 5.531425 0.363712 -1.380537
C 5.338223 -0.857014 -0.735070
C 4.051956 -1.299962 -0.474577
H -2.143942 -0.068855 -3.027019
H 0.485638 -0.623593 -3.184393
H -0.072538 0.895969 -2.438452
O 2.072781 1.377088 -1.979005
H 4.566839 2.073903 -2.290794
H 6.539310 0.726074 -1.575168
H 6.191576 -1.455828 -0.423810
H 3.894497 -2.238444 0.055764
C -3.909530 -2.222419 -2.902454
C -3.566309 -3.067392 -0.564481
C -4.157818 -0.664231 -0.936502
H -5.223779 -0.921206 -0.873930
H -3.801274 -0.411282 0.071365
H -4.056363 0.226850 -1.571272
H -4.631103 -3.328654 -0.511138
H -3.015286 -3.946088 -0.925112
H -3.213182 -2.823157 0.445061
H -4.957402 -2.539857 -2.827476
H -3.882480 -1.367506 -3.592163
H -3.339643 -3.046196 -3.352703
H 1.905459 2.219391 -1.498368

Reaction 40

reactant

34
Energy: -748.344116842
C -4.061714 0.034855 0.189366
O -4.641920 0.075351 -0.886451
C -4.833178 -0.113876 1.505096
N -2.704277 0.105129 0.253584
H -2.206041 0.086261 1.131346
C -1.933926 0.228517 -0.974548
C -0.507371 0.174118 -0.699987
H -2.233882 -0.574910 -1.664470
H -2.198239 1.168183 -1.484618
C 0.678215 0.126473 -0.456463
C 2.075774 0.061368 -0.174415
C 2.677582 -1.162963 0.140536
C 4.039836 -1.207362 0.414452
C 4.812524 -0.054852 0.378443
C 4.214303 1.166206 0.063289
C 2.852294 1.227462 -0.210617
H 2.070094 -2.064814 0.164826
H 4.509951 -2.158186 0.659314
H 5.879690 -0.073287 0.588851
O 5.012714 2.262428 0.040655
H 2.378252 2.178631 -0.456818
C -3.958457 -0.080278 2.754231
C -5.850395 1.026590 1.560944
C -5.569487 -1.453377 1.431661
H -4.589391 -0.179205 3.647905
H -3.238131 -0.911276 2.778914
H -3.410906 0.868799 2.850304
H -6.210623 -1.581675 2.315139
H -6.194697 -1.498347 0.532526
H -4.862590 -2.294790 1.400284
H -6.501641 0.909416 2.438458

H -5.350981 2.002844 1.637549
H -6.470978 1.031064 0.657913
H 4.483834 3.037205 -0.195440

I_c

69
Energy: -4348.96923192
Cu 0.697982 0.084341 -0.533682
Cl 1.322165 -0.452270 -2.555969
C 1.929163 1.525199 -0.725613
O -0.593066 -1.458908 -0.392276
S -1.423373 -1.880734 0.791653
O -2.145108 -0.773070 1.425532
C -2.734830 -2.838437 -0.059944
O -0.764451 -2.842853 1.665338
F -3.611373 -3.280728 0.825590
F -3.378146 -2.055077 -0.934130
F -2.214441 -3.860253 -0.714502
C 1.356120 2.758667 -0.485355
C 2.214417 3.857194 -0.370327
C 3.584884 3.705612 -0.526256
C 4.119564 2.448473 -0.798391
C 3.286885 1.336491 -0.906713
C -1.039594 2.999031 2.128484
O -0.157521 3.850987 2.103916
C -2.513903 3.401410 2.056525
N -0.755420 1.669895 2.247469
C 0.604166 1.241990 2.422847
C 1.091163 0.198961 1.498382
C 1.799619 -0.780390 1.229076
C 2.696052 -1.818449 0.865932
C 2.227190 -3.102321 0.564295
C 3.142468 -4.087557 0.207491
C 4.507553 -3.795383 0.166732
C 4.966744 -2.519397 0.481451
C 4.073089 -1.519817 0.826989
H 0.287356 2.894891 -0.346494
H 1.779405 4.825642 -0.129484
H 4.241786 4.569188 -0.439668
H 5.191876 2.323798 -0.943371
H 3.693983 0.356136 -1.147023
H -1.468432 0.952798 2.132803
H 1.230521 2.139515 2.305214
H 0.767419 0.855578 3.441715
H 1.165093 -3.330799 0.615540
O 2.651590 -5.313085 -0.081126
H 5.217058 -4.576412 -0.111270
H 6.034174 -2.311156 0.451980
H 4.412483 -0.517543 1.079759
C -3.486364 2.235717 2.194990
C -2.750531 4.401829 3.190638
C -2.721846 4.097877 0.710121
O -3.306412 1.144424 -1.025029
C -4.526634 0.698902 -1.635316
C -5.546187 0.599081 -0.531684
C -2.110333 0.896484 -1.545864
O -1.137971 1.243133 -0.889836
C -2.028045 0.234091 -2.882875
H -4.818442 1.427365 -2.405522
H -4.374014 -0.274606 -2.115128
H -6.508063 0.256221 -0.929875
H -5.201774 -0.114818 0.227239
H -5.693702 1.572775 -0.049492
H -2.777185 0.625676 -3.579775
H -1.022939 0.364955 -3.288607
H -2.197408 -0.843502 -2.758855
H -3.362241 1.707966 3.151446
H -4.515946 2.621105 2.168508
H -3.382494 1.510127 1.377694
H -3.756001 4.464374 0.633008
H -2.039514 4.950748 0.608036
H -2.543584 3.402362 -0.120498
H -3.777173 4.790817 3.136660
H -2.621421 3.926376 4.173426
H -2.049709 5.241404 3.121628
H 3.375722 -5.905750 -0.326034

I_1

55
Energy: -4041.44750757

Cu -0.005844 0.125623 0.008765
Cl 0.003518 0.320418 2.132896
C 1.859553 0.195621 0.315099
O -2.025797 0.306011 -0.272778
S -2.052867 1.685895 -0.891970
O -2.722169 1.775532 -2.175435
C -3.073526 2.639863 0.296007
O -0.689917 2.251630 -0.781710
F -3.132802 3.900711 -0.100452
F -4.294478 2.130485 0.332491
F -2.535919 2.581672 1.500669
C 2.620327 -0.949324 0.440717
C 4.005338 -0.794536 0.356899
C 4.569932 0.466736 0.183200
C 3.759666 1.593904 0.095934
C 2.371811 1.472649 0.185364
H 2.167688 -1.932937 0.572818
H 4.639660 -1.676367 0.434436
H 5.652216 0.572503 0.131538
H 4.198639 2.583220 -0.023584
H 1.724250 2.347606 0.150242
C -0.440394 -3.206763 0.691330
O 0.624475 -3.551061 0.190734
C -0.817282 -3.576459 2.123927
N -1.367439 -2.535791 -0.057180
C -1.073907 -2.224155 -1.435834
C -0.110780 -1.121340 -1.638615
C 0.611885 -0.317889 -2.227399
C 1.548435 0.534887 -2.871868
C 1.164243 1.811242 -3.295913
C 2.106661 2.630541 -3.911073
C 3.413152 2.174321 -4.098036
C 3.782367 0.898995 -3.679695
C 2.859208 0.068094 -3.066784
H -2.150721 -2.062397 0.375446
H -0.656561 -3.118269 -1.916220
H -2.010498 -1.955138 -1.937441
H 0.152261 2.174376 -3.129260
O 1.698238 3.857843 -4.305547
H 4.146111 2.824702 -4.578403
H 4.804536 0.558964 -3.833241
H 3.132411 -0.925263 -2.718272
C -2.037603 -2.827824 2.652907
C -1.109994 -5.081600 2.099316
C 0.392565 -3.304388 3.017274
H 2.434295 4.329141 -4.719927
H 0.183811 -3.647538 4.039905
H 1.275631 -3.835982 2.643996
H 0.623504 -2.230798 3.057119
H -1.347462 -5.430708 3.113791
H -1.968547 -5.310625 1.451722
H -0.240816 -5.641001 1.732057
H -2.209950 -3.108465 3.700499
H -1.896357 -1.737657 2.625322
H -2.953500 -3.087122 2.101636

TS_{rc}^{A}

55
Energy: -4041.42600955
Cu -0.442459 -0.499652 -0.824007
Cl -0.052383 -1.163016 -2.819561
C -1.957259 -1.608924 -0.902533
O 0.877864 0.943225 -0.876483
S 1.643173 1.719645 0.162275
O 2.572813 0.885537 0.935608
C 2.725087 2.719608 -0.929186
O 0.838555 2.673268 0.914547
F 3.504882 3.483160 -0.176589
F 3.485072 1.924273 -1.669037
F 1.994894 3.487232 -1.718809
C -1.918053 -2.897157 -0.400374
C -3.108546 -3.622339 -0.353915
C -4.294478 -3.060952 -0.818409
C -4.297155 -1.771730 -1.340363
C -3.116158 -1.032317 -1.394094
H -0.985582 -3.328027 -0.040474
H -3.101564 -4.634843 0.047964
H -5.218537 -3.635487 -0.782794
H -5.219508 -1.331560 -1.716487
H -3.115945 -0.022967 -1.806310
C 2.008961 -1.789798 1.203563

O 0.919761 -1.910712 0.608729
C 3.319772 -2.207104 0.578654
N 1.943366 -1.387233 2.490766
C 0.607080 -1.009332 2.921061
C -0.232199 -0.646161 1.767572
C -1.035439 -0.049530 1.001729
C -2.215115 0.805748 1.109748
C -2.231585 2.090105 0.571585
C -3.408608 2.835391 0.632121
C -4.561233 2.294439 1.202121
C -4.531941 1.005730 1.723287
C -3.366799 0.253414 1.685819
H 2.736439 -0.887278 2.874544
H 0.147533 -1.837902 3.479635
H 0.668464 -0.135002 3.582733
H -1.335628 2.536827 0.144384
O -3.372985 4.083853 0.109918
H -5.479304 2.883068 1.235374
H -5.433925 0.583337 2.162612
H -3.346120 -0.770378 2.056280
C 3.359920 -1.742093 -0.876877
C 4.523272 -1.663225 1.342545
C 3.319438 -3.743312 0.633058
H 4.249705 -4.118092 0.186233
H 3.265668 -4.111009 1.667561
H 2.475682 -4.157730 0.067527
H 5.441861 -1.987831 0.837305
H 4.520038 -0.565655 1.357679
H 4.572761 -2.049159 2.371571
H 4.272521 -2.131410 -1.347927
H 2.495516 -2.106555 -1.445527
H 3.374001 -0.648017 -0.943278
H -4.242050 4.497057 0.207047

TS_{ar}^{B}

55
Energy: -4041.43735347
Cu -0.012994 -0.727104 -0.522038
Cl 0.375401 -1.078748 -2.625163
C -1.802103 -1.471714 -0.681314
O 1.707524 0.208177 -0.310698
S 1.889648 1.639812 0.138997
O 2.592133 1.716622 1.423854
C 3.101806 2.206238 -1.114610
O 0.696611 2.458810 -0.038310
F 3.449343 3.456783 -0.854565
F 4.180595 1.434784 -1.064973
F 2.566827 2.136788 -2.318035
C -2.001964 -2.696278 -0.059187
C -3.055063 -3.499592 -0.494760
C -3.893680 -3.062957 -1.513973
C -3.682621 -1.821496 -2.112876
C -2.630757 -1.014891 -1.700811
H -1.340980 -3.024410 0.739512
H -3.217717 -4.467528 -0.023908
H -4.720059 -3.690158 -1.843482
H -4.331874 -1.481234 -2.917433
H -2.457504 -0.045603 -2.164492
C 1.960083 -1.920058 1.259249
O 0.973835 -2.449428 0.727901
C 3.370309 -2.320338 0.881304
N 1.796278 -0.980992 2.225243
C 0.467162 -0.480691 2.521330
C -0.427909 -0.307474 1.343360
C -1.534060 -0.022231 0.816847
C -2.723752 0.799179 0.741799
C -2.561028 2.163836 0.503775
C -3.690938 2.982616 0.480504
C -4.955134 2.435330 0.697025
C -5.097861 1.069252 0.930181
C -3.990603 0.237126 0.945533
H 2.535524 -0.287157 2.315678
C -0.241244 -1.365358 3.544934
H 0.613487 0.528907 2.936476
H -1.570630 2.587252 0.336316
O -3.492999 4.299292 0.246426
H -5.833821 3.082023 0.680360
H -6.090829 0.656189 1.096215
H -4.088600 -0.835177 1.105693
C 4.462037 -1.421997 1.447991
H 3.481992 -3.329358 1.317236

C 3.486538 -2.447504 -0.636344
H 5.444149 -1.801757 1.141790
H 4.376551 -0.396042 1.060613
H 4.455685 -1.386115 2.545480
H 4.460245 -2.879971 -0.897784
H 2.696715 -3.083853 -1.048363
H 3.403575 -1.461906 -1.112040
H -1.230561 -0.962839 3.792149
H -0.360539 -2.378825 3.145821
H 0.361806 -1.418028 4.458213
H -4.342945 4.761274 0.247210

I_2

55
Energy: -4041.49040134
Cu 0.269951 -1.430954 -1.963374
Cl 0.929704 -3.481987 -2.443223
C 2.064996 0.393633 -0.691504
O -0.122693 -1.744114 -0.151644
S -1.640109 -1.705706 0.053525
O -2.335428 -1.917132 -1.220120
C -1.880392 -3.237983 1.035858
O -2.074752 -0.596897 0.889160
F -3.171960 -3.358416 1.305817
F -1.473925 -4.283917 0.347815
F -1.201799 -3.151011 2.166026
C 2.688233 -0.451327 -1.619683
C 3.788702 -1.219461 -1.247113
C 4.286457 -1.134879 0.045758
C 3.691971 -0.269282 0.964524
C 2.590526 0.491408 0.600930
H 2.364088 -0.450315 -2.661927
H 4.245596 -1.887539 -1.974203
H 5.141784 -1.739177 0.341838
H 4.084091 -0.199692 1.977718
H 2.105357 1.144154 1.325656
C -1.116524 -0.672999 -4.135722
O 0.039185 -1.029796 -3.778742
C -1.708624 -1.349820 -5.356834
N -1.840808 0.206941 -3.453870
C -1.464174 0.828794 -2.186158
C -0.134250 0.406125 -1.691133
C 0.813150 1.095183 -1.050152
C 0.641749 2.510086 -0.645369
C -0.525664 2.916645 0.000636
C -0.682005 4.249897 0.380603
C 0.327920 5.174225 0.118949
C 1.497652 4.758409 -0.510997
C 1.665807 3.434299 -0.889608
H -2.792861 0.362006 -3.760706
H -1.440874 1.921425 -2.330441
H -2.260334 0.600690 -1.461865
H -1.299543 2.196905 0.268129
O -1.836610 4.583847 1.005947
H 0.202705 6.215973 0.418411
H 2.285968 5.483446 -0.706052
H 2.581556 3.106116 -1.378758
C -2.894495 -0.595298 -5.949888
C -0.615699 -1.521681 -6.411257
C -2.158423 -2.729499 -4.844501
H -2.582567 -3.301439 -5.680897
H -1.312941 -3.290457 -4.424007
H -2.924408 -2.636546 -4.061893
H -1.022298 -2.084443 -7.261445
H -0.257606 -0.552625 -6.784944
H 0.237303 -2.075052 -6.004216
H -3.222305 -1.108020 -6.862817
H -3.767073 -0.580011 -5.279605
H -2.635373 0.436477 -6.226048
H -1.807779 5.518024 1.255348

TS_{rc}^{B}

55
Energy: -4041.46222735
Cu -0.772536 1.313771 0.228785
Cl -2.263194 2.666542 -0.663563
C 0.340224 -1.589070 1.721041
O 0.953568 1.672349 0.946074
S 2.055552 1.656027 -0.104845
O 1.523310 1.369607 -1.439115

C 2.506072 3.432292 -0.146915
O 3.239172 0.938268 0.339895
F 3.459084 3.622972 -1.046535
F 1.443410 4.149674 -0.477101
F 2.942637 3.817732 1.041105
C -0.187040 -0.648654 2.619061
C -0.102271 -0.845492 3.986481
C 0.513901 -1.987485 4.492614
C 1.046152 -2.927752 3.616549
C 0.962787 -2.731661 2.244987
H -0.645511 0.258156 2.236575
H -0.508042 -0.094864 4.661671
H 0.586171 -2.138164 5.568259
H 1.534784 -3.820502 4.002496
H 1.381088 -3.473679 1.567681
C -3.018437 -0.344025 -0.564470
O -2.292293 -0.233719 0.459115
C -4.524360 -0.378639 -0.434347
N -2.421841 -0.539576 -1.740190
C -0.970834 -0.399675 -1.781645
C -0.485470 -0.493300 -0.373707
C 0.226575 -1.428762 0.269482
C 1.004717 -2.347061 -0.618214
C 2.366228 -2.141935 -0.813477
C 3.079764 -3.033955 -1.617165
C 2.431840 -4.113948 -2.218131
C 1.068873 -4.304580 -2.017090
C 0.345558 -3.425647 -1.220761
H -2.952615 -0.432723 -2.595338
H -0.526832 -1.221585 -2.359243
H -0.692608 0.551224 -2.257857
H 2.882193 -1.292092 -0.362797
O 4.400846 -2.798497 -1.777244
H 2.997552 -4.805368 -2.844553
H 0.571381 -5.153355 -2.483389
H -0.720859 -3.573014 -1.046975
C -4.942797 0.543289 0.710156
C -5.203599 0.059873 -1.729922
C -4.891721 -1.832142 -0.103796
H -6.288391 0.098248 -1.570351
H -4.873912 1.062712 -2.034225
H -5.036395 -0.647491 -2.556074
H -5.978674 -1.907305 0.031914
H -4.599690 -2.517557 -0.911559
H -4.406742 -2.160058 0.824506
H -6.035407 0.514568 0.812383
H -4.497597 0.224475 1.659338
H -4.628215 1.576791 0.516599
H 4.780224 -3.465870 -2.365824

Reaction 41

reactant

34
Energy: -748.344933334
C -4.061454 -0.006269 0.184221
O -4.646261 -0.011813 -0.890133
C -4.829789 -0.089042 1.507895
N -2.704201 0.060148 0.241152
H -2.202875 0.074828 1.117224
C -1.933599 0.122476 -0.992408
C -0.506733 0.097338 -0.717041
H -2.226293 -0.719661 -1.637907
H -2.208521 1.030397 -1.551882
C 0.680093 0.075083 -0.472873
C 2.076947 0.041844 -0.190402
C 2.741890 -1.181245 -0.004386
C 4.096620 -1.221537 0.271786
C 4.820303 -0.031446 0.367495
C 4.176843 1.193088 0.184948
C 2.818288 1.226385 -0.090800
H 2.173684 -2.106523 -0.081694
H 4.620489 -2.163779 0.417064
O 6.143525 -0.128074 0.639590
H 4.744058 2.122280 0.259131
H 2.314251 2.180455 -0.234248
C -3.950362 -0.013357 2.751857
C -5.831631 1.066322 1.517941
C -5.584040 -1.420324 1.494613
H -4.579169 -0.068228 3.650881
H -3.238775 -0.850283 2.808431

H -3.392286 0.932832 2.806765
H -6.224421 -1.501628 2.384237
H -6.212033 -1.495936 0.599512
H -4.888426 -2.271683 1.498246
H -6.480917 0.996359 2.401992
H -5.318756 2.038086 1.550165
H -6.455635 1.040014 0.617607
H 6.528193 0.758812 0.677394

I$_c$

69
Energy: -4348.97554269
Cu 0.689735 0.104434 -0.525560
Cl 1.341398 -0.454741 -2.541865
C 1.878701 1.582566 -0.646029
O -0.572589 -1.464483 -0.394687
S -1.417418 -1.880978 0.778769
O -2.156384 -0.775236 1.394640
C -2.708576 -2.860003 -0.079655
O -0.764885 -2.831873 1.672107
F -3.593247 -3.299918 0.799092
F -3.346137 -2.094938 -0.972730
F -2.169218 -3.886069 -0.713386
C 1.285593 2.793885 -0.345911
C 2.107958 3.921199 -0.262640
C 3.472110 3.819524 -0.497661
C 4.031736 2.585337 -0.819220
C 3.232203 1.446478 -0.898081
C -0.990389 2.989702 2.259852
O -0.090800 3.808496 2.414075
C -2.440257 3.443776 2.072330
N -0.745477 1.648506 2.222994
C 0.583273 1.153716 2.453242
C 1.091452 0.175525 1.463889
C 1.839066 -0.798974 1.251350
C 2.713136 -1.837212 0.898005
C 2.244486 -3.156738 0.704170
C 3.123173 -4.151944 0.339292
C 4.481474 -3.856467 0.166519
C 4.962939 -2.556015 0.368832
C 4.084739 -1.554478 0.726174
H 0.222050 2.884837 -0.144176
H 1.655144 4.871533 0.015787
H 4.103984 4.703460 -0.432424
H 5.097728 2.501157 -1.027153
H 3.657051 0.486675 -1.185988
H -1.477930 0.967770 2.035073
H 1.244591 2.033407 2.453488
H 0.662069 0.679314 3.444246
H 1.185592 -3.363477 0.853552
H 2.789944 -5.174071 0.176994
O 5.286029 -4.871145 -0.189405
H 6.024424 -2.342325 0.240677
H 4.440406 -0.538081 0.888178
C -3.462060 2.314330 2.155479
C -2.735340 4.482603 3.154614
C -2.509857 4.107198 0.693412
O -3.331323 1.111003 -1.066253
C -4.542236 0.643895 -1.677262
C -5.559202 0.516640 -0.573576
C -2.129121 0.875670 -1.581590
O -1.164508 1.236317 -0.923312
C -2.028961 0.208889 -2.915500
H -4.849899 1.370120 -2.443513
H -4.370938 -0.323495 -2.163296
H -6.513802 0.155062 -0.972791
H -5.199508 -0.192828 0.182149
H -5.727280 1.483666 -0.085379
H -2.768968 0.598405 -3.623524
H -1.017549 0.339778 -3.306250
H -2.199033 -0.868576 -2.792555
H -3.410178 1.785494 3.117947
H -4.473332 2.737469 2.068193
H -3.343931 1.581306 1.346690
H -3.517418 4.513145 0.521898
H -1.786481 4.930561 0.625161
H -2.294343 3.379964 -0.101242
H -3.735078 4.911516 2.997583
H -2.715753 4.030211 4.156207
H -1.994929 5.289613 3.131534
H 6.194921 -4.553046 -0.293910

I_1

55
Energy: -4041.45330541
Cu 0.048503 0.035216 0.099921
Cl -0.051347 0.409166 2.198525
C 1.909998 0.021433 0.399439
O -1.925973 0.275149 -0.305288
S -1.906500 1.614283 -1.011955
O -2.475663 1.605400 -2.349453
C -3.034549 2.621299 0.025034
O -0.568582 2.211753 -0.843105
F -3.069186 3.856098 -0.450865
F -4.250386 2.099958 -0.013529
F -2.601770 2.641160 1.271404
C 2.620362 -1.143606 0.615101
C 4.013277 -1.045520 0.624786
C 4.640697 0.185082 0.445075
C 3.883385 1.335674 0.253704
C 2.488661 1.267116 0.239604
H 2.124816 -2.105995 0.749509
H 4.604065 -1.947466 0.778351
H 5.727426 0.247800 0.469408
H 4.368285 2.302980 0.129595
H 1.883595 2.165103 0.116740
C -0.534038 -3.230854 0.822818
O 0.551514 -3.691341 0.488026
C -1.119962 -3.477886 2.211133
N -1.294318 -2.526227 -0.070001
C -0.804717 -2.298084 -1.407903
C 0.074381 -1.110000 -1.559661
C 0.741680 -0.335554 -2.261714
C 1.539048 0.594586 -2.940953
C 0.958321 1.732414 -3.544565
C 1.751865 2.642586 -4.206916
C 3.136677 2.439748 -4.270614
C 3.726983 1.321392 -3.667592
C 2.932694 0.405439 -3.009001
H -2.107306 -2.001256 0.228180
H -0.238935 -3.186004 -1.714802
H -1.661845 -2.170311 -2.080278
H -0.117527 1.881902 -3.466186
H 1.333376 3.527809 -4.679304
O 3.857436 3.363715 -4.928221
H 4.807377 1.182231 -3.716891
H 3.373935 -0.463457 -2.523040
C -2.335299 -2.612508 2.533680
C -1.520255 -4.957645 2.237336
C -0.014720 -3.225769 3.236791
H 4.796266 3.126337 -4.909202
H -0.373844 -3.488355 4.241418
H 0.868740 -3.833696 3.010340
H 0.282931 -2.167874 3.246194
H -1.906584 -5.220802 3.231775
H -2.307645 -5.171246 1.500111
H -0.657342 -5.598038 2.017044
H -2.661291 -2.816089 3.562423
H -2.106596 -1.538428 2.470848
H -3.191644 -2.839015 1.881241

TS_{rc}^A

55
Energy: -4041.42729126
Cu -0.324920 -0.527517 -0.856894
Cl 0.152997 -1.235208 -2.819552
C -1.801037 -1.689848 -0.932016
O 0.921219 0.982658 -0.919042
S 1.616818 1.817522 0.124521
O 2.538260 1.039021 0.963019
C 2.713287 2.816315 -0.953765
O 0.749638 2.769714 0.805246
F 3.455814 3.609225 -0.193873
F 3.509750 2.023083 -1.656034
F 1.992143 3.555296 -1.779419
C -1.740421 -2.959159 -0.385560
C -2.909189 -3.719746 -0.351251
C -4.096379 -3.211529 -0.869856
C -4.121351 -1.940514 -1.434649
C -2.962100 -1.166948 -1.476828
H -0.807402 -3.349716 0.016687

H -2.883810 -4.718038 0.084137
H -5.003156 -3.813530 -0.843756
H -5.043427 -1.543520 -1.856779
H -2.980415 -0.173845 -1.926202
C 2.091618 -1.652885 1.296573
O 1.034494 -1.840178 0.662662
C 3.444560 -2.023613 0.735894
N 1.954408 -1.224280 2.570392
C 0.581934 -0.913876 2.936239
C -0.217372 -0.596026 1.742779
C -0.998282 -0.034555 0.925985
C -2.192868 0.799321 0.967824
C -2.279253 1.999995 0.252120
C -3.458110 2.724960 0.243807
C -4.576924 2.250533 0.931002
C -4.504959 1.046802 1.631582
C -3.317834 0.328504 1.649887
H 2.702466 -0.670466 2.970372
H 0.141640 -1.764423 3.477243
H 0.567039 -0.036682 3.596925
H -1.402432 2.374593 -0.271958
H -3.536061 3.666481 -0.295059
O -5.704953 2.997430 0.875138
H -5.381398 0.667446 2.159002
H -3.268745 -0.631492 2.163568
C 3.519954 -1.599104 -0.730301
C 4.589555 -1.402261 1.530129
C 3.513944 -3.555987 0.836977
H 4.477684 -3.898096 0.437337
H 3.437001 -3.896016 1.879458
H 2.713522 -4.026497 0.252721
H 5.542004 -1.695338 1.070377
H 4.534541 -0.306178 1.515027
H 4.615487 -1.758370 2.570678
H 4.470083 -1.951806 -1.153480
H 2.699434 -2.025826 -1.320215
H 3.478298 -0.508301 -0.829424
H -6.401838 2.565872 1.388872

TS_{ar}^{B}

55
Energy: -4041.44005227
Cu -0.006250 -0.727787 0.464551
Cl -0.388049 -1.176492 2.552863
C 1.695632 -1.670062 0.486758
O -1.555595 0.516551 0.371612
S -1.534946 1.958910 -0.069315
O -2.137663 2.138575 -1.395247
C -2.746955 2.658519 1.115222
O -0.270510 2.636459 0.195517
F -2.912602 3.947649 0.864489
F -3.911784 2.035076 0.976317
F -2.311869 2.501002 2.350248
C 1.726082 -2.859865 -0.230267
C 2.651186 -3.836902 0.133015
C 3.538938 -3.604662 1.177777
C 3.505199 -2.395287 1.871839
C 2.580199 -1.417934 1.531446
H 1.028202 -3.029833 -1.047688
H 2.676898 -4.778160 -0.413066
H 4.265438 -4.367233 1.452633
H 4.191768 -2.215481 2.697077
H 2.543209 -0.474044 2.071571
C -2.196586 -1.517742 -1.256892
O -1.308976 -2.212661 -0.742226
C -3.661253 -1.699093 -0.898396
N -1.870943 -0.613516 -2.218044
C -0.474472 -0.421097 -2.511993
C 0.443250 -0.243446 -1.358729
C 1.595072 -0.096479 -0.861421
C 2.834634 0.627936 -0.769243
C 2.787251 2.025633 -0.634731
C 3.959509 2.758886 -0.611057
C 5.189470 2.107562 -0.724649
C 5.243106 0.714764 -0.854181
C 4.072437 -0.020890 -0.865407
H -2.444358 0.225942 -2.282987
H -0.098438 -1.290676 -3.071725
H -0.381787 0.460509 -3.160097
H 1.818667 2.514438 -0.531344
H 3.948288 3.840718 -0.501374

O 6.299304 2.872870 -0.699676
H 6.207630 0.213254 -0.942168
H 4.107932 -1.105576 -0.958250
C -4.558941 -0.605725 -1.470678
C -4.060372 -3.056766 -1.493167
C -3.798056 -1.742321 0.624463
H -5.111513 -3.266586 -1.253173
H -3.954198 -3.062088 -2.587628
H -3.441939 -3.861894 -1.078064
H -4.828923 -2.018747 0.885437
H -3.114756 -2.476957 1.065936
H -3.574055 -0.765668 1.070864
H -5.595449 -0.796932 -1.163166
H -4.287299 0.391205 -1.095278
H -4.544549 -0.589263 -2.569802
H 7.084907 2.313212 -0.780595

I_2

55
Energy: -4041.49158395
Cu 0.269137 -1.433125 -1.965263
Cl 0.924478 -3.492406 -2.418854
C 2.072514 0.397294 -0.713980
O -0.134119 -1.723581 -0.150687
S -1.651235 -1.677515 0.049405
O -2.345310 -1.896639 -1.223476
C -1.901411 -3.199718 1.044597
O -2.084666 -0.560187 0.875178
F -3.194310 -3.311605 1.312260
F -1.498186 -4.253532 0.366919
F -1.225449 -3.106039 2.175861
C 2.690145 -0.452848 -1.641607
C 3.793105 -1.219000 -1.272581
C 4.299883 -1.127646 0.016361
C 3.711373 -0.257411 0.934578
C 2.607472 0.501386 0.574350
H 2.359217 -0.457264 -2.681647
H 4.245033 -1.890892 -1.999270
H 5.157274 -1.730350 0.309739
H 4.109901 -0.183000 1.944955
H 2.126950 1.157449 1.299104
C -1.112002 -0.690884 -4.149314
O 0.039039 -1.055378 -3.786685
C -1.707707 -1.374943 -5.364841
N -1.829953 0.203161 -3.478670
C -1.453536 0.832190 -2.213936
C -0.125843 0.408688 -1.713968
C 0.819871 1.099115 -1.069253
C 0.639355 2.506688 -0.655142
C -0.528930 2.922141 -0.001950
C -0.697036 4.240960 0.389668
C 0.310085 5.172218 0.134956
C 1.488598 4.769979 -0.497210
C 1.650610 3.446751 -0.879066
H -2.779763 0.362681 -3.790066
H -1.422782 1.923468 -2.367805
H -2.254946 0.613438 -1.492452
H -1.292552 2.183614 0.244872
H -1.592739 4.569251 0.912471
O 0.094956 6.447907 0.533304
H 2.280523 5.496721 -0.685367
H 2.573839 3.132272 -1.365443
C -2.888937 -0.619294 -5.965582
C -0.615188 -1.562355 -6.417054
C -2.165461 -2.747314 -4.840249
H -2.591454 -3.325179 -5.671619
H -1.323463 -3.308396 -4.412991
H -2.932055 -2.642747 -4.059705
H -1.024243 -2.130619 -7.262403
H -0.251718 -0.598649 -6.799339
H 0.234599 -2.116510 -6.004416
H -3.219567 -1.138865 -6.873615
H -3.761562 -0.592392 -5.295715
H -2.623681 0.408206 -6.251751
H 0.869769 6.986581 0.319805

TS_{rc}^{B}

55
Energy: -4041.46368278
Cu -1.025355 1.182465 0.163160

Cl -2.732114 2.134094 -0.853983
C 0.614347 -1.277192 1.899888
O 0.574781 1.960890 0.849054
S 1.693846 2.091330 -0.173655
O 1.307992 1.542892 -1.475863
C 1.722337 3.907577 -0.418301
O 2.992602 1.728481 0.371906
F 2.642104 4.214140 -1.320750
F 0.537514 4.314409 -0.846629
F 2.008322 4.515149 0.721570
C -0.135771 -0.404262 2.702332
C -0.063321 -0.467038 4.083076
C 0.764179 -1.403133 4.698920
C 1.518730 -2.272947 3.918766
C 1.446900 -2.210999 2.533819
H -0.764953 0.347131 2.234218
H -0.646544 0.229223 4.682283
H 0.825944 -1.447136 5.784810
H 2.172250 -3.004492 4.390533
H 2.038720 -2.897231 1.931053
C -2.845810 -0.958275 -0.559542
O -2.202775 -0.626802 0.470870
C -4.315482 -1.303233 -0.469123
N -2.173498 -1.104828 -1.702036
C -0.784798 -0.659471 -1.720827
C -0.339800 -0.557686 -0.299763
C 0.528413 -1.264778 0.436838
C 1.532442 -2.032728 -0.355555
C 2.839387 -1.553261 -0.506434
C 3.773489 -2.286467 -1.219361
C 3.414350 -3.507922 -1.793088
C 2.112392 -3.990883 -1.652960
C 1.178381 -3.250313 -0.938755
H -2.681112 -1.167873 -2.575427
H -0.155961 -1.396744 -2.236043
H -0.698755 0.298302 -2.253628
H 3.113343 -0.590483 -0.070861
H 4.791030 -1.926158 -1.352869
O 4.370896 -4.178810 -2.475919
H 1.833970 -4.948664 -2.095314
H 0.161131 -3.627713 -0.819660
C -4.964842 -0.427501 0.601174
C -5.018075 -1.091683 -1.808600
C -4.384059 -2.780299 -0.056501
H -6.093253 -1.269188 -1.681187
H -4.888610 -0.061857 -2.168808
H -4.677103 -1.796101 -2.582162
H -5.435832 -3.075566 0.053732
H -3.922774 -3.433384 -0.810321
H -3.879345 -2.945168 0.904038
H -6.029608 -0.683812 0.676586
H -4.499846 -0.586774 1.580529
H -4.870401 0.636043 0.347710
H 4.002145 -5.002442 -2.824509

Reaction 42

reactant

33
Energy: -772.364114804
C -4.052724 -0.355889 0.241834
O -4.640430 -0.806085 -0.731683
C -4.814648 0.128100 1.479848
N -2.694788 -0.269410 0.257504
H -2.186548 0.097723 1.049001
C -1.937581 -0.718908 -0.898942
C -0.512993 -0.537324 -0.675985
H -2.173489 -1.775209 -1.103096
H -2.270764 -0.166625 -1.791680
C 0.665438 -0.371537 -0.454513
C 2.053271 -0.181086 -0.205415
C 3.028473 -0.498595 -1.161771
C 4.376687 -0.304078 -0.895726
C 4.777995 0.212813 0.333868
C 3.830670 0.536080 1.299728
C 2.491542 0.335797 1.017957
H 2.702166 -0.901631 -2.118511
H 5.118204 -0.556589 -1.650655
H 5.834316 0.366463 0.545357
H 4.109143 0.941331 2.269562
F 1.581485 0.645824 1.943993

C -3.924022 0.658597 2.598797
C -5.763400 1.233420 1.012449
C -5.628194 -1.061577 1.992878
H -4.547934 0.982521 3.442804
H -3.239068 -0.111118 2.983670
H -3.335730 1.532088 2.281513
H -6.269347 -0.748614 2.828912
H -6.260832 -1.466174 1.194701
H -4.972287 -1.867402 2.351891
H -6.405443 1.556944 1.843770
H -5.206648 2.110485 0.652940
H -6.398395 0.873357 0.195043

I_c

68
Energy: -4372.98735728
Cu 0.604790 -0.025291 -0.497843
Cl 1.317834 -0.751257 -2.449552
C 1.821791 1.440996 -0.649471
O -0.739918 -1.511413 -0.328305
S -1.753170 -1.702119 0.767251
O -2.826963 -0.698013 0.728847
C -2.559415 -3.251467 0.207052
O -1.187386 -1.994710 2.078242
F -3.481805 -3.603516 1.088403
F -3.149884 -3.053154 -0.971379
F -1.673488 -4.222757 0.087861
C 1.254025 2.605301 -0.169398
C 2.068281 3.736951 -0.079918
C 3.398233 3.682578 -0.475190
C 3.929029 2.492877 -0.963558
C 3.138562 1.345839 -1.054947
C -1.055623 2.752332 2.372898
O -0.086576 3.433229 2.684922
C -2.415942 3.395938 2.098035
N -0.957784 1.395150 2.216513
C 0.254398 0.717639 2.590383
C 0.979955 -0.022676 1.531168
C 1.850178 -0.838250 1.193580
C 2.863747 -1.721542 0.766243
C 2.561192 -3.047281 0.412628
C 3.542051 -3.919785 -0.023430
C 4.855402 -3.471081 -0.100569
C 5.191392 -2.163481 0.254856
C 4.203304 -1.294058 0.682037
H 0.218578 2.659719 0.152330
H 1.639507 4.650040 0.330403
H 4.024917 4.569679 -0.403443
H 4.965299 2.445933 -1.296014
H 3.544452 0.423376 -1.462738
H -1.768191 0.836400 1.960376
H 0.926086 1.494271 2.983161
H 0.051472 -0.015838 3.383809
F 1.313137 -3.466074 0.495307
H 3.260461 -4.933321 -0.296164
H 5.630085 -4.154240 -0.443552
H 6.224074 -1.828634 0.194739
H 4.435452 -0.266959 0.958376
C -3.569152 2.399950 2.052796
C -2.668824 4.426406 3.197995
C -2.294341 4.107545 0.746072
O -3.279914 1.398315 -1.250999
C -4.532325 0.850749 -1.690126
C -5.604952 1.433965 -0.809259
C -2.132916 0.827055 -1.596876
O -1.126269 1.192988 -1.001243
C -2.135089 -0.176314 -2.703349
H -4.688899 1.112199 -2.746817
H -4.497877 -0.242782 -1.594911
H -6.594779 1.128717 -1.167733
H -5.483523 1.082702 0.221710
H -5.553902 2.529979 -0.812290
H -2.796537 0.132086 -3.520604
H -1.115442 -0.314152 -3.071018
H -2.482963 -1.144517 -2.324130
H -3.663116 1.838993 2.993804
H -4.511068 2.946014 1.899262
H -3.468273 1.684945 1.225670
H -3.234517 4.629020 0.513707
H -1.484391 4.849247 0.771191
H -2.096890 3.390286 -0.062057

H -3.593982 4.979167 2.982987
H -2.783844 3.943393 4.178506
H -1.838211 5.137187 3.265341

I_1

54
Energy: -4065.46562022
Cu 0.021551 0.110914 -0.021193
Cl -0.005641 0.273333 2.108827
C 1.880806 0.094130 0.324121
O -1.969651 0.365811 -0.317241
S -1.961533 1.769180 -0.890904
O -2.761327 1.952934 -2.083328
C -2.792910 2.751991 0.418074
O -0.554518 2.231330 -0.888245
F -2.848822 4.017413 0.033162
F -4.020561 2.291342 0.597604
F -2.122651 2.669330 1.552997
C 2.596234 -1.083597 0.428702
C 3.988768 -0.980208 0.423400
C 4.608831 0.265419 0.348508
C 3.845406 1.425791 0.277783
C 2.451046 1.353208 0.283391
H 2.103861 -2.055319 0.491654
H 4.584823 -1.889382 0.487221
H 5.695425 0.330968 0.363025
H 4.325649 2.402133 0.235879
H 1.840038 2.254585 0.263286
C -0.511907 -3.231066 0.710985
O 0.549550 -3.638559 0.251984
C -0.960288 -3.570324 2.130118
N -1.371049 -2.513526 -0.074625
C -1.023758 -2.250885 -1.449881
C -0.076183 -1.134909 -1.663170
C 0.649100 -0.359166 -2.285779
C 1.511597 0.539350 -2.952914
C 0.981904 1.606973 -3.694059
C 1.802233 2.511085 -4.345239
C 3.180687 2.350840 -4.256674
C 3.736176 1.296244 -3.530953
C 2.907644 0.393850 -2.886337
H -2.154514 -2.009060 0.321332
H -0.573774 -3.158535 -1.871367
H -1.940930 -2.018077 -2.003659
F -0.329084 1.733282 -3.786687
H 1.348365 3.323392 -4.906656
H 3.830870 3.061011 -4.764274
H 4.815951 1.183336 -3.465893
H 3.317157 -0.429140 -2.302422
C -2.176036 -2.776456 2.600111
C 0.221024 -3.329027 3.069693
H -0.038525 -3.659966 4.084713
H 1.101525 -3.888987 2.733833
H 0.482781 -2.262750 3.113570
C -1.302666 -5.065101 2.108585
H -1.593792 -5.393832 3.115828
H -2.141370 -5.272644 1.428479
H -0.438872 -5.657631 1.783382
H -2.401371 -3.040540 3.641930
H -1.998263 -1.691940 2.568817
H -3.075811 -3.011352 2.012525

TS_{rc}^{A}

54
Energy: -4065.45021627
Cu 0.589750 0.505562 -0.650724
Cl 0.250234 1.147254 -2.662732
C 2.186576 1.507623 -0.724468
O -1.193829 -0.341015 -0.439589
S -1.685608 -1.669063 0.082885
O -2.600279 -1.502240 1.220422
C -2.764765 -2.169315 -1.310617
O -0.657182 -2.691973 0.196417
F -3.352675 -3.321609 -1.029179
F -3.696984 -1.243004 -1.506528
F -2.044092 -2.300589 -2.409910
C 2.169701 2.783566 -0.186487
C 3.356936 3.515618 -0.188058
C 4.519658 2.976366 -0.727719
C 4.499878 1.700278 -1.279115

C 3.321766 0.953988 -1.291311
H 1.259445 3.204802 0.233335
H 3.362718 4.518377 0.237283
H 5.440791 3.556500 -0.728584
H 5.401096 1.277217 -1.720653
H 3.299535 -0.027289 -1.759128
C -1.717766 1.746898 1.380702
O -0.549534 2.058193 1.076950
C -2.927885 2.336245 0.691405
N -1.871994 0.942557 2.460853
C -0.593410 0.555470 3.041572
C 0.409609 0.440398 1.976961
C 1.137484 -0.140913 1.116934
C 2.039758 -1.274955 1.174987
C 2.312623 -2.023451 0.029336
C 3.144757 -3.124883 0.033346
C 3.747972 -3.498712 1.228992
C 3.510378 -2.771766 2.393360
C 2.662662 -1.674441 2.364517
H -2.587771 0.217307 2.393725
H -0.298316 1.291082 3.802494
H -0.696670 -0.429791 3.517875
F 1.739187 -1.656891 -1.127839
H 3.300851 -3.672737 -0.892218
H 4.407957 -4.363389 1.249006
H 3.987217 -3.062024 3.326888
H 2.482412 -1.093553 3.268176
C -2.658435 2.499925 -0.801926
C -4.183080 1.496096 0.913315
C -3.108803 3.718602 1.341356
H -3.976160 4.216224 0.887607
H -3.288593 3.635898 2.422164
H -2.226244 4.350396 1.178502
H -5.021721 1.969718 0.386835
H -4.069399 0.478462 0.517609
H -4.460170 1.436458 1.975252
H -3.503122 3.031975 -1.259569
H -1.745255 3.078141 -0.989091
H -2.547636 1.528039 -1.297182

TS_{ar}^{B}

54
Energy: -4065.45108932
Cu -0.190889 0.777087 0.427433
Cl 0.221546 1.459332 2.443239
C -1.935787 1.606404 0.458200
O 1.450472 -0.306112 0.415671
S 1.353922 -1.810407 0.253663
O 1.819042 -2.235325 -1.069069
C 2.664669 -2.323832 1.429247
O 0.106528 -2.357363 0.770771
F 2.784323 -3.642079 1.402556
F 3.823068 -1.775822 1.075997
F 2.354063 -1.933100 2.650113
C -2.119517 2.685311 -0.395731
C -3.126990 3.602759 -0.104888
C -3.941893 3.417810 1.006766
C -3.754143 2.314939 1.838365
C -2.746267 1.395539 1.568679
H -1.479315 2.820014 -1.265273
H -3.274952 4.459488 -0.759734
H -4.731491 4.134001 1.226555
H -4.383098 2.173110 2.715445
H -2.587101 0.537925 2.219187
C 1.972578 1.550016 -1.378809
O 1.030290 2.231519 -0.954033
C 3.415183 1.867342 -1.025954
N 1.726457 0.535390 -2.253282
C 0.348976 0.238328 -2.543241
C -0.571438 0.125603 -1.389976
C -1.689849 -0.090809 -0.860591
C -2.818571 -0.976675 -0.758905
C -2.601489 -2.300201 -1.178469
C -3.610306 -3.247242 -1.154219
C -4.877826 -2.869405 -0.729860
C -5.133240 -1.552182 -0.351525
C -4.113145 -0.614100 -0.366930
H 2.341443 -0.274005 -2.217080
H -0.061646 1.018120 -3.201933
H 0.308246 -0.720112 -3.078734
F -1.413620 -2.644411 -1.654985

H -3.383182 -4.260235 -1.476027
H -5.676510 -3.608122 -0.707230
H -6.133068 -1.255824 -0.043223
H -4.309821 0.415492 -0.079325
C 4.390200 0.770819 -1.444556
C 3.741042 3.160804 -1.786552
C 3.520437 2.113533 0.479223
H 4.771722 3.467437 -1.562469
H 3.657040 3.019085 -2.873710
H 3.065077 3.970813 -1.486456
H 4.531764 2.471852 0.715890
H 2.795240 2.865201 0.811467
H 3.332638 1.193280 1.045955
H 5.407634 1.068668 -1.158866
H 4.180041 -0.182946 -0.940265
H 4.393992 0.609751 -2.531941

I_2

54
Energy: -4065.50973534
Cu 0.272145 -1.382474 -1.943615
Cl 0.980046 -3.420557 -2.406786
C 2.029085 0.436503 -0.655918
O -0.156205 -1.697773 -0.140730
S -1.679278 -1.784866 0.015168
O -2.315386 -1.891606 -1.303515
C -1.847673 -3.439390 0.792921
O -2.210723 -0.819248 0.959506
F -3.135808 -3.665433 1.009109
F -1.365280 -4.367489 -0.005477
F -1.200388 -3.455171 1.944875
C 2.673802 -0.390600 -1.585160
C 3.765465 -1.166552 -1.200931
C 4.231887 -1.107163 0.104435
C 3.615050 -0.259506 1.025699
C 2.522983 0.508537 0.650659
H 2.371827 -0.371239 -2.633917
H 4.238482 -1.821876 -1.929230
H 5.079567 -1.717684 0.409407
H 3.981995 -0.210675 2.049445
H 2.020859 1.146523 1.376988
C -1.137494 -0.675011 -4.105175
O 0.035913 -0.990152 -3.764640
C -1.737068 -1.392643 -5.298766
N -1.869106 0.201843 -3.427461
C -1.479901 0.862256 -2.185700
C -0.151984 0.449290 -1.680190
C 0.786329 1.147152 -1.036195
C 0.656374 2.574437 -0.669132
C -0.484128 3.093133 -0.053294
C -0.592355 4.425359 0.314640
C 0.473897 5.281172 0.069738
C 1.635386 4.796231 -0.528544
C 1.723282 3.457562 -0.882540
H -2.835131 0.314984 -3.707658
H -1.449338 1.948135 -2.374058
H -2.267250 0.673737 -1.442107
F -1.511898 2.280384 0.215996
H -1.505835 4.761596 0.799320
H 0.399486 6.328562 0.355461
H 2.474947 5.462616 -0.714555
H 2.631950 3.067829 -1.340523
C -2.965564 -0.690879 -5.869518
C -0.666923 -1.538804 -6.380271
C -2.122788 -2.780310 -4.756951
H -2.558155 -3.373791 -5.572328
H -1.244862 -3.309191 -4.362468
H -2.860660 -2.703444 -3.946322
H -1.073513 -2.131251 -7.210080
H -0.357831 -0.562794 -6.778935
H 0.217863 -2.051609 -5.988248
H -3.298024 -1.228458 -6.766314
H -3.819621 -0.698875 -5.175675
H -2.752970 0.345767 -6.166546

TS_{rc}^{B}

54
Energy: -4065.48310393
Cu 0.421206 0.333216 0.686174
Cl 1.611478 1.701524 1.932710

C -2.078731 0.888125 1.143132
O 0.786387 -1.589101 0.794836
S 1.902193 -1.939771 -0.164286
O 2.342199 -0.745011 -0.904922
C 3.279796 -2.324537 0.980402
O 1.649853 -3.144960 -0.938320
F 4.363633 -2.616628 0.275498
F 3.524915 -1.281695 1.750930
F 2.955538 -3.363657 1.733293
C -2.490923 2.221469 1.195616
C -2.478698 2.904323 2.404752
C -2.063572 2.259874 3.568463
C -1.662429 0.931037 3.525158
C -1.662389 0.243834 2.314863
H -2.789451 2.724213 0.275344
H -2.784506 3.948375 2.439502
H -2.045149 2.802660 4.511444
H -1.322491 0.430317 4.428950
H -1.369987 -0.806842 2.275601
C 0.698133 1.725266 -1.818849
O -0.037233 1.776731 -0.780244
C 1.711590 2.804406 -2.093456
N 0.526755 0.674270 -2.601488
C -0.393675 -0.336159 -2.102637
C -0.846567 0.128553 -0.757238
C -2.041210 0.121252 -0.142535
C -3.206844 -0.679098 -0.536630
C -3.103742 -1.931599 -1.149281
C -4.207690 -2.684502 -1.510893
C -5.477910 -2.188675 -1.245266
C -5.623341 -0.958472 -0.606984
C -4.503065 -0.225027 -0.246487
H 1.204691 0.440189 -3.316483
H -1.259279 -0.419203 -2.777402
H 0.110423 -1.309858 -2.064546
F -1.888814 -2.453696 -1.367030
H -4.048464 -3.654543 -1.975506
H -6.353473 -2.772028 -1.522412
H -6.615317 -0.572862 -0.381501
H -4.616487 0.727019 0.270393
C 1.870407 3.029394 -3.596463
C 1.274660 4.094284 -1.400547
C 3.038712 2.316872 -1.483183
H 3.796630 3.092422 -1.657610
H 2.944239 2.152320 -0.400924
H 3.379886 1.379304 -1.939709
H 2.033345 4.866187 -1.581488
H 0.314994 4.460019 -1.790181
H 1.180799 3.946012 -0.318307
H 2.574251 3.855581 -3.756589
H 2.290721 2.155779 -4.114563
H 0.919987 3.298335 -4.076797

Reaction 43

reactant

33
Energy: -772.364205134
C -4.053955 0.005226 0.186397
O -4.631122 -0.025410 -0.891156
C -4.824497 -0.103286 1.506432
N -2.699134 0.122032 0.249793
H -2.205570 0.173297 1.128886
C -1.930268 0.218084 -0.981154
C -0.503397 0.162579 -0.708065
H -2.236022 -0.599096 -1.651536
H -2.188020 1.147820 -1.512810
C 0.682594 0.118792 -0.467024
C 2.080600 0.054994 -0.188386
C 2.700897 -1.182154 0.039292
C 4.061416 -1.240333 0.311315
C 4.824694 -0.078029 0.360952
C 4.195568 1.136706 0.132917
C 2.842680 1.229573 -0.139398
H 2.101982 -2.089294 -0.002339
H 4.537741 -2.202924 0.486669
H 5.891323 -0.095178 0.570935
F 4.920639 2.257359 0.178653
H 2.388125 2.201811 -0.312726
C -3.953312 0.003992 2.753909
C -5.866211 1.016381 1.510948

C -5.531513 -1.460300 1.491003
H -4.583309 -0.074882 3.650206
H -3.211403 -0.805985 2.812756
H -3.431516 0.970524 2.812487
H -6.172494 -1.562795 2.377908
H -6.152698 -1.558784 0.593411
H -4.806662 -2.286820 1.498830
H -6.515201 0.924969 2.393156
H -5.388170 2.005727 1.543067
H -6.486469 0.966517 0.609030

I_c

68
Energy: -4372.98953996
Cu 0.712401 0.077551 -0.533063
Cl 1.349001 -0.470164 -2.547512
C 1.934263 1.526350 -0.736578
O -0.566342 -1.478640 -0.393069
S -1.392575 -1.899515 0.794012
O -2.094918 -0.788884 1.442527
C -2.723757 -2.839100 -0.047835
O -0.733190 -2.874689 1.653980
F -3.581951 -3.295023 0.847860
F -3.382527 -2.039188 -0.894650
F -2.218255 -3.850180 -0.730379
C 1.352082 2.752972 -0.483799
C 2.201841 3.857665 -0.363899
C 3.572614 3.719061 -0.528570
C 4.116182 2.469040 -0.815151
C 3.292393 1.350795 -0.928462
C -1.050188 2.987145 2.128281
O -0.178820 3.849275 2.083533
C -2.530082 3.370795 2.070993
N -0.748420 1.663194 2.262365
C 0.618327 1.253599 2.425232
C 1.106692 0.204831 1.508501
C 1.813774 -0.771682 1.229706
C 2.704451 -1.813897 0.861292
C 2.230990 -3.104797 0.580514
C 3.154450 -4.067217 0.222298
C 4.515560 -3.800714 0.144657
C 4.975220 -2.520528 0.438822
C 4.079763 -1.522133 0.792202
H 0.283388 2.880445 -0.336870
H 1.759116 4.819537 -0.111216
H 4.222680 4.587372 -0.438395
H 5.188402 2.354917 -0.968905
H 3.705956 0.377428 -1.184641
H -1.452507 0.936192 2.155429
H 1.232935 2.156804 2.289614
H 0.799310 0.880847 3.446278
H 1.171536 -3.340240 0.655301
F 2.731455 -5.299053 -0.052212
H 5.193935 -4.601032 -0.141611
H 6.040067 -2.304823 0.388340
H 4.422906 -0.516837 1.028772
C -3.487540 2.192676 2.208935
C -2.768128 4.359252 3.215561
C -2.759088 4.074681 0.732049
O -3.293379 1.136781 -1.025230
C -4.510651 0.723702 -1.664055
C -5.566010 0.688844 -0.591082
C -2.095010 0.878992 -1.534572
O -1.126804 1.215688 -0.866683
C -2.006080 0.217520 -2.871211
H -4.751707 1.444423 -2.458681
H -4.379029 -0.265952 -2.117088
H -6.532357 0.392759 -1.014881
H -5.283927 -0.034345 0.184313
H -5.675162 1.674575 -0.122997
H -2.738647 0.624063 -3.577066
H -0.994049 0.330356 -3.264860
H -2.198491 -0.856729 -2.752048
H -3.349291 1.658335 3.159739
H -4.521710 2.566189 2.195193
H -3.382814 1.475581 1.384283
H -3.799259 4.426285 0.666300
H -2.090451 4.938256 0.630808
H -2.577569 3.388180 -0.105486
H -3.800112 4.735438 3.174997
H -2.623169 3.877495 4.193069

H -2.078946 5.208445 3.146330

I$_1$

54
Energy: -4065.46776292
Cu -0.009263 0.126098 -0.001153
Cl 0.009855 0.317207 2.121755
C 1.855547 0.199909 0.311753
O -2.031674 0.313744 -0.282734
S -2.049142 1.692779 -0.902441
O -2.713326 1.787956 -2.188214
C -3.061848 2.656821 0.284215
O -0.680820 2.248418 -0.792715
F -3.107122 3.917980 -0.111945
F -4.287362 2.159317 0.318356
F -2.525590 2.591647 1.489029
C 2.617641 -0.945366 0.426200
C 4.001479 -0.789868 0.325206
C 4.563702 0.472108 0.148266
C 3.752494 1.599705 0.075183
C 2.365833 1.477823 0.181858
H 2.166620 -1.929210 0.562105
H 4.636859 -1.671728 0.392837
H 5.645216 0.578300 0.083926
H 4.189846 2.589500 -0.045952
H 1.717674 2.352693 0.159998
C -0.441819 -3.202393 0.696060
O 0.622462 -3.538717 0.188781
C -0.809267 -3.575955 2.129815
N -1.377000 -2.537480 -0.048025
C -1.094604 -2.232033 -1.430215
C -0.127385 -1.136100 -1.645180
C 0.596729 -0.330437 -2.227378
C 1.533778 0.523152 -2.871303
C 1.157081 1.810919 -3.278523
C 2.114721 2.605434 -3.880204
C 3.415204 2.166343 -4.092622
C 3.772422 0.882250 -3.693011
C 2.839920 0.055615 -3.083290
H -2.162305 -2.070067 0.387294
H -0.686594 -3.129391 -1.912852
H -2.034039 -1.958983 -1.924213
H 0.151283 2.185433 -3.102582
F 1.784242 3.835079 -4.270537
H 4.126078 2.836982 -4.569891
H 4.788541 0.529560 -3.855646
H 3.107191 -0.944896 -2.750146
C -2.029074 -2.832025 2.666565
C -1.098046 -5.081944 2.103117
C 0.404416 -3.302943 3.017536
H 0.202098 -3.650000 4.040088
H 1.287203 -3.830658 2.638177
H 0.632360 -2.228863 3.059791
H -1.329978 -5.433681 3.117921
H -1.958902 -5.311997 1.458999
H -0.229020 -5.638339 1.730973
H -2.193996 -3.113555 3.715060
H -1.892202 -1.741373 2.638389
H -2.947463 -3.094548 2.121004

TS$_{rc}^{A}$

54
Energy: -4065.44588888
Cu 0.006469 -0.004032 0.003842
Cl 0.001077 0.018301 2.139310
C -0.796223 1.690908 0.112888
O 0.409525 -1.927965 -0.160939
S 1.208672 -2.809741 -1.082167
O 2.639459 -2.821250 -0.771329
C 0.572712 -4.454223 -0.580656
O 0.856532 -2.680968 -2.497291
F 1.176203 -5.387787 -1.300921
F 0.809376 -4.669118 0.700610
F -0.733131 -4.508874 -0.804027
C -0.040024 2.841921 -0.015794
C -0.708259 4.065672 -0.055027
C -2.095298 4.116266 0.050634
C -2.825786 2.942249 0.202820
C -2.174691 1.709779 0.242890
H 1.043727 2.793408 -0.099494

H -0.133130 4.983713 -0.168739
H -2.607874 5.076325 0.025193
H -3.909694 2.976446 0.302052
H -2.742906 0.789048 0.375362
C 3.241489 0.090075 -0.960140
O 2.218747 0.743847 -0.667719
C 4.336225 -0.176810 0.047793
N 3.371483 -0.263657 -2.252734
C 2.276816 0.138762 -3.120908
C 1.045983 0.323127 -2.338453
C -0.141425 0.179228 -1.949348
C -1.466980 -0.019103 -2.527850
C -2.039785 -1.291261 -2.584393
C -3.312270 -1.409583 -3.118425
C -4.032484 -0.319800 -3.583814
C -3.449236 0.940442 -3.509000
C -2.174223 1.100305 -2.980195
H 3.957986 -1.049590 -2.506183
H 2.543819 1.059781 -3.659326
H 2.073485 -0.661747 -3.845255
H -1.500423 -2.173595 -2.248916
F -3.865225 -2.621224 -3.188314
H -5.028579 -0.473192 -3.991468
H -3.997760 1.810359 -3.864798
H -1.722717 2.087955 -2.897593
C 3.704853 -0.636452 1.362277
C 5.351157 -1.202056 -0.448359
C 5.030250 1.180043 0.250062
H 5.836549 1.061824 0.985839
H 5.474910 1.550541 -0.684668
H 4.327460 1.932447 0.628156
H 6.115606 -1.346642 0.325657
H 4.877395 -2.173466 -0.636623
H 5.876200 -0.860774 -1.353169
H 4.497479 -0.762823 2.111933
H 2.982955 0.096790 1.742209
H 3.184894 -1.593828 1.235735

TS_{ar}^{B}

54
Energy: -4065.45739144
Cu -0.034708 -0.743203 -0.518114
Cl 0.338285 -1.104707 -2.620801
C -1.829979 -1.475625 -0.663724
O 1.682605 0.204573 -0.320619
S 1.882944 1.633480 0.126597
O 2.574697 1.705968 1.417466
C 3.116181 2.169834 -1.119531
O 0.704525 2.471684 -0.063353
F 3.497677 3.409347 -0.856672
F 4.171883 1.366630 -1.063116
F 2.586516 2.115165 -2.325795
C -2.034027 -2.693044 -0.028906
C -3.094624 -3.493645 -0.450977
C -3.936226 -3.061276 -1.469624
C -3.720356 -1.827165 -2.081841
C -2.660719 -1.023530 -1.683601
H -1.370801 -3.018589 0.769023
H -3.260759 -4.455920 0.030033
H -4.768306 -3.686270 -1.788601
H -4.371080 -1.490825 -2.886813
H -2.482287 -0.061400 -2.159980
C 1.955977 -1.918823 1.244997
O 0.969280 -2.446901 0.711891
C 3.366162 -2.313628 0.862653
N 1.792077 -0.986723 2.217176
C 0.463148 -0.493304 2.523273
C -0.435313 -0.308496 1.350317
C -1.535410 -0.014852 0.815463
C -2.704195 0.835796 0.732307
C -2.505139 2.201118 0.505337
C -3.621829 3.021285 0.481832
C -4.904277 2.531298 0.677704
C -5.080334 1.167378 0.898331
C -3.988492 0.311727 0.917018
H 2.530365 -0.292560 2.312384
C -0.238388 -1.389799 3.541532
H 0.608363 0.512540 2.948018
H -1.504974 2.604679 0.347427
F -3.458905 4.325147 0.266968
H -5.744293 3.221333 0.651517

H -6.082331 0.772567 1.051820
H -4.115181 -0.759017 1.069343
C 4.458834 -1.442134 1.467851
H 3.467048 -3.338313 1.262799
C 3.495763 -2.387264 -0.657865
H 5.441045 -1.819188 1.158754
H 4.384542 -0.404361 1.110055
H 4.441711 -1.439526 2.565733
H 4.457348 -2.842821 -0.924703
H 2.689765 -2.979219 -1.103692
H 3.453803 -1.381351 -1.094249
H -1.229219 -0.995137 3.794905
H -0.352162 -2.401203 3.135896
H 0.367528 -1.445469 4.452664

I_2

54
Energy: -4065.51056457
Cu 0.266149 -1.432783 -1.966224
Cl 0.921632 -3.484137 -2.440292
C 2.063484 0.394486 -0.693062
O -0.123045 -1.743101 -0.153975
S -1.640236 -1.696940 0.053938
O -2.338372 -1.907776 -1.218164
C -1.886061 -3.224829 1.041953
O -2.067158 -0.582378 0.886430
F -3.177658 -3.338487 1.313020
F -1.484178 -4.273886 0.356295
F -1.205773 -3.136199 2.170677
C 2.687082 -0.448866 -1.622670
C 3.790100 -1.214133 -1.252354
C 4.290384 -1.128215 0.039589
C 3.695688 -0.264285 0.959616
C 2.591373 0.493487 0.598320
H 2.360802 -0.448819 -2.664230
H 4.247601 -1.880671 -1.980443
H 5.148000 -1.730111 0.333778
H 4.089659 -0.193639 1.971959
H 2.106366 1.144114 1.325111
C -1.114531 -0.674809 -4.141573
O 0.039744 -1.033690 -3.781255
C -1.705607 -1.352265 -5.362598
N -1.838573 0.207418 -3.462376
C -1.463726 0.830961 -2.195090
C -0.136195 0.404445 -1.695547
C 0.809421 1.092419 -1.050423
C 0.630908 2.503503 -0.636041
C -0.535595 2.899763 0.024666
C -0.672259 4.226330 0.402656
C 0.308991 5.171895 0.150451
C 1.473852 4.764536 -0.493912
C 1.641736 3.440527 -0.879669
H -2.788901 0.365633 -3.773175
H -1.438163 1.922989 -2.343521
H -2.262454 0.605391 -1.472829
H -1.306754 2.178624 0.294129
F -1.783398 4.602653 1.038504
H 0.154333 6.199817 0.469219
H 2.258687 5.491478 -0.693918
H 2.556009 3.120910 -1.377782
C -2.887551 -0.595134 -5.960155
C -0.610824 -1.530889 -6.413955
C -2.161383 -2.728969 -4.847041
H -2.587160 -3.301078 -5.682427
H -1.318537 -3.292483 -4.424740
H -2.927688 -2.631167 -4.065303
H -1.017584 -2.094663 -7.263335
H -0.248671 -0.564207 -6.789783
H 0.239399 -2.086169 -6.003704
H -3.214771 -1.108903 -6.872659
H -3.761877 -0.575536 -5.292257
H -2.624415 0.435071 -6.238292

TS_{rc}^{B}

54
Energy: -4065.48275221
Cu -0.781326 1.308545 0.247811
Cl -2.269255 2.675069 -0.625989
C 0.342262 -1.598607 1.712252
O 0.951156 1.664520 0.947112

S 2.054739 1.646054 -0.102095
O 1.527393 1.353592 -1.436879
C 2.506105 3.422076 -0.147815
O 3.237007 0.927695 0.347289
F 3.462067 3.608783 -1.044506
F 1.444201 4.137241 -0.484113
F 2.937933 3.810062 1.040698
C -0.184841 -0.664776 2.617242
C -0.097898 -0.870317 3.983182
C 0.520657 -2.014602 4.481177
C 1.052509 -2.948708 3.598410
C 0.966561 -2.743934 2.228257
H -0.645657 0.244022 2.242242
H -0.503852 -0.124676 4.663694
H 0.594804 -2.171990 5.555667
H 1.542531 -3.843464 3.977716
H 1.383620 -3.482881 1.546778
C -3.020123 -0.341295 -0.567438
O -2.295402 -0.237726 0.458415
C -4.525845 -0.383612 -0.439845
N -2.420866 -0.523325 -1.743865
C -0.970318 -0.378482 -1.781601
C -0.486759 -0.489235 -0.374327
C 0.227026 -1.428388 0.261556
C 1.013250 -2.333947 -0.630940
C 2.379954 -2.121725 -0.810145
C 3.077465 -3.004764 -1.619973
C 2.465875 -4.078856 -2.250644
C 1.102049 -4.275523 -2.063194
C 0.372209 -3.406831 -1.257928
H -2.950754 -0.411742 -2.599074
H -0.522932 -1.189311 -2.371893
H -0.694304 0.579628 -2.244407
H 2.891666 -1.279079 -0.342832
F 4.382766 -2.817152 -1.803012
H 3.063244 -4.738744 -2.875098
H 0.607345 -5.116886 -2.544779
H -0.695718 -3.560589 -1.098044
C -4.949857 0.523035 0.714774
C -5.204576 0.067070 -1.731539
C -4.887407 -1.842591 -0.126890
H -6.289654 0.100213 -1.573051
H -4.877871 1.074313 -2.024274
H -5.034150 -0.630540 -2.565284
H -5.974302 -1.923801 0.005424
H -4.591025 -2.517415 -0.941934
H -4.403369 -2.179238 0.798772
H -6.042496 0.488897 0.814393
H -4.505520 0.194648 1.661098
H -4.639300 1.560086 0.534247

Reaction 44

reactant

33
Energy: -772.364715130
C -4.053508 0.000947 0.186410
O -4.632391 -0.015432 -0.890710
C -4.825264 -0.096064 1.506758
N -2.697055 0.091364 0.248839
H -2.200779 0.124136 1.127277
C -1.926138 0.171389 -0.982526
C -0.499404 0.126758 -0.707647
H -2.227031 -0.655991 -1.642807
H -2.189107 1.092252 -1.526695
C 0.687006 0.089948 -0.465698
C 2.084917 0.039815 -0.186234
C 2.723894 -1.191573 0.022218
C 4.082304 -1.247734 0.295827
C 4.799310 -0.062015 0.358609
C 4.200818 1.172925 0.156983
C 2.841588 1.218903 -0.114919
H 2.138670 -2.107261 -0.034568
H 4.596011 -2.191766 0.460184
F 6.106612 -0.110828 0.621350
H 4.804986 2.074957 0.215485
H 2.347999 2.175008 -0.277476
C -3.951765 -0.006300 2.753979
C -5.847022 1.041796 1.513828
C -5.556193 -1.440313 1.489417
H -4.582707 -0.074125 3.650543

H -3.225221 -0.830180 2.811858
H -3.411643 0.950070 2.812835
H -6.198290 -1.533288 2.376587
H -6.179608 -1.526078 0.592053
H -4.846016 -2.279522 1.495066
H -6.497695 0.959993 2.395766
H -5.351442 2.022405 1.548182
H -6.467840 1.005008 0.611670

I_c

68
Energy: -4372.99252678
Cu 0.707603 0.088787 -0.524154
Cl 1.348355 -0.492432 -2.532385
C 1.927673 1.541250 -0.700189
O -0.574705 -1.467371 -0.385623
S -1.410226 -1.895807 0.790929
O -2.121967 -0.791839 1.440270
C -2.731750 -2.834631 -0.066788
O -0.757319 -2.875363 1.651943
F -3.597092 -3.296396 0.819183
F -3.384341 -2.034203 -0.917091
F -2.217545 -3.843117 -0.748024
C 1.353765 2.763735 -0.410217
C 2.202221 3.871259 -0.313993
C 3.566364 3.739714 -0.532469
C 4.102787 2.494189 -0.849465
C 3.279340 1.373192 -0.940146
C -1.047132 2.978070 2.171444
O -0.165520 3.830969 2.175671
C -2.521048 3.379904 2.085058
N -0.763360 1.646866 2.261610
C 0.592173 1.211098 2.447233
C 1.095656 0.197036 1.496070
C 1.826281 -0.774145 1.244131
C 2.713502 -1.809888 0.877016
C 2.244116 -3.113176 0.622869
C 3.136103 -4.102532 0.252121
C 4.484857 -3.785882 0.146430
C 4.980456 -2.512324 0.403215
C 4.088299 -1.519880 0.764591
H 0.291485 2.885560 -0.218091
H 1.765732 4.829375 -0.036996
H 4.216532 4.609581 -0.459232
H 5.168711 2.385753 -1.045593
H 3.686912 0.404863 -1.223447
H -1.477290 0.932965 2.135092
H 1.221143 2.110920 2.368748
H 0.735818 0.793279 3.456401
H 1.181230 -3.320984 0.730536
H 2.809233 -5.117658 0.042837
F 5.339142 -4.738720 -0.206849
H 6.047260 -2.325868 0.312797
H 4.437554 -0.510352 0.974729
C -3.494595 2.212601 2.202891
C -2.773537 4.373462 3.221499
C -2.712121 4.083238 0.739499
O -3.291090 1.145389 -1.038760
C -4.505849 0.734998 -1.683411
C -5.565854 0.696479 -0.615108
C -2.090962 0.894157 -1.548766
O -1.124514 1.234652 -0.881047
C -1.998245 0.233373 -2.885543
H -4.743912 1.458495 -2.476483
H -4.373049 -0.252968 -2.139867
H -6.530486 0.402380 -1.044211
H -5.287650 -0.029586 0.158938
H -5.676825 1.680410 -0.143790
H -2.724358 0.645156 -3.595169
H -0.982902 0.340336 -3.272488
H -2.198382 -0.839754 -2.768605
H -3.377761 1.673452 3.153889
H -4.523930 2.598318 2.173068
H -3.385519 1.496658 1.377610
H -3.746237 4.447178 0.649899
H -2.031136 4.938804 0.651309
H -2.520243 3.392548 -0.092239
H -3.798697 4.764210 3.154467
H -2.659845 3.891989 4.203223
H -2.070666 5.212413 3.168305

I_1

54
Energy: -4065.47066106
Cu 0.046981 0.068913 0.029590
Cl 0.038623 0.303548 2.148786
C 1.915024 0.095514 0.319541
O -1.962460 0.294397 -0.281123
S -1.955949 1.667777 -0.914379
O -2.604381 1.754249 -2.210624
C -2.979961 2.653203 0.244901
O -0.586993 2.214445 -0.796048
F -3.009096 3.909715 -0.169023
F -4.209880 2.166065 0.265640
F -2.463646 2.600754 1.458489
C 2.653759 -1.064924 0.440649
C 4.043112 -0.936049 0.381122
C 4.635680 0.316256 0.235770
C 3.848254 1.459365 0.149115
C 2.456513 1.362655 0.208423
H 2.182449 -2.042285 0.553824
H 4.658571 -1.831293 0.457004
H 5.720547 0.401820 0.206036
H 4.307730 2.441793 0.052134
H 1.827851 2.251383 0.172653
C -0.436800 -3.241372 0.748382
O 0.635083 -3.626449 0.294652
C -0.878217 -3.574215 2.171313
N -1.313159 -2.554268 -0.045701
C -0.964917 -2.282639 -1.419551
C -0.039874 -1.144364 -1.621113
C 0.651651 -0.344476 -2.255807
C 1.504036 0.568822 -2.916900
C 0.988085 1.763410 -3.451675
C 1.835220 2.655307 -4.085167
C 3.187110 2.345598 -4.171940
C 3.725528 1.175092 -3.650993
C 2.876330 0.283023 -3.021499
H -2.111325 -2.069617 0.345192
H -0.490238 -3.178589 -1.838742
H -1.884690 -2.072943 -1.978107
H -0.072829 1.980753 -3.346090
H 1.472975 3.588923 -4.507236
F 4.000677 3.205692 -4.775360
H 4.792625 0.989499 -3.741959
H 3.262949 -0.634704 -2.581386
C -2.102094 -2.790089 2.637140
C -1.201997 -5.073202 2.163922
C 0.301381 -3.309330 3.106604
H 0.047328 -3.633812 4.125068
H 1.188585 -3.861126 2.775030
H 0.549308 -2.239353 3.140429
H -1.486941 -5.396585 3.174646
H -2.039123 -5.297570 1.487243
H -0.331263 -5.657790 1.842643
H -2.321386 -3.047157 3.681987
H -1.936931 -1.703684 2.595839
H -3.001203 -3.041062 2.055090

TS_{rc}^{A}

54
Energy: -4065.44736983
Cu -0.329553 -0.521511 -0.850515
Cl 0.144138 -1.235419 -2.809289
C -1.816698 -1.668256 -0.939108
O 0.923857 0.983145 -0.913710
S 1.631501 1.813172 0.125856
O 2.546420 1.027416 0.964276
C 2.734748 2.799773 -0.956822
O 0.774444 2.775243 0.806322
F 3.484159 3.588508 -0.199815
F 3.523493 1.997862 -1.657656
F 2.018114 3.541905 -1.783494
C -1.775355 -2.932069 -0.378799
C -2.955087 -3.675617 -0.343474
C -4.132047 -3.156262 -0.874380
C -4.136338 -1.891657 -1.453783
C -2.965851 -1.135149 -1.498707
H -0.850298 -3.330464 0.033921
H -2.946464 -4.669242 0.102948
H -5.047425 -3.744818 -0.846569

H -5.050522 -1.486586 -1.885088
H -2.965733 -0.148449 -1.962289
C 2.080260 -1.668160 1.294324
O 1.016972 -1.842773 0.666372
C 3.425199 -2.054111 0.725116
N 1.955323 -1.233924 2.566660
C 0.590994 -0.899222 2.940432
C -0.213836 -0.583441 1.749511
C -0.999463 -0.025739 0.936483
C -2.196900 0.806175 0.975418
C -2.263488 2.024081 0.291743
C -3.444859 2.752543 0.283761
C -4.554518 2.243439 0.941173
C -4.519687 1.030031 1.611119
C -3.332393 0.311788 1.626848
H 2.716100 -0.697828 2.966648
H 0.140977 -1.736761 3.493509
H 0.595033 -0.014671 3.591287
H -1.376525 2.407983 -0.206912
H -3.517802 3.708636 -0.228449
F -5.693794 2.937890 0.923280
H -5.416667 0.663366 2.103934
H -3.287204 -0.661170 2.115256
C 3.496918 -1.623901 -0.739743
C 4.582018 -1.448782 1.514396
C 3.476797 -3.587513 0.819672
H 4.433493 -3.939598 0.411956
H 3.403075 -3.930870 1.861284
H 2.666779 -4.046114 0.239213
H 5.528273 -1.754357 1.050122
H 4.541846 -0.351995 1.499574
H 4.608448 -1.805691 2.554662
H 4.441911 -1.982852 -1.169006
H 2.670047 -2.041090 -1.327639
H 3.464318 -0.532221 -0.833817

TS_{ar}^{B}

54
Energy: -4065.45959065
Cu 0.093289 -0.730329 -0.513165
Cl 0.529006 -1.145570 -2.595532
C -1.676501 -1.519997 -0.678807
O 1.772041 0.288689 -0.312068
S 1.905216 1.729774 0.119997
O 2.628239 1.849827 1.389752
C 3.059984 2.337669 -1.167705
O 0.675502 2.499520 -0.037962
F 3.338097 3.611639 -0.939769
F 4.181319 1.630976 -1.125077
F 2.504950 2.212281 -2.357801
C -1.864584 -2.733147 -0.030695
C -2.889823 -3.570721 -0.469032
C -3.713599 -3.179254 -1.518388
C -3.515545 -1.948920 -2.144590
C -2.491171 -1.109265 -1.729273
H -1.215789 -3.025915 0.791632
H -3.042807 -4.529494 0.023237
H -4.517867 -3.833176 -1.850477
H -4.151905 -1.644620 -2.973466
H -2.327002 -0.149890 -2.216225
C 2.072367 -1.805777 1.318943
O 1.111481 -2.378865 0.785385
C 3.499464 -2.172230 0.973170
N 1.862957 -0.845781 2.254558
C 0.513867 -0.384017 2.520385
C -0.367157 -0.268584 1.324217
C -1.480811 -0.027632 0.788361
C -2.676653 0.772137 0.677601
C -2.537141 2.155221 0.498640
C -3.667787 2.958639 0.445399
C -4.914857 2.366203 0.575300
C -5.076405 0.996059 0.748525
C -3.948523 0.195106 0.788571
H 2.580151 -0.128989 2.343455
C -0.178228 -1.268137 3.555559
H 0.619532 0.639320 2.914076
H -1.538052 2.578564 0.394834
H -3.597588 4.034097 0.304120
F -6.000685 3.133942 0.528957
H -6.078064 0.584679 0.842736
H -4.041062 -0.883672 0.907234

C 4.556627 -1.255749 1.574415
H 3.621072 -3.182897 1.402057
C 3.655432 -2.283490 -0.542796
H 5.554457 -1.612486 1.292536
H 4.459726 -0.229380 1.190612
H 4.518667 -1.226458 2.671426
H 4.642375 -2.697678 -0.783168
H 2.886654 -2.928792 -0.980216
H 3.567579 -1.295279 -1.012188
H -1.183218 -0.893166 3.781704
H -0.258895 -2.293793 3.178563
H 0.414421 -1.280241 4.477016

I_2

54
Energy: -4065.51123041
Cu 0.264695 -1.435882 -1.973436
Cl 0.895916 -3.500888 -2.422330
C 2.079447 0.393395 -0.711666
O -0.129196 -1.722710 -0.157540
S -1.643944 -1.649145 0.054118
O -2.351870 -1.867850 -1.211010
C -1.910477 -3.157250 1.066523
O -2.050959 -0.515578 0.872098
F -3.203093 -3.247117 1.342101
F -1.526095 -4.222544 0.396215
F -1.226431 -3.061571 2.192437
C 2.694845 -0.461451 -1.636714
C 3.799888 -1.223797 -1.267379
C 4.312022 -1.123362 0.019061
C 3.726388 -0.248095 0.933966
C 2.619503 0.506599 0.573618
H 2.360903 -0.471937 -2.675779
H 4.250740 -1.898642 -1.991980
H 5.172003 -1.722302 0.312400
H 4.129571 -0.165781 1.941808
H 2.141793 1.166064 1.297213
C -1.103198 -0.681569 -4.159554
O 0.044284 -1.054383 -3.792359
C -1.700093 -1.362863 -5.375652
N -1.817343 0.216935 -3.491150
C -1.441586 0.843232 -2.224999
C -0.118850 0.407391 -1.720448
C 0.825510 1.090722 -1.067720
C 0.631658 2.492820 -0.633440
C -0.522629 2.863948 0.065904
C -0.705143 4.176675 0.484655
C 0.279539 5.109219 0.199180
C 1.444253 4.769380 -0.475605
C 1.618340 3.454569 -0.882090
H -2.763620 0.385883 -3.808518
H -1.403476 1.934117 -2.378662
H -2.246595 0.629000 -1.506481
H -1.258940 2.101639 0.323961
H -1.587083 4.483016 1.041896
F 0.110233 6.371671 0.595530
H 2.192960 5.534414 -0.666588
H 2.528513 3.162260 -1.404995
C -2.870771 -0.596695 -5.983719
C -0.605517 -1.566512 -6.422598
C -2.174826 -2.728007 -4.846452
H -2.608163 -3.302996 -5.675937
H -1.339844 -3.298329 -4.417723
H -2.939794 -2.611626 -4.065872
H -1.017441 -2.134287 -7.266815
H -0.230582 -0.608506 -6.807989
H 0.236885 -2.127836 -6.004533
H -3.202412 -1.115327 -6.891867
H -3.746374 -0.559157 -5.318252
H -2.593687 0.427175 -6.271560

TS_{rc}^{B}

54
Energy: -4065.48374581
Cu -0.990215 1.209543 0.163662
Cl -2.662679 2.216768 -0.854027
C 0.587581 -1.303789 1.890431
O 0.631843 1.934721 0.850684
S 1.750717 2.039956 -0.175664
O 1.341582 1.517452 -1.481402

C 1.842790 3.857023 -0.398956
O 3.038245 1.625336 0.360113
F 2.764510 4.139313 -1.306986
F 0.669944 4.312737 -0.809445
F 2.162896 4.437604 0.745589
C -0.138583 -0.416462 2.699432
C -0.060407 -0.485299 4.079492
C 0.748656 -1.442007 4.688169
C 1.479586 -2.326018 3.901576
C 1.402497 -2.257918 2.517232
H -0.752514 0.350896 2.237068
H -0.624548 0.222080 4.683862
H 0.814776 -1.490998 5.773532
H 2.118946 -3.073460 4.367627
H 1.976881 -2.955735 1.910959
C -2.871912 -0.872640 -0.555388
O -2.213949 -0.558733 0.472387
C -4.350297 -1.173540 -0.456595
N -2.208830 -1.039526 -1.699461
C -0.806394 -0.640339 -1.722138
C -0.356834 -0.547829 -0.302079
C 0.493555 -1.282268 0.428355
C 1.470580 -2.081946 -0.370415
C 2.774880 -1.617130 -0.562104
C 3.683523 -2.378532 -1.286628
C 3.273826 -3.595067 -1.811204
C 1.984754 -4.078097 -1.637823
C 1.081583 -3.309718 -0.913419
H -2.720032 -1.094433 -2.571386
H -0.203320 -1.401185 -2.234689
H -0.689595 0.311524 -2.259585
H 3.071094 -0.650415 -0.150956
H 4.701744 -2.037952 -1.457238
F 4.144527 -4.326936 -2.507534
H 1.711873 -5.041053 -2.062626
H 0.063033 -3.667764 -0.755443
C -4.969397 -0.271474 0.609939
C -5.051722 -0.952120 -1.794999
C -4.459494 -2.645109 -0.032484
H -6.130822 -1.098948 -1.662210
H -4.895711 0.071162 -2.163220
H -4.733459 -1.671328 -2.564588
H -5.519034 -2.909411 0.081335
H -4.018382 -3.316773 -0.782015
H -3.958317 -2.816976 0.928649
H -6.040249 -0.498112 0.692967
H -4.503847 -0.435658 1.588237
H -4.847697 0.787116 0.347583

Reaction 45

reactant

35
Energy: -728.480472012
C -4.019029 -0.099497 0.160806
O -4.603682 -0.063405 -0.913040
C -4.784554 -0.015767 1.485879
N -2.663470 -0.202691 0.216331
H -2.164869 -0.263910 1.092150
C -1.895446 -0.286492 -1.017064
C -0.469167 -0.190675 -0.752532
H -2.127746 -1.227490 -1.541068
H -2.233540 0.514081 -1.691751
C 0.718437 -0.143408 -0.506938
C 2.111102 -0.040520 -0.228408
C 2.920225 -1.183410 -0.174404
C 4.278193 -1.090529 0.083778
C 4.841495 0.168357 0.294455
C 4.060885 1.312668 0.249360
C 2.686791 1.233498 -0.009402
H 2.452437 -2.151341 -0.348014
H 4.894014 -1.986260 0.120176
H 5.907681 0.260474 0.496737
H 4.508797 2.291713 0.422390
N 1.898274 2.365315 -0.006875
C -3.904301 -0.105903 2.728269
C -5.527600 1.321712 1.481005
C -5.796264 -1.162846 1.490444
H -4.530726 -0.040836 3.628227
H -3.362477 -1.061558 2.783441
H -3.178388 0.718576 2.783077

H -6.441435 -1.094480 2.377602
H -6.423850 -1.124087 0.592975
H -5.291590 -2.139182 1.513095
H -6.168347 1.401739 2.370414
H -4.824961 2.167181 1.492023
H -6.153693 1.408992 0.585710
H 2.368599 3.239658 -0.197266
H 0.997648 2.274676 -0.461689

I_c

70
Energy: -4329.11909349
Cu 0.513610 0.025388 -0.557947
Cl 1.127951 -0.658567 -2.552779
C 1.784458 1.435372 -0.616563
O -0.940696 -1.396641 -0.427165
S -1.826338 -1.775082 0.719397
O -2.655732 -0.686895 1.238992
C -3.015203 -2.882992 -0.129609
O -1.158175 -2.623251 1.712466
F -3.939292 -3.285065 0.725907
F -3.614111 -2.225219 -1.125072
F -2.388642 -3.934232 -0.629781
C 1.273230 2.656848 -0.221521
C 2.140872 3.750609 -0.183025
C 3.473738 3.605376 -0.544295
C 3.952387 2.361467 -0.946828
C 3.105250 1.253972 -0.983718
C -0.973220 2.846782 2.345201
O 0.017157 3.483521 2.683233
C -2.309310 3.546046 2.076250
N -0.932269 1.495419 2.148263
C 0.246268 0.747135 2.483502
C 0.910584 -0.015450 1.383470
C 1.790891 -0.905138 1.242317
C 2.824450 -1.775408 0.948600
C 2.575501 -3.161488 0.666021
C 3.681284 -3.965517 0.301623
C 4.949259 -3.440500 0.256610
C 5.208515 -2.087510 0.565577
C 4.158407 -1.275089 0.898609
H 0.236503 2.775069 0.078496
H 1.754319 4.708666 0.161757
H 4.144563 4.462110 -0.510780
H 4.991535 2.243090 -1.251782
H 3.471361 0.286816 -1.322078
H -1.760942 0.973486 1.874757
H 0.969335 1.480962 2.868464
H 0.025716 0.027100 3.286549
N 1.348388 -3.688173 0.746037
H 3.508231 -5.015061 0.066757
H 5.777100 -4.091612 -0.020811
H 6.223737 -1.701198 0.531499
H 4.307968 -0.221344 1.128861
C -3.503214 2.597113 2.028953
C -2.517583 4.582841 3.178993
C -2.165639 4.255781 0.725641
O -3.427105 1.195235 -1.103502
C -4.661566 0.694728 -1.635915
C -5.577354 0.437384 -0.467053
C -2.244170 0.939632 -1.654850
O -1.253841 1.313025 -1.045082
C -2.191815 0.229448 -2.970232
H -5.068283 1.448348 -2.325360
H -4.485351 -0.228301 -2.198741
H -6.539079 0.045133 -0.818248
H -5.118923 -0.294686 0.209611
H -5.762640 1.358977 0.096825
H -2.925613 0.629876 -3.679176
H -1.180740 0.309836 -3.375444
H -2.401628 -0.837474 -2.816410
H -3.606141 2.020156 2.959252
H -4.424655 3.183529 1.900586
H -3.445233 1.896646 1.185239
H -3.073532 4.837337 0.510023
H -1.310801 4.945746 0.739859
H -2.023418 3.529781 -0.087415
H -3.419358 5.174888 2.968815
H -2.649323 4.101674 4.158317
H -1.657109 5.257112 3.246719
H 1.204489 -4.655046 0.492942

H 0.527050 -3.166823 1.063064

I$_1$

56
Energy: -4021.59752581
Cu -0.004546 0.057108 0.350434
Cl -0.045504 0.400878 2.461288
C 1.873273 0.024291 0.485105
O -1.976773 0.335234 0.046800
S -2.048634 1.626391 -0.731837
O -2.588049 1.462261 -2.083761
C -3.308700 2.570961 0.205416
O -0.793940 2.376349 -0.611531
F -3.491092 3.743632 -0.381949
F -4.448975 1.900958 0.214522
F -2.891548 2.756364 1.442526
C 2.602797 -1.146811 0.570563
C 3.995513 -1.039705 0.552834
C 4.611442 0.205420 0.457996
C 3.840986 1.360999 0.383112
C 2.447396 1.281046 0.404308
H 2.121256 -2.123958 0.623044
H 4.595108 -1.947115 0.612010
H 5.698018 0.274849 0.451454
H 4.317037 2.338654 0.322741
H 1.837770 2.184072 0.372004
C -0.499248 -3.242167 0.680911
O 0.558843 -3.691856 0.256616
C -1.030432 -3.611859 2.064523
N -1.265541 -2.413795 -0.092488
C -0.815330 -2.021324 -1.405899
C 0.048447 -0.795397 -1.424990
C 0.698932 -0.095738 -2.241512
C 1.530969 0.713817 -2.981826
C 1.021404 1.811579 -3.757081
C 1.958108 2.595614 -4.469905
C 3.302263 2.316597 -4.409487
C 3.811291 1.243880 -3.645824
C 2.933452 0.456512 -2.952452
H -2.079648 -1.944731 0.284664
H -0.250280 -2.859552 -1.829836
H -1.692791 -1.822510 -2.034199
N -0.290888 2.073141 -3.849867
H 1.593615 3.431169 -5.066329
H 3.992944 2.947566 -4.967477
H 4.880642 1.054038 -3.607404
H 3.277611 -0.372367 -2.334572
C -2.252410 -2.806512 2.497563
C -1.388197 -5.100242 2.000510
C 0.108194 -3.396566 3.063344
H -0.580571 2.928836 -4.303185
H -0.984841 1.658119 -3.225244
H -0.203065 -3.738948 4.059961
H 0.998924 -3.959686 2.761443
H 0.374723 -2.332407 3.137977
H -1.718766 -5.446601 2.989536
H -2.204214 -5.284564 1.286818
H -0.519831 -5.695374 1.693211
H -2.544486 -3.110551 3.511613
H -2.045949 -1.726362 2.531331
H -3.122704 -2.989874 1.849822

TS$_{rc}^{A}$

56
Energy: -4021.57604420
Cu 0.645558 0.274166 -0.701884
Cl 0.156232 0.877789 -2.717702
C 2.174481 1.372956 -0.854426
O -1.243269 -0.400797 -0.294801
S -1.865766 -1.675812 0.185577
O -2.747829 -1.484022 1.340009
C -2.978754 -2.057800 -1.218320
O -0.929764 -2.802128 0.250207
F -3.654805 -3.168284 -0.973820
F -3.832580 -1.057342 -1.397924
F -2.256244 -2.215937 -2.316016
C 2.068318 2.712701 -0.519151
C 3.213233 3.504591 -0.596832
C 4.423768 2.960861 -1.014339
C 4.495093 1.617220 -1.361973

C 3.359557 0.809605 -1.290817
H 1.120105 3.135001 -0.196420
H 3.147576 4.558679 -0.330103
H 5.311295 3.588234 -1.076271
H 5.435405 1.184543 -1.700784
H 3.420850 -0.237771 -1.579337
C -1.661481 1.768705 1.401054
O -0.494291 2.053676 1.075961
C -2.866582 2.380393 0.720948
N -1.814153 0.982173 2.494678
C -0.531179 0.585126 3.060024
C 0.460912 0.461716 1.983233
C 1.212053 -0.127460 1.148919
C 2.211914 -1.177849 1.238207
C 2.387353 -2.030205 0.134481
C 3.420704 -2.962344 0.137822
C 4.266298 -3.066031 1.235733
C 4.077635 -2.245911 2.346268
C 3.054573 -1.309285 2.347517
H -2.554101 0.280000 2.466108
H -0.220856 1.324989 3.811369
H -0.637924 -0.392711 3.548819
N 1.489925 -1.864655 -0.950201
H 3.546945 -3.619719 -0.722417
H 5.071886 -3.797913 1.228498
H 4.738334 -2.327577 3.206867
H 2.923533 -0.635091 3.192645
C -2.635153 2.461757 -0.785867
C -4.152218 1.614598 1.020327
C -2.965080 3.798581 1.307634
H -3.824876 4.313660 0.858823
H -3.111433 3.774598 2.396511
H -2.061227 4.380603 1.087500
H -4.985248 2.104782 0.499970
H -4.103162 0.576216 0.668453
H -4.393783 1.614779 2.092511
H -3.462392 3.020231 -1.244220
H -1.696480 2.976417 -1.025062
H -2.592457 1.465021 -1.239865
H 1.868376 -2.205442 -1.831536
H 0.605572 -2.355696 -0.760894

TS_{ar}^{B}

56
Energy: -4021.57997554
Cu 0.107224 -0.530786 0.539126
Cl -0.290382 -0.811486 2.657061
C 1.942918 -1.145186 0.717130
O -1.623031 0.380548 0.278663
S -1.880283 1.765934 -0.250803
O -2.591639 1.751657 -1.531031
C -3.096663 2.359504 0.985550
O -0.723631 2.654525 -0.139427
F -3.493237 3.579271 0.658798
F -4.142527 1.544669 0.990468
F -2.545188 2.378921 2.182525
C 2.192287 -2.428279 0.242752
C 3.256690 -3.145002 0.785968
C 4.060490 -2.567091 1.762372
C 3.799733 -1.273995 2.218478
C 2.731539 -0.555978 1.704739
H 1.559345 -2.858341 -0.531138
H 3.458440 -4.155267 0.434300
H 4.900402 -3.125241 2.172203
H 4.424552 -0.827785 2.989732
H 2.519518 0.454644 2.049799
C -1.794649 -1.842316 -1.224346
O -0.808919 -2.341672 -0.665191
C -3.208401 -2.290412 -0.886444
N -1.625640 -0.907472 -2.194093
C -0.302861 -0.394655 -2.454470
C 0.591307 -0.184801 -1.277759
C 1.769091 0.098458 -0.903451
C 3.044730 0.735106 -1.017380
C 3.194749 2.102242 -0.671166
C 4.474270 2.673644 -0.826864
C 5.542085 1.922066 -1.271940
C 5.397398 0.562289 -1.579149
C 4.155889 -0.019686 -1.433695
H -2.377751 -0.234671 -2.324856
H 0.238003 -1.089634 -3.112986

H -0.415293 0.557062 -2.990493
N 2.172700 2.831858 -0.154596
H 4.608470 3.725591 -0.575045
H 6.515753 2.398504 -1.377089
H 6.247160 -0.023212 -1.920806
H 4.006807 -1.078702 -1.643313
C -4.289471 -1.388356 -1.475217
C -3.343539 -3.702920 -1.470758
C -3.351568 -2.343896 0.635600
H -4.336041 -4.106767 -1.229009
H -3.236926 -3.695692 -2.565124
H -2.584427 -4.373966 -1.050552
H -4.334404 -2.763170 0.891221
H -2.574996 -2.971881 1.086730
H -3.272720 -1.342632 1.078441
H -5.274716 -1.778186 -1.187406
H -4.222902 -0.358752 -1.095788
H -4.261771 -1.365848 -2.574209
H 2.306397 3.827188 -0.047818
H 1.199392 2.538560 -0.217749

I_2

56
Energy: -4021.63234396
Cu 0.251937 -1.411692 -1.982538
Cl 0.899642 -3.473706 -2.442610
C 2.004058 0.420224 -0.652479
O -0.126060 -1.729341 -0.165838
S -1.635756 -1.784276 0.058449
O -2.352254 -1.886294 -1.214780
C -1.807865 -3.414187 0.884061
O -2.087546 -0.786120 1.021836
F -3.088269 -3.603849 1.166587
F -1.384427 -4.365797 0.081501
F -1.105248 -3.419277 2.002720
C 2.644972 -0.433389 -1.560768
C 3.735746 -1.200892 -1.159467
C 4.206891 -1.106790 0.142809
C 3.597592 -0.229887 1.040961
C 2.506512 0.531024 0.647654
H 2.345897 -0.435692 -2.610853
H 4.207664 -1.874120 -1.872114
H 5.054949 -1.709935 0.461258
H 3.971412 -0.149968 2.060324
H 2.013181 1.198473 1.353766
C -1.130309 -0.678303 -4.163174
O 0.026828 -1.025214 -3.799527
C -1.717952 -1.373619 -5.376029
N -1.858106 0.205882 -3.492168
C -1.476033 0.859491 -2.243906
C -0.162894 0.425375 -1.716867
C 0.759522 1.119297 -1.040515
C 0.590192 2.540766 -0.658866
C -0.530073 2.993261 0.074653
C -0.612713 4.361550 0.383837
C 0.381262 5.246071 0.003139
C 1.505047 4.795957 -0.690589
C 1.598594 3.449860 -1.002888
H -2.808296 0.360469 -3.804633
H -1.413963 1.945025 -2.426789
H -2.285657 0.701264 -1.515607
N -1.548505 2.140400 0.459885
H -1.477955 4.718261 0.944052
H 0.285045 6.299312 0.263066
H 2.294704 5.485976 -0.979149
H 2.468872 3.070023 -1.539201
C -2.906636 -0.631794 -5.979349
C -0.622941 -1.552997 -6.427102
C -2.162020 -2.748982 -4.847755
H -2.583188 -3.332957 -5.677307
H -1.314481 -3.301228 -4.419879
H -2.929172 -2.649532 -4.066947
H -1.025863 -2.127559 -7.271138
H -0.268791 -0.586795 -6.811696
H 0.232113 -2.097550 -6.012500
H -3.231844 -1.157527 -6.885795
H -3.779482 -0.611431 -5.309540
H -2.651638 0.397385 -6.268656
H -2.149823 2.499817 1.190991
H -1.322083 1.160135 0.619050

TS_{rc}^{B}

56
Energy: -4021.60453142
Cu -0.029135 -1.274974 0.356870
Cl 0.572899 -3.332480 -0.171219
C 0.556473 1.863835 1.548718
O -1.772582 -0.624609 0.850255
S -2.760083 -0.567511 -0.306897
O -2.083797 -0.714205 -1.596541
C -3.714157 -2.114779 -0.066876
O -3.723320 0.510537 -0.138051
F -4.636265 -2.205346 -1.012863
F -2.907466 -3.158713 -0.137938
F -4.303025 -2.095508 1.118109
C 0.448791 0.861296 2.522938
C 0.504752 1.170191 3.871497
C 0.664698 2.492123 4.279341
C 0.764902 3.500202 3.325445
C 0.708765 3.191104 1.973711
H 0.300742 -0.172079 2.219680
H 0.410200 0.374836 4.607950
H 0.701959 2.735672 5.339620
H 0.883765 4.536836 3.635203
H 0.787338 3.981844 1.229247
C 2.717246 -1.122547 -0.505518
O 2.060223 -0.728650 0.494085
C 4.030734 -1.850289 -0.326616
N 2.280429 -0.780880 -1.718394
C 0.968044 -0.149074 -1.805285
C 0.637871 0.349417 -0.436701
C 0.540891 1.570694 0.110190
C 0.327002 2.669491 -0.868787
C -0.942963 3.276601 -0.990850
C -1.094462 4.296562 -1.948982
C -0.037969 4.690523 -2.748355
C 1.218732 4.090658 -2.625094
C 1.383190 3.083227 -1.688481
H 2.664888 -1.234737 -2.537416
H 1.003316 0.701580 -2.497861
H 0.220147 -0.865346 -2.177078
N -1.990999 2.927602 -0.181190
H -2.070607 4.770019 -2.054109
H -0.193331 5.479294 -3.482886
H 2.053112 4.413815 -3.243289
H 2.354084 2.599105 -1.562176
C 4.002453 -2.608019 0.999092
C 4.274565 -2.826207 -1.477078
C 5.122724 -0.771824 -0.294524
H 5.182816 -3.405391 -1.268476
H 3.439037 -3.531479 -1.582222
H 4.445851 -2.317233 -2.437572
H 6.097337 -1.250820 -0.132576
H 5.169940 -0.211410 -1.238356
H 4.951432 -0.061205 0.524622
H 4.956731 -3.134130 1.131512
H 3.863525 -1.924620 1.844501
H 3.184985 -3.339999 1.013831
H -1.962263 2.036743 0.302972
H -2.921118 3.110647 -0.536653

Reaction 46

reactant

35
Energy: -728.476448379
C -4.073751 0.043221 0.188489
O -4.648008 0.186030 -0.881999
C -4.857242 -0.160256 1.489978
N -2.715633 0.058949 0.260675
H -2.222268 -0.053534 1.134119
C -1.933096 0.246598 -0.952011
C -0.509505 0.201429 -0.661123
H -2.211091 -0.528089 -1.683444
H -2.208492 1.204657 -1.419461
C 0.673289 0.159367 -0.403121
C 2.068231 0.106981 -0.102590
C 2.642545 -1.079271 0.374476
C 4.000529 -1.104529 0.663637
C 4.790299 0.023340 0.484751
C 4.228320 1.214658 0.006547

C 2.861520 1.244751 -0.281338
H 2.019858 -1.960573 0.510273
H 4.456550 -2.020488 1.036223
H 5.857137 -0.010938 0.708129
N 5.024189 2.329492 -0.224540
H 2.403621 2.160129 -0.656803
C -3.985105 -0.320281 2.731087
C -5.765945 1.058952 1.656364
C -5.712878 -1.414071 1.300358
H -4.623589 -0.460569 3.613867
H -3.330022 -1.201448 2.666020
H -3.367157 0.569744 2.920600
H -6.369001 -1.559616 2.170070
H -6.332711 -1.322283 0.401275
H -5.086485 -2.311199 1.194108
H -6.423357 0.924894 2.526944
H -5.178562 1.974944 1.812856
H -6.385827 1.199344 0.763602
H 4.536758 3.216103 -0.239403
H 5.879264 2.372498 0.314684

I_c

70
Energy: -4329.10511565
Cu 0.720843 0.072425 -0.536087
Cl 1.280303 -0.474587 -2.571976
C 1.951057 1.507740 -0.764534
O -0.564781 -1.474909 -0.366536
S -1.400098 -1.921043 0.801892
O -2.061676 -0.824830 1.513469
C -2.765673 -2.768075 -0.082509
O -0.777479 -2.966088 1.607135
F -3.663076 -3.200581 0.787537
F -3.367427 -1.918580 -0.922219
F -2.303071 -3.790604 -0.781121
C 1.391737 2.750004 -0.536607
C 2.258860 3.845333 -0.466995
C 3.624494 3.681158 -0.651852
C 4.145000 2.414561 -0.907078
C 3.303115 1.305970 -0.971037
C -1.071261 2.979598 2.094832
O -0.221713 3.859301 1.998602
C -2.560375 3.331495 2.081850
N -0.737344 1.667613 2.262229
C 0.642601 1.289715 2.388592
C 1.122636 0.229286 1.478966
C 1.846757 -0.747582 1.236166
C 2.743883 -1.790880 0.899503
C 2.268399 -3.083701 0.649580
C 3.166354 -4.105763 0.335517
C 4.537907 -3.808112 0.291939
C 5.003865 -2.525097 0.551969
C 4.121080 -1.498647 0.851295
H 0.328184 2.896923 -0.370109
H 1.836170 4.822855 -0.240887
H 4.288580 4.542129 -0.600492
H 5.213004 2.279405 -1.072887
H 3.698806 0.316982 -1.195596
H -1.426272 0.923440 2.177696
H 1.233688 2.201221 2.210732
H 0.863560 0.947025 3.412102
H 1.198697 -3.279296 0.713718
N 2.708237 -5.373100 0.036334
H 5.243466 -4.602171 0.045341
H 6.073701 -2.328579 0.517371
H 4.467791 -0.489985 1.065217
C -3.488483 2.133706 2.246906
C -2.781930 4.311401 3.237465
C -2.846807 4.035522 0.754516
O -3.295680 1.073949 -0.970598
C -4.517755 0.747007 -1.645642
C -5.583518 0.680370 -0.584135
C -2.098959 0.910905 -1.524235
O -1.130428 1.224925 -0.848288
C -2.013734 0.363968 -2.912178
H -4.729212 1.526559 -2.392000
H -4.414969 -0.213576 -2.163985
H -6.552173 0.424835 -1.028499
H -5.321634 -0.083389 0.158808
H -5.676624 1.644434 -0.069492
H -2.774533 0.794583 -3.572300

H -1.013890 0.544527 -3.311461
H -2.163919 -0.723616 -2.873797
H -3.316994 1.607414 3.196740
H -4.530815 2.484325 2.255384
H -3.385080 1.414499 1.424114
H -3.895188 4.367315 0.724193
H -2.198104 4.911583 0.634742
H -2.678250 3.355724 -0.091009
H -3.823298 4.663403 3.234191
H -2.591686 3.830192 4.207529
H -2.115513 5.176759 3.147131
H 3.355875 -6.137325 0.170729
H 1.763337 -5.587190 0.327102

I_1

56
Energy: -4021.58309435
Cu 0.003767 0.118877 -0.001494
Cl -0.009289 0.362120 2.117353
C 1.868560 0.183886 0.310000
O -2.014826 0.307022 -0.314810
S -2.039016 1.687693 -0.929253
O -2.684898 1.778619 -2.225953
C -3.092354 2.631159 0.238486
O -0.685468 2.267787 -0.795657
F -3.152633 3.892581 -0.159057
F -4.310535 2.113625 0.247431
F -2.581973 2.577345 1.454557
C 2.623441 -0.961514 0.461473
C 4.009191 -0.813944 0.376158
C 4.579067 0.440579 0.173592
C 3.773885 1.568774 0.059631
C 2.385453 1.455449 0.151811
H 2.166905 -1.940589 0.611667
H 4.639637 -1.696689 0.472618
H 5.661702 0.540293 0.117814
H 4.216234 2.552867 -0.086837
H 1.742353 2.332459 0.094380
C -0.448462 -3.199382 0.715318
O 0.619194 -3.562693 0.234842
C -0.847122 -3.542217 2.148814
N -1.358146 -2.529520 -0.055228
C -1.041789 -2.237446 -1.433440
C -0.079361 -1.132854 -1.634837
C 0.646677 -0.341949 -2.238758
C 1.578451 0.512851 -2.882720
C 1.189076 1.796455 -3.282052
C 2.114328 2.646842 -3.891560
C 3.420007 2.176033 -4.102930
C 3.793664 0.895030 -3.716740
C 2.884466 0.047620 -3.100847
H -2.145247 -2.046674 0.359565
H -0.612438 -3.137605 -1.891176
H -1.970935 -1.980928 -1.955081
H 0.171891 2.132170 -3.083614
N 1.766480 3.940930 -4.231598
H 4.149115 2.836755 -4.573344
H 4.813771 0.559817 -3.894781
H 3.163907 -0.950703 -2.772486
C -2.071046 -2.779183 2.647965
C -1.145761 -5.046267 2.147608
C 0.352049 -3.258424 3.052975
H 2.301304 4.374359 -4.971994
H 0.775666 4.137088 -4.286798
H 0.128283 -3.580934 4.079162
H 1.237404 -3.800957 2.701484
H 0.587432 -2.185260 3.075162
H -1.397183 -5.376311 3.165116
H -1.997212 -5.283485 1.493571
H -0.274462 -5.615440 1.801054
H -2.259647 -3.041802 3.697455
H -1.923973 -1.690266 2.604866
H -2.980269 -3.043115 2.087846

TS_{rc}^{A}

56
Energy: -4021.56090347
Cu -0.374806 -0.738149 -0.737112
Cl -0.023625 -1.325118 -2.763357
C -1.742221 -2.031794 -0.687679

O 0.794893 0.866334 -0.830263
S 1.594459 1.767188 0.071754
O 3.006027 1.389874 0.143281
C 1.547979 3.317167 -0.909224
O 0.947475 2.103467 1.338877
F 2.211364 4.263712 -0.263751
F 2.091870 3.132797 -2.097507
F 0.284465 3.713192 -1.067612
C -1.452937 -3.329703 -0.306347
C -2.503603 -4.245166 -0.247086
C -3.798627 -3.856008 -0.574299
C -4.053607 -2.546219 -0.965964
C -3.016109 -1.616525 -1.032285
H -0.436889 -3.628492 -0.057976
H -2.298696 -5.271523 0.054783
H -4.611766 -4.578484 -0.529382
H -5.063565 -2.236320 -1.230062
H -3.216935 -0.590390 -1.340425
C 2.280099 -1.541945 1.110945
O 1.171242 -1.966084 0.724843
C 3.569114 -1.915916 0.412981
N 2.277254 -0.814108 2.243584
C 0.972914 -0.648342 2.865105
C -0.070718 -0.680100 1.835563
C -0.960038 -0.246041 1.052018
C -2.116272 0.635121 1.144518
C -2.230727 1.708575 0.262721
C -3.320578 2.579648 0.347283
C -4.319558 2.320172 1.296998
C -4.211358 1.229559 2.148143
C -3.111724 0.380320 2.092285
H 3.035810 -0.175479 2.451520
H 0.820412 -1.427048 3.626251
H 0.918058 0.342567 3.336259
H -1.445087 1.895614 -0.468802
N -3.425314 3.643392 -0.531844
H -5.184113 2.981626 1.357356
H -4.997888 1.042434 2.877523
H -3.025716 -0.479288 2.753410
C 3.416920 -1.659552 -1.087141
C 4.775251 -1.162559 0.964963
C 3.741486 -3.421341 0.669044
H 4.661146 -3.763811 0.176667
H 3.829443 -3.641488 1.742672
H 2.898619 -3.992207 0.261379
H 5.674056 -1.492134 0.428271
H 4.675599 -0.080578 0.813097
H 4.942586 -1.376404 2.031155
H 4.327161 -1.999801 -1.598981
H 2.561011 -2.202725 -1.506776
H 3.278883 -0.591264 -1.293096
H -4.013626 4.409428 -0.231707
H -2.552921 3.956927 -0.939402

TS_{ar}^{B}

56
Energy: -4021.57201022
Cu 0.103088 -0.723842 0.459693
Cl -0.197278 -1.038023 2.583068
C 1.817299 -1.648945 0.481688
O -1.491221 0.476483 0.346410
S -1.542385 1.892720 -0.168323
O -2.207657 1.979596 -1.473601
C -2.720838 2.629663 1.028054
O -0.294460 2.628159 0.003013
F -2.907256 3.905382 0.725199
F -3.884205 1.993528 0.962076
F -2.238166 2.533543 2.251840
C 1.839906 -2.870516 -0.178270
C 2.803865 -3.807385 0.189679
C 3.733290 -3.503225 1.177897
C 3.702813 -2.262128 1.812651
C 2.740574 -1.322662 1.469309
H 1.110783 -3.095209 -0.953620
H 2.826937 -4.773876 -0.310578
H 4.491407 -4.234088 1.453391
H 4.424953 -2.024392 2.591372
H 2.709888 -0.351213 1.958572
C -2.112065 -1.670006 -1.147182
O -1.180018 -2.306476 -0.635492
C -3.552910 -1.888421 -0.720741

N -1.856516 -0.801802 -2.160958
C -0.479710 -0.574998 -2.519555
C 0.479548 -0.333621 -1.414162
C 1.626855 -0.141816 -0.929210
C 2.871068 0.596980 -0.879218
C 2.805722 1.954949 -0.570004
C 3.978806 2.720263 -0.552277
C 5.193744 2.094322 -0.863384
C 5.239981 0.738645 -1.167631
C 4.086840 -0.033252 -1.167217
H -2.461889 0.013573 -2.243172
H -0.097852 -1.448846 -3.068572
H -0.443553 0.288771 -3.196719
H 1.839596 2.407205 -0.341934
N 3.935082 4.052108 -0.190166
H 6.112101 2.682089 -0.853125
H 6.197973 0.278496 -1.403170
H 4.113729 -1.100429 -1.380311
C -4.509968 -0.841067 -1.282382
C -3.933821 -3.273789 -1.261336
C -3.617717 -1.895424 0.807495
H -4.963761 -3.512028 -0.962973
H -3.882560 -3.303872 -2.359285
H -3.268492 -4.047343 -0.858768
H -4.637296 -2.154706 1.124078
H -2.921768 -2.628402 1.231330
H -3.359745 -0.913007 1.222778
H -5.525449 -1.055860 -0.924567
H -4.254868 0.174468 -0.949354
H -4.544043 -0.857204 -2.381104
H 4.691289 4.638916 -0.514896
H 3.028789 4.499546 -0.231492

I_2

56
Energy: -4021.62384106
Cu 0.205707 -1.472885 -1.942117
Cl 0.768691 -3.571324 -2.328541
C 2.082971 0.375516 -0.728589
O -0.185396 -1.692717 -0.116941
S -1.692493 -1.563118 0.113926
O -2.428950 -1.789444 -1.132987
C -1.995930 -3.032889 1.170948
O -2.051132 -0.398192 0.911111
F -3.285809 -3.064427 1.473600
F -1.667873 -4.130534 0.523390
F -1.286973 -2.935054 2.281264
C 2.673022 -0.544495 -1.606394
C 3.774388 -1.295898 -1.207177
C 4.310456 -1.118858 0.061577
C 3.751676 -0.178344 0.926722
C 2.647193 0.565653 0.536859
H 2.324264 -0.615747 -2.638159
H 4.205606 -2.020503 -1.894885
H 5.169255 -1.707723 0.378212
H 4.174841 -0.034270 1.919384
H 2.191666 1.280041 1.221538
C -1.097184 -0.711122 -4.175140
O 0.021116 -1.130869 -3.770979
C -1.665048 -1.358266 -5.423997
N -1.799990 0.211684 -3.528917
C -1.431134 0.842509 -2.261549
C -0.120763 0.387570 -1.742631
C 0.836036 1.068785 -1.108426
C 0.642028 2.481969 -0.699425
C -0.455998 2.807632 0.096946
C -0.655422 4.125370 0.529692
C 0.256976 5.109576 0.126081
C 1.351189 4.775428 -0.661718
C 1.563504 3.464527 -1.072921
H -2.725215 0.421696 -3.881228
H -1.369917 1.930509 -2.423913
H -2.247722 0.648933 -1.550968
H -1.130821 2.015122 0.430523
N -1.698978 4.432669 1.384073
H 0.112419 6.138681 0.456705
H 2.052552 5.555096 -0.955507
H 2.426349 3.197385 -1.681071
C -2.845982 -0.596390 -6.016626
C -0.551066 -1.485926 -6.463558
C -2.112898 -2.755657 -4.963688

H -2.512968 -3.307630 -5.824916
H -1.273453 -3.320409 -4.537208
H -2.899390 -2.690730 -4.198636
H -0.935033 -2.028625 -7.337023
H -0.199738 -0.501740 -6.802664
H 0.301901 -2.040196 -6.058134
H -3.162630 -1.093607 -6.941952
H -3.724414 -0.595700 -5.353780
H -2.586588 0.440415 -6.274011
H -2.006959 5.395181 1.399797
H -2.456168 3.764263 1.431733

TS_{rc}^{B}

56
Energy: -4021.59975161
Cu -0.770897 1.147826 0.366534
Cl -2.164650 2.725286 -0.317493
C 0.141732 -1.890332 1.612814
O 1.020390 1.410481 1.015869
S 2.077189 1.640588 -0.052523
O 1.525424 1.505128 -1.401455
C 2.426752 3.428722 0.156697
O 3.336555 0.971124 0.248964
F 3.356191 3.791579 -0.715671
F 1.330439 4.135845 -0.055179
F 2.866333 3.659478 1.383346
C -0.368617 -0.999454 2.567664
C -0.374238 -1.327515 3.912797
C 0.130129 -2.555246 4.335288
C 0.644916 -3.447930 3.400566
C 0.656196 -3.118155 2.052305
H -0.742373 -0.028266 2.252945
H -0.762584 -0.614276 4.636957
H 0.130611 -2.809770 5.393645
H 1.047170 -4.406528 3.723003
H 1.062807 -3.817462 1.323695
C -3.018096 -0.248924 -0.713898
O -2.354114 -0.275932 0.357080
C -4.529894 -0.231275 -0.675846
N -2.361106 -0.352320 -1.868324
C -0.902557 -0.310118 -1.829908
C -0.494811 -0.578213 -0.421025
C 0.134213 -1.594605 0.178136
C 1.048315 -2.354139 -0.727438
C 2.391016 -2.002722 -0.691924
C 3.309100 -2.610212 -1.556141
C 2.847151 -3.608347 -2.426729
C 1.504815 -3.967054 -2.438299
C 0.583829 -3.340499 -1.601645
H -2.837173 -0.149651 -2.738102
H -0.484519 -1.100994 -2.468015
H -0.535959 0.661626 -2.192320
H 2.728223 -1.220858 -0.010341
N 4.637121 -2.235916 -1.507320
H 3.550610 -4.099694 -3.099452
H 1.170299 -4.749107 -3.118598
H -0.470966 -3.615070 -1.613633
C -4.986729 0.545348 0.557886
C -5.106658 0.410867 -1.936117
C -4.973339 -1.697348 -0.570930
H -6.196538 0.484202 -1.833553
H -4.713624 1.426282 -2.081954
H -4.920526 -0.187289 -2.840851
H -6.068763 -1.738561 -0.508461
H -4.658312 -2.280705 -1.447145
H -4.561171 -2.170602 0.329550
H -6.084011 0.554481 0.589205
H -4.617925 0.079920 1.478852
H -4.619574 1.579122 0.528421
H 5.183037 -2.411510 -2.340465
H 4.807123 -1.319475 -1.107249

Reaction 47

reactant

35
Energy: -728.477950385
C -4.068105 -0.099115 0.180881
O -4.651712 -0.089575 -0.894345
C -4.842107 -0.066974 1.503752

N -2.710296 -0.128151 0.240540
H -2.210688 -0.143849 1.117576
C -1.932155 -0.145697 -0.990593
C -0.507626 -0.095542 -0.707833
H -2.185715 -1.046949 -1.570520
H -2.241406 0.703683 -1.618729
C 0.678076 -0.053500 -0.458389
C 2.072122 0.001173 -0.170332
C 2.835358 -1.171132 -0.062996
C 4.188666 -1.118934 0.219094
C 4.831964 0.113144 0.404678
C 4.074582 1.288459 0.298454
C 2.721747 1.230785 0.015511
H 2.346327 -2.133656 -0.204451
H 4.767538 -2.040104 0.292998
N 6.195695 0.170026 0.637780
H 4.563948 2.253393 0.434516
H 2.143441 2.149883 -0.064777
C -3.963588 -0.125480 2.749257
C -5.650802 1.231835 1.508744
C -5.795459 -1.262791 1.493212
H -4.596305 -0.106123 3.647085
H -3.370243 -1.050515 2.793848
H -3.284162 0.736822 2.818177
H -6.448688 -1.234088 2.376756
H -6.418401 -1.247723 0.591831
H -5.242714 -2.212841 1.510964
H -6.297468 1.272065 2.396691
H -4.991108 2.111097 1.529170
H -6.277680 1.295968 0.612009
H 6.536619 1.015492 1.076063
H 6.616238 -0.666424 1.020695

I_c

70
Energy: -4329.11281686
Cu 0.692821 0.112579 -0.519019
Cl 1.352382 -0.474495 -2.532189
C 1.872684 1.596469 -0.610859
O -0.569832 -1.459851 -0.376705
S -1.417467 -1.872414 0.794262
O -2.164078 -0.768036 1.403149
C -2.702343 -2.860730 -0.063059
O -0.768180 -2.819062 1.695474
F -3.587677 -3.301803 0.815109
F -3.341963 -2.104212 -0.961702
F -2.157363 -3.888363 -0.690978
C 1.278635 2.814496 -0.339927
C 2.094736 3.947696 -0.289534
C 3.458739 3.845729 -0.526038
C 4.022571 2.605136 -0.814944
C 3.227747 1.461381 -0.858608
C -0.989887 3.000408 2.260116
O -0.094698 3.821604 2.426664
C -2.440388 3.451360 2.065014
N -0.740937 1.660692 2.213085
C 0.587994 1.164595 2.443118
C 1.094848 0.193171 1.442376
C 1.854679 -0.791101 1.290791
C 2.725519 -1.830230 0.976260
C 2.255896 -3.155617 0.804007
C 3.128348 -4.156146 0.454682
C 4.499877 -3.881689 0.268215
C 4.976519 -2.566157 0.458598
C 4.105144 -1.561705 0.798965
H 0.215223 2.903905 -0.137003
H 1.638551 4.903187 -0.034661
H 4.087547 4.733431 -0.485112
H 5.088801 2.519985 -1.021728
H 3.657000 0.494845 -1.117204
H -1.472540 0.980337 2.021037
H 1.248651 2.044575 2.448506
H 0.664049 0.686528 3.432409
H 1.195199 -3.355499 0.953008
H 2.767155 -5.174484 0.314606
N 5.360789 -4.881345 -0.060101
H 6.038126 -2.358952 0.328256
H 4.464498 -0.543506 0.941773
C -3.460885 2.320461 2.146018
C -2.742621 4.492613 3.142771
C -2.505690 4.110533 0.683796

O -3.336673 1.102867 -1.077499
C -4.544784 0.628793 -1.686179
C -5.561936 0.504655 -0.582059
C -2.132051 0.870531 -1.591965
O -1.170043 1.243455 -0.938728
C -2.028486 0.191243 -2.919566
H -4.855195 1.349491 -2.456731
H -4.370807 -0.340580 -2.167387
H -6.515884 0.138645 -0.978906
H -5.200098 -0.199360 0.177727
H -5.731796 1.473936 -0.098889
H -2.758161 0.584543 -3.636516
H -1.011773 0.307489 -3.301745
H -2.213037 -0.883012 -2.790759
H -3.410274 1.791487 3.108507
H -4.472644 2.742103 2.055991
H -3.339991 1.587450 1.337653
H -3.512062 4.518269 0.509045
H -1.780333 4.932190 0.614430
H -2.290405 3.380093 -0.108032
H -3.742375 4.919458 2.979857
H -2.726809 4.043072 4.145755
H -2.003210 5.300559 3.121001
H 6.296260 -4.655930 -0.366206
H 4.995301 -5.773034 -0.362164

I_1

56
Energy: -4021.59070808
Cu 0.163779 0.047457 0.229664
Cl 0.208776 0.337292 2.349911
C 1.979431 -0.412540 0.376139
O -1.729163 0.656999 -0.081670
S -1.557595 2.028967 -0.702248
O -2.210823 2.191566 -1.992718
C -2.480026 3.095848 0.469177
O -0.148521 2.437206 -0.593384
F -2.393738 4.354548 0.063797
F -3.752002 2.729833 0.493711
F -1.967045 2.988565 1.679671
C 2.402693 -1.725160 0.477176
C 3.777134 -1.964888 0.432264
C 4.677948 -0.910811 0.304742
C 4.213515 0.397958 0.225156
C 2.843190 0.663134 0.265164
H 1.700624 -2.555774 0.571608
H 4.136615 -2.990826 0.498113
H 5.747932 -1.111023 0.280576
H 4.914326 1.228225 0.145452
H 2.472171 1.687533 0.225454
C -1.023926 -3.186045 0.698829
O -0.119374 -3.956813 0.400195
C -1.749444 -3.294707 2.038702
N -1.429585 -2.201608 -0.162480
C -0.796190 -2.038550 -1.450099
C 0.066181 -0.828702 -1.545036
C 0.695885 -0.071775 -2.314446
C 1.487048 0.830432 -3.006139
C 0.938193 2.029527 -3.526233
C 1.736471 2.922506 -4.194427
C 3.115010 2.662825 -4.361113
C 3.669701 1.472607 -3.838428
C 2.870525 0.575731 -3.175279
H -2.151798 -1.539900 0.097287
H -0.176198 -2.932705 -1.600574
H -1.553513 -1.998276 -2.244221
H -0.120704 2.231973 -3.370669
H 1.317386 3.846096 -4.591879
N 3.898042 3.543123 -5.033853
H 4.734467 1.279785 -3.964803
H 3.290216 -0.337551 -2.754746
C -2.471379 -4.645497 2.032989
C -0.673582 -3.288950 3.127710
C -2.753231 -2.175584 2.301181
H 4.901315 3.432419 -5.040391
H 3.537947 4.451246 -5.288617
H -3.203439 -2.321114 3.292258
H -2.279757 -1.182822 2.304520
H -3.580147 -2.179237 1.575222
H -1.140951 -3.434040 4.111550
H 0.049588 -4.095210 2.959241

H -0.132655 -2.331808 3.144647
H -2.948808 -4.816003 3.007856
H -3.255394 -4.677063 1.262586
H -1.765483 -5.462138 1.840497

TS_{rc}^{A}

56
Energy: -4021.56036203
Cu -0.023012 0.004599 -0.007954
Cl -0.038366 -0.040271 2.133484
C -1.468745 1.209742 0.084763
O 1.301547 -1.458747 -0.172691
S 2.215682 -2.096969 -1.185200
O 3.615749 -1.715770 -0.995465
C 2.102589 -3.848460 -0.654782
O 1.720418 -2.083296 -2.560632
F 2.848984 -4.596860 -1.453666
F 2.524894 -3.983189 0.589942
F 0.841315 -4.257140 -0.734476
C -1.213009 2.569038 0.137562
C -2.298813 3.442443 0.189832
C -3.600416 2.951890 0.198150
C -3.825351 1.580348 0.156506
C -2.751363 0.691671 0.104764
H -0.193891 2.948263 0.136727
H -2.116554 4.515685 0.228648
H -4.442328 3.640602 0.243781
H -4.841066 1.187771 0.170969
H -2.932520 -0.382614 0.077772
C 2.758091 1.395325 -1.116715
O 1.582191 1.683600 -0.813852
C 3.903582 1.570455 -0.142872
N 2.969423 1.000034 -2.386319
C 1.786077 0.992719 -3.232869
C 0.599216 0.706206 -2.422610
C -0.315978 -0.017520 -1.927873
C -1.311274 -0.938564 -2.435878
C -1.649094 -2.090338 -1.710870
C -2.567581 -2.995916 -2.205073
C -3.202849 -2.769697 -3.435709
C -2.879838 -1.609417 -4.154779
C -1.944869 -0.715964 -3.664550
H 3.783440 0.445534 -2.625670
H 1.705989 1.951181 -3.765752
H 1.865811 0.172093 -3.960353
H -1.143427 -2.302239 -0.767584
H -2.804692 -3.894404 -1.635632
N -4.163466 -3.644573 -3.902954
H -3.374412 -1.414049 -5.106454
H -1.706693 0.183742 -4.230681
C 3.561315 0.851692 1.163820
C 5.229518 1.060389 -0.698887
C 3.996758 3.083477 0.105647
H 4.808413 3.278745 0.818817
H 4.219753 3.632679 -0.820385
H 3.063612 3.473884 0.528914
H 6.016670 1.238195 0.045011
H 5.194524 -0.019368 -0.891994
H 5.525311 1.594224 -1.614226
H 4.359430 1.045533 1.893110
H 2.613352 1.207896 1.586253
H 3.482260 -0.231598 1.011942
H -4.351505 -3.619645 -4.896303
H -4.137585 -4.583045 -3.526759

TS_{ar}^{B}

56
Energy: -4021.57523604
Cu -0.020220 -0.749348 0.466390
Cl -0.409705 -1.223091 2.550570
C 1.684289 -1.690342 0.468248
O -1.552939 0.527040 0.388453
S -1.517448 1.966840 -0.055583
O -2.109851 2.151990 -1.386024
C -2.732987 2.679966 1.117097
O -0.251145 2.638545 0.216309
F -2.888549 3.969578 0.859541
F -3.901712 2.064634 0.973209
F -2.308810 2.526345 2.356633
C 1.711507 -2.878139 -0.253247

C 2.625376 -3.864602 0.112017
C 3.507584 -3.644222 1.164091
C 3.479186 -2.437387 1.862952
C 2.564909 -1.451223 1.519958
H 1.017793 -3.039014 -1.076162
H 2.647472 -4.803706 -0.437994
H 4.226233 -4.413736 1.440382
H 4.162335 -2.266380 2.692870
H 2.533859 -0.508106 2.061845
C -2.210746 -1.500746 -1.262843
O -1.336857 -2.212784 -0.748535
C -3.679896 -1.663066 -0.912418
N -1.866910 -0.594552 -2.215748
C -0.465667 -0.420352 -2.499869
C 0.443862 -0.250293 -1.338005
C 1.608776 -0.110563 -0.862836
C 2.843670 0.608898 -0.767914
C 2.799778 2.008225 -0.644457
C 3.970547 2.739884 -0.615698
C 5.216982 2.099596 -0.710423
C 5.255891 0.696560 -0.828592
C 4.088782 -0.036136 -0.844568
H -2.427081 0.254135 -2.276352
H -0.097872 -1.296033 -3.055446
H -0.357450 0.458365 -3.149547
H 1.831456 2.500125 -0.553022
H 3.931911 3.823709 -0.508756
N 6.380734 2.821732 -0.643751
H 6.220168 0.193271 -0.897408
H 4.127177 -1.121627 -0.929293
C -4.559982 -0.555980 -1.485538
C -4.094727 -3.013161 -1.513371
C -3.824828 -1.709133 0.609657
H -5.149689 -3.209771 -1.278794
H -3.983635 -3.016203 -2.607400
H -3.488801 -3.827761 -1.098353
H -4.860462 -1.973141 0.864843
H -3.152730 -2.453492 1.052111
H -3.590303 -0.736792 1.060148
H -5.600882 -0.735480 -1.185673
H -4.278174 0.435639 -1.103814
H -4.537754 -0.534663 -2.584466
H 7.233529 2.385371 -0.964172
H 6.330781 3.819181 -0.796219

I_2

56
Energy: -4021.62482768
Cu 0.272545 -1.433215 -1.966518
Cl 0.924621 -3.500729 -2.396610
C 2.076849 0.400891 -0.730943
O -0.149837 -1.703709 -0.151183
S -1.667350 -1.655248 0.037631
O -2.354588 -1.884668 -1.237142
C -1.926183 -3.168976 1.043068
O -2.107119 -0.531947 0.852191
F -3.221029 -3.276386 1.304850
F -1.521957 -4.229384 0.376204
F -1.256542 -3.068667 2.177703
C 2.689560 -0.460149 -1.651988
C 3.789423 -1.228405 -1.277817
C 4.298344 -1.128255 0.009525
C 3.715181 -0.247086 0.920845
C 2.614260 0.513596 0.555708
H 2.358985 -0.469896 -2.692111
H 4.237040 -1.908745 -1.999323
H 5.153431 -1.732288 0.306991
H 4.115836 -0.165336 1.929846
H 2.138144 1.179247 1.274502
C -1.106648 -0.711003 -4.161043
O 0.042033 -1.077194 -3.794833
C -1.704819 -1.404601 -5.370332
N -1.820896 0.194041 -3.500644
C -1.444169 0.832763 -2.240420
C -0.117440 0.412772 -1.736072
C 0.827023 1.105427 -1.090330
C 0.646681 2.511963 -0.680036
C -0.523328 2.933684 -0.036639
C -0.688059 4.252605 0.355299
C 0.319228 5.197638 0.116811
C 1.500423 4.775238 -0.513411

C 1.660472 3.455061 -0.894031
H -2.770872 0.351161 -3.812449
H -1.408952 1.922605 -2.403113
H -2.248063 0.622937 -1.518940
H -1.293711 2.199910 0.204105
H -1.595192 4.557598 0.877736
N 0.180470 6.503578 0.548372
H 2.298865 5.496655 -0.689919
H 2.586571 3.140343 -1.375464
C -2.886132 -0.653265 -5.976247
C -0.613340 -1.600404 -6.422152
C -2.162728 -2.772659 -4.835308
H -2.586834 -3.358007 -5.662457
H -1.321136 -3.329305 -4.401463
H -2.930781 -2.661873 -4.057108
H -1.023138 -2.174818 -7.263033
H -0.249642 -0.639685 -6.811765
H 0.236510 -2.151634 -6.005787
H -3.218535 -1.180489 -6.879255
H -3.757680 -0.619592 -5.305258
H -2.620225 0.371463 -6.271735
H 0.767095 7.193079 0.098055
H -0.763363 6.827551 0.711283

TS_{rc}^{B}

56
Energy: -4021.59795156
Cu -1.032794 1.166579 0.162915
Cl -2.737181 2.122835 -0.858329
C 0.581059 -1.289091 1.915023
O 0.565768 1.956045 0.853982
S 1.684079 2.099037 -0.166151
O 1.303588 1.557996 -1.473119
C 1.701638 3.916547 -0.400804
O 2.985987 1.744320 0.377832
F 2.619971 4.234317 -1.301732
F 0.514983 4.320008 -0.827241
F 1.984448 4.520760 0.741995
C -0.170100 -0.406338 2.705177
C -0.122000 -0.471134 4.087028
C 0.681086 -1.419670 4.716030
C 1.434872 -2.300867 3.947976
C 1.387385 -2.236686 2.562047
H -0.781091 0.353970 2.227179
H -0.705191 0.233140 4.676885
H 0.723724 -1.465160 5.802828
H 2.067792 -3.043784 4.430038
H 1.976063 -2.932633 1.967310
C -2.840870 -0.977360 -0.586357
O -2.214834 -0.645432 0.452960
C -4.310306 -1.329765 -0.517855
N -2.153057 -1.117511 -1.721449
C -0.767848 -0.660360 -1.725359
C -0.333345 -0.568382 -0.300022
C 0.522563 -1.277174 0.450003
C 1.547853 -2.026396 -0.327214
C 2.868474 -1.565685 -0.385919
C 3.824803 -2.270170 -1.095973
C 3.491726 -3.453354 -1.772222
C 2.166392 -3.910785 -1.716574
C 1.211268 -3.201182 -1.005727
H -2.650253 -1.181019 -2.600700
H -0.130181 -1.385748 -2.245787
H -0.686606 0.304061 -2.246942
H 3.135378 -0.632782 0.113940
H 4.849124 -1.899890 -1.139257
N 4.459963 -4.178166 -2.440917
H 1.896890 -4.837347 -2.224332
H 0.184225 -3.569401 -0.962103
C -4.977839 -0.458923 0.545285
C -4.995772 -1.117999 -1.866124
C -4.378662 -2.807820 -0.109358
H -6.071847 -1.300459 -1.753816
H -4.865812 -0.086709 -2.222042
H -4.641211 -1.818995 -2.636730
H -5.430596 -3.108248 -0.015279
H -3.902977 -3.457288 -0.857332
H -3.887233 -2.972243 0.858103
H -6.042680 -0.719139 0.605555
H -4.525395 -0.618802 1.530389
H -4.883060 0.605569 0.295760

H 4.134574 -4.813618 -3.157118
H 5.292896 -3.670951 -2.709346

Reaction 48

reactant

33
Energy: -673.153098765
C -4.058140 0.007907 0.185254
O -4.640120 -0.004525 -0.890334
C -4.826386 -0.098931 1.507049
N -2.702046 0.101671 0.245077
H -2.203941 0.131332 1.122610
C -1.933000 0.190144 -0.987062
C -0.506143 0.140715 -0.713158
H -2.236614 -0.631924 -1.652685
H -2.195413 1.115676 -1.523528
C 0.679887 0.099636 -0.470043
C 2.078127 0.043723 -0.188988
C 2.705474 -1.189283 0.039155
C 4.065012 -1.242686 0.313779
C 4.815560 -0.070836 0.363510
C 4.200951 1.158004 0.137375
C 2.841405 1.218610 -0.136610
H 2.110588 -2.099981 -0.003969
H 4.543148 -2.204873 0.489197
H 5.881907 -0.115348 0.578319
H 4.785526 2.075640 0.174760
H 2.352119 2.174570 -0.315095
C -3.950746 -0.008699 2.752729
C -5.855280 1.032345 1.521011
C -5.548880 -1.447690 1.485909
H -4.579426 -0.082806 3.650413
H -3.219739 -0.828901 2.806363
H -3.415406 0.950220 2.813431
H -6.188135 -1.548534 2.374304
H -6.174006 -1.533587 0.589728
H -4.833334 -2.282349 1.486159
H -6.503728 0.942654 2.403844
H -5.365812 2.015900 1.558607
H -6.477509 0.995508 0.619835

I_c

68
Energy: -6844.41758839
Cu -1.045056 0.000973 -0.578855
Cl -1.446799 0.256586 -2.693992
C -2.034844 -1.603389 -0.891811
O -0.623215 1.929790 -0.345354
S 0.296035 2.691505 0.575246
O 1.286213 1.863274 1.260562
C 1.261323 3.665757 -0.647184
O -0.411806 3.690438 1.366858
F 2.234717 4.318686 -0.029428
F 1.809232 2.858816 -1.559525
F 0.479528 4.530169 -1.268609
C -1.481593 -2.873853 -0.944909
C -2.323079 -3.990516 -0.973830
C -3.699716 -3.833038 -0.986739
C -4.248342 -2.554282 -0.984084
C -3.414265 -1.442877 -0.954708
C 2.135821 -1.935059 2.358935
O 1.794225 -3.061225 2.701117
C 3.609153 -1.551332 2.207683
N 1.208775 -0.989339 2.049476
C -0.172943 -1.383045 2.099254
C -1.095231 -0.439314 1.442709
C -2.101071 0.284742 1.459224
C -3.323243 0.995283 1.438976
C -3.370467 2.397980 1.337414
C -4.602283 3.034191 1.351070
C -5.777699 2.296278 1.476825
C -5.735678 0.907040 1.592556
C -4.515883 0.252787 1.569253
Br 0.379018 -3.224778 -0.962588
H -1.871325 -4.980305 -0.986747
H -4.341816 -4.710916 -1.017718
H -5.326923 -2.411289 -1.026878
H -3.846129 -0.443726 -1.002596
H 1.444528 -0.057961 1.712587

H -0.281934 -2.380537 1.643437
H -0.515851 -1.493134 3.141554
H -2.444410 2.965184 1.254702
H -4.644916 4.117850 1.267770
H -6.738045 2.809087 1.489453
H -6.656405 0.337653 1.700912
H -4.458318 -0.830847 1.657757
C 3.853011 -0.044875 2.196794
C 4.384382 -2.191513 3.356818
C 4.063379 -2.165015 0.878647
O 3.179212 0.479296 -1.141182
C 4.231823 0.962557 -1.980944
C 5.152214 1.754535 -1.089909
C 2.023108 0.029282 -1.625338
O 1.151155 -0.232237 -0.818487
C 1.874156 -0.139826 -3.104917
H 4.746866 0.107921 -2.444549
H 3.813151 1.591095 -2.778743
H 5.979413 2.179822 -1.669454
H 4.596236 2.570109 -0.611970
H 5.568123 1.115459 -0.301824
H 2.794897 -0.496580 -3.578716
H 1.052413 -0.831882 -3.302597
H 1.603980 0.825497 -3.554685
H 3.443183 0.441266 3.093184
H 4.936500 0.144754 2.184831
H 3.424144 0.448980 1.314768
H 5.125608 -1.939174 0.699945
H 3.935032 -3.255530 0.893088
H 3.481522 -1.754971 0.041257
H 5.463481 -2.041780 3.210582
H 4.108239 -1.740804 4.320262
H 4.177563 -3.265597 3.414606

I_1

54
Energy: -6536.90862787
Cu 0.060652 0.231038 0.256307
Cl -0.949639 0.273525 2.132239
C 0.999070 -1.148428 1.139237
O -0.689707 1.955030 -0.495429
S 0.522575 2.860536 -0.398981
O 0.912639 3.496817 -1.642205
C -0.066452 4.198804 0.709448
O 1.559131 2.138294 0.366219
F 0.915315 5.067618 0.887903
F -1.100409 4.806727 0.152468
F -0.425568 3.693733 1.874730
C 0.684335 -2.489733 1.024249
C 1.586907 -3.436099 1.507743
C 2.765460 -3.017677 2.115744
C 3.049747 -1.661694 2.245186
C 2.149198 -0.708082 1.772927
Br -0.904831 -3.034565 0.171653
H 1.350354 -4.493360 1.402753
H 3.460572 -3.760905 2.501593
H 3.966015 -1.332997 2.731769
H 2.344138 0.356853 1.894100
C -3.278162 -0.997608 -1.218586
O -3.248640 -2.137881 -1.662041
C -4.400796 -0.531176 -0.290681
N -2.297354 -0.088917 -1.511852
C -1.191765 -0.435413 -2.367230
C 0.132269 -0.415725 -1.705644
C 1.363013 -0.414611 -1.619577
C 2.767995 -0.547459 -1.481760
C 3.594981 0.586039 -1.432990
C 4.962105 0.423117 -1.268411
C 5.506861 -0.855139 -1.161090
C 4.690228 -1.983165 -1.223522
C 3.321794 -1.834786 -1.382509
H -2.348205 0.866941 -1.180199
H -1.387549 -1.454726 -2.729312
H -1.143172 0.240794 -3.232774
H 3.147257 1.574734 -1.504290
H 5.607541 1.297537 -1.222252
H 6.581206 -0.974002 -1.030035
H 5.122955 -2.978192 -1.142064
H 2.661866 -2.700475 -1.421079
C -4.301857 0.932175 0.131300
C -5.720097 -0.769325 -1.027850

C -4.336387 -1.424054 0.952381
H -5.168761 -1.180266 1.627248
H -4.409243 -2.482103 0.672891
H -3.395897 -1.273253 1.501789
H -6.565019 -0.539742 -0.364005
H -5.804067 -0.126659 -1.915835
H -5.799831 -1.813364 -1.351452
H -5.146877 1.175934 0.789092
H -3.382988 1.142454 0.698746
H -4.358072 1.617863 -0.727294

TS_{rc}^{A}

54
Energy: -6536.88223724
Cu -0.343191 -0.123348 -0.735998
Cl -0.281044 -0.933475 -2.711148
C -2.208534 -0.190036 -0.974083
O 1.420789 0.789437 -0.819724
S 2.452380 1.340924 0.127916
O 3.570595 0.423342 0.349372
C 3.128205 2.704878 -0.893881
O 1.898304 1.994899 1.312647
F 4.066041 3.340833 -0.208236
F 3.648633 2.233849 -2.013367
F 2.155268 3.559953 -1.190029
C -3.061317 -1.187362 -0.538909
C -4.441648 -0.997269 -0.611179
C -4.950520 0.181261 -1.140145
C -4.087226 1.169168 -1.604676
C -2.711012 0.978098 -1.534087
Br -2.413640 -2.806254 0.188660
H -5.102064 -1.783440 -0.250410
H -6.028134 0.321383 -1.199035
H -4.479136 2.088517 -2.035360
H -2.034050 1.742097 -1.916879
C 1.840240 -1.997264 0.986501
O 0.703349 -1.867505 0.499279
C 2.921124 -2.802116 0.298787
N 2.022893 -1.494895 2.226404
C 0.849038 -0.886844 2.827246
C -0.036194 -0.320925 1.804827
C -0.695011 0.493132 1.111454
C -1.514472 1.683041 1.310161
C -1.115379 2.930105 0.821926
C -1.963401 4.021747 0.963158
C -3.205468 3.878471 1.575387
C -3.598980 2.635462 2.062140
C -2.757339 1.538004 1.933219
H 2.951064 -1.260326 2.555682
H 0.313980 -1.629095 3.437905
H 1.158766 -0.051644 3.470632
H -0.137774 3.037702 0.355983
H -1.647924 4.993989 0.588947
H -3.866396 4.737656 1.675275
H -4.568004 2.516673 2.543760
H -3.064696 0.554449 2.287875
C 2.984468 -2.405846 -1.176085
C 4.287020 -2.638284 0.957586
C 2.457514 -4.262851 0.423855
H 3.191915 -4.914144 -0.068261
H 2.379365 -4.573261 1.475813
H 1.484069 -4.410273 -0.059793
H 5.018040 -3.251643 0.415315
H 4.627714 -1.596327 0.914459
H 4.288972 -2.989222 2.000489
H 3.704066 -3.059040 -1.687972
H 2.007860 -2.514520 -1.663435
H 3.311293 -1.365519 -1.289243

TS_{ar}^{B}

54
Energy: -6536.90468413
Cu -0.036759 -1.189387 0.365194
Cl -0.225011 -2.230307 2.257155
C 1.578569 -0.329058 0.926310
O -1.615243 0.517485 0.584972
S -1.791031 1.889658 0.036908
O -2.261743 1.919658 -1.358994
C -3.217865 2.512859 1.003907
O -0.697787 2.811131 0.363455

F -3.530360 3.738306 0.612254
F -4.271950 1.720794 0.815267
F -2.921811 2.528821 2.293450
C 2.772673 -1.053733 0.879024
C 3.877861 -0.633103 1.600849
C 3.778430 0.520592 2.377463
C 2.585359 1.237125 2.446727
C 1.480654 0.817991 1.718166
Br 2.898199 -2.613217 -0.178911
H 4.802786 -1.204220 1.559087
H 4.649571 0.859473 2.935282
H 2.517074 2.131668 3.061758
H 0.554996 1.393799 1.717999
C -2.242043 -1.591636 -1.170020
O -1.377670 -2.275432 -0.570929
C -3.711112 -1.819364 -0.892766
N -1.866839 -0.725995 -2.126430
C -0.461288 -0.577351 -2.416756
C 0.403603 -0.262502 -1.226627
C 1.426175 0.467861 -1.028966
C 2.469817 1.415139 -1.211747
C 2.208904 2.780600 -0.998893
C 3.217072 3.704483 -1.229293
C 4.474711 3.278596 -1.653255
C 4.738989 1.923495 -1.851944
C 3.742131 0.987322 -1.630397
H -2.424934 0.123538 -2.233666
H -0.084238 -1.517356 -2.843461
H -0.358272 0.215854 -3.165846
H 1.218845 3.080029 -0.651459
H 3.022675 4.763738 -1.075182
H 5.260860 4.010986 -1.829715
H 5.724261 1.601055 -2.181982
H 3.924843 -0.076338 -1.779523
C -4.608180 -0.781310 -1.559631
C -4.020270 -3.211512 -1.464822
C -3.930408 -1.823100 0.621453
H -5.654481 -1.007354 -1.316253
H -4.402102 0.236374 -1.201134
H -4.517295 -0.798954 -2.654914
H -4.969740 -2.109145 0.832659
H -3.259360 -2.533461 1.118596
H -3.746224 -0.830513 1.048868
H -5.075859 -3.450578 -1.278683
H -3.850377 -3.245848 -2.550513
H -3.400481 -3.979672 -0.987275

I_2

54
Energy: -6536.94697504
Cu 0.693216 0.321884 0.577182
Cl 1.579964 0.976247 2.472051
O 1.508277 -1.381588 0.521051
S 2.475404 -1.415533 -0.665244
O 2.772948 -0.052979 -1.120409
C 3.997333 -2.026900 0.158244
O 2.108965 -2.405227 -1.666750
F 4.951889 -2.133473 -0.753824
F 4.372795 -1.179987 1.093487
F 3.764870 -3.213196 0.692201
C 0.460035 2.434268 -1.127351
O 0.265312 2.041972 0.052821
C 0.912161 3.874365 -1.280477
N 0.321144 1.661593 -2.198111
C -0.059327 0.247869 -2.194294
C -0.438137 -0.210184 -0.843152
C -1.493723 -0.880285 -0.392604
C -2.470215 -1.562098 -1.266441
C -2.028463 -2.285261 -2.380505
C -2.943536 -2.932883 -3.203304
C -4.303724 -2.869874 -2.918061
C -4.747347 -2.168966 -1.798603
C -3.837579 -1.524686 -0.971071
H 0.578197 2.057136 -3.093360
H -0.934686 0.131910 -2.853693
H 0.773815 -0.331081 -2.618094
H -0.956955 -2.381839 -2.563350
H -2.589486 -3.503646 -4.060015
H -5.019821 -3.379614 -3.560347
H -5.810341 -2.126738 -1.567429
H -4.183183 -0.973987 -0.096480

C 0.984867 4.334610 -2.732273
C -0.061670 4.760674 -0.500049
C 2.308670 3.923307 -0.642433
H 2.713185 4.940676 -0.727848
H 2.267459 3.644994 0.417401
H 2.999283 3.231280 -1.145687
H 0.293027 5.799273 -0.526209
H -1.068516 4.732860 -0.940034
H -0.133706 4.441333 0.545233
H 1.282311 5.390304 -2.756609
H 1.743606 3.784876 -3.309253
H 0.013974 4.260798 -3.243285
C -1.635050 -0.986477 1.090354
C -1.188627 -2.141065 1.744458
C -1.256932 -2.252243 3.125041
C -1.788975 -1.209835 3.878835
C -2.262708 -0.064784 3.252562
C -2.188608 0.037268 1.868361
H -0.749415 -2.935792 1.143522
H -0.883522 -3.148591 3.614954
H -1.833132 -1.283472 4.963492
H -2.684456 0.754980 3.828710
Br -2.898155 1.603499 1.058058

TS_{rc}^{B}

54
Energy: -6536.92600039
Cu -0.752394 -1.352301 0.348318
Cl -0.862329 -3.497244 -0.180880
C 0.822188 1.484676 1.161258
O -2.149747 -0.287602 1.096905
S -3.100171 0.294820 0.065740
O -2.744658 -0.121257 -1.296232
C -4.637955 -0.628125 0.445421
O -3.381031 1.700921 0.306661
F -5.592790 -0.249495 -0.390587
F -4.421174 -1.924983 0.307298
F -5.019173 -0.375784 1.687468
C 2.143735 1.417680 1.608154
C 2.480517 1.679481 2.930078
C 1.477977 2.019832 3.830831
C 0.157887 2.114728 3.402957
C -0.167183 1.857913 2.076623
Br 3.553994 1.039827 0.386945
H 3.520469 1.620976 3.242742
H 1.736223 2.220095 4.868964
H -0.627675 2.391508 4.102615
H -1.199332 1.925188 1.734429
C 1.793556 -1.954296 -0.772981
O 1.330269 -1.329014 0.223720
C 2.851075 -3.023083 -0.605806
N 1.364285 -1.592931 -1.977286
C 0.272495 -0.627372 -2.036136
C 0.176469 -0.020697 -0.675638
C 0.449280 1.224025 -0.256063
C 0.257381 2.385739 -1.145105
C -0.730104 2.389993 -2.141417
C -0.868141 3.488565 -2.981691
C -0.036221 4.593283 -2.836407
C 0.927611 4.607082 -1.830381
C 1.067672 3.519436 -0.981555
H 1.575990 -2.164213 -2.785891
H 0.514516 0.175526 -2.749184
H -0.657896 -1.120764 -2.351978
H -1.440086 1.564566 -2.204440
H -1.650256 3.487345 -3.738441
H -0.150858 5.453950 -3.492999
H 1.572142 5.474882 -1.702250
H 1.821420 3.536426 -0.195999
C 2.912520 -3.432683 0.862774
C 2.498006 -4.235984 -1.472068
C 4.197250 -2.432725 -1.044555
H 3.206705 -5.044491 -1.251873
H 1.481861 -4.592310 -1.258587
H 2.585564 -4.026337 -2.548622
H 4.971561 -3.204762 -0.945076
H 4.181267 -2.101138 -2.091750
H 4.480674 -1.580068 -0.414616
H 3.686714 -4.200487 0.987868
H 3.164742 -2.578322 1.502988
H 1.951717 -3.845047 1.196108

Reaction 49

reactant

33
Energy: -673.153098765
C -4.058140 0.007907 0.185254
O -4.640120 -0.004525 -0.890334
C -4.826386 -0.098931 1.507049
N -2.702046 0.101671 0.245077
H -2.203941 0.131332 1.122610
C -1.933000 0.190144 -0.987062
C -0.506143 0.140715 -0.713158
H -2.236614 -0.631924 -1.652685
H -2.195413 1.115676 -1.523528
C 0.679887 0.099636 -0.470043
C 2.078127 0.043723 -0.188988
C 2.705474 -1.189283 0.039155
C 4.065012 -1.242686 0.313779
C 4.815560 -0.070836 0.363510
C 4.200951 1.158004 0.137375
C 2.841405 1.218610 -0.136610
H 2.110588 -2.099981 -0.003969
H 4.543148 -2.204873 0.489197
H 5.881907 -0.115348 0.578319
H 4.785526 2.075640 0.174760
H 2.352119 2.174570 -0.315095
C -3.950746 -0.008699 2.752729
C -5.855280 1.032345 1.521011
C -5.548880 -1.447690 1.485909
H -4.579426 -0.082806 3.650413
H -3.219739 -0.828901 2.806363
H -3.415406 0.950220 2.813431
H -6.188135 -1.548534 2.374304
H -6.174006 -1.533587 0.589728
H -4.833334 -2.282349 1.486159
H -6.503728 0.942654 2.403844
H -5.365812 2.015900 1.558607
H -6.477509 0.995508 0.619835

I_c

68
Energy: -6844.42904911
Cu -0.530375 0.850247 0.516649
Cl -1.084595 1.674709 2.467940
C 1.015600 1.944234 0.694804
O -2.183883 -0.287698 0.396489
S -2.594870 -1.153328 -0.768172
O -1.498892 -1.958135 -1.312766
C -3.665572 -2.371725 0.089308
O -3.478770 -0.482584 -1.713229
F -4.134496 -3.242985 -0.786735
F -2.959932 -3.037197 1.010768
F -4.673368 -1.763874 0.689818
C 2.207749 1.245290 0.690579
C 3.382882 2.000743 0.661222
C 3.360532 3.387299 0.643672
C 2.134101 4.044701 0.666772
C 0.942951 3.325925 0.704830
C 2.381139 -1.096775 -1.899552
O 3.250098 -0.234348 -1.942520
C 2.741479 -2.563101 -1.652939
N 1.061234 -0.796424 -2.081952
C 0.675255 0.549473 -2.392334
C -0.336839 1.164084 -1.506883
C -1.275235 1.956812 -1.330135
C -2.267719 2.926761 -1.058175
C -3.590547 2.557979 -0.752063
C -4.522210 3.547407 -0.481269
C -4.157260 4.891791 -0.526500
C -2.852525 5.263498 -0.847640
C -1.904756 4.287756 -1.107092
H 2.257787 0.160824 0.694969
Br 5.042748 1.098029 0.651051
H 4.294060 3.944256 0.624350
H 2.103217 5.133117 0.677550
H -0.014625 3.838408 0.774505
H 0.323382 -1.481548 -1.939115
H 1.596217 1.151344 -2.345441
H 0.280647 0.622193 -3.418142

H -3.858653 1.503434 -0.742074
H -5.544771 3.269235 -0.235970
H -4.898969 5.659789 -0.313684
H -2.577186 6.315074 -0.891552
H -0.880930 4.554555 -1.365278
C 1.561602 -3.521618 -1.774133
C 3.811790 -2.934847 -2.681362
C 3.339654 -2.642820 -0.246129
O 0.202436 -3.190747 1.317401
C -0.334058 -4.334239 1.998772
C -0.418490 -5.438526 0.978543
C -0.003495 -1.947595 1.732221
O 0.426317 -1.050604 1.019262
C -0.718095 -1.724046 3.025306
H 0.334514 -4.590131 2.833204
H -1.324579 -4.097426 2.404424
H -0.819527 -6.350931 1.434391
H -1.077884 -5.134919 0.156151
H 0.571457 -5.659745 0.562795
H -0.378671 -2.419740 3.800856
H -0.568147 -0.691347 3.347218
H -1.794966 -1.873460 2.874810
H 1.109178 -3.489459 -2.775742
H 1.914336 -4.550041 -1.609029
H 0.781199 -3.317948 -1.028933
H 3.686138 -3.666712 -0.043526
H 4.191465 -1.957381 -0.149089
H 2.590239 -2.380595 0.513206
H 4.168915 -3.957562 -2.494987
H 3.410579 -2.898638 -3.704220
H 4.661805 -2.245996 -2.622516

I_1

54
Energy: -6536.90749979
Cu -0.690249 0.111591 -0.169636
Cl -0.837902 1.239873 -1.972458
C 0.954832 -0.381259 -0.952245
O -2.585395 0.420831 0.494528
S -3.153360 -0.964870 0.272956
O -3.741750 -1.585116 1.444472
C -4.526621 -0.652072 -0.901975
O -2.151801 -1.742096 -0.489980
F -5.086907 -1.806257 -1.225022
F -5.424995 0.130758 -0.327829
F -4.062091 -0.067357 -1.990452
C 2.139407 0.231404 -0.595341
C 3.306636 -0.414479 -0.999702
C 3.278940 -1.589273 -1.744162
C 2.053936 -2.141453 -2.099770
C 0.860164 -1.529109 -1.718120
H 2.172682 1.144758 -0.000177
Br 4.975994 0.314151 -0.481166
H 4.211997 -2.059454 -2.045609
H 2.021084 -3.053269 -2.693615
H -0.104443 -1.940281 -2.010259
C 0.298848 3.043367 1.212657
O 1.410846 2.610535 1.494026
C 0.108298 4.313024 0.387831
N -0.818306 2.427289 1.705992
C -0.663980 1.260193 2.541877
C -0.217783 0.040122 1.833907
C 0.151566 -1.127554 1.694738
C 0.674234 -2.428314 1.487052
C -0.185425 -3.507292 1.221982
C 0.353546 -4.764136 0.993943
C 1.734134 -4.951817 1.035601
C 2.588856 -3.885290 1.309528
C 2.065073 -2.622135 1.534455
H -1.737579 2.622810 1.330183
H 0.079061 1.477395 3.319875
H -1.626842 1.045944 3.019165
H -1.258835 -3.332974 1.175266
H -0.305366 -5.603159 0.780984
H 2.149173 -5.941975 0.854103
H 3.665559 -4.039192 1.340913
H 2.715608 -1.771630 1.735087
C -1.322294 4.518980 -0.102588
C 0.504561 5.469419 1.314125
C 1.067534 4.255897 -0.800719
H 1.025467 5.202429 -1.356890

H 2.097190 4.096065 -0.460334
H 0.799110 3.442934 -1.489742
H 0.433497 6.421644 0.770507
H -0.160340 5.526712 2.187961
H 1.534773 5.346983 1.670388
H -1.365785 5.421984 -0.725919
H -1.671408 3.678628 -0.719886
H -2.026974 4.674416 0.727704

TS_{rc}^{A}

54
Energy: -6536.88554744
Cu -0.001781 0.209990 0.840458
Cl 0.131904 -0.434093 2.874160
C 1.849117 0.511145 0.939649
O -1.960452 0.339162 0.820831
S -3.060569 0.170486 -0.195492
O -3.457511 -1.226203 -0.383456
C -4.441193 0.965633 0.711496
O -2.889426 0.968278 -1.408488
F -5.531957 0.924648 -0.038774
F -4.669684 0.337275 1.850194
F -4.133719 2.232033 0.961229
C 2.740356 -0.429865 0.455939
C 4.089756 -0.087177 0.449286
C 4.537472 1.141735 0.918929
C 3.609807 2.051353 1.414552
C 2.251558 1.741019 1.434695
H 2.407395 -1.394114 0.079350
Br 5.346075 -1.333254 -0.234656
H 5.599107 1.376085 0.902180
H 3.947412 3.012850 1.797809
H 1.528760 2.454298 1.830135
C -0.813591 -2.470885 -1.027625
O 0.101927 -1.786695 -0.524519
C -1.342900 -3.717524 -0.356176
N -1.223816 -2.101826 -2.255161
C -0.556787 -0.933919 -2.807657
C -0.045549 -0.081309 -1.725449
C 0.123585 0.922354 -0.984704
C 0.281163 2.367802 -1.101147
C -0.696757 3.246432 -0.626013
C -0.478636 4.616772 -0.702956
C 0.706735 5.116185 -1.234916
C 1.683707 4.238418 -1.695798
C 1.476886 2.866643 -1.628203
H -2.142788 -2.368918 -2.586310
H 0.255198 -1.248726 -3.479204
H -1.281440 -0.332336 -3.373129
H -1.626475 2.852396 -0.221478
H -1.245285 5.299198 -0.340983
H 0.870929 6.190985 -1.286997
H 2.615530 4.621723 -2.107812
H 2.247038 2.169067 -1.957713
C -1.607555 -3.418859 1.120132
C -2.602325 -4.254372 -1.028652
C -0.210481 -4.749644 -0.483398
H -0.526869 -5.686070 -0.005500
H 0.022524 -4.967162 -1.535545
H 0.702469 -4.400766 0.014052
H -2.929918 -5.157111 -0.497593
H -3.421100 -3.526006 -0.983167
H -2.421383 -4.548040 -2.073457
H -1.912627 -4.347942 1.620102
H -0.711117 -3.035007 1.622528
H -2.409177 -2.679201 1.235001

TS_{ar}^{B}

54
Energy: -6536.89515215
Cu -0.122943 -0.049193 0.530505
Cl -0.269784 -0.380332 2.666886
C 1.797151 0.245367 0.638995
O -2.092236 -0.071825 0.324602
S -2.939169 1.029718 -0.268587
O -3.463550 0.665205 -1.588825
C -4.379269 0.963227 0.865379
O -2.359024 2.358408 -0.110590
F -5.284262 1.844906 0.471585
F -4.912518 -0.252211 0.835053

F -3.995699 1.242755 2.095620
C 2.578607 -0.752363 0.069502
C 3.885249 -0.895839 0.526650
C 4.406256 -0.059691 1.505821
C 3.600458 0.940928 2.045678
C 2.290346 1.102846 1.618060
H 2.182217 -1.411650 -0.699264
Br 4.978205 -2.244397 -0.228620
H 5.433771 -0.188605 1.837472
H 3.996607 1.593841 2.820849
H 1.658753 1.881331 2.040360
C -1.241224 -2.204959 -1.044804
O -0.140313 -2.122745 -0.480580
C -2.279023 -3.231041 -0.627335
N -1.510940 -1.405838 -2.109533
C -0.538723 -0.405023 -2.465815
C 0.040082 0.399998 -1.362283
C 0.833008 1.255030 -0.880999
C 1.388649 2.591547 -0.894506
C 0.510017 3.678313 -0.813701
C 1.021696 4.969266 -0.881496
C 2.388876 5.174055 -1.035333
C 3.260661 4.087863 -1.114497
C 2.767250 2.795393 -1.032516
H -2.481498 -1.138207 -2.260508
H 0.313370 -0.885509 -2.969841
H -1.007762 0.284509 -3.180286
H -0.556609 3.493021 -0.688417
H 0.344807 5.818373 -0.813355
H 2.782647 6.187481 -1.091037
H 4.329442 4.252220 -1.235913
H 3.437180 1.937377 -1.079404
C -3.651842 -2.978955 -1.245099
C -1.736212 -4.581831 -1.115490
C -2.381482 -3.237232 0.898390
H -4.351318 -3.744997 -0.885785
H -4.063721 -2.001156 -0.958033
H -3.632934 -3.049865 -2.342056
H -3.054229 -4.047473 1.210981
H -1.401445 -3.397882 1.361829
H -2.778782 -2.286672 1.275590
H -2.429896 -5.381422 -0.822399
H -1.636782 -4.602288 -2.210250
H -0.755911 -4.793659 -0.671211

I_2

54
Energy: -6536.94852935
Cu -0.624616 -0.473083 -0.021380
Cl -1.513109 -2.314168 0.784046
C 2.214773 -0.062775 0.098797
O -0.418602 0.388149 1.632029
S -1.383229 1.575282 1.723013
O -2.480082 1.420393 0.762969
C -2.101716 1.286197 3.387443
O -0.701243 2.859729 1.776808
F -2.966728 2.257307 3.639520
F -2.720225 0.124993 3.414130
F -1.141077 1.307199 4.293856
C 1.886997 -1.389338 -0.209964
C 2.407978 -2.423237 0.562220
C 3.251935 -2.163024 1.632156
C 3.592792 -0.842678 1.919026
C 3.086996 0.201568 1.158143
H 1.296586 -1.628263 -1.094902
Br 1.965343 -4.205726 0.131110
H 3.642733 -2.984639 2.227662
H 4.256029 -0.632071 2.755878
H 3.336590 1.233870 1.398380
C -1.903240 -0.076434 -2.354467
O -1.162297 -0.903632 -1.755396
C -3.112569 -0.634216 -3.079576
N -1.696517 1.234608 -2.321074
C -0.637376 1.919856 -1.580327
C 0.256181 0.991737 -0.850280
C 1.574045 1.044595 -0.644981
C 2.403986 2.194232 -1.073223
C 2.016423 3.500446 -0.755868
C 2.802357 4.577707 -1.152888
C 3.977569 4.360099 -1.864970
C 4.377568 3.060065 -2.167809

C 3.600514 1.981432 -1.766947
H -2.390235 1.826133 -2.760890
H -0.027328 2.488397 -2.301095
H -1.125251 2.636801 -0.903931
H 1.121434 3.659706 -0.152191
H 2.500901 5.590543 -0.891410
H 4.591430 5.204125 -2.174860
H 5.302115 2.886925 -2.715944
H 3.911602 0.961586 -1.993570
C -3.761389 0.366443 -4.030377
C -2.694737 -1.889603 -3.845928
C -4.093122 -1.010461 -1.955621
H -4.997746 -1.447738 -2.399245
H -3.646605 -1.739876 -1.267131
H -4.387314 -0.128043 -1.369978
H -3.583663 -2.343334 -4.302812
H -1.982557 -1.655069 -4.648837
H -2.235819 -2.624321 -3.176048
H -4.588731 -0.126648 -4.555682
H -4.202991 1.226022 -3.504002
H -3.059901 0.731509 -4.793907

TS_{rc}^{B}

54
Energy: -6536.92688618
Cu 0.574622 -0.174055 -0.858055
Cl -0.636005 -1.298814 -2.202176
C -2.534770 0.855555 -0.419268
O 2.276691 0.739976 -0.996769
S 3.445439 0.028846 -0.342252
O 3.113705 -1.366262 -0.041773
C 4.649757 -0.027521 -1.723046
O 4.068131 0.816691 0.717386
F 5.749783 -0.634209 -1.307651
F 4.131238 -0.697495 -2.735887
F 4.943730 1.202598 -2.108387
C -2.819554 -0.475239 -0.095466
C -3.932193 -1.087454 -0.649935
C -4.767803 -0.414890 -1.531394
C -4.482882 0.909472 -1.851691
C -3.385372 1.546943 -1.292502
H -2.169829 -1.031993 0.573786
Br -4.290037 -2.895157 -0.220678
H -5.625831 -0.924272 -1.963872
H -5.125015 1.444795 -2.548166
H -3.165071 2.581773 -1.548675
C 0.951593 -0.895406 1.890331
O 0.017791 -1.042559 1.065293
C 1.453676 -2.075496 2.698961
N 1.420840 0.334078 2.123424
C 0.864578 1.478007 1.412049
C -0.146366 1.034747 0.394529
C -1.373735 1.538436 0.164949
C -1.518775 2.984401 0.500963
C -0.580394 3.920772 0.056436
C -0.715530 5.263560 0.392702
C -1.787263 5.680653 1.174875
C -2.729957 4.753618 1.614784
C -2.603482 3.412767 1.276317
H 2.329683 0.450675 2.558968
H 0.367242 2.145763 2.131712
H 1.696280 2.014645 0.937264
H 0.243315 3.590386 -0.577769
H 0.015027 5.985832 0.033495
H -1.894786 6.731732 1.436491
H -3.572403 5.078105 2.223004
H -3.342151 2.686020 1.611799
C 2.860263 -1.842322 3.245013
C 0.456720 -2.220201 3.859646
C 1.422388 -3.331650 1.829979
H 1.718493 -4.195049 2.440214
H 0.418341 -3.515903 1.431502
H 2.116174 -3.242080 0.986779
H 0.752835 -3.076015 4.480498
H 0.443820 -1.325053 4.496991
H -0.560018 -2.402045 3.488766
H 3.188710 -2.746438 3.773352
H 3.577050 -1.643972 2.437380
H 2.895884 -1.022391 3.977710

Reaction 50

reactant

33
Energy: -673.153098765
C -4.058140 0.007907 0.185254
O -4.640120 -0.004525 -0.890334
C -4.826386 -0.098931 1.507049
N -2.702046 0.101671 0.245077
H -2.203941 0.131332 1.122610
C -1.933000 0.190144 -0.987062
C -0.506143 0.140715 -0.713158
H -2.236614 -0.631924 -1.652685
H -2.195413 1.115676 -1.523528
C 0.679887 0.099636 -0.470043
C 2.078127 0.043723 -0.188988
C 2.705474 -1.189283 0.039155
C 4.065012 -1.242686 0.313779
C 4.815560 -0.070836 0.363510
C 4.200951 1.158004 0.137375
C 2.841405 1.218610 -0.136610
H 2.110588 -2.099981 -0.003969
H 4.543148 -2.204873 0.489197
H 5.881907 -0.115348 0.578319
H 4.785526 2.075640 0.174760
H 2.352119 2.174570 -0.315095
C -3.950746 -0.008699 2.752729
C -5.855280 1.032345 1.521011
C -5.548880 -1.447690 1.485909
H -4.579426 -0.082806 3.650413
H -3.219739 -0.828901 2.806363
H -3.415406 0.950220 2.813431
H -6.188135 -1.548534 2.374304
H -6.174006 -1.533587 0.589728
H -4.833334 -2.282349 1.486159
H -6.503728 0.942654 2.403844
H -5.365812 2.015900 1.558607
H -6.477509 0.995508 0.619835

I_c

68
Energy: -6844.42906821
Cu 0.172172 0.754527 0.515456
Cl 0.484257 1.794086 2.409900
C 1.984250 0.210741 0.704808
O -1.730709 1.380510 0.383074
S -2.716181 1.214179 -0.744999
O -2.782179 -0.150024 -1.274077
C -4.288218 1.413530 0.178696
O -2.699586 2.310263 -1.705678
F -5.311721 1.256797 -0.642596
F -4.372015 0.487835 1.141395
F -4.349996 2.608515 0.738172
C 2.165547 -1.156321 0.615476
C 3.472790 -1.643568 0.535351
C 4.539093 -0.759039 0.578171
C 4.336113 0.612282 0.697285
C 3.037052 1.106977 0.766548
C -0.007815 -2.944607 -1.650301
O 1.180397 -3.225085 -1.530178
C -1.081542 -4.023318 -1.498076
N -0.422711 -1.689693 -1.991697
C 0.542672 -0.673293 -2.300144
C 0.471827 0.580491 -1.519558
C 0.598223 1.811294 -1.429422
C 0.862238 3.191437 -1.257578
C -0.170640 4.109576 -0.997825
C 0.142950 5.448246 -0.824297
C 1.464493 5.880719 -0.918204
C 2.490321 4.976364 -1.189563
C 2.196986 3.632569 -1.353335
H 1.339785 -1.860264 0.558460
H 3.635754 -2.712434 0.416048
Br 6.308853 -1.426051 0.474430
H 5.185139 1.290472 0.751543
H 2.865166 2.174524 0.890302
H -1.398139 -1.409953 -1.917568
H 1.532898 -1.127743 -2.142925
H 0.476051 -0.380860 -3.360085
H -1.196970 3.751852 -0.946252
H -0.650296 6.162872 -0.616296

H 1.698441 6.935163 -0.781476
H 3.518710 5.322180 -1.269818
H 2.981684 2.908088 -1.566451
C -2.509316 -3.507607 -1.629821
C -0.804600 -5.049179 -2.601938
C -0.890184 -4.679302 -0.130714
O -2.663949 -2.123704 1.444287
C -3.880609 -2.336823 2.176817
C -4.856292 -2.967998 1.220090
C -1.731113 -1.266952 1.842931
O -0.749296 -1.141669 1.123857
C -1.935184 -0.524966 3.123803
H -3.663648 -2.989650 3.034569
H -4.264070 -1.380860 2.552078
H -5.807673 -3.169613 1.725460
H -5.042115 -2.291894 0.376257
H -4.460713 -3.912881 0.828142
H -2.336916 -1.171069 3.911981
H -0.989742 -0.080853 3.440663
H -2.647166 0.293517 2.949621
H -2.698404 -3.054619 -2.613482
H -3.208082 -4.350149 -1.524014
H -2.749472 -2.773731 -0.849819
H -1.585843 -5.523994 -0.020338
H 0.134601 -5.052859 -0.020460
H -1.091991 -3.963449 0.677168
H -1.507695 -5.889579 -2.515113
H -0.933323 -4.603024 -3.598589
H 0.218581 -5.435335 -2.527554

I_1

54
Energy: -6536.90832544
Cu -0.782207 0.002809 -0.116146
Cl -1.198464 0.869392 -2.017853
C 0.971005 0.454105 -0.646361
O -2.647145 -0.699494 0.277195
S -2.400218 -2.183095 0.103836
O -2.755091 -3.003788 1.245863
C -3.563130 -2.624799 -1.243412
O -1.048126 -2.348929 -0.472587
F -3.419317 -3.907686 -1.532896
F -4.803689 -2.397829 -0.844130
F -3.304145 -1.898423 -2.314886
C 1.587790 1.618122 -0.230007
C 2.972856 1.695683 -0.375466
C 3.665689 0.632927 -0.946037
C 3.011378 -0.503413 -1.404448
C 1.626264 -0.590863 -1.272909
H 1.029883 2.434222 0.231574
H 3.507820 2.580244 -0.036258
Br 5.552296 0.729905 -1.087390
H 3.571775 -1.315758 -1.862051
H 1.088564 -1.463376 -1.641051
C -1.674244 3.005440 1.156820
O -0.551724 3.232653 1.595284
C -2.389383 3.974139 0.218982
N -2.357808 1.894643 1.566363
C -1.734031 0.985052 2.498374
C -0.635711 0.169032 1.935569
C 0.290376 -0.644506 1.931034
C 1.431746 -1.482446 1.874912
C 1.305091 -2.845180 1.558496
C 2.445586 -3.628079 1.462721
C 3.701903 -3.066366 1.684860
C 3.829996 -1.717748 2.014330
C 2.698830 -0.923440 2.112166
H -3.180208 1.571667 1.072514
H -1.319106 1.563908 3.333344
H -2.501957 0.305448 2.885035
H 0.315696 -3.256258 1.367374
H 2.356154 -4.682429 1.210024
H 4.592419 -3.687185 1.601332
H 4.814007 -1.285547 2.183847
H 2.775263 0.136291 2.350011
C -3.612254 3.373862 -0.469675
C -2.822190 5.155320 1.096451
C -1.382525 4.453026 -0.825768
H -1.844957 5.217527 -1.464894
H -0.500608 4.889066 -0.342443
H -1.053049 3.624569 -1.468114

H -3.307962 5.920867 0.475752
H -3.538727 4.838181 1.867671
H -1.955971 5.608495 1.594315
H -4.035386 4.110840 -1.165109
H -3.357600 2.479113 -1.056081
H -4.409573 3.122011 0.245057

TS_{rc}^{A}

54
Energy: -6536.88501549
Cu 0.080645 -0.123335 -0.720519
Cl -0.020342 -0.895557 -2.710748
C -1.774897 -0.395926 -0.652634
O 1.913047 0.563392 -0.892318
S 3.082998 0.833241 0.017772
O 3.930290 -0.343076 0.221180
C 4.054965 1.965439 -1.047459
O 2.754878 1.613943 1.209553
F 5.147247 2.337326 -0.397274
F 4.398390 1.361445 -2.170469
F 3.330880 3.040964 -1.329447
C -2.306871 -1.539753 -0.083684
C -3.690393 -1.638506 0.052800
C -4.496306 -0.599098 -0.396172
C -3.954022 0.534509 -0.988076
C -2.571316 0.634761 -1.124745
H -1.661734 -2.343378 0.265873
H -4.138176 -2.519722 0.507443
Br -6.377295 -0.734065 -0.201059
H -4.602661 1.331644 -1.344528
H -2.137280 1.519561 -1.591016
C 1.842749 -2.303189 1.148656
O 0.715231 -1.974996 0.723114
C 2.683094 -3.356430 0.463050
N 2.221651 -1.747717 2.314843
C 1.264534 -0.819940 2.896289
C 0.422265 -0.237505 1.842377
C -0.106382 0.619425 1.085935
C -0.690533 1.953440 1.177308
C -0.070498 3.060224 0.590502
C -0.693372 4.301238 0.646708
C -1.930751 4.444714 1.268008
C -2.551314 3.338395 1.840898
C -1.937181 2.093417 1.795980
H 3.204619 -1.682788 2.550639
H 0.654237 -1.336341 3.651421
H 1.802816 0.010622 3.372708
H 0.901724 2.945863 0.116444
H -0.204197 5.163420 0.197431
H -2.413940 5.419476 1.302369
H -3.522029 3.441554 2.322462
H -2.427509 1.214012 2.213811
C 2.687862 -3.089116 -1.042538
C 4.108953 -3.411290 1.003191
C 1.969783 -4.687088 0.753048
H 2.524635 -5.501645 0.269303
H 1.928759 -4.897739 1.831281
H 0.947507 -4.684259 0.355698
H 4.659859 -4.194988 0.467736
H 4.634530 -2.461984 0.840574
H 4.137307 -3.676476 2.070730
H 3.242848 -3.893132 -1.544106
H 1.670571 -3.063614 -1.452130
H 3.172605 -2.132509 -1.271724

TS_{ar}^{B}

54
Energy: -6536.89570571
Cu -0.254301 -0.270628 0.271736
Cl -0.115042 -0.899046 2.340166
C 1.676965 -0.259465 0.079208
O -2.206054 -0.011151 0.416381
S -2.953865 1.274828 0.151518
O -3.750142 1.197218 -1.077352
C -4.162653 1.215147 1.529158
O -2.148951 2.471408 0.367796
F -4.971460 2.259286 1.446729
F -4.879031 0.100930 1.441951
F -3.525790 1.237586 2.683659
C 2.209397 -1.251879 -0.734494

C 3.535963 -1.635882 -0.554570
C 4.300875 -1.002087 0.416614
C 3.768006 0.003882 1.220014
C 2.443485 0.376956 1.052083
H 1.595851 -1.733642 -1.493133
H 3.972098 -2.417236 -1.172584
Br 6.108798 -1.503747 0.643654
H 4.382433 0.480773 1.979902
H 2.013215 1.159219 1.674300
C -1.936182 -1.997983 -1.344816
O -0.757581 -2.155393 -0.995062
C -3.039746 -2.929646 -0.876517
N -2.250188 -1.010324 -2.223709
C -1.204867 -0.102424 -2.620139
C -0.326457 0.430744 -1.549484
C 0.656739 1.093021 -1.120687
C 1.417038 2.322186 -1.062317
C 0.760944 3.493799 -0.667177
C 1.464819 4.692821 -0.655389
C 2.801724 4.724462 -1.038500
C 3.451820 3.553930 -1.429922
C 2.767448 2.348886 -1.432162
H -3.176348 -0.593967 -2.148008
H -0.537760 -0.603506 -3.337492
H -1.672584 0.748668 -3.132753
H -0.285387 3.442259 -0.367049
H 0.962501 5.606375 -0.344033
H 3.346180 5.667126 -1.029339
H 4.497838 3.582636 -1.728219
H 3.265151 1.423553 -1.720720
C -4.439874 -2.403885 -1.181930
C -2.809879 -4.245399 -1.633937
C -2.882307 -3.165835 0.626098
H -5.180443 -3.119822 -0.802103
H -4.638969 -1.439623 -0.693378
H -4.617967 -2.299812 -2.261812
H -3.612220 -3.919862 0.950927
H -1.876029 -3.525965 0.868675
H -3.054229 -2.243912 1.195306
H -3.562563 -4.982398 -1.322867
H -2.901149 -4.104153 -2.720484
H -1.815160 -4.653521 -1.416494

I_2

54
Energy: -6536.94888167
Cu -0.688349 -0.606330 -0.540986
Cl -0.558416 -2.703693 -1.205499
C 1.725758 1.017032 -0.391430
O -0.132556 -0.901001 1.225174
S -1.317464 -0.679553 2.172725
O -2.581679 -0.758886 1.433537
C -1.216892 -2.200998 3.195901
O -1.123855 0.445873 3.073910
F -2.187409 -2.156379 4.096316
F -1.367681 -3.264766 2.436304
F -0.048567 -2.245352 3.809654
C 1.740944 0.086189 -1.438036
C 2.828595 -0.760254 -1.626217
C 3.918013 -0.654692 -0.774318
C 3.940034 0.284493 0.254366
C 2.846494 1.115391 0.440416
H 0.929807 0.066450 -2.167961
H 2.823308 -1.493585 -2.428898
Br 5.411307 -1.788349 -1.023556
H 4.806623 0.351306 0.908068
H 2.845017 1.834518 1.258346
C -2.858210 0.352419 -1.787810
O -1.725200 -0.150548 -2.025793
C -4.047178 -0.229893 -2.526254
N -3.046634 1.303430 -0.880862
C -2.025982 1.856171 0.007446
C -0.684131 1.260672 -0.191781
C 0.522150 1.831126 -0.123720
C 0.698791 3.249378 0.272342
C 0.101079 3.735700 1.439628
C 0.277871 5.063788 1.814854
C 1.051106 5.913668 1.030476
C 1.661902 5.430741 -0.125077
C 1.494631 4.103786 -0.498727
H -4.001202 1.583938 -0.693932

H -1.951055 2.938521 -0.186165
H -2.385276 1.720183 1.038166
H -0.464444 3.051464 2.074003
H -0.180177 5.430943 2.731591
H 1.188810 6.952648 1.324997
H 2.274849 6.091670 -0.735543
H 1.976756 3.717888 -1.396904
C -5.279081 0.669258 -2.490044
C -3.647769 -0.515718 -3.973478
C -4.333027 -1.552999 -1.793300
H -5.181586 -2.054751 -2.277608
H -3.461421 -2.220313 -1.825972
H -4.586579 -1.380068 -0.737867
H -4.481871 -1.014113 -4.483930
H -3.418489 0.408983 -4.520406
H -2.772508 -1.172669 -4.016583
H -6.070525 0.213425 -3.097844
H -5.697324 0.777373 -1.477961
H -5.078745 1.667029 -2.905126

TS_{rc}^{B}

54
Energy: -6536.92222767
Cu 1.385916 -0.057000 -1.040354
Cl 3.195722 -0.846939 -2.006262
C -1.636749 -0.232768 0.599700
O 0.558943 1.651274 -1.154820
S 1.128800 2.673583 -0.179415
O 2.179355 2.092253 0.659244
C 2.008439 3.789227 -1.338015
O 0.092680 3.469653 0.459209
F 2.595175 4.759130 -0.653827
F 2.926738 3.101259 -1.997681
F 1.152317 4.315320 -2.197860
C -1.736968 -0.349859 -0.795116
C -2.966410 -0.367057 -1.428514
C -4.125373 -0.264159 -0.665566
C -4.061894 -0.138878 0.716492
C -2.823070 -0.122104 1.339800
H -0.834852 -0.404750 -1.396599
H -3.029099 -0.447070 -2.510898
Br -5.809418 -0.287891 -1.523143
H -4.976286 -0.052902 1.298582
H -2.776340 -0.026856 2.422763
C 1.714598 -2.769991 -0.058027
O 0.764159 -2.078766 -0.513130
C 1.966681 -4.165355 -0.582336
N 2.412668 -2.281332 0.966920
C 2.158413 -0.897042 1.347347
C 0.845230 -0.517327 0.749384
C -0.349910 -0.257929 1.297527
C -0.313567 0.095302 2.749389
C -0.387133 1.429031 3.155892
C -0.378603 1.732155 4.513859
C -0.297142 0.716749 5.462445
C -0.222276 -0.613314 5.056494
C -0.227377 -0.924541 3.701371
H 3.291526 -2.711548 1.225862
H 2.085762 -0.810136 2.439418
H 2.977427 -0.249440 1.003377
H -0.438733 2.220234 2.405766
H -0.428988 2.772222 4.830212
H -0.289837 0.962025 6.523063
H -0.165058 -1.410087 5.796221
H -0.175790 -1.963314 3.369718
C 1.642670 -4.199824 -2.075225
C 3.415991 -4.589642 -0.355290
C 1.011949 -5.089285 0.187253
H 3.579253 -5.569514 -0.820854
H 4.116861 -3.879502 -0.815365
H 3.660769 -4.708257 0.710924
H 1.134393 -6.117775 -0.176921
H 1.221905 -5.081354 1.265869
H -0.033489 -4.793373 0.032683
H 1.813983 -5.215237 -2.455393
H 0.596277 -3.932901 -2.261141
H 2.277465 -3.498342 -2.631300

Reaction 51

reactant

33
Energy: -673.153098765
C -4.058140 0.007907 0.185254
O -4.640120 -0.004525 -0.890334
C -4.826386 -0.098931 1.507049
N -2.702046 0.101671 0.245077
H -2.203941 0.131332 1.122610
C -1.933000 0.190144 -0.987062
C -0.506143 0.140715 -0.713158
H -2.236614 -0.631924 -1.652685
H -2.195413 1.115676 -1.523528
C 0.679887 0.099636 -0.470043
C 2.078127 0.043723 -0.188988
C 2.705474 -1.189283 0.039155
C 4.065012 -1.242686 0.313779
C 4.815560 -0.070836 0.363510
C 4.200951 1.158004 0.137375
C 2.841405 1.218610 -0.136610
H 2.110588 -2.099981 -0.003969
H 4.543148 -2.204873 0.489197
H 5.881907 -0.115348 0.578319
H 4.785526 2.075640 0.174760
H 2.352119 2.174570 -0.315095
C -3.950746 -0.008699 2.752729
C -5.855280 1.032345 1.521011
C -5.548880 -1.447690 1.485909
H -4.579426 -0.082806 3.650413
H -3.219739 -0.828901 2.806363
H -3.415406 0.950220 2.813431
H -6.188135 -1.548534 2.374304
H -6.174006 -1.533587 0.589728
H -4.833334 -2.282349 1.486159
H -6.503728 0.942654 2.403844
H -5.365812 2.015900 1.558607
H -6.477509 0.995508 0.619835

I_c

73
Energy: -4426.33789150
Cu 0.446979 0.685479 0.560177
Cl 0.849169 1.679723 2.461824
C 2.177465 -0.066840 0.792821
O -1.369875 1.528037 0.385952
S -2.337430 1.430024 -0.765116
O -2.522493 0.063713 -1.260901
C -3.913466 1.805903 0.096161
O -2.186358 2.492056 -1.751950
F -4.908743 1.778436 -0.772705
F -4.151327 0.887891 1.040676
F -3.860331 2.994825 0.669224
C 2.190829 -1.446797 0.728123
C 3.434014 -2.079986 0.663738
C 4.617422 -1.345033 0.691842
C 4.552797 0.049696 0.791086
C 3.329934 0.701859 0.848344
C -0.049591 -2.992448 -1.633161
O 1.095678 -3.411013 -1.496412
C -1.246420 -3.936778 -1.508009
N -0.310236 -1.694208 -1.962115
C 0.769725 -0.788909 -2.233765
C 0.792116 0.471148 -1.460615
C 1.038890 1.684667 -1.382166
C 1.438010 3.032814 -1.225497
C 0.496669 4.061242 -1.041285
C 0.942010 5.364969 -0.890644
C 2.304954 5.653630 -0.932652
C 3.241047 4.639016 -1.128042
C 2.815164 3.328749 -1.268808
H 1.284820 -2.043865 0.676584
H 3.442828 -3.164206 0.562124
C 5.966024 -1.980308 0.616912
H 5.485909 0.609037 0.833854
H 3.280982 1.783439 0.958564
H -1.249857 -1.308269 -1.908749
H 1.699028 -1.342807 -2.030986
H 0.781091 -0.501010 -3.297024
H -0.562961 3.813058 -1.030175
H 0.220429 6.165262 -0.742108
H 2.642537 6.681969 -0.815029
H 4.302800 4.872668 -1.167431

H 3.528391 2.520454 -1.423889
C -2.598992 -3.255334 -1.680726
C -1.066339 -4.996682 -2.599334
C -1.166539 -4.602017 -0.133982
O -2.695653 -1.911042 1.454130
C -3.948441 -1.996462 2.148843
C -4.952787 -2.528465 1.161696
C -1.713380 -1.106635 1.842664
O -0.711529 -1.080834 1.141443
C -1.887935 -0.302391 3.090281
H -3.824082 -2.663892 3.013710
H -4.242857 -1.005644 2.512047
H -5.936374 -2.628745 1.634628
H -5.038311 -1.840511 0.311285
H -4.643612 -3.510620 0.784028
H -2.360075 -0.883322 3.889971
H -0.917082 0.075436 3.417226
H -2.523704 0.565229 2.868045
H -2.710367 -2.807249 -2.678492
H -3.396947 -4.004218 -1.573379
H -2.767433 -2.475128 -0.926719
H -1.962699 -5.354355 -0.035042
H -0.197771 -5.097840 -0.000629
H -1.295538 -3.861555 0.665915
H -1.865331 -5.747898 -2.525087
H -1.117885 -4.547207 -3.601385
H -0.097802 -5.499839 -2.497615
O 6.973558 -1.297583 0.628646
C 6.043975 -3.482405 0.523490
H 5.571902 -3.957737 1.393620
H 5.521174 -3.851288 -0.369024
H 7.095105 -3.778695 0.475788

I_1

59
Energy: -4118.81563277
Cu -0.559415 0.006049 -0.138807
Cl -1.072008 0.826737 -2.038640
C 1.147411 0.540512 -0.739666
O -2.378218 -0.764174 0.331800
S -2.083642 -2.240222 0.165951
O -2.371020 -3.058328 1.328917
C -3.276304 -2.737602 -1.135109
O -0.747427 -2.365160 -0.454182
F -3.108685 -4.021708 -1.405233
F -4.509274 -2.534112 -0.700889
F -3.070049 -2.025606 -2.227475
C 1.699365 1.758775 -0.393237
C 3.071321 1.907812 -0.587944
C 3.840496 0.866528 -1.117698
C 3.219108 -0.328245 -1.491893
C 1.848777 -0.496637 -1.332863
H 1.102177 2.557568 0.047651
H 3.535202 2.851051 -0.302006
C 5.321192 0.958976 -1.274609
H 3.832478 -1.123907 -1.911512
H 1.353771 -1.415331 -1.644710
C -1.563858 2.967854 1.141739
O -0.439729 3.286934 1.513414
C -2.400084 3.860503 0.228251
N -2.141246 1.818637 1.604994
C -1.407138 0.971311 2.514010
C -0.317020 0.181568 1.896504
C 0.633457 -0.604833 1.874033
C 1.784311 -1.420833 1.761950
C 1.661466 -2.796204 1.501610
C 2.806344 -3.557621 1.323002
C 4.063918 -2.961651 1.404577
C 4.190299 -1.600464 1.679013
C 3.054859 -0.828405 1.861069
H -2.964800 1.430218 1.163010
H -0.959900 1.599064 3.294934
H -2.111031 0.272185 2.980310
H 0.669218 -3.234072 1.415286
H 2.719292 -4.620867 1.110334
H 4.957355 -3.563822 1.249085
H 5.176192 -1.142538 1.729200
H 3.126034 0.241204 2.052375
C -3.599891 3.150530 -0.394010
C -2.887368 5.015547 1.111785
C -1.486729 4.404350 -0.869210

H -2.040015 5.119597 -1.492977
H -0.621433 4.917062 -0.433426
H -1.121460 3.596217 -1.518182
H -3.463908 5.728944 0.506800
H -3.537691 4.652509 1.920500
H -2.039003 5.546133 1.561740
H -4.111899 3.837393 -1.080974
H -3.300430 2.267124 -0.976717
H -4.341756 2.849742 0.360444
O 5.973578 -0.022403 -1.580105
C 5.986661 2.290502 -1.034948
H 5.555025 3.072581 -1.673286
H 5.857028 2.615087 0.006477
H 7.054754 2.194235 -1.246761

TS_{rc}^{A}

59
Energy: -4118.79275132
Cu -0.153723 -0.139099 -0.730722
Cl -0.226099 -0.904787 -2.725140
C -2.001723 -0.459390 -0.689061
O 1.659081 0.598607 -0.883970
S 2.815845 0.886616 0.037442
O 3.691291 -0.270489 0.232316
C 3.764069 2.056875 -1.007869
O 2.460080 1.643684 1.236507
F 4.844910 2.446003 -0.348726
F 4.125743 1.477336 -2.137988
F 3.014686 3.118100 -1.277177
C -2.509501 -1.634705 -0.163372
C -3.891270 -1.762484 -0.050613
C -4.741254 -0.733477 -0.466612
C -4.190164 0.430297 -1.011081
C -2.814922 0.572712 -1.135197
H -1.846184 -2.432457 0.164361
H -4.299613 -2.680139 0.371394
C -6.225852 -0.820438 -0.361544
H -4.866361 1.217693 -1.339160
H -2.392220 1.481169 -1.565195
C 1.646903 -2.296512 1.126414
O 0.514033 -1.994754 0.696316
C 2.519491 -3.321332 0.437882
N 2.003669 -1.741666 2.300242
C 1.016785 -0.848231 2.885188
C 0.167935 -0.273706 1.832579
C -0.377104 0.578055 1.082190
C -0.996609 1.895196 1.187160
C -0.402318 3.026581 0.621147
C -1.061151 4.248507 0.687820
C -2.307891 4.348422 1.298747
C -2.901930 3.217779 1.851774
C -2.251787 1.991777 1.796618
H 2.982989 -1.650462 2.542449
H 0.414244 -1.391497 3.627643
H 1.527915 -0.010361 3.378286
H 0.577013 2.945975 0.154542
H -0.593165 5.130183 0.254108
H -2.819738 5.308124 1.339570
H -3.880359 3.286985 2.323528
H -2.721090 1.092763 2.196618
C 2.535373 -3.036358 -1.064300
C 3.939890 -3.349680 0.993760
C 1.834472 -4.671672 0.704072
H 2.414442 -5.467335 0.218342
H 1.785296 -4.895530 1.779322
H 0.817363 -4.688233 0.294011
H 4.515246 -4.113756 0.455692
H 4.444817 -2.386732 0.847805
H 3.962405 -3.626070 2.058537
H 3.112100 -3.823116 -1.568755
H 1.522420 -3.026385 -1.485197
H 3.003722 -2.067831 -1.276487
O -6.930388 0.115206 -0.692892
C -6.833961 -2.097154 0.164498
H -6.546106 -2.957527 -0.454153
H -6.496011 -2.305692 1.188505
H -7.922425 -1.996040 0.159383

TS_{ar}^{B}

59

Energy: -4118.80269823
Cu -0.045057 -0.311576 0.308054
Cl 0.033346 -0.931712 2.380857
C 1.891094 -0.392966 0.156852
O -1.985336 0.049213 0.411755
S -2.664506 1.359247 0.086105
O -3.469263 1.264638 -1.136265
C -3.869270 1.440596 1.466410
O -1.793121 2.518670 0.239200
F -4.601052 2.536062 1.340676
F -4.663530 0.378424 1.426397
F -3.227493 1.466366 2.618298
C 2.382189 -1.447464 -0.602618
C 3.678532 -1.894394 -0.362604
C 4.477900 -1.272061 0.597836
C 3.961171 -0.196722 1.331928
C 2.668301 0.246260 1.121556
H 1.758314 -1.922895 -1.356376
H 4.061035 -2.730195 -0.946436
C 5.882627 -1.698760 0.877411
H 4.597432 0.270117 2.081126
H 2.263802 1.076624 1.697086
C -1.773450 -1.998832 -1.301546
O -0.609181 -2.198326 -0.925441
C -2.923528 -2.872438 -0.833368
N -2.027797 -1.017667 -2.205915
C -0.935148 -0.167787 -2.603553
C -0.048043 0.343841 -1.530160
C 0.964458 0.960972 -1.100913
C 1.782895 2.154330 -1.073444
C 1.173634 3.374670 -0.758387
C 1.936068 4.537216 -0.780793
C 3.284490 4.484992 -1.118566
C 3.887319 3.266420 -1.430780
C 3.143722 2.097565 -1.398265
H -2.936385 -0.560125 -2.158451
H -0.283044 -0.713219 -3.302029
H -1.354436 0.694956 -3.138385
H 0.117283 3.389280 -0.490611
H 1.470614 5.488378 -0.530842
H 3.875131 5.399294 -1.135058
H 4.942751 3.229600 -1.692641
H 3.603940 1.136172 -1.624233
C -4.292516 -2.314521 -1.211457
C -2.713278 -4.227997 -1.522692
C -2.827590 -3.044308 0.683337
H -5.071207 -2.986125 -0.827065
H -4.471791 -1.323261 -0.771876
H -4.429139 -2.251038 -2.300427
H -3.580294 -3.774088 1.011560
H -1.836358 -3.404155 0.982225
H -3.009695 -2.095948 1.203553
H -3.503436 -4.923483 -1.209159
H -2.759072 -4.133012 -2.617138
H -1.742866 -4.659844 -1.249181
O 6.567140 -1.082285 1.671312
C 6.422131 -2.906198 0.154520
H 5.806446 -3.793895 0.351273
H 6.427542 -2.748327 -0.932439
H 7.444386 -3.091711 0.494043

I_2

59
Energy: -4118.85712236
Cu -0.604461 -0.557165 -0.550886
Cl -0.800740 -2.606653 -1.342788
C 1.975233 0.754898 -0.565417
O 0.101098 -1.041417 1.116949
S -0.934903 -0.760214 2.212136
O -2.273956 -0.642028 1.626321
C -0.898925 -2.356691 3.118557
O -0.507584 0.266095 3.149975
F -1.736599 -2.270320 4.141079
F -1.273654 -3.334561 2.321369
F 0.322289 -2.582201 3.567021
C 1.753231 -0.094539 -1.656759
C 2.676395 -1.082715 -1.980574
C 3.850674 -1.216314 -1.242491
C 4.094117 -0.332964 -0.182554
C 3.170733 0.639189 0.154965
H 0.890771 0.061693 -2.306810

H 2.460447 -1.747429 -2.814998
C 4.874897 -2.264164 -1.534348
H 5.021229 -0.450107 0.375190
H 3.348306 1.298320 1.003674
C -2.746366 0.757263 -1.522515
O -1.718933 0.122550 -1.888108
C -4.048453 0.426248 -2.226131
N -2.733603 1.641414 -0.532513
C -1.578513 1.988880 0.294003
C -0.343452 1.268622 -0.090642
C 0.922491 1.694869 -0.126999
C 1.318079 3.052436 0.317054
C 0.895387 3.546792 1.555394
C 1.279671 4.818457 1.968630
C 2.087229 5.603618 1.152083
C 2.524754 5.110212 -0.075396
C 2.149950 3.838547 -0.487929
H -3.621288 2.038287 -0.251288
H -1.392084 3.069631 0.184097
H -1.854787 1.795679 1.341038
H 0.303725 2.907012 2.211705
H 0.957663 5.190245 2.939760
H 2.387727 6.598215 1.477099
H 3.165260 5.718666 -0.711521
H 2.496395 3.443444 -1.442917
C -5.163221 1.425626 -1.934709
C -3.790899 0.355902 -3.731865
C -4.432524 -0.964888 -1.695101
H -5.364309 -1.289460 -2.177487
H -3.646700 -1.701370 -1.907389
H -4.594297 -0.946578 -0.608084
H -4.709902 0.034340 -4.238643
H -3.503762 1.335000 -4.139190
H -2.998566 -0.364153 -3.961783
H -6.050435 1.149495 -2.517938
H -5.475188 1.415047 -0.879508
H -4.890005 2.451149 -2.221435
O 5.922493 -2.291742 -0.916371
C 4.569065 -3.281600 -2.602425
H 3.639036 -3.821124 -2.379220
H 4.437493 -2.800261 -3.580905
H 5.398309 -3.991359 -2.661286

TS_{rc}^{B}

59
Energy: -4118.82929921
Cu 1.267905 -0.058636 -0.936253
Cl 3.209864 -0.787446 -1.675089
C -1.922635 -0.313761 0.322250
O 0.440581 1.637201 -1.184557
S 0.855365 2.684493 -0.159175
O 1.791199 2.134914 0.824387
C 1.863981 3.805941 -1.201292
O -0.271195 3.469892 0.319184
F 2.347029 4.782463 -0.449027
F 2.866839 3.129845 -1.738366
F 1.117163 4.321885 -2.163379
C -1.840880 -0.443253 -1.071906
C -2.983408 -0.514924 -1.847492
C -4.248890 -0.463275 -1.255260
C -4.338481 -0.333736 0.132831
C -3.196896 -0.255265 0.910333
H -0.868663 -0.464381 -1.554018
H -2.879752 -0.601859 -2.927517
C -5.516271 -0.535560 -2.041706
H -5.328815 -0.292284 0.581560
H -3.285583 -0.152453 1.990014
C 1.576123 -2.726518 0.129646
O 0.661601 -2.078137 -0.448423
C 1.940002 -4.117872 -0.335469
N 2.126145 -2.196528 1.220940
C 1.772811 -0.819692 1.545403
C 0.528843 -0.501110 0.784947
C -0.732132 -0.272362 1.175471
C -0.886609 0.127552 2.607434
C -1.049087 1.472025 2.946943
C -1.223827 1.824717 4.281275
C -1.235330 0.847803 5.272698
C -1.068926 -0.492684 4.933922
C -0.891228 -0.853719 3.602806
H 2.981816 -2.588623 1.593297

```
H 1.562865 -0.723390 2.618822
H 2.601080 -0.142267 1.293209
H -1.024697 2.233774 2.165499
H -1.345284 2.873259 4.546005
H -1.372388 1.131295 6.314779
H -1.083460 -1.259423 5.706678
H -0.768322 -1.901727 3.323042
C 1.791871 -4.187508 -1.854657
C 3.368790 -4.479535 0.064866
C 0.939054 -5.068110 0.337460
H 3.621960 -5.459216 -0.358973
H 4.090378 -3.749005 -0.325863
H 3.493296 -4.571552 1.154300
H 1.142956 -6.096033 0.010139
H 1.022187 -5.035326 1.432647
H -0.092276 -4.817867 0.057866
H 2.045582 -5.200492 -2.192848
H 0.764021 -3.966801 -2.163909
H 2.458361 -3.468577 -2.347846
O -6.594653 -0.525550 -1.478433
C -5.421689 -0.620005 -3.543530
H -4.886135 0.245703 -3.955413
H -4.873502 -1.518909 -3.856173
H -6.432049 -0.652182 -3.959410
```
